# Abstracts from the 5th International Conference on Prevention & Infection Control (ICPIC 2019): Geneva, Switzerland. 10-13 September 2019

**DOI:** 10.1186/s13756-019-0567-6

**Published:** 2019-09-09

## Slide session: Surgical Site Infection

### O1 THE EFFECT OF POSTOPERATIVE CONTINUATION OF ANTIBIOTIC PROPHYLAXIS ON THE INCIDENCE OF SURGICAL SITE INFECTION: A SYSTEMATIC REVIEW AND META-ANALYSIS

#### S. De Jonge^1^, Q. Boldingh^1^, J. Solomkin^2^, P. Dellinger^3^, M. Egger^4^, G. Salanti^4^, B. Allegranzi^5^, M. Boermeester^1^

##### ^1^Surgery, Amsterdam UMC, Amsterdam, Netherlands; ^2^Surgery, University of Cincinnati , Cincinnati; ^3^Surgery, University of Washington, Seatle, United States; ^4^Institute for Social and Preventive Medicine, University of Bern, Bern; ^5^Infection Prevention and Control Global Unit, World Health Organization, Geneva, Switzerland

###### **Correspondence:** S. De Jonge

**Introduction:** Surgical antibiotic prophylaxis (SAP) is frequently continued for several days after surgery to prevent surgical site infection (SSI).Continuing SAP after the operation may have no advantage compared to immediate discontinuation and unnecessarily expose patients to risks associated with antibiotic use. In 2016, the World Health Organization (WHO) recommended discontinuation of SAP.

**Objectives:** We present an update of the evidence that formed the basis for this recommendation.

**Methods:** For this systematic review and meta-analysis we searched MEDLINE, Embase, CINAHL, CENTRAL, and WHO regional medical databases from Jan 1990 to August 2018 for randomised controlled trials (RCT) comparing the effect of postoperative SAP continuation to its discontinuation. We excluded studies comparing regimens that also differed with regard to dose and agent used, and studies that did not administer the first dose preoperatively by intravenous infusion. We extracted data from published reports and contacted the authors if important information was missing. We combined studies using random effects meta-analysis. We planned subgroup analyses and meta-regression for studies adhering to current standards of practice in SAP.

**Results:** We identified 83 relevant RCTs. The main meta-analysis included 52RCTs with 19,273 participants. The combined relative risk of SSI comparing postoperative SAP continuation with discontinuation was 0·89 (95% confidence interval: 0·79-1·00). There was little heterogeneity (tau^2^: 0·001). Subgroup analysis showed that the effectiveness of postoperatively discontinued SAP depends on appropriateness of SAP practices. When SAP best practices (i.e., timely administration of the first dose and redosing when indicated according to the procedure duration) were applied, there was no benefit of postoperative SAP continuation in reducing SSI compared to discontinuation of SAP.

**Conclusion:** There is no strong evidence for a benefit of postoperative continuation of SAP. These findings support WHO recommendations against this practice. A protocol for this review was registered with at PROSPERO:CRD42017060829.

**Disclosure of Interest**: None declared

### O2 IMPACT OF CLIMATE FACTORS ON SURGICAL SITE INFECTION RATES. DATA FROM 17 YEARS OF SURVEILLANCE IN GERMANY

#### S. J. S. Aghdassi^1^, F. Schwab^1^, P. Hoffmann^2^, P. Gastmeier^1^

##### ^1^Institute of Hygiene and Environmental Medicine, Charité – Universitätsmedizin Berlin, corporate member of Freie Universität Berlin, Humboldt-Universität zu Berlin, and Berlin Institute of Health, Berlin; ^2^Potsdam Institut für Klimafolgenforschung, Potsdam, Germany

###### **Correspondence:** S. J. S. Aghdassi

**Introduction:** Surgical site infections (SSI) are among the most frequent healthcare-associated infections in German hospitals. Besides well-known patient-related and procedure-related risk factors for SSI, a focus has been placed recently on other risk factors, including season and temperature.

**Objectives:** Our objective was to determine how selected climate factors influence SSI-rates and for which SSI-causing pathogens effects of climate factors are most noticeable.

**Methods:** SSI-rates were calculated for procedures included in the German SSI surveillance system, which were conducted in the years 2000-2016. The procedures were associated with department- and patient-related data. To investigate the impact of climate factors, data on temperature, precipitation, and other meteorological parameters provided by the German Meteorological Service were utilised. Postcodes were used to match climate data and surveillance data. Due to a high correlation with other climate factors, analyses were executed with a focus on temperature. A descriptive analysis was conducted using chi-squared test. Through multivariable logistic regression adjusted odds ratios (AOR) were calculated for SSI-rates in reference to the mean temperature (both as a categorical and a continuous variable) during the month of surgery. A p-value of <0.05 was considered significant.

**Results:** Altogether 2,004,793 procedures and 32,118 SSI (13,811 superficial and 18,307 deep) were included. Temperatures ≥20°C were associated with a significantly higher SSI-risk than temperatures <5°C (AOR 1.132). This was observed for SSI caused by gram-positive and gram-negative bacteria. This association was most prominent for superficial SSI with gram-negative pathogens (AOR 1.378). When viewed as a continuous variable, we found that an increase of 1°C in mean temperature resulted in a 0.7% higher overall SSI-risk.

**Conclusion:** Climate factors influence SSI-rates. Higher temperatures increase the risk of SSI, this effect is especially caused by temperatures ≥20°C which seem to represent a threshold. The expected rise of global temperatures until the end of the century when compared to preindustrial conditions may increase the incidence of SSI and its effect has to be recognised when developing future SSI-prevention strategies.

**Disclosure of Interest**: None declared

### O3 REDUCING STAPHYLOCOCCUS AUREUS SURGICAL SITE INFECTIONS USING AN ANTI-STAPHYLOCOCCAL BUNDLE IN NEW ZEALAND

#### N. Grae, A. Morris, S. Roberts

##### ^1^Infection Prevention & Control Programme, New Zealand Health Quality & Safety Commission, Wellington, New Zealand

###### **Correspondence:** N. Grae

**Introduction:** *Staphylococcus aureus (S. aureus)* is a major cause of cardiac and orthopaedic surgical site infections (SSIs) in New Zealand (NZ). A preoperative bundle to reduce *S. aureus* SSIs has been implemented in many countries; the evidence supporting this is of moderate quality. A quality improvement (QI) collaborative to develop and implement a preoperative anti-staph bundle to reduce *S. aureus* SSI rates in target surgical procedures was established.

**Objectives:** The aim of the collaborative was to implement an anti-staph bundle in different clinical pathways (cardiac and orthopaedic surgery) across numerous hospitals.

**Methods:** Eight publicly funded and private surgical hospitals were recruited. The ten-month collaborative included three one-day learning sessions, monthly webinars, one-to-one teleconferences, and site visits. The key components of the bundle included pre-operative skin and nasal decolonisation. The choice of agents and method of delivery was at the discretion of the respective teams. Education, auditing, and documentation tools were developed and shared across all teams.

**Results:** All hospitals fully implemented an anti-staph bundle. The bundle has been applied to over 4,700 procedures; 1977 cardiac, 2732 orthopaedic. Compliance rates for bundle implementation exceeded 95% for all sites. One year post-implementation there has been an overall 49% reduction in *S. aureus* SSI rates (p-value = 0.004); 38% cardiac (p = 0.076) and 74% orthopaedic (p = 0.006). A decrease in SSI rate [run chart] and standard statistical methods support reduction in the overall *S. aureus* SSI rate.

**Conclusion:** Universal preoperative decolonisation reduced the burden of *S. aureus* SSI in this patient group. The collaborative methodology supported networking and acquisition of QI skills by participants. It also facilitated local adaption of a bundle to suit a hospitals workflow and provided a set of protocols and tools for other NZ hospitals to use.

**Disclosure of Interest**: None declared

### O4 IMPACT OF A BUNDLE INTERVENTION TO REDUCE SURGICAL SITE INFECTION AFTER CRANIOTOMY: A BEFORE-AFTER PROSPECTIVE STUDY

#### E. Jimenez-Martinez^1^, G. Cuervo ^2^, A. Hornero^1^, P. Ciercoles^1^, C. Cabellos^3^, A. Gabarros^4^, D. Garcia-Somoza^5^, J. Carratalà^3^, M. Pujol^1^

##### ^1^Infection Control Team; ^2^Infection diseases; ^3^Infectious Disease; ^4^Neurosurgery; ^5^Microbiology , Bellvitge University Hospital, L'Hospitalet del Llobregat, Spain

###### **Correspondence:** E. Jimenez-Martinez

**Introduction:** Consequences of surgical site infection after craniotomy (SSI-CRAN) can be devastating for both the patient and the health system, given its high morbi-mortality and costs associated.

**Objectives:** To determine whether the implementation of a surgical care bundle (SCB) is effective to reduce the risk of developing a SSI-CRAN.

**Methods:** All patients undergoing craniotomy from 2013-2017 were included. Post-discharge surveillance was carried out actively up to 1 year post-surgery. Incidence of SSI-CRAN before and after the SCB implementation was measured. Main SCB measures were: preoperative shower, appropriate hair removal, 1g vancomycin powder in the subgaleal space and a sterile absorbent drape to cover the surgical wound. Given the lack of randomization, a propensity score (PS) matching 1:1 of receiving SCB was estimated.

**Results:** 1017 patients included, 595 pre-SCB period and 422 SCB period. The overall prevalence of SSI-CRAN in SCB period was lower (15.3%vs.3.5%, p<0.001). For the PS, 421 pairs were matched. Multivariate Cox PS-matched analysis of factors associated with SSI-CRAN found that SCB implementation (AOR:0.23, 95% CI:0.13–0.40; p<0.001) and CSF leak (AOR:3.93, 95% CI:1.11–12.68; p=0.025) were independently associated with this complication.

**Conclusion:** The implementation of a SCB was effective in reducing the incidence rates of SSI-CRAN in a tertiary university hospital. Hospitals should embrace strategies to increase SCB compliance.

**Disclosure of Interest**: None declared

### O5 MANAGEMENT AND OUTBREAK INVESTIGATION OF SERRATIA MARCESCENS NEUROSURGICAL SITE INFECTIONS ASSOCIATED WITH A CONTAMINATED SHAVING RAZOR USED FOR PREOPERATIVE SCALP SHAVING

#### E. J. Kim^1^, W. B. Park^2^, N. J. Kim^2^, S. J. Kim^1^, Y. R. Oh^1^, W. S. Cho^3^

##### ^1^Infection control center, Seoul National University Hospital; ^2^Department of Internal Medicine, Seoul National University College of Medicine; ^3^Department of Neurosurgery, Seoul National University Hospital, Seoul, Korea, Republic Of

###### **Correspondence:** E. J. Kim

**Introduction:** *Serratia marcescens* is an opportunistic pathogen that can cause various healthcare-associated infections. And several sources of outbreaks in hospital have been identified that include contaminated nebulizers, intravenous injection fluids and drugs, disinfectants, and soaps.

**Objectives:** This study was conducted to identify the source of the outbreak and describe the infection control measures.

**Methods:** Between October 6 and 21, 2018, 5 SSI cases with *S. marcescens* occurred in a 1786-bed tertiary care hospital in Seoul, South Korea. Epidemiologic investigation was launched with extensive environmental sampling and screening of healthcare workers and implemented the infection control measures. The whole genome sequencing of environmental samples and patient isolates was used for molecular biological analysis.

**Results:** During the outbreak periods, a total of 43 neurosurgical operations performed and 5 cases (11.6%) developed SSIs. *S. marcescens* was isolated from cerebrospinal fluid, wound, pus, tissue and blood*.* These patients were located in different wards, and their operations were performed by different surgeons in different operative room. 2 patients were adults and 3 patients were pediatrics. But they all took a preoperative scalp shaving on a barbershop in the hospital. Among the 387 surveillance samples, *S. marcescens* were detected on 2 shaving brushes and 4 razors that had been used to shave multiple patients’ hair before surgery. And the phylogenetic analysis using whole genome sequencing revealed close clustering among isolates from the razors and the 4 patients.

An infection control team recommended to stop using the razors in barbershop, ward or operating room and to let trained doctors or nurses shave scalps using disposable clippers for surgery. The outbreak ended after implementing this infection control measures.

**Conclusion:** This study found microbiological and epidemiological evidence that contaminated razors in barbershop were the source of this outbreak. As recommendations in many guidelines, proper hair removal using the disposal clippers before surgery is very important for preventing SSIs.

**Disclosure of Interest**: None declared

### O6 BARRIERS AND FACILITATORS TO IMPLEMENTING A BUNDLED INTERVENTION TO PREVENT S. AUREUS SURGICAL SITE INFECTIONS (SSI) AMONG CARDIAC AND ORTHOPEDIC SURGERY PATIENTS: WHY DID IT WORK BETTER FOR ORTHOPEDIC SURGERIES?

#### M. Schweizer^1^, S. Hockett-Sherlock^2^, E. Perencevich^1^, C. Goedken^1^, L. Herwaldt^2^, H. Reisinger^2^

##### ^1^Iowa City VA Hospital; ^2^University of Iowa, Iowa City, United States

###### **Correspondence:** M. Schweizer

**Introduction:** In a prior multicenter study, we implemented an SSI prevention bundle including: 1) *S. aureus* nasal screening, 2) chlorhexidine (CHG) bathing, 3) mupirocin decolonization for *S. aureus* carriers, 4) vancomycin & cefazolin prophylaxis for methicillin-resistant *S. aureus* (MRSA) carriers, & cefazolin for all others. The intervention decreased rates of complex *S. aureus* SSI by 42%. The decrease was larger for orthopedic compared with cardiac surgery patients.

**Objectives:** When expanding implementation to additional hospitals, we aimed to identify barriers & facilitators to bundle implementation.

**Methods:** This bundle was implemented in 11 US Veterans Affairs (VA) hospitals. Individual & group semi-structured interviews were conducted before implementation (7/2014 - 9/2014) & during/after implementation (1/2017 - 12/2017) of the bundle at 6 VA hospitals. We interviewed hospital epidemiologists, infection preventionists, surgeons, case managers, & lab directors/staff. Transcripts & field notes were analyzed for thematic content.

**Results:** 56 employees were interviewed before implementation & 51 were interviewed during/after implementation. Interviewees stated that most orthopedic patients had a pre-operative clinic visit within 30 days before surgery. At hospitals with rapid PCR testing, *S. aureus* screening was performed at the beginning of the clinic visit so that the results were known before the patient left the clinic. Thus, the patient could be sent home with mupirocin & CHG as needed. However, cardiac surgery patients did not have standardized clinic visits before surgery. Cardiac surgery patients recovering from angiography might be screened for *S. aureus* but were discharged before instructions for use of mupirocin or CHG could be provided. This intervention was most successful when a nurse championed the bundle. A barrier was that nurses were not allowed to directly order mupirocin for patients. Requiring physicians to order mupirocin & CHG was a barrier to implementation.

**Conclusion:** Decolonization agents that do not require patients to be screened for *S. aureus* or do not require prescriptions could improve implementation for cardiac surgery.

**Disclosure of Interest**: M. Schweizer Grant/Research support from: PDI Healthcare, S. Hockett-Sherlock: None declared, E. Perencevich: None declared, C. Goedken: None declared, L. Herwaldt: None declared, H. Reisinger: None declared

## Innovation Academy - The pitch

### I1 MOTION DETECTION AND ARTIFICIAL INTELLIGENCE IN THE AUTOMATIC DOCUMENTATION OF KEY FIGURES FOR HAND HYGIENE COMPLIANCE

#### E. Khaljani, MBA, S. C. Slama, T. Ebeling

##### HygNova GmbH, Berlin, Germany

###### **Correspondence:** E. Khaljani

**Introduction:** Direct observation is the gold standard in the detection of hand hygiene compliance, although known biases influence quality of data. In this study, data of motion detection sensors was examined with mathematical algorithms to assess the value of improved technological opportunities in hand hygiene monitoring.

**Objectives:** The goal of the study was to detect four properties concerning relevant parameters for hand hygiene monitoring in a laboratory setup:

1. Are patients’ beds occupied by patients or not?

2. If patients are present, is it possible to observe direct contact of healthcare professionals (HCPs) with patients as a surrogate for a moment of hand hygiene?

3. Can information be gathered without wearable devices?

4. Can data be gathered without personalization of patients and healthcare professionals?

**Methods:** Motion detection sensors were mounted over patients’ beds. Presence of patients and the treatment of healthcare professionals were recorded in an operationalized setup. 67 recordings took place which lasted between 2 to 5 minutes. In some recordings, there were duplications of observed cases (bed occupied and direct contact with patient). When HCPs were closer to patients than 10 cm, direct contact was noted. No wearable devices were used to gather data.

Data was analyzed with an algorithm, which was developed for the purpose of the study. Direct observation took place simultaneously to the recordings to validate the results of the algorithm.

**Results:** In 53 recordings, the presence of the patient in the bed had to be defined. In 52 out of 53 recordings, the presence of the patient could be detected (98,1% of cases compared to 100% by direct observation). There was one false negative case.

In 26 recordings, direct contacts of HCPs were examined. In all 26 cases, direct contacts of HCPs could be determined (100% compared to 100% by direct observation).

In no case patients’ or HCPs could be identified out of the recorded data (0% compared to 100% by direct observation). No wearable devices had to be used in the study.

**Conclusion:** The results show that algorithmic analysis of motion detection data can lead to information which can be used in the evaluation of hand hygiene compliance. Further studies will be necessary to identify if Artificial Intelligence can detect the 5 moments of hand hygiene out of motion detection data with an acceptable deviation.

**Disclosure of Interest**: E. Khaljani, MBA Shareholder of: The author is a shareholder of the HygNova GmbH which uses motion detection technology to approximate the 5 WHO moments of hand hygiene in hospitals., S. Slama Shareholder of: The author is a shareholder of the HygNova GmbH which uses motion detection technology to approximate the 5 WHO moments of hand hygiene in hospitals., T. Ebeling Shareholder of: The author is a shareholder of the HygNova GmbH which uses motion detection technology to approximate the 5 WHO moments of hand hygiene in hospitals.

### I3 SMARTRUB®: A NEW ELECTRONIC DEVICE TO MEASURE AND PROVIDE INDIVIDUAL FEEDBACK OF THE QUALITY OF HAND HYGIENE ACTION

#### Y.-A. Robert^1^, S. Fourquier^2^, Y. Martin^1,3^, D. Pires^3^, C. Guitart^3^, R. Beuchat^2^, D. Pittet^3^

##### ^1^iQati, Rolle; ^2^hepia; ^3^IPC program, Genève, Switzerland

###### **Correspondence:** Y.-A. Robert

**Introduction:** We developed a unique electronic device, “SmartRub® powered by iQati^TM”^ (SmartRub®), designed to educate and monitor the quality of individual hand hygiene (HH) actions by HCW during routine patient care. The device measures both the duration and volume of alcohol-based handrub (ABHR) used during each HH action, and provides the HCW with real-time feedback (FB).

**Objectives:** Measure and improve HH quality through individual FB

**Methods:** SmartRub® is composed of an electronic wristband and an electronic cylinder inserted into a pocket-sized ABHR dispenser. Each HCW has their own designated device. A vibration is activated in the dispenser when the HCW reaches the targeted ABHR volume customized to their hands’ surface, giving HCW FB on the volume. A vibration activated in the wristband after a defined time following the start of the HH gesture, giving HCW FB on the duration of the action. The FB can be fully parametrized, including being activated or not. SmartRub® is compatible with both rinse and gel ABHRs.

**Results:** To assess the precision of the measures, 5 HCWs performed 2200 ABHR uses and 70 HH friction gestures. The error on the volume measurement averaged -0.02 + (SD) 0.1 mL and the error on the duration -0.1+ (SD) 1.4 sec. SmartRub® was tested in a tertiary hospital for its ability (sensitivity/specificity) to detect HH actions in: (a) laboratory conditions, (b) a simulated path in a clinical ward, and (c) real-life conditions in a clinical ward (overall sensitivity to detect “true” HH actions, 96.8%; specificity, 98.3%). The effect of SmartRub® has been demonstrated in a clinical trial; the quality of the HH gestures improved when FB was used: the average volume of ABHR used increased from 1.4 mL to 2.1 mL and duration of friction increased from 9.3 to 11.1 sec. SmartRub® acceptability was tested among HCWs; the majority found it easy to use and useful; half of them affirmed its use made them change their HH behaviour.

**Conclusion:** SmartRub® allows HCWs to know in real-time whether the quality of their HH action is appropriate in terms of volume of ABHR used and duration of friction. It encourages them to improve their own performance continuously, thus helping to implement behavior change.

**Disclosure of Interest**: None declared

### I4 EFFECTIVENESS OF WHATS APP IN PROMOTING HAND HYGIENE AWARENESS PLEDGES AMONG HEALTH CARE PERSONALS IN INDIA

#### D. Sureshkumar^1^, S. Saravanakumar^2^, J. Hemalatha^3^

##### ^1^Infectious Diseases, Best of IDs, Chennai; ^2^Medicine, KMC Manipal, Mangalore; ^3^Pharmacy, Best of IDs, CHENNAI, India

###### **Correspondence:** D. Sureshkumar

**Introduction:** Infection prevention and control organizations encourages health care personals(HCPs) to take a pledge on infection prevention topics such as hand hygiene (HH) to create awareness & actively engage them in infection prevention. Social networking services like WhatsApp are increasingly used by HCPs and organizations to share medical knowledge and to disseminate health information to wider HCP community. However, research is limited on the effectiveness of WhatsApp in promoting HH awareness pledge among HCPs.

**Objectives:** Our objective was to evaluate the effectiveness of WhatsApp in promoting HH awareness pledge among HCPs.

**Methods:** This observational study was conducted from May 5 to May 19, 2019 among HCPs belonging to randomly selected different WhatsApp networks operating from India with majority of members engaged or working in Indian health care industry. In the first part of study from May 5 to May 12 education on hand hygiene and the importance of promoting hand hygiene within health care were circulated daily. In the second part from May 12 to May 19, members were encouraged to take HH pledge with periodic daily remainders. The rate of HCPs taking HH awareness pledge was calculated in percentage and reasons for not taking HH pledge were analyzed.

**Results:** 1111 HCPs belonging to nine WhatsApp networks participated in this study. Majority (718/1111, 3/9 Groups) of the members were belonging to physicians WhatsApp groups. The effectiveness of HH awareness pledge were highest among Infectious disease (ID) physicians (13/23, 56.52%) group followed by hospital based groups (38/115, 28.14%) & Clinical microbiologists (50/187, 26.73%). The HH WhatsApp awareness pledge campaign failed to create awareness among ID trainees and physicians networks in this study.

**Conclusion:** The effectiveness of WhatsApp networks HH awareness pledge campaign was 13.27% (129/972) among different HCPs WhatsApp groups in India. The reasons for high acceptance to HH awareness pledge in certain groups and failure to create awareness in other groups need to be analyzed in detail to identify ideal WhatsApp networks for future infection control & HH awareness campaigns.

**Disclosure of Interest**: None declared

### I5 SMART MONITORING OF HAND HYGIENE

#### T. Gebhardt

##### GWA Hygiene, Stralsund, Germany

###### **Correspondence:** T. Gebhardt

**Introduction:** We believe that monitoring the hand hygiene behavior should be done automized and objectively. These characteristics have been considered during the development of the hand hygiene monitoring "NosoEx".

**Objectives:** It is the overall goal to reduce hospital-acquired infections. Since our hands are the transmission path number one for germs awareness for hand hygiene must be increased. Direct feedback to healthcare workers is essential because they often overerstimate themselves or do not feel addressed during hygiene trainings. Consequently, NosoEx provides data about the status quo and the development of the hand disinfection numbers over time. This data can be divided into job groups like doctors, nurses and therapists. Ultimately, hygiene trainings can be performed more directly.

**Methods:** Every existing dispenser model (wall dispensers, point of care dispensers, individual bottles of hand rub) can be upgraded with NosoEx sensors. Moreover, every healthcare worker gets a badge that can be worn at the clothing. The badges have different colors that represent the job groups. Associated with that, the compliance with data protection laws and work councils is ensured. In addition to that, all collected data is visualized in a user-friendly software. There the hospital ward map is digitized and the position of dispensers are shown. Consequently, additional key figures like filling level and use frequency can be seen for every dispenser.

**Results:** Our client "Hospital Lüneburg" increased the disinfectant use by 35% after NosoEx has been installed. This was important because the selected wards have been under average. Moreover, data patterns have been identified. For example, the use of dispensers on the corridor has been much higher than the use of dispensers in the patient room. The recommendation was that this area of the ward should be analyzed more deeply during the compliance observation. In addition to that, the NosoEx system has been recognized that certain job groups did not reach the minimum amount of 3ml per disinfection. Followed by that, trainings about the correct hand disinfection have been realized.

**Conclusion:** Hand hygiene monitoring solutions have to be seamlessly integrated into the infrastructure of the hospital and into the job routines of the healthcare workers. Direct feedback with job-group related data can be more sustainable. Moreover, providing the data automized might reduce the workload of data analysis that is done by infection control officers.

**Disclosure of Interest**: None declared

### I6 DEVELOPMENT OF 1,3,5-TRIAZINE–PYRAZOLE AS EFFECTIVE HAND-WASH WITH POTENT ANTIBACTERIAL ACTIVITY

#### U. P. Singh

##### Department of Pharmaceutical Sciences, Sam Higginbottom University of Agriculture, Technology & Sciences, Allahabad, India

###### **Correspondence:** U. P. Singh

**Introduction:** Hands are the most vulnerable part of body involved in the transmission of microbes and infections. Therefore, hand hygiene is the most crucial step to prevent further transmission of germs, and bacterial organisms to prevent the infections.

**Objectives:** The present study was conducted to evaluate the efficacy and antibacterial activity of 1,3,5-triazine–pyrazole as hand sanitizer.

**Methods:** The compound was developed as hand-wash using surfactant and tested for antibacterial efficacy, viz. zone of inhibition (ZOI) against *Staphylococcus aureus*, *Salmonella*, and *Escherichia coli* using dip well Agar Diffusion Technique. Antioxidant activity (DPPH assay) was also conducted to determine the effect on oxidative stress. The product was subjected to storing at different temperature conditions like 40 °C, 25 °C & 37 °C for 4 weeks for stability testing. The effect of active ingredient of hand-wash was evaluated for inhibition of DNA Gyrase via molecular docking with 3D crystal structure of DNA Gyrase enzyme.

**Results:** The formulated compound showed excellent antibacterial activity tested organism with ZOI with lowest against *E. Coli* (1.2 mm) and widest against *S. aureus* to 4.3 mm, with MIC= 3 - 34 μg/ml against tested organism. The hand-wash showed excellent inhibition of oxidative stress with 87.32%. The compound showed excellent inhibition of DNA Gyrase enzyme via interacting with Glu58 and Ile186 of DNA Gyrase with Ki = 12.34 μM. The hand-wash was found to significantly stable over the period of tested duration, in indicated by no change in color and no phase separation.

**Conclusion:** The developed 1,3,5-triazine-pyrazole hold promise for prospective hand-sanitizer due to excellent antibacterial activity.

**Disclosure of Interest**: None declared

### I7 A NOVEL OPEN-SOURCE INNOVATION FOR HAND-HYGIENE MONITORING AND COMPLIANCE USING INTERACTION DESIGN (ID): CAPACITY FOR GLOBAL SCALING UP INTERNET OF THINGS (IOT) TECHNOLOGY

#### M. J. Blaak^1^, R. DiMaio^1^, R. Sweetzir^2^, C. Betuzzi^3^, J. Vayalumkal^3^, C. Dowler^3^, K. McIntyre^3^, J. Kaufman^1^, J. Kupis^1^, J. Conly^1,3^, G. Hallihan^1^

##### ^1^W21C, University of Calgary, Calgary; ^2^Cisco Systems, Edmonton; ^3^Alberta Children's Hospital, Alberta Health Services, Calgary, Canada

###### **Correspondence:** M. J. Blaak

**Introduction:** We demonstrated that interactive, real-time visualizations (RtV) of alcohol-based rub (ABR) dispenser Frequency of Use (FoU) could improve hand hygiene compliance (HHC).^1^ ABR dispensers, with added sensor technology, allowed FoU transmission to visualization platforms visible to staff and patients.

**Objectives:** Create an open-source, low cost, scalable solution with improved reliability, capable to integrate with enterprise networks. We sought to extend RtV capabilities, develop administrative dash-boards of aggregate data, enhance performance, and improve battery life.

**Methods:** An iterative design process was used, with a multidisciplinary team of engineers, physicians, infection control professionals, and human factors specialists in a pediatric hospital unit (PHU). A 16-week trial was set-up to evaluate: 1) HHC and FoU with and w/o RtV, 2) hardware/software performance and 3) maintenance burden.

**Results:** We created a modular network of smart ABR dispensers, using local Wi-Fi network and a MQTT messaging protocol. The current iteration has 11 unique ID pediatric themes (e.g., dinosaurs, animals) for RtV. Data dashboard implementation was achieved and is accessible on any device (IoS, Android, Hospital Network) capable of achieving IoT capability. Independence of the innovation will be assessed by its ability to be maintained by the PHU team without technical assistance. Preliminary data shows trends of increased use of ABR dispensers when RtV is present.

**Conclusion:** We have created an open source, extensible, IoT ready innovation, which can be scaled to a health system’s network and context, at a fraction of the cost of commercially available systems offering similar functionality, making the innovation inclusive to low- and middle income countries. This low maintenance innovation allows for modular growth within established IT networks. Further development has the potential to expand on the utility of the data captured and promote the use of ABR dispensers. All documents relating to the innovation are publically available on GitHub.

**References**

1. Kupis, J, et al. Abstracts from ICPIC 2017. ARIC 2017, 6(Suppl 3):O22

**Disclosure of Interest**: None declared

### I8 POLYMER METALLISATION VIA COLD SPRAY AND 3D PRINTING FOR THE MITIGATION OF NOSOCOMIAL INFECTION TRANSMISSION

#### M. D. I. Lucas^1^, I. Botef^1^, S. van Vuuren^2^

##### ^1^Faculty of Engineering and the Built Environment, School of Mechanical, Industrial and Aeronautical Engineering; ^2^Department of Pharmacy and Pharmacology, Faculty of Health Sciences, University of the Witwatersrand, Johannesburg, South Africa

###### **Correspondence:** M. D. I. Lucas

**Introduction:** Touch-contact transmission of pathogens promotes the aggressive spread of nosocomial infections. Additive manufacturing (AM) techniques and metals with known antimicrobial properties may offer a complementary solution to current disinfection practices.

**Objectives:** To characterise the antimicrobial activity of AM developed coatings to combat nosocomial infection transmission.

**Methods:** Polymer metallisation via cold spray and 3D printing was used to develop various copper, silver and zinc coatings. Two independent, *in vitro* assays were conducted using the prevalent pathogens: *Staphylococcus aureus* (ATCC 25923), *Enterococcus faecalis* (ATCC 29212), *Pseudomonas aeruginosa* (ATCC 27853), *Klebsiella pneumoniae* (ATCC 13887) and a yeast *Candida albicans* (ATCC 10231); as well as the associated resistant strains: gentamicin-methicillin-resistant *S. aureus* (ATCC 33592), *P. aeruginosa* (DSM 46316) and a clinically resistant *C. albicans*. A diffusion assay evaluated coating efficacy based on the extent of inhibition zones, while an adapted time kill assay simulated pathogenic exposure via touch-contact in a dry environment, evaluating the rate of antimicrobial efficacy.

**Results:** Effective, particle-embedded, cold spray coatings were achieved. Against standard pathogens copper-zinc blended coatings exhibited synergistic activity under diffusive test conditions; while under touch-contact conditions copper coatings repeatedly achieved complete microbial elimination within a 15 min. exposure period on polymer substrates and within 7 min. on copper substrates. The addition of 5wt% silver to the latter coating brought microbial elimination down to just 5 min., against the resistant pathogens. Copper metal, in comparison, achieved 92.9% and 85.4% microbial reduction for each respective pathogen type over 3 hrs.

**Conclusion:** The innovative and adaptable surface coating technology showed enhanced antimicrobial activity and so, its potential use for the mitigation of surface contact transmission of infections was confirmed.

**Disclosure of Interest**: None declared

### I9 OZIRES: AN EX-TOY ROBOT THAT BECAME A PROFESSOR WHO TEACHES HEALTHCARE WORKERS HOW, WHEN, AND WHY WASH THEIR HANDS

#### B. Couto^1^, I. Oliveira^2^, B. Mendes^2^, M. Nogueira^3^, M. Peixoto^3^, M. Vrandecic^3^, C. Starling^4^

##### ^1^Biobyte Sistemas Ltda; ^2^UniBH; ^3^Biocor; ^4^Infection Control Ltda, Belo Horizonte, Brazil

###### **Correspondence:** B. Couto

**Introduction:** We are a team that fights against nosocomial infections. We always argued why people don’t wash their hands! One such answer was that washing hands is so unsophisticated gesture, without any technology, that people just don't do it. So, we imagine a robot in our team, to help healthcare workers clean their hands. But, how to do that, they were so expensive! Then, in 2017 we met a US$ 200 toy robot (http://www.meccano.com/meccanoid-about): 122 cm tall programmable humanoid robot with voice commands.

**Objectives:** a) How to adapt a toy robot to be an instrument of health training and continuous education of healthcare workers? b) What is the effectiveness of the use of the robot on the compliance with hand hygiene?

**Methods:** After some adaptations, the robot has changed! We gave him a name (Ozires), a spy camera, a better audio system, an alcohol gel dispenser, a mini-projector, and a purpose: the ex-toy robot became a Professor involved with hand hygiene campaigns. The mini projector allows video lessons even in small rooms. Ozires, accompanied by infection control practitioners, performs short video-lecture presentations and own reports of the institution's data regarding infections and the hand hygiene rate, working from 10 to 15 minutes in each target sector.

**Results:** After the insertion of Ozires in Hospital A, hand hygiene rate increased from about 36%, between January and July, to 65% after August/2016. Hospital B: Ozires started his lectures in May/2018. Hand hygiene adherence increased from about 23%, between July-Dec/2017, to 60% after July/2018.

**Conclusion:** We succeeded in adapting a toy robot as training instrument of healthcare workers, creating a new education tool, a robot tutor. Hand hygiene compliance raised significantly after the intervention in both hospitals. Now, we are working in how to change the robot processor to another one like Raspberry Pi to connect him to an artificial intelligence like IBM Watson. One day... one dream: Ozires participating of antimicrobial stewardship!

**Disclosure of Interest**: None declared

### I10 DOES THE APPLICATION OF A SILICON DIOXIDE NANOCOATING FACILITATE THE CLEANING IN PATIENT ROOMS?

#### D. Jouck^1^, K. Magerman^1,2^, L. Waumans^1,2^, A. Forier^1^, M. Blommen^1^

##### ^1^Department of Infection Prevention and Control; ^2^Clinical Laboratory, Jessa Hospital, Hasselt, Belgium

###### **Correspondence:** D. Jouck

**Introduction:** In recent years, more attention has been paid to the importance of the environment in the transmission of micro-organisms from one patient to another. Applying a silicon dioxide (SiO2) nanocoating, with particles ≤1 millionth of a millimeter, could facilitate the cleaning of surfaces by making the surface antistatic and hydrophobic, preventing water and dirt from adhering.

**Objectives:** This study aimed to determine whether nanocoated patient rooms with SiO2 achieve better cleaning results compared to non-coated patient rooms.

**Methods:** After a thorough cleaning, a SiO2 nanocoating was applied to different surfaces of two patient rooms. Among other things, parts of the floor, bedside table, toilet flush button, toilet seat, medical trolley and light switch were provided with a SiO2 nanocoating. In order not to influence the cleaning results, the cleaning staff were not informed about which rooms were coated. The SiO2 nanocoating itself was also invisible to the naked eye.

One week, 4 weeks and 12 weeks after applying the SiO2 nanocoating, the surfaces on the coated and uncoated patient rooms were checked by 2 measuring methods. First, the degree of contamination of the surface was determined by means of ATP measurements (Hygiena Ultrasnap™ for surface tests in combination with a Hygiena systemSURE II device). The result was expressed in relative light units (RLU). Subsequently, a determination of the total aerobic bacterial count was performed by sampling with a non-selective nutrient medium (RODAC™). This result was expressed in the number of colony forming units (CFU).

The measurements and sampling were always carried out immediately after the final cleaning when the patient was allowed to leave the hospital.

**Results:** A total of 206 measurements were made. The results of the coated patient rooms were not significantly different from the patient rooms where no SiO2 nanocoating were applied (chi squared test, p = .379 for CFU and p = .204 for RLU).

**Conclusion:** The added value of applying the SiO2 nanocoating could not be demonstrated in this study. However, this needs to be further investigated, before general conclusions are drawn.

**Disclosure of Interest**: None declared

## Slide session: Burden of healthcare-associated influenza

### O7 SEASONAL NOSOCOMIAL INFLUENZA INFECTION: A PROSPECTIVE 13 YEARS SURVEILLANCE AMONG PATIENTS AND HEALTHCARE WORKERS IN LYON, FRANCE

#### L. Henaff^1^, V. Escuret^2^, P. Vanhems^1,3^

##### ^1^Laboratoire des Pathogènes Emergents, Epidémiologie et Santé Internationale UMR_S1111, UMR5308, Centre International de Recherche en Infectiologie; ^2^Laboratoire de virologie, Groupement Hôpitaux du Nord; Centre National de Référence des virus des infections respiratoires; ^3^Service Hygiène, Epidémiologie et Prévention, Hospices Civils de Lyon, Hôpital Edouard Herriot, Lyon, France

###### **Correspondence:** L. Henaff

**Introduction:** Whereas the incidence of seasonal influenza infections in healthcare settings is underestimated, the risk of Nosocomial Influenza (NI) outbreaks is real.

**Objectives:** The objective of this prospective surveillance study was to describe epidemiological characteristics of patients and healthcare workers (HCWs) with Influenza Like Illness (ILI) and compares NI and community-acquired influenza (CAI) over 13 influenza seasons.

**Methods:** Patients and HCWs were included during influenza seasons (October-April 2004-18) in 46 wards (medicine, surgery, geriatric), in a 1000 beds hospital (Edouard Herriot university-affiliated Hospital, Lyon). A nasal swab was obtained and analyzed by reverse transcriptase polymerase chain reaction in order to detect influenza viruses. An ILI case was defined as temperature ≥37.8° C without prior use of antipyretics, and/or cough or sore throat. A NI was considered if symptoms appeared at least 72 h after admission while CAI was defined if symptom onset until 24 h after admission.

**Results:** Overall, 837 patients and 274 HCWs were included. 76.9% of ILI patients were hospitalized in geriatric ward, 20.9% in medical ward and 2.2% in surgical ward. Baseline characteristics of these patients were different according to the hospitalization ward. The sex ratio was 1.25 in surgical *vs* 0.52 in geriatric ward. Influenza vaccine coverage was 55.4% in geriatric *vs* 27.8% in surgical ward.

A total of 247 people (22%, 188 patients, 59 HCWs) were influenza laboratory-confirmed. Among patients, 59 (31.4%) had NI with a median symptom onset of 12 days; 18.6% presented their symptoms 3 to 5 days after admission and 16.9% between 46 and 71 days. Influenza vaccine coverage among NI cases was 37.9% *vs* 42.9% for CAI cases. All-cause mortality rate was 3.4% in NI cases *vs* 3.6% in CAI cases.

**Conclusion:** The results demonstrated the importance of implementing surveillance of influenza in hospitals to estimate the proportion of NI and to emphasize the need to improve infection control measures implementation and vaccine use for high-risk patients and HCWs.

**Disclosure of Interest**: None declared

### O8 HOSPITAL-BASED SURVEILLANCE OF INFLUENZA IN SWITZERLAND – A PILOT STUDY

#### A. Iten^1^, A. Thiabaud^2^, N. Troillet ^3^, L. Senn^4^, D. Flury^5^, S. Kuster^6^, C. Balmelli^7^, C. Gardiol^8^, L. Kaiser^9^, O. Keiser^2^

##### ^1^Infection Control, HUG; ^2^Institut de Santé Globale, Université de Genève, Geneva; ^3^Institut Central , Hôpital du Valais, Sion; ^4^Centre Hospitalier Universitaire du Canton de Vaud, CHUV, Lausanne; ^5^Kanstonspital Sankt Gallen , Sankt Gallen; ^6^Universitätsspital Zürich, Zürich; ^7^Ente Ospedaliero Cantonale, Bellinzona; ^8^Office fédéral de la santé publique, Bern; ^9^Maladies infectieuses, HUG, Geneva, Switzerland

###### **Correspondence:** A. Iten

**Introduction:** Until 2018, the national reporting system for influenza in Switzerland was twofold with: 1) voluntary reports of influenza-like illness (ILI) by selected primary care clinicians. 2) weekly reports of laboratory-confirmed cases. No national surveillance system existed for hospitals. With support from the Federal Office of Public Health (FOPH), we developed a pilot study for hospital-based influenza cases in Switzerland.

**Objectives:** To test the computerized system developed for influenza surveillance in Swiss hospitals

**Methods:** Three university hospitals and three cantonal hospitals participated. Data collection followed WHO recommendations using a standardised questionnaire (demographic data, information on the influenza episode, optional information about the patient’s health). Data are collected by study team of the participating sites in a secure REDCap database. Data quality checks and descriptive analyses were done weekly, and results were reported back.

**Results:** From 01.11.2018 to 24.05.2019, 1705 cases of influenza were announced. Site 3 declared 34.7% of cases, Site 4 16.8%, Site 6 15.5%, Site 2 14.7%, Site 5 12%, and Site 1 6.3%. The Influenza epidemic started during the week 2018-47 in Western Switzerland, and three to four weeks later in other sites. Most patients were old adults (67.2% over age 65). The majority of cases (98.5%) was due to Influenza A; Influenza B was reported in 24 patients. Most cases were diagnosed in medicine (51.3%) and geriatrics (11.5%). The proportion of nosocomial cases was 30% during the beginning of the season, and decreased to 20% in recent weeks, with variation between sites.

**Conclusion:** Our pilot system allowed us to get a better understanding of the morbidity and spread of severe influenza cases in Switzerland. Simplification of the questionnaire, direct import of existing data, automated analysis, and additional tools for epidemic management will help to reduce the workload and ensure that all data are entered in time. Inclusion of other hospitals is needed.

**Disclosure of Interest**: None declared

### O9 STUDY ASSESSING THE BURDEN OF INFLUENZA IN NURSING HOMES RESIDENTS WITH INFLUENZA-LIKE ILLNESS DURING 2016-2017 AND 2017-2018 INFLUENZA SEASON

#### L. Qalla-Widmer, D. Héquet, C. Petignat

##### Unité cantonale HPCI Vaud, Lausanne, Switzerland

###### **Correspondence:** L. Qalla-Widmer

**Introduction:** Influenza is a significant cause of morbidity and mortality in elderly. They are at high risk of complications after influenza virus infection. Data on the epidemiology of influenza within nursing homes (NH) are limited.

**Objectives:** The purpose of this prospective study was to better describe the burden of influenza among residents of NH of canton of Vaud, Switzerland, with influenza-like illness during 2016-2017 and 2017-2018 influenza seasons.

**Methods:** First, we determined the proportion of influenza-like illness due to influenza in NH residents. We specifically assessed the impact of a positive influenza PCR on clinical features, morbidity and mortality, 30 and 90 days after diagnosis, as compared to a negative influenza PCR. Moreover, influenza vaccination rates of the residents and the healthcare workers within each nursing home were assessed at the end of each influenza season.

**Results:** A PCR test was performed on 509 residents from 61 NH. 227 influenza virus infections were diagnosed; 181 influenza A and 46 influenza B. Compared to residents without influenza virus infection (IVI), residents with IVI were more often feverish with a high fever (69.1% and 88.5% respectively, p<0.0001) are significantly more frequently hospitalized within 30 days after diagnosis (17.6 % vs 7.1%, p=0.0003). Any cause mortality at 30 days was similar in both groups (12.8% vs 10.6%, p=0.48). Only 18.1% of IVI residents were treated with an antiviral and 60.4% of them received antibiotics. Influenza vaccination rates of the healthcare workers and residents were respectively 50% and 82%.

**Conclusion:** During influenza season, the feverish residents should be suspected to have influenza virus infection. Residents should be diagnosed (PCR) and treated with an antiviral where appropriate to limit the risk of hospitalization. Healthcare workers should be encouraged to be vaccinated against influenza in order to acquire a better herd immunity within the NH which will limit the spread of influenza.

**Disclosure of Interest**: None declared

### O10 HEALTH-CARE ASSOCIATED INFLUENZA PREVENTION PROJECT (HAIP): FIRST MULTICENTER SURVEILLANCE RESULTS (NOSOCOMIAL INFLUENZA AND PREVENTION MEASURES

#### D. Flury^1^, J. Notter^1^, R. Kuhn^1^, D. Nicca^2,3^, M. Schlegel^1^ on behalf of HaIP-Team

##### ^1^Division of Infectious Disease and Infection Control, Kantonsspital St.Gallen, St.Gallen; ^2^Institut of Nursing Science , University Basel; ^3^Ressort Pflege/MTT, University Hospital Basel, Basel, Switzerland

###### **Correspondence:** D. Flury

**Introduction:** Widely accepted definitions in the area of influenza prevention measures on a hospital level are sparse but urgently needed to compare data between hospitals.

**Objectives:** The Health-care associated Influenza Prevention Project (HAIP) is a multicentre project aiming to i) evaluate the burden of nosocomial influenza and the adherence with preventive measures and ii) measure the effectiveness of a complex prevention intervention. Here we present outcomes on nosocomial infection and adherence to preventive measures of two influenza seasons 2017-18 and 2018-19.

**Methods:** A manual with definitions for nosocomial influenza, adherence measurements for patient isolation, vaccination rate, antiviral treatment, hand-hygiene, wearing masks and cough etiquette was developed, tested at a pilot hospital and rolled out after education and teaching in 4 additional (1private, 3public) secondary and tertiary hospitals in German-speaking Switzerland (145’929 admission/year.) Quality control was done for hand-hygiene adherence (interobserver variability) and for influenza diagnosis (sensitivity and specificity for testing) by a point prevalence study on selected wards.

**Results:** Rates of nosocomial infection overall in the two season were 2.02/10000 respective 2.32/10000 patient-days during influenza season and 1.24/1000 respective 1.44/1000 admissions during influenza season. Rate of isolation 98% (interhospital range (IR) 91-100%)in the first, and 99% (IR 91-100%) in the second season, antiviral treatment 55% (IR 23-96%) respective 76% (IR 33-100%); adherence with hand-hygiene was 76% in both season, adherence with cough etiquette overall 87% in both season, adherence with mask wearing 96% in the first and 92% in the second season, vaccination rate overall was low in both season. Quality control data showed an interobserver variability of 66% (95%CI 60-72%) and accuracy of influenza-diagnostic showed a sensitivity of 81% and a specificity of 94%.

**Conclusion:** A harmonised influenza-surveillance is feasible but needs precise instructions, teaching/manual and a regular feedback. Differences in nosocomial infections depend on influenza season due to the virus itself but also on the extend of surveillance and the application of the influenza-protection measures.

**References**

**Disclosure of Interest**: None declared

## Slide session: Social aspects in infection control implementation

### O11 SOCIAL NETWORK ANALYSIS ON THE INFLUENCE EXERTED BY CHANGE AGENTS TO DIFFUSE FAVOURABLE HH BEHAVIOUR IN A DEVELOPING COUNTRY

#### Y. F. Lee^1,2^, W. Zingg^3^, M. McLaws^4^, L. M. Ong^5^, S. A. Husin^1^, H. H. Chua^6^, S. Y. Wong^6^, D. Pittet^3^

##### ^1^Ministry of Health, Kuala Lumpur, Malaysia; ^2^Institute of Global Health, Geneva University; ^3^Infection Control Program and WHO Collaborating Centre on Patient Safety, University of Geneva Hospitals and Faculty of Medicine, Geneva, Switzerland; ^4^School of Public Health and Community Medicine, University of New South Wales, Sydney, Australia; ^5^Clinical Research Centre & Department of Medicine, Penang; ^6^Sarawak General Hospital , Kuching, Malaysia

###### **Correspondence:** Y. F. Lee

**Introduction:** Based on the Diffusion of Innovation Theory, we hypothesised that peer-identified as change agents (CAs) were early adopters who would act as role models to improve hand hygiene (HH) compliance through their social network.

**Objectives:** To explore the different influence for favourable HH behaviour exerted by peer-identified CAs and management-selected change agents (MSCAs), and desirable attributes of HH leadership within a social network.

**Methods:** The intervention was conducted in two acute medical wards at Sarawak General Hospital, Malaysia; one with peer-identified CAs, and one with MSCAs. The primary outcome was HH compliance. The secondary outcomes measured (i) desirable HH leadership attributes using questionnaires (ii) opinion of ward staff towards peer-identified CAs and MSCAs through question & answer sessions, and (iii) social network connectedness of healthcare workers (HCWs) for HH improvement using NodeXL Pro software.

**Results:** Both wards achieved more than 10 percentage point improvement in HH compliance. Healthcare workers on both wards believed ‘strictness’ was the most desirable attribute of HH leadership. Healthcare workers reported peer-identified CAs led by example while MSCAs were authoritative. The distance between HCWs seeking HH advice from their nominated leaders (NLs) were two individuals. Ties between all HCWs were low, and nominations between HCWs to a NLs were low on both wards. Years of working experience of the top five most powerful NLs on both wards were similar.

**Conclusion:** Leadership by peer-identified CAs achieved equally improved HH as MSCAs lead wards and were regarded as early adopters. Close connectedness of staff with NLs, low ties and low nominations between individual HCWs suggests both wards have a hierarchical organisational structure. The closeness of HCWs with their leaders is common in hierarchical leadership structures, as was the preference on both wards for strict role models, possible reflecting local cultural norms.

**Disclosure of Interest**: None declared

### O12 PATIENT PARTICIPATION IN HEALTHCARE WORKERS’ HAND HYGIENE AUDIT: AN EXPLORATORY STUDY

#### L. Côté^1,2^, M. C. Gallani^3,4^, Y. Longtin^5^

##### ^1^Infection prevention and control service, Institut universitaire de cardiologie et de pneumologie de Québec; ^2^Faculty of Medicine; ^3^Faculty of Nursing, Université Laval; ^4^IUCPQ-UL Research Center, Institut universitaire de cardiologie et de pneumologie de Québec - Université Laval, Québec; ^5^Faculty of Medicine, McGill University, Montréal, Canada

###### **Correspondence:** L. Côté

**Introduction:** Hand hygiene (HH) is the most important measure to prevent hospital-acquired infections. Audit of HH practices is a key step towards its improvement. The World Health Organization suggests that patients can perform HH audit, but few studies have explored this avenue.

**Objectives:** Exploratory study among bariatric surgery patients in a tertiary care institute in Québec (Canada) to explore: 1) the feasibility of involving patients to audit healthcare worker’s (HCW) HH practice “before contact with the patient or their environment” (acceptance, competence, performing the behavior), 2) psychosocial variables associated with auditing behavior, and 3) patient-auditors experience.

**Methods:** In phases A (n=14) and B (n=25) of the study, patients were trained to audit HCWs’ HH. Following verification of their competency, they performed audits over a 24-hour period. In phase B, the behavioral determinants and auditors’ experience were measured with validated questionnaires developed for this study.

**Results:** The majority of patients agreed to participate (43/79; 54%), demonstrated competence to perform audits (32/39; 82%) and performed ≥ 1 HH audit (32/33; 97%). Patients performed on average 8,6 ±6,2 observations. The main barrier reported was incapacity to visualize HCW behaviour in the corridor while the auditor is in the room. Moral norm and perceived behavioral control accounted for 86% of the variability of the intention to perform the audit (p<0.001, R^2^=86%). The level of education accounted for 50% of the variability in behavioral frequency (p=0.002, R^2^=50%). 94% (30/32) of patients reported a positive overall experience. 80% (16/20) found the audits easy to accomplish and felt comfortable auditing HCWs. Most (14/15; 93%) reported that auditing did not modify patient-HCW relationship and 85% (17/20) reported no negative impact on their perception of the quality of care.

**Conclusion:** Patients can be involved as prospective auditors of HCW’s HH and their overall experience is positive. These results could be taken into account when planning future interventions involving patient-auditors.

**Disclosure of Interest**: None declared

## Slide session: Infection control surveillance revisited

### O14 WORKLOAD INDICATORS OF STAFFING NEED AS A TOOL TO DETERMINE INFECTION CONTROL STAFFING IN HOSPITALS: AN OBSERVATIONAL STUDY

#### L. Wundavalli^1^, U. S. Agrawal^2^, S. Satpathy^3^, B. R. Debnath^2^, T. A. Agnes^2^

##### ^1^Hospital Administration, North Eastern Indira Gandhi Regional Institute of Health and Medical Sciences, Shillong; ^2^All India Institute of Medical Sciences, Delhi, India; ^3^Hospital Administration, All India Institute of Medical Sciences, Delhi, India

###### **Correspondence:** L. Wundavalli

**Introduction:** Staffing as a ratio of infection control professionals to inpatient beds does not take into account the complex nature of the work and the varying degree of acuity and risk in different care settings.

**Objectives:** To calculate the staffing requirement for the infection control unit of a cancer hospital using World Health Organisation's Workload Indicators of Staffing Need (WISN).

**Methods:** Descriptive study conducted for a period of six months. Hospital: unit of study and type of infection control activity: unit of sampling. Interviews were held with infection control nurses to list all their activities. Unit time taken for each activity was observed. Records were perused to obtain annual workload statistics. WISN was used to calculate the nurse requirement.

**Results:** We identified 14 broad category infection control activities, miscellaneous support activities and 6 additional activities to be performed by 4 infection control nurses with a total available working time of 6132 hours for an annual load of 6238.25 (+/-372) hours for a 182 bedded cancer hospital with 69,331 annual admissions. 78% of the time was spent on core infection control activities and 22% on other support and additional activities. 44% of the time was spent on active surveillance. 56% of the time was spent on education. WISN ratio for the current workload is 1. If active surveillance is included, the WISN ratio is 0.75. One additional nurse will be required to implement active surveillance.

**Conclusion:** WISN can measure all infection control activities and translate workload into nursing full time equivalents. The step by step elucidation of the WISN tool in this paper may serve as a reference for manpower planning.

**References**

*Am J Epidemiol* 1980;111

Storr J, Twyman A, Zingg W, Damani N , Kilpatrick C , Reilly J, et al and the WHO Guidelines Development Group.Core components for effective infection prevention and control programmes: new WHO evidence-based recommendations. *Antimicrobial Resistance & Infection Control* 2017; 6:6.

Bartles R, Dickson A, Babade O. A systematic approach to quantifying infection prevention staffing and coverage needs. *Am J Infect Control* 2018; 46(2018):487-491

Workload Indicators of Staffing Need User’s Manual. World Health Organisation.

**Disclosure of Interest**: None declared

### O15 REDEFINING WHOLE GENOME SNP THRESHOLDS FOR DISTINGUISHING PATHOGENIC BACTERIAL ISOLATES IN CLINICAL SETTINGS

#### A. Shelenkov^1^, D. Shagin^1^, Y. Mikhaylova^1^, Y. Yanushevich^1^, V. Fomina^2^, V. Akimkin^1^

##### ^1^Central Research Institute of Epidemiology; ^2^Pirogov National Medical and Surgical Center, Moscow, Russian Federation

###### **Correspondence:** A. Shelenkov

**Introduction:** To effectively control the pathogenic bacteria spread in hospital settings it is critically important to develop accurate and reliable protocols of isolates’ comparison between different patients and departments. However, traditional typing methods may be insufficient in the cases of multi-drug resistant strains with high level of genome variability.

**Objectives:** Facilitate the developing of a reliable procedure for distinguishing pathogenic bacteria strains based on their whole genomes.

**Methods:** 6 isolates of *Klebsiella pneumoniae*, 5 of *Pseudomonas aeruginosa* and 4 of *Staphylococcus aureus* were obtained from peripheral blood, urine and soft tissues of 81-year old female patient of intensive care unit in Moscow hospital during the period of four months. Genomic DNA was isolated with DNeasy kit (Qiagen) and used for paired-end library preparation with Nextera Kit (Illumina) and sequencing on Hiseq platform. Genome assemblies were made using SPAdes program and genomic comparison were performed using roary, dnadiff and custom software. The isolates were classified using MLST-based scheme and, in addition, capsular gene scheme (K- and O-loci) for *K. pneumoniae*.

**Results:** Although the bacterial isolates of each species did have the same types (ST11,KL27,O2v2 for *K.pneumoniae*, ST2613 for *P.aeruginosa* and ST8 for *S.aureus*, respectively) and similar core-genome composition, the numbers of single nucleotide polymorphisms (SNP) between the pairs of isolates have significantly exceeded the thresholds previously proposed for strain discrimination in literature (23-31 vs. 18 for *K.pneumoniae*, 70-85 vs. 37 for *P.aeruginosa,* 30-53 vs. 15 for *S.aureus*, respectively). At the same time, the number of SNPs between the isolates of the same MLST-type from different patients were always above 100 and sometimes exceeded 1000.

**Conclusion:** Our results suggest that although the number of core-genome SNPs proves to be useful measure for distinguishing distant bacterial strains, more research is needed to establish appropriate thresholds for it. We believe that the results obtained will be useful for developing new standards of NGS-based epidemiological surveillance of healthcare-associated infections since they allow to define new strain distinguishing thresholds based on genomic sequences.

**Disclosure of Interest**: None declared

### O16 VALIDATION OF A SEMIAUTOMATED SURVEILLANCE ALGORITHM FOR DEEP SURGICAL SITE INFECTIONS AFTER PRIMARY TOTAL HIP OR KNEE ARTHROPLASTY – INTERIM ANALYSIS OF A MULTICENTRE STUDY

#### J. Verberk^1,2,3^, S. van Rooden^3^, M. Koek^2^, T. Hopmans^2^, M. Bonten^1,3^, S. de Greeff^2^, M. van Mourik^1^

##### ^1^Medical Microbiology, UMC Utrecht, Utrecht; ^2^Epidemiology and Surveillance, RIVM, Bilthoven; ^3^Julius Center, UMC Utrecht, Utrecht, Netherlands

###### **Correspondence:** J. Verberk

**Introduction:** Surgical site infections (SSIs) complicate approximately 2% of primary total hip (THA) or total knee arthroplasty (TKA). Accurate and timely identification through surveillance is essential for targeted implementation and monitoring of preventive interventions. The availability of electronic health records facilitates (semi)automated surveillance, enabling high-quality large scale surveillance.

**Objectives:** This study assesses the validity of a previously published semiautomated surveillance algorithm for deep surgical site infections after THA or TKA, relying on retrospective routine care data.

**Methods:** Multicentre retrospective cohort study in four independent hospitals in the Netherlands. From all adult patients who underwent a THA or TKA, the following data were extracted from the electronic health records: microbiology results, antibiotics, (re)admissions, and surgical procedures within the 120 days following the primary surgery. Patients were classified with a low and high probability of having developed a deep SSI after THA or TKA, according to a previously developed algorithm. Sensitivity, positive predictive value (PPV) and workload reduction as compared to the traditional (manual) surveillance reported to the national surveillance database PREZIES were calculated.

**Results:** Four hospitals each extracted data from at least 1000 THA and TKA surgeries performed between 2012-2018. Preliminary analysis for one centre, based on 2395 records, demonstrates 77.4% sensitivity, 75% PPV and a workload reduction of 98.7%. Discrepancy analysis and analysis for the other centres are currently ongoing.

**Conclusion:** The results of this validation study are a prerequisite for successful broader implementation of semiautomated surveillance for SSIs after THA or TKA in the near future.

**Disclosure of Interest**: None declared

### O17 CLUSTER ANALYSIS WITH ANTIMICROBIAL RESISTANCE (AMR) DATA: DATA FROM SURVEILLANCE AND MONITORING SYSTEMS IN GERMANY

#### B. Suwono^1,2^, T. Eckmanns^2^, H. Kaspar^3^, B.-A. Tenhagen^1^

##### ^1^Epidemiology, Zoonoses and Antimicrobial Resistance, German Federal Institute for Risk Assessment; ^2^Infectious Diseases Epidemiology, Robert Koch Institute; ^3^Antimicrobial Resistance Monitoring, Federal Office of Consumer Protection and Food Safety, Berlin, Germany

###### **Correspondence:** B. Suwono

**Introduction:** German One Health Initiative (GOHI) has been initiated as a part of German National Action Plan on AMR.

**Objectives:** Under this framework, this study aims to cluster data on AMR from German national surveillance and monitoring systems from both human and animal sectors.

**Methods:** *Escherichia coli* data are collected from Antibiotic Resistance Surveillance (ARS) for humans, Zoonosis-Monitoring for non-clinical food-producing animal isolates and German Resistance Monitoring for Veterinary Medicine (GERM-Vet) for clinical animal isolates from 2014 to 2017. Human data originated from outpatients, general wards and intensive care units. Food-producing animal data were stratified by animal type and clinical vs. non-clinical origin. Three human origins and thirty-eight animal origins (e.g. broilers from slaughterhouse-non clinical, pigs-clinical, etc.) were analyzed. Ampicillin, cefotaxime, ciprofloxacin and gentamicin were the four antibiotics that were included, since they were frequently tested throughout these three systems. Cluster analysis was run based on sixteen resistance combinations; e.g. all susceptible (0000); using hierarchical clustering (Euclidian; average) in R.

**Results:** Three different clusters were detected based on the resistance combinations in isolates from each origin. Clinical human isolates clustered together (outpatient, general ward and intensive care unit). The cluster was closely related with isolates from pigs (weaners, clinical piglets, clinical and non-clinical sows, pork, growers < 50kg and fattening pigs). All isolates that having low resistance rates (<20%) against these four antibiotics clustered together (mostly non-clinical food-producing animals). All poultry isolates (turkeys and broilers) except broilers from organic farms grouped together with clinical isolates from pigs and bovines <1 year.

**Conclusion:** This study is the first study on cluster analysis using the data from national surveillance and monitoring systems for AMR for humans and food-producing animals in Germany. Detecting the closest relationship based on the resistance combination might be helpful for infection and prevention control. However, further analyses to better understand the clusters are necessary.

**Disclosure of Interest**: None declared

### O18 IMPACT OF NATURAL LANGUAGE PROCESSING OF CLINICAL NOTES ON DETECTION OF HEALTHCARE-ASSOCIATED INFECTIONS

#### J. Kozák^1^, L. Vraná^1^, P. Vavřinová^2^

##### ^1^Datlowe, s.r.o., Prague; ^2^Hospital Jihlava, Jihlava, Czech Republic

###### **Correspondence:** J. Kozák

**Introduction:** Manual active surveillance of healthcare-associated infections (HAIs) based on monitoring of all inpatient admissions and outpatient visits is very demanding and time consuming. Various automated methods have been introduced, e.g., monitoring positive microbiological cultivations. Not all HAIs are confirmed microbiologically however, as some relevant information for HAI detection might be hidden in unstructured clinical notes written by physicians and nurses. Sips et al. (2017) presents an overview of automated surveillance methods based on natural language processing (NLP) which allows analyzing unstructured texts. The authors state that NLP shows promising results, but needs further exploration.

**Objectives:** We measure the impact of NLP on the surveillance of HAIs.

**Methods:** Cooperating with Hospital Jihlava, treating about 25,000 inpatients annually, we have developed a technology based on NLP, which automatically detects potential HAIs by reading all available electronic health records. Potential HAIs are validated by members of the infection prevention team in Hospital Jihlava and this feedback is used to further improve the performance of NLP analysis.

**Results:** The technology has been deployed for more than a year. We have collected data on 920 confirmed HAIs during the past 12 months (1.5.2018 – 30.4.2019). 35.7% (328 cases) of those HAIs were initially not detected microbiologically, thus showing serious limitations when using traditional monitoring of positive lab results.

**Conclusion:** Methods based on NLP have proved the effectivity of detecting HAIs hidden in the unstructured texts, as well as those microbiologically confirmed. NLP does require reliable electronic health records that are not always readily available, but the trend of digitization is undeniable and before long most of the health data will be electronic. Therefore, NLP brings an enormous potential for automating the surveillance of HAIs.

**References**

Sips, M. E., Bonten, M. J. M., van Mourik, M. S.M.: Automated surveillance of healthcare-associated infections: state of the art, Current Opinion in Infectious Diseases: 2017, Vol. 30 (4), pp. 425–431, doi: 10.1097/QCO.0000000000000376

**Disclosure of Interest**: J. Kozák Employee of: Datlowe, s.r.o., the company which develops the solution HAIDi for automated detection of HAIs using advanced data analytics, i.e. natural language processing., Shareholder of: Datlowe, s.r.o., L. Vraná Employee of: Datlowe, s.r.o., the company which develops the solution HAIDi for automated detection of HAIs using advanced data analytics, i.e. natural language processing., P. Vavřinová: None declared

### O19 ENVIRONMENTAL SHEDDING OF TOXIGENC CLOSTRIDIOIDES DIFFICILE BY ASYMPTOMATIC PATIENTS

#### M. Gilboa, E. Houri Levi, C. Cohen, I. Tal, O. Feld Simon, A. Brom, Y. Eden Friedman, S. Segal, C. Rubin, G. Rahav, G. Regev Yochay, on behalf of the ShIC research group

##### Sheba Medical Centre, Ramat-Gan, Israel

###### **Correspondence:** M. Gilboa

**Introduction:** *Clostridioides difficile* (*CD*) is a leading cause of health care associated infections. The role of asymptomatic carriers in the shedding and transmission of *C. difficile* is unclear. This study is a nested study in a larger CD carriage surveillance study.

**Objectives:** Here, we aimed to determine the burden of environmental shedding of *C. difficile* among asymptomatic carriers in an inpatient non-epidemic setting.

**Methods:** CD carriage was determined upon admission in asymptomatic patients by PCR for CD toxin of a rectal swab. Environmental contamination of rooms inhabited by either CD carriers, CDI, or non-CD carrier patients was assessed by obtaining environmental specimens from 10 high-touch sites in the patient's room and bathroom. Specimens were cultured and toxigenic strains were identified by PCR. We created a contamination scale designating each room a level of environmental contamination combining the total number of colony forming units and the number of contaminated sites in the room.

**Results:** 117 rooms were screened; 70 rooms inhabited by carriers, 30 by active CDI patients and 17 by non CD-carriers (control). In the control group, 94% of the rooms were clean, with no colonies discovered, and one participant (6%) had a medium scale contamination. In the carrier group 27 rooms (39%) had more than residual contamination, from which 14 rooms (20%) had heavy contamination. In the CDI group 10 (33%) rooms had more than residual contamination from which 3 (10%) had heavy contamination. In a multivariate analysis adjusted for age, gender, Independency in ADL activities, and antibiotic use the contamination score of carriers' rooms was significantly higher than those of none carriers (p=0.028). The contamination score of active CDI patients was higher than that of the non-carriers bud did not reach statistical significance (p=0.083), yet, most (75%) of the CDI patients were treated with anti-CDI antibiotics on the day of screening. Patient's bathrooms were not more contaminated than the rooms themselves.

**Conclusion:** Here we showed that the environment of CD carriers is significantly higher than patients who were non-carriers and at least as contaminated as that of patients with an active CD infection (after initiation of treatment).

**Disclosure of Interest**: None declared

## Slide session: Antimicrobial stewardship

### O20 APPROPRIATENESS OF PROTECTED ANTI GRAM-NEGATIVE ANTIBIOTICS IN EIGHT SWISS HOSPITALS: PRELIMINARY RESULTS OF THE NRP72 PROJECT “OPA STUDY”

#### E. Moulin^1^, C. Plüss-Suard^1^, C. Bellini^2^, L. Christin^3^, C. Chuard^4^, O. Clerc^5^, A. Cometta^6^, V. Erard^4^, O. Marchetti^7^, N. Troillet^2^, C. Voide^2^, G. Zanetti^1^, L. Senn^1^

##### ^1^Centre Hospitalier Universitaire Vaudois, Lausanne; ^2^Institut Central des Hôpitaux, Sion; ^3^Groupement Hospitalier de l'Ouest lémanique, Nyon; ^4^Hôpital de Fribourg, Fribourg; ^5^Hôpital de Pourtalès, Neuchâtel; ^6^Etablissement hospitalier du nord vaudois, Yverdon-les-Bains; ^7^Etablissement Hospitalier de la Côte, Morges, Switzerland

###### **Correspondence:** E. Moulin

**Introduction:** Recent data on appropriateness of antibiotic therapies in Swiss hospitals are lacking and the extent of room for improvement is unknown.

**Objectives:** In the context of the NRP72 on antimicrobial resistance, we initiated the OPA project in eight Swiss hospitals located in the French-speaking part of Switzerland consisting in the evaluation of the impact of weekly clinical audits and multifaceted feedback strategies on reducing the use of anti-Gram-negative antibiotics that deserve a restrictive prescription: quinolones, 3rd- and 4th-generations cephalosporins, piperacillin/tazobactam and carbapenems. We report here the preliminary results on appropriateness.

**Methods:** Internal medicine, general surgery and intensive care units of participating hospitals were allocated to either intervention or control group. The intervention consisted in one-day weekly audits of protected antibiotic prescriptions over six months by a tandem of an infectious diseases specialist and a senior physician in charge of the patients, using a standardized checklist, followed by immediate feedback to prescribers and monthly reports to the medical team. Additionally, a website with didactic material dedicated to prescribers was created.

**Results:** Among a total of 9565 in-patients charts reviewed, we identified 1681 (18%) patients receiving a protected antibiotic targeted by the study. The auditing tandem proposed an optimization of the antibiotic therapy in 398/1681 (24%) patients including 167 (42%) stops, 87 (22%) switch to the oral route and 86 (22%) de-escalations.The adhesion rate to the propositions made by the tandem was 54%.

**Conclusion:** Preliminary results showed that there is room for improvement in prescriptions of protected antibiotics in the Swiss hospital setting. Special attention should focus on shorter durations, early switch to the oral route and de-escalations. Medical directors, head physicians and pharmacists of participating hospitals are aware of antibiotic resistance threat and support local initiatives aiming at antibiotic use optimization.

**Disclosure of Interest**: None declared

### O21 EFFICACY OF GENTAMICIN IN THE PREVENTION OF POST-TRANSPLANT INFECTIONS IN LIVER TRANSPLANT RECIPIENTS; A RANDOMIZED CONTROLLED TRIAL

#### G. Pouladfar^1^, M. Shafikhani^2^, A. Vazin^3^, S. Nikeghbalian^4^, A. Abbasian^1^

##### ^1^Professor Alborzi Clinical Microbiology Research Center; ^2^Student Research Committee; ^3^Pharmaceutical Sciences Research Center; ^4^Transplant Research Center, Abu Ali Sina for Medicine & Organ Transplant Hospital, Shiraz University of Medical Sciences, Shiraz, Iran, Islamic Republic Of

###### **Correspondence:** G. Pouladfar

**Introduction:** Post-transplant infections (PTIs) are one of the most important causes of mortality and morbidity after liver transplantation (LT).

**Objectives:** In this study, we aimed to compare the efficacy of combination of ceftizoxime with ampicillin-sulbactam versus combination of gentamicin with ampicillin-sulbactam as prophylactic antibiotic regimens used to prevent early bacterial PTIs in LT recipients.

**Methods:** All consecutive patients aged 18 years old and over who underwent LT at the Abu-Ali Sina transplantation hospital in Shiraz, Iran from July 2018 to April 2019, were included prospectively in this study. Randomization was performed in permuted blocks. We randomly assigned participants to receive two prophylactic antibiotic regimens: either combination of intravenous ceftizoxime and ampicillin-sulbactam (ceftizoxime group) or gentamicin and ampicillin-sulbactam (gentamicin group). These regimens started one hour before surgery and continued for 48 hours after LT. The rate and type of bacterial infections, the length of hospital and intensive care unit stays, mortality rate and renal function status were assessed during one-month period after LT.

**Results:** Totally, 230 participants were equally allocated to two groups. One participant in the gentamicin group and five in the ceftizoxime group were excluded due to expiring earlier than 3 days after LT. The rate of bacterial PTI during the first month after transplantation was 25.4% which was significantly lower in gentamicin group (13.16%) than in ceftizoxime group (38.18%) (p-value <0.01). The lengths of ICU and hospital stays and mortality rate were significantly lower in gentamicin group (p-value <0.01). There was no statistically significant difference in renal function status between the two groups. (p-value= 0.16).

**Conclusion:** The results showed that gentamicin can serve as a promising agent in a prophylactic antibiotic regimen in patients undergoing LT.

**Disclosure of Interest**: None declared

### O22 DE-IMPLEMENTING LOW VALUE ANTIBIOTIC PRESCRIBING ACROSS LEVELS OF CARE

#### M. Schweizer^1^, C. Hartmann^2^, K. Gupta^2^

##### ^1^Iowa City VA Hospital, Iowa City; ^2^Boston VA Healthcare System, Boston, United States

###### **Correspondence:** M. Schweizer

**Introduction:** Performing urinalyses and urine cultures in asymptomatic patients is one of the most common reasons for inappropriate antibiotic use. However, de-implementing this practice has been difficult, especially for clinical scenarios deemed to be high risk for infectious complications, such as among patients with delirium or those undergoing orthopedic implant surgery.

**Objectives:** Using the dual process theory framework “Developing De-Implementation Strategies Based on Un-Learning and Substitution,” an educational intervention citing new Infectious Diseases Society of America guidelines and providing a pneumonic “ABCs of ASB” was created and delivered didactically to providers. The goal was to increase performance of evidence-based prevention actions in place of low-value urine screening and treating of asymptomatic patients.

**Methods:** Clinical providers and staff (physicians, nurses, nurse practitioners, trainees) in 3 different levels of care (acute inpatient, long term, and outpatient) were included. A web-based anonymous and confidential pre and post question format was delivered to assess influence on provider behavior.

**Results:** Responses from a range of 250-279 unique providers were collected. For scenario #1 (patient with delirium and a positive urine culture and no other infectious symptoms), the choice to give antibiotics was reduced from 45% pre to 4% post, Chi-square p < 0.01). For scenario #2 (patient having knee replacement and positive preoperative urine culture, no other symptoms) the choice to give antibiotics was reduced by the same magnitude (~50%) but a lower absolute number (67% pre and 33% post, chi-square p < 0.01). Changes in predicted behavior were similar across levels of care.

**Conclusion:** Substituting evidence-based practices in place of low value practices is an appealing framework for influencing provider behavior. Our work demonstrates that education can successfully reduce the intention to use antibiotics for asymptomatic patients with positive urine cultures.

**Disclosure of Interest**: M. Schweizer Grant/Research support from: PDI Healthcare, C. Hartmann: None declared, K. Gupta: None declared

### O23 HEALTHCARE-ASSOCIATED INFECTIONS: RESULTS OF THE 2017 AND 2018 GLOBAL POINT PREVALENCE SURVEY OF ANTIMICROBIAL CONSUMPTION AND RESISTANCE (GLOBAL-PPS)

#### A. Versporten^1^, I. Pauwels^1^, S. Le Page^2^, H. Goossens^1^ on behalf of the Global-PPS network

##### ^1^Laboratory of Medical Microbiology, University of Antwerp, Antwerp, Belgium; ^2^bioMérieux, Marcy l ’Etoile, France

###### **Correspondence:** A. Versporten

**Introduction:** Point Prevalence Surveys of antimicrobial use (AMU) and healthcare-associated infections (HAI) are well established surveillance methods for monitoring AMU and HAI in hospitals. bioMérieux provided unrestricted funding support for the survey.

**Objectives:** We aimed to asses worldwide variation of quantity and quality of AMU for HAI.

**Methods:** Validated Global-PPS data was used from 628 hospitals (H) in 57 countries (C), including Europe (20C;179H); Africa (8C;116H), Asia (16C;169H), South-America (10C;90H), North-America (2C;65H), and Oceania (1C;9H) from 2017 to 2018. Detailed data was collected for all inpatients receiving an antimicrobial on the day of the survey. Denominator included all admitted inpatients. A web-based application was used for data-entry, validation and reporting (www.global-pps.com).

**Results:** Out of 152,966 admitted patients, 40.8% recieved at least one antimicrobial (range: 30.1% in Europe to 63.4% in Africa). HAI prevalence was 9.0% (range: 7.2% in Europe to 13.7% in South-America). Top 3 HAI included pneumonia (28.6%), skin and soft tissue infections (11.3%) and intra-abdominal sepsis (9.4%). Out of all antimicrobials (n=141,169); antibiotics for systemic use represented 89.0% (n=125,705) of which 22.3% (n=28,018) were prescribed to treat a HAI (range: 12.9% in Africa to 32.4% in South-America). Top 3 antibiotics for HAI were penicillin/β-lactamase-inhibitor (21.8%); carbapenems (14.6%; highest in South-America: 22.8%) and quinolones (10.8%; highest in North-America: 13.5%). Among 13,751 patients with at least one HAI, 53.0% got a targeted antibiotic treatment among which an ESBL-producing Enterobacteriaceae was most often reported (12.2%; range: 4.6% in North-America to 22.1% in South-America). The reason to treat a HAI was recorded in 85.1% of antibiotic prescriptions; a stop/review date in 42.2% and local guidelines were missing in 15.9% of antibiotic prescriptions.

**Conclusion:** The Global-PPS provides quantifiable outcomes to assess and compare quantity and quality of antibiotic prescribing for HAI in hospitalized patients worldwide. Hospitals use these data for quality improvement of antibiotic prescribing, development of local prescribing guidelines, practice changes, and for measuring the impact of interventions through repeated PPS.

**Disclosure of Interest**: None declared

### O24 THE IMPACT OF AN INFECTIOUS DISEASES PHYSICIAN-LED ANTIMICROBIAL STEWARDSHIP PROGRAM ON “HIGH-END” ANTIBIOTIC CONSUMPTION, RESISTANCE, EXPENDITURE AND PATIENT OUTCOME

#### R. A. Moghnieh, L. Awad, D. Abdallah, M. Jadayel, S. Al-Hassan, R. Dabbagh, S. Droubi, N. Droubi

##### Makassed General Hospital, ^2^Beirut Arab University, Beirut, Lebanon

###### **Correspondence:** R. A. Moghnieh

**Introduction:** The rational use of antimicrobials is the key to curb down antimicrobial resistance. Makassed General Hospital adopted an Infectious Diseases specialist (IDS)-led antimicrobial stewardship program (ASP) in September,2016. Previously, broad-spectrum antibiotic (BSAB) dispensing was restricted through an institutional policy set by the hospital Pharmacy and Therapeutics Committee.

**Objectives:** We studied the effect of this IDS-led ASP on BSAB consumption levels, expenditure, resistance in pathogens causing nosocomial bacteremia. We also assessed the ASP effect on in-hospital and Intensive Care Unit (ICU) mortality.

**Methods:** Our study comprised of two periods: pre-ASP (Oct.2011-Sep.2015) and post-ASP (Oct.2016-Sep.2018). Both periods were divided to quarters (Q). Each Q comprised of 3 months. The period from Oct.2015 to Sep.2016 was a wash out period and was excluded from the analysis. We calculated the average/Q and the average Q-to-Q variation (AQV) before and after ASP for: BSAB consumption expressed in the number of defined daily dose (DDD)/1000 patient (pt) days (PD), BSAB expenditure expressed in US dollars/PD, the percentage of resistant bacteria from the total number of nosocomial pathogens causing bacteremia, in-hospital mortality (number of deaths/1000PD), and ICU-mortality (number of ICU deaths/1000 ICU days).

**Results:**

**Table:** 

	Average/Q before ASP	Average/Q after ASP	AQV before ASP(%)	AQV after ASP(%)
BSAB consumption				
carbapenems(C)	154 DDD/1000PD	157 DDD/1000PD	4.7	-2.8
tigecycline	31 DDD/1000PD	12 DDD/1000PD	12.1	3.0
colistin	68 DDD/1000PD	58 DDD/1000PD	28.9	2.8
Expenditure	46 USD/PD	37 USD/PD	3.6	-0.7
Bacteria causing bacteremia				
C resistant (R) Acinetobacter baumannii	20%	13%	46.7	14.9
CR Pseudomonas aeruginosa	7%	4%	-57.7	-87.2
Mortality				
total	8 pt/1000PD	7 pt/1000PD	7.8	3.5
ICU	53 pt/1000 ICU days	47 pt/1000 ICU days	11.6	9.3

**Conclusion:** Our IDS-led ASP succeeded in controlling BSAB prescription rates and in decreasing the incidence of resistant pathogens causimg bacteremia without compromising pt outcome, not to mention its economic effect in reducing AB expenditure.

**Disclosure of Interest**: None declared

### O25 UPDATE ON SURVEILLANCES TO MITIGATE ANTIBIOTIC RESISTANT CARE INFECTIONS IN BELGIUM

#### B. Catry^1,2^, K. Latour^1^, E. Vandael^1^

##### ^1^Healthcare associated infections & Antimicrobial resistance (NSIH), Sciensano; ^2^Faculty of Medecine, Université libre de Bruxelles, Brussels, Belgium

###### **Correspondence:** B. Catry

**Introduction:** Based upon point prevalence surveys, the latest estimations of healthcare-associated infections in Belgium are 7.3% in acute care facilities (2017) and 3.5% in long-term care facilities (2016).

**Objectives:** To update the antimicrobial consumption and resistance situation in Belgian acute care hospitals.

**Methods:** By Royal Decree, Belgian acute care hospitals, mandatorily have to participate in the surveillance of methicillin resistant *Staphylococcus aureus* (MRSA) and multiresistant Gram-negative bacteria (MRGN). Participation in the surveillance of resistant enterococci is optional. The surveillance of antibiotic use is based on reimbursement data and expresses consumption in defined daily doses (DDDs) per 1000 patient days and per 1000 admissions (World Health Organization version 2018).

**Results:** The median antibiotic consumption in acute care Belgian hospitals in 2017 (592.6 DDDs/1000 patient days) remained similar to the previous years. In a 15-year period (2003-2017), there was a small increase in the median consumption in DDDs/1000 patients and a small decrease (2008-2016) in DDDs/1000 admissions. The high variation in antibiotic consumption between acute care hospitals and the high use of broad-spectrum antibiotics (especially fluoroquinolones) should be targets for improvement.

In line with surrounding countries, a further decrease of the incidence of nosocomial MRSA was noticed: from 0.36 to 0.08/1000 admissions (2004-2017). The incidence of MRGN (ESBL; extended spectrum beta-lactamase, and to a lesser extent CPE; carbapenemase producing enterobacteriaceae) however is jeopardizing this favorable trend. In 2017, the median of incidence of ESBL+ *Escherichia coli* (3.79/1000 admissions) and *Klebsiella pneumoniae* (1.87/1000 admissions) surpassed the incidence of nosocomial MRSA. Also other enteric multidrug resistant organisms (MDRO) like vancomycin resistant *Enterococcus faecium* (0.068/1000 admissions in 2017) are on the rise.

**Conclusion:** In summary, while infection control has reduced the incidence of MRSA, the containment of other MDRO, in particular the reservoir in the gastrointestinal tract, now needs priority. To assess the appropriateness of antibiotic use, a diagnosis driven data collection is needed.

**Disclosure of Interest**: None declared

### O26 PILOTING THE GAMIFIED ANTIMICROBIAL STEWARDSHIP DECISION SUPPORT APP (GADSA): INCREASING COMPLIANCE WITH GUIDANCE FOR PRESCRIPTION OF SURGICAL ANTIBIOTIC PROPHYLAXIS IN NIGERIA

#### C. E. Wood^1^, O. Olufemi^2^, F. Ogunsola^2^, P. Okonji^2^, E. Kpokiri^3^, S. Luedtke^4^, L. Shallcross^5^, D. Soriano^1^, C. Lefevre-Lewis^5^, G. Birjovanu^1^, A. Hayward^5^, P. Kostkova^1^, F. NCube^6^, A. Molnar^7^, S. Wiseman^1^

##### ^1^IRDR Centre for Digital Public Health in Emergencies, University College London, London, United Kingdom; ^2^Lagos University College of Medicine, Lagos; ^3^Niger Delta University Teaching Hospital, Bayelsa State, Nigeria; ^4^London School of Hygiene and Tropical Medicine; ^5^University College London; ^6^Public Health England, London, United Kingdom; ^7^Swinburne University of Technology, Melbourne, Australia

###### **Correspondence:** C. E. Wood

**Introduction:** 20-50% of surgical antibiotic prophylaxis prescription in Nigeria is thought to be non-compliant with WHO guidance. Game-based decision support mobile apps improve engagement with text-based guidance and develop practical skills. Development of the app involved building decision tree algorithms based on guidance published by WHO and Sanford with co-design input from surgeons at three hospital sites: Lagos University Teaching Hospital (LUTH), Lagos State University Teaching Hospital (LASUTH), Niger Delta University Teaching Hospital (NDUTH).

**Objectives:** Evaluate preliminary impact of a game-based smartphone app on prescribing behaviour and compliance with guidelines for surgical antibiotic prophylaxis

**Methods:** Surgeons were recruited by local project leads from the hospital sites. The Android-based app was used for an 8-week period (April-June 2019) to record prescribing decisions for elective surgeries. Pre- and post-pilot, surgeons completed a 20-item questionnaire on prescribing habits, intention to comply and attitudes towards, compliance with guidance. In-app feedback was provided by an interactive ‘mentor’. Badges were awarded for compliant decisions and interaction with the app. Data on decisions and interactions were collected. Feedback on acceptability and usability was collected via focus groups/questionnaires at week 4 and 8.

**Results:** 80 surgeons (consultant; 5-10yrs experience; 60% male) joined the pilot. Preliminary data (May 2019) shows positive impact on prescribing behaviours and attitudes towards compliance, with reduction in inappropriate prolongation of antibiotics post-surgery. The pilot completes in June, full results available August and presented at ICPIC 2019.

**Conclusion:** This pilot reflects willingness of surgeons to use an innovative solution with potential to reduce inappropriate use of antibiotics and improve compliance with prescribing guidance.

**Disclosure of Interest**: None declared

## Slide session: Hand hygiene

### O27 PREVENTION OF CROSS-TRANSMISSION BEFORE ASEPTIC PROCEDURE (WHO MOMENT 2) USING A SIMPLIFIED METHOD FOR HAND HYGIENE

#### H. Soule, M. Abbas, J. Sauser, C. Fankhauser, D. Pires, D. Pittet

##### Infection Control Programme and WHO Collaborating Centre on Patient Safety, Geneva University Hospitals, Geneva 4, Switzerland

###### **Correspondence:** H. Soule

**Introduction:** In previous studies, we have shown that a simplified method for hand hygiene (HH; 15 sec rubbing with a hand size-adjusted volume of alcohol and fingertips first followed by the rest of the hands) can lead to a reduction of approximately 2 log_10_ on hands artificially contaminated with either *Escherichia coli* or *Staphylococcus aureus*.

**Objectives:** We evaluated the efficacy of this simplified HH method in a laboratory experiment simulating WHO moment 2 (before aseptic procedure).

**Methods:** Twenty nurses were enrolled in the study. After handwashing with soft soap, 10 μl of a 10^8^ cfu/mL *S. aureus* NC10788 suspension was deposited on each of their fingertips and let dried for 3 minutes. They then simulated injection in an infusion set, both with and without previous HH with isopropanol 60% (v/v) according to our simplified method (see above). They manipulated the 3-way stopcock integrated in the infusion set and the flow-regulating clamp. The whole procedure lasted approximately 3 minutes. The 3-way stopcock and the flow-regulating clamp were then cut, placed in 100 ml tryptone soy mixture, shaken vigorously, and both dilutions and filtration of the recovering solution were inoculated on agar for 48 hours. We had previously established that our method was able to detect a contamination of those 2 parts of the infusion set inferior to 30 cfu. The number of colonies was counted and Wilcoxon signed-rank test was used to test the difference between the results with and without HH. The Hodges-Lehmann estimator was applied to estimate the difference between the medians.

**Results:** The median amount of baseline *S. aureus* on the fingertips of the volunteers was 10^6.8^ cfu. Without HH, bacteria were detected after all 20 experiments (range: 25 to 25 800 cfu), whereas with HH, bacteria were not detected after all 20 experiments. The difference between estimated medians was 351 cfu (95% CI 193-570; p = 0.0001).

**Conclusion:** Even with a high contamination of the fingertips with *S. aureus*, when HH was performed with this simplified method before an aseptic procedure, no bacteria were recovered from 2 critical parts of the infusion set, allowing safer care and patient safety.

**Disclosure of Interest**: None declared

### O28 IMPACT OF THE SEVENTH MULTIMODAL COUNTRY-WIDE CAMPAIGN TO PROMOTE HAND HYGIENE IN BELGIAN HOSPITALS

#### H. De Pauw^1^, A. Uwineza^1^, N. Benhammadi^1^, B. Catry^1,2^, A. Simon^3^ on behalf of Working Group Hand Hygiene BAPCOC (Belgian Antibiotic Policy Coordination Committee)

##### ^1^Healthcare associated infections & Antimicrobial resistance (NSIH), Sciensano; ^2^Faculty of Medecine, Université libre de Bruxelles; ^3^Université catholique de Louvain, Brussels, Belgium

###### **Correspondence:** H. De Pauw

**Introduction:** Hand hygiene (HH) compliance by healthcare professionals has been recognized as the most important factor in preventing transmission of healthcare-associated infections to patients (1).

**Objectives:** We report here the outcome of the seventh Belgian national hand hygiene campaign organized in 2016.

**Methods:** The campaign was mainly focused on healthcare workers having contact with patients in hospitals (acute, chronic and psychiatric), and also for the first time on the patients themselves (patient questionnaires). Compliance to hand hygiene guidelines was measured using a standardized observation roster (2). An online tool (NSIHweb 2.0) was used to collect the individual or aggregated compliance data, with the possibility to obtain immediate feedback. The patient questionnaire was filled out in paper format and manually introduced in a databank.

**Results:** A total of a total of 235,816 hand hygiene opportunities were registered from 170 participating hospitals. At the national level, all specialties combined, the compliance (= hand hygiene opportunities with soap and/or alcohol / total number of hand hygiene opportunities observed) was 71.6% before the campaign and 78.0% after the campaign.

Ninety-seven Belgian sites / hospitals voluntarily forwarded patient inquiries and 17,454 received questionnaires were included in the analysis. The survey showed that 59.0% of the participants reported being aware that the hospital was participating in the hand hygiene campaign.

**Conclusion:** The seventh national campaign was another success in terms of very high participation rates, and a compliance rate tending to approach a 80% margin during post-campaign. Patient empowerment was for the first time positively stimulated.

**References**

(1) European Centre for Disease Prevention and Control. (2013). Point prevalence survey of healthcare-associated infections and antimicrobial use in European acute care hospitals. Stockholm: ECDC. doi 10.2900/86011

(2) World Health Organization Patient Safety: WHO Guidelines on Hand Hygiene in Health Care: First Global Patient Safety Challenge, Clean Care is Safer Care. Geneva Switzerland: World Health Organization; 2009.

**Disclosure of Interest**: None declared

### O29 IMPACT OF THE MULTIMODAL STRATEGY FOR INCREASING HAND HYGIENE COMPLIANCE: FOURTEEN YEARS’ EXPERIENCE IN A MIDDLE INCOME COUNTRY

#### E. Alp^1^, S. Tasgin^2^, D. Altun^2^, T. Orhan^2^, F. Cevahir^2^, O. Cakir^2^, E. Aktas^2^, C. Altay Kurkcu^2^, A. UluKilic^2^

##### ^1^Ministry of Health, Ankara; ^2^Erciyes University, Kayseri, Turkey

###### **Correspondence:** E. Alp

**Introduction:** Hand hygiene is a gold standard for the prevention of health-care associated infections (HAIs). However, compliance rates are generally poor among healthcare workers (HCWs) in routine practice.

**Objectives:** We aimed to assess the efficacy of WHO multimodal strategy for improving hand hygiene (HH) compliance in a middle-income country.

**Methods:** The WHO multimodal HH improvement strategy has been implemented since 2004 in a referral university hospital in Turkey. The intervention consisted of introducing alcohol-based hand rub at bedside and nurses’ treatment and dressing rooms; monitoring HH compliance; providing performance feedback; educating staff; posting reminders in the workplace; and promoting an institutional safety climate. A bundle strategy has been implemented for the prevention of device-associated infections in intensive care units (ICUs). HH compliance in medical, anaesthesiology and ICUs, hand rub consumption and HAIs rates were evaluated at baseline and at follow-up.

**Results:** The compliance of HH increased in medical, anaesthesiology and ICUs in all five moments. Also, the usage of alcohol based hand rub has increased from 195 litres in 2003 to 11543 litres in 2018. Point prevalence studies revealed a decrease in HAIs from 8.2% to 5.7% between 2007 and 2018. Device-associated infection rates (ventilator-associated pneumonia, catheter associated blood stream infections, catheter associated urinary tract infections) in ICUs also decreased during the time. Furthermore, multi-drug resistant pathogens (*Acinetobacter baumannii, Pseudomonas aeruginosa, Klebsiella pneumonia, Escherichia coli*) incidence rates decreased. Small bowel operations, colon, gastric, cholecystectomy, craniotomy, fusion, cranial shunt infection rates decreased over the time.

**Conclusion:** The WHO multimodal improvement strategy has shown to be effective in improving HH compliance and decreasing HAIs rates in a middle income country with limited nurses and heavy workload.

**Disclosure of Interest**: None declared

### O30 IMPACT OF USING A DEVICE PROVIDING INDIVIDUAL FEEDBACK ON HEALTHCARE WORKERS HAND HYGIENE BEHAVIOUR: A STEPPED WEDGE CLUSTER-RANDOMIZED CLINICAL TRIAL (SMARTRUB®)

#### D. Pires^1^, A. Gayet-Ageron^1^, Y. Robert^2^, C. Fankhauser^1^, E. Tartari^1^, C. G^1^, A. Peters^1^, F. Tymurkaynak^1^, S. Fourquier^3^, H. Soule^1^, R. Beuchat^3^, W. Zingg^1^, F. Bellissimo-Rodrigues1^1^, Y. Martin^1^, D. Pittet^1^

##### ^1^IPC, University of Geneva Hospitals; ^2^iQati; ^3^HEPIA, Geneva, Switzerland

###### **Correspondence:** D. Pires

**Introduction:** We tested the effect of an innovative wristband (SmartRub^®^ powered by iQati^TM^) that provides automatic, instantaneous and individual feedback on the correct duration of hand friction and volume of alcohol-based handrub (ABHR).

**Objectives:** We hypothesised that using it in patient care would improve hand hygiene (HH) compliance by 20%.

**Methods:** We performed a stepped wedge, cluster-randomized, controlled, open-label clinical trial. All wards of our geriatric university hospital were randomized to 1 of 4 steps. Each step consisted in a unique sequence of 3 phases: baseline (no device;1-4 months), transition (device without feedback;1 month) and intervention (device with feedback;1-4 months). Primary outcome was HH compliance measured by direct HH observation. Secondary outcomes were the duration of HH friction and ABHR volume. Generalized linear mixed models with nested random effects on the intercept (HCW within ward-level) were performed on an intention-to-treat level.

**Results:** A total of 97 of the 370 HCWs participated (63 nurses, 32 auxiliary nurses, 2 physios). Overall, 6’878 HH opportunities (opp) were observed with a median of 72 opp per HCW (IQR 61-84). Mean HH compliances (95%CI) were 72% (67-76), 70% (65-75) and 62% (57-67) at baseline, transition and intervention, respectively. HH compliance decreased significantly over time (p=0.015) and there was no effect of the duration of active device use (p=0.448). HH compliance was independently and inversely associated with age (p=0.015) and workload (p<0.001). Both ABHR volume and duration of HH friction have increased significantly from transition to intervention (1.4 mL, 95%CI 1.1-1.6 to 2.1 mL 95%CI 1.8-2.3 and 9.3 sec 95%CI 8.5-10.0 to 11.1 sec 95%CI 10.3-11.8).

**Conclusion:** The use of SmartRub^®^ did not show an effect on HH compliance. We observed a gradual decrease in compliance throughout the study that could be attributed to fading of the initial Hawthorne effect. On the other hand, the use of SmartRub^®^ improved the quality of HH both in ABHR volume and duration of HH friction.

**Disclosure of Interest**: None declared

### O31 EXPLORING INSTITUTIONAL SAFETY CLIMATE TO PROMOTE HAND HYGIENE: RESULTS FROM AN INTERNATIONAL SURVEY

#### E. Tartari^1^, M. Borg^2^, E. Castro-Sánchez^3^, W. Zingg^1^, B. Allegranzi^4^, D. Pittet^1^

##### ^1^University Hospital of Geneva, Geneva, Switzerland; ^2^Mater Dei Hospital , Msida, Malta; ^3^Imperial College London, London, United Kingdom; ^4^World Health Organization, Geneva, Switzerland

###### **Correspondence:** E. Tartari

**Introduction:** Institutional safety climate (ie, the safety aspects of organizational culture) is an essential component of the World Health Organization's (WHO) multimodal hand hygiene (HH) improvement strategy for sustained health systems quality.

**Objectives:** We sought to explore the key elements representing the institutional safety culture.

**Methods:** We developed a survey based on the WHO Hand Hygiene Self-Assessment Framework (HHSAF). A convenience sample of infection control preventionists (ICPs) from more than 100 countries attending the International Conference on Prevention and Infection Control (ICPIC) in June 2017 was invited by email to complete the survey. The survey included questions regarding the following subcategories within the HHSAF: 1) commitment of leadership; 2) champions and role models; 3) patient participation; 4) system for accountability 5) HH compliance targets and 6) reporting.

**Results:** 198 ICPs from 71 countries across all WHO regions completed the questionnaire (14% Africa; 11% Americas; 5% South East Asia; 43% Europe; 9% Eastern Mediterranean and 18% Western Pacific). Only 9% of respondents reported having all six elements in place. 36% reported that facility leadership made a clear commitment to support HH improvement and undertook leadership hospital walkabouts. 38% had a designated system of HH champions (HHC), primarily nurses. Significantly less doctors and almost no housekeepers were designated HHC (p= 0.007). It was almost non-existent for patients to challenge nurses (3.7%) and doctors (2.1%) about poor hand hygiene practices. 44% of respondents claimed some form of HH accountability systems was present. However, programmes that reward good HH practices and disincentivize non-compliance were only reported by 26% and 12%, respectively. 42% reported established HH institutional targets, of which only 24% were required to publicly report them.

**Conclusion:** Our findings demonstrate that there is a general lack of inclusion of key elements constituting institutional safety climate within HH programmes worldwide. Unless political and leadership determinants are addressed, sustained and genuine HH improvement is unlikely.

**Disclosure of Interest**: None declared

### O32 PREVENTATIVE MEASURES TO IMPROVE HAND HEALTH OF HEALTHCARE WORKERS AND THE POTENTIAL IMPACT UPON HAND HYGIENE BEHAVIOUR

#### J. Hines^1^, S. Kezic^2^, T. Rustemeyer^3^, M. Soltanipoor^2,3^

##### ^1^Research & Development, SC Johnson Professional, Derby, United Kingdom; ^2^Coronel Institute of Occupational Health , Amsterdam Public Health Research Institute, Amsterdam UMC; ^3^Department of Dermatology, Amsterdam UMC, Amsterdam, Netherlands

###### **Correspondence:** J. Hines

**Introduction:** Healthcare workers (HCWs) are at high risk of developing hand dermatitis (HD). Current guidelines on HD prevention recommend use of emollients however adherence is poor. Compliance to hand hygiene (HH) guidelines depends on several factors including skin health.

**Objectives:** To assess whether provision of emollient cream, electronic monitoring and feedback on consumption can improve skin care in HCWs and to consider the relationship between improved skin health and HH compliance.

**Methods:** A cluster randomized controlled trial was conducted on 19 academic hospital wards, including 501 HCWs, for 12 months. Intervention wards were provided with hand cream dispensers equipped with an electronic system to monitor use, regularly communicated using posters. Process measures were self-reported and electronically measured cream use in the intervention group (IG) vs control group (CG). Primary and secondary outcomes were change from baseline in Hand Eczema Severity Index (ΔHECSI) and Natural Moisturizing Factor (ΔNMF). HH compliance was audited independently and trends later compared.

**Results:** Self-reported cream use at follow-up was significantly higher in IG than in CG before and during shift. At baseline there was no difference between groups. In IG, electronically measured cream use averaged 0.4 events per shift per HCW. HECSI reduced in IG by -6.2 and in CG by -4.2 points. There was no difference in ΔHECSI or ΔNMF between groups however relative improvement was significantly higher in IG (56% vs. 44%). In a subgroup of HCW with mild HD, IG showed significantly larger HECSI decrease than CG (P<0.001). HH compliance on IG wards increased from 55% (Q3 2015) to 70% (Q2 2017) during the project.

**Conclusion:** The intervention improved hand cream use, however consumption remained low. Although there was no significant effect on the primary outcomes, the intervention showed overall positive effects on HECSI and may be considered a practical means to promote skin care in HCWs. Reported HH compliance increased during the study. While causality cannot be assigned, this suggests a relationship and highlights the importance of HCW skin health in infection prevention strategies.

**Disclosure of Interest**: J. Hines Employee of: SC Johnson Professional, S. Kezic: None declared, T. Rustemeyer: None declared, M. Soltanipoor: None declared

### O33 THE EFFECT OF HAND RUB CONSUMPTION ON HEALTHCARE-ASSOCIATED STAPHYLOCOCCUS AUREUS BLOODSTREAM INFECTIONS IN FINNISH ACUTE CARE HOSPITALS

#### D. Arifulla, E. Sarvikivi, J. Ollgren, S. Toura, O. Lyytikäinen

##### NATIONAL INSTITUTE FOR HEALTH AND WELFARE, Helsinki, Finland

###### **Correspondence:** D. Arifulla

**Introduction:** Improved hand hygiene (HH) has been shown to reduce healthcare-associated (HA) *Staphylococcus aureus* (SA) bloodstream infections (BSI). In the Finnish hospital infection program (SIRO) HA-SA-BSI rates have been increasing.

**Objectives:** Our aim was to investigate whether HA-SA-BSI rates in Finnish acute care hospitals were associated with HH activities.

**Methods:** Information on hand rub consumption and HH observations in Finnish acute care hospitals were collected as a part of national web-based surveys in 2014, 2015, 2017 and 2018. During 2014-2018, 20 hospitals conducted laboratory-based surveillance of HA-BSIs with a common protocol. Patient-days were obtained from hospitals’ databases to calculate incidence densities (ID). Association between hand rub consumption and HH observations was estimated with linear mixed regression, and HH and SA-BSI for each hospital was assessed by Locally Weighted Scatterplot Smoothing (LOWESS).

**Results:** Overall SA-BSI ID was 2.1/10,000 patient-days (range by hospital, 0.7-4.0; range by year, 1.8-2.2). Mean hand rub consumption was 60 liters/1000 patient-days (range by hospital, 23-143), and by survey, 54-94% of the hospitals performed HH observation. Hand rub consumption was 1.5 (95% confidence interval, 1.1-2.0) times greater in hospitals performing HH observation. We detected a slight negative association between SA-BSI IDs and hand rub consumption in the LOWESS analysis.

**Conclusion:** Hand rub consumption seemed to have an effect on HA-SA-BSI. Hand rub consumption, a rough surrogate of HH compliance, is easily available. Observation would give more accurate picture on HH, but it is resource intensive and needs standardized methods and training if used as a national process indicator.

**Disclosure of Interest**: None declared

## Slide session: Multidrug resistant Gram-negatives

### O34 EFFECTIVENESS OF A MULTIMODAL STRATEGY TO REDUCE CARBAPENEM-RESISTANT KLEBSIELLA PNEUMONIAE INFECTIONS: THE TEN YEARS’ EXPERIENCE OF A 400 BEDS TERTIARY HOSPITAL IN NORTHERN ITALY

#### C. Alicino^1^, D. Raiteri^1^, S. Tigano^2^, L. Santoriello^1^, S. Brenci^1^, C. Valle^1^, P. Pavan^1^, F. Lillo^1^, G. Barabino^1^, G. Riccio^2^, C. Airoldi^3^, L. Garra^1^

##### ^1^ASL 2 Savonese, Pietra Ligure; ^2^ASL 2 Savonese, Albenga; ^3^ASL 2 Savonese, Savona, Italy

###### **Correspondence:** C. Alicino

**Introduction:** In recent years, carbapenem-resistant *Klebsiella pneumoniae* (CR-Kp) has become endemic in Italy and effective interventions are needed to contrast its burden.

**Objectives:** The study aimed to evaluate the effectiveness of multimodal strategy for reducing the incidence of CR-Kp pneumoniae infections at the 400 beds tertiary hospital “Santa Corona of Pietra Ligure”, Liguria Region, Northern Italy.

**Methods:** The intervention effect was analyzed with time series regression analysis. The study included a pre-intervention period (January 2009 – August 2009), a multimodal intervention period without routine rectal screening (September 2009 – December 2014) and with routine rectal screening (January 2015 – December 2018). Multimodal intervention consisted of contact precautions, patient isolation, enhanced environmental cleaning. By negative binomial regression, monthly incidence of Kp-RC isolations on blood culture, urine culture, respiratory samples and on other clinical samples were compared. The analysis was conducted separately for the Intensive Care Unit (ICU) and for the other wards.

**Results:** Preliminary results demonstrated that multimodal interventions without screening was effective in reducing monthly incidence of Kp-RC isolations on blood culture both in ICU (Incidence Rate Ratio – IRR: 0.90; p<0.001) and in the other wards (IRR: 0.85; p<0.001). The introduction of rectal screening was associated to a further decrease of Kp-RC isolations on blood culture in ICU (IRR: 0.92; p<0.001), while the impact in the remaining wards was modest (IRR: 0.99; p<0.001). A reduction in the incidence of isolation, even though more limited, was observed also in urine culture and other clinical samples. With respect of respiratory samples, the reduction was observed only after the implementation of rectal swab screening and was more pronounced in the ICU (IRR:0.93; p <0.001).

**Conclusion:** Early adoption of a multimodal strategy has been shown to be effective in reducing the incidence of Kp-RC infections. Routine screening with rectal swab allowed a further reduction to be achieved, particularly in the bloodstream infections occurred in ICU.

**Disclosure of Interest**: None declared

### O35 HOUSEHOLD TRANSMISSION OF ESBL-PRODUCING ESCHERICHIA COLI OR KLEBSIELLA PNEUMONIAE AFTER HOSPITAL DISCHARGE OF A KNOWN ESBL CARRIER

#### M. E. Riccio^1^, C. Brossier^1^, R. Martischang^1^, G. Renzi^2^, J. Schrenzel^2^, S. Harbarth^1^

##### ^1^Infection Control Program; ^2^Bacteriology Laboratory, Geneva University Hospitals (HUG), Geneva, Switzerland

###### **Correspondence:** M. E. Riccio

**Introduction:** The increasing prevalence of ESBL-producing *Enterobacteriacae* (ESBL-PE) in the community is a cause of concern. ESBL-PE transmission among household members may play an important role in ESBL-PE dissemination, but has been understudied hitherto.

**Objectives:** The aim of this study was to determine the rate of ESBL-PE transmission within households and estimate the rate of spontaneous ESBL-PE decolonization.

**Methods:** This was a prospective, observational cohort study of ESBL-PE household transmission, in the context of the multicenter MODERN project, funded by the Joint Programming Initiative on Antimicrobial Resistance. From Nov 2017 to Nov 2018, ESBL-PE carrying inpatients, and their households’ members, were recruited at HUG. During 4-months follow-up, personal information and 4 stool samples were collected from all participants. ESBL-producing *E. coli* and *K. pneumoniae* isolates were identified after microbiological work-up of stool samples.

**Results:** 22 households (52 participants) were enrolled in Geneva. A total of 100/179 stool samples were positive for ESBL-*E. coli* (n=71) *K. pneumoniae* (n=17) or both (n=12). Both new colonization (n=8) and decolonization (n=12) events were observed during follow-up. The incidence rate of ESBL-PE acquisition in previously ESBL-PE negative household members was 3.59/100 patient-weeks at risk, while the rate of spontaneous ESBL-PE decolonisation among ESBL-PE positive participants was 1.95/100 patient-weeks of follow-up. 12/20 households with complete follow-up had at least one 2^nd^ ESBL-PE-positive household member. A total of 452 ESBL-PE isolates were stored for further molecular analysis. Based on crude phenotypic comparisons, 10 possible transmission (or acquisition) events were identified.

**Conclusion:** 60% of households had secondary ESBL-PE cases. Incidence of ESBL-PE acquisition among family members was higher than the rate of spontaneous loss of carriage. Possible transmission events were observed in 50% of the households based on phenotypical comparison; ESBL-PE clonal relatedness will be determined by WGS.

**Disclosure of Interest**: None declared

### O36 CO-CARRIAGE AND ACQUISITION OF ESBL-PRODUCING ENTEROBACTERIACEAE AMONG HOUSEHOLD MEMBERS: A SYSTEMATIC REVIEW

#### R. Martischang^1^, E. M. Riccio^1^, M. Abbas^1^, A. Stewardson^2^, J. Kluytmans^3^, S. Harbarth^1^

##### ^1^Geneva University Hospital, Geneva, Switzerland; ^2^Monash University and Alfred Health, Melbourne, Australia; ^3^University Medical Center Utrecht, Utrecht, Netherlands

###### **Correspondence:** R. Martischang

**Introduction:** While the epidemiology of ESBL-producing *Enterobacteriaceae* (ESBL-PE) has been extensively studied in hospitals, the data on community transmission is scarce.

**Objectives:** We conducted a systematic review to assess the prevalence of ESBL-PE co-carriage and acquisition rate in households.

**Methods:** We searched Cochrane Library, PubMed, Embase and CINAHL databases for cross-sectional or cohort studies published between 1990 and 2018 evaluating co-carriage proportions and/or acquisition rates of ESBL-PE among household members, with no language restriction. We excluded studies focusing on animal-to-human transmission, non-household settings, or specific settings (e.g. farms, foodborne outbreaks, aboriginal populations). The primary outcomes were co-carriage proportions and acquisition rates, stratified according to the definition of relatedness, which was assessed phenotypically or genotypically. Co-carriage proportions of clonally-related ESBL-PE were transformed via the double-arcsine method and pooled using a random-effects model. Potential biases were assessed manually.

**Results:** We identified 13 eligible studies. Among 770 household members of index patients colonized or infected by an ESBL-PE, prevalence of ESBL-PE co-carriage ranged from 11% to 37% in 10 studies. Overall, 13% (95%CI: 9-16%) had a clonally-related strain. Those proportions were higher for *Klebsiella pneumoniae* (20-25%) compared to *Escherichia coli* (10-20%). Acquisition rates of ESBL-PE among household members of a previously identified carrier ranged between 1.47-18.34 per 1000 person-weeks in 5 studies including 223 initially ESBL-PE free household members. When restricting to clonally-related ESBL-PE, the rates ranged between 2.03-3.92 events in 4 studies with 162 initially ESBL-PE free household members. We identified a high risk of bias, as well as a large amount of heterogeneity between studies.

**Conclusion:** ESBL-PE household co-carriage is frequent, suggesting intrafamilial acquisition. There is a need for further studies evaluating the intrafamily risk of ESBL-PE transmission.

**Disclosure of Interest**: None declared

### O37 RESULTS OF A FIVE-YEAR ADMISSION-SCREENING FOR MULTIDRUG-RESISTANT GRAM-NEGATIVE BACTERIA IN A TERTIARY CARE CENTER IN EASTERN SWITZERLAND

#### E. Lemmenmeier^1^, P. Kohler^1^, O. Nolte^2^, M. Schlegel^1^, W. C. Albrich^1^

##### ^1^Infektiologie und Spitalhygiene, Kantonsspital St.Gallen, 9007 St.Gallen; ^2^Bakteriologie, Mykologie, Parasitologie, Zentrum für Labormedizin, 9001 St.Gallen, Switzerland

###### **Correspondence:** E. Lemmenmeier

**Introduction:** Multidrug-resistant Gram-negative bacteria (MRGN) are a concern worldwide. We analyzed data from a targeted hospital admission screening in order to assess trends over time and to identify risk factors for MRGN colonization.

**Objectives:** Detection of risk factors could lead to a risk-based screening.

**Methods:** Patients hospitalised abroad within the last 6 months were rectally screened after admission. Additional samples were obtained from wounds, urinary catheters, tracheal secretions, if applicable. After enrichment in TSB broth samples were streaked on chromogenic ESBL/OXA-48 screening plates. Susceptibility testing was done with the BD Phoenix^TM^instrument and ESBL/CPE confirmation with phenotypic methods. The presence of the most relevant carbapenemase genes was confirmed by PCR.

MRGN were defined as Gram-negative bacteria producing an ESBL or a carbapenemase. For risk factor analysis, ESBL *E. coli* were excluded. Asia, Africa and Southern/Eastern Europe were regarded as high-risk regions, compared to Australia, America and Western/Northern Europe (low-risk).

**Results:** From 03/13-07/18 458 patients underwent admission screening. 111 (24%) were colonized with MRGN. We found 129 isolates including 21 carbapenemase-producers (16%), 38 non-*E. coli* ESBL (30%) and 70 *E.coli* ESBL (54%). Over time, the proportion of *E. coli* ESBL among all screened patients showed an increasing trend (p=0.09), whereas the other MRGNs remained stable. In univariable analysis, hospitalisation in a high-risk region, central venous and urinary catheters, open wounds, diabetes, antibiotics before screening were identified as risk factors. In multivariable analysis only high-risk region remained significantly associated with MRGN colonization (OR 2.4, 95% CI 1.2-5.0, p=0.017). Among 128 patients hospitalised in low-risk regions, only 4 were colonized with MRGN.

**Conclusion:** Over 5 years, the proportion of detected colonization with carbapenemase-producers and non-*E.coli* ESBL remained stable among patients who were recently hospitalised abroad. The predominant risk factor for colonization was hospitalisation in a high-risk region. A negligible proportion of patients hospitalised in low-risk regions was colonized with MRGN, questioning the utility of our screening program for this population.

**Disclosure of Interest**: None declared

### O38 NO OBVIOUS IMPACT OF CONTACT ISOLATION DISCONTINUATION ON NOSOCOMIAL ESBL-ESCHERICHIA COLI SPREAD

#### B. Clarivet, L. Senn, G. Prod'hom, D. S. Blanc, B. Grandbastien

##### CHUV - centre hospitalier universitaire vaudois, Lausanne, Switzerland

###### **Correspondence:** L. Senn

**Introduction:** The prevalence of Extended-Spectrum β-Lactamase producing *Escherichia coli* (ESBL-EC) has increased over the last 10 years. In order to limit their nosocomial transmission, international guidelines recommended contact isolation for ESBL-EC colonized/infected patients. The relevance of this measure was questioned as the transmission is mainly in the community and, in 2014, Swissnoso advocated its discontinuation.

**Objectives:** The objective of this study was to measure the impact of contact isolation discontinuation on ESBL-EC cases in the Lausanne University Hospital (CHUV).

**Methods:** A case was defined as a first ESBL-EC bacteriuria between January 1, 2007 and December 31, 2018 in a hospitalized patient. We defined two periods: P1 (1st quarter 2007 to 3rd quarter 2012) and P2 (4th quarter 2012 to 4th quarter 2018), after contact isolation discontinuation for ESBL-EC. We used interrupted time series analysis to measure the impact of this strategy on the incidence of cases occurring >48h after admission.

**Results:** During the study period, a total of 1’484 ESBL-EC bacteriuria occurred corresponding to 889 cases. The mean incidence was 0.15/1000 patients-days (pd) in P1 and 0.36/1000 pd in P2 (p<10E4). Among them, 488 (54.9%) cases occurred >48h after admission; their mean incidence was 0.09/1000 pd [0.08 - 0.10] in P1 and 0.19/1000 pd [0.17-0.21] in P2. Time series analysis did not show a significant association between contact isolation discontinuation and changes in the incidence of ESBL-EC bacteriuria occurring >48h after admission (p = 0.47). Similarly, the mean incidence of cases at admission was 0.06/1000 pd (95% CI: [0.05 - 0.07]) in P1 and 0.17/1000 pd [0.15-0.19] in P2.

**Conclusion:** Following the discontinuation of contact isolation, no significant change in the evolution of the incidence of ESBL-EC bacteriuria occurring >48h after admission has been identified. The parallel increase in ESBL-EC bacteriuria detected at admission supports the hypothesis of a predominantly community reservoir.

**Disclosure of Interest**: None declared

### O39 PREVALENCE OF MCR IN SALMONELLA FROM HOSPITAL IN SOUTH CHINA

#### Y. Yu, R. Y. Sun, L. X. Fang, J. Sun, Y. F. Zhou, X. P. Liao, Y. H. Liu

##### College of Veterinary Medicine, South China Agricultural University, Guangzhou, China

###### **Correspondence:** Y. Yu

**Introduction:** Salmonella is a major global foodborne pathogen and different serotypes were identified worldwide. In China, Salmonella causes an estimated 22.2% of foodborne diseases, and the majority of diseases are associated with the ingestion of contaminated meat products.

**Objectives:** The current study aimed to analyze the prevalence and characterization of colistin resistance in Salmonella isolated from hospital in South China.

**Methods:** From 2009 to 2018, totally 6709 Salmonella strains were collected from hospitals in Guangdong province, including *Typhimurium Salmonella* (1867), serotype of 1,4,5,12:i:- (3163), *Enteritidis Salmonella* (1584), *Indiana Salmonella* (32), and *Derby Salmonella* (54). Antimicrobial Susceptibility test was performed especially for colistin.

**Results:** MICs and PCRs shown that 4.41% (296/6709) strains were resistant to colistin and carrying the *mcr* genes, with ratios of 96.28 % (259/269) and 3.71% (10/269) for *mcr-1* and *mcr-3*, respectively. The genomic analysis of the 74 *mcr-1*-bearing *Typhimurium Salmonella* and its variant serotype revealed that the ST34 is the dominant type (70/74), followed by ST19(4/74). The prevalent plasmids carrying *mcr-1* gene are IncHI2(69/74), IncX4(3/74), and IncI2 (2/74). Multi-drug resistance to beta-lactams, FFL, fosfomycin and quinolone was observed as well. Meanwhile, all the 10 *mcr-3*-positive Salmonella strains belong to ST34, and the mcr-3 gene was located on IncA/C-ST3 plasmids (7/10) in size of 140 ~ 160 kb and chromosome (3/10). Interestingly, this IncA/C plasmid is hybridized with IncFII plasmid and dissemination of *mcr-3* is possibly mediated by IS26 or IS15DI.

**Conclusion:** In this study, we found *Typhimurium Salmonella* variant has a higher resistant rate to colistin. Differently from *Escherichia coli*, *mcr-1* genes were mostly carried by IncHI2 plasmid instead of IncX4 plasmid, and for *mcr-3* was bearing by IncA/C plasmid most frequently. Being one of the Highest Priority Critically Important Antimicrobial for human medicine, colistin is often used as the last therapy available for serious bacterial infections in clinics. The discovery of plasmid-borne *mcr* genes in *Salmonella* poses a threat to public health globally and further prevent and control strategy is definitely needed.

**Disclosure of Interest**: None declared

### O40 ASSOCIATION OF ULTRAVIOLET-C ENHANCED TERMINAL ROOM DISINFECTION WITH HOSPITAL-ONSET GRAM-NEGATIVE BLOODSTREAM INFECTION: NATIONWIDE STEP-WEDGE TIME-SERIES ANALYSIS

#### M. Goto, E. C. Balkenende, G. S. Clore, R. Nair, E. N. Perencevich, on behalf of VA-CDC Practice-Based Research Network

##### Internal Medicine, University of Iowa/Iowa City VAMC, Iowa City, United States

###### **Correspondence:** M. Goto

**Introduction:** The role of the hospital environment is increasingly recognized in the transmission of Gram-negative rod (GNR) pathogens. Enhanced terminal room cleaning with ultraviolet C (UVC) disinfection has become more commonly used as a strategy to reduce the incidence of *Clostridioides difficile* or vancomycin-resistant enterococci, but its effectiveness in reducing GNR infections has not been evaluated.

**Objectives:** We aimed to assess the association of UVC disinfection during terminal cleaning with the incidence of hospital-onset (HO) GNR bloodstream infection (BSI) within the nationwide Veterans Health Administration (VHA) System in the United States.

**Methods:** We obtained information regarding UVC disinfection use and timing of implementation at each hospital through a survey of all acute care hospitals within the VHA system. Episodes of HO GNR BSI (defined as at least 48 hours of acute inpatient stay before the first positive blood culture for GNR) between 1/2010 and 12/2018 were identified, and bed days of care (BDOC) was used as the denominator. We analyzed the association of UVC disinfection with incidence rates of HO GNR BSI using a non-randomized, step-wedge design, using negative binomial regression model with hospital-specific random intercept, the presence or absence of UVC disinfection use for each month, baseline trend, and seasonality as explanatory variables.

**Results:** Among 143 VHA acute care hospitals, 136 hospitals (95%) responded to the survey and were included in the analysis. UV-C use was reported from 42 hospitals with various implementation start dates (range: 6/2010-6/2017). We identified 14,427 episodes of HO GNR BSI and 25,614,888 BDOC from the 136 hospitals during the study period. In addition to a baseline declining trend (-0.42% per month), UV-C use was associated with a lower incidence rate of HO GNR BSI (incidence rate ratio: 0.915; 95% confidence interval: 0851-0.984; p=0.016).

**Conclusion:** In this large quasi-experimental analysis within the VHA System, the enhanced terminal room cleaning with UVC disinfection was associated with an 8.5% lower incidence of HO GNR BSI. This finding suggests that UVC disinfection at time of patient discharge is a potentially effective component in infection prevention bundles targeting HO GNR BSI.

**Disclosure of Interest**: None declared

## Slide session: Chlorhexidine baths and mouthwashes

### O41 EFFECT OF DAILY CHLORHEXIDINE BATHING ON HOSPITAL-ACQUIRED INFECTION IN INTENSIVE CARE UNITS

#### F. A. Van Laer, E. Van Cauwenberg, H. Jansens

##### Infection Control, Antwerp University Hospital, Edegem, Belgium

###### **Correspondence:** F. A. Van Laer

**Introduction:** This study presents the results of the effect of daily chlorhexidine 2% (CHX) bathing on the incidence of hospital-acquired multidrug resistant micro-organisms (HA-MDRO) and central line associated bloodstream infections (CLABSI) in intensive care units (ICU) in the Antwerp University Hospital.

**Objectives:** To evaluate the impact of daily chlorhexidine bathing on HA-MDRO en CLABSI.

**Methods:** In July 2014 bathing the patients with CHX-impregnated washcloths was introduced in all five intensive care units (a total of 45 beds). The mean incidence of HA-MDRO per 1000 patient days before the introduction of the CHX-wascloths from 2006 to June 2014 was compared with the mean incidence of HA-MDRO after introduction of the CHX-washcloths from July 2014 to the end of 2018. The incidence of CLABSI per 1000 catheter-days from January 2014 to June 2014 was compared with the incidence from July 2014 to the end of 2016. After 2016 other preventive measures (i.e. the use of needles connectors, disinfecting caps for connectors,…) were implemented to reduce CLABSI.

**Results:** The incidence of MDRO was 6.51 per 1000 patient-days (from 2006 to June 2014) versus 2.94 per 1000 patient-days (from July 2014 to the end of 2018) after introduction of CHX-impregnated washcloths, the equivalent of a 54,8 % lower rate. There was just a small decrease of HA-MRSA acquisition from 0,31 to 0,28 per 1000 patient-days, the equivalent of a 9.77% lower rate. The incidence of CLABSI in the first six months of 2014 was 2,92 per 1000 catheter-days and decreased after introduction of CHX-washcloths to 2,24 per 1000 catheter-days, the equivalent of a 23,4% lower rate. From 2017 to the end of 2018, after introduction of other additional preventive measures, there was a further decrease in the incidence of CLABSI to 1,32/1000 catheter-days, the equivalent of a 45,2% lower rate compared with the incidence in 2014.

**Conclusion:** Daily bathing with chlorhexidine-impregnated washcloths reduced the risk of acquisition of MDRO and development of CLABSI in ICU.

**Disclosure of Interest**: None declared

### O42 IMPACT OF CHLORHEXIDINE BATHS ON SUSPECTED SEPSIS AND BLOODSTREAM INFECTIONS IN HOSPITALIZED NEONATES IN ZAMBIA

#### T. Westling^1^, C. Cowden^2^, L. Mwananyanda^3^, C. Pierre^4^, D. Hamer^5^, S. E. Coffin^2^

##### ^1^Biostatistics and Epidemiology, UPenn School of Medicine; ^2^Pediatrics, Children's Hospital of Philadelphia, Philadelphia, United States; ^3^Right to Care, Lusaka, Zambia; ^4^Internal Medicine, Boston Medical Center; ^5^Internal Medicine, Boston University, Boston, United States

###### **Correspondence:** S. E. Coffin

**Introduction:** Sepsis is the leading cause of infectious morbidity and mortality among hospitalized neonates. In high-resource pediatric and adult intensive care units, use of aqueous chlorhexidine (CHG) solution has been associated with reduced risk of bloodstream infections (BSI).

**Objectives:** To assess the impact of admission bathing of neonates with 2% CHG on BSI, sepsis, and mortality in a low-income hospital setting.

**Methods:** We conducted a secondary analysis of data from the Sepsis Prevention in Neonates in Zambia (SPINZ) study, a prospective observational cohort study performed at a large public referral hospital in Lusaka, Zambia. The SPINZ study assessed the impact of an infection control bundle (consisting of alcohol hand rub, SMS hygiene reminders, enhanced environmental cleaning, and CHG baths) on sepsis, BSI, and all-cause mortality. Episodic shortages in study staffing resulted in some enrolled babies not receiving a CHG bath. Using the Mantel-Haenszel log-rank test to compare the stratified Kaplan-Meier curves,we compared inborn babies enrolled during the study intervention phase who did and did not receive a CHG bath within the first 3 days of life.

**Results:** The majority of inborn, enrolled babies >1.5 kg received a CHG bath within 3 days of NICU admission (864 of 1233, 70%). Using survival analysis, we found that neonates who received a CHG bath within the first three days of admission had significantly lower rates of suspected sepsis (p=0.0003) and BSI due to a pathogenic organism (p=0.001), but not statistically significant lower rates of death (p=0.07). Comparing CHG-bathed to not-bathed babies, the hazard ratio for suspected sepsis was 0.66 (CI: 0.53, 0.83; p=0.0003), for BSI due to a pathogen was 0.46 (CI: 0.29, 0.77; p=0.002), and for death was 0.77 (CI: 0.58, 1.03; p=0.08).

**Conclusion:** In our single center study, CHG bathing at admission was associated with a reduced risk of suspected sepsis and BSI due to a pathogenic organism. Future analysis is required to determine whether this association persists after adjustment for severity of illness and likelihood to receive a CHG bath.

**Disclosure of Interest**: None declared

### O43 CHLORHEXIDINE ORAL TOPICAL APPLICATION FOR CRITICAL PATIENTS: SAVIOR OR KILLER?

#### W. T. Bellissimo-Rodrigues, M. G. Menegueti, M. G. Mussolin, L. D. Macedo, A. Basile-Filho, R. Martinez, J. P. Souza, F. Bellissimo-Rodrigues

##### Ribeirão Preto Medical School, University of São Paulo, Ribeirão Preto, Brazil

###### **Correspondence:** F. Bellissimo-Rodrigues

**Introduction:** Chlorhexidine (CHX) oral topical application has been extensively used for preventing respiratory tract infections among critical patients, despite controversial effectiveness demonstrated in different clinical trials. More recently, this practice has been found suspect of enhancing mortality in hospitalized patients, for reasons not clearly understood. Acute Respiratory Distress Syndrome (ARDS) was hypothesized as a pathway for this association.

**Objectives:** To reassess data from a clinical trial evaluating oral care in the intensive care unit (ICU), in order to search for potential pathways for the CHX-associated mortality.

**Methods:** This is a post-hoc analysis of a randomized clinical trial (RBR-89CP93) evaluating a dental care intervention aimed to prevent respiratory tract infections in the ICU setting, funded by two non-profit foundations (FAPESP and FAEPA). We analyzed data from adult patients who were assigned to receive dental care provided by a dentist (experimental group) or routine oral care provided by the nursing staff (control group). Both groups used 0.12% CHX oral solution, if fully conscious, or 2% CHX oral gel, if unconscious, three times a day throughout their ICU stay. Adverse events potentially related to CHX use were reassessed and their relationship with in-ICU death was evaluated through a logistic regression model.

**Results:** Among the 254 patients included, 18 (7.09%) developed CHX-induced oral mucositis, which was independently associated with age (OR=1.05; 95%CI: 1.02-1.09) and the intervention (OR=6.53; 95%CI: 1.74-24.48), and inversely associated with being edentulous (OR=0.09; 95%CI: 0.02-0.45). On the other hand, CHX-induced oral mucositis was an independent risk factor for death (OR= 5.62; 95%CI: 1.94-16.25) and death due to respiratory tract infections (OR=3.27; 95%CI: 1.18-9.08). No death due to ARDS was reported.

**Conclusion:** CHX-induced oral mucositis was found to be a relevant risk factor for death in this clinical trial and may be a clinical pathway explaining why CHX enhances mortality among critical patients. Intensivists should be very cautious when prescribing CHX for critical patients and should immediately suspend it when any sign of oral mucositis is observed.

**Disclosure of Interest**: None declared

## Slide session: Outbreaks and late breakers

### O44 QUELLING CANDIDA AURIS OUTBREAK: THE C AURIS PREVENTION PROTOCOL - THE FIGHT BEYOND MEDICINE

#### K. Alexander, S. Brown, W. Javaid, S. Lorin, J. Ehni, D. Mazo, B. Camins, B. Koll

##### Infection Prevention and Control, Mount Sinai Health System, Brooklyn, United States

###### **Correspondence:** K. Alexander

**Introduction:** Identifying routes of transmission among hospitalized patients during a C. *auris* healthcare-associated outbreak can be tedious, particularly among patients with prolonged and complex hospital stays. Currently there are few data available on the effectiveness of interventions to prevent and control the transmission of C. *auris*. Engagement, education, execution, and evaluation were the cornerstones used by a hospital in New York to develop a C. *auris* prevention protocol (CAP).

**Objectives:** To evaluate the post-implementation of the CAP to quell the transmission of C. *auris* during a period of high incidence in a New York City Hospital.

**Methods:** A review of patients with C. *auris* from January 2016 to April 2019 at our hospital was conducted after the CAP bundle was implemented January 2017. The bundle included four elements: engagement, education, execution and evaluation. Education and engagement strategies included multidisciplinary work, debriefing sessions, live simulations, and leadership involvement. Execution strategies standardized the interventions into simple tasks to facilitate seamless comprehension. These consisted of hand hygiene, special contact precaution, dedicated staff and equipment, routine terminal cleaning of a patient’s room and equipment with bleach, secure waste and linen disposal, and hospital-wide communication. Evaluation strategies used a benchmark approach to appraise adherence with interventions and patient outcomes. Cleaning compliance was monitored and 100% compliance was required. This was followed by UV light disinfection.

**Results:** A total of 42 patients in our facility from January 2016 to April 2019 were noted to be colonized/infected with C. *auris*. Of the 42, 5 patients were noted to be associated with exposure to in our facility. Findings reveal that there were no new C. *auris* cases when compliance to cleaning methods was above average (76.9%) and execution of special contact precaution and hand hygiene was above average (69.2%).

**Conclusion:** The CAP bundle fortified multidisciplinary teamwork, increased staff/patient knowledge, standardized and simplified infection prevention processes, and created a verification and feedback process. The lessons learned at our facility maybe applicable to other hospitals facing the burden of C. *auris* around the globe.

**Disclosure of Interest**: None declared

### O45 FIRST REPORTED NOSOCOMIAL OUTBREAK OF NDM-1 PRODUCING ESCHERICHIA COLI IN SWITZERLAND

#### R. Martischang, M.-N. Chraïti, V. Lazarevic, N. Gaïa, C. Bandiera-Clerc, H. Soule, G. Renzi, A. Iten, C. Ginet, D. Pittet, J. Schrenzel, S. Harbarth

##### Geneva University Hospital, Geneva, Switzerland

###### **Correspondence:** R. Martischang

**Introduction:** Since 2008, NDM-producing *Enterobacteriaceae* has spread globally. In late 2017, a patient transferred from Dubai was identified as NDM-producing *E.coli* carrier, and placed under contact precautions during two hospital stays at HUG in Jan & Jul 2018. Between Nov 2018 and May 2019, 3 secondary cases who had not travelled outside Switzerland for the past 12 months were found colonized with NDM-producing *E. coli* by routine screening swabs or urine cultures. Nosocomial cross-transmission was strongly suspected.

**Objectives:** We report an outbreak investigation guided through molecular approaches.

**Methods:** Roommates’ screening (July & Nov 18, May 19), and environmental screening and disinfection (May 19) in the concerned patient room were performed. Following Illumina iSeq sequencing, the relatedness between 4 NDM isolates was assessed by cgMLST and cgSNP analyses.

**Results:** Spatiotemporal analyses identified the simultaneous passage of 2 patients in a newly opened surgical step-down unit in July 18, and staggered passage of 3 patients in the same room on a private floor from Nov 18 through Apr 19. As of today (May 25), 20 environmental samples and all further contact screening swabs have been negative. Sequencing analysis confirmed cross-transmission with *E. coli* ST354 NDM-1 (<10SNPs). Standard precautions were reinforced in the concerned units. We implemented a computerized readmission alert system of all contact patients with potential exposition, requiring mandatory screening at re-admission. One of the patients died of surgical complications unrelated to *E. coli* NDM-1 carriage.

**Conclusion:** To our knowledge, this cluster represents the first nosocomial NDM-producing *E. coli* outbreak in Switzerland, with late outbreak detection due to hidden transmission despite strict contact precautions for the index case. This *E. coli* ST354 clone has so far mostly been reported from animals, and was rarely associated with carbapenemases. This outbreak confirms the high nosocomial transmission potential of these ultraresistant *Enterobacteriaceae*.

**Disclosure of Interest**: None declared

### O46 MEASLES OUTBREAK IN HONG KONG – FROM THE HONG KONG INTERNATIONAL AIRPORT TO THE HOSPITAL

#### S. C. WONG^1^, V. CHENG^1^, K. Y. YUEN^2^

##### ^1^INFECTION CONTROL UNIT, QUEEN MARY HOSPITAL; ^2^DEPARTMENT OF MICROBIOLOGY, THE UNIVERSITY OF HONG KONG, Hong Kong, Hong Kong

###### **Correspondence:** S. C. WONG

**Introduction:** Global resurgence of measles resulted in outbreaks in international airports, communities, and hospitals. However, a direct linkage of measles transmission from airport to hospital has not been well described.

**Objectives:** We reported an outbreak of measles with a clear epidemiological link from Hong Kong International Airport (HKIA) to the hospital.

**Methods:** Epidemiological investigation for the outbreaks in the HKIA and Hospital A were conducted. Infection control preparedness to prevent nosocomial transmission of measles in 43 hospitals under the governance of Hospital Authority included isolation of suspected case with epidemiological link to airborne infection isolation rooms, contact tracing for exposed patients and healthcare workers (HCWs), provision of MMR vaccination, and timely education forum to HCWs. Environmental and air samples were tested for measles RNA. Phylogenetic analysis of hemagglutinin gene of measles virus isolates collected from infected case in the HKIA, Hospital A, and the community was analyzed.

**Results:** Twenty-nine staff working at the HKIA of diverse rank and working location were infected with measles from 4 March 2019 to 3 April 2019. A significantly lower proportion of affected staff had history of travel compared with non-HKIA related measles cases in Hong Kong (9/29, 31% vs 27/36, 75%, p<0.01). During their incubation period, seven (70%) of 10 staff who could recall the exposure history had visited self-serviced food premises at the HKIA. The food trays were not adequately disinfected after use as observed during the epidemiological field investigation, although measles RNA was undetectable in the environmental and air samples. One baggage handler was admitted to a general ward in Hospital A before the onset of rash, with two HCWs, who had received two doses of MMR vaccine, being infected requiring subsequent contact tracing of 168 persons (97 patients and 71 HCWs). Phylogenetic analysis showed that the measles virus isolated in two infected HCWs was closely related to the HKIA outbreak strain, which was genotype B3.

**Conclusion:** Pre-exantham transmission of measles poses a great challenge to the hospital infection control. Breakthrough infection of measles may also occur in vaccinated HCWs.

**Disclosure of Interest**: None declared

## Slide session: MRSA-VRE

### O47 GENOMIC SURVEILLANCE OF METHICILLIN-RESISTANT STAPHYLOCOCCUS AUREUS: A MATHEMATICAL EARLY MODELLING STUDY OF COST EFFECTIVENESS

#### A. Dymond^1^, H. Davies^1^, S. Mealing^1^, V. Pollit^1^, F. Coll^2^, N. M. Brown^3^, S. J. Peacock^4^

##### ^1^York Health Economics Consortium (YHEC), York; ^2^London School of Hygiene & Tropical Medicine, London; ^3^Cambridge University Hospitals NHS Foundation Trust; ^4^Department of Medicine , University of Cambridge, Cambridge, United Kingdom

###### **Correspondence:** A. Dymond

**Introduction:** Used proactively, genomic surveillance of methicillin-resistant *Staphylococcus aureus* (MRSA) could direct early and highly targeted infection control interventions to prevent ongoing spread.

**Objectives:** Here, we evaluate the cost-effectiveness of this intervention in a model that compared whole genome sequencing plus current practice versus current practice alone.

**Methods:** A UK cost-effectiveness study was conducted using an early model from the perspective of the National Health Service (NHS) and personal social services. Effectiveness of sequencing was based on the relative reduction in total MRSA acquisitions in a cohort of hospitalised patients in the year following their index admissions. Resource use and costs were reflective of the UK NHS. Sensitivity analysis was used to illustrate and assess the level of confidence associated with the conclusions of our economic evaluation.

**Results:** A cohort of 65,000 patients were ran through the model. Assuming that sequencing would result in a 90% reduction in MRSA acquisition, 290 new MRSA cases were avoided. This gave an absolute reduction of 28.8% and avoidance of five MRSA-related deaths. Base case results indicated that the use of routine, proactive MRSA sequencing would be associated with estimated cost savings of over £728,290 per annual hospitalised cohort. The impact in total quality adjusted life years (QALYs) was relatively modest, with sequencing leading to an additional 14.28 QALYs gained. Results were most sensitive to the probability of an MRSA negative patient acquiring MRSA during their hospital admission.

**Conclusion:** We showed that proactive genomic surveillance of MRSA is likely to be cost-effective, with the model results indicating that routine MRSA sequencing would result in fewer MRSA cases and have a small, positive impact on health-related quality of life.

Routine prospective MRSA sequencing could be used to detect outbreaks and prevent unnecessary action in the event of a pseudo-outbreak. Further evaluation is required in the context of a prospective study.

**Disclosure of Interest**: A. Dymond Employee of: York Health Economics Consortium, H. Davies Employee of: York Health Economics Consortium, S. Mealing Employee of: York Health Economics Consortium, V. Pollit Employee of: York Health Economics Consortium, F. Coll Consultant for: Next Gen Diagnostics LLC, N. Brown: None declared, S. Peacock Grant/Research support from: Health Innovation Challenge Fund, Consultant for: Specific Technologies and Next Gen Diagnostics LLC

### O48 DIAGNOSTIC ACCURACY AND PREDICTION OF FOLLOW-UP POSITIVE CULTURES BY XPERT®-VANA/VANB ASSAY FROM A SELECTIVE ENRICHMENT BROTH DURING A VANB VANCOMYCIN-RESISTANT ENTEROCOCCUS FAECIUM OUTBREAK

#### V. Piezzi^1^, A. Lüthi^2^, F. Suter-Riniker ^2^, C. Casanova^2^, S. Droz^2^, J. Marschall^1^, S. L. Leib^2^, R. Sommerstein^1^, P. Bittel ^2^

##### ^1^Infectious Diseases, Bern University Hospital; ^2^Institute for Infectious Diseases, University of Bern, Bern, Switzerland

###### **Correspondence:** V. Piezzi

**Introduction:** Rapid and precise diagnosis of newly colonized patients during an outbreak with vancomycin-resistant enterococci (VRE) is of utmost importance for infection control. Currently, molecular-based methods lack specificity to identify *vanB*-VRE from rectal swabs. Additionally, it is unknown whether a molecular-based method can predict a positive culture in follow-up swabs.

**Objectives:** The aim of this study was to answer these questions by testing swabs following inoculation in a selective enrichment broth.

**Methods:** Prospective study between July and August 2018 during an v*anB*-VRE outbreak. All consecutive rectal swabs were tested by both conventional culture and a molecular-based method. Swabs were inoculated in a selective enrichment broth (4.5 mg/L vancomycin, 2 mg/L meropenem, 16 mg/L amoxicillin) at 35°C for 20-25 hours. We then used the Xpert®-vanA/vanB assay to detect the *vanB* gene. For conventional culture-based testing, the broths were plated on selective/chromogenic plates. We identified colonies by MALDI-TOF MS and confirmed the presence of *vanB* with the Xpert®-vanA/vanB assay. For data analysis we used *pROC package* in R.

**Results:** We included 597 rectal swabs from 396 patients. 32/597 swabs (5.4%) were culture-positive *vanB*-VRE*.* Sensitivities and specificity, using culture as the gold standard, per PCR cycling time threshold are shown in Figure 1. The calculated ROC area under the curve was 0.99 (95%CI: 0.98-1). The negative predictive value at a cycling time of 33 was 99% (95%CI: 98%>100%).

128 (32%) patients had a median of 1 (range 1-4) follow-up screenings. In 6/128 (4.7%) patients with >1 screen, a follow-up swab was culture-positive. Preceding cycling time values were not indicative of subsequent culture positivity (Figure 2).

**Conclusion:** The use of the Xpert®-vanA/vanB assay with prior enrichment in selective broth yielded an excellent specificity for the diagnosis of *vanB*-VRE and a high negative predictive value for ruling it out. During an outbreak this approach is attractive in that it can quickly exclude VRE carriers. However, the molecular method is unable to predict culture-positive VRE in follow-up swabs.

**Disclosure of Interest**: None declared

### O49 IMPACT OF THE MRSA PREVENTION PROGRAM ON ISOLATION OF MRSA FROM CLINICAL CULTURES AMONG LONG-TERM CARE RESIDENTS IN THE US DEPARTMENT OF VETERANS AFFAIRS HEALTHCARE SYSTEM

#### V. W. Stevens^1^, M. Z. David^2^, M. Jones^1^, D. Linkin^3^, M. E. Evans^4^, R. E. Nelson^1^, N.-C. N. Chang^1^, W. Bilker^2^, T. M. Willson^1^, E. Lautenbach^2^

##### ^1^IDEAS Center of Innovation, VA Salt Lake City Health Care System, Salt Lake City; ^2^University of Pennsylvania; ^3^Philadelphia VA Medical Center, Philadelphia; ^4^National Infectious Diseases Service, US Department of Veterans Affairs, Lexington, United States

###### **Correspondence:** V. W. Stevens

**Introduction:** In 2009, the US Department of Veterans Affairs (VA) expanded its comprehensive methicillin-resistant *Staphylococcus aureus* (MRSA) prevention program to all long-term care (LTC) facilities.

**Objectives:** The objective of this study was to examine the impact of the program on the incidence of healthcare-associated MRSA (HA-MRSA) isolated from clinical cultures in LTC residents

**Methods:** We conducted a retrospective interrupted time series analysis of the monthly rates (per 10,000 resident-days) of HA-MRSA isolated from clinical (i.e., non-surveillance) cultures in VA LTC residents between 1 January 2005 and 30 September 2015. Coagulase negative Staphylococci (CoNS) bloodstream cultures were used as a non-equivalent dependent variable, defined as two positives from different samples in <24 hours. Facilities with at least 12 months of pre- and post-intervention data were included. We used segmented Poisson regression to separately estimate the intercept and slope changes from pre-intervention (1 January 2005 to 31 Dec 2008) to intervention rollout (1 January 2009 to 30 June 2009) and intervention (1 July 2009 to 30 September 2015) periods.

**Results:** Prior to the intervention, the HA-MRSA rate was significantly declining (b=-.004, p=0.001). There was no change in the slope during the rollout or post-intervention periods (p=.54 and p=0.22, respectively). No significant level (intercept) changes were observed during the same periods (p=0.58 and p=0.91, respectively). Rates of CoNS were stable before and after the intervention

**Conclusion:** We found a decreasing HA-MRSA trend but no further statistical decrease in trend associated with the expansion of the MRSA initiative to LTC facilities. Future work will assess whether the observed downward trend is due to decreasing importation following the implementation of the program in acute care facilities in 2007 or other factors, and account for hand hygiene and environmental cleaning activities.

**Disclosure of Interest**: None declared

### O50 EVALUATING THE COST-EFFECTIVENESS OF DECOLONIZATION FOR PREVENTION OF MRSA INFECTIONS USING A DYNAMIC TRANSMISSION MODEL

#### R. E. Nelson^1^, W. Ray^1^, M. A. Rubin^1^, M. Schweizer^2^

##### ^1^IDEAS Center of Innovation, VA Salt Lake City Health Care System, Salt Lake City; ^2^Iowa City Veterans Affairs Health Care System, Iowa City, United States

###### **Correspondence:** R. E. Nelson

**Introduction:** All Veterans admitted to a VA hospital are tested for MRSA carriage and positive patients are placed in contact precautions. An additional strategy for prevention of MRSA transmission is decolonization using an antimicrobial agent such as mupirocin, chlorhexidine, or povidone iodine.

**Objectives:** Our goal was to perform a cost-effectiveness analysis (CEA) of adding decolonization to the current strategies using a dynamic, agent-based simulation model.

**Methods:** Our simulation model tracked patients according to MRSA carriage, clinical infection, and detection status and their movement through 3 wards (intensive care unit, surgery, or other) in an acute care hospital over a 1-year period (roughly 22,000 inpatient admissions). We assumed patient-to-patient transmission of MRSA was more common between patients in the same ward than different wards. Rates of MRSA acquisition were calibrated to data from VA acute care hospitals from 2007-2015 and we varied the assumed effectiveness of decolonization in eradicating MRSA from 0-100%. For the CEAs, the effectiveness outcomes were life-years (LYs) gained and HAIs prevented and costs were taken from the VA perspective. We used values for the pre-discharge cost ($24,726, 95% CI: $11,204-$38,249), post-discharge cost ($11,676, 95% CI: $6,260-$17,091), and relative risk of mortality (2.77, 95% CI: 2.39-3.21) associated with MRSA HAIs from published studies using VA data. We ran 10,000 iterations of the model with and without decolonization drawing parameter values from pre-specified distributions.

**Results:** Decolonization was dominant (i.e., both more effective and less costly) relative to the VA’s current MRSA Initiative in 43.7%, 74.9%, 96.6%, and 99.8% of the 10,000 iterations for which decolonization effectiveness ranged from 0-25%, 25-50%, 50-75%, and 75-100%, respectively, for the HAIs prevented effectiveness measure. For the LYs effectiveness measure, it was cost-effective at a willingness-to-pay threshold of $100,000/LY in 54.3%, 56.8%, 61.4%, 64.5% of the 10,000 iterations of the same ranges for decolonization effectiveness.

**Conclusion:** Our CEA results using a dynamic transmission model suggest that decolonization of MRSA carriers may be a cost-effective strategy for prevention of MRSA transmission and infections in the VA healthcare system.

**Disclosure of Interest**: None declared

### O51 METHICILLIN-RESISTANT STAPHYLOCOCCUS AUREUS (MRSA) PREVALENCE AMONG HEALTHCARE WORKERS (HCW) IN CONTACT TRACINGS IN A DUTCH TEACHING HOSPITAL, 2010-2017

#### V. Weterings^1,2^, H. Kievits^1^, J. Kluytmans^1,3,4^

##### ^1^Infection control, AMPHIA HOSPITAL, Breda; ^2^Medical Microbiology, Radboud University Medical Centre, Nijmegen; ^3^Center for Health Sciences and Primary Care, UMC Utrecht, Utrecht, Utrecht; ^4^Microvida Laboratory for Microbiology, AMPHIA HOSPITAL, Breda, Netherlands

###### **Correspondence:** V. Weterings

**Introduction:** In The Netherlands, the national guideline on MRSA prevention and control advocate screening of HCW after unprotected contact to MRSA carriers. Although this strategy is successful, contact tracing of staff is time consuming and costly.

**Objectives:** We evaluated our contact tracing policy for HCW over the years 2010 – 2017.

**Methods:** This retrospective, observational study was performed in a large Dutch teaching hospital. In accordance with the national guideline, all HCW who had been in close and unprotected contact with an MRSA carrier were included in contact tracing. When there had been a long period of unprotected admission prior to an MRSA finding, or when the index case was a HCW, than the entire (nursing) team was tested.All samples of HCW who were tested for MRSA carriage as part of contact tracing from 2010 until 2017 were included. A pooled nose, throat and perineum swab was collected using the eSwab medium (Copan) and inoculated on chromID MRSA agar plates (bioMérieux) after enrichment in a broth. Molecular typing was performed using multiple locus variable number of tandem repeat analysis (MLVA).

**Results:** In total, MRSA carriage was assessed in 8,142 samples (range: 728 – 1,448 samples per year) from 287 contact tracings (range: 26 – 55 contact tracings per year). Thirty HCW were colonized with MRSA (0.37%; 95%CI 0.26 – 0.53). None of them developed a clinical infection. Eight HCW (0.10%;95%CI 0.05 – 0.19) were colonized with the same MLVA type as the index case, and were detected in 6/287 contact tracings (2%). Notable, a different MLVA type as the index case was found in 22 HCW (0,27%; 95%CI 0,18 – 0,41) of which 7/22 HCW (31.8%) were intermittent carriers.

**Conclusion:** This study shows that when MRSA contact tracing is performed according to the national guideline only 1 out 1000 samples results in a secondary case. This is similar to the population carriage rate of MRSA in The Netherlands. More frequently an unrelated strain is found. These findings raise question marks regarding the validity of the current strategy to perform contact tracing after unprotected exposure.

**Disclosure of Interest**: None declared

### O52 VRE SCREENING PROGRAM AND COSTS OF THREE VRE OUTBREAKS 2012, 2015 AND 2018

#### I. Pruis^1^, A. Sandijck^1^, A. Burggraaf^1^, P. Smit^1^, J. V. D. Stel^2^, M. Damen^1^

##### ^1^Department of Medical Microbiology and Infection Prevention, ^2^Department of quality and safety, Maasstad Hospital, Rotterdam, Netherlands

###### **Correspondence:** I. Pruis

**Introduction:** During 2012, 2015 and 2018, three outbreaks with Vancomycin resistant Enterococcus faecium (VRE) occurred at Maasstad hospital, Rotterdam, The Netherlands.

**Objectives:** The main objective of this study was to evaluate the response to these outbreaks. The response including, a cost/benefit analysis of our VRE screening program was performed.

**Methods:** Our VRE screening program involved weekly testing for all >7 days-hospitalized patients. Rectal swabs were screened with PCR after incubation in enrichment broth. If indicated, VRE strains were typed with Multiple Loci Sequence Typing (MLST). The costs of the VRE outbreaks were estimated as well as the costs of our VRE screening program.

**Results:** In 2012, VRE outbreak occurred with a ST712 strain among 30 positive patients/568 screened on 7 departments (7 months). After the outbreak, the screening program was implemented.In 2015, VRE outbreak occurred with a ST18 strain among 5 positive patients/161 screened on 3 departments (4 months). This outbreak was discovered due to the VRE screening program.In 2018, VRE outbreak occurred with a ST612 strain among 55 positive patients/1300 screened on 7 departments (5 months).Transmission of ST612 VRE in 2018 was undetected for 3 months due to an impaired screening program (implementing a new electronic patient record).Many measures were taken in the 2018 outbreak; intensified screening/typing (€ 73.856), extended isolation precautions (€ 102.500), upgraded cleaning and disinfection (€ 492.000), overtime for employees of infection prevention and laboratory (€ 55.500) and extra nursing staff (€12.936). For the 2018 outbreak, the costs were estimated to be € 740.000 (excluding closing beds, discarded medication, sickleave of staff, reputation damage and other non-material losses). Extrapolating the 2018 outbreak costs to previous outbreaks costs are estimated to be € 199.000 (2015) and € 800.000 (2012). The cost of our VRE screening program is € 107.000/year.

**Conclusion:** The VRE screening program costs seem to outweigh the costs of outbreak control measures. Early detection of VRE is important to limit the size of the outbreak. If the screening program was not hampered in 2018, the outbreak could have potentially been limited to the 2015 outbreak size. Based on a very crude estimate, this would have saved at least € 300.000.

**Disclosure of Interest**: None declared

## Poster Session: Device associated blood-stream infections 1

### P1 A CLUSTER OF PSEUDOMONAS AERUGINOSA BACTEREMIA IN IMMUNOCOMPROMISED PATIENTS IN A TERTIARY CARE CENTER IN LEBANON

#### N. K. Zahreddine^1^, R. AHMADIEH^1^, J. TANNOUS^1^, Z. KANAFANI^2^, S. KANJ^2^

##### ^1^Infection Control and Prevention Program; ^2^INFECTIOUS DISEASES, AMERICAN UNIVERSITY OF BEIRUT MEDICAL CENTER, Beirut, Lebanon

###### **Correspondence:** N. K. Zahreddine

**Introduction:** *Pseudomonas aeruginosa* is an opportunistic pathogen that cause serious conditions in immunocompromised patients. Contaminated water systems have been reported to contribute to *P. aeruginosa* transmissions in healthcare settings.

**Objectives:** The purpose of this study is to investigate the source of a cluster of *P. aeruginosa* bacteremia in immunocompromised patients.

**Methods:** An ongoing prospective surveillance is conducted in the oncology floor at the American University of Beirut Medical Center. Central Line Blood Stream Infections (CLABSI) was diagnosed using the CDC/NHSN (Centers for Disease Control and Prevention/National health and Safety Network) methodology.

Water samples and environmental cultures from patients’ rooms and sinks were collected when a potential environmental source for CLABSI was suspected. Relatedness of environmental results to CLABSI organisms was studied using phenotypic Antimicrobial Susceptibility (AMS) profiles and molecular typing methods using random amplification of polymorphic DNA (RAPD). Elements of the CLABSI prevention bundle were reemphasized for staff assigned to these units.

**Results:** Three patients developed CLABSI with *P. aeruginosa* within a period of 2 weeks during October 2018. One water culture grew *P. aeruginosa*, whereas negative results were reported from all the remaining environmental cultures during the same period. While molecular typing results showed identical RAPD patterns in both the clinical and the environmental isolates, substantial differences were noted in the phenotypic AMS profiles/patterns among the same isolates suggesting that these isolates are not related.

**Conclusion:** Hospital water systems can be potentially contaminated with *P. aeruginosa* and may cause infections in vulnerable patients in healthcare settings. The investigation of this cluster did not reveal the source but was halted following infection control interventions at multiple levels.

The isolated organisms were not thought to be related based on different AMS despite their identical RAPD patterns. Such findings dictate the need for more sensitive and discriminatory molecular test such as Pulse Field Gel Electrophoresis (PFGE), multilocus sequence typing (MLST) or Maximum-entropy Linear Discriminant Analysis (MLDA).

**Disclosure of Interest**: None declared

### P2 FOUR- YEAR RESISTANCE PATTERNS OF GRAM-NEGATIVE BACTERIA ISOLATED FROM BLOODSTREAM INFECTIONS AT A TERTIARY HOSPITAL IN IRAN

#### G. Pouladfar, Z. Jafarpour, B. Pourabbas, A. Abbasian, M. Anvarinejad, P. Abbasi, M. Hoseini, M. A. Dehyadegari, M. A. Shahidi

##### Professor Alborzi Clinical Microbiology Research Center, Shiraz University of Medical Sciences, Shiraz, Iran, Islamic Republic Of

###### **Correspondence:** G. Pouladfar

**Introduction:** Antimicrobial resistance, especially in health care setting, is a growing, serious, and potentially preventable public health problem worldwide.

**Objectives:** We aimed to investigate the antimicrobial resistance pattern of Gram-negative bacteria (GNB) isolated from patients with bloodstream infections (BSI) at a tertiary care hospital in Iran.

**Methods:** We conducted a cross-sectional study of blood cultures submitted to the Professor Alborzi Clinical Microbiology Research Center at a 1000-bed university-affiliated hospital in Shiraz, southern Iran. All GNB isolated from August 2014 to September 2018 were included. Antimicrobial susceptibility testing was done by Kirby bauer’s disc diffusion method. Extended-spectrum β-lactamase (ESBL) producers were detected by double disc synergy test (DDST). The data entry and analysis were performed using the WHONET 5.6 software.

**Results:** Overall, among 4869 pathogens isolated from blood culture, GNB were the predominant isolates (3712, 76.2%). The most frequently isolated GNB were *Stenterophomonase maltophilia* (1318, 35.5%), followed by *Pseudomonase spp.* (466, 13%), *Escherichia coli* (n=397, 11%), *Acinetobacter baumannii* (317, 9%), and *Klebsiella pneumonia* (253, 7%). The number of Enterobacteriaceae were 947 (25.5%) and non-fermenting Gram-negative bacilli (NFGNB) 2741 (73.8%). Totally, 8.9% of Enterobacteriaceae isolates were carbapenem-resistant. In addition, 492 out of 716 Enterobacteriaceae isolates tested by DDST were ESBL producer (68.7%). On the other hand, 38.1% of NFGNB were carbapenem-resistant and 375 out of 396 NFGNB isolates tested by DDST were ESBL producer (94.7%). The resistance rates of Enterobacteriaceae to amikacin were 19.5%, gentamicin 30.5%, and ciprofloxacin 41.6%. The resistance rates of NFGNB to amikacin were 52.7%, gentamicin 54.5% and ciprofloxacin 35.3%.

**Conclusion:** GNB were the predominant bacteria isolated from patients with BSI. The high antibiotic resistance rate of these bacteria is a warning about a serious health problem. We need immediate actions including implementations and adhesions to infection control practices and antibiotic stewardship programs to overcome this serious health problem.

**Disclosure of Interest**: None declared

### P3 CHLORHEXIDINE BATHING TO PREVENT HEALTHCARE-ASSOCIATED BLOODSTREAM INFECTIONS IN PATIENTS WITH HAEMATOLOGICAL MALIGNANCIES: A PROSPECTIVE CONTROLLED COHORT STUDY

#### K.-L. Tien^1^, J.-T. Wang^1^, C.-T. Fang^2^, Y.-C. Chen^1^

##### ^1^Center for Infection Control, National Taiwan University Hospital; ^2^College of Public Health, National Taiwan University, Taipei, Taiwan

###### **Correspondence:** K.-L. Tien

**Background:** Patients with haematological malignancies hospitalised for myelosuppressive chemotherapy are at high risk of serious healthcare-associated infections. Chlorhexidine (CHG) bathing decreases incidence of bloodstream infections at intensive care units, but its effect has not been assessed in patients with haematological malignancies at non-critical-care units.

**Methods:** This is a prospective, concurrent controlled, cohort study at a university medical centre. Adults with haematological malignancies hospitalised for cytotoxic chemotherapy at non-critical-care units were offered daily 2% CHG bathing. We compared outcomes of patients chose to take CHG bathing (CHG group) and that of those chose not to take (usual care group). The primary outcome was gram-positive cocci, skin-flora-related, or central-line-associated bloodstream infections. The negative-control outcome was gut-origin baecteremia. Outcomes were monitored by a rule-based healthcare associated infections surveillance and classification system. Multivariable Cox regression analyses were used to adjust covariates. (registration no: #201508030RIPD)

**Findings:** The CHG group (*n*=485) had a crude incidence rate of primary outcome 60% lower than that in the usual care group (*n*=408) (3·4 vs. 8·4 per 1,000 patient-days, p< 0·001) but had a similar crude incidence of negative-control outcome (4·5 vs. 3·2 per 1,000 patient-days, p=0·297). In multivariable analyses, CHG bathing was associated with a 70% decrease in the primary outcome (adjusted hazard ratio [HR] 0·3, p<0·001). In contrast, CHG bathing had no effect on the negative-control outcome (adjusted HR=1·0, p=0·923). CHG bathing was well tolerated by participants in the CHG group.

**Interpretation:** CHG bathing is a highly effective approach to prevent gram-positive-cocci /skin-flora/central-line-associated baecteremia in patients with haematological malignancies hospitalised for cytotoxic chemotherapy at non-critical-care units.

**Disclosure of Interest**: None declared

### P4 THE USE CHLOREXIDINE GLUCONATE IMPREGNATED DRESSING AS PREVENTION CATHETER-RELATED INFECTION: INTEGRATIVE REVIEW

#### F. D. O. Andrade^1^, V. D. B. Poveda^2^, R. T. N. Turrini^2^, S. G. Esperandio^3^

##### ^1^Nursing, Hospital of Clinic of Federal University of Parana, Curitiba; ^2^Nursing, School Nursing of University of Sao Paulo, Sao Paulo; ^3^Nursing, Hospital Municipal of Sao Joao do Ivai, Sao Joao do Ivai, Brazil

###### **Correspondence:** F. D. O. Andrade

**Introduction:** Catheter-related infection are associated with increased rates of morbidity, mortality, increased length of hospital stay, and consequent increase in medical costs. It is known that one of the main sources of microbial colonization of central venous catheters is the microorganisms of the patient's own microbiota of the skin (endogenous microorganisms) located at the catheter insertion site.

**Objectives:** To compare and evaluate the efficacy of chlorhexidine gluconate impregnated transparent dressing compared to conventional dressing (with dry gauze) to reduce the count of skin microorganisms on the site of the catheter insertion, with consequent reduction of the catheter-related infection

**Methods:** This is an integrative review, performed through the databases: Virtual Health Library, Cranial, Cochrane, Embase, PubMed, using the keywords: antisepsis, chlorhexidine, catheter-related infection.

**Results:** 68 studies, observational or experimental, of which 2 were included, one (50%) meta-analysis and one (50%) randomized clinical trial, published between 2014 and 2019, in the English language, were produced in the United States and China. The studies analyzed demonstrated that there was a significant reduction of catheter-related infection in patients who used the chlorhexidine gluconate impregnated dressing compared to patients using the conventional dressing because it is believed that the insertion of the catheter to be exposed to continuous antiseptic action, and the easy visibility of the catheter insert.

**Conclusion:** The use of the chlorhexidine gluconate impregnated transparent dressing has been shown to be effective in reducing infection rates related to the central venous catheter. However, further studies should be conducted to evaluate the cost-effectiveness of chlorhexidine gluconate impregnated transparent dressing in order to provide safe, harmless care to patients who need to use the central venous catheter.

**Disclosure of Interest**: None declared

### P5 Withdrawn

### P6 INFECTIOUS COMPLICATIONS RELATED TO THE USE OF CENTRAL VENOUS ACCESS DEVICES AND PERIPHERALLY INSERTED CENTRAL CATHETERS: A COMPARATIVE STUDY

#### A. N. B. Mota^1^, V. de Brito Poveda^2^, R. N. T. Turrini^1^

##### ^1^Medical and surgical nursing, University of São Paulo (Universidade de São Paulo-USP); ^2^Medical and surgical nursing department, UNIVERSITY OF SÃO PAULO, São Paulo, Brazil

###### **Correspondence:** V. de Brito Poveda

**Introduction:** The use of Peripherally Inserted Central Catheters has shown benefits. However, the literature is controversial regarding its superiority for reducing bloodstream infection rates.

**Objectives:** The objective of the present study was to identify and compare the incidence of infectious complications related to the use of Central Venous Access Devices and Peripherally Inserted Central Catheters.

**Methods:** This prospective cohort study was carried out in intensive care units and medical and surgical clinics of a university hospital specialized in cardiopulmonology in the city of São Paulo, Brazil. The central venous catheters were evaluated on the day of insertion and monitored on a daily basis *in loco* throughout the hospitalization, up to their removal or hospital discharge (alive or deceased). The medical records of the patients were also reviewed in search of relevant information related to the catheters.

**Results:** 189 catheters were analyzed, and catheter-related bloodstream infection was confirmed in one (2.6%) patient with whom a Central Venous Access Device was used. Catheter-related bloodstream infection was be observed in 0.89/1000 catheter-days in Central Venous Access Devices and in 0/1000 catheter-days in Peripherally Inserted Central Catheters.

**Conclusion:** Overall, there was higher incidence of catheter-related bloodstream infection in patients using Central Venous Access Devices. 7However, the lack of more evidence to corroborate the greater efficacy of Peripherally Inserted Central Catheters compared to Central Venous Access Devices does not support indicating Peripherally Inserted Central

**Disclosure of Interest**: None declared

### P7 AUDIT ON GOOD PRACTICES RELATED TO PERIPHERAL VENOUS CATHETERS IN JANUARY 2019

#### G. Brahimi^1^, S. AIT SEDDIK^2^, H. KHELLAF^2^, N. CHEBOUB^1^, A. EL KECHAI^1^, A. CHETITAH^1^, K. CHABANE^1^, M. CHERCHARI^1^, A. DAHLI^1^, S. SLAOUTI^1^, I. BOUFASSA^1^, A. LARINOUNA^1^, A. REBOUH^3^, R. BELKAID^1^

##### ^1^ÉPIDÉMIOLOGIE; ^2^ ÉPIDÉMIOLOGIE, CHU BENI MESSOUS; ^3^ÉPIDÉMIOLOGIE, INSP, Alger , Algeria

###### **Correspondence:** G. Brahimi

**Introduction:** The practice of peripheral venous catheters (PVC) can be trivialized, and creates gaps from the guidelines.

**Objectives:** -Measure the application of good practices related to the installation, handling, duration and removal of PVCs.

- Identify the practices to be improved.

**Methods:** The audit was carried out in 14 medical departments from 30/12/2018 to 07/01/2019. The collection of data was done by direct observation at the time of the care and interview of the staff. The entry and analysis of the data was carried out on the EPIDATA software.

**Results:** 90 PVC were observed and followed until ablation. The pose was performed by public health nurses in 62.2% of cases. There is no published protocol on PVC's good practices in the audited services. A hand hygiene (HH) before the pose was observed in 6.7% of the cases and the wearing of gloves in 72.2% however their change was not realized in 64.4% neither by opportunity nor by patient. The first three skin preparation steps (debridement, rinsing and drying) weren't observed and the cutaneous antisepsis was carried out in 96.7% with alcohol at 70 ° and povidone iodine in 3.3%. Immediate removal of the mandrel in a nearby collector was observed in 52.2% of cases. There is no traceability of the catheter placement in the patients' file.The manipulation of PVC is preceded by an HH in 32.2% and a disinfection of the opening of the venous line by a sterile compress in 10% of cases. The fixation of the catheter was made using a non-sterile, non-transparent dressing. Surveillance of the perfusion line and insertion site was provided in 100% of cases. 18% of the withdrawals were preceded by a HH and 34.8% by a desinfection of the insertion point using a sterile pad. The duration of maintenance of the device was respected in 100% of the cases.

**Conclusion:** This audit allowed us to reflect on the need for training of the nursing staff. The installation of PVC must be the subject of a written protocol validated by CLIN.

**Disclosure of Interest**: None declared

### P8 NEW MUTATIONS IN GYRA AND PARC GENES IN CARBAPENEM- RESISTANT E. COLI AND A. BAUMANNII AMONG CATHETER RELATED BLOOD STREAM INFECTION PATIENTS IN EGYPTIAN ICUS

#### A. R. Elmanakhly, A. A. Elkholy

##### Infection prevention and control department, Dar Alfouad hospital, Cairo, Egypt

###### **Correspondence:** A. R. Elmanakhly

**Introduction:** Resistance of *E. coli* and *A. baumannii to antimicrobials* is a growing global concern. The most serious resistance bacteria that are carrying carbapenem-resistant genes and acquire changes in the structure of DNA gyrase and topoisomerase IV enzymes that lead to fluoroquinolone- resistance.

**Objectives:** evaluate the presence of mutations in the *gyrA* and *parC* genes in Egyptian ICU and their correlation with carbapenem resistant genes *E. coli* and *A. baumannii* isolates from patients in ICUs of a tertiary care hospital in Egypt.

**Methods:** A total of 300 consecutive and non-duplicate *A. baumannii* and *E. coli* clinical isolates were isolated from ICU patients in 4 tertiary hospitals in Egypt. The bacterial isolates were identified by VITEK-2 (Bio Merieux, France). Antimicrobial susceptability testeing was preformed according to CLSI guidelines. Phenotypic detection of carbapenemase was done by carba-NP test, followed by molecular identification of carbapenemase encoding genes *bla*_NDM_, *bla*_OXA-48_ and *bla*_KPC_ by multiplex PCR. The quinolone resistance-determining regions (QRDRs) of *gyrA* and *parC* genes were amplified by singleplex PCR followed by reverse and forward sequencing to detect the gene mutation. The DNA sequences were compared with the sequences of wild type of these genes available in GenBank database. Then, the obtained DNA sequences and their amino acid sequences were analysed using bioinformatics tools.

**Results:** *All isolates* showed high level of resistance among tested antimicrobial agents (cephalosporins, aminoglycosides, carbapenems, penicillins) that ranged from 36% to 100%. Carba-NP detected 43.59% of the carbapenem resistant isolates. Multiplex PCR detected that 17.95%, 46.15% and 2.56% of isolates were harbouring *bla*_KPC_, *bla*_NDM_ and *bla*_OXA-48_ respectively. PCR and sequencing technique showed combined gene mutation in 8 carbapenem resistant *E. coli* and *A. baumannii* isolates and specific new substitutions observed in gyrA and parC. On the other hand, were point mutations were observed in two A. baumannii isolates, whereas Ser172Leu mutation was observed in two *E. coli* isolates.

**Conclusion:** presence of carbapenem resistance genes in combination with single and multiple mutations in QRDR causes the presence of highly resistant *E. coli* and *A. baumannii* isolates in the Egyptian hospitals.

**Disclosure of Interest**: None declared

### P9 PRACTICES OF NURSES REGARDING HANDLING OF CENTRAL LINE CATHETER AT A TERTIARY CARE CENTER

#### N. V. Singh, N. V. Singh, S. Ghai, S. Ghai, R. Devi, S. Ghai, N. Vir Singh, GD Puri

##### NATIONAL INSTITUTE OF NURSING EDUCATION, POSTGRADUATE INSTITUTE OF MEDICAL EDUCATION AND RESEARCH, CHANDIGARH, CHANDIGARH, India

###### **Correspondence:** N. V. Singh

**Introduction:** Central venous catheters(CVC)are used to administer the life supportive medications. These catheters place the patients at risk of complications including blood stream infection. Nurses have an important role in the prevention of such infections. There is a need to develop standard guidelines for these types of procedures.

**Objectives:** To observe baseline practices of nursing personnel regarding care of central line catheter in ICUs of a tertiary care centre.

**Methods:** An Observation checklist was developed to assess the baseline practices of nursing personnel. The practices were observed regarding hand washing, maximal barrier precautions, skin asepsis, frequency of central line care and dressing change, replacement of IV fluid administration sets. Focus group discussions were conducted to identify the problems encountered by them during the care of CVC and seeking their suggestions.

**Results:** Hand washing was performed by 91.7% of the nurses before accessing the insertion site. 66.7% performed hand hygiene before donning gloves to handle CVC and 75% performed hand hygiene after removing the gloves. Extension lines and high pressure lines were changed after 72 hrs by 91.6% of the nurses. Everyone secured the CVC line well to the patients. None of the nurses wiped the catheter lumen after use, before and after sampling and before connecting new drug. 91.6% wore clean gloves to remove the old dressing, stabilized the catheter hub while removing the old dressing and performed proper cleaning of CVC line. Only 41.6% documented the date and time of dressing change in nurses’ record.

**Conclusion:** Total 6 focus group discussions were conducted. The problems verbalized by the nursing personnel that hindered the proper care of the central venous catheter line were the none availability of a written protocol on CVC line care, Inadequate nurse patient ratio and supply of articles, absence of inservice education programme and lack of coordination between the members of the health care team

**Disclosure of Interest**: None declared

### P10 CONTROLLING HEALTHCARE ASSOCIATED PRIMARY BLOODSTREAM INFECTION IN NEONATAL INTENSIVE CARE UNIT

#### W. A. Mazi^1^, N. A. Bouafia^1^, T. J. Kalarikkal^2^, S. H. Al Wagdani^1^, R. I. Abutaha^2^

##### ^1^Infection Prevention and Control; ^2^Neonatal Intensive Care Unit, King Faisal Medical Complex, Taif, Saudi Arabia

###### **Correspondence:** W. A. Mazi

**Introduction:** Health care-associated blood stream infections (HABSIs) are cause of morbidity and mortality in very low birth weight neonates.

**Objectives:** To reduce HABSI.

**Methods:** A prospective study was conducted in a 92-bed capacity level II/III NICU category at King Faisal Medical Complex, Taif, Saudi Arabia, from January 2018 to April 2019. Criteria of HABSI was carried out using the Centers for Disease Control and Prevention and National Healthcare Safety Network (CDC/NHSN, USA) guidelines. Incidence rate, utilization ratio, benchmarking, and statistical analysis were carried out using the NHSN recommendations.

NICU was segregated into 5 zones based on patient clinical condition; NICU zone1 for admission till improved, NICU zone 2 for respiratory support without ventilator, NICU zone 3 for high dependency care, gaining weight with minimal respiratory support without nutrition support, NICU zone 4 for special care baby, weighted more than 1500 gram and preparing for discharge home and NICU zone 5 for chronic patients and stay until discharge home. Each zone was assigned nurses based on patient to nurse ratio as 1:4, 1:5, 1:6 and 1:7 and 1:8; respectively. Hand hygiene compliance was emphasized with continuous monitoring hand hygiene using camera observation located in the unit with regular feedback. Central-line bundle prevention measures were implemented.

**Results:** Hand hygiene and central-line bundle compliance rates were sustained in high level (ranged from 78% to 89% and 90%, respectively).

The incidence rate per 1000-central line days among each birth-weight category exceeded 90th percentile before intervention and declined to 75th percentile after intervention with different utilization ratio benchmarking to NHSN, USA.

Two outbreak episodes were reported caused by extended spectrum beta lactamase (ESBL) *Klebsiella pneumonia* and *Escherichia coli*. After intervention in August 2018, no case of ESBL *E. coli* with only one case of ESBL *K. pneumonia* were reported since January to April 2019. There is significant reduction in incidence of primary bloodstream infection (2.14- *vs* 0.48/100 admission) (*p-value* 0.0001).

**Conclusion:** Segregation patients with maintain in patient to nurse ration and compliance to hand hygiene assumed significant role in managing and controlling of primary bloodstream infection in NICU.

**References**

Not applicable.

**Disclosure of Interest**: None declared

### P11 APPLICATION OF SOCIETY HEALTHCARE EPIDEMIOLOGY OF AMERICA/INFECTIOUS DISEASES SOCIETY OF AMERICA BASIC RECOMMENDATIONS TO REDUCE CENTRAL LINE-ASSOCIATED BLOODSTREAM INFECTIONS IN INTENSIVE CARE UNIT

#### W. A. Mazi^1^, M. H. Abdulwahab^1^, M. M. Al Ashqar^1^, Y. S. Aldecoa^1^, Z. R. Bahat^1^, O. S. Yasin^2^

##### ^1^Infection Prevention and Control; ^2^Intensive Care Unit, King Faisal Medical Complex, Taif, Saudi Arabia

###### **Correspondence:** W. A. Mazi

**Introduction:** Healthcare associated infections (HAIs) increase mortality, length of hospital stay, cost of care, bacterial resistance, antibiotic usage and other adverse events. Central line-associated bloodstream infection (CLABSI) remains an important cause of morbidity and mortality in intensive care units (ICUs), particularly in developing countries.

**Objectives:** To reduce CLABSI rate targeting the National Healthcare Safety Network, USA (NHSN) Benchmark.

**Methods:** A prospective intervention was conducted in the 27-bed medical/surgical intensive care unit of King Faisal Medical Complex-Taif, Kingdom of Saudi Arabia from January to December 2018. The basic Society Healthcare Epidemiology of America/Infectious diseases Society of America (SHEA/IDSA) practice recommendations to reduce CLABSI were introduced and implemented during the year 2018. CLABSI was identified using the Centers for Disease Control and Prevention and NHSN criteria. Incidence rate, ratio, benchmarking, and statistical analysis were carried out using the NHSN recommendations. Bacterial identification and antimicrobial susceptibility of isolates were determined according to Clinical Laboratory Standards Institute guidelines, 2016. External validation surveillance was conducted to ensure no any missed CLABSI case.

**Results:** The number of reported CLABSI cases was 4 cases in 2018. The incidence rate was 0.67/1000 central line-days with utilization ratio 0.51 indicated achievement to the NHSN benchmark as planned (standardized infection ratio 1).

*Klebsiella pneumonia* extended spectrum beta lactamase (ESBL) was the most common microorganisms causative agent. No outbreak was observed during the study period.

**Conclusion:** CLABSI incidence rate and ratio in medical-surgical ICU in King Faisal Medical Complex is within the NHSN benchmark, 2018.

**Disclosure of Interest**: None declared

### P12 HEALTHCARE-ASSOCIATED BLOODSTREAM INFECTION RATE REDUCTION IN THE NEURO-ICU IN RUSSIA

#### K. Ershova^1^, I. Savin^2^, O. Ershova^3^, G. Danilov^2^, N. Kurdumova^2^, M. Shifrin^4^, I. Alexandrova^5^

##### ^1^Department of Anesthesiology, Keck School of Medicine, University of Southern California, Los Angeles, United States; ^2^Department of Critical Care; ^3^Department of Infection Control; ^4^Department of Statistics; ^5^Department of Microbiology, Burdenko Neurosurgery Institute, Moscow, Russian Federation

###### **Correspondence:** O. Ershova

**Introduction:** According to the CDC, the rate of healthcare-associated bloodstream infections (HABSI) can be decreased by infection control programs [1].

**Objectives:** The goal of the study was to evaluate the effect of the infection control program on the rate and etiologic profile of HABSI in the Russian neuro-ICU.

**Methods:** This prospective study included high-risk patients (LOS in the ICU >2 days). Data was collected as a part of the infection control program and surveillance. We defined HABSI cases according to the 2008 CDC definition. We conducted microbiologic assay of all central line catheters removed from patients in the ICU, and blood samples when HABSI was suspected.

**Results:** A total of 3178 patients was included in the study from 2011 to 2018. We observed 156 HABSI including 131 CLABSI. The incidence of HABSI declined from 7.0% in 2011 to 2.7% in 2018, p-value=0.041. The incidence of CLABSI did not change, averaging at 3.96 per 1000 catheter-days with a maximum of 4.9 in 2014 and a minimum of 1.9 in 2013. The average mortality rate was 19.9 per 100 patients with HABSI which is higher than an overall mortality rate in the ICU (15%).

On average we tested 320 central line catheters annually. The bacterial growth was detected in 39 per 100 tested catheters in 2011 and in 21 per 100 in 2018, that constitutes the significant decrease, p-value=0.03. Among the most frequent pathogens identified at the catheters, there were 28% of coagulase-negative *Staphylococcus spp.*, 14% of *Acinetobacter baumannii*, and 11% of *Klebsiella pneumoniae*.

The etiologic spectrum of CLABSI remained stable over time and on average contained 26% of coagulase-negative *Staphylococcus* spp., 17.8% of *Klebsiella pneumoniae*, 10.5% of *S. aureus*, and 6.2% of *Candida spp*.

**Conclusion:** We found that the infection control and surveillance program is associated with a declining incidence of HABSI in the ICU and with a declining rate of bacterial contamination of central catheters. The most frequent pathogens identified on catheters and in blood samples was coagulase-negative *Staphylococcus* spp.

**References**

1. https://www.cdc.gov/hai/pdfs/progress-report/2017-Progress-Report-Executive-Summary-H.pdf.

**Disclosure of Interest**: None declared

### P12b BLOODSTREAM INFECTION ANTIBIOGRAM IN SYRIAN FEBRILE NEUTROPENIC PATIENTS

#### A. Alrstom* ^1^, N. Daher^1^, R. Abouharb^1^

##### ^1^Internal Medicine, DAMASCUS UNIVERSITY, DAMASCUS, Syrian Arab Republic

###### **Correspondence:** A. Alrstom

**Introduction:** The local antibiogram is so important to guide antimicrobial therapy especially in febrile neutropenic patients. Hence early appropriate antimicrobial therapy improves patients’ outcome.

**Objectives:** This research study aimed to record the microbiological profile of organisms which cause bloodstream infections in febrile neutropenic patients.

**Methods:** Prospective cohort study included all patients with febrile neutropenia (FN) with haematological malignancies who admitted to Al-Mouwasat university hospital (a tertiary care centre) over a period of 18 months. The characteristics of patients were recorded and thorough physical examinations were performed then routine blood culture samples were drawn before antibiotics administration and standard bacterial culture protocol was applied to identify isolates and antimicrobial susceptibility profile.

**Results:** In our study, 123 febrile neutropenia episodes developing in 109 patients. The most underlying malignancy was acute myeloid leukaemia. Fever of unknown origin was the most predominant clinical features (32.8%). Out of the 123 episodes of neutropenic fever, 42 (34.1%) of blood culture specimens yielded positive growth. Gram-negative bacilli (GNB) were the most common pathogen (n=37; 88%) with a high rate of extended-spectrum beta-lactamase (ESBL) suggestive pattern at antibiotic susceptibility tests (n=18; 48.6%). Carbapenem-resistant was demonstrated in 8.1% of all cultivated isolates; suggestive carbapenemresistant Enterobacteriaceae (CRE) pattern. The presence of past neutropenic fever episode within the previous 3 months was the most risk factor associated with ESBL and CRE positive pathogens acquisition.

**Conclusion:** Gram-negative bacilli are the predominant pathogens in Syrian febrile neutropenic patients and most are still resistant to all first-line antibiotics. Empirical antibiotic therapy for neutropenic patients should be tailored according to local antibiograms.

Infection control and infectious diseases practitioners may need to apply tougher infection control measures to prevent nosocomial infections in this population.

**Disclosure of Interest:** None Declared

## Poster Session: Surgical site infection: Intervention to reduce the burden 1

### P13 SUCCESSFUL POST-PROCEDURE INTERVENTION IN A TERTIARY CARE SETTING WITH HIGH RATE OF SURGICAL SITE INFECTION AMONG PATIENTS WITH CORONARY ARTERY BYPASS GRAFT

#### O. Slim^1^, M. Alshamrani^1^, B. Abukhzam^1^, S. Abdulebdeh^2^, D. Abagguey^1^, A. El-Saed^1^, H. Balkhy^3^

##### ^1^Infection Prevention & Control; ^2^cardiac department, King Abulaziz Medical City, Riyadh, Saudi Arabia; ^3^Antimicrobial Resistance, World Health Organization , Genva, Switzerland

###### **Correspondence:** O. Slim

**Introduction: The rate of surgical site infection (SSI) among our patients who underwent coronary artery bypass graft (CABG) has been consistently higher than reported by the US National Healthcare Safety Network (NHSN). Post-discharge personal hygiene has been raised as a possible contributing factor.**

**Objectives: The objective was to examine the impact of using post-procedure antiseptic body shower on infection and mortality in CABG patients.**

**Methods:** Interventional study was conducted among all patients who underwent CABG at king Abulaziz Medical City, Riyadh, Saudi Arabia between October 2018 and March 2019. The intervention was educational sessions focusing on appropriate usage of chlorhexidine gluconate 4% antiseptic body shower during hospital stay and 7 days post-discharge. The outcome was the development of superficial SSI according to NHSN criteria. This was assessed from outpatient records, emergency visits, and a phone call. Additionally, the phone call was used to confirm the compliance with the intervention.

**Results:** Out of 111 patients included in the current study, 87 (78.4%) were compliant with the post-procedure antiseptic body shower and 13 (11.7%) developed superficial SSI. Compared with non-compliant patients, patients who were compliant with the intervention had markedly low SSI rate (2.3% vs. 45.8%, p<0.001) and no mortality (0.0% vs. 8.3%, p=0.045). The difference in SSI remained significant after adjustment for the risk index categories in both groups (p<0.001). Additionally, total and post-procedure length of hospital stay were shorter among compliant patients compared with non-compliant patients (17.8±8.4 vs.31.1±30.3 days, p<0.001 and 11.5±6.6 vs. 24.1±29.2 days, p<0.001, respectively).

**Conclusion:** The current findings indicate that the use of chlorhexidine gluconate 4% antiseptic body shower was very effective in reducing the risk of infection, mortality, and length of stay. Additionally, the findings highlight the importance of patient education and personal hygiene. The findings still need to be confirmed in large randomized studies.

**Disclosure of Interest**: None declared

### P14 THE IMPACT OF ENDOSCOPIC VEIN HARVESTING TECHNIQUE ON SECONDARY SURGICAL SITE INFECTION AMONG PATIENTS UNDERGOING CORONARY ARTERY BYPASS GRAFT

#### M. Alshamrani^1^, A. Arifi^2^, O. Slim^1^, B. Abukhzam^1^, H. Eid^1^, A. El-Saed^1^, H. Balkhy^3^

##### ^1^Infection Control; ^2^cardiac department, King Abulaziz Medical City, Riyadh, Saudi Arabia; ^3^Antimicrobial resistance, WHO, Geneva, Switzerland

###### **Correspondence:** M. Alshamrani

**Introduction:** The rate of surgical site infection (SSI) at the donor site of patients undergoing coronary artery bypass graft (CABG) at our hospital used to be threefold higher than reported by the US National Healthcare Safety Network (NHSN). In an effort to reduce the SSI, saphenous vein harvesting using endoscope was implemented in June 2016.

**Objectives:** The objective was to compare the impact of using endoscopic vein harvesting (EVH) versus traditional open vein harvesting (OVH) on secondary SSI among CABG patients.

**Methods:** Prospective surveillance was done among patients who underwent CABG at king Abulaziz Medical City (KAMC), Riyadh, Saudi Arabia between June 2016 and March 2019. The surveillance methodology and secondary SSI definition was similar to NHSN ones. Post-discharge surveillance included surgical follow-up, outpatient clinic visits, and emergency visits. Endoscopic vein harvesting was done only by one surgeon who had the clinical skills for the technique.

**Results:** A total 474 patients were included in the current analysis. The average age was 60.9±10.1 years and 70.0% were males. Out of 474 patients, endoscopic vein harvesting was done among 275 (58.0%) patients and secondary SSI was detected in 11 (2.3%) patients. Compared with OVH, EVH was associated with lower secondary SSI rate, which was marginally significant (1.1% vs. 4.0%, p=0.059). Compared with NHSN, standardized infection ratios (SIRs) adjusted for differences in risk index categories between KAMC and NHSN were similar in KAMC patients with EVH (SIR=1.23, 95% CI=0.25-3.58, p=0.737) but much higher in KAMC patients with OVH (SIR=4.07, 95% CI=1.75-8.02, p=0.004).

**Conclusion:** The current findings indicate that endoscopic vein harvesting was effective in reducing the risk of SSI at the donor site among our cohort of patients. Implementing the endoscopic vein harvesting technique to all CABG patients may further reduce the secondary SSI rate.

**Disclosure of Interest**: None declared

### P15 SWISSNOSO SSI INTERVENTION MODULE: RESULTS FROM THE PILOT STUDY, APRIL 2017-MARCH 2019

#### A. C. Szelecsenyi^1^, A. S. Schweiger^1,2^, M. Schlegel^3^, N. Troillet^4,5^, S. P. Kuster^6^, D. Vuichard Gysin^1,2^, R. Sommerstein^7^, A. F. Widmer^2^, on behalf of Swissnoso

##### ^1^Swissnoso, National Center for Infection Control, Bern; ^2^Infectious Diseases & Hospital Epidemiology, University Hospital Basel, Basel; ^3^Division of Infectious Diseases and Hospital Epidemiology, Kantonsspital St. Gallen, St. Gallen; ^4^Service of Infectious Diseases, Central Institute of the Valais Hospitals, Sion; ^5^Service of Preventive Medicine, University Hospital of Lausanne, Lausanne; ^6^Division of Infectious Diseases and Hospital Epidemiology, University and University Hospital Zurich, Zürich; ^7^Department of Infectious Diseases, Bern University Hospital, University of Bern, Bern, Switzerland

###### **Correspondence:** A. C. Szelecsenyi

**Introduction:** Surgical site infections (SSI) are the most common health-care associated infections. A large fraction of these can be prevented.

**Objectives:** In parallel with the existing Swissnoso SSI surveillance, a pilot study was set up in 10 acute-care hospitals, aiming at implementing a nationwide SSI intervention module measuring compliance with SSI prevention process parameters.

**Methods:** From April 2017 to March 2019, every participating hospital set up an interdisciplinary working group and collected data on its compliance with the three major elements of preoperative management: hair removal, skin disinfection and perioperative antimicrobial prophylaxis. Each hospital had to monitor a minimum of 10 operations per quarter. Compliance with the individual elements and the overall compliance were determined according to the process parameters observed. Poisson regression was used to determine increase with compliance throughout the pilot period.

**Results:** A total of 590 observations were performed. The overall compliance measured in 8 hospitals increased from 55% (95% CI, 45-67) in Q2 2017 to 78% (95% CI, 65-92) in Q1 2019 (p=0.029). The relative increase per quarter was 5.2% (95% CI, 0.5-10%, p=0.029). The lowest compliance rate was observed for perioperative antimicrobial prophylaxis in Q3 2017 with 68% (95% CI, 56-80). Errors were particularly due to incorrect redosing practice. Best compliance was achieved with hair removal in Q1 2018 with 100%.

**Conclusion:** The introduction of the bundle for prevention of SSI led to a continuous and significant increase of the overall compliance. This module will thus be proposed to all Swiss acute care hospitals that already participate in the Swissnoso SSI surveillance aiming at reducing SSI rates on a national level.

**Disclosure of Interest**: None declared

### P16 RESULTS OF AN INSTITUTIONAL BUNDLE TO REDUCE THE SURGICAL SITE INFECTION (SSI) IN COLO-RECTAL SURGERY

#### V. Pomar^1^, M. A. Cotura^1^, G. Azparren^2^, J. Bollo^3^, J. A. Fernández^2^, G. Horta^4^, M. V. Moral^2^, P. Pascual^1^, M. P. Pallares^4^, S. Piñol^2^, L. Ramírez^3^, N. Roch^3^, E. Targarona^3^, N. Benito^1^, M. Gurgui^1^, A. Moral^3^, J. Lopez-Contreras^1^

##### ^1^Infectious Diseases Unit; ^2^Anesthesiology; ^3^Surgery; ^4^Operating room, Hospital de la Santa Creu i Sant Pau, Barcelona, Spain

###### **Correspondence:** J. Lopez-Contreras

**Introduction:** Multidisciplinary teams allow to join forces and improve the healthcare outcomes, it is important the implementation of the institucional bundles to reduce incidence of surgical site infection (SSI)

**Objectives:** To determine the colo-rectal surgical site infection (SSI) incidence after the implementation of a specific bundle.

**Methods:** Colo-rectal SSI was 25% and 26% in 2013 and 2014 in our Hospital. In 2014 a multidisciplinary team was created to improve results which included General Surgeons, Anaesthesiologist, nurses from the operating room (OR) and surgical ward and infection control practitioners.

The team measured the following measures: pre-surgical body hygiene, hair removal, perioperative normothermia, normoglycemia, antibiotic prophylaxis, laparoscopic approach and aseptic measures in the OR. Moreover the measures below have been implemented:

During this period a nurse has been hired to manage the cases. The team maintaines quarterly sessions to follow up the evolution of the different indicators and the incidence of SSI.

**Results:** The SSI incidence, adequate antibiotic prophylaxis and normothermia are summarized in the table:

**Table:** 

**SSI Incidence**	2012 N=141	2013 N=183	2014 N=162	2015 N=241	2016 N=263	2017 N=255	
Global n (%)	35 (25)	48 (26)	34 (21)	44 (18)	34 (13)	30 (12)	P=0.02
Organ-space n(%)	17 (12)	33 (18)	22 (14)	35 (14)	22 (8)	22 (9)	P=0.64
**Measures**	2012 N=141	2013 N=183	2014 N=162	2015 N=241	2016 N=263	2017 N=255	
Adequate AB prophylaxis (%)	100 (71)	101 (55)	138 (85)	206 (86)	222 (84)	217 (85)	P=0.04
Normothermia n(%)	57 (40)	66 (36)	34 (49)	104 (58)	125 (64)	175 (69)	P<0.001

**Conclusion:** The creation of this multidisciplinary team has been associated with an improvement of the process measures compliance and also with an important reduction of colo-rectal SSI.

**Disclosure of Interest**: None declared

### P17 REDUCING THE INCIDENCE OF SURGICAL SITE INFECTION POST CESAREAN SECTION AFTER IMPLEMENTING AN IMPROVEMENT PROJECT AT TERTIARY HEALTHCARE CENTER

#### N. A. Bouafia^1^, W. A. Mazi^1^, W. A. Sayed^2^, S. H. Alwagdani^1^, A. A. AlTalhi^3^, M. H. AlZahrani^4^, A. A. AlAmri^4^, A. M. Dahlawi^5^, M. R. ALYAMI^6^

##### ^1^INFECTION PREVENTION AND CONTROL, KING FAISAL MEDICAL COMPLEX; ^2^OBSTETRIC AND GYNECOLOGY DEPARTMENT, MATERNITY TOWER -KING FAISAL MEDICAL COMPLEX; ^3^HEAD OF OPERATING ROOM and MEDICAL DIRECTOR, KING FAISAL MEDICAL COMPLEX; ^4^OPERATING ROOM; ^5^MEDICAL DIRECTOR, MATERNITY TOWER -KING FAISAL MEDICAL COMPLEX; ^6^HOSPITAL DIRECTOR, KING FAISAL MEDICAL COMPLEX, TAIF, Saudi Arabia

###### **Correspondence:** N. A. Bouafia

**Introduction:** Surgical site infections (SSI) constitute a significant problem in surgical procedures, particularly with caesarean sections (CS).

**Objectives:** To reduce the CS-SSI rate by improving compliance with the guideline of Society of Healthcare Epidemiology of America (SHEA).

**Methods:** A quality improvement project was implemented based on prospective surveillance data of post-CS SSI conducted for all women undergone caesarean delivery at King Faisal Medical Complex-Taif, Saudi Arabia between January and April 2018 (pre-interventional period). This was followed by intervention and follow up period between May and October 2018. CS-SSI was identified using the Centers for Disease Control and Prevention and National Healthcare Safety Network (NHSN, USA) criteria based on ongoing and re-admission surveillance methods. Risk factors for CS-SSI was assessed according to SHEA recommendations and appropriate interventions were implemented according to findings.

**Results:** During the pre-interventional phase, CS-SSI incidence increased from 0.67% to 2.21%. Almost half of SSI occurred during the first week following the CS (52%). There was no statistical significant difference between the women undergone CS during the pre- and post-interventional phase regarding specific risk factors. Analysis of risk practices showed that seven among eighteen recommendations of SHEA were not applied. Principle interventions were: revision of surgical prophylaxis policy and procedures, training of new staff, appropriate skin preparation, better compliance to SSI bundle, follow-up patient within a week of hospital discharge and patient’s education on wound care. After intervention, 94% of SHEA recommendations were applied and SSI incidence rate decreased to reach 0.39% in October 2018.

**Conclusion:** Our quality Improvement project to reduce SSI incidence rate achieved its target and is sustained by continuous preventive actions.

**Disclosure of Interest**: None declared

### P18 PROMOTING SAFE SURGERY IN CORONARY ARTERY BYPASS (CABG) IN A TERTIARY HOSPITAL IN MALAYSIA

#### S. S. Samsudin^1,2^, S. S. L. Goh^1^, S. Saaibon^1,2^, R. Zhazali^1,2^, R. Ramli^2^, N. Hashim^3^, M. F. Zainal Abidin^3^, A. Amin^4^, M. Kumaran^4^, I. Azmi^4^, S. Krishnasamy^4^, Z. Zulkifli^4^, S. Hashim^4^, A. Mokhtar^4^, S. Ponnampalavanar^1,5^

##### ^1^Infection Control; ^2^Nursing; ^3^Anesthesia; ^4^Surgery, University Malaya Medical Centre; ^5^Medicine, University Malaya, Kuala Lumpur, Malaysia

###### **Correspondence:** S. S. Samsudin

**Introduction:** Surgical site infections (SSIs) following coronary artery bypass graft (CABG) procedures is a global public health problem.

**Objectives:** To determine the incidence of SSIs and the impact of implementation of perioperative preventive measures in patient who underwent CABG in University Malaya Medical Centre (UMMC).

**Methods:** Patients who underwent CABG in UMMC from January 2017 to December 2018 were prospectively followed up by the infection control nurse for 90 days post operation using electronic medical records (EMR). The study periods were divided into pre-intervention (January-May 2017), implementation of intervention (June-December 2017) and post intervention (January-December 2018). Data was collected using standardized SSI surveillance form and analyzed using SPSS version 20.

**Results:** A total of 260 patients were included (pre-intervention, intervention and post intervention period were 53, 76, 131 patients respectively). The incidence of SSI reduced from 21/100 procedures to 14/100 procedures during the pre and post-intervention periods respectively. The rate of sternal infections (SI) was 8% and 9% in the pre and post intervention periods respectively. Venous graft site (VGS) infection reduced from 13% to 7.6%. The interventions implemented were pre-operative bathing, clipping instead of shaving, using 2% chlorhexidine gluconate in 70% alcohol solution for surgical skin preparation, appropriate antibiotic prophylaxis and intraoperative redosing.

**Conclusion:** The SSI rates post CABG, especially VGS infections reduced after implementation of evidence based interventions. These interventions should be implemented as a standard of care for all surgical procedures in UMMC. The reason sternal SSI did not improve needs further investigation.

**Disclosure of Interest**: None declared

### P19 REDUCTION IN THE SURGICAL SITE INFECTION (SSI) RATE FOLLOWING SUSTAINED IMPROVEMENT IN PROCESS MEASURES

#### N. Grae, A. Morris, S. Roberts

##### Infection Prevention & Control Programme, New Zealand Health Quality & Safety Commission, Wellington, New Zealand

###### **Correspondence:** N. Grae

**Introduction:** The Health Quality & Safety Commission's national Surgical Site Infection Improvement (SSII) programme started in 2012 with hip and knee arthroplasty data reported from July 2013 and cardiac procedures from July 2016. The programme aims to improve the standard of care by measuring compliance with interventions known to reduce the risk of surgical site infections (SSI) and to use high quality data to inform quality improvement initiatives that reduce harm.

**Objectives:** The programme aims to improve standard of care to reduce the risk of SSI in orthopaedic and cardiac surgery.

**Methods:** Performance with process measures (surgical antimicrobial prophylaxis (SAP) and skin antisepsis) collected on all publicly-funded hip and knee arthroplasty and cardiac procedures; approximately 12,500 procedures annually. Quality and Safety Markers (QSMs) were established to set the expected levels of performance. The outcome measure is the SSI rate. Public reporting is quarterly through an online dashboard.

**Results:** Between July 2013 and December 2018; over 56,000 hip and knee arthroplasty procedures were performed. A significant aggregated improvement in the QSM performance for the timing, choice and dose of SAP and a significant improvement in the outcome measure; orthopaedic SSI rate mean decreasing from 1.23% (*SD = 0.34*) to 0.92% (*SD = 0.39*) (*p-value = 0.0008*).

Between July 2016 and December 2018, over 6800 adult and paediatric cardiac procedures were performed. The aggregated QSM compliance for antibiotic timing, choice and dose and skin prep was high at the start and has been sustained. The cardiac SSI rate mean is 4.4% (*SD = 1.27*).

**Conclusion:** The process measures for most orthopaedic procedures achieve the QSM targets. Concurrent with this is the improved outcome measure, reduced SSI rate, for orthopaedic procedures.

Five District Health Board hospitals perform publicly-funded cardiac surgery. Compliance with the process measures was high from the start of the programme suggesting that spread of best practice beyond the orthopaedic operating rooms may have occurred in these hospitals.

There has been a high level of engagement with the programme by the respected surgical services supported by timely feedback of process and outcome measures.

**Disclosure of Interest**: None declared

### P20 PROMOTING COMPLIANCE WITH SURGICAL SITE INFECTION PREVENTION GUIDELINES BY PSYCHOLOGICALLY TAILORED INTERVENTIONS: STUDY PROTOCOL OF THE MULTI-CENTER PARALLEL-GROUP CLUSTER-RANDOMIZED CONTROLLED "WACH"-TRIAL

#### T. von Lengerke^1^, B. Schock^2^, I. Hartlep^2^, P. Schipper^2^, I. Tomsic^1^, C. Krauth^3^, I. F. Chaberny^2^

##### ^1^Department of Medical Psychology, Hannover Medical School, Centre of Public Health and Healthcare, Hannover; ^2^Institute of Hygiene, Hospital Hygiene and Environmental Medicine, Leipzig University Hospital, Leipzig; ^3^ Institute of Epidemiology, Social Medicine and Health Systems Research, Hannover Medical School, Centre of Public Health and Healthcare, Hannover, Germany

###### **Correspondence:** T. von Lengerke

**Introduction:** Surgical site infections (SSI) are among the most prevalent nosocomial infections in Germany [1]. Despite national recommendations [2], evidence both on compliance with pre-, intra- and postoperative measures and interventions to promote compliance is lacking.

**Objectives:** To present the rationale and protocol of the WACH-trial ("Wundinfektionen und Antibiotikaverbrauch in der Chirurgie"), funded by the German Federal Ministry of Health (grant-ID: ANNIE2016-55-038; DRKS-ID: DRKS00015502) and approved by the Ethics Committee at the Faculty of Medicine of Leipzig University on June 12th, 2018 (034/18ek), based on the protocol co-developed with the Clinical Trial Centre Leipzig.

**Methods:** Study protocol.

**Results:** WACH is multi-center parallel-group cluster-randomized controlled trial. It expands the PSYGIENE-trial's approach of psychological tailoring [3-4; DRKS00010960] from hygienic hand disinfection in intensive care at one tertiary university hospital to SSI-prevention in six general hospitals. Target groups are physicians and nurses in surgical/anesthesiological wards/operating theatres. First, compliance and its determinants (COM-B-model [5]) are empirically assessed. Second, tailored interventions will both be developed in and suggested to three of the hospitals. Third, the hypothesis will be tested whether tailored interventions lead to stronger compliance improvements and SSI-reductions than usual implementation interventions.

**Conclusion:** First multi-center data on compliance with pre-, intra- and postoperative SSI-preventive measures and its promotion in German healthcare is expected.

**References**

[1] Behnke et al. The prevalence of nosocomial infection and antibiotic use in German hospitals. Dtsch Arztebl Int 2017;114:851-7

[2] KRINKO. [Prevention of surgical site infections]. Bundesgesundheitsbl 2018; 61:448-73

[3] von Lengerke et al. Promoting hand hygiene compliance. Dtsch Arztebl Int 2017;114:29-36

[4] von Lengerke et al. Impact of psychologically tailored hand hygiene interventions on nosocomial infections with multidrug-resistant organisms. Antimicrob Resist Infect Control 2019;8:56

[5] Michie et al. The behaviour change wheel. Implement Sci 2011;6:42

**Disclosure of Interest**: None declared

### P21 TRICLOSAN-COATED SUTURES REDUCE THE RISK OF SURGICAL SITE INFECTIONS: A SYSTEMATIC REVIEW AND META-ANALYSIS

#### T. Mulder^1^, M. Abbas^2^, S. Harbarth^2^, J. Kluytmans^1^

##### ^1^Julius Center for Health Sciences and Primary Care, UMC Utrecht, Utrecht, Netherlands; ^2^Infection Control Programme, Geneva University Hospitals and Faculty of Medicine, Geneva, Switzerland

###### **Correspondence:** T. Mulder

**Introduction:** The effect of triclosan-coated sutures (TCS) on surgical site infection (SSI) risk has been extensively studied, yet several studies have been published recently.

**Objectives:** The aim of this study was to systematically review and update the available evidence regarding the effectiveness of TCS to reduce SSI.

**Methods:** PubMed, MEDLINE, Embase, Scopus and Cochrane library were searched for studies that compared TCS with non-coated sutures (NCS) for SSI prevention. Observational studies could be included if they met the EPOC quality criteria. A pooled risk ratio (RR) with 95% confidence interval (CI) was calculated and a random effects model was used to account for potential clinical heterogeneity. The primary outcome was SSI of any type, and the secondary outcome was deep incisional and organ/space SSI.

**Results:** 27 studies with a total of 12,850 patients were included in the meta-analysis. These were all randomized clinical trials (RCTs) as none of the observational studies met the predefined quality standard. The risk of SSI was 7.0% (TCS group) versus 9.2% (NCS group). Pooling all studies demonstrated a risk reduction of 27% with TCS compared to NCS (RR 0.73 [95% CI 0.62 - 0.85]) corresponding to a number needed to treat (NNT) of 45 patients to prevent 1 SSI. Statistical heterogeneity (*I*^2^) was 34%. Evaluation of the effectiveness of TCS on the development of deep and organ/space SSI was based on 13 studies (n = 8,584). The risk of deep and organ/space SSI reduced from 2.5% (NCS) to 1.7% (TCS) (RR 0.75 [95% CI 0.56-1.02]; NNT 125).

**Conclusion:** This meta-analysis of 27 RCTs shows that TCS are associated with a significant reduction of SSI risk. Subgroup analysis shows a statistically non-significant association between TCS and risk of deep and organ/space SSI. Data on adverse events related to TCS, such as development of biocide resistance remain scarce.

**Disclosure of Interest**: None declared

## Poster Session: Surgical site infection: Risk factors

### P22 RISK FACTORS FOR 30 - DAY SURGICAL SITE INFECTIONS AFTER TOTAL HIP AND KNEE ARTHROPLASTY - A SINGLE-CENTER EXPERIENCE

#### V. Marusic^1^, L. Markovic-Denic^1^, V. Nikolic^1^, O. Djuric^1,2,3^, E. Dubljanin-Raspopovic^4^, M. Kadija^5^

##### ^1^Institute of Epidemiology, Faculty of Medicine, University of Belgrade, Belgrade, Serbia; ^2^Section of Public Health, Department of Biomedical, Metabolic and Neural Sciences, Center for Environmental, Nutritional and Genetic Epidemiology (CREAGEN), University of Modena and Reggio Emilia; ^3^Servizio Epidemiologia, Direzione Sanitaria-Azienda USL-IRCCS di Reggio Emilia, Reggio Emilia, Italy; ^4^Centre for Rheumatology; ^5^Institute for Orthopedic Surgery and Traumatology, Clinical Centre of Serbia, Belgrade, Serbia

###### **Correspondence:** V. Marusic

**Introduction:** Very few studies from the Southern European countries have assessed the risk factors for the development of surgical site infections (SSIs) after primarily clean orthopedic procedures with prosthetic joint implantation.

**Objectives:** To analyze risk factors (RF) for the development of 30-days SSIs after total hip arthroplasty (THA) and total knee arthroplasty (TKA).

**Methods:** A prospective cohort study was conducted at University Clinic for orthopedic surgery, Clinical Center of Serbia, from December 2016 to December 2017. All patients undergoing THA and TKA were enrolled in the study. SSIs were diagnosed on the basis of the Centers for Disease Control and Prevention definitions. Univariate and multivariate logistic regressions were performed.

**Results:** Out of 319 operative procedures (OP) performed, 67.1% were THA and 32.9% TKA. SSI occurred in 25 cases, with incidence rate of 7.8%. For THA and TKA, rates of total SSI were 7.48 (95% CI, 3.96-11.03) and 8.57 (95% CI, 3.32-13.92) respectively. Out of all SSI, the most represented were superficial incisional infections (64%) while others were deep infections. SSI cases mean age was 67.96±9.03 years and nearly half were females (52%). SSI *patients* required prolonged *hospitalization (30.2 vs. 19.4 days; p<0.001).* Spinal anesthesia (56% vs. 36.4%, SSI vs. w/o SSI; p=0.052) and prolonged AMP (> 1 day) (8.9 vs. 7.2 days, p=0.029), were risk factors for SSI development, while AMP on time, within 1h before operation was protective factor (p=0.040). First-generation cephalosporin was chosen in highest percentage (31.5%) for the AMP, then third-generation cephalosporin in 24.5%, and in 30.7% it was a combination of three antibiotics which included Vankomycin. Multivariate analysis identified only one major risk factor for SSIs: prolonged AMP (RR=1.17; 95%CI: 1.02-1.34; p=0.019).

**Conclusion:** Risk factors for the SSIs in orthopedic patients who undergo THA and TKA in Serbia do not influence heavily SSI risk except AMP. More adequate antimicrobial stewardship programs should be taken into account according to new hospital guideline.

**Disclosure of Interest**: None declared

### P23 RISK FACTOR ANALYSIS OF SURGICAL SITE INFECTIONS IN ELDERLY PATIENTS WITH INTESTINAL OBSTRUCTION AFTER EMERGENCY SURGERY

#### B. Gao^1^, S. Feng^2^, X. Xie^3^, H. Ge^4^, Z. Tang^4^, X. Yang^4^, W. An^5^, S. Hu^5^

##### ^1^Infectious diseases unit, Tianjin 4th Centre Hospital affiliated to Tianjin Medical University and Nankai University, Tianjin; ^2^Infection Control Department, First People’s Hospital of Zunyi; ^3^Department of Gastrointestinal Surgery, First People’s Hospital of Zunyi and 3rdaffiliated hospital of Zunyi Medical University, Zunyi; ^4^Department of Gastrointestinal Surgery, First People’s Hospital of Zunyi and 3rdaffiliated hospital of Zunyi Medical University; ^5^Infection Control Department, First People’s Hospital of Zunyi and 3rd affiliated hospital of Zunyi Medical University, Zunyi, Guizhou, China

###### **Correspondence:** B. Gao

**Introduction:** Infection control and prevention is one of core elements of healthcare quality improvement**.**

**Objectives:** To investigate the etiology of surgical site infections (SSIs) in elderly patients with intestinal obstruction after emergency surgery in order to reduce their occurrence.

**Methods:** We conducted a retrospective study of all patients aged≥60 years with an ileus who underwent an emergency surgery from Jan 2014 to Feb 2019 in a tertiary teaching hospital in western China. Clinical variables included age, sex, NNIS indexes, prophylactic use of antimicrobial agents, perioperative hemoglobin levels, serum albumin levels, hemorrhage and postoperative morbidity. Multivariate analysis was used to identify variables independently associated with SSIs. SSI was defined by the criteria of the Chinese national guideline for the prevention and control of SSIs persistent wound discharge or dehiscence, visible abscess or gangrene and bacterial contamination confirmed by discharge liquid culture.

**Results:** One hundred and eleven ileac patients aged≥60 years were included. All had documented antimicrobial prophylaxis of more than one dose; initial doses were not determinately administered within 30 min to 1 hour before the surgical procedures. Thirty-four patients were diagnosed with SSIs. A total of 38 bacterial strains were isolated from SSI patients, mainly *Escherichia coli* (15/38), *Enterococcus* (7/38), *Proteus* species (6/38) and other enterobacter (5/38). Multivariate logistic regression analysis indicated that the classification of incision site was an independent risk factor for SSI. Hospital length of stay of patients with SSIs was significantly longer than those without SSIs (30.91±10.91days vs 23.94±10.64 days, respectively; *P=0.008*).

**Conclusion:** Prevention kits and reasonable prophylactic use of antimicrobial agents should be introduced to reduce SSIs in the setting of elderly ileac patients undergoing an emergency surgery.

**Disclosure of Interest**: None declared

### P24 ETIOLOGY, INCIDENCE AND RISK FACTORS FOR MENINGITIS AFTER VENTRICULOPERITONEAL SHUNT PROCEDURES: A MULTICENTER STUDY

#### L. G. Giarola^1^, D. C. Silva^1^, H. R. Couto^1^, F. A. Bracarense^1^, F. L. Carvalho^1^, G. L. Souza^1^, R. F. Rocha^1^, A. N. Silveira^1^, C. D. Oliveira^1^, H. D. Carvalho^1^, B. R. Couto^2^, H. O. Pereira^3^

##### ^1^Centro universitário de Belo Horizonte; ^2^Hospital Lifecenter; ^3^Hospital Metropolitano Odilon Behrens, Belo Horizonte, Brazil

###### **Correspondence:** D. C. Silva

**Introduction:** Surgical site infection associated with shunt placement, treatment for hydrocephalus, is the most common complication and cause of mortality.

**Objectives:** The objective is to answer three questions: a) What is the risk of meningitis after shunt placement? b) What are the risk factors for meningitis? c) What main microorganisms cause meningitis?

**Methods:** Data based on NHSN/CDC protocols were collected between Jul/2015-Jun/2018 from 12 hospitals at Belo Horizonte, Brazil. Outcomes: meningitis, hospital death and total length of hospital stay. 26 independent variables evaluated by univariate and multivariate analysis. Sample size=926.

**Results:** 71 cases of meningitis diagnosed (risk=7.7% [I.C.95%=6.1%;9.6%]).. Mortality rate in patients without infection was 10%; hospital death of infected patients was 13%. Hospital days of stay in non-infected patients: mean=21; median = 9, std.dev. = 28; hospital stay in infected patients: mean =34, median =27, std. dev. = 37 (p=0.025). Three main risk factors identified by logistic regression model: age beneath two years (Odds Ratio = 3.20), preoperative hospital length of stay greater than four days (OR = 2.02) and surgical procedures besides ventricular shunt. 31% of all patients were <2 years old. 430 patients had more than four preoperative days. If a patient two years old has surgery four days post hospital admission, the risk of meningitis is increased from 9% to 18% (p=0.026). From 71 meningitis, in 45 (63%) the etiologic agent was identified: Staphylococcus aureus (33%), Staphylococcus epidermidis (22%), Acinetobacter sp (7%), Enterococcus sp (7%), Escherichia coli (7%), Pseudomonas sp (7%), and other (18%).

**Conclusion:** We identified two intrinsic risk factors for meningitis after ventricular shunt, age less than two years and multiple surgical procedures, and one extrinsic risk factor, the preoperative length of hospital stay.

**Disclosure of Interest**: None declared

### P25 PATIENTS WITH LOWER LEG SURGERIES AND MALE PATIENTS AS RISK GROUPS FOR POSTOPERATIVE WOUND INFECTIONS

#### M. M. Strybos, R. Otchwemah, J. Hoffmann, F. Mattner

##### Institute for Hygiene, Kliniken der Stadt Köln gGmbH, Cologne, Germany

###### **Correspondence:** M. M. Strybos

**Introduction:**

The project "HygArzt" (ZMVI1-2516FSB111), funded by the Federal Ministry of Health, is intended to investigate the effects of infection protection measures (IPM) by physicians responsible for hygiene.

**Objectives:**

As a starting point for the development of tailored IPM, the pre-intervention phase of the study was used to analyse which body parts after surgery (SX) and which patient groups entail a particular risk of postoperative wound infections (SSI).

**Methods:**

In order to identify SSI, clinical signs of infection were recorded according to KISS and CDC definitions. For this purpose, data of previous illnesses as well as of current and previous infections from the hospital management system, admission forms, discharge letters and nursing documentation were aggregated with current patient data and laboratory findings. In addition, early visits were made three times a week to record signs of infection that had not yet been documented.

For the evaluation "infections brought along" (165 SX) as well as multiple surgeries from the time of the occurrence of the SSI (91 SX) were excluded and only new infections (42 SX) were considered.

**Results:**

In the pre-intervention phase of the study, 1978 surgeries were executed (in 469 cases several surgeries on the same patient) with a total SSI infection rate (InfR) of 2.44% (CI 95% 1.7; 3.1) for the surgeries. The body parts with the most surgeries were the knee (552 surgeries) with an infection rate of 1.44%, lower leg (487) InfR = 4.22%, hip (311) InfR = 3.49%, shoulder (179) InfR = 1.21%, forearm (153) InfR = 2.82%, thigh (89) InfR = 2.49%. A *χ²*-test showed that the risk of getting an SSI with a lower leg surgery was significantly higher (*p* = .008) than with all other surgeries. The evaluation of wound infections by sex (♂ = 1171 SX; ♀ = 807 SX) showed a higher probability of men receiving SSI for all surgeries (♂ = 37 SSI; ♀ = 10 SSI) (*p* = .024). This was also shown in relation to SSI only for lower legs (♂ = 325 SX, 16 SSI; ♀ = 152 SX, 1 SSI) (*p* = .019).

**Conclusion:**

The analysis of the patient data identified two risk groups with an increased risk to develop an SSI. When developing preventive measures, these risk groups should be specifically targeted.

**Disclosure of Interest**: None declared

### P26 IS THERE A NEED FOR CHANGING PERIOPERATIVE PROPHYLAXIS DURING ANTIBIOTIC THERAPY AND REPETITIVE DEBRIDEMENT FOR ORTHOPEDIC INFECTIONS?

#### I. Uçkay^1^, M. Abbas^2^, T. Studhalter^3^, L. Wuarin^2^, S. Harbarth^2^

##### ^1^Balgrist University Hospital; ^2^Geneva University Hospitals, Geneva; ^3^Balgrist University Hospital, Zürich, Switzerland

###### **Correspondence:** I. Uçkay

**Introduction:** The appropriate antibiotic prophylaxis for repetitive debridement on infected orthopedic sites is unclear.

**Objectives:** We establish the epidemiology of surgical site infections of surgical site infections in adult orthopedic surgery.

**Methods:** We performed a retrospective cohort study examining repetitive SSIs occurring in infected adult orthopedic patients; and by performing group comparisons and logistic regression analyses.

**Results:** Among 2480 first episodes of various orthopedic infections (median patient age 56 years, 833 immunosuppressed: implant-related (n=648); osteoarticular (n=1153); soft tissue infection (n=1327), 862 (862/2480; 35%) revealed multiple debridements for the same episode. The median number of debridements was 1 (range, 1-15 interventions). Upon repetitive intraoperative sampling, we detected pathogens in 507 cases (507/862; 59%), of which 265 new SSIs (265/862; 31%), of which the microorganisms of 174 episodes (20%) were resistant to current antibiotic therapy. In multivariate analysis, repetitive debridements were associated with new SSIs (odds ratio regarding the “second look” 13.7, 95%CI 8.8-21.2). These new and resistant pathogens were Gram-positive in 64% and Gram-negative in 36%. We failed to identify a predicative microbiological pattern. Likewise, we failed to identify an optimal theoretical antibiotic prophylaxis during re-debridement for cases that were already under treatment.

**Conclusion:** In our single-center large cohort and regarding multi-debrided orthopedic infections, new SSIs with resistant pathogens occurred in 20%; and already after the 2nd look; with no ideal supplementary prophylaxis regimen identified.

**Disclosure of Interest**: None declared

### P27 HYPERGLYCEMIA MANAGEMENT AND ITS ASSOCIATION WITH STERNAL SITE INFECTIONS AMONG CABG PATIENTS IN A QUATERNARY CARE HOSPITAL

#### A. M. Alexander

##### Quality Assurance, Aster Medcity, Cochin, Kerala, Cochin, India

###### **Correspondence:** A. M. Alexander

**Introduction:** Peri-operative hyperglycemia is identified as a significant risk factor for deep sternal wound infection. During HIC surveillance, a higher than usual SSI rates were noted.A preliminary audit done showed poor Post-OP glycemic control among patients who developed SSI despite of having an established in- hospital protocol.Gaps in adherence towards the glycemia protocol were identified and the protocol was re-introduced.All CABG patients were tracked via watsapp messenger to ensure RBS <200mg% at home during their post-op period,up to 2 weeks.

**Objectives:** The Objectives of this project was to assess the adherence towards Glycaemic management among CABG patients during pre and post implementation phase and to assess the association of post discharge Glycemic management and Occurrence of sternal site infections among CABG patients.

**Methods:** A prospective data collection method with Purposive sampling and PDCA Cycle was adopted for the entire framework of this project. All CABG patients with sternal site infections from January 2018 to June 2018, were taken as the control group. All CABG cases between the months of October 2018 to December 2018 were taken as study samples during the process of study. Point surveillance of those CABG patients, prior to surgery, entire peri-op period and post discharge follow up via watsapp messenger up to 2 weeks, was done to ensure RBS <200mg%. The patients who acquired sternal site infections between the months of October 2018 to December 2018 were considered as the experimental group.

**Results:** During the course of post implementation phase, Weekly feedbacks on perioperative glycemic control were given to the respective departments to improve their outcome. Tracking of discharged CABG patients via watsapp messenger ensured the patients compliance to RBS< 200mg/dl. Adherence towards Glycemic management protocol in post-op period was improved in the post implementation phase (82.195%).In the Post-Implementation Phase,the average sternal site infections reduced to 0.33%.The study conducted concluded that, there is a significant association between sternal site infection rates and the post discharge glycemic management, as ‘*p’-*value (P = < 0. 001) was significant.

**Conclusion:** This project demonstrated that post discharge glycemic control is an important risk factor which can be favorably modified by patient education and engagement.

**Disclosure of Interest**: None declared

### P28 SURGICAL SITE INFECTION AFTER BARIATRIC SURGERY: A SMALL RISK THAT DEFINES LIFE AND DEATH OF PATIENTS

#### L. G. Giarola^1^, G. G. Gianeschi^1^, J. C. Maciel^1^, R. C. Á. P. Paranhos^1^, T. G. Pinheiro^1^, Y. A. M. Amaral^1^, D. C. Silva^1^, H. R. Couto^1^, C. E. F. Starling^2^, B. R. G. M. Couto^2^

##### ^1^Centro universitário de Belo Horizonte; ^2^Hospital Lifecenter, Belo Horizonte, Brazil

###### **Correspondence:** L. G. Giarola

**Introduction:** Surgical site infection (SSI) in bariatric surgery can lead to devastating outcomessuch as peritonitis, sepsis, septic shock and organ space infection.

**Objectives:** The objective of our study is to answer four questions: a) What is the SSI risk after bariatric surgery? b) What are the risk factors for SSI after bariatric surgery? c) What are the main outcomes to SSI in bariatric surgery? d) What are the main bacteria responsible for SSI in bariatric surgery?

**Methods:** A retrospective cohort study assessed 8,672 patients undergoing bariatric surgery between 2014/Jan and 2018/Dec from two hospitals at Belo Horizonte, Brazil. Data were gathered by standardized methods defined by the National Healthcare Safety Network (NHSN)/CDC procedure-associated protocols for routine SSI surveillance. Outcome: SSI, hospital death and total length of hospital stay. 20 preoperative and operative variables were evaluated by univariate and multivariate analysis (logistic regression).

**Results:** 77 SSI were diagnosed (risk = 0.9% [C.I.95% = 0.7%;1.1%]). Mortality rate in patients, without infection was 0.03% (3/8,589) while hospital death of infected patients was 4% (3/77; RR = 112; p < 0.001). Hospital length of stay in non-infected patients (days): mean = 2, std.dev.= 0.9; hospital stay in infected patients: mean = 7, std. dev. = 15.6 (p < 0.001). Two main factors associated with SSI after bariatric surgery were identified by logistic regression: duration of procedure (hours), OR = 1.4;p=0.001, and laparoscopy procedure, OR = 0.3;p=0.020. From 77 SSIs, in 28 (36%) we identified 34 etiologic agents. The majority of SSI (59%) was caused by species of Streptococcus (32%), Klebsiella (15%), and Enterobacter (12%).

**Conclusion:** SSI is rare after bariatric surgery, however, when it happens, it’s a disaster for the patient and is mainly caused by species of Streptococcus. The incidence of SSI can be reduced significantly when laparoscopy procedure is used and the surgeon is able to perform a rapid surgery.

**Disclosure of Interest**: None declared

### P29 MAJOR RISK FACTORS CONTRIBUTING TO SURGICAL SITE INFECTIONS POST-CAESARIAN SECTION

#### N. A. Bouafia^1^, W. A. Mazi^1^, W. S. Ahmad^2^, S. H. Alwagdani^1^, A. A. AlTalhy^3^, M. H. AlZahrani^4^, A. A. AlAmri^4^, A. M. Dahlawi^5^, M. R. ALYAMI^6^

##### ^1^INFECTION PREVENTION AND CONTROL, KING FAISAL MEDICAL COMPLEX; ^2^OBSTETRIC AND GYNECOLOGY DEPARTMENT, MATERNITY TOWER -KING FAISAL MEDICAL COMPLEX; ^3^HEAD OF OPERATING ROOM AND MEDICAL DIRECTOR, KING FAISAL MEDICAL COMPLEX; ^4^OPERATING ROOM; ^5^MEDICAL DIRECTOR, MATERNITY TOWER -KING FAISAL MEDICAL COMPLEX; ^6^HOSPITAL DIRECTOR, KING FAISAL MEDICAL COMPLEX, TAIF, Saudi Arabia

###### **Correspondence:** N. A. Bouafia

**Introduction:** Surgical site infection post cesarian section (CS-SSI) is a major cause of prolonged hospital stay and poses a burden to the health care system

**Objectives:** To determine risk factors associated with CS-SSI in order to improve patient care

**Methods:** A prospective study was conducted for all women undergone caesarean section procedures during 2018 in King Faisal Medical Complex-Taif, Kingdom of Saudi Arabia**.** Patients’ socio-demographic and clinical data were collected from patient’s file. CS-SSI was identified and incidence rate was calculated using the Centers for Disease Control and Prevention and National Healthcare Safety Network (NHSN, USA) criteria based on ongoing and re-admission surveillance methods**.** Risk factor for CS-SSI was assessed and analysed using SPSS version 18**.**

**Results:** In total, 30 patients developed CS-SSI among 3268 women underwent CS in 2018. The cumulative incidence rate of CS-SSI was 0.82 % (ranged from 0.24-2.21%). CS-SSIs were classified as superficial (66.7%), deep (30%) and organ (3.3%) infections. More than half of SSI (53.3%) occurred during the first week following the CS. The most frequent risk factors for CS-SSI revealed in our study were: immediate emergency CS (83.3%) followed by higher body mass index (BMI > 30 kg/m2) with standard dosage of cefazoline 2 gram prophylactic antibiotic within 60 minutes before incision (76.7%), multiple gravida patients (60%), general anesthesia (60%), previous CS (40%) and Premature rupture of membrane (33.3%).

**Conclusion:** Our incidence rate of CS-SSI is lower than NHSN hospitals and risk factors revealed in this study are mostly associated to population characteristics and are worldwide documented. However, more research regarding adjustment of antibio-prophylaxis dose to BMI are needed.

**Disclosure of Interest**: None declared

### P30 INCIDENCE AND RISK FACTORS FOR SURGICAL SITE INFECTIONS IN COLON SURGERY: A MULTICENTERED STUDY IN TWELVE BRAZILIAN HOSPITALS

#### G. Lauar E Souza, H. Dias Duarte de Carvalho, C. de Deus Martins Oliveira, A. André Martins de Araújo, B. Roberto Gonçalves Marinho Couto

##### Instituto de Ciências Biológicas e da Saúde, Centro Universitário de Belo Horizonte, Belo Horizonte, Brazil

###### **Correspondence:** G. Lauar E Souza

**Introduction:** Infection in colon surgery, and all surgery, is very harmful for the patient. Better understanding of its risk factors may help improve patient safety.

**Objectives:** This quantitative, multicentered study calculates surgical site infection (SSI) risk, describes risk factors, average hospital length of stay, compares length of stay in infected and non-infected patients, infection impact on mortality rates in colon surgery.

**Methods:** Data was collected between 2012 and 2017 from 12 hospitals from Belo Horizonte, a major city in Brazil (2.5 million inhabitants). Outcome variables were: SSI, hospital death and total length of hospital stay(days). The 23 independent variables were analyzed using Epi Info and applying statistical two-tailed test hypothesis with significance level of 5%.

**Results:** A total of 8,261 surgeries were analyzed and 284 patients presented SSI, a risk of 3.4% (Confidence interval (CI) of 95%=[3.1%;3.9%]).The variables associated to SSI risk were: patient age above 70 years (Relative Risk (RR)=1.9;p<0.001), general anesthesia (RR=7.9;p<0.001), ASA Score above 2 (RR=2.2;p<0.001), surgery duration above 2h (RR=4.8;p<0.001), emergency surgery (RR1.7;p=0.022), concomitant surgical procedure (RR=4.4;p<0.001), first hospitalization of the patient(RR=0.65;p=0.002), postoperative hospital stay above 4 days (RR=2.6;p<0.001). The average time of hospital stay in infected patients was 25 days (standard deviation-SD=30.5 days). In non-infected patients, average time was 7 days (SD=16 days; p<0.001). The mortality among patients with SSI was 22%, observed to be 4% in non-infected (p<0.001). General anesthesia, with the highest associated relative risk, is the main risk factor for SSI in colon surgery.

**Conclusion:** There is a 95% chance of incidence of infection in 3 out of 100 patients. The average length of stay in infected patients was 3.6 times higher, and mortality rates in infected patients is 5.5 times higher. Lengthier surgery time (>2hours) is a risk factor for SSI. Evidence suggest that healthcare professionals should be concerned about SSI, and surgeons should look forward to reduce the time of surgery when possible, without neglecting patient safety.

**Disclosure of Interest**: None declared

### P31 RISK PREDICTION FOR SURGICAL SITE INFECTION IN CRANIOTOMY PATIENTS

#### F. H. B. D. Souza, B. R. G. M. Couto, J. D. O. Matias, L. L. de Araújo, L. S. Rossati, L. R. Polidoro

##### Centro Universitário de Belo Horizonte - UNIBH, Belo Horizonte, Brazil

###### **Correspondence:** F. H. B. D. Souza

**Introduction:** Based on data obtained from hospitals in the region of Belo Horizonte city (Brazil), the evaluation of relevant factors such as: deaths, age, duration of surgery, number of hospitalizations, potential contamination and surgical site infection (SSI) resulting from surgeries of craniotomy was performed. The possibility of predictions of SSI was analyzed through pattern recognition algorithms based on Artificial Neural Networks of the MLP (Multilayer Perceptron) type.

**Objectives:** This article aims to demonstrate the SSI predictive power of pattern recognition algorithms as an analysis of the event with the aid of current technologies.

**Methods:** Data were collected by Hospital Infection Control Committees in hospitals of Belo Horizonte between 2016 and 2018. Noisy records were filtered and the occurrences were analyzed. Finally, the predictive power of MLPs to predict SSI was evaluated, where were experimented: 5 types of MLPs (Momentum, Backpropagation Standard, Weight Decay, Resilient Propagation and Quick Propagation); 3, 5, 7 and 10 neurons in the hidden layer; with varying resampling in quantity of records for test (65% and 75%) and for validation (35% and 25%). Comparisons occurred by measuring the AUC (Area Under the ROC Curve - ranging from 0 to 1).

**Results:** From 1096 records, 289 were intact for analysis, where: 16% were deaths; an age group of 40 to 65 years (average of 56); average time of 186 minutes of surgery (ranging from 95 to 250 minutes); the number of hospitalizations ranged from 1 (90.6%) to 8 (0.3%); potential contamination was in 2.7% as contaminated, 23.5% potentially contaminated and 72.3% as clean; the occurrence of SSI reached 4%. The prediction AUCs ranging from 0.7 to 0.994.

**Conclusion:** After filtering all the records, a high index of noise was recorded due to subjectivity at the moments of data collection. Considering the data used, 16% of deaths were reported. Linking the deaths to SSI alone would not be possible (only 4%). Finally, the analyzed structures (MLPs) demonstrated a relevant predictive power capable of guiding intelligent monitoring software.

**Disclosure of Interest**: None declared

### P32 POWER OF PREDICTION OF SURGICAL SITE INFECTION IN CAESAREAN SURGERY USING PATTERN-BASED ANALYSIS BASED ON MULTILAYER PERCEPTRON ARTIFICIAL NEURAL NETWORKS

#### F. H. B. D. Souza, B. R. G. M. Couto, A. M. R. Maroca, I. H. S. Soares, J. M. D. C. Duarte, S. C. Costa

##### Centro Universitário de Belo Horizonte - UNIBH, Belo Horizonte, Brazil

###### **Correspondence:** F. H. B. D. Souza

**Introduction:** The purpose of this paper is to report the power of predictions of surgical site infections (SSI) in obstetrics, using neural networks based on MLPs (Multi Layer Perceptron). Data were collected from hospitals in Belo Horizonte city (Brazil) between 2016 and 2018.

**Objectives:** This paper aims to statistically evaluate the profile of surgeries and test the predictive power of SSI of pattern recognition algorithms, in the case of Artificial Neural Networks of the type MLP (Multilayer Perceptron).

**Methods:** A data collection in 6 different hospitals was performed and filtered noisy records, which enabled a statistical analysis of the profile of the evaluated hospitals. Thus, an SSI prediction power of five types of MLPs (Backpropagation Standard, Momentum, Resilient Propagation, Weight Decay and Quick Propagation) was made with configurations: 3, 5, 7 and 10 neurons in the hidden layer; a division of the database for the resampling process 65% (or 75%) for learning, 35% (or 25%) for validation; and a comparison by measuring the AUC (Area Under the Curve - ranging from 0 to 1).

**Results:** A total of 7,698 records (with 46 characteristics) were collected, with 3,517 consistent evaluated. It was recorded: average of 33 years of the patients; 80% the surgical accomplishment occurred in the first hospitalization; the majority of surgeries had estimated durations between 44 minutes and 88 minutes; potential contamination around 90%; 73.1% of emergency surgeries; 99.6% of cases with 2 professionals and 2.5% of SSI. The MLPs reached AUCs of 0.98.

**Conclusion:** Despite the high noise index of the database, it was possible to sample relevant for the evaluation of the profile of hospitals in Belo Horizonte. The predictive process presented an accuracy of extreme relevance, reaching 0.98 for the SSI prediction power.

**Disclosure of Interest**: None declared

### P33 MEDICINE ALLIED TO TECHNOLOGY: THE USE OF ARTIFICIAL NEURAL NETWORKS IN THE PREDICTION OF SURGICAL SITE INFECTION FOR GENERAL SURGERY SERVICE

#### F. H. B. D. Souza, B. R. G. M. Couto, G. M. Braga, J. A. Teixeira, R. C. Santos, J. M. C. Martins, K. S. D. Sousa, D. N. De Souza, G. B. Alves

##### Centro Universitário de Belo Horizonte - UNIBH, Belo Horizonte, Brazil

###### **Correspondence:** F. H. B. D. Souza

**Introduction:** This research represents a trial of surgical site infection (SSI) in patients submitted to general surgery procedures in hospitals in Belo Horizonte, a 3,000,000 inhabitants city from Brazil.

**Objectives:** The objective is to statistically evaluate these incidences and to enable a study of the SSI prediction power of pattern recognition algorithms, in the case of Artificial Neural Networks of the type MLP (Multilayer Perceptron).

**Methods:** Data collection on SSI was performed in 5 different hospitals between July 2016 and June 2018 and performed three procedures: a preprocessing of the database collected for valid sample use; a statistical analysis on the profile of the hospitals collected and; an evaluation of the predictive power of five types of MLPs (Backpropagation Standard, Momentum, Resilient Propagation, Weight Decay and Quick Propagation) for SSI prediction. The MLPs were tested with 3, 5, 7 and 10 neurons in the hidden layer and with a partitioning of the database for the resampling process (65% or 75% for test, 35% or 25% for validation). They were compared by measuring the AUC (Area Under the Curve - ranging from 0 to 1) presented for each of the configurations.

**Results:** 13,383 data were collected and 7,566 records were usable where: 2.0% of SSI; the predominance of patients' ages was between 35 and 62 years; the mean duration of the procedure was 101 minutes; the mean hospitalization time (without SSI) was 4 days, versus 17 days in the positive cases. The predictive power of the proposed settings was between 0 and 0.6.

**Conclusion:** Despite the high noise index of the database, it was possible to sample relevant for the evaluation of the profile of General Surgery Service patients. However, for the predictive process, although there are results higher than 0.5, the database requires more SSI case samples, since only 2% of positive samples unbalanced the database.

**Disclosure of Interest**: None declared

### P34 USING ARTIFICIAL NEURAL NETWORKS TO PREDICT OF SURGICAL SITE INFECTION IN ORTHOPEDIC PATIENTS

#### F. H. B. D. Souza, B. R. G. M. Couto, G. S. D. M. Serpa, I. P. D. A. L. Abelha, L. F. Valadão, M. E. C. Bernardes, M. C. B. Vidigal, Y. D. A. E. S. Haddad

##### Centro Universitário de Belo Horizonte - UNIBH, Belo Horizonte, Brazil

###### **Correspondence:** F. H. B. D. Souza

**Introduction:** This paper demonstrates an initial experiment to predict the risk of surgical site infection (SSI) after Orthopedic procedures in Belo Horizonte, a 3,000,000 inhabitants city from Brazil.

**Objectives:** The objectives are: collect data in 6 hospitals between 2016 and 2018; filtering the resulting database; benchmarking statistical assessments; to submit the resulting database to an initial experiment with MLP (Multilayer Perceptron) based pattern recognition algorithms for the evaluation of SSI occurrence prediction power.

**Methods:** The data were collected and the resulting database was filtered for use of intact samples. A statistical analysis was applied defining the behavioral profile. An evaluation of the SSI predictive power of five types of MLPs (Backpropagation Standard, Momentum, Resilient Propagation, Weight Decay and Quick Propagation) was performed. The MLPs were tested with 3, 5, 7 and 10 neurons in the hidden layer; a resampling process of 65% (and 75%) for learning and 35% (and 25%) for validation; and measuring the AUCs (Area Under the ROC Curve - between 0 and 1).

**Results:** 10069 records were collected with 46 variables each. Only 3673 were intact for study, where: 77 contracted SSI; length of hospitalization ranged from 0 to 186 days (mean 5 days for normal and 22 for SSI cases); 29 cases of death; duration of surgeries of 125 minutes (the majority concentrated between 90 and 190) and the mean age of the patients is approximately 50 years old. The initial experiments for SSI prediction showed a maximum AUC of 0.5845.

**Conclusion:** Despite the high noise index of the database, a relevant sample was obtained for the hospitals evaluated. However, for the predictive process, despite some results higher than 0.5, the database requires more SSI case samples, since only less than 1% of SSI samples generated an unbalance of the database.

**Disclosure of Interest**: None declared

### P35 RISK OF SURGICAL SITE INFECTION AFTER CARDIAC SURGERIES: PATTERN-BASED ANALYSIS BASED ON MULTILAYER PERCEPTRON ARTIFICIAL NEURAL NETWORKS

#### F. H. B. D. Souza, B. R. G. M. Couto, A. C. L. Michelini, A. P. R. de Melo, L. G. Guerra, L. M. B. Costa, M. V. Melo, O. J. de Oliveira Junior

##### ^1^Centro Universitário de Belo Horizonte - UNIBH, Belo Horizonte, Brazil

###### **Correspondence:** F. H. B. D. Souza

**Introduction:** This paper proposes a pattern-based analysis to predict the probability of surgical site infection (SSI) in cardiac surgeries in four hospitals in Belo Horizonte (Brazil), according to a data collection between 2016 and 2018.

**Objectives:** As specific objectives this work is to carry out a data collection and processing process; perform a statistical evaluation on the data involved and then a battery of experiments based on Multilayer Perceptron to analyze the probability of SSI prediction.

**Methods:** After the data collection: a filtering of the database collected for use of intact sample was performed; a statistical analysis on the profile of the hospitals collected; and finally, experiments to evaluate the SSI predictive power of five types of MLPs (Backpropagation Standard, Momentum, Resilient Propagation, Weight Decay and Quick Propagation), each with: 3, 5, 7 and 10 neurons in the hidden layer; a resampling process with 65% (and 75%) data for learning, 35% (and 25%) for validation; a comparison by measuring the AUC (Area Under the Curve - ranging from 0 to 1) for accuracy.

**Results:** 3078 records were collected, each with 46 variables, where 459 were used. It was found: average age of 65 years; approximately 250 surgeries with general anesthesia; the average time of surgery in 183 minutes (between 95 and 280 minutes); post-surgery hospitalization time ranging from 0 to 115 days (average 13 days); incidence of SSI 4.1% (these patients remained on average 29 hospitalized days) and a mortality rate of 10.0%. The predictive power ranged from 0.46 to 0.73.

**Conclusion:** In spite of the high index of noise in the database, it was possible an interesting sample for the evaluation of the profile of the hospitals. The predictive process, had achieved results higher than 0.5, figuring a very promising technique for predict process.

**Disclosure of Interest**: None declared

### P36 ALGORITHMS FOR PATTERN RECOGNITION FOR PREDICTION OF SURGICAL SITE INFECTION IN VASCULAR SURGERIES

#### F. H. B. D. Souza, B. R. G. M. Couto, F. B. de Mello, I. Caldeira, J. S. F. R. de Morais, L. G. Mota, M. F. Dumont, R. W. Savio, S. M. Mazzoni

##### ^1^Centro Universitário de Belo Horizonte - UNIBH, Belo Horizonte, Brazil

###### **Correspondence:** F. H. B. D. Souza

**Introduction:** This paper demonstrates an analysis based on pattern recognition algorithms based on artificial neural networks (type MLP - Multilayer Perceptron) for the prediction of surgical site infection (SSI) for vascular surgery.

**Objectives:** The main objective of this study is to demonstrate the predictive power of recognition algorithms based on MLP for vascular surgery in hospitals in Belo Horizonte.

**Methods:** Firstly, a data collection was performed between 2016 and 2018 in hospitals in Belo Horizonte, a 3,000,000 inhabitants city from Brazil, by the Hospital Infection Control Committees (CCIH). Thus, a filtering of the collected records (removal of noisy) and a behavioral analysis of the hospitals was performed. Finally, it was evaluated the SSI predictive power of five types of MLPs (Momentum, Backpropagation Standard, Weight Decay, Resilient Propagation and Quick Propagation). The MLP’s were tested with 3, 5, 7 and 10 neurons in the hidden layer. In the resampling process, the configurations of 65% (or 75%) for testing and 35% (or 25%) for validation were executed. They were compared by measuring the AUC (Area Under the ROC Curve - ranging from 0 to 1) for each configuration.

**Results:** From 3882 collected records, 2286 were intact. In a behavioral analysis: 2.4% present SSI; almost 80% of the cases vascular surgeries were in the peripheral regions of the body, 90% were not of urgency or emergency; cases of limb amputation reached 462 (approximately 25% of cases). Finally, the 40 experiments with MLPs showed AUCs between 0.85 and 0.996 for SSI prediction.

**Conclusion:** SSI values are lower than 2.5%, confirming the low degree of life risk, due to the time it takes to perform procedures to clean the surgical sites, which avoids contamination and because they are low impact surgeries. life of the patient. The database was useful for the prediction process of SSIs with AUCs of 0.996, allowing for intelligent software for monitoring with prediction of considerable SSI assertiveness, even with a considerable noise index and with imbalance (only 2,4% of SSI cases) for such surgery.

**Disclosure of Interest**: None declared

## Poster session: Clostridium difficile

### P37 DIARRHEAL SYMPTOMS EXTRACTED FROM CLINICAL NOTES FOR PATIENTS TESTING POSITIVE FOR CLOSTRIDIOIDES DIFFICILE IN THE INPATIENT, OUTPATIENT, AND LONG-TERM CARE SETTINGS

#### V. W. Stevens, G. Divita, K. Khader, M. Samore

##### IDEAS Center of Innovation, VA Salt Lake City Health Care System, Salt Lake City, United States

###### **Correspondence:** V. W. Stevens

**Introduction:** A clinical diagnosis of *Clostridioides difficile* infection (CDI) requires the presence of diarrheal symptoms and adequate documentation, posing substantial challenges for electronic surveillance efforts and large-scale epidemiological studies in electronic health record data

**Objectives:** To describe patterns in mentions of diarrheal symptoms in clinical notes among patients with a positive laboratory test for *C. difficile*

**Methods:** We conducted a retrospective cohort study of all positive tests for *C. difficile* in the US Department of Veterans Affairs health system in 2016. Episodes were classified as healthcare-acquired (HA), community onset-healthcare associated (CO-HCA), or presumed community-acquired (p-CA) based on Centers for Disease Control surveillance criteria. All clinical notes within the 7 days before and after the positive test were selected. Mentions of diarrhea were extracted using an ontology and dictionary-based natural language processing pipeline built using the V3NLP framework. Two reviewers manually classified 600 asserted snippets as true positive or false positive. NLP performance was measured using the positive predictive value (PPV).

**Results:** In 2016, there were 11,826 positive laboratory tests for *C. difficile*. Of these, 2,735 (23%) were HA, 3,576 (30%) were CO-HCA, and 5,515 (47%) were p-CA. The positive predictive value of the NLP tool was 86.4%. We identified mentions of diarrhea within 2 days before or after a positive test in 7,361 (62.2%) of all episodes: 1,803 (65.9%), 2,524 (70.6%), and 3,034 (55.0%) of HA, CO-HCA, and p-CA episodes, respectively. Removing duplicates within 14 days of a prior positive only slightly increased the yield (62.9% of all episodes). 60% of mentions occurred between 2 days before to 2 days after the positive test.

**Conclusion:** Extraction of diarrheal symptoms from clinical note text has the potential to enhance studies of the epidemiology and transmission of CDI. We identified evidence of diarrhea within 2 days of a positive test in approximately 62% of episodes. Further work is needed to determine whether the absence of diarrhea mentions reflects inappropriate testing of patients without diarrhea, under-documentation of diarrheal symptoms, or other factors.

**Disclosure of Interest**: None declared

### P38 COMMUNITY VERSUS HEALTHCARE-ASSOCIATED CLOSTRIDIUM DIFFICILE INFECTION: A 12 YEARS PROSPECTIVE STUDY IN A FRENCH UNIVERSITY HOSPITAL

#### N. Khanafer^1,2^, C. U. Edouard Herriot Hospital^2^, L. Oltra^2^, V. Pergay^1,2^, O. Dauwalder^2^, F. Vandenesch^1,2^, P. Vanhems^1,2^

##### ^1^Lyon 1 University; ^2^Hospices Civils de Lyon, Lyon, France

###### **Correspondence:** N. Khanafer

**Introduction:** *C. difficile* infection (CDI) weighs heavily on healthcare system due to increased incidence, morbidity and mortality, as well as costs. CDI is mainly considered as a health-care associated (HCA) after exposure to broad-spectrum antibiotics. However, CDI has been reported outside health care institutions in people previously thought to be at low risk.

**Objectives:** The objective of this study was to compare characteristics of CDI cases regarding the infection presumed acquired in the community or in the hospital.

**Methods:** Between November 2006 and December 2018, a prospective surveillance study of CDI was conducted in a 900-bed French university hospital. National and European definitions of CDI case, relapses and origin of acquisition were applied. Standardized questionnaire was used for data collection.

**Results:** A total of 931 (=989 episodes) patients were included with a mean incidence rate of 2.3 per 1000 hospital-stays. Most of episodes were HCA (76.5%). The remaining cases were community-acquired (CA) (18.3%) or unknown origin (5.2%). The mean age of patients was 60.1 and 67.5 years for CA and HCA cases respectively (P<0.001) and 30.7% of CA patients were ≤45 years (P<0.001). In CA cases, women were more prevalent (59.2% vs 48.9% in HCA-CDI, P=0.01). CA-CDI group had lower rate of recent antimicrobial exposure (41% vs 79%, P<0.001), proton pomp inhibitor (39.5% vs 61.7%, P<0.001) and gastrointestinal surgery (2.37% vs 7.8%, P=0.008). Fever (>38°C), abdominal pain and ileus were significantly more frequent in CA cases (37.3% vs 26.6%; 45.5% vs 24.1% and 4.3% vs 1.2% respectively). Pseudomembranous colitis was more frequent in CA group compared to HCA cases (7.7% vs 4.5%, P=0.07). However, relapses and death were more frequent in HCA-CDI (8.5% vs 5.7% and 11% vs 6.6% respectively).

**Conclusion:** We found that approximately 20% of all CDI cases were CA with most of half not exposed to antimicrobial or proton pomp inhibitor drugs. Monitoring and active surveillance of CDI is needed to improve our understanding of the changing epidemiology of the disease.

**Disclosure of Interest**: None declared

### P39 MULTI-COMPONENT STRATEGY TO PREVENT C. DIFFICILE INFECTIONS IN ACUTE CARE HOSPITAL

#### P. Grzesiowski^1^, D. Pawlik^2^, A. Kadecka^3^, A. Sakowska^4^

##### ^1^Centre of Postgraduate Medical Education, Foundation for Infection Prevention Institute, Warsaw; ^2^Microbiology, Primary Hospital Makow Maz., Makow; ^3^Secondary Hospital , Ciechanow, Poland; ^4^Microbiology, Secondary Hospital, Ciechanow, Poland

###### **Correspondence:** P. Grzesiowski

**Introduction:** Clostridioides difficile infections are currently one of the most important public health threats in developed countries. In Poland, during last 10 years, hospital C. difficile infections increased significantly, reaching over 15,000 cases per year associated with high morbidity, mortality, and economic costs.

**Objectives:** In order to reduce the incidence of hospital C. difficile infections, we performed the prospective, single center observational study, on the assessment of the effectiveness of multi-component strategy.

**Methods:** Study setting: secondary acute 650-bed hospital

Study period: 2014-2018

Study method: single center, prospective, observational study

Bundle intervention: multi-component strategy consisted of hand hygiene implementation according to WHO guidelines, environmental cleaning improvement, fumigation of isolation rooms with hydrogen peroxide after patient discharge, regular quality control of cleaning procedures by using fluorescent marking gel (UV-DAZO), single room isolation of infected patients, antibiotic stewardship and medical staff education with regular feedback.

**Results:** During study period we observed significant, gradual, 4-fold decrease of hospital C. difficile infections (145 vs 38 cases), with increase of hand hygiene procedures as measured by alcohol hand rub usage (5,4L vs 27 L/1000 patient days) and improvement in cleaning high-touch surfaces as measured by removed fluorescent gel marks (45% vs 98%).

**Conclusion:** The bundle intervention significantly reduced C. difficile hospital infection rates. Results of our study support effectiveness the multi-component strategy based on hand hygiene, environmental cleaning, isolation procedures, antibiotic stewardship and staff education.

**References**

Barker A., Ngam C., Musuuza J. et al: Reducing Clostridium difficile in the inpatient setting: A systematic review of the adherence to and effectiveness of C. difficile prevention bundles. Infect Control Hosp Epidemiol. 2017; 38(6): 639–650.

Dubberke E, Carling P, Carrico R, et al. Strategies to prevent Clostridium difficile infections in acute care hospitals: 2014 update. Infect Control Hosp Epidemiol. 2014;35:628–645.

Mattner F, Winterfeld I, Oswald B, Solbach W. Successful bundle of prevention measures against a high CDAD incidence at a university hospital. Hyg Med. 2008;33:346–352.

**Disclosure of Interest**: None declared

### P40 CLOSTRIDIOIDES DIFFICILE INFECTION: APPLICATION OF A 4 DAYS CUT-OFF CLASSIFICATION

#### R. Loss^1^, L. Arnoldo^2^, R. Aschbacher^3^, M. Lopez^1^, P. Santa^1^, E. Pagani^3^, M. Bombonato^1^, F. Girardi^1^

##### ^1^Hospital Hygiene , Bozen; ^2^University of Udine, Udine; ^3^Laboratory of Microbiology , Bozen, Italy

###### **Correspondence:** R. Loss

**Introduction:** *Clostridioides difficile* infection (CDI) is the leading cause of antibiotic associated diarrhea in hospitalized patients, with a variable incubation time ranging from 2 to 19 days. According to a recent proposal (Mc Lure et al. 2018), the recommended 48 h cut- off can overestimate the incidence of hospital acquired CDI (HA CDI), because of high sensitivity but poor specificity to detect infection acquired during hospital staying.

**Objectives:** We conducted a retrospective study to evaluate the incidence and epidemiology of CDI in an Italian tertiary care hospital and we reclassified CDI cases using a cut-off of 4 days.

**Methods:** All laboratory diagnosis based cases of CDI of a 3 years period were reviewed. Each case was classified according to ECDC criteria as HA CDI, Community -acquired CDI (CA CDI) and indeterminate and reclassified using a cut-off of 4 days as HA CDI and non HA CDI.

**Results:** Out of 183 CD positive patients, 120 cases referred to hospitalized patients and were analyzed. Incidence remained stable around 3,20/10.000 patient days; 42,5% of patients had previous admission to hospital in last 4 weeks and 74% came from home. For HA CDI, median interval to diagnosis was 10,7 days. According to ECDC definition 80,8% of cases (97/120) were HA CDI , 16,7% CA-CDI, 2,5% indeterminate. Among the HA-CDI onset of symptoms was in the community in 27 cases, for 70 cases onset was in hospital. Using the classification based on a 4 day cut-off, HA CDI drop to 57 (47,5%) and not hospital related CDI increase to 63 (52,5 %). Lacking 13 CDI cases developed at 3 and 4 day and can be probably imported from community.

**Conclusion:** Our study showed variability of CDI in terms of characteristics, origin, incubation time. This study applied the 4 days cut-off classification, showing that half of the HA cases developed very late in the hospitalization. More analysis are required to verify if these cases classified as hospitals cases were rather already colonized and coming from the community.

**References**

**Disclosure of Interest**: None declared

## Poster session: Influenza 1

### P41 DOES INTRODUCTION OF A RAPID INFLUENZA PCR IMPROVE PATIENT CARE OF ACUTE HOSPITALIZED PATIENTS DUE TO INFLUENZA LIKE ILLNESS?

#### M. Boonstra^1^, O. Pontesilli^1^, P. Smit^1^, T. van der Graaf^2^, R. El Moussaoui^3^, C. van Noord^3^, M. Damen^1^

##### ^1^Medical Microbiology and Infection Prevention; ^2^Business Intelligence; ^3^Internal Medicine, Maasstad Hospital, Rotterdam, Netherlands

###### **Correspondence:** M. Boonstra

**Introduction:** In our hospital contact-droplet isolation should be applied to adult patients with influenza (flu) and influenza like symptoms (ILS) of unknown cause. During the flu season isolation beds are scarce and acceleration of diagnosis and patient flow is preferred.

**Objectives:** Objectives were (1) to determine if introduction of a rapid polymerase chain reaction (PCR) for flu virus (Xpert Flu) would reduce isolation days per flu negative patient with ILS by comparing flu season 2017-2018 to 2018-2019,(2) the number of patients admitted to correct wards (pulmonary/internal medicine) and (3) frequency of reassignment to different wards/rooms.

**Methods:** Adult patients who underwent a flu test were included. During December 2017-April 2018 (season 1, S1) an in-house viral respiratory PCR panel, including flu A/B, was performed (24-48 hours). In December 2018-April 2019 (season 2, S2) the Xpert Flu was performed (2 hours). Information on hospital admittance and duration of isolation per patient, based on labelling of patients was retrieved from the electronic patient record (EPR).

**Results:** 429 (S1) and 478 (S2) patients were included in the analysis. Comparing S1 and S2: 70% (100/142) and 72% (96/133) flu positive admitted patients were labelled for isolation, mean isolation duration was 83 hours (±14,8 95% confidence interval (CI)) and 129 hours (±64,2 95% CI), one flu negative patient and zero patients were labelled for isolation, 64% (239/374) and 70% (374/408) were admitted to the correct ward, and the mean frequency of bed reassignment per patient was 0.54 (±0.08 95% CI) and 0.46 (±0.07 95% CI).

**Conclusion:** The effect on isolation could not be determined because labels were incorrectly placed in the EPR, therefore not accessible for automatic analysis. Frequency of admittance to correct wards, and mean frequency of reassignment to different wards tended to be improved in S2, however not significantly. Overall, patient care seems to be improved in the rapid-PCR season, however data are biased by differences in influenza prevalence and other improvements in patient care, moreover isolation should be registered differently in order to evaluate quality of care.

**Disclosure of Interest**: None declared

### P42 INFLUENZA IMMUNIZATION: KNOWLEDGE AND ATTITUDES AMONG OFFICE BASED PRACTITIONERS IN PARIS AREA IN 2018/2019

#### A. DESLANDES, E. SERINGE, K. LEBASCLE, P. ASTAGNEAU

##### CPIAS ILE-DE-FRANCE, PARIS, France

###### **Correspondence:** A. DESLANDES

**Introduction:** Influenza virus is a major public health concern, causing high morbidity and mortality every year. As virus transmission could be mediated by healthcare workers (HCW), their vaccination remains the main prevention measure. In France, despite recommendations to be vaccinated annually, the vaccination rate of HCW remains poor in healthcare facilities (around 25%). However, few data are available in office based practitioners (OBP).

**Objectives:** To assess vaccine coverage among OBP and identify barriers to vaccination.

To evaluate knowledge and attitudes regarding influenza transmission and preventing methods.

**Methods:** A descriptive study was performed during the 2018-2019 flu epidemic based on an online questionnaire sent to office based physicians, midwives, nurses and physiotherapists in Paris area. They were asked to report their influenza immunization status and reasons for accepting or refusing vaccination. The survey also assessed knowledge and attitudes regarding influenza virus transmission and prevention methods (masks and vaccination).

**Results:** A total of 556 practitioners completed the survey, mostly nurses (56%) and physiotherapists (43%). Neither physicians nor midwives responded to the study. The overall reported influenza vaccination coverage (95% CI) was 63% [59.3-67.5]. The main reasons for being vaccinated were to protect themselves (51%) and to protect patients (37%). Among HCW refusing influenza vaccination (n=182), the 3 most common reasons were: using other prevention methods (37%), doubts regarding the efficacy of influenza vaccination (30%) and opposition to influenza vaccination (30%).

HCW refusing vaccination had more misconceptions about the vaccine. Only 59% of them knew that the vaccine was not able to transmit the flu whereas 79% of vaccinated people knew that fact (p<0.0001).

**Conclusion:** The influenza immunization coverage of OBP is higher than those reported in healthcare facilities.

However, barriers against vaccination remain important issues, especially misconceptions regarding the vaccine efficacy or safety. Addressing these knowledge gaps in both initial formation and continuing medical education of HCW could lead to better protection and reduced morbidity among patients.

**Disclosure of Interest**: None declared

### P43 HOW TO ACHIEVE 80% INFLUENZA VACCINATION COVERAGE IN A LARGE NORWEGIAN UNIVERSITY HOSPITAL

#### M. E. Gjerde, K. S. Kilhus

##### Department of Research and Development, Division of Patient safety and Infection Control, HELSE BERGEN, Bergen, Norway

###### **Correspondence:** M. E. Gjerde

**Introduction:** The World Health organization recommend 75% influenza vaccination coverage among healthcare workers (HCWs). In 2017, the total vaccination coverage among HCWs in Norway was approx. 17%. In 2018, Norwegian health authorities followed up by explicitly stating the objective of 75% coverage among HCW. We describe the strategy accomplishing 80% vaccination coverage among all employees in a Norwegian University Hospital with 12 000 employees.

**Objectives:** Achieve and sustain 75% influenza vaccination coverage as a patient safety measure.

**Methods:** The last 20 years seasonal influenza vaccine has been offered free of charge to employees. In 2015, the Hospital Management and Infection Control team introduced an Infection Control program using Quality Improvement methodology. The main objective was to empower leaders to take responsibility and put infection control on the daily agenda by setting local goals. The Infection Control team created a toolkit and offered to facilitate departments. Measures to improve vaccination coverage are management commitment, education, vaccination availability, communication strategies and comparing results within the hospital. The national objective enabled hospital management to set an explicit goal for vaccination coverage stimulating competition within and between hospitals.

**Results:** The vaccination coverage among employees in Haukeland University hospital was 19% from 2006-2014. After introducing the Infection Control program in 2015, the coverage reached 41%. The health authorities introduced an explicit objective 2018, contributing to an all-time high of 80% vaccination coverage.

**Conclusion:** Introducing an Infection Control program ensuring patient safety, continuous quality improvement and management commitment is an important foundation for achieving increased coverage. The introduction of an explicit national objective was a necessary measure to be able to achieve satisfying results.

**Disclosure of Interest**: None declared

### P44 RELENTLESS EDUCATION EFFORTS TO COUNTER ‘BELIEFS’ ASSOCIATED WITH UPTAKE OF STAFF FLU VACCINATIONS

#### H. K. Lam^1^, K. Rajwinder^2^

##### ^1^Clinical Operations, Matilda International Hospital, Hong Kong; ^2^41 mount kellett road, matilda international hospital, the peak, Hong Kong

###### **Correspondence:** H. K. Lam

**Introduction:** It is common knowledge that influenza can kill and the best way to prevent its spread is through vaccination. However, it is often noted that “belief” which exists around the effectiveness and side effects of flu vaccines hinders uptake by healthcare workers. Even though education is delivered year on year to counter these “beliefs”, however, health promotion conducted during non-stormy waters do not impact or change perspectives.

**Objectives:** In order to further increase uptake for this year, various measures over the past year were designed to examine the real reasons behind staff’s refusal of vaccination, with the ultimate aim of steamrolling the reasons to increase uptake.

**Methods:** At the kick-off of the 2017/18 Staff Flu Vaccination Campaign, the infection control team(ICT) created various educational opportunities encompassing booth games in the staff canteen supplemented by on-site education followed by interviews of staff by means of using iPads. A survey was designed to be offered in both English and Chinese and was launched online and staff who refused to receive a flu jab during the 2017/18 campaign were invited to complete it.

**Results:** 72 submissions with 7% claiming that they did not know where to receive a jab. In examining the “beliefs”, 35% answered “others” as reasons for refusing which included “no time to get a jab”, “being sick at that moment” or “the lack of vaccine stock during that particular period”. 20% indicated “a fear of side effects”. Same number of staff (18%) claimed “the fear of needles” and “ineffectiveness of vaccine demonstrated by past experience”.

**Conclusion:** With all feedbacks attained over the year, ICT concluded that the main reason of refusal was because of a lack of knowledge on influenza. Thus to kick start the 2018/19 campaign, a microbiologist was invited to give an educational talk on influenza to counter “beliefs” in relation to side effects, effectiveness etc. were all addressed supported by scientific data and etiology. Learning from past experience, more stock of vaccines was purchased to prevent insufficient supply. To allow more opportunities to receive a jab, ICT set up a vaccination booth in staff canteen and provided on-site injection in a bi-weekly basis. As a result of these series of improvement strategies, the overall staff flu vaccination compliance rate increased steadily to 60% in 2018/19.

**Disclosure of Interest**: None declared

### P45 INFLUENZA IMMUNIZATION : HOW TO ENCOURAGE HEALTHCARE WORKERS TO GET VACCINATED ?

#### S. CYRILLE, E. SERINGE, K. LEBASCLE, P. ASTAGNEAU

##### CPIAS ILE-DE-FRANCE, PARIS, France

###### **Correspondence:** S. CYRILLE

**Introduction:** Influenza vaccination is important to prevent influenza transmission and associated morbidity among patients in all healthcare facilities (HCF). Despite French recommendations for all healthcare workers (HCW) to be vaccinated annually against influenza, the vaccination rate is suboptimal.

**Objectives:** To assess vaccine coverage among HCW and identify barriers to vaccination in order to promote specific measures that could improve vaccination coverage.

**Methods:** A descriptive study was conducted during the 2018-2019 flu epidemic based on an online questionnaire sent to all hospital infection control teams in Paris area. Participants were asked to report their HCW influenza immunization coverage and select potential barriers of influenza vaccination as identified from the literature. They were also invited to assess the effectiveness of various strategies known to improve influenza vaccination compliance.

**Results:** Overall, 35% (*N*=141) of healthcare facilities responded to the study. The overall influenza vaccination coverage (95% CI) was reported to be 24.4% [22.2-26.6] including 43.5% [38.5-48.5] for physicians and midwives, and 22.6% [19.9-25.3] for nurses and nursing assistants. Low compliance with seasonal influenza vaccination among HCW was primarily attributed to doubts regarding the efficacy of influenza vaccination (86%), followed by concerns regarding vaccine safety (66%) and social media influence (45%) (*n*=141). The most effective strategies proposed to increase influenza vaccination uptake were 1) being vaccinated directly in the hospital wards by a colleague (*n*=119; 79%), 2) use a mobile vaccination cart (*n*=79; 78%), 3) set up a vaccination stand (*n*=62; 61%).

**Conclusion:** Although slightly in progress as compared to previous years, **t**he influenza immunization coverage among HCW remains below expectation in these healthcare settings. The identification of barriers suggests the need for better information on flu vaccine towards healthcare professionals in addition to implementation of active vaccination campaign.

**Disclosure of Interest**: None declared

### P46 STAFF INFLUENZA VACCINATION MEETING THE JOINT COMMISSION REQUIREMENTS OF 90% BY 2020

#### A. Dababneh^1^, S. Bowman ^2^, K. Dixon ^3^, C. McHenry ^2^, M. Moran ^3^, J. Rybarczyk^4^, S. spah^2^, J. streets Benningfield ^3^, R. Varathraj Palraj ^1^, C. Wilker ^5^, B. Withers ^2^

##### ^1^Infectious diseases, Mayo Clinic, Rochester; ^2^occupational health; ^3^IPAC , MCHS La Crosse , La Crosse; ^4^IPAC , MCHS Sparta , Sparta; ^5^Internal medicine , MCHS La Crosse , La Crosse , United States

###### **Correspondence:** A. Dababneh

**Introduction:** Influenza vaccination for health care workers on an annual basis is currently recommended by CDC, the Advisory Committee on Immunization Practices (ACIP), and the Healthcare Infection Control Practices Advisory Committee (HICPAC) recommend that all U.S.. Joint commission has set a goal of achieving 90 % vaccination by 2020^1^. At Mayo Clinic Health System in La Crosse WI vaccination rates for 2015 influenza season was 86.9%.

**Objectives:** improve staff influenza vaccination rates to above 90% and sustain it.

**Methods:** A multidisciplinary team approach was developed to promote influenza vaccination as a Patient Safety initiative. Compliance with influenza program included vaccination, provide proof of medical contraindication from a Medical Provider, provide proof of vaccination at another location, or declination. Influenza vaccine clinic was made available. Departments were allowed to administer vaccine to staff, egg free vaccine was made available, promotional campaign was implemented, email updates and letters of non-compliant staff. Attendance of a physician led face to face education was mandatory prior to declination. Questionnaire was provided to employees before and after the lecture to asses barriers to vaccination.

**Results:** By the compliance due date, January 11, 2017 the 2016-2017 influenza season staff vaccination, vaccination rate was 91.7% 3,128/3,412. 5.9 % improvement compared to 2015-2016 influenza season vaccination rate . 239 questionnaires were completed. 47.3% had patient care responsibilities,52 % reporting prior flu like illness. 89.1% found the educational component informative. 67.2% believe that the vaccine is safe. 41.1% thought that the influenza vaccine was effective. 29.4% have objections to vaccines in general and 1.3% changed their mind after hearing the lecture.

**Conclusion:** mulideisicplinary team approach with education for influenza vaccine declination is a useful tool to improve vaccination rates among hospital employees

**References**

1. Strengthened stardards onf flu vaccinations to pressure hospitals to progress. ED management 2012 Mar 24 : SUPPL 1-3

**Disclosure of Interest**: None declared

### P47 GIVING HEALTHCARE WORKERS (HCW) A CHOICE BETWEEN VACCINATION AND WEARING A MASK DURING SEASONAL INFLUENZA EPIDEMIC: A POSSIBLE STRATEGY TO INCREASE VACCINATION COVERAGE

#### A. Iten^1^, C. Bonfillon^2^, C.-A. Siegrist^3^, D. Pittet^1^

##### ^1^Infection Control Program; ^2^Hospital Health Service; ^3^Pediatrics, HUG, Geneva, Switzerland

###### **Correspondence:** A. Iten

**Introduction:** Vaccination against seasonal influenza is the cornerstone of protection for both patients and hospital staff (HCW). Various actions are recommended to increase hospital staff adherence to seasonal influenza vaccination: information campaigns, management support, and vaccination available directly in patient care wards, day and night, at no cost. Despite the implementation of these measures, the vaccination rate of our institution remained stable at around 24% for a decade. As Swiss law does not allow vaccination to be mandatory, an alternative solution was tried: the obligation for staff to choose between vaccine and wear of a mask during the epidemic period.

**Objectives:** To describe the evolution of the vaccination rate among HCW, following the introduction of a strategy that consists in choosing between vaccination and the wear of a mask during the epidemic period.

**Methods:** The vaccination campaign takes place every year from late October to mid-December, with the option for the hospital staff vaccinated until the peak of the influenza epidemic. Vaccination is offered by dedicated and trained staff. Personal information and hospital sector of the vaccinated persons are systematically collected.

**Results:** From October 2011 to February 2019, seasonal influenza vaccination was offered to 91,181 people. During this period, the averaged vaccination rate increased by 9.7% for all staff (season 2011/2012: 29.3%; season 2018/2019: 39.0%), by 11.6% for medical staff and by 6.2% for the rest of staff. The champions of this evolution were doctors (+11.6%) and social workers (+11.5%). This increase differs from one sector to another: max. +18.5% for the gynaecology/obstetrics department; min. +3.9% for the operations department.

**Conclusion:** The requirement to choose between the vaccine and wearing a mask during an epidemic increases adherence to influenza vaccination. However, the effect of this measure is inhomogeneous: a detailed analysis of the results is necessary to plan additional measures.

**Disclosure of Interest**: None declared

### P48 EVALUATION OF A MULTIMODAL STRATEGY TO PROMOTE INFLUENZA VACCINATION OF HEALTHCARE WORKERS

#### P. Bressin, L. Senn, P. Genoud, D. Christen, M. Cote, J. Scheurer, G. Zanetti, C. Lazor Blanchet

##### CHUV - centre hospitalier universitaire vaudois, Lausanne, Switzerland

###### **Correspondence:** L. Senn

**Introduction:** Influenza vaccination of healthcare workers (HCWs) remains one major preventive measure against nosocomial Influenza. However, many HCWs still decline vaccination. Our University Hospital have implemented an active promotion of this vaccination.

**Objectives:** To evaluate the impact of a multimodal strategy to promote Influenza vaccination in HCWs.

**Methods:** A multimodal strategy has been implemented in 2015. It was based on i) posters throughout the hospital, ii) a ‘treasure hunt’ (stickers with QR codes opening short videos) and iii) messages on intranet and in internal newsletters. Access to the vaccine has been facilitated through several vaccination points located at strategic places (changing rooms, hospital entrances, cafeterias, etc.), and the deployment of ‘vaccine delegates’ in each ward (n = 100). The number of vaccines administrated was collected to calculate the vaccination coverage (VC). During the Influenza season, an institutional directive obligates to wear a mask by all unvaccinated HCWs in contact with patients. The evolution of VC and the proportion of putative nosocomial influenza cases were monitored.

**Results:** Between the 2011-12 and 2018-19 Influenza seasons, the VC of HCWs in contact with patients increased from 29.5% to 50% (p<10-6), in both nurses (+75%) and physicians (+57%). Physicians’VC remained higher than nurses’VC (respectively 55% vs. 42%). The proportion of vaccinations carried out by vaccine delegates was 36% in 2018-19. The proportion of nosocomial Influenza was 22% and 18% respectively over the last two seasons. The campaign tools were shared with partner hospitals.

**Conclusion:** Since the implementation of this multimodal strategy to promote Influenza vaccination, we observed a significant increase in VC. Strong support from the hospital direction seems to have been a factor of success. This experience reinforces the interest for a multimodal strategy.

**Disclosure of Interest**: None declared

## Poster session: Epidemiology of Carbapenemases

### P49 INCIDENCE OF MULTIDRUG-RESISTANT, EXTENSIVELY DRUG-RESISTANT AND PANDRUG-RESISTANT GRAM-NEGATIVE BACTERIA IN BRAZILIAN INTENSIVE CARE UNITS

#### G. L. E. Souza^1^, R. F. de Andrade Rocha^1^, A. D. N. Silveira^1^, H. D. D. D. Carvalho^1^, C. D. D. M. Oliveira^1^, L. G. Giarolla^1^, E. M. M. Leite^2^, E. U. Silva^3^, B. R. G. M. Couto^4^, C. E. F. Starling^5^, on behalf of NOIS Project

##### ^1^Instituto de Ciências Biológicas e da Saúde, Centro Universitário de Belo Horizonte; ^2^Risoleta Tolentino Neves Hospital; ^3^Madre Teresa Hospital; ^4^Avenida Professor Mario Werneck, 1685, Centro Universitário de Belo Horizonte; ^5^Lifecenter Hospital, Belo Horizonte, Brazil

###### **Correspondence:** G. L. E. Souza

**Introduction:** Understanding the incidence of multidrug-resistant (MDR), extensively drug-resistant (XDR) and pandrug-resistant (PDR) can ultimately result in accurate treatment of healthcare associated infections (HAI) in intensive care units (ICU's).

**Objectives:** This research provides a public report, and benchmarks for the resistance rate of gram-negative MDR, XDR and PDR bacteria causing HAI's in 12 ICU's from Belo Horizonte, a brazillian city with aproximately 2,5 million inhabitants.

**Methods:** Data was collected between Jan/2013 to Dec/2017. All hospitals complied with ECDC HAI surveillance protocols. Antimicrobial resistance from 6 gram-negatives were evaluated: *Acinetobacter sp., Klebsiella sp., Proteus sp., Enterobacter sp., Escherichia coli,* and *Pseudomonas sp..* Benchmarks were defined as p10, p20, p50 and p70 percentiles for total multi-resistance rate, or TMR (MDR+XDR+PDR = TMR).

**Results:** A total of 6,242 strains were tested: no PDR were found. *Acinetobacter sp.*: 1432 out of 1858 (77%) were MDR, and 206 (11%) were XDR. *Klebsiella sp.*: 813/1.566 (52%) MDR; 2/1566 (0,1%) XDR. *Proteus sp.*: 163/507 (32%) MDR; 0/507 (0%) XDR. *Enterobacter sp.*: 148/471 (31%) MDR; 0/471 (0%) XDR. *Escherichia coli*: 0/681 = 157/681 (23%) MDR; (0%) XDR. *Pseudomonas sp.*: 180/1159 (15%) MDR; 41/1159 (3%) XDR. Benchmarks: *Acinetobacter sp.* [74, 82, 91.5, 93]. *Klebsiella sp.* [31,39, 53.5, 62]. *Proteus sp.* [17,18, 28.5, 33]. *Enterobacter sp.* [16, 19, 30, 42]. *E. coli* [13, 13, 22.5, 29]. *Pseudomonas sp.* [7, 7, 18.5, 26].

**Conclusion:** *Acinetobacter sp.* presented highest prevalence, and a TMR of 88%. *Pseudomonas sp.* was the 3^rd^ most prevalent strain, but had the lowest TMR and second highest XDR rate. Benchmarks can be used for healthcare quality assessment.

**Disclosure of Interest**: None declared

### P50 PREVALENCE OF ESBL AND MBL ENCODING GENES IN ACINETOBACTER BAUMANNII STRAINS ISOLATED FROM PATIENTS OF INTENSIVE CARE UNITS IN KURDISTAN, IRAQ

#### S. T. Baban, P. Akram, D. Jalal

##### Infection control and prevention, Surgical Specality Hospital, Erbil, Iraq

###### **Correspondence:** S. T. Baban

Antibiotic resistance in Acinetobacter spp., particularly Acinetobacter baumannii, is increasing rapidly. A. baumannii possesses two intrinsic b-lactamase genes, in addition to weak permeability and efflux systems, that together confer a natural reduced susceptibility to antibiotics. The aim of this study was to investigate the prevalence of ESBL and MBL encoding genes among *A. baumannii* isolates. In this study, 300 *A. baumannii* strains were isolated from ICU wards of three hospitals of Erbil City, Iraq in 2019. Phenotypic identification of the production of ESBLs and MBLs has been carried out by using DDST methods. PCR technique was used for amplification of the ESBL and MBL encoding genes, namely: CTX-M, SHV, TEM, OXA-51, VIM-Family, IMP-Family, SPM-1, SIM-1, and GIM-1. Eighty seven (87%), 95 (95%), 98 (98%) and 95 (95%) out of 100 *A. baumannii* isolates were resistant to imipenem, meropenem, ceftazidime and cefotaxime, respectively. Also, 99% and 7% of the isolates were MBLs and ESBLs produced phenotypically. Ninety (30%), 60 (20%) and 180 (60%) out of 100 *A. baumannii* isolates have been confirmed to harbor the *bla*_VIM_-family, TEM and SHV genes, respectively. Our results show no significant relationship between the detected gens with production of MBLs and ESBLs in spite of high prevalence of MBL encoding and drug resistant *A. baumannii*. Probably some other genes rather than what we studied are involved in phenotypic production of MBLs and ESBLs and subsequent drug resistance in Kurdistan area, Iraq.

**Disclosure of Interest**: S. Baban Employee of: No conflict of interest, Grant/Research support from: No conflict of interest, Speaker's bureau of: No conflict of interest, Shareholder of: No conflict of interest, Consultant for: No conflict of interest, Paid instructor for: No conflict of interest, Other conflict with: No conflict of interest, P. Akram: None declared, D. Jalal: None declared

### P51 RESISTANCE TO THIRD GENERATION CEPHALOSPORIN DUE TO TEM AND CTX-M-1 TYPE EXTENDED-SPECTRUM Β-LACTAMASE GENES AMONG CLINICAL ISOLATES OF GRAM-NEGATIVE BACILLI IN ASELLA, CENTRAL ETHIOPIA

#### T. B. Tufa^1,2^, F. André ^1,2^, S. Abdissa^1,2^, K. Achim ^3^, M. Colin ^3^, P. Klaus ^3^, F. Torsten ^1,2^, H. Dieter ^1,2^

##### ^1^DGHID, University Hospital, Düsseldorf, Germany; ^2^Hirsch Institute of Tropical Medicine, Asella, Ethiopia; ^3^Institute of Medical Microbiology and Hospital Hygiene, Düsseldorf, Germany

###### **Correspondence:** T. B. Tufa

**Introduction:** Lack of local data concerning causative pathogens and resistance patterns results in suboptimal empirical treatment and unfavorable clinical outcomes.

**Objectives:** to characterize antimicrobial resistance patterns of Gram-negative bacteria isolated from hospitalized patients with febrile infections in Central Ethiopia.

**Methods:** In total, 684 Patients ≥1 year of age admitted to the Asella Teaching Hospital with fever from April 2016 to June 2018 were included. Blood and other appropriate clinical specimens were cultured. Susceptibility testing was performed using the Kirby-Bauer method and VITEK2. Confirmation of species identification and identification of resistance-genes were conducted using MALDI-ToF-MS and PCR at a microbiology laboratory in Düsseldorf, Germany.

**Results:** In total, 684 study participants were included; 54% were male and the mean age was 26.7 years. At sample collection, 57.2% (391/684) patients received antibiotic therapy. Ceftriaxone was the most commonly prescribed antibiotic (78%).

The overall culture positivity rate was 7.5%. Of 83 cultured organisms, 38(46%) were Gram-negative, 43(52%) Gram-positive and 2(2%) Candida species. Among the 38 Gram-negative isolates, 16 (42%) were *E. coli*, 15 (39%) *K. pneumoniae* and 4 (11%) *P. aeruginosa*. Resistance against commonly used antibiotics for Gram-negative bacteria at the study site was: piperacillin/tazobactam 48%(13), ampicillin/sulbactam 93%(25), cefotaxime 89%(24), ceftazidime 74%(20), cefipime74%(20), and amikacin 4%(1). Of 27 Gram-negative bacteria available for resistance-gene detection, *bla*_NDM-1_ was detected in one *K. pneumoniae* isolate and *bla*_NDM-1_ plus *bla*_OXA-51_ in *A. baumannii.* 81% (22/27) of the Gram-negative rods were confirmed to contain ESBL-genes as follows: TEM 17 (77%), CTX-M-1-group 15(68%), and SHV-6(27%).

**Conclusion:** We found a high prevalence (81%) of ESBL-producing bacteria and 7.4% carbapenem-resistance. The 3rd generation cephalosporin were the most prescribed drugs at the study site. Strengthening of antimicrobial stewardship programs is required in order to face the threat of multidrug-resistant bacteria.

**Disclosure of Interest**: None declared

### P52 BURDEN OF A ST258 KPC-KLEBSIELLA PNEUMONIAE CLONE HARBOURING KPC-2 AND RMTB METHYLASE IN A BRAZILIAN TERTIARY CENTRE

#### D. O. Andrey^1,2^, P. Dantas^3^, W. B. Brasileiro Martins^4^, K. Sands^2^, E. Portal^2^, T. R. Walsh^2^, E. A. Medeiros^3^, A. C. Gales^4^

##### ^1^Infectious Diseases Division, Geneva University Hospitals and Medical School, Geneva, Switzerland; ^2^School of Medicine, Cardiff University, Cardiff, United Kingdom; ^3^Infection Control; ^4^Alerta Lab, Division of infectious Diseases Escola Paulista de Medicina, Hospital Sao Paulo, UNIFESP, Sao Paulo, Brazil

###### **Correspondence:** D. O. Andrey

**Introduction:** KPC-*K. pneumoniae* (KPC-KP) infections are challenging, and not least in middle-income countries, where novel b-lactams/b-lactamase inhibitors are often not available. Remaining options are often restricted to "old antibiotics" such as aminoglycosides and polymyxins.

**Objectives:** Hospital São Paulo (HSP) is a Brazilian teaching hospital, where KPC-KP are endemic. The role of aminoglycoside resistance in bloodstream infections (BSI) due to KPC-KP was examined.

**Methods:** This is a subanalysis within a retrospective clinical-microbiological study of 125 BSI due to KPC-KP during 2014-16 at HSP (one isolate per BSI). AST was determined by agar dilution (EUCAST interpretation). BSI cases caused by KPC-KP non-susceptible (NS) to aminoglycoside (both amikacin and gentamicin) were compared to the remaining cases. PFGE of all isolates and WGS (Illumina, MinIon long-reads) of a subset of isolates were carried out. Clinical data was compared between both groups.

**Results:** Forty-nine (39%) of 125 KPC-KP isolates were amikacin and gentamicin NS (>128mg/L MIC for both in 45 cases). Among NS isolates, PFGE analysis coupled with *in silico* MLST identified 40 isolates belonging to ST258, 6 to ST11, 2 to ST16 and one to ST437. Among those, a subset of 31 were sequenced showing 84% positivity for *rmtB* 16S-rRNA methylase gene (ST258 isolates N=24, ST11 N=1 and ST16 N=1). No other 16S-rRNA methylase was identified. Genomic assembly of one ST258, one ST11, and one ST16 isolate showed highly similar 177 kb IncA/C2 plasmids, harbouring *rmtB*. Outcome analysis showed 65% all-cause 30-day mortality for the cohort, 61% in the aminoglycoside-NS group vs 66% in the aminoglycoside susceptible group (*p*=0.6).

**Conclusion:** We report the burden and clonal spread of a KPC-2 *rmtB+* ST258 KP clone in a tertiary hospital, and in two cases probable horizontal acquisition of an IncA/C2 plasmid by ST11 and ST16 KPC-KP isolates. Although we did not identify an impact on mortality, the dissemination of 16S-rRNA methylase *rmtB* plasmids in an endemic setting for KPC-producing CC258 clones, providing resistance to most available aminoglycosides including new therapeutic options as plazomicin, is worrisome.

**Disclosure of Interest**: None declared

### P53 THE EMERGENCE OF BLANDM-5 POSITIVE ESCHERICHIA COLI IN A BANGLADESHI HOSPITAL: LINKAGE BETWEEN CLINICAL INFECTIONS AND FAECAL CARRIAGE

#### R. Farzana^1^, L. S. Jones^2^, M. A. Rahman^3^, K. Sands^4^, E. Portal^1^, I. Boostrom^1^, B. Hasan^1^, T. R. Walsh^1^

##### ^1^Cardiff University; ^2^Public Health Wales, Cardiff, United Kingdom; ^3^Dhaka Medical College, Dhaka; ^4^Cardiff University, Cardiff, Bangladesh

###### **Correspondence:** R. Farzana

**Introduction:** Carbapenem resistance has become global concern, and in particular *bla*_NDM-5_ which is emerging worldwide.

**Objectives:** To investigate the epidemiology and transmission dynamics of *bla*_NDM-5_ positive *Escherichia coli* in Bangladeshi hospital

**Methods:** We performed this study at Dhaka Medical College Hospital (DMCH), comprised of 700 rectal swabs from inpatients and OPD during 13.05.17 to 17.07.18 and clinical samples between 20.10.16 and 31.07.17. Microbiology was undertaken at Cardiff University and included MALDI-TOF, agar dilution MIC, illumina MiSeq and S1 PFGE with *bla*_NDM_ probing.

**Results:** Rectal swabs yielded 457 single growth (65.3%), 147 mixed growth (21%) and 96 no growth (13.7%), of which the proportion of *E. coli* was 41.7%. *E. coli* (n=292) showed resistance to antimicrobials: amoxiclav (97.9%), piperacillin-tazobactam (86%), ceftriaxone (96.6%), ceftazidime (96.6%), cefotaxime (95.9%), cefepime (93.2%), ciprofloxacin (91.4%), levofloxacin (88%), co-trimoxazole (82.2%), meropenem (71.6%), imipenem (67.9%), gentamicin (58.5%), amikacin (50%), fosfomycin (1%) and colistin (0.3%). Faecal carriage of *bla*_NDM_ was 28.3% (198/700); *bla*_NDM_ variants were *bla*_NDM-5_ (n=165), *bla*_NDM-1_ (n=17), *bla*_NDM-7_ (n=13) and *bla*_NDM-4_ (n=3). Patients visited to OPD had no history of antibiotic intake during sample collection, however, 88.7% (340/383) of inpatients were on antibiotics. *E. coli* harbouring *bla*_NDM-5_ was isolated from inpatients more than outpatients (*p*<0.1). There was a significant association in burn patients (*p*<0.00001). We observe a high prevalence of *bla*_NDM-5_ (38/223, 17.4%) among clinical *E. coli*. Faecal NDM-5 positive *E. coli* was distributed in 28 different sequence types (STs); the majority belonged to ST167 (n=38) followed by ST405 (n=18). There was association between faecal NDM-5 positive *E. coli* and ST167 (*p*<0.01). Similarly, clinical NDM-5 positive *E. coli* were significantly related to ST167 (*p*<0.001). We did not observe any relation between plasmids of specific size and clonal type.

**Conclusion:** Gut colonization of *bla*_NDM-5_ is a reservoir for clinical infections by MDR bacteria via horizontal transfer in selective antibiotic pressure along with clonal spread.

**Disclosure of Interest**: None declared

### P54 MOLECULAR CHARACTERIZATION OF CARBAPENEM RESISTANT KLEBSIELLA PNEUMONIAE ISOLATES COLLECTED FROM A CENTRAL PUBLIC HOSPITAL IN DURBAN, SOUTH AFRICA BETWEEN 2016 AND 2017

#### Y. Ramsamy^1,2,3^, K. P. Mlisana^4^, M. Allam ^5^, D. Amoako^6^, A. Ismail^7^, A. Akebe^6^, S. Y. Essack^6^

##### ^1^Antimicrobial Research Unit , KwaZulu Natal; ^2^Medical Microbiology , National Health Laboratory Services; ^3^Medical Microbiology , University of KwaZulu Natal , Durban; ^4^Medical Microbiology , National Health Laboratory Services, University of KwaZulu Natal, Johannesburg; ^5^Sequencing Core Facility, National Institute for Communicable Diseases, Sandringham; ^6^Antimicrobial Research Unit , University of KwaZulu Natal , Durban; ^7^Sequencing Core Facility, , National Institute for Communicable Diseases, Sandringham , South Africa

###### **Correspondence:** Y. Ramsamy

**Introduction:** Ongoing dissemination of carbapenemase-producing *Enterobacteriaceae* (CPE) represents a public health issue. Early detection and infection prevention and control strategies are key in limiting spread of CPE within healthcare systems.

**Objectives:** The study aimed to investigate the genomic epidemiology and clonal relationships among the carbapenem-resistant *K. pneumoniae* isolated. This is important in evaluating the effectiveness of infection prevention and control programs and provides information for the development of new antimicrobial targets.

**Methods:** Between May 2016 and May 2017, 10 isolates(5 rectal swabs and 5 blood stream infections) were obtained from patients admitted to intensive care unit in Durban, South Africa.Following phenotypic microbial identification and antibiotic susceptibility tests,isolates were subjected to whole genome sequencing.

**Results:** All isolates were extensively drug-resistant with detectable phenotypic and genotypic resistance to tested β-lactams. Resistance to carbapenems were conferred by NDM-1 mediated by the acquisition the p18-43_01-like multi-replicon [ColRNAI, IncFIB(pB171), Col440I, IncFII, IncFIB(K) and IncFII(Yp)] plasmids. Interestingly, all the ten isolates had the same plasmid multilocus sequence type (IncF[K12:A-:B36]) and capsular serotype KL149, affirming the epidemiological linkage between the *K. pneumoniae* clones. All but one isolate belonged to ST152 sequence type. A novel sequence type, ST3136 differed from the primary clone by a single-locus variant in the *rpob* allele. Findings suggest an independent plasmid acquisition followed by local dissemination revealing horizontal spread of this blaNDM-1-bearing plasmid structure.

**Conclusion:** The acquisition of resistance-encoding plasmids, horizontal transfer and clonal dissemination facilitate the spread of carbapenemase genes in a public hospital. The valuable information provides a better understanding of the molecular mechanisms and spread of drug-resistant strains within healthcare settings

**References**

**Disclosure of Interest**: None declared

### P55 ACTIVE SURVEILLANCE CULTURE OF CARBAPENEM-RESISTANT KLEBSIELLA PNEUMONIAE AND CARBAPENEM-RESISTANT ACINETOBACTER BAUMANNII IN AN EMERGENCY ICU IN A TEACHING HOSPITAL IN CHINA

#### F. Qiao, W. Huang

##### Infection Control Department, WEST CHINA HOSPITAL,SICHUAN UNIVERSITY, Chengdu, China

###### **Correspondence:** F. Qiao

**Introduction:** Carbapenem-resistant *Klebsiella pneumoniae (*CRKP*) and* carbapenem-resistant *Acinetobacter baumannii* (CRAB) represent an urgent health concern in China.

**Objectives:** To understand the prevalence and risk factors associated with rectal CRKP and CRAB colonization among emergency ICU patients on admission and their infection during their stay in mainland China.

**Methods:** A prospective observational study was performed in an 18-bed emergency ICU in a teaching hospital in China in 2018. Rectal swabs for CRKP and CRAB detection were obtained on emergency ICU admission.

**Results:** During the study period, 531 patients were screened with rectal swab, and the screening rate was 81.94%. A total of 18 patients (3.39%, 95% confidence interval [CI]: 1.85%>4.93%) and 38 (7.16%, 95% confidence interval [CI]: 4.97%>9.35%) were already colonized with CRKP on EICU admission. Patients who were already colonized with CRKP on EICU admission were more likely to develop a CRKP infection compared with those without CRKP colonization at admission (20.0% vs 2.2%, RR=9.02, 95% CI: 3.58-22.76, P<0.001). While there’s no difference in CRAB infection between patients with or without CRAB colonization (26.0% vs 17.0%, RR=1.53, 95% CI: 0.91-2.57, P=0.118).

**Conclusion:** The CRKP and CRAB colonization rate among EICU patients in mainland China is very high. Patients with CRKP-positive rectal swabs were more likely to develop CRKP infections during their stay in the hospital.

**Disclosure of Interest**: None declared

### P56 DETECTION OF CARBAPENEMASE-PRODUCING GRAM-NEGATIVE BACTERIA FROM HOSPITAL ENVIRONMENT IN SLOVAKIA: THREE-YEARS MULTICENTRE STUDY HOSPITAL-ENVIRO-REZ

#### L. Michalikova^1,2,3^, S. Kissová^4^, L. Pazderka^1,2,3^, S. Kucharíková^1^, J. Prnová^5,6^, J. Brňová^1,3,6^

##### ^1^Department of Laboratory Medicine, TRNAVA UNIVERSITY; ^2^Department of Clinical Microbiology, Analytx s.r.o.; ^3^Centre of Microbiology and Infection Prevention, Trnava university, Trnava; ^4^Medirex , Bratislava; ^5^Department of Public Health, Trnava university; ^6^Department of Hospital Hygiene and Epidemiology, University Hospital Trnava, Trnava, Slovakia

###### **Correspondence:** L. Michalikova

**Introduction:** Hospital environments are potential reservoirs of bacteria associated with nosocomial infections. Carbapenems producing Gram-negative bacteria (CPE-GNB) can survive on inanimate surfaces for months, thus serving as a transmission source to healthcare workers and susceptible patients.

**Objectives:** In this study we assess the occurrence of carbapenems producing Gram-negative from hospital environment in Slovakia.

**Methods:** Multicentre national study was performed to monitor prevalence of carbapenemases-producing Gram-negative bacteria from January 2015 to December 2017. The bacterial ability to produce these enzymes were analysed with rapid imunochromatographic assay (NG-Test Carba 5) and with rapid molecular assay (Gene Xpert Carba-R).

**Results:** Overall 2114 samples from hospital environment (*Klebsiella* spp., *Pseudomonas* spp., *Escherichia coli*, *Enterobacter* spp., *Staphylococcus aureus* and *Enterococcus* spp.) were analysed. Of all Gram-negative environmental bacteria (n=985) were confirmed 57 (5,8%) resistant to meropenem. Isolates were further tested for ability to produce carbapenemases. Molecular testing with Gene Xpert CarbaR method identified 5 *bla*VIM possesing *Pseudomonas* spp. and 3 *bla*NDM possessing *Klebsiella pneumoniae.* Immunochromatographic method NG-Test were identified 11 carbapenemases-producers, 5 VIM producers (*Pseudomonas* spp.), 3 NDM producers (*Klebsiella pneumoniae*), and 3 IMP producers (*Pseudomonas* spp.) respectivelly. CPE-GNB isolates were detected from sink (6), manometer cuff (2), aspirator (2) and one ready to use whipes respectively.

**Conclusion:** Detection of carbapenems producing Gram-negative bacteria from various sampling sites indicated decontamination processes failures and the surfaces in the hospitals were potential exogenous sources of nosocomial infection.

**This study was supported by a research grant from the MŠVVaŠ SR.**

**Disclosure of Interest**: None declared

### P57 HIGH RATE OF EXTENDED-SPECTRUM BETA-LACTAMASE PRODUCING GRAM-NEGATIVE INFECTIONS AND ASSOCIATED MORTALITY IN ETHIOPIA: A SYSTEMATIC REVIEW AND META-ANALYSIS

#### T. B. Tufa^1,2^, T. Tufa^3^, F. André ^1,2^, K. Achim ^4^, M. Colin ^4^, F. Torsten ^1,2^, P. Klaus ^4^, H. Dieter ^1,2^

##### ^1^Hirsch Institute of Tropical Medicine, Asella, Ethiopia; ^2^DGHID, University Hospital, Düsseldorf, Germany; ^3^Addis Ababa University, Addis Ababa, Ethiopia; ^4^Institute of Medical Microbiology and Hospital Hygiene, Düsseldorf, Germany

###### **Correspondence:** T. B. Tufa

**Introduction:** Extended-spectrum beta-lactamase (ESBL)-producing Gram-negative bacteria have become a serious threat to global health. To date, regular surveillance of multidrug-resistant (MDR) pathogens is lacking in Ethiopia.

**Objectives:** To summarize and analyze published data regarding ESBL-producing bacteria in different regions of Ethiopia.

**Methods:** A systematic search was conducted on PubMed, PubMed Central, and Google Scholar until March 2019. Eligible studies were selected using the following criteria. A random effect model estimated the pooled proportion of ESBL- producing Gram-negative bacteria. The publication bias and the variation in proportion estimates attributed to heterogenicity were also assessed.

**Results:** Totally, 1782 Gram-negative bacteria isolated from 5191 clinical samples were included. The pooled proportion estimates of ESBL-producing Gram-negative was 52% (95% CI: 49–54%; P< 0.01).There was a high level of heterogeneity, random model methods (I^2^ = 95%, P <0.01). Among different species, ESBL rates were 65.7% (262) *Klebsiella* spp., 60.6% (n=20) for *Enterobacter* spp., 47.8% (n=22) for *Citrobacter* spp., 47.0% (n=383) for *E. coli*, 45.7% (n=85) for *Salmonella* spp., 28.6% (n=14) for *Proteus* spp., 16.7% (n=4) for *P. aeruginosa*, and 14.3% (n=3) for *Acinetobacter* spp. A total 81 isolates were positive for the ESBL-encoding genes: 82.7% (67/81) CTX-M-1 group, 17.3 %(14/81) *bla*_TEM_ were reported. Two studies reported mortality associated with infections by ESBL-producing pathogens or resistant to the 3rd generation cephalosporins was12 of 14 (86%) of patients infected with ESBL-producing bacteria died.

**Conclusion:** In this meta-analysis, the pooled phenotypic prevalence of ESBL-producing pathogens is high and is associated with a high mortality, however the available data is scarce. This highlights the need for establishing and upgrading clinical microbiology laboratories in the country for routine antibiotic susceptibility testing. CTX-M-1 group is the highest predominantly detected resistant genes. The capacity to detect ESBL genes is desirable for continuous surveillance of MDR.

**Disclosure of Interest**: None declared

### P58 BELGIAN ANTIMICROBIAL RESISTANCE SURVEILLANCE EXTENDED: ISOLATES FROM URINARY SAMPLES

#### L. Catteau, K. Mertens, B. Catry

##### Sciensano, Brussels, Belgium

###### **Correspondence:** L. Catteau

**Introduction:** International comparisons related to the burden of antimicrobial resistant infections requires standardization of surveillance tools. From 2017 onwards, the national EARS-Net subproject for Belgium (EARS-BE) included isolates from urine samples next to blood and cerebrospinal fluid (CSF) samples.

**Objectives:** We report here the antimicrobial resistance results for 3 major Gram-negative species and stratify the results by sample and laboratory type.

**Methods:** EARS-BE methodology is described in detail elsewhere (1). Briefly, all Belgian laboratories (hospital and non-hospital) that performed routine antimicrobial susceptibility tests (AST) in 2017 were invited to transfer their results to Sciensano. For each patient, only the 1^st^ specimen within the study year was included.

**Results:** 31 hospital labs submitted results for blood/CSF while 24 (19 hospital & 5 non-hospital labs) participated for urine samples. Within hospital labs, levels of resistance for *E. coli* isolates from urine samples were only slightly lower as compared to blood/CSF isolates. For *K. pneumonia,* all antibiotic resistance levels are very similar between both sample types. Concerning *P. aeruginosa*, lower levels of resistance were observed in urine isolates for ceftazidime (5.3% vs 7.2%), carbapenems (6.3% vs 8.2%), but slightly higher for aminoglycosides (9.5% vs 7.7%) and fluoroquinolones (13.7% vs 10.4%). Concerning urine isolates, much lower levels of resistance were observed in non-hospital labs in comparison to hospital labs, except for the fluoroquinolones in *P. aeruginosa* (14.6% for non-hospital labs vs 13.7% for hospital labs).

**Conclusion:** For urine isolates analyzed in hospital laboratories, overall resistance levels of Gram-negative bacteria followed more or less those of blood/CSF isolates. These bacteria from non-hospital labs are in general more susceptible. This shows the interest to include urine isolates in EARS-BE given the size of collected data and the opportunity to monitor the antimicrobial resistance in non-hospital settings.

**References**

(1) Struyf T, Mertens K. The European Antimicrobial Resistance Surveillance Network Belgium (EARS-Net BE) protocol 2017: Including data call, instructions for participating laboratories, data definition, reporting procedure (Version 3, 26/02/2018). Sciensano. 2018.

**Disclosure of Interest**: None declared

### P59 COMBINED STRATEGIES TO REDUCE THE IMPACT OF MULTIDRUG-RESISTANT GRAM-NEGATIVE BACTERIA IN A LOW RESOURCE SETTING IN BRAZIL: PRELIMINARY RESULTS

#### C. B. Pinheiro^1^, V. L. P. de Amorim^1^, T. D. Correa^2^, J. M. B. D. M. Anacleto^3^, J. Y. Kawagoe^4^, C. B. Dal Forno^1^

##### ^1^Infection Prevention and Control, Hospital Municipal Dr. Moysés Deutsch; ^2^Intensive Care Unit, Hospital Israelita Albert Einstein; ^3^Intensive Care Unit, Hospital Municipal Dr. Moysés Deutsch; ^4^Professional Master's Degree in Nursing, Albert Einstein Israelite School of Health Sciences, São Paulo, Brazil

###### **Correspondence:** C. B. Dal Forno

**Introduction:** Multidrug-resistant Gram-negative bacteria (MRGNB) are a worldwide challenge, especially in resource-limited settings.

**Objectives:** To evaluate the impact of three main interventions implemented in an intensive care unit (ICU) of a resource-limited hospital.

**Methods:** Before-after study conducted from July 2017- April 2019 in a 20-bed ICU at 300-bed public hospital. Three main pillar of interventions were applied to control MRGNB: 1-July 2017: antimicrobial stewardship program (ASP) led by infectious disease physician to reduce carbapenems prescriptions 2- September 2018: new alcohol-based hand rub (ABHR)- an ethanol/n-propanol product at point of care to increase hand hygiene (HH) compliance and 3- December 2018: new cleaning product (n-propanol/quaternary ammonium) to improve cleaning/disinfection of frequently-touched surfaces. The main outcomes evaluated were: antibiotic use, point prevalence of MRGNB colonization by rectal swab specimens (October 2017, January 2018, April 2019), annual ABHR consumption (mL/patient-day) and incidence of hospital-acquired infections (HAI) by MRGNB by semester (sem).

**Results:** There were a significant decrease: carbapenem prescription from July 2017 to April 2019: 1.400 g/DDD/month to 770 g/DDD/month) and the prevalence of MRGNB patients colonization: October 2017 (50%); January 2018 (50%) and April (11.1%). ABHR consumption (mL/patient-day) increased from 36.4 (2017) to 54.2 (2018) and 72.81 (jan-apr 2019). The incidence of HAI by MRGNB/1000 patient-days increased from the 2^nd^ sem 2017 (2.60) to 1^st^ sem 2018 (3.22), but decreased in the 2^nd^ sem 2018 (2.30) and jan-apr (2.64).

**Conclusion:** There were a favorable impact of ASP in reducing the utilization of carbapenems and the prevalence of patient colonization with MRGNB, but not a impact on the incidence of HAI by MRGNB. Other infection prevention and control measures must be evaluated.

**References**

CDC. The Core Elements of Human Antibiotic Stewardship Programs in Resource-Limited Settings: National and Hospital Levels. Atlanta, GA:US Department of Health and Human Services, CDC; 2018

**Disclosure of Interest**: C. Pinheiro: None declared, V. de Amorim: None declared, T. Correa: None declared, J. Anacleto: None declared, J. Kawagoe Employee of: Patient Safety Consultant - B Braun, C. Dal Forno: None declared

### P60 PREVALENCE OF ANTIBIOTIC-RESISTANT ENTEROBACTERIACEAE IN A LARGE NETWORK OF NURSING HOMES

#### C. Legeay^1^, B. Fuchs^2^, T. Haudebourg^2^, S. Corvec^2^, G. Birgand^2^ on behalf of CARBEHPAD Study Group (RTH Pays de la Loire, Raymond F, Poulain C)

##### ^1^Infection Control and Prevention, CHU d'Angers, Angers; ^2^CHU Nantes, Nantes, France

###### **Correspondence:** C. Legeay

**Introduction:** Nursing homes (NH) have been described to be a reservoir of extended spectrum beta-lacatamase producing *Enterobacteriaceae* (ESBLPE). In a regional context of recurrent carbapenemase producing *Enterobacteriaceae* (CPE) outbreaks in acute care settings, we suspect the same phenomenon for CPE.

**Objectives:** This study aimed to evaluate the prevalence of, and factors associated with, ESBLPE and CPE digestive carriage in a large network of NHs in western France.

**Methods:** Fifty NH from the 5 departments of the Pays de la Loire region were randomly selected considering the geographical situation and the density of NHs. In each facility, one third of residents was included in the study. Stool samples were collected with swab and cultured on selective chromogenic media. Mass spectrometry was used to identify *Enterobacteriaceae* species. ESBL or AmpC production was confirmed with MAST^®^ test. Carbapenemase production was tested through CORIS^®^ test. Antibiotic susceptibility tests were performed using Vitek 2. Demographic and clinical data were collected prospectively on the day of the survey. Proportions were compared using Chi-square or Fisher exact test.

**Results:** Overall, 734 residents (30 NHs) were included. Among them, 83 (11.3%) were ESBLPE carriers (71% *Escherichia coli*) and 44 (6%) AmpC carriers (41% *Citrobacter* spp, 31% *Enterobacter* spp). No CPE was identified. Among 146 strains, 91% were susceptible to nitrofurantoin, 64% to ciprofloxacin, 55% to cefoxitin and 75% to trimethoprim. Among all residents, 522 (75%) were female, mean age was 85 years old. 412 (60%) were dement, 29 (4%) travelled abroad in the prior 12 months, respectively 26 (4%) and 5 (0.7%) had a a urinary tract or peripheral venous catheter and respectively 389 (58%) and 446 (67%) had fecal or urinary incontinence. The prevalence of ESBLPE carriage varied from 0 (four NHs) to 45% across nursing homes and 6.0 to 16.4% across departments (p=0.16). Factors associated with the ESBLPE carriage were history of travel abroad (n=9 [31%] ESBLPE+ vs 67 [10%]; OR=3.9; IC95=1.7–8.9; p<0.01), and dementia (n=35 [8%] ESBLPE+ vs 41 [15%]; OR=0.5; IC95=0.3–0.9; p<0.01).

**Conclusion:** This study suggests that NH do not represent a large CPE reservoir in an endemo-epidemic context. The results provide clinical information on the ESBLPE risk among resident for the antimicrobial stewardship in this region.

**Disclosure of Interest**: None declared

### P61 COLONIZATION WITH MULTIDRUG-RESISTANT BACTERIA IN AN INFECTIOUS DISEASES INTENSIVE CARE UNIT

#### M. Lupse^1^, T. Szilagyi^2^, L. Herbel^3^, D. Miclaus^3^, M. Flonta^4^, A. Dicea^3^, A. Muntean^3^

##### ^1^Infectious Diseases, University of Medicine and Pharmacy Cluj-Napoca; ^2^Infectious Diseases; ^3^ICU; ^4^Microbiology, University Hospital of Infectious Diseases, Cluj-Napoca, Romania

###### **Correspondence:** M. Lupse

**Introduction:** Bacterial resistance is an important issue in Intensive care Units (ICU). Screening patients admitted into ICU for multidrug-resistant (MDR) bacteria nasal and fecal carriage became a rule in Romania

**Objectives:** To monitorize colonization with MDR bacteria and the persistence of carriage of such pathogens among patients admitted to the ICU of the University Hospital of Infectious Diseases Cluj-Napoca, Romania and to determine the incidence for nosocomial infections associated with colonization with MDR pathogens, considering that colonization precedes infection.

**Methods:** Prospective observational study: from 01.10.2017 to 15.08.2018 we have included all adult patients admitted directly to the ICU (only first admittance) or with previous admittance in the general wards for less than 48 hours. We performed nasal and rectal swab screening for MDR bacteria (Meticilin Resistant Staphylococcus Aureus (MRSA), ESBL producing Enterobacteriaceae (ESBL-EN), Carbapenemases Producing Gram negative bacteria (CBP-GNB), Vancomycin resistant Enterococcus (VRE), during hospitalization on days 0, 7 and 14.

**Results:** We have included 75 patients, 34 women (45,3%), average age 62.9 years (min 22, max 88 years) with average length of hospitalization of 15 days (min 2 days, max 58 days). Upon admission, 42 patients (56%) were colonized with MDR bacteria, in day 7 there were 65 colonized patients and in day 14, 39 patients. 10 out of 30 CBP-GNB colonized patients and 7 out of 34 VRE colonized patients developed infections with MDR bacteria during hospitalization.

**Conclusion:** MDR bacterial colonization is frequent in patients admitted to the ICU. Colonization with MDR bacteria during hospitalization in the ICU is highest in the first week and decreases significantly later. Colonization could represent a risk factor for increased rates of infection, especially with CBP-GNB and VRE. Active surveillance program for identification of MDR bacteria colonized patients is necessary and useful.

**Disclosure of Interest**: None declared

### P62 COLONIZATION BY MULTIDRUG-RESISTANT BACTERIA IN ONCOLOGICAL PATIENTS AT A UNIVERSITY HOSPITAL IN SOUTHERN BRAZIL

#### T. C. R. Klein^1^, S. B. Filho^1^, G. T. D. Sasso^2^, F. Lunardi^2^, E. O. Luz^2^

##### ^1^Infection Control, University Hospital – UFSC; ^2^Nursing Department, UFSC, Florianópolis, Brazil

###### **Correspondence:** T. C. R. Klein

**Introduction:** Oncological patients frequently present with recurrent infections and require use of broad-spectrum antimicrobials, which may induce microbial resistance.This study shows the prevalence of colonization by multidrug-resistant (MDR) bacteria in cancer patients in 2016 and 2017 in a brasilian hospital.

**Objectives:** To show the prevalence of colonization by MDR bacteria in cancer patients in 2016 and 2017 in a brasilian hospital.

**Methods:** Observational retrospective study from the results of cultures performed in the hospital laboratory. Inclusion criteria: adults with oncologic disease with MDR colonization related to health care admitted in 2016 and 2017. The following microorganisms were identified as MDR bacteria: carbapenem-resistant Enterobacteriaceae with or without carbapenemase production (*Pseudomonas spp, Acinetobacter spp, Klebsiella spp, Escherichia spp, Enterobacter spp, Citrobacter spp*), methicillin-resistant *S. aureus* and vancomycin-resistant *Enterococcus faecium* (VRE).

**Results:** 426 new MDR bacteria cases of colonization were recorded. 69 patients met the inclusion criteria. Of these, 62% were men and 37.68%, women.15% were hospitalized in internal medicine wards, 34.78% in intensive care units and 14.49% in surgical wards. 47.82% of patients had hemato-oncological diseases, 33.33% had gastrointestinal cancer, and 13% had other types of cancer. Overall, 66.66% had a central venous catheter, 52.17% had an urinary catheter, and 43.47% underwent surgical procedures. Carbapenemase-producing *Enterobacteriaceae*(CPE) were identified in 52.17% of patients, VRE in 20%, carbapenem resistant *P*. *aeruginosa*in 18.84%, other multidrug-resistant *Enterobacteriaceae* 14.49%, and the remaining 11.59%. Time between hospitalization and colonization ranged from 4 to 61 days.

**Conclusion:** Colonization by MDR bacteria in cancer patients in this study was more frequent in men admitted to an internal medicine ward with hemato-oncological diseases. More than half had central venous catheter or urinary catheter. Many patients went through surgical procedures. CPEs were the most frequently identified microorganisms. Time for colonization identification had wide variation, but in the first week the colonization rate was lower.

**Disclosure of Interest**: None declared

## Poster session: Microbiological detection of Carbapenemases

### P63 MICROBIOTA OF DIGESTIVE SYSTEM IN COMMUNITY VERSUS HOSPITAL SETTINGS

#### M. M. Abdelhalim

##### Clinical Pathology Department, Faculty of medicine- Cairo University, Cairo, Egypt

###### **Correspondence:** M. M. Abdelhalim

**Introduction:** The healthy microbiota provides protective functions, which refers to the microbiota's ability to prevent colonization and/or expansion of pathogens. Antibiotics use and other exposures in hospitalized patients are associated with disruptions of intestinal microbiota that may select for antibiotic resistance

**Objectives:** Investigation of the effect of hospitalization on intestinal environment that favors the proliferation and virulence of ESBLs producing *Enterobacteriaceae*

**Methods:** A total of 230 rectal swabs were collected from 165 patients from different departments and outpatient clinics of Cairo University Specialized Pediatric Hospital. Out of the 230 total samples,130/230 pre and 48 hours post admission samples, were collected from 65 inpatients, and100/230 samples from 100 outpatients, with no GIT infection symptoms nor previous known antibiotic exposure or hospital admission. Antibiotics susceptibility, with screening of ESBL and AMPC antimicrobial activity were performed according to CLSI 2015 guidelines.Genotypic identification of resistance mechanisms was achieved using PCR assays targeting blaTEM, blaSHV, and blaCTX-M genes

**Results:** ESBL producers were isolated from 40% of outpatients compared to 72.3% and 84.6% of inpatients 1^st^ and 2^nd^ samples respectively, with P- value(< 0.05). Hospitalization has worsen the resistance pattern of isolates of 31/65 (47.7%) cases. Molecular study of ESBL genes showed that, CTX was the most common ESBL gene detected in 78.6% of screened isolates

**Conclusion:** Antibiotics create dysbiosis of microbiota, thereby exposed human microbiome has become a significant reservoir of resistance genes, contributing to the increasing difficulty in controlling bacterial infections.

**Disclosure of Interest**: None declared

### P64 PHENOTYPIC AND GENOTYPIC DETECTION OF CARBAPENEM RESISTANT ACINETOBACTER BAUMANNII IN SURGICAL AND INTENSIVE CARE UNITS (ICUS) IN AL AZHAR UNIVERSITY HOSPITAL – NEW DAMIETTA, EGYPT

#### M. G. M. Elsherbeny^1^, M. Abdelnasser Aly^2^, E. S. A. Gouda^2^, M. M. A. Saleh^1^, M. M. Balboula^3^

##### ^1^Microbiology and Immunology, Faculty of Medicine, Al-Azhar University, New Damietta; ^2^Microbiology and Immunology, Faculty of Medicine, Al-Azhar University, cairo; ^3^Surgery, Al-Azhar University, New Damietta, Egypt

###### **Correspondence:** M. G. M. Elsherbeny

**Introduction:** *Acinetobacter baumannii* has emerged as a healthcare-associated pathogen worldwide. Several epidemiological studies have reported the occurrence of multi-drug resistant *A. baumannii* infections in different regions of the world. The spread of carbapenem-resistant *A. baumannii* is of global concern.

**Objectives:** This work was carried out to detect *Carbapenem*-Resistant *A. baumannii* in Surgical Departments and ICUs. It also aimed to determine the occurrence of *blaoxa-51-like, bla OXA-23, blaIMP* and *blaVIM* genes among the isolated strains.

**Methods:** The current study was conducted on 500 patients attending Surgical Departments and ICUs, Al Azhar University Hospital, New Damietta, Faculty of Medicine in the period between May 2016 and Sept 2018. All samples were traced to the species level using API 20NE system followed by assesment of the different phenotypic assays for detection of *carbapenemase* production using multiplex polymerase chain reaction (PCR).

**Results:** Postoperative infections was detected in 217 (43.4%) out of 500 of patients. *A. baumannii* was considered to be the third most common Gram-negative organism isolated (27, 12.3 %). *A. baumannii* isolates were predominant in ICUs (14, 51.9%). C*arbapenemase* production was detected in *A. baumannii* isolates using the *modified carbapenem inactivation method (mCIM), the modified Hodge test (MHT) and the Carba NP test (p value = 0.000*). Using multiplex PCR analysis, most isolates (12; 44.4%) carried *bla OXA-51-like* gene, followed by ten (37%) isolates that carried both blaOXA-51-like and bla *OXA-23-like* genes. Only one (3.7%) isolate carried *blaOXA*-23-like gene. Metallo-β-lactamase encoding (MBLs) genes were most frequently detected with *bla IMP in* 13 (48.1%) of isolates.

**Conclusion:** The current study suggests that *A. baumannii* is one of the most commonly detected isolate in our hospitals. The *mCIM* assay is the most useful phenotypic method for detection of C*arbapenemase* production. Detection of *Carbapenem resistance genes* is alarming a serious healthcare problem in our hospital.

**Disclosure of Interest**: None declared

### P65 MOLECULAR EPIDEMIOLOGY OF BETA-LACTAM RESISTANCE AMONG ETEROBACTERIACEAE IN FOUR TERTIARY- CARE HOSPITALS IN EGYPT

#### A. Elkholy^1^, S. A. Girgis^2^, A. R. Elmanakhly^3^, D. H. Abdel hamid^2^, M. A. Zahran^3^

##### ^1^Microbiology and immunology, Cairo university, Faculty of midicine; ^2^Microbiology and immunology, Ain Shams university, Faculty of medicine; ^3^Infection control, Dar Alfouad hospital, Cairo, Egypt

###### **Correspondence:** A. Elkholy

**Introduction:** Monitoring the trends in antimicrobial resistance is essential to guide patient therapy and to take preventive actions.

**Objectives:** We report here the molecular epidemiology of B- lactem resistance among Gram-negative pathogens isolated from ICUs in 4 major tertiary hospitals in Egypt by multiplex PCR assays for extended – spectrum β-lactamase (ESBL), carbapenemases genes and Amp-C β-lactamases, as part of the Study for Monitoring Antimicrobial Resistance Trends (SMART).

**Methods:** This study was conducted in 4 major tertiary- care hospitals participating in the (SMART) from 2012 to 2016. Enterobacteriaceae isolates were isolated from respiratory (RTI), urinary (UTI) and intra- abdominal infections (IAI). Molecular testing was done by multiplex PCR in a reference laboratory.

**Results:** A total of 493 isolates were identified from IAI (37.53%), UTI (33.67%), RTI (25.15%) and others (3.65%). As regards EsβL, the *bla*_CTX-M-15_ was detected in 73.83% of the isolates followed by *bla*_TEM-OSBL_ (48.68%), and *bla*_SHV-OSBL (_44.02%). 51.12% of *K. pneumoniea* isolates harbored *bla*_SHV-OSBL_ and 12.78% harbored *bla*_SHV-12_ gene. *E. coli* didn’t show any predominance of SHV genes otherwise it showed a high prevalence of *bla*_TEM-OSBL_ (47.48%) and *bla*_CTX-M15_ (68.475%). Carbapenemase genes were detected in 20% of isolates from 2012 till 2014; with a marked increase in the resistance rate to more than 50% of isolates in 2015 and 2016. *K. pneumoniae* isolates showed high rates of *blaOXA-48* (40.60%), followed by *bla*_NDM-1_ (23.68%). *E. coli* isolates showed 2.28% of *bla*_OXA48_ , 5.47% of *bla*_OXA-181_ and 9.58% of *bla*_NDM-5_.

Nevertheless, 3 (0.45%) isolates harboring *bla*_VIM_ were first detected in *K. pneumoniea* and *E. coli* in 2016 and only 2 (0.75%) isolates of *bla*_KPC_ were detected in 2015.

The rate of AmpC β-lactamases ranged from 2.56% in 2014 to 15.11% in 2016 with a total rate of 9.74%, which is significantly lower than ESBL and carbapenem- resistance patterns. However, there is an emergence of new CMY-59 and CMY-42 in Egypt.

**Conclusion:** The high rate of resistance is alarming, which calls for a national antimicrobial stewardship program, not only in hospitals, but also in the community, among animals and in the environment.

**Disclosure of Interest**: None declared

### P66 EXTENDED-SPECTRUM-BETA-LACTAMASE PRODUCING BACTERIA RELATED URINARY TRACT INFECTION IN RENAL TRANSPLANT RECIPIENTS

#### M. Wysocka^1^, B. Krawczyk^1^, J. Gołębiewska^2^, M. R. Oggioni^3^, M. Fordon^1^

##### ^1^Gdańsk University of Technology; ^2^Medical University of Gdańsk, Gdańsk, Poland; ^3^University of Leicester, Leicester, United Kingdom

###### **Correspondence:** M. Wysocka

**Introduction:** Urinary tract infection (UTI) is a common complication after kidney transplantation, often associated to graft loss and increased healthcare costs. The recent rise in incidence of extended-spectrum beta-lactamase+ (ESBL) producing bacteria causing UTI among renal transplant patients (RTx) poses new and significant challenges in terms of management and outcome.

**Objectives:** We examined the molecular epidemiology of ESBL producing bacteria causing UTI in these patients.

**Methods:** Detection of ESBLs and carbapenemases produced by studied strains was performed using PCR. Phylogenetic background of *Klebsiella pneumoniae* isolates was analyzed by PCR Melting Profiles (MP) and the following VFs genes: *fimH-1, uge, irp-2, kpn, ycfM, mrkD, rmpA, magA, hlyA, cnf-1* by multiplex PCR. Multiple patient characteristics including demographics, immunosuppression, recurrences, allograft function and outcome were analyzed.

**Results:** A total of 64 *K. pneumoniae* strains isolated from renal transplant patients were recovered from clinical specimens in a university hospital. More than 80% strains carried ESBL genes. A highly resistant NDM-1 *K. pneumoniae* were reported. PCR MP typing showed a diverse population, among which different genetic profiles appeared in isolates from two or more patients, suggesting nosocomial infections. VF gene profiles were highly homogenous.

**Conclusion:** RTx patients are at a higher risk for developing ESBL producing bacteria associated UTI. Overall, the coexistence of virulence and carbapenem resistance in clinical *K. pneumoniae* isolates is a serious concern. Moreover, the emergence of NDM-1 among isolates warrants stringent surveillance and control measures.

**Disclosure of Interest**: None declared

### P67 DIVERSITY OF Β-LACTAMASES OF BROAD HYDROLYTIC SPECTRUMS IN MDR GRAM NEGATIVE NOSOCOMIAL ISOLATES FROM AN EGYPTIAN HOSPITAL

#### A. K. Abdulall^1^, H. A. El-Mahallawy^2^, A. H. El-Manakhly^3^ on behalf of Antimicrobial resistance

##### ^1^Microbiology and Immunology, Faculty of Pharmacy - Al-Azhar University; ^2^clinical pathology, clinical pathology; ^3^clinical microbiology lab., Dar El foad hospital, Giza, Egypt

###### **Correspondence:** A. K. Abdulall

**Introduction: Introduction:** Hospital acquired infections due to MDR Gram-negative bacilli are becoming a global health care issue. Productions of multiple types of β-lactamases with broad hydrolytic spectrum have been observed lately in MDR phenotypes. These include the extended spectrum β-lactamases (ESβLs), AmpC β-lactamases, class A, *Klebsiella pneumoniae* carbapenemases (KPC) and class B carbapenemases, metallo-β-lactamases (MBL).

**Objectives:** Therefore, rapid detection and proper reporting of such multidrug resistance markers is mandatory to avoid their dissemination.

**Methods:** 100 MDR Gram negative isolates recovered from inpatients hospitalized at the National Cancer Institute, Cairo, Egypt. They were tested for production of ESβLs, Amp C β-lactamases, MBL and KPC by several phenotypic detection tests.

**Results:** 25% of the studied isolates were further classified as extensive drug resistant (XDR) phenotypes. *E.coli* and *Klebsiellae pneumoniae* were the predominant species in this study. 78% of the studied isolates were ESβL producers, 29% were Amp C β-lactamases producers, 17% had MBL and only 6% were KPC producers. This was the first report on emergence of KPC producing phenotypes in Egypt. *Klebsiella pneumoniae* was the major producers of ESβL, MBL and KPC B-lactamases, while *Escherichia coli* were the predominant Amp C β-lactamase producing isolates. The coexistence of the four types of β-lactamases detected in this study was observed in two XDR *K. pneumoniae* isolates.

**Conclusion:** Phenotypic detection methods of indicator mechanisms of multidrug resistance should be employed in routine laboratory work. Phenotypic methods were efficient, subjective, and easy to carry and have relative low costs. It is an economic solution for infection control in hospital settings in developing countries with limited financial resources

**Disclosure of Interest**: A. Abdulall Employee of: no, Grant/Research support from: no, Speaker's bureau of: no, Shareholder of: no, Consultant for: no, Paid instructor for: no, Other conflict with: no, H. El-Mahallawy: None declared, A. El-Manakhly: None declared

### P68 Withdrawn

### P69 A NEW MULTIPLEX PCR TO DETECT CONCURRENT EXISTENCE OF QNRB, AAC(6’) AND RMTA GENES AMONG (MDR)P. AERUGINOSA ISOLATES FROM BURN WOUND INFECTIONS

#### M. Hakemi-Vala^1^, A. Shaban Ghahrood^2^, A. Hashemi^1^, G. Talebi^3^

##### ^1^Microbiology, Shahid Beheshti University of Medical Sciences; ^2^Microbiology, Pharmaceutical Sciences Branch, Islamic Azad University, Tehran, Iran; ^3^Microbiology, Faculty of Basic Sciences, Tehran Science and Research Branch, Islamic Azad University, Tehran, Iran , Tehran, Iran, Islamic Republic Of

###### **Correspondence:** M. Hakemi-Vala

**Introduction:** Recently by emerging and increasing of multi drug resistant bacteria (MDR), need to design new fast methods of detection is mandatory.

**Objectives:** The aim of this study was to design a new multiplex PCR to detect concurrent existence of qnrB, aac(6’) and rmtA genes among (MDR)*P. aeruginosa* isolates from burn wound infections.

**Methods:** 92 isolates of *P. aeruginosa* of burn wound infections of Motahhari hospital in Tehran were collected during 2017-2018. Antimicrobial susceptibility test (AST) was done according to CLSI protocol by using disk diffusion method. The frequency of each of qnrB, aac(6’) and rmtA genes were determined by PCR and confirmation was done by sequencing.. Then, a new multiplex PCR was used to evaluate the presence of these three genes, simultaneously. *P. aeruginosa* ATCC 27853 was used as a control strain.

**Results:** Based on AST the resistant profile of collected isolates was, 81 (88.32%) to Ciprofloxacin, 80 (87.4%) to Meropenem, 78 (84.64%) to Gentamicin, 79 (85.56%) to Cefepime, 75 (81.88%) to Amikacin, 66 (71.74%) to Aztreonam, 63 (69%) to Ceftazidime. All isolates were susceptible to Colistin. of The frequency of qnrB, aac(6’) and rmtA genes were 0(0%), 8(9.2%) and 38(41.4%) respectively. Concurrent presence of aac(6’) and rmtA genes was confirmed in 2 (1.84%) isolates by the new designed multiplex PCR.

**Conclusion:** The results of this study showed; A) high rate of antibiotic resistance among Pseudomonas aeruginosa isolates from burn patients, B) Colistin is the best choice for treatment, C) the frequency of rmtA gene is higher than aac (6’) gene among these bacteria *and D) the* newly designed multiplex PCR was qualified to detect concurrent existence of qnrB, aac(6’) and rmtA resistant genes in these bacteria in just one reaction.

**Disclosure of Interest**: None declared

### P70 COMPARATIVE GENOMICS AND GENETIC CHARACTERIZATION OF INCX3 PLASMIDS

#### Y. Yu, R. S. Yang, J. Sun, Y. F. Zhou, X. P. Liao, Y. H. Liu

##### College of Veterinary Medicine, South China Agricultural University, Guangzhou, China

###### **Correspondence:** X. P. Liao

**Introduction:** IncX3 plasmid is associated with the dissemination of the ESBL (*bla*_SHV_) and carbapenemase genes (*bla*_NDM_, *bla*_OXA_, *bla*_KPC_) from multiple species of Enterobacteriaceae.

**Objectives:** To characterize the underlying evolution process of IncX3 plasmid in Enterobacteriaceae.

**Methods:** 152 IncX3 plasmids were retrieved from Genbank nucleotide database in July 2018, excluding the hybrid, uncompleted sequence and replicon deleted plasmids. All plasmids were applied for core-genome alignments to construct the phylogenetic tree and gain insights into the phylogeny of all IncX3 plasmids.

**Results:** The most prevalent region of IncX3 plasmid is China (63/152), followed by the United States (24/152). *Escherichia coli* (74/152) and *Klebsiella pneumoniae* (46/152) are the most frequently host strains, on the other hand *bla*_NDM_ (93/152) and *bla*_SHV_ (59/152) are the most prevalent resistance genes carried by IncX3 plasmid. Phylogenetic tree revealed the high stability of the plasmid core genome, and 152 plasmids were divided into 4 clades, among which146 plasmids were belonged to clade 1.

**Conclusion:** The analysis of variable region uncovered that the IncX3 type plasmid may only harbor *bla*_SHV_ gene in the early stage, and *bla*_NDM_, *bla*_OXA_ and *bla*_KPC_ are possibly inserted into the plasmid by the insertion sequence or further evolved by different recombination events.

**Disclosure of Interest**: None declared

### P71 REAL-TIME PCR-BASED DETECTION OF ESBL- AND CARBAPENEMASE GENES POSSESSING ENTEROBACTERALES IN URINE SPECIMENS FROM ADULT PATIENTS WITH SUSPECTED URINARY TRACT INFECTIONS

#### O. Timoshina^1^, Y. Savochkina^1^, S. Polikarpova^2^, O. Timofeeva^2^, A. Guschin^1^, D. Shagin^1^, V. Akimkin^1^

##### ^1^Federal Budget Institution of Science "Central Research Institute for Epidemiology" of The Federal Service on Customers' Rights Protection and Human Well-being Surveillance; ^2^State Budgetary Healthcare Institution "Municipal Clinical Hospital №15 named O.M. Filatov", Moscow, Russian Federation

###### **Correspondence:** O. Timoshina

**Introduction:** The spread of resistance to cephalosporins and carbapenems among pathogens causing urinary tract infections (UTI) poses an important threat to public health.

**Objectives:** The aim of this study was to evaluate prevalence of *Enterobacterales* possessing ESBL and carbapenemase genes revealed in urine specimens from adult patients with suspected UTI.

**Methods:** A retrospective analysis of 260 urine samples obtained from adult patients, who were examined for diagnosis of UTI at 1265-bed hospital in Moscow between October 2017 and January 2018, was performed using two real-time PCR assays. The first PCR assay composed of two multiplex tests allowing quantitative detection of *E.coli, K. pneumoniae, Proteus spp.* and other *Enterobacterales* DNA. The second PCR assay composed of three multiplex tests: the first one was for CTX-M group ESBL genes detection (“Amplisens ESBL CTX-M-FL”), the second one was for KPC- and OXA-48-like carbapenemase genes detection (“Amplisens MDR KPC/OXA-48–FL”), and the last one – for VIM-, IMP-, and NDM metallo-beta-lactamase (MBL) genes detection (“Amplisens MDR MBL–FL”). All these real-time PCR-based assays had been developed and evaluated previousely in our laboratory.

**Results:** According to the results of real-time PCR *Enterobacterales* DNA load exceeding 2x10^4^ GE/ml (genome equivalents per milliliter) was revealed in 95 urine samples. In most of these cases (N=77) *E.coli* DNA was revealed and also *K. pneumoniae* (N=11) and *Proteus spp* (N=9) DNA were detected.

The CTX-M group ESBL genes were detected in 37/95 (39%) samples with *Enterobacterales* DNA load more than 2x10^4^ GE/ml. The OXA-48-like carbapenemase genes were found in 2/95 cases (2%). The NDM group MBL genes were revealed in 4/95 cases (4%). In every of these 5 samples carbapenemase genes were detected along with *blaCTX-M* genes. The presence of *blaNDM* along with *blaOXA-48-like* and *blaCTX-M* was shown in one sample containing *K. pneumoniae.*

**Conclusion:** Using real-time PCR-based technique a significant prevalence of *Enterobacterales* possessing group NDM MBL genes (4%), OXA-48-like carbapenemase genes (2%), and CTX-M group ESBL genes (39%) was revealed in urine from patients with suspected UTI.

**Disclosure of Interest**: None declared

### P72 THE EFFECT OF VARYING MULTIDRUG-RESISTENCE (MDR) DEFINITIONS ON RATES OF MDR GRAM-NEGATIVE RODS

#### A. Wolfensberger^1^, S. P. Kuster^1^, M. Marchesi^2^, R. Zbinden^2^, M. Hombach^2,3^

##### ^1^Department of Infectious Diseases and Hospital Epidemiology, UNIVERSITY HOSPITAL ZÜRICH; ^2^Institute of Medical Microbiology, University of Zurich, Zürich; ^3^Present affiliation: Roche Diagnostics International AG, Rotkreuz, Switzerland , Rotkreuz, Switzerland

###### **Correspondence:** A. Wolfensberger

**Introduction:** The number of Gram-negative bacteria that are resistant to multiple antibiotics is on a constant rise and infections due to these resistant organisms pose an increasing threat to the achievements of modern medicine. Definitions of multidrug resistance (MDR) are neither harmonized between countries, nor between hospitals in the same country, nor do guidelines on IPC standards for patients with GN-MDRO exist to date.

**Objectives:** To determine the effects of different definitions of multidrug-resistance on rates of Gram-negative multidrug-resistant organisms (GN-MDRO).

**Methods:** MDR definitions of the European Centre for Disease Prevention and Control (ECDC), the German Commission of Hospital Hygiene and Infection Prevention (KRINKO) and the University Hospital Zurich (UHZ) were applied on a dataset comprising isolates of *Escherichia coli*, *Klebsiella pneumoniae, Enterobacter* sp.*, Pseudomonas aeruginosa,* and *Acinetobacter baumannii* complex. Rates of GN-MDRO were compared and the percentage of patients ever having had a GN-MDRO was calculated.

**Results:** In total 11’407 isolates from a three year period were included. For *Enterobacteriaceae* and *P. aeruginosa,* highest MDR-rates resulted from applying the ‘ECDC-MDR’ definition. ‘ECDC-MDR’ rates were up to four times higher compared to ‘KRINKO-3MRGN’ rates, and up to six times higher compared to UHZ rates. Lowest rates were observed when applying the ‘KRINKO-4MRGN’ definitions. Comparing the ‘KRINKO-3MRGN’ with the UHZ definitions did not show uniform trends, but yielded higher rates for *E. coli* and lower rates for *P. aeruginosa*. On the patient level, the percentages of GN-MDRO carriers were 2.1%, 5.5%, 6.6%, and 18.2% when applying the ‘KRINKO-4MRGN’, ‘UHZ-2015’, ‘KRINKO-3MRGN’, and the ‘ECDC-MDR’ definition, respectively.

**Conclusion:** Different MDR-definitions lead to considerable variation in rates of GN-MDRO. Differences arise from the number of antibiotic categories required to be resistant, the categories and drugs considered relevant, and the antibiotic panel tested. MDR definitions should be chosen carefully depending on their purpose and local resistance rates, as definitions guiding isolation precautions have direct effects on costs and patient care.

**Disclosure of Interest**: None declared

### P73 RECTAL CARRIAGE OF CIPROFLOXACIN-RESISTANT ENTEROBACTERIACEAE AMONG PATIENTS IN TWO DUTCH HOSPITALS: RESULTS FROM THE I-4-1-HEALTH PROJECT

#### M. Kluytmans - van den Bergh^1,2^, C. Verhulst^1^, B. Diederen^3^, E. Maas^3^, V. Weterings^1^, I. Willemsen^1^, J. Kluytmans^1,2^, on behalf of the i-4-1-Health Study Group

##### ^1^Amphia Hospital, Breda; ^2^UMC Utrecht, Utrecht; ^3^ZorgSaam Hospital, Terneuzen, Netherlands

###### **Correspondence:** C. Verhulst

**Introduction:** Antimicrobial resistance (AMR) in Gram-negatives poses a threat to global public health, with the gut as main reservoir.

**Objectives:** To determine the prevalence and predictors of rectal carriage of ciprofloxacin-resistant Enterobacteriaceae (cipR-E) among patients in Dutch hospitals, and the occurrence of co-carriage of cipR-E and extended-spectrum beta-lactamase-producing (ESBL-E) or carbapenem-resistant Enterobacteriaceae (CRE).

**Methods:** Repeated AMR prevalence surveys were performed in 9 wards of 2 Dutch hospitals in 2017/2018. Perianal or, if appropriate, gastrointestinal stoma swabs (Fecal Swab, COPAN Italy) were pre-enriched in a non-selective tryptic soy broth (COPAN Italy) and subsequently cultured on selective agar plates (McC cipro 2 mg/L, in-house; ChromID ESBL/CARBA/OXA48, bioMérieux) to identify cipR-E, ESBL-E and CRE. Cultures with a negative growth control or transport time >72 h were invalid. Vitek2 (bioMérieux) and combined disk diffusion (Rosco) were used for susceptibility testing and confirmation of ESBL production. EUCAST guidelines were used for interpretation. Demographic patient data were retrieved from the medical record. A multivariable logistic regression model was used to identify independent predictors for cipR-E rectal carriage.

**Results:** Of 1,226 patients hospitalised, 887 (72%) had a valid culture. The prevalence of cipR-E carriage was 12% (109/887). Independent predictors for cipR-E carriage were age (OR_adj_ 1.020; 95% CI 1.003–1.039) and days since hospital admission (OR_adj_ 1.017; 95% CI 1.005–1.030). Hospital, year of culture, specialty, sex, nursing home residency or previous hospitalisation (1 yr) were not independently associated with cipR-E carriage. Data on antibiotic use were not available. *E. coli* was the predominant cipR-E (70%; 94/135). Co-carriage with ESBL-E was identified in 31% (34/109) of cipR-E carriers compared to 4% (35/778) of non-cipR-E carriers (RR 6.9; 95% CI 4.4–10.8). Co-carriage with CRE was <1% for cipR-E carriers and non-carriers.

**Conclusion:** Rectal carriage of cipR-E is common among patients in Dutch hospitals, with age and days since hospital admission being independent predictors. CipR-E carriers are at increased risk for ESBL-E carriage.

**Disclosure of Interest**: M. Kluytmans - van den Bergh Grant/Research support from: Interreg Vlaanderen-Nederland / COPAN Italy / bioMérieux , C. Verhulst Grant/Research support from: Interreg Vlaanderen-Nederland / COPAN Italy / bioMérieux, B. Diederen Grant/Research support from: Interreg Vlaanderen-Nederland / COPAN Italy / bioMérieux, E. Maas Grant/Research support from: Interreg Vlaanderen-Nederland / COPAN Italy / bioMérieux, V. Weterings Grant/Research support from: Interreg Vlaanderen-Nederland / COPAN Italy / bioMérieux, I. Willemsen Grant/Research support from: Interreg Vlaanderen-Nederland / COPAN Italy / bioMérieux, J. Kluytmans Grant/Research support from: Interreg Vlaanderen-Nederland / COPAN Italy / bioMérieux

### P74 PREDICTIVE ACCURACY OF RISK PREDICTION MODEL OF CARBAPENEM-RESISTANT ENTEROBACTERIACEAE (CRE) COLONIZATION: A PROSPECTIVE COHORT STUDY IN INTENSIVE CARE UNITS

#### J. Y. Song^2^, I. Jeong^1^

##### ^1^PUSAN NATIONAL UNIVERSITY; ^2^PUSAN NATIONAL UNIVERSITY Yangsan Hospital, Yangsan, Korea, Republic Of

###### **Correspondence:** I. Jeong

**Introduction:** We had developed the risk model of carbapenem-resistant Enterobacteriaceae (CRE) colonization through retrospective cohort study. The risk prediction logistic model for CRE colonization was E (logit of CRE colonization) = -2.821 + 1.606 (isolation of multi-drug resistant organisms) + 1.347 (≥15 days of cephalosporin administration) + 0.980 (≥15 days of carbapenem administration) + 0.544 (≥21 points of APACHEII score). The risk prediction model showed .795 of area under receiver operator characteristic (ROC) curve, and 68.9% of correct classification, 79.8% of sensitivity and 66.2% of specificity at .20 of cutting point.

**Objectives: :** This study was aimed to externally validate the predictive accuracy of previous risk prediction model of CRE colonization developed by researchers.

**Methods:** This retrospective cohort study was done with medical record review at a tertiary hospital between November 1, 2017 and May 31, 2018. The subjects were 414 adult patients (48 CRE carriers and 366 no-CRE carriers) admitted to intensive care units during the study time.

**Results:** The area under the ROC curve (AUC) of The risk prediction model was 0.883. When predicted probability values for CRE colonization in the curve of 0.20 and 0.25 were used as the cutoff point to distinguish between CRE carriers and no-CRE carriers, the sensitivity was 52.1% and 45.8%, the specificity was 92.6% and 95.1%, the positive predictive value was 48.1% and 55.0%, the negative predictive value was 93.6% and 93.0%, and the classification accuracy was 87.9% and 89.4%, respectively.

**Conclusion:** The risk prediction model of carbapenem-resistant Enterobacteriaceae (CRE) colonization showed higher AUC in validation stage than that in developmental stage. Therefore, we recommend for nurse in ICUs to utilize the model to identify the high risk population of CRE.

**Disclosure of Interest**: None declared

## Poster session: Candida Auris

### P75 CONTROL OF CANDIDA AURIS IN HEALTHCARE INSTITUTIONS. OUTCOME OF AN INTERNATIONAL SOCIETY OF ANTIMICROBIAL CHEMOTHERAPY EXPERT MEETING

#### N. Kenters^1^, on behalf of International Society of Antimicrobial Chemotherapy, Working Group Infection Prevention and Control, M. Kiernan^2^, A. Chowdhary^3^, D. W. Denning^4^, J. Pemán^5^, S. Schelenz^6^, E. Tartari^7^, A. Widmer^8^, J. F. Meis^9^, A. Voss^1,10^ on behalf of an International Society of Antimicrobial Chemotherapy, Working Group Infection Prevention and Control

##### ^1^Medical microbiology, CWZ, Nijmegen, Netherlands; ^2^Richard Wells Research Centre, University of West London, United Kingdom; ^3^Department of Medical Mycology, University of Delhi, Dehli, India; ^4^Faculty of Biology, Medicine and Health, University of Manchester, Manchester Academic Health Science Centre, and National Aspergillosis Centre, Manchester University NHS Foundation Trust, Manchester, United Kingdom; ^5^Department of Clinical Microbiology, Hospital Universitari i Politècni La Fe, Valencia, Spain; ^6^Department of Microbiology, Royal Brompton Hospital, London, United Kingdom; ^7^Faculty of Health Sciences, University of Malta, Msida, Malta; ^8^University of Basel Hospitals & Clinics, Basel, Switzerland; ^9^Centre of Expertise in Mycology Radboudumc/CWZ; ^10^Medical Microbiology, Radboudumc, Nijmegen, Netherlands

###### **Correspondence:** N. Kenters

**Introduction:** *Candida auris* is a difficult to detect, emerging fungal pathogen responsible for invasive infections and outbreaks in healthcare facilities worldwide, which have been difficult to control. Evidence for infection prevention and control (IPC) remains scarce.

**Objectives:** To provide pragmatic guidance and recommendations for IPC measures

**Methods:** A group of infection prevention and mycology experts reviewed the published literature on *C. auris* and identified best practice based on available scientific evidence, existing guidelines and expert opinion. The available literature from both laboratory and clinical settings was reviewed, drawing on the expert experience of outbreaks in order to formulate implementable guidance.

**Results:** We have proposed a set of recommendations on key interventions needed to contain a single case or *C. auris* outbreak including: screening, standard and extra precautions, cleaning and disinfection, patient transfers, outbreak management, decolonization and treatment. Measures to contain a *C. auris* single case or outbreak need a different approach than for other MDROs since transmission before the first clinical case appears is likely.

**Conclusion:** Healthcare facilities can use the IPC recommendations identified by international experts and current epidemiology on *C. auris* to prevent transmission and control outbreaks.

**Disclosure of Interest**: None declared

### P76 ANTIMICROBIAL PHOTODYNAMIC THERAPY FOR CONTROL OF THE NEWLY EMERGING NOSOCOMIAL PATHOGEN, C. AURIS

#### C. Cross, C. Romo, N. Loebel

##### Ondine Research Labs, Bothell, United States

###### **Correspondence:** N. Loebel

**Introduction:** Multi-drug resistant *C. auris* infections are being increasingly reported from around the world. *C. auris* is difficult to identify with standard laboratory methods and infections can be invasive and deadly. In many cases, *C. auris* is resistant to any of the drug classes used to treat fungal infections and therefore has high potential to cause outbreaks in healthcare facilities.

**Objectives:** Antimicrobial devices such as ultraviolet robots and disinfectant sprays are helpful to decolonize facilities and equipment, but are unsafe for direct human use. A new approach to safe elimination of *Candida auris* from the nares and skin of colonized patients and healthcare workers would contribute to reducing infections by this evolving nosocomial threat. Antimicrobial photodynamic therapy (aPDT) has been successfully deployed in tens of thousands of patients in Canada (MRSAid/Steriwave nasal decolonization system, Ondine Biomedical Inc., Canada), with the goal of eradicating MRSA from the anterior nares as a means of reducing post-surgical staphylococcal infections. The objective of the present work was to demonstrate expanded effectiveness of aPDT against both planktonic and biofilm forms of *C. auris*.

**Methods:** 48-hr planktonic and biofilm cultures of *Candida auris* clinical isolates (CDC, Atlanta, GA) were established in 96-well black polystyrene plates with clear well bottoms. Cultures were exposed to photosensitizer and light, as well as standard positive and negative controls. Experiments were also conducted to demonstrate absence of cross-resistance induction to standard antifungals including azoles and Amphotericin B.

**Results:** aPDT using standard clinical parameters completely eradicated (10^6^ logs) both planktonic and biofilm forms of all *C. auris* strains tested. Experiments at sublethal aPDT doses demonstrated no increase in MIC to any tested antifungal. Intriguingly, the MIC of Amphotericin B to *C. auris* decreased 8-fold after sublethal aPDT exposure, implying an increase in susceptibility to this Amphotericin B which was inherited across successive fungal generations.

**Conclusion:** aPDT using standard clinical parameters proved highly effective at eradicating all clinical isolates of *C. auris* tested, including multi-drug resistant strains. This work establishes a potential foundation for control of *C. auris* as a newly emerging nosocomial pathogen.

**Disclosure of Interest**: C. Cross Employee of: Ondine Research Labs, C. Romo Employee of: Ondine Research Labs, N. Loebel Employee of: Ondine Research Labs

### P77 MANAGEMENT OF CANDIDA AURIS EXPOSURE AMONG PATIENTS AND HEALTHCARE WORKERS AT TERTIARY CARE SETTING

#### A. Mohammed^1^, M. M. Alshamrani^1^, A. El-Saed^1^, M. Alghoribi^2^, S. Al Johani^3^, H. Cabanalan^1^, H. Balkhy^4^

##### ^1^Infection Prevention & Control, King Abulaziz Medical City; ^2^Infectious Diseases, King Abdullah International Medical Research Center; ^3^Microbiology, King Abulaziz Medical City, Riyadh, Saudi Arabia; ^4^Antimicrobial Resistance, World Health Organization, Geneva, Switzerland

###### **Correspondence:** A. Mohammed

**Introduction:** Candida auris is an emerging multidrug-resistant fungal pathogen that has been implicated in a number of invasive infections and outbreaks in healthcare facilities.

**Objectives:** The objective was to describe our local experience with candida auris exposure at a tertiary care hospital at Riyadh, Saudi Arabia.

**Methods:** A surveillance study has been conducted at King Abdulaziz Medical City, Riyadh between March 2018 and May 2019. After detecting the index cases, screening of all exposed healthcare workers (HCWs) and patients began. Post-exposure screening included those who had direct contact or shared the same location with a laboratory confirmed case. A total of 707 HCWs and 253 patients were screened. Screening procedure for HCWs included swabbing of nares, axilla, and groin for fungal culture. For patients, screening procedure included additionally rectal swab.

**Results:** During the study, a total 23 primary cases have been detected. Post-exposure screening identified 11 more cases, all were patients. The cases gradually increased to reach peak in April 2019 with much reduction in May 2019. For primary cases, positive clinical specimens were mainly urine (65.2%), blood (26.1%), and tracheal aspirate (13.0%). Males represented 67.6% of the patients and the average age was 63.9±19.8 years. At the time of diagnosis, 52.9% of patients were admitted to intensive care units and 47.1% to wards. The median (IQR) duration before diagnosis was 30 (12.8-73) days. All primary cases were isolated on the day of results release (median 4.0 days from sampling). All screened patients were under isolation during screening. The median (IQR) length of hospital stay was 80.5 (33.8-134.5) days. The mortality by the end of the study was (4/34) 11.8%, with 38.2% discharged and 50.0% still in the hospital.

**Conclusion:** This study demonstrates a risk of cross transmission of candida auris in healthcare facilities among patients. As all screened HCWs were negative, colonization among them is unlikely. Active screening of exposed individuals followed by appropriate infection control measures may help early detection of candida auris and guard against future large scale spread in healthcare settings.

**Disclosure of Interest**: None declared

### P78 ORAL COLONIZATION BY CANDIDA SPP. IN LIVER TRANSPLANT PATIENTS: MOLECULAR IDENTIFICATION, ANTIFUNGAL SUSCEPTIBILITY TESTING AND ASSOCIATED FACTORS

#### C. S. Sabadin^1^, S. L. Lopes^1^, D. A. Da Matta^1^, L. Rigo^2^, A. S. Melo^1^, O. F. Gompertz^1^, D. A. Barbosa^1^

##### ^1^Federal University of São Paulo , São Paulo; ^2^Meridional Faculty , Passo Fundo, Brazil

###### **Correspondence:** C. S. Sabadin

**Introduction:** Oral colonization by *Candida* spp. has been associated with increased risk of systemic infection in transplant recipient patients.

**Objectives:** To investigate oral colonization by *Candida* spp., identify to species level, evaluate antifungal susceptibility profile and associated factors.

**Methods:** A prospective cohort study was carried out with 97 liver transplant patients attended by a hospital transplant center, which is a reference for liver transplants in southern Brazil. All patients were submitted to two oral swab collections, with a 6-month interval. The samples were cultured in CHROMagar® *Candida* for yeast isolation and the identification was carried out by sequencing the ITS region of rDNA. The susceptibility test was performed to Fluconazole (FLC), Amphotericin B (AMB) and Micafungin (MFG) using the broth microdilution method recommended by CLSI, document M27-A3. Pearson's Chi-square test was used to analyze the qualitative variables (p> 0.05).

**Results:** Eighty-two patients were investigated for colonization and fifteen were excluded for presenting oral candidiasis. The total of 82 patients, 50 were colonized in the first collection and 49 in the second. *C. albicans* was the most prevalent species in both collections (n=31/50 and n=29/49 respectively). It is worth highlighting the finding of 11 isolates of *C. dubliniensis* and one of *C. fermentati* in the study. In 23 patients the yeast species remained the same and in 25 the species substitution occurred. Regarding FLC, 38 isolates from the first collection were susceptible (S), 7 susceptible dose dependent (SDD) and 5 resistant (R). In the second collection, there were 32 isolates S, 11 SDD and 6 R. For AMB and MFG all the isolates were susceptible. In the analysis of factors associated with colonization, none of them presented statistically significant difference.

**Conclusion:** Colonization of the oral cavity of liver transplant patients by *Candida* spp. surpassed 50%. The prevalent species was *C. albicans*. Most of the isolates were susceptible to antifungal agents, however, species resistant to FLC were identified, which may be considered a risk factor for infections resistant to treatment with this agent.

**Disclosure of Interest**: None declared

## Poster session: Antimicrobial resistance in Low and Middle Income Countries 1

### P79 IDENTIFICATION AND ANTIMICROBIAL RESISTANCE PROFILLING OF PSEUDOMONAS AERUGINOSA USING HIGH-THROUGHPUT SEQUENCING

#### D. Shagin^1^, Y. Mikhaylova^1^, A. Shelenkov^1^, Y. Yanushevich^1^, V. Fomina^2^, M. Zamyatin^2^, V. Akimkin^1^

##### ^1^Central Research Institute of Epidemiology; ^2^Pirogov National Medical and Surgical Center, Moscow, Russian Federation

###### **Correspondence:** D. Shagin

**Introduction:** *Pseudomonas aeruginosa* is a dangerous opportunistic pathogen and the most abundant bacterial species causing nosocomial infections in Russia. Clinical isolates *P. aeruginosa* are characterized by rapid acquisition to antimicrobial resistance.

**Objectives:** The problem of increasing prevalence in hospitals of multiresistant bacteria, especially those that are resistant to carbapenem antibiotics, emphasize the need of typing methods that allow to comprehensively identify and compare intra- and interlaboratory isolates easily.

**Methods:** In this work whole-genome sequencing was carried out on 70 isolates of *P. aeruginosa* including 5 environmental isolates collected from Pirogov National Medical and Surgical Center on an Illumina HiSeq platform. Sensitivity spectrum to antimicrobial drugs was determined by disc-diffusion method. The assembly was performed with Spades. Determinants of antimicrobial resistance, genes of virulence, sequence-types were revealed with Resfinder 2.1, Virulence finder and MLST 1.8, respectively.

**Results:** According to MLST analysis, 70 *P. aeruginosa* strains can be divided into 16 different sequence types (ST) with the prevalence of ST654 (35 isolates). Six samples characterized by new sequence types. All of the strains carried β-lactamase genes of PAO-type. 52% of strains possessed β-lactamase genes of VIM type, 20% - of OXA type. 77% of strains carried β-lactamase genes of several types simultaneously. All of the isolates examined had fosA gene responsible for fosfomicin resistance.

**Conclusion:** The data of whole genome analysis were consistent with the results of disc-diffusion method, especially for carbapenemases. Nonetheless, some cases of discrepancy were revealed. Phylogenetic analysis displayed high similarity groups consisted of strains isolated both from the same patient (different clinical sources) and from different patients. Thus, application of Next Generation Sequencing technologies allowed us to get the most complete information regarding the hospital strains studied . High-throughput sequencing can be used both to predict the sensitivity / resistance of microorganisms to antimicrobial drugs and for the epidemiological surveillance of *P. aeruginosa* strains.

**Disclosure of Interest**: None declared

### P80 BURKHOLDERIA CEPACIA COMPLEX (BCC) AMONG NON-CYSTIC FIBROSIS HOSPITALIZED PATIENTS IN KUWAIT

#### L. Vali, D. Al-Kayyali, A. A. Dashti

##### Medical Laboratory Sciences, KUWAIT UNIVERSITY, Kuwait, Kuwait

###### **Correspondence:** L. Vali

**Introduction:** *Burkholderia cepacia* complex (BCC) is a group of opportunistic pathogens that colonize in the lung of cystic fibrosis patients, however recently colonization and infections among hospitalized patients without cystic fibrosis and hospital outbreaks have been increasingly reported.

**Objectives:** In this study we characterized 8 BCC isolates from 7 non-cystic fribrosis patients from different sources of infection in Kuwait.

**Methods:** Isolates were identified to the species level by PCR-RFLP for *recA* gene and Multi-locus sequence typing (MLST). They were tested for antibiotic sensitivity using E-Test and agar dilution method. Pulsed-field Gel Electrophoresis (PFGE) was applied. Whole genome sequencing was performed for four of the isolates based on the antibiotic sensitivity results.

**Results:** Six solates were identified as *B*. *cenocepacia* (genomovar IIIA), 1 as *B. cepacia* (genomovar I) and 1 as *B. multivorans* (genomovar II). Results of PFGE using *Spe*I for *B*. *cenocepacia* illustrated diversity of these isolates. Three novel STs (ST-1282, ST-1284, ST-1288) and three novel allelic profiles for *gltB* (563)*, lepA* (523) and *gyrB* (846) for the newly identified ST 1288 were identified. MIC values showed all isolates were resistant to at least one group of antibiotics: ceftazidime (n=1), meropenem (n=2), minocycline (n=1), levofloxacin (n=1), and chloramphenicol (n=8) and one resistant to trimethoprim-sulfamethoxazole. Isolates 38 and 39 were obtained from one ICU patient diagnosed with Hypoxemic Respiratory Failure due to secondary pneumonia at two different time points before and after ceftazidime treatment. *B.cenocepacia* IIIA ST306 isolated from blood (isolate 38) was not resistant to ceftazidime but resistance was observed in the second blood specimen (isolate 39). Isolate 39 ultimately resulted in sepsis and death of the patient. WGS showed an allele difference in a polysaccharide capsule I gene (wcbT) putative acyl CoA transferase, which may cause an exclusion of the antibiotic (locus_tag=BCAL3218). Isolate 39 may have also survived the antibiotic treatment by orbE gene (ornibactin biosynthesis ABC transport protein).

**Conclusion:** This study confirms the ability of BCC to adapt to environmental changes and a potential problem for infection control team to eliminate BCC from hospital environment.

**Disclosure of Interest**: None declared

### P81 BURDEN OF MULTIDRUG-RESISTANT BACTERIA IN HOSPITAL ENVIRONMENT IN SLOVAKIA: RESULTS FROM THREE-YEAR MULTICENTRE PREVALENCE STUDY HOSPITAL-ENVIRO-REZ

#### L. Pazderka^1,2,3^, J. Brňová^1,2,4^, L. Michalikova^1,2,3^, S. Kissová^5^, A. Liskova^6^, S. Kucharíková^1^, J. Prnová^4,7^, P. Petruš^8^, V. Krčméry^6^

##### ^1^Department of Laboratory Medicine; ^2^Centre of Microbiology and Infection Prevention, Trnava University; ^3^Department of Clinical Microbiology, AnalytX s.r.o.; ^4^Department of Hospital Hygiene and Epidemiology, University Hospital Trnava, Trnava; ^5^Medirex; ^6^Department of Molecular Microbiology, St. Elisabeth University, Bratislava; ^7^Department of Public Health, Trnava University, Trnava; ^8^Clinic of Surgery and Transplant Center, Comenius University, Jessenius Faculty of Medicine in Martin, Martin, Slovakia

###### **Correspondence:** L. Pazderka

**Introduction:** The role of hospital environment in harboring and transmitting multidrug-resistant (MDR) organisms has become clearer due to a series of publications linking environmental contamination with increased risk of hospital-associated infections.

**Objectives:** The aim of this study was to assess burden of antibiotic resistance in bacteria isolated from hospital environment in Slovakia.

**Methods:** Three-year multicentre prevalence study was performed in January 2015 to December 2017. Clinically significant positive strains isolated from hospital environment of all Slovak hospitals were included. Antimicrobial susceptibility was determined using disc diffusion and colorimetric test (EUCAST). The presence of genes encoding multiresistance were performed by polymerase chain reaction and imunochromatographic assay. Statistical analyses were performed using R-project and P<0,05 was considered significant.

**Results:** Overall 2114 samples from hospital environment (Klebsiella spp., Pseudomonas spp., Escherichia coli, Enterobacter spp., Staphylococcus aureus and Enterococcus spp.) were analysed. Phenotype of MDR was confirmed in 492 (23,3%) isolates, and occurrence was similar in Gram-negative and Gram-positive bacteria (49,0% vs. 51,0%; P>0,05). MRSA and VRE from all Staphylococcus aureus and Enterococuss spp. positive swabs were found in 16,8,% and 5,8% respectively. None VRSA was isolated from hospital environment in Slovakia. Carbapenem and colistin resistant isolates were found in Gram-negative bacteria (985) in 6,1% and 2,5% respectively.

**Conclusion:** This is the first study concerning on burden of MDR bacteria in hospital environment in Slovakia. These results highlight the significance of better infection control practise and performing effective measures to reduce spreading of MDR bacteria within Slovakia health care facilities.

**This study was supported by a research grant from the MŠVVaŠ SR.**

**Disclosure of Interest**: None declared

### P82 EPIDEMIOLOGICAL ANALYSIS OF CONTACT PRECAUTIONS FOR MRDO´S IN A HOSPITAL IN SOUTHERN BRAZIL

#### C. C. Ponzi, N. V. Polezze, B. V. Biavatti, K. Catapan, L. J. C. de Mendonça

##### Health Care Associated Infections Control, Hospital Unimed Chapecó, Chapecó, Brazil

###### **Correspondence:** C. C. Ponzi

**Introduction:** MDRO colonization implies higher morbidity and hospital costs, and efforts to control cross-transmission must be persued.

**Objectives:** to describe the epidemiological characteristics of preemptive contact precautions (PCP) in a reference private hospital in Souther Brazil.

**Methods:** All patients with risk for colonization by MDRO´s (CDC criteria) are set to PCP at admission and oral, axillary and inguinal swabs are collected, and data was prospectivelly colected from Jan. 2015 to Dec. 2018 and analyzed by descriptive statistics and Fisher´s Exact Test

**Results:** 516 patients met criteria for PCP. 44,8% were transfered from a lower-complexity institution and 28,3% had more than 4 hospital stays in the previous 6 months. Most patients (63,2%) had positive result for MDRO (42,5% MRSA, 17,3% ESBL or KPC and 3,4% associated other Gram-negative MDRO and MRSA). Consireding data from the National Association of Private Hospitals in Brazil, patients colonized by MDRO had longer lenght of stay (p < 0,00001). In hospital all-causes mortality of MDRO colonized patients was 10%, compared to 5% of MDRO-negative patients.

**Conclusion:** Reference hospitals usually receive patients from smaller institutions, and the fact that most of the patietns were colonized by MDRO´s may imply lack of adequate infection control practices in those institutions. PCP, hand hygiene and rational use of antimicrobials must be strongly encouraged in all size health-care facilities.

**Disclosure of Interest**: None declared

### P83 DETECTIONS OF EXTENSIVELY DRUG-RESISTANT GRAM NEGATIVE BACILLI AMONGST IMMUNE-COMPROMISED PATIENTS IN A TERTIARY FEDERAL MEDICAL CENTRE IN THE NIGERIAN STATE OF KATSINA

#### I. Yusuf^1^, A. S. Kankara^2^

##### ^1^Microbiology, Bayero University, Kano, Kano; ^2^Microbiology, Federal Medical Center Katsina, Katsina, Nigeria

###### **Correspondence:** I. Yusuf

**Introduction:** The rate at which pathogenic Gram negative bacteria (GNB) are defying any form of treatment with different antibiotics is worrisome and need for the study arises.

**Objectives:** Study on occurrence of extensively drug resistant (XDR) GNB was conducted on immune compromised patients (ICP) in intensive care, accidents and emergency, and special care baby units as well as Obstetrics and Gynaecology, surgical and Medical wards of a tertiary medical centre in Nigerian state of Katsina, North West Nigeria.

**Methods:** The GNB isolated from urine, stool, sputum, wound swabs and catheter tips were tested for susceptibility to 12 categories of antimicrobials using disc diffusion method according to Clinical Laboratory Standards Institute (CLSI) 2018 break points.

**Results:** Out of 68 GNB isolated from 400 different clinical samples of ICPs, 15 (22.1%) exhibited extensively drug resistance. The majority of the XDR GNB were recovered from patients that had stayed on admission between the range of 0-9 days in the hospital. Variable resistances were expressed by *Escherichia coli* (37.5%), *Klebsiella pneumoniae* (60%) and 100% (n=1) by *Enterobacter spp.* against carbapenem (meropenem). Equally alarming is the resistance to colistin by *Escherichia coli 50%, Klebsiella pneumoniae 20%* as well as 75% resistance against Tigecycline expressed by *Escherichia coli* and 60% by *Klebsiella pneumoniae.* The isolates were phenotypically screened for ESBLs (53%), AmpC (13%), carbapenemase (0%) and metallo beta-lactamases (0%). Co- production of ESBL and AmpC were expressed by 25% *Escherichia coli* isolates. Piperacillin+Tazobactam antibiotic combinations with commonly prescribed antibiotics: ceftriaxone, ceftazidime, gentamicin, exhibited more synergistic performances than similar antibiotic combinations with amoxicillin.

**Conclusion:** Detection of XDR GNB among critically ill patients will have great impacts on patient care, infection control, and need to establish or resuscitate antibiotic stewardship in the facility is highly advised.

**References**

CLSI. Performance Standards for Antimicrobial Susceptibility Testing. 28th ed. CLSI supplement M100, Wayne, PA: Clinical and Laboratory Standards institute 2018.

**Disclosure of Interest**: None declared

### P84 CIPROFLOXACIN RESISTANCE IN SALMONELLA ENTERICA BLOODSTREAM INFECTIONS FROM HOSPITALIZED PATIENTS, CAMBODIA 2015-2018

#### S. Ho, S. Chea, S. Seng, S. Tuy, T. Sok, K. Hour, S. Thai, L. Eng

##### Laboratory, Calmette Hospital, Phnom Penh, Cambodia

###### **Correspondence:** S. Ho

**Introduction:** *Salmonella enterica* serovar Typhi (*Salmonella* Typhi) and *Salmonella enterica* serovar Paratyphi (*Salmonella* Paratyphi A, B, C) are among the most common bloodstream pathogens in low and middle income countries. In Cambodia, however, there is limited data on common serotypes and antibiotic susceptibility patterns.

**Objectives:** The study aims to identify the most common serotypes of *S. enterica* causing bloodstream infections in Cambodia as well as their antibiotic susceptibility patterns

**Methods:** We reviewed and analyzed hospital microbiology and antibiotic susceptibility data from 2015-2018.

**Results:** We found 166 cases of *Salmonella* infection, among which 125 (75%) were *S.* Paratyphi A, 25 (15%) were *S.* Typhi and 16 (10%) were other *Salmonella spp*. The most affected age groups were patients 20-29 years old (38.5%), 30-39 years old (18%), and 10-19 years old (9.6%). For *S.* Paratyphi A, 52% were resistant and 28.8% were intermediately susceptible to ciprofloxacin. *S.* Paratyphi A had very low resistance to commonly used antibiotics including Trimethoprim/sulfamethoxazole (0.8%), Amoxicillin (1.6%) and Chloramphenicol (0.8%). 28% of *S.* Typhi infections were resistant and 44% were intermediately susceptible to ciprofloxacin while higher was found for trimethoprim/sulfamethoxazole (60%), amoxicillin (64%) and chloramphenicol (52%).

**Conclusion:** Our study, in line with previous work, found that *S.* Paratyphi A was the most common serotype among *S.enterica* bloodstream infection in Cambodia, but we found very high ciprofloxacin resistance in *S.* Paratyphi A, while previous studies had shown low ciprofloxacin resistance. Antibiotic resistance in the second-most common serotype *S.* Typhi was also higher than that of *S.* Paratyphi A. This review contributes valuable clinical information for use in guiding physician decisions on antibiotic use, formulating national antibiotic guidelines, and educating the community on the prevention and control of salmonellosis.

*Keywords: Resistance, Salmonella, Serotype, Bloodstream infection, ciprofloxacin*

**Disclosure of Interest**: None declared

### P85 DRUG-RESISTANT BACTERIAL SEPTICAEMIA IN HOSPITALIZED PATIENTS, PHNOM PENH, CAMBODIA

#### L. Eng, S. H. Ho, S. Chea, T. Sok, S. Seng, S. Tuy, K. Hour, S. Thai, S. Ek, S. Bory, on behalf of Laboratory staffs and Infectious Diseases Team at Calmette hospital

##### Laboratory, Calmette Hospital, Phnom Penh, Cambodia

###### **Correspondence:** L. Eng

**Introduction:** Septicaemia is a leading cause of death in Intensive Care Units (ICUs). With more than 30 million cases and 6 million deaths worldwide per year, early detection and confirmation are critical to successful treatment while specific information on the situation Cambodia is lacking.

**Objectives:** The objectives of our study were to identify the common causes of septicaemia in the ICUs and on the General Medicine wards (GMWs) as well as to determine their antimicrobial resistance profiles.

**Methods:** We reviewed the laboratory results from the clinical laboratory of a tertiary care hospital in Phnom Penh, Cambodia from January to December 2018.

**Results:** From January to December 2018, bacterial septicaemia was confirmed in 25% (147/577) of ICU specimens and 18% (495/2751) of GMW specimens. In the ICUs, isolated species were *E. coli* 10%, *B. pseudomallei* (10%)*, E. faecium* (5%), *S. maltophilia* (5%), *K. pneumoniae* (3%), *B. cepacia* (3%), *A. xylosoxidans (3%)*, *Sphingomonas* spp. (3%)*, A. baumannii* (2%), and *S. aureus* (1%). In the GMWs, we found *E. coli (*11%), *S.* paratyphi A (10%)*, B. cepaciae* (8%), *S. maltophilia* (7%), *B. pseudomallei* (6%), *S. aureus* (4%), *A. xylosoxidans* (4%), and *K. pneumoniae* (1%).

Seven percent of *E. coli* spp. isolated from the ICUs were resistant to carbapenems, compared to 2% of those isolated from the GMWs, and 87% from the ICUs were resistant to fluoroquinolones compared to 62% from the GMWs. 100% of *S. aureus* in the ICUs and 65% in the GMW was methicillin-resistant, or MRSA. There was some resistance of *S.* paratyphi A to fluoroquinolones (37%) and trimethoprim-sulfamethoxazole (12%) in the GMWs, but all *S.* paratyphi A was sensitive to third generation cephalosporines. *A. baumannii* infections from the ICUs were all resistant to all beta-lactamines and fluoroquinolones.

**Conclusion:** Patients hospitalised in the ICUs have a higher chance of resistant bacterial blood infections that are than those in the GWMs. Our results should inform Cambodian physicians the different management for patients in the ICUs from those on the GMWs to improve clinical outcomes and infection control, and can be used to develop national and international policies and guidelines.

**Disclosure of Interest**: None declared

### P86 ANTIBACTERIAL RESISTANCE SURVEILLANCE OF CLINICAL ISOLATES AT AN ONCOLOGY CENTRE

#### V. Bhat^1^, R. Kelkar^2^, S. Biswas^2^, N. Khattry^3^, S. Gupta^3^

##### ^1^Microbiology, ACTREC, Tata Memorial Centre; ^2^Microbiology, Tata Memorial Centre; ^3^Medical Oncology, ACTREC, Tata Memorial Centre, Navi Mumbai, India

###### **Correspondence:** V. Bhat

**Introduction:** Increasing antibiotic resistance in bacterial pathogens is a major cause for concern in recent times. Regular Surveillance of antibiotic resistance helps in earlier detection of resistance trends.

**Objectives:** In this study, we present the results of bacterial antibiotic resistance surveillance in an oncology setting

**Methods:** This study includes bacterial isolates recovered from infections cancer patients in the year 2018. Samples included blood cultures, pus/wound swabs, urine, tissue biopsies, sterile body fluids and respiratory specimens. All samples were processed in the microbiology laboratory as per Standard Laboratory Protocols. Organisms were identified to species level and susceptibility testing was performed and interpreted as per Clinical laboratory Standards Institute (CLSI) guidelines. Antibiotic resistance percentages to individual organisms was computed.

**Results:** A total of 4728 blood cultures, 681 urine cultures, 987 pus/tissue/swabs, 73 sterile body fluids and 241 respiratory cultures were processed for bacterial cultures. Positive cultures yielded 299 Gram positive and 806 Gram negative isolates. *Escherichia coli*(263) was the commonest organism followed by *Klebsiella pneumonia*e(173), *Pseudomonas aeruginosa*(180), *Staphylococcus aureus*(97), E*nterococcus spp*(74) and *Acininetobacter*(46). Resistance of *K. pneumonia*e and *E. coli* to cefotaxime and ceftazidime was high at > 60%. Imipenem resistance was also high at >50% for both organisms. Resistance to piperacillin- tazobactam was seen in 36.1% of *E.coli* and 48.6% of *K.pneumoniae.* Resistance of *P. aeruginosa* to ceftazidime, piperacillin –tazobactam, amikacin and meropenem was 27.2, 25, 26.1 and 22.2 % respectively. Among *Acinetobacter spp*, resistance to cefotaxime, piperacillin-tazobactam, meropenem and amikacin was high at 78.2%, 60.9%, 52.1 and 67.4% respectively. Vancomycin resistance (VRE) was seen in 6.8% of *Enterococci.* Of the *S. aureus* isolates, methicillin resistance was seen in 38.1% (MRSA).

**Conclusion:** There is a high degree of antibiotic resistance among commonly isolated Gram negative bacteria such as *E.coli*, *K. pneumoniae* and *Acinetobacter spp* to cephalosporins, β- lactam/β-lactamase inhibitor combinations, fluoroquinolones and carbapenems in our setting.

**Disclosure of Interest**: None declared

### P87 SURVEILLANCE OF ANTIMICROBIAL RESISTANCE AT A UNIVERSITY HOSPITAL IN BENIN

#### T. A. Ahoyo^1^, S. ASSAVEDO^2^, P. D. FONTON^1^

##### ^1^BIOLOGIE HUMAINE, EPAC/UAC, ABOMEY CALAVI; ^2^MINISTERE SANTE, CABINET MINISTERE SANTE, COTONOU, Benin

###### **Correspondence:** T. A. Ahoyo

**Introduction:** Several antibiotics were routinely used in the treatment of healthcare associated infections, and may have contributed to the emergence of antibiotic-resistant strains in our context. Widespread resistance severely complicates management of infections

**Objectives:** we assess the activity of three antibiotics doxycycline, trimethoprim -sulfamethoxazole and meropenem during the management of infection control program.

**Methods:** Using standard microbiological methods for identification of microorganisms, all clinical bacterial isolates from inpatients received in hospital laboratory during an 18 months' period (december 2015 to Jun 2017) were recorded and analyzed. Antibiotics susceptibility was performed using agar disk diffusion method on Mueller Hinton. Mycobacteria and anaerobic bacteria were not included in the study.

**Results:** A total of 3478 bacterial isolates were collected of which 64% (n = 2226) were Gram negative (2005 Enterobactericea and others 221) and 36% (n = 1252) Gram-positive. The proportions of the strains varied depending on the types of infection. Almost 37% of the collected strains originated from Bloostream, followed by strains of venous and urinary catheter infection 35%, wound infection after clean surgy 10% and others 13%. A high proportion of strains showed resistance to doxycycline **60%**, trimetroprim/sulfamethoxazol **87%** and meropenem **7%**. No significant difference in the antibiotic resistance of the strains based on their origin. A disk diffusion method is not optimal for testing certain important resistance.

**Conclusion:** Reasonably priced antibiotics such as, doxycycline, trimethoprim/sulfamethoxazole are now of limited benefit in the treatment of Healthcare Associated Infections. The surveillance generate valuable information on antibiotics resistance, which can be used to prepare locally applicable recommendations.

**References**

Ahoyo T. A., Bankolé H., Adéoti F., Attolou A., Assavedo S., Amoussou-guenou M., Kindé-gazard D. and Pittet D. 2014 Prevalence of nosocomial infections and anti-infective therapy in Benin: results of the first nationwide survey in 2012 . Antimicrobial Resistance and Infection Control, 3:17http://www.aricjournal.com/content/3/1/17

**Keywords:** Pathogens, Health care Associated Infections, antibiotic resistance Benin

**Disclosure of Interest**: None declared

### P88 Withdrawn

### P89 Withdrawn

### P90 DISTRIBUTION OF BLATEM GENE AMONG ESCHERICHIA COLI STRAINS ISOLATED FROM DIFFERENT CLINICAL SAMPLES

#### S. T. Baban

##### Infection control and prevention, Surgical Specality Hospital, Erbil, Iraq

###### **Correspondence:** S. T. Baban

Emerging antibiotic resistance and Extended-Spectrum Beta-Lactamase (ESBL) producing *Escherichia coli* causing different nosocomial infections are rapidly increasing at alarming levels and it poses a major health burden in the 21^st^ century globally. The aim of this study is to determine the distribution of *bla*_TEM_ gene ESBL-producing *E. coli* from clinical specimens in hospitals in Erbil city. A total of 200 samples were collected from urine, human vaginal secretions and stool at all hospitals. The isolation and identification of *Escherichia coli* and antimicrobial susceptibility were performed by using Vitek compact system. Phenotypic screening of Extended Spectrum β-lactamase production in *E.coli* was confirmed by using both Double disk diffusion and Standard disk diffusion techniques. Moreover, PCR technique was used for genotypic detection of an ESBL gene *bla*_TEM_ according to the standard protocol. This study showed that 66.6% of *E. coli* isolates were identified as producing extended-spectrum β-lactamase enzymes isolated from different clinical specimens. The ESBL-producing E. coli isolates were detected using double disk synergy test (76.7%) in comparison to standard disk diffusion test (80%). Genotypic screening results confirmed that all ESBL-producing *E. coli* isolates (66.7%) were carried *bla*_TEM_ gene (700 bp) in clinical specimens (50% urine, 13.33% wound and 3.33% sputum). All ESBL-positive *E. coli* isolates showed high rates of susceptibility to Carbapenems antibiotic group including Imipenem (83.3%), Meropenem (81.7%), and Ertapenemes (80.0%). The increased prevalence of TEM β-lactamase gene in ESBL-producing *E. coli* observed in this study for the first time is considered as alarming because there is a limited treatment options remained for infections. Attempts to reduce the dissemination of multi-drug resistant E. coli through compliance with strict hospital infection control and prevention standards are imperative. Findings of this study may help clinicians selecting appropriate antimicrobial therapy in patients with different infections caused by ESBL-producing *E. coli*.

**Disclosure of Interest**: None declared

### P91 HIGH PREVALENCE OF PLASMID-MEDIATED QUINOLONE RESISTANCE DETERMINANTS IN ESCHERICHIA COLI IN CAMEROON

#### E. E. Lyonga Mbamyah^1^, M. Toukam^1^, C. Nkenfou^2^, A. M. Smith^3^, M. T. Mesembe^4^, H. K. Gonsu^5^, A. C. Betbeui^6^, A. B. Eyoh^4^, G. M. Ikomey^1^, S. Koulla-Shiro^1^

##### ^1^Faculty of Medicine and Biomedical Sciences, Department of Microbiology; ^2^Higher Teachers' Training College, University of Yaounde 1, Yaounde, Cameroon; ^3^Center for Enteric Diseases, National Institute for Communicable Diseases, Johanesburg, South Africa; ^4^CSCCD, FMBS; ^5^Faculty of Medicne and Biomedical Sciences, Department of Microbiology, University of Yaounde 1, Yaounde; ^6^Faculty of Science, University of Buea, Buea, Cameroon

###### **Correspondence:** E. E. Lyonga Mbamyah

**Introduction:** Plasmid carrying genes may contribute to the development of higher levels of fluoroquinolone resistance and may pose a threat by allowing the rapid spread of resistance among organisms.

**Objectives:** · Identify *E. coli* isolates and describe the antimicrobial resistance profile of these isolates.

· Detect the presence, prevalence and diversity of genes responsible for quinolone resistance among these *E. coli*

*·* Genotypically characterize selected plasmid mediated quinolone resistant (PMQR) *E. coli* strains.

**Methods:** Identification and antimicrobial susceptibility testing of *E. coli* species was done using VITEK 2. The detection, prevalence and diversity of plamid-mediated quinolone resistance (PMQR) genes were carried out using conventional PCR. Sequencing was done using the Applied Biosystem 3500 genetic analyser. DNA fingerprint was obtained using Pulsed-Field Gel electrophoresis.

**Results:** The prevalence *E. coli* was 178/440(39.5%). The resistance rates were: ampicillin 77.0%; amoxicillin/clavulanic acid 48.9%; CI=ciprofloxacin 39.7%; cefuroxime axetil 36.2%; cefuroxime 34.5%; gentamicin 30.5%; cefotaxime 28.7%; ceftazidime 24.7%; amikacin 13.8%; nitrofurantoin 10.1%; piperacillin/tazobactam 2.8%; and imipenem 1.1%. The detected plasmid mediated quinolone resistance (PMQR) genes were: *qnrA* 0; *qnrB* 4; *qnrS* 3: *Aac(6`)Ib-cr* 33 and *qepA,* 2. There were several mutations detected for the parC-*E. coli*. At position 80 serine (S) is replaced by Aspartic acid in strain K08 and asparagine in strain 402. All *E. coli* strains were non-human diarrhoeagenic strains. One pair of *E. coli* strains was 93.6% identical and another pair 92.7% similarity in the phylogenetic tree.

**Conclusion:** Fluoroquinolone resistance was high and the PMQR determinant causing this resistance was also high. To avoid the spreading of these PMQR determinants continuous surveillance of antimicrobial resistance should be carried out.

**Disclosure of Interest**: None declared

## Poster session: Antimicrobial use and stewardship 1

### P92 THE GLOBAL POINT PREVALENCE SURVEY OF ANTIMICROBIAL CONSUMPTION AND RESISTANCE (GLOBAL-PPS): RESULTS OF ANTIMICROBIAL PRESCRIBING FOR A GENERAL HOSPITAL IN URMIA, IRAN

#### C. Alinia^1^, S. Gheibi^2^, A. Versporten^3^, I. Pauwels^3^, H. Goossens^3^

##### ^1^Public health; ^2^Maternal and Childhood Obesity Research Center, Urmia University of Medical Sciences, Urmia, Iran, Islamic Republic Of; ^3^Laboratory of Medical Microbiology, Vaccine and Infectious Diseases Institute, University of Antwerp, Antwerp, Belgium

###### **Correspondence:** C. Alinia

**Introduction:** This study has done to monitor antimicrobial prescribing and resistance rates in Iran

**Objectives:** To measure the prevalence rate of antimicrobial prescription at hospital level

To address the quality of antibiotics prescription

**Methods:** A uniform and standardized method for surveillance of antimicrobial use in hospitals was used to assess the variation in antimicrobial prescribing in West Azerbaijan province, Iran. PPS was conducted in 2018, in child and neonatal wards in a teaching and general hospital. The survey included all inpatients receiving an antimicrobial on the day of the PPS. Data collected included details on the antimicrobial agents, reasons and indications for treatment as well as a set of quality indicators. A web-based application was used for data-entry, validation and reporting as designed by the University of Antwerp (www.global-pps.com). bioMérieux provided unrestricted funding support for the survey.

**Results:** The overall prevalence rate was achieved as 60.6%. Among the different specialized wards, the lowest and highest prevalence rates was related to Haematology−Oncology Medical (30%) and Neonatal Medical (77.8%) Wards, respectively. The most antibiotic use in Pediatric and neonatals wards were other beta-lactams and Penicillins, respectively. Pneumonia, prophylactic urinary tract infection, and prophylaxis of central nervous system infections, were the three most common diagnosis treated with therapeutic antibiotics. Overall, the reason for treatment was recorded in 84.97% of antimicrobial prescriptions, and a stop or review date in 3%. Local antibiotic guidelines were missing for 88.6% of the prescriptions. None of the patients received a targeted antibacterial treatment for systemic use.

**Conclusion:** The findings indicate that Iran has the highest prevalence rate of antibiotic prescription related to child and neonatal wards among all countries studied in Global PPS study. In addition to, the indicators confirmed the low quality of antibiotic prescription.

**Disclosure of Interest**: None declared

### P93 BITTER OR SWEET: THE INCREASING USAGE OF ANTIBIOTICS OVER THE GLOBE

#### H. K. Lam^1^, K. Rajwinder^2^

##### ^1^Clinical Operations, Matilda International Hospital, Hong Kong; ^2^41 mount kellett road, matilda international hospital, the peak, Hong Kong

###### **Correspondence:** H. K. Lam

**Introduction:** Antibiotic is a sugarcoating poison as it saves many lives from fatal infections. It can also be a potential bomb if it is being misused and will lead to the growth of antibiotic-resistance in human.

**Objectives:** To prevent most infections from being incurable in the future, our hospital has set up an Antibiotic Stewardship Programme (ASP) with a team to monitor the usage of Big Gun (BG) antibiotics. Our ASP team is composed of the executive medical director (EMD), infection control officer (ICO), pharmacist, infection control coordinator(ICC) with a microbiologist as our honorably consultant.

**Methods:** In hardware, we have a BG Prescribing form. In software, we have a computer system which audits on the usage of BG antibiotics with indication, patient’s diagnosis, medical history, culture result, inflammation markers recorded. Once a BG antibiotic is prescribed, pharmacist will attach the form together with MAR in the dispensing process. The physician has to complete the form before ICC’s collection who will then input the data into the system. Afterwards, an email will automatically be sent to the case physician and the ASP team. EMD and ICO act as auditors and will send feedback to the physician on the appropriateness of the prescription with reference to IMPACT, a local antibiotic guideline, and according to patient’s information given.

**Results:** From September 2018 to March 2019, 54 BG was antibiotics have been recorded. Most (44%) were prescribed by family doctors. Among the types of BG prescribed, Piperacillin & Tazobactam (25%) is mostly prescribed. Most indications are respiratory infection(36%) followed by orthopaedic(20%). The purpose of prescription are recorded as 55% empirical, 27% pathogenic and 18% prophylactic. 67% are regarded “appropriate”, with 24% “not-appropriate” and 9% “don’t know”. 9% of physicians replied disagree to the audit outcome with the remaining either agree or no comment.

**Conclusion:** Seeing the high prescription rate from family doctors, the issue has been discussed and evaluated in doctors’ meeting. Physicians are open to feedback the rational for their prescription if it is regarded as Not Appropriate or Don’t Know. The audit allows for discussions between the auditors prior to finalizing a ‘verdict’ which reduces the likelihood of wrongly affecting clinical practice. ASP raised physicians’ awareness and a decrease of BG prescription is noted.

**Disclosure of Interest**: None declared

### P94 POINT PREVALENCE STUDY OF ANTIBIOTIC COMPLIANCE ACROSS DISCIPLINES AT A TERTIARY MALAYSIAN HOSPITAL

#### M. K. Yin, C. L. Lau

##### Pharmacy, Universiti Kebangsaan Malaysia Medical Center, Kuala Lumpur, Malaysia

###### **Correspondence:** M. K. Yin

**Introduction:** Point prevalence study of antibiotics is the audit of judicious antibiotic use in accordance to local and national antibiotic guidelines. Inappropriate antibiotic use is associated with prolonged hospitalization, increased healthcare costs and development of antimicrobial resistance.

**Objectives:** To determine the rate of antibiotic compliance in accordance to local and national antibiotic guidelines across various disciplines in a tertiary hospital.

**Methods:** All antibiotic prescriptions from 43 wards of various disciplines were audited daily over a span of 2 weeks. Screening of prescriptions started at 8am using information obtained from case notes for indication and diagnosis pertaining to each antibiotic. Any discrepancies were discussed with the primary discipline team and infectious disease consultant. Data collected was then analyzed for compliance to local therapeutic guidelines, appropriateness of therapy in terms of dosage, spectrum of coverage, duration and choice by a team of pharmacists and infectious disease consultant.

**Results:** Of the 346 cases screened, obstetric and gynaecology was shown to have the lowest level of compliance to local guidelines for empirical antibiotic use (33.3%, n=15), followed by orthopedics (36.0%, n=25), surgery (42.4%, n=59), medical (52.6%, n=95), pediatrics (54.2%, n = 35), and intensive care (60.0%, n =5).

**Conclusion:** Compliance to local and national antibiotic guidelines was seen to be lowest among obstetricians for empirical use. Lack of up to date knowledge on antibiotic guidelines was seen as the possible barrier for appropriate antibiotic use.

**Disclosure of Interest**: None declared

### P95 POINT PREVALENCE SURVEY OF ANTIMICROBIAL USE IN A TERTIARY TEACHING HOSPITAL IN THE PHILIPPINES

#### A. F. G. Malundo^1^, F. A. R. Palabrica^1^, R. P. Berba^2^

##### ^1^Philippine General Hospital, Manila, Philippines; ^2^Hospital Infection Control Unit, Philippine General Hospital, Manila, Philippines

###### **Correspondence:** A. R. Palabrica

**Introduction:** The overuse and misuse of antimicrobials drives antimicrobial resistance.

**Objectives:** This study aimed to evaluate antimicrobial prescribing practices and determine areas for quality improvement.

**Methods:** A point prevalence survey of antimicrobial use among patients admitted in the Philippine General Hospital was conducted in 2017 and 2018 using the Global Point Prevalence Survey protocol. The study was conducted as part of a nationwide project headed by the Philippine Antimicrobial Point Prevalence Survey team. A descriptive analysis of the prevalence of antimicrobial use was reported along with relative frequencies of antimicrobials. Findings were compared using chi-square test.

**Results:** The prevalence of antimicrobial use was 49.83% and 47.73% in 2017 and 2018, respectively (*p=*0.21). A significant decline in antimicrobial use was noted in the neonatal and pediatric ICU (*p*<0.05), while usage increased in the neonatal medical ward *(p=*0.02). Antimicrobials were prescribed mostly for community-acquired infections, and as empiric therapy, with pneumonia being the most common infection requiring antimicrobials. About three fourths of antibiotics for surgical prophylaxis were given for >24 hours, with cefuroxime being the preferred agent. In 2017, cefuroxime was the most prescribed antibiotic, but was surpassed by meropenem in 2018. Broad-spectrum antibiotics were used often in critically-ill, and in medical patients.

Physician documentation of indication for antimicrobials improved from 68.80% in 2017 to 74.74% in 2018 (*p*<0.05). Guideline compliance increased from 63.50% to 74.96%, though not statistically significant (*p*=0.40). Nearly half of noncompliant prescriptions were for surgical prophylaxis. Documentation of antibiotic stop-date was not common practice, that is <30% in two years of survey.

**Conclusion:** Overall prevalence of antimicrobial use did not change from 2017 to 2018, however was evident in specific areas. Antimicrobial use varied depending on hospital area, patient profile, and indication. Interventions should focus on (1) improving physician awareness of guidelines, (2) education on appropriate surgical prophylaxis, (3) antibiotic deescalation, and (4) proper documentation.

**Disclosure of Interest**: None declared

### P96 HEALTHCARE ASSOCIATED INFECTIONS (HAI) AND ANTIBIOTIC USE SURVEY AT FOUR REGIONAL HOSPITALS IN SIERRA LEONE

#### A. Maruta^1^, H. Benya^2^, N. S. Kamara^3^

##### ^1^IPC, World health organisation, ^2^IPC, CDC, ^3^IPC, Ministry of Health and Sanitation, Freetown, Sierra Leone

###### **Correspondence:** A. Maruta

**Introduction:** Healthcare-associated infections (HAI) are a safety concern globally for both patients and healthcare providers. Healthcare-associated infections are a major threat for patient safety and the impact on health and well-being are longer duration of illness, longer treatment, higher mortality, treatment with expensive medicines, increased burden on the health system and huge economic impact. Surveillance of HAIs is an important infection control activity and also an indicator of quality patient outcomes. Knowledge of the prevalence of antibiotic resistance is a pre-requisite for infection prevention and control and is essential for healthcare policy makers to conduct effective responses.

**Objectives:** The main aim was to gather baseline data and information required for the development of an HAI and AMR strategy in Sierra Leone and to determine the prevalence of healthcare-associated infections

**Methods:** The point prevalence survey was conducted at four regional hospitals in Sierra Leone. A questionnaire-based survey was designed to collect information on the prevalence of HAI, antibiotic prescribing patterns and capacity of the health facility to promote an antibiotic stewardship program. The multi-disciplinary surveillance team included doctors, nurses, pharmacists and laboratory personnel. A questionnaire-based survey was designed to collect information on the prevalence of HAI, antibiotic prescribing patterns and capacity of the health facility to promote an antibiotic stewardship program.

**Results:** Data from 327 patients were collected out of 441 inpatients. About 114 patients were not included in the survey as they did not meet the inclusion criteria. The most common type of antibiotic prescribed was Ceftriaxone (54%) followed by Metronidazole (49%). Overall the prevalence of antibiotic use was 73.7% (95% CI: 69.3-77.7). Highest antibiotic use was in neonatal unit followed by ICU and paediatric wards across all hospitals

**Conclusion:** The survey has demonstrated that a point-prevalence survey methodology can be applied successfully to surveillance of HAI and antibiotic use across hospitals in Sierra Leone and the results can identify targets for patient safety and quality improvement.

**Disclosure of Interest**: None declared

### P97 EVALUATION OF ANTIBIOTIC PRESCRIPTION IN A GENERAL HOSPITAL OF DR CONGO: SPECIFIC CASE OF NYANKUNDE HOSPITAL

#### E. K. Kabululu^1^, K. Mutendela^2^, S. Linda^3^, M. Jeanne^4^, G. Ray-Barruel^5^

##### ^1^Infection control, CME Nyankunde, Beni, Congo, The Democratic Republic of the; ^2^Medecine interne, Hopitaux de lannemezan, lannemezan, France; ^3^communication, UCB, Bukavu; ^4^Gynecology, CME nyankunde, Beni, Congo, The Democratic Republic of the; ^5^Research, Griffith University, Bisbane, Australia

###### **Correspondence:** E. K. Kabululu

**Introduction:** Antibiotic overuse or misuse is one of the main antimicrobial drug prescription problems in low income countries, which leads to substantial modification of the bacterial ecology in health care facilities. In African hospitals, the list of essential antibiotics available is very restricted and contains less than ten antibiotics. However, resistance to older antibiotics is increasing and the development of new molecules has stalled.

**Objectives:** To monitor the using of antimicrobial in the D R C facility

**Methods:** We conducted a cross-sectional study to determine the prevalence of patients who received one or more antibiotics during their hospitalization. Data were collected on two different days in a 2-week period at Nyankunde Hospital, Beni city, in the province of North Kivu, DR Congo.

**Results:** In this study, the overall antibiotherapy rate was 43.2% (59/137 inpatients). Patients with antimicrobial therapy were aged between 4 months and 84 years; gender included 29 women and 30 men. Patients receiving antimicrobial therapy were admitted to different wards, as follows: Surgery (5%), Obstetrics (9%), Pediatrics (18%), Internal Medicine (30%), VIP ward (18%), Emergencies (11%) and Intensive care (6%). The common first diagnosis was sepsis syndrome (59%), and 47% of inpatients had also a second associated diagnosis. No bacterial culture tests were performed on any patient. However, 19% of patients had positive Widal Felix serodiagnosis testing and 15% had positive malaria rapid diagnostic tests (RDT). For those receiving single antibiotics (n=27), patients received mainly ceftriaxone (n=22, 37%) or ampicillin (n=20, 34%). Those receiving two antibiotics (n=32) usually had gentamicin (n=21, 35%) added, and for those who received three antibiotics (n=6) metronidazole (n=5, 8%) was the most common addition.

**Conclusion:** At this DR Congo hospital, 43.2% of inpatients had antibiotic therapy. The main reason was sepsis (proven or suspected), either as first or second syndrome. Microbial cultures are not feasible, and only RDTs of malaria and *Salmonella* infectious diseases are available. In these conditions, it is not possible to monitor antimicrobial resistance

**Disclosure of Interest**: None declared

### P98 EVALUATION OF INJECTABLE ANTIBIOTICS RECONSTITUTION AND ADMINISTRATION IN THE PEDIATRIC SERVICE OF GABRIEL TOURÉ UNIVERSITY HOSPITAL

#### L. Bengaly^1^, A. T. Traoré^2^, A. Fané^1^

##### ^1^Pharmacie, Hôpital Universitaire Gabriel Touré; ^2^Pharmacie, Hôpital du Mali, Bamako, Mali

###### **Correspondence:** L. Bengaly

**Introduction:** Bacterial resistance to antibiotics is a public health problem that spares no health system in the world. Antibiotics misuse in health facilities is one of the main factors behind the emergence of this antimicrobial resistance. Good reconstitution practices contribute to the proper use of injectable antibiotics in hospitals.

**Objectives:** The aim was to evaluate antibiotic dilution and administration techniques in the pediatric service and specifically to identify dilution solvents, their volumes and modes of administration.

**Methods:** The study was prospective and focused on children hospitalized in the pediatric service between March and September, 2016. Data were collected by direct observations of care practices associated with additional informations by interviewing care staff.

**Results:** A total of 2737 replenishment and administration sessions were collected. Distilled water was the most used solvent (87.9%) followed by sodium chloride 0.9% injectable solution (with 6.60%), glucose 5% injectable solution (2.10%), glucose 10%solution (with 1.7%) and sodium lactate, compound injectable solution (1.5%). Solvent volumes between 2.5ml and 10ml were the most used for reconstitution and were used for direct intravenous administration. The prescribed dilution volumes and dosages were respected in 77.34% of cases. Ceftriaxone was the most reconstituted antibiotic (65.07%) followed by amoxicillin (12.46%). Combinations of antibiotics or with other products in the same syringe have been reported (495 cases or 18.08%): ceftriaxone + gentamicin (96.16%) Ceftriaxone + Gentamicin + dexametaxone (2.22%) Cefotaxime + Gentamicin + Methylprednisolone (0.81%). Combinations of drugs in the same administration syringe were contrary to medical prescription guidelines. A single vial of solution (sodium chloride 0.9%, glucose 5% or glucose 10%) was used to reconstitute the several antibiotics for different children and was used by all teams with risks of contamination.

**Conclusion:** The shortcomings identified made it possible to formulate recommendations for better patient care, which will enhance the care quality.

**Disclosure of Interest**: None declared

### P99 PREVALENCE OF ANTIBIOTIC USE AND ADMINISTRATION AMONG HOSPITALIZED ADULT PATIENTS AT A TERTIARY CARE HOSPITAL IN KILIMANJARO TANZANIA

#### F. J. Muro^1,2,3^, F. Lyamuya^2,3,4^, R. Mallya^3,5^, B. Mmbaga^2,3,6,7^, G. Tillekeratne ^6^

##### ^1^Community Health, Kilimanjaro Christian Medical Centre; ^2^Faculty of Medicine, Kilimanjaro Christian Medical University College; ^3^Research, Kilimanjaro Clinical Research Institute; ^4^Faculty of Medicine; ^5^Maternal and Child Health, Kilimanjaro Christian Medical Centre, Moshi, Tanzania, United Republic of; ^6^Global Health, Duke Global Health Institute, Durham, United States; ^7^Paediatric, Kilimanjaro Christian Medical Centre, Moshi, Tanzania, United Republic of

###### **Correspondence:** F. J. Muro

**Introduction:** Antimicrobial stewardship programs (ASPs) have been shown to improve the appropriate use of antimicrobials, especially in high-income countries. However, ASPs are relatively less well implemented in low-or-middle income countries. To improve the effectiveness of ASPs in these settings, it is important to determine the core actions and targets for improving antimicrobial use.

**Objectives:** To describe the prevalence and patterns of antibiotic use at a tertiary care hospital in Tanzania.

**Methods:** Consecutive patients admitted to an adult medical ward at a tertiary care hospital, Kilimanjaro Christian Medical Centre in Moshi, Tanzania were enrolled from June 2018 to March 2019. The medical record was reviewed for data regarding antibiotics prescribed, indications for use, and microbiologic testing ordered.

**Results:** Of 1103 patient’s enrolled majority were males (663, 60.1%), with the median age being 54 years (IQR 39-70). About one-third (390, 35.4%) of the patients received antimicrobials during hospitalization, with pneumonia being the leading indication for antimicrobial use (158, 40.5%). Most commonly used antibiotics included ceftriaxone 285 (73.1%), metronidazole 155 (39.7%), and amoxicillin/ampicillin 46 (11.8%) patients. Median duration of antimicrobial use was 5 days (IQR 3-7). Few patients on antimicrobials (27, 6.9%) had culture results, of which half (15, 55.6%) were positive for an organism and a minority (8, 29.6%) were susceptible to the antibiotics being used. Overall mortality in the cohort was 22.7% and median duration of hospitalization was 5 days (IQR 3-8).

**Conclusion:** Antibiotics were used in a substantial proportion of admitted patients. However, in most cases, treatment was empirical with limited use of culture results. Future ASP efforts can target the improved use of microbiologic cultures to target antimicrobial use.

**Disclosure of Interest**: None declared

### P100 THE GLOBAL POINT PREVALENCE SURVEY OF ANTIMICROBIAL CONSUMPTION AND RESISTANCE (GLOBAL-PPS) IN TOGO

#### M. Salou^1^, F. Nounhou^1^, A. Versporten^2^, I. Pauwels^2^, K. D. Ekouevi^3^, H. Goossens^2^, A. Y. Dagnra^1^

##### ^1^Laboratory of Medical Microbiology, University of Lomé, Lomé, Togo; ^2^Laboratory of Medical Microbiology, University of Antwerp, Antwerp, Belgium; ^3^Public health department, University of Lomé, Lomé, Togo

###### **Correspondence:** A. Versporten

**Introduction:** The Global-PPS is a standardized tool to assess antimicrobial use (AMU) and resistance and helps to establish antibiotic stewardship programs.

**Objectives:** We aim to report results of the survey in 9 hospitals in Togo.

**Methods:** The standardized Global-PPS method was used to assess variation in antimicrobial prescribing in 9 hospitals in December 2018. The survey included all inpatients receiving an antimicrobial on the day of the PPS. Data included details on antimicrobial agents, reasons and indications for treatment and a set of quality indicators. bioMérieux provided unrestricted funding support for the survey.

**Results:** Of the 9 hospitals, 2 were tertiary care hospitals. The survey included 713 patients of which 89.8% were treated with at least one antimicrobial. Top 3 indications for AMU were malaria (32.2%), other undefined (17%) and gastro-intestinal infections (6.3%). Out of 1062 antimicrobials, 16.9% were antimalarials and 74.7% antibacterials for systemic use of which ceftriaxone (27.2%), amoxicillin (17.8%) and metronidazole (11.7%) were most frequent prescribed. Therapeutic prescribing (n=716) accounted for 67.4% of which 95.8% for a community and 4.2% for a hospital acquired infection. Antimicrobials used for medical or surgical prophylaxis (SP) accounted for 13.8% and 17.4%. Ceftriaxone, ciprofloxacin and amoxicillin were most often prescribed for SP (30.2%, 19.2%, 17.4% respectively). Prolonged SP (≥1 day) in adults and children was common (69%). The reason for antimicrobial prescription was documented in 83.3% of cases while a stop/review date was only documented in 25.0% of cases. Guideline compliance reached 98.1%. No patients were reported to have received a microbiology−based treatment.

**Conclusion:** This survey was the first conducted in the country. It is important setting-up a tailored antimicrobial stewardship program in each hospital. The challenge remains reinforcement of infection prevention and the medical bacteriology lab capacity by offering antimicrobial susceptibility testing to monitor prescription.

**Disclosure of Interest**: None declared

### P101 THE GLOBAL POINT PREVALENCE SURVEY OF ANTIMICROBIAL CONSUMPTION AND RESISTANCE (GLOBAL-PPS) IN BURKINA FASO

#### A.-S. Ouedraogo^1^, A. Versporten^2^, A. Nagalo^3^, I. Pauwels^2^, H. Goossens^2^, A. Ouedraogo^3^, A. Poda^4^

##### ^1^Department of Medical Bacteriology and Virology Souro Sanou Teaching Hospital, Bobo-Dioulasso, Burkina Faso, Bobo Dioulasso, Burkina Faso; ^2^Laboratory of Medical Microbiology, University of Antwerp, Antwerp, Belgium; ^3^Souro Sanou Teaching Hospital, Bobo-Dioulasso, Burkina Faso; ^4^1Souro Sanou Teaching Hospital, Bobo-Dioulasso, Burkina Faso , Bobo Dioulasso, Burkina Faso

###### **Correspondence:** A.-S. Ouedraogo

**Introduction:** Burkina Faso experiences difficulties in the management of increased antimicrobial resistance.

**Objectives:** We aimed to assess for the first time, antimicrobial use in hospitals in Burkina Faso.

**Methods:** The standardized Global-PPS method assessed antimicrobial prescribing in 7 hospitals in Burkina Faso in February-March 2019. The survey included all inpatients receiving an antimicrobial on the day of the PPS. Data included details on the antimicrobial agents, reasons and indications as well as a set of quality indicators. bioMérieux provided unrestricted funding support for the survey.

**Results:** The survey included 1190 inpatients. Overall antimicrobial (AM) prescription rate was 71.8% with highest rates found in newborn wards (89.2%). Prophylaxis in neonates (12.4%) and digestive tract infections (11.9%) were the most common indications. Out of 1080 AMs, systemic antibacterials (78.9% of which 26.8% ceftriaxone), antiparasitics (13.4% of which 9.5% artesunate), and antimycobacterials (3.2%) were the most prescribed antimicrobial classes. Community acquired infections were common (85.2%). Healthcare-associated infections accounted for less than 5% of antimicrobials and included mainly surgical site infections (55.6%) and infections on invasive materials (27.8%). Surgical prophylaxis (n=62 antibiotics; 5.7%) lasted mostly for more than 2 days (87.1%). The reason for the antimicrobial prescription was documented in 82% of cases, but this corresponded only half to national or international guidelines (52.9%). A stop/review date was rarely reported (6.2%). Most antimicrobials were prescribed empirically (99%). Antimicrobial therapy was oriented towards ESBL (3 cases/7), MRSA (2cases/7) and non-fermenter gram negative bacilli producer of ESBL (1case/7) only.

**Conclusion:** Antimicrobial prevalence in hospitals in Burkina Faso is high. Continued education of caregivers on rational antimicrobial use and improved access to microbiological investigations is needed. We will expand the study to all health regions to provide a picture of the national situation and needs.

**Disclosure of Interest**: None declared

### P102 THE GLOBAL POINT PREVALENCE SURVEY OF ANTIMICROBIAL CONSUMPTION AND RESISTANCE: 2018-2019 RESULTS FOR HOSPITAL-ACQUIRED PNEUMONIA IN 8 MEXICAN HOSPITALS

#### E. Gonzalez-Diaz^1^, A. Versporten^2^, S. Loza^3^, J. Corona^4^, I. Pauwels^2^, J. Araujo^5^, D. Basurto^6^, N. Hernández^7^, M. Almaraz^8^, J. Molina^9^, D. Torres^10^, H. Goossens^2^

##### ^1^Unidad de Vigilancia Epidemiologica Hospitalaria & Medicina Preventiva, Hospital Civil de Guadalajara Fray Antonio Alcalde, Guadalajara, Mexico; ^2^Laboratory of Medical Microbiology, Vaccine and Infectious Diseases Institute, University of Antwerp, Antwerp, Belgium; ^3^Hospital de Especialidades CMN Siglo XXI , IMSS, Mexico City; ^4^Hospital Angeles del Carmen, Guadalajara; ^5^Hospital Central Dr. Morones Prieto, San Luis Potosí; ^6^Hospital General Regional 200 IMSS, Estado de Mexico; ^7^bioMerieux, Guadalajara; ^8^HGR 1 IMSS Dr. Carlos McGregor Sánchez, HGR 1 IMSS Dr. Carlos McGregor Sánchez, Ciudad de Mexico; ^9^Hospital Cardiología IMSS, Monterrey; ^10^HR de Alta Especialidad de la Península de Yucatán, Mérida, Mexico

###### **Correspondence:** I. Pauwels

**Introduction:** Despite advances in HAP and VAP, they continue to account for 22% of all HAIs. In an effort to minimize patient harm and unnecessary antibiotics and reduce antibiotic resistance development a recommendation is generate local antibiograms to guide healthcare professionals to the optimal choice of antibiotics and antimicrobial stewardships programs be implemented.

**Objectives:** The Global-PPS (www.global-pps.com) [G-PPS] aims to assess variation of antimicrobial prescribing worldwide. We describe antibiotic treatment for Hospital-Acquired Pneumonia (HAP) in Mexico.

**Methods:** A standardized surveillance of antimicrobial use assessed HAP prevalence and variation in antimicrobial therapy in 8 Mexican hospitals. The G-PPS was conducted from May 2018 and April 2019, in 6 tertiary and 2 secondary care hospitals. Data collected included antimicrobials, indications for treatment and quality indicators.

**Results:** The G-PPS included 2,181 patients on antimicrobials, 1,930 on adult, 193 on pediatric and 58 on neonatal wards**.**Of all treated adults, 315 patients (16.3%) were diagnosed with pneumonia, 57.5% (n=181) had HAP.For adult HAP patients, 36.5%of prescriptions were targeted, of which 49.1% against at least one multidrug resistant organism (MDRO). Of all targeted prescriptions, 37% were targeting ESBL Gram-negatives and 18.5% targeted other MDROs. In the adult ward,reason for prescription was documented in 95.6% of cases and 83%complied with local guidelines. Biomarkers were used to progress treatment in 41.4% of cases. Overall, the most commonly prescribed antibiotics were meropenem (23%), linezolid (8.8%) and vancomycin (7.4%).

**Conclusion:** These finding show the need to implement antimicrobial stewardship in all hospitals to improve antibiotic prescribing by reducing carbapenem use, enhancing compliance to guidelines and performing post-prescription review.

**Disclosure of Interest**: None declared

### P103 ANTIBIOTIC USE TRENDS IN A PEDIATRIC HEART SURGERY HOSPITAL IN GUATEMALA

#### H. G. Maldonado^1^, J. Guerra^1^, G. Calvimontes^2^, R. Mack^3^, J. Barnoya^4^

##### ^1^Control y Prevención de Infecciones Intrahospitalarias; ^2^Departamento de Cirugía Cardiovascular Pediátrica y Cardiología Pediátrica “Dr. Aldo Castañeda”; ^3^Unidad de Terapia Intensiva Cardiovascular; ^4^Departamento de Investigación, Unidad de Cirugía Cardiovascular de Guatemala, Guatemala, Guatemala

###### **Correspondence:** H. G. Maldonado

**Introduction:** Hospitalized children often receive inappropriate antibiotics. However, scant data are available on antibiotic use in congenital heart surgery (CHS), particularly from low-middle income countries (LMICs).

**Objectives:** We assessed antibiotic use trends in patients undergoing CHS in Guatemala, a LMIC.

**Methods:** From Jan 2015 to Mar 2019 we measured days of therapy (DOT), length of treatment (LOT), and antibiotic-free days (AFD). All rates were adjusted to 1000 patient-days. Trends for DOT rate per antibiotic family type and quarterly series were analyzed using Pearson correlation. Analyses were done in STATA.

**Results:** 1335 children underwent to CHS, using a total of 11351 days of antibiotic therapy in 14172 patient-days. Mean rates were 801 DOT, 477 LOT, and 523 AFD per 1000 patient-days. DOT (r = -0.25, p= 0.32), and LOT (r = -0.49, p= 0.04) rates showed a downward trend. The observed mean DOT rates by antibiotic family per 1000 patient-days were: 167 for anti-pseudomonal penicillins, 148 carbapenems, 136 vancomycin, 85 amynoglucosides, 84 cephalosporins, 44 fluoroquinolones, and 32 fosfomycin. A downward trend was observed for piperacillin-tazobactam (r = -0.50, p = 0.03), aminoglycosides (r = -0.48, p = 0.049) and fluoroquinolones (r = -0.41, p = 0.04). An upward trend was observed for cephalosporins (r = 0.54, p = 0.02), and fosfomycin (r = 0.6, p = 0.01). Carbapenems (r = -0.12, p = 0.63), and vancomycin (r = 0.07, p = 0.78) maintained a stationary trend.

**Conclusion:** Our findings suggest that DOT rates are similar in Guatemala to those reported in high-income countries, however carbapenems and vancomycin had greater DOT rates. Interventions are needed in order to decrease inappropriate antibiotic prescription in this resource-limited setting.

**Disclosure of Interest**: None declared

### P104 QUANTITY AND QUALITY OF ANTIBIOTIC PRESCRIBING FOR SEPSIS IN HOSPITALISED ADULTS: RESULTS OF THE 2015, 2017 AND 2018 GLOBAL POINT PREVALENCE SURVEY OF ANTIMICROBIAL CONSUMPTION AND RESISTANCE (GLOBAL-PPS)

#### I. Pauwels^1^, A. Versporten^1^, S. Le Page^2^, H. Goossens^1^, on behalf of the Global-PPS network

##### ^1^Laboratory of Medical Microbiology, University of Antwerp, Wilrijk, Belgium; ^2^bioMérieux, Marcy l’Etoile, France

###### **Correspondence:** I. Pauwels

**Introduction:** Sepsis is a severe condition, requiring rapid initiation of antimicrobial therapy.

**Objectives:** We aimed to describe the quantity and quality of hospital antibiotic prescribing in adult sepsis patients in five continental regions.

**Methods:** The Global-PPS assessed hospital antimicrobial prescribing patterns using a standardised method. A total of 662 hospitals in 67 countries participated in a PPS of antimicrobial use at least once in 2015, 2017 or 2018. We descriptively analysed data on adult patients receiving a systemic antibiotic (ATC J01) on the day of the survey. bioMérieux provided unrestricted funding support for the survey.

**Results:** Of 73,080 adults on systemic antibiotics, 2.6% were being treated for sepsis, 51.7% of which were on antibiotics for a healthcare-associated (HA) sepsis, ranging from 38.5% in Africa to 77% in South America. Overall, 21.7% of HA sepsis cases were related to the use of invasive devices. Microbiological results were used to inform treatment in 26.7% of prescriptions. Of all patients, 69.3% were on single-agent therapy, ranging from 48.7% in South America to 80.4% in Europe. A penicillin/β-lactamase-inhibitor was the most common regimen (25.7%), followed by a carbapenem (12.1%) and a 3^rd^ generation cephalosporin (10.1%). Overall, 12.1% of sepsis patients were being treated with one or more Reserve antibiotic, ranging from 7.7% in Africa to 25.6% in South America. Stop/review date and reason for treatment were documented in 35.9% and 80.7% of prescriptions respectively. Guideline compliance was reported to be up to 79.8%, yet guidelines were missing in 22.9% of prescriptions.

**Conclusion:** These data illustrate challenges related to antibiotic prescribing for sepsis patients, such as the use of broad-spectrum agents, low documentation of stop/review date, sub-optimal use of microbiology to inform treatment and a high proportion of Reserve prescribing. The use of Reserve antibiotics was particularly high in South America, with 1 in 4 sepsis patients exposed to these last-resort drugs. PPS results can support local stewardship teams in designing contextualised interventions, even for critical conditions such as sepsis.

**Disclosure of Interest**: None declared

### P105 POINT PREVALENCE SURVEY OF ANTIMICROBIAL USE AND RESISTANCE IN THE INTENSIVE CARE UNITS OF GEORGIA

#### M. Tsereteli^1,2^, G. Chakhunashvili^2^, O. Tsagareishvili^2^, D. Tsereteli^2^

##### ^1^High Technology Medical Center, University Clinic; ^2^Communicable Disease, National Center for Disease Control and Public Health, Tbilisi, Georgia

###### **Correspondence:** M. Tsereteli

**Introduction:** Antimicrobial resistance (AMR) is predominantly due to inappropriate use of antibiotics, so it is mandatory for healthcare facilities to monitor antimicrobial consumption.

**Objectives:** Assessment of antibiotic use and AMR in intensive care units (ICUs) of Georgian hospitals through point-prevalence survey.

**Methods:** Point Prevalence Survey (PPS) of Antimicrobial Consumption and Resistance was conducted according to the Global-PPS protocol in ICUs of 10 multi-profile hospitals of three biggest cities of Georgia (Tbilisi, Kutaisi, and Batumi). PPS was performed in June, 2018.

**Results:** On the day of the survey, total number of beds at the ICUs was 176, with 119 patients (bed occupancy percentage of 67.6%).

Antibiotics were administered to 74 patients (62.2%). 46 (62.2%) patients received empirical antibiotic therapy. Among them, 16 (34.8%) had administered two or more antibiotics. Empirically, the following antibiotics were used most frequently: Ceftriaxone – 17 (37.0%), Vancomycin – 8 (17.4%) and Carbapenems 6 (13.0%).

Only 28 (37.8%) patients have received etiotropic antibiotics therapy, moreover, 15 (53.8%) patients have received it with two or more antibiotics.

Healthcare-associated infections were seen in 34 patients (28.6% of ICU patients), including: ventilator-associated pneumonia - 22 cases (64.7% of all nosocomial infections), surgical site infection - 7 cases (20.6%), catheter-associated bloodstream infection – 2 cases (5.9%), catheter-associated urinary tract infection – 2 cases (5.9%), *C. difficile*-associated infection - 1 case (2.9%).

Among enrolled patients, following multi-resistant strains were isolated and identified from clinical samples: 5 ESBL-producing non-fermenting gram negative bacilli, 4 ESBL-producing *Enterobacteriaceae*, 3 carbapenem-resistant *Enterobacteriaceae*, 3 carbapenem-resistant non-fermenting gram negative bacilli, 1 methicillin-resistant *Staphylococcus aureus*.

**Conclusion:** PPS revealed the necessity of interventions in several directions in order to improve quality of antibiotic prescriptions. In the future PPS can be used as a forceful tool to monitor appropriate use of antibiotics.

**Disclosure of Interest**: None declared

### P106 THE GLOBAL POINT PREVALENCE SURVEY OF ANTIMICROBIAL CONSUMPTION AND RESISTANCE (GLOBAL-PPS): 2015, 2017 AND 2018 RESULTS OF ANTIBIOTIC PRESCRIBING FOR PNEUMONIA IN GEORGIA

#### I. Korinteli, I. Pauwels^2^, A. Versporten^2^, H. Goossens^2^, H. Phagava^1^, K. Pagava^1^

##### ^1^Tbilisi state medical university, Tbilisi, Georgia; ^2^University of Antwerp, Antwerp, Belgium

###### **Correspondence:** I. Korinteli

**Introduction:** Pneumonia is a frequent reason for antibiotic use and a common cause for hospitalisation, both in children and adults.

**Objectives:** We aimed to assess antibiotic prescribing patterns for pneumonia in Georgian hospitals to identify potential targets for antimicrobial stewardship.

**Methods:** The Global-PPS was conducted in 18 Georgian hospitals in 2015, 2017 and 2018. The survey included all inpatients receiving an antimicrobial on the day of PPS. Data included details on antimicrobial agents, reasons and indications for treatment and a set of quality indicators.

**Results:** In total 79 wards with 895 inpatients were surveyed of which 77.8% were admitted to hospitals in Tbilisi. Of all patients on antibiotics, 29.8% were treated for pneumonia, with the highest rates on paediatric intensive care units (18.9%) and paediatric medical wards (45.3%). Up to 98.6% of pneumonia, cases were community-acquired infections (CAI). Regarding the antimicrobial quality indicators, documentation of the reason of prescription was 100%, yet the stop/review date was missing in 93.1% of cases. Overall compliance to antibiotic guidelines was 91.9% and treatment was mostly empiric (87.0%). Of targeted prescriptions, 75.0% was for treatment of MRSA. CRP was used in the decision to treat in 98.1% of patients. CRP levels in blood were 123 mg/L on average. For adults, commonly prescribed antibacterial drugs for systemic use (ATC J01) were ceftriaxone (26.4%), followed by cefepime (10.7%) and meropenem (9.1%). In paediatric and neonatal units, the top 3 consisted of ceftriaxone (31.0%) ampicillin/enzyme inhibitor (25.4%) and meropenem (14.3%).

**Conclusion:** The high proportion of CAI pneumonia, empirical prescribing and the use of broad-spectrum antibiotics are worrisome findings. In addition, documentation of the stop/review date is remarkably low, yet high use of CRP in the decision to treat indicates an attempt to rationalise antibiotic use. For optimisation it is essential to plan specific stewardship interventions such as introduction of a cut off policy after 72 hours, increase of targeted treatment and availability of updated guidelines.

**Disclosure of Interest**: None declared

### P107 ASSESSING THE LEARNING NEEDS AND BARRIERS FOR IMPLEMENTATION OF ANTIMICROBIAL STEWARDSHIP IN HOSPITALS THAT HAVE PARTICIPATED IN THE GLOBAL POINT PREVALENCE SURVEY ON ANTIMICROBIAL CONSUMPTION AND RESISTANCE (GLOBAL-PPS)

#### I. Pauwels^1^, A. Versporten^1^, E. Vlieghe^2^, H. Goossens^1^

##### ^1^Laboratory of Medical Microbiology, University of Antwerp, Wilrijk; ^2^Department of General Internal Medicine, Infectious Diseases and Tropical Medicine, University Hospital Antwerp, Edegem, Belgium

###### **Correspondence:** I. Pauwels

**Introduction:** Point prevalence surveys (PPS) have proven to be instrumental in informing antimicrobial stewardship (AMS) activities, yet translating PPS findings into contextualised interventions can be challenging.

**Objectives:** We aim to evaluate the impact of the Global-PPS on local AMS programmes and assess health care professionals’ educational needs and barriers for implementing AMS.

**Methods:** An internet-based survey containing 24 questions was disseminated within the Global-PPS network, including contacts from participating and non-participating hospitals. Responses were collected from February up to May 2019 and were descriptively analysed.

**Results:** A total of 250 respondents from 73 different countries completed the survey, 198 of which were Global-PPS participants. Up to 70% (n=176) of responses were from low-and middle-income (LMIC) countries. When asked which AMS components were present in their hospital, 69.2% (HIC 89.2%; LMIC 60.8%) replied they had local prescribing guidelines and 50% (HIC 70.3%; LMIC 41.5%) reported the presence of an AMS committee. Of the Global-PPS participants who reported AMS activities (n=187), 69% stated that one or more of those activities was initiated as a result of PPS findings. Prolonged surgical antibiotic prophylaxis was the most common (63.6%; n=126) target for improvement, identified from Global-PPS findings. Out of 18 possible barriers, a lack of time was most commonly (33.8%) reported as the number one barrier for implementation of AMS in HIC, as compared to a lack of cooperation from prescribers (13.1%), time (12.5%) and qualified personnel (12.5%) in LMIC. Overall learning needs were: skills to optimise therapeutic (62%) and prophylactic antibiotic use (54.8%), followed by the design of PPS-based interventions (52.7%) in HIC and the management of multidrug-resistant infections (44.3%) in LMIC.

**Conclusion:** These results will inform the development of a dedicated e-learning course, targeting Global-PPS participants worldwide and focused at the translation of PPS-findings into locally tailored AMS interventions, thus contributing to a sustained response to AMR in hospitals worldwide.

**Disclosure of Interest**: None declared

### P108 SURVEILLANCE OF ANTIMICROBIAL STEWARDSHIP PROGRAM AND ANTIMICROBIAL CONSUMPTION: PILOT SURVEILLANCE OF JAPAN SURVEILLANCE FOR INFECTION PREVENTION AND HEALTHCARE EPIDEMIOLOGY (J-SIPHE)

#### M. Endo^1^, K. Hayakawa^1,2^, T. Tajima^1^, K. Suzuki^1^, T. Suzuki^1,2^, S. Tsuzuki^1^, N. Matsunaga^1^, N. Ohmagari,^1,2^

##### ^1^AMR Clinical Reference Center; ^2^Disease Control and Prevention Center, National Center for Global Health and Medicine Hospital, Tokyo, Japan

###### **Correspondence:** M. Endo

**Introduction:** We constructed J-SIPHE system to facilitate AMR measures in Japan. Along with other AMR related parameters, J-SIPHE collects data on antimicrobial stewardship program (ASP) as well as antimicrobial use (AMU) at healthcare facilities.

**Objectives:** We evaluated the data of ASP and AMU during the J-SIPHE pilot surveillance period.

**Methods:** From April to November 2018, 28 facilities provided data on ASP and AMU. AMU data (such as Antimicrobial Use Density [AUD] and Days of Therapy [DOT]) can be registered semi-automatically using the medical fee statement (receipt) file at each facility. Both AMU and ASP data (such as available antibiotics and ASP practice) were registered by the online surveillance system. ASP data were required for targeted antibiotics classes (anti-*P. aeruginosa* [anti-PA] penicillins/cephalosporins, carbapenems, anti-MRSA agents, fluoroquinolones, colistin, tigecycline, antifungal agents).

**Results:** The median number of beds at participating facilities were 396 (IQR: 309–496) and the median average hospital stay was 12.3 days (IQR: 9.9–14.6). Carbapenems and linezolid were the most commonly targeted antibiotics by ASP (carbapenems: 15 [53.6%] required notification [RN], 13 [46.4%] RN and prospective audit and feedback [PAF]; linezolid: 13 [46.4%] RN, 11 [39.3%] RN and PAF, 4 [14.3%] pre-authorization), followed by other anti-MRSA agents. Among ASP targeted antibiotics, anti-PA penicillin was the most prescribed (median AUD: 2.4 [IQR: 1.7–2.7], median DOT: 3.1 [IQR: 2.4–3.7]), followed by carbapenems (median AUD: 2.2 [IQR: 1.7–2.9], median DOT: 2.3 [IQR: 1.9–3.9]). For anti-MRSA agents, glycopeptides (median AUD: 0.7 [IQR: 0.4–1.2], median DOT: 1.0 [0.6–1.7]) were mainly used.

**Conclusion:** Our system combined ASP practice with semi-automated AMU surveillance enabling comparison among facilities and monitoring trends of ASP targeted antibiotics. Widespread use of J-SIPHE could provide useful information on the current status of ASP/AMU and on high priority targets of ASP in Japan.

**Disclosure of Interest**: None declared

### P109 INFLUENCE OF ANTIMICROBIAL STEWARDSHIP IN THE ADEQUACY OF ANTIMICROBIAL PRESCRIPTION - A CROSS-SECTIONAL SURVEY

#### A. Silva-Pinto, R. Duro, N. Rocha-Pereira, P. Andrade, J. Mourato-Torres, C. Lima-Alves

##### Infection Control and Antimicrobial Stewardship Unit, CENTRO HOSPITALAR UNIVERSITÁRIO SÃO JOÃO, Porto, Portugal

###### **Correspondence:** A. Silva-Pinto

**Introduction:** Antimicrobial stewardship is a multimodal strategy to optimize adequacy of antimicrobial prescription, crucial in the approach to decrease antimicrobial resistance.

**Objectives:** To assess the adequacy of antimicrobial prescription in a university hospital and to compare the adequacy in departments with or without antimicrobial stewardship interventions.

**Methods:** We performed a cross-sectional study based on the point prevalence survey rules from WHO. One third of the patients in each department were randomly selected. After selection, we classified the adequacy of each antimicrobial prescribed (considering necessity of treatment, spectrum, dose, administration route and duration of therapy), according to local recommendations and, in its absence, national/international guidelines. We compared antimicrobial prescription adequacy in departments with specific ongoing antimicrobial stewardship interventions (Cardiothoracic Surgery, Haematology, Orthopaedics, Plastic Surgery, Urology, Vascular Surgery) and those without such interventions.

**Results:** On the day of the study, 898 patients were hospitalized. We randomly included 316 (35%). Of those, 119 had at least one antimicrobial prescription (37,7%), corresponding to 172 antimicrobials (33 (19,2%) were prescribed as prophylaxis and 139 (80,8%) as treatment). Table 1 depicts some variables of interest. We considered antimicrobial prescription as adequate in 107 patients (62,2%). Comparing departments with and without antimicrobial stewardship interventions, we noticed a statistically significant difference: 76,6% vs. 56,8% (p=0,021; Chi Squared test).

**Conclusion:** Antimicrobial stewardship is a multimodal strategy with a positive impact in the adequacy of antimicrobial prescriptions.

**Disclosure of Interest**: None declared

### P110 SECONDARY ANALYSIS OF THE HALT STUDY DATA IRELAND

#### A. Vellinga^1^, K. Burns^2^, H. Murphy^2^, M. Tandan^1^

##### ^1^National University of Ireland, Galway, Galway; ^2^Health Protection and Surveillance Centre, Dublin, Ireland

###### **Correspondence:** A. Vellinga

**Introduction:** Ireland participated in repeated point prevalence surveys (PPS) of healthcare-associated infections (HAI) and antimicrobial use (AMU) in long-term care facilities (LTCF) in 2012 and 2016, known as the HALT surveys.

**Objectives:** To explore risk factors of AMU and HAI in Irish LTCF.

**Methods:** Data for the HALT study are collected at institutional level, which provides aggregated prevalence and makes in-depth analysis difficult due to low numbers. To increase the power of the study, all LTCF participating in HALT 2016 were contacted to ask for additional data on all current residents; age, sex, urinary catheter use and presence of disorientation. The individual data was matched to the existing 2016 HALT database and multilevel analyses were performed.

**Results:** The prevalence of AMU and HAI in Irish LTCF in 2016 was 9.8% and 4.4% respectively

Prophylactic AMU (40%) was identified as a particular concern in the LTCF setting, more often recorded for females, residents living in LTCFs for more than one year, residents with an intellectual disability, and residents with a urinary catheter.

Additional data was obtained from 80 LTCF (out of 224) and 3,816 residents. Analyses showed that decreased AMU was associated with the number of health care assistants, presence of a coordinating physician in the LTFC, an antimicrobial stewardship committee, feedback on antimicrobial consumption, and medical care by residents’ personal GP. Less HAI was associated with feedback on surveillance of infection prevention & control practices in LTCF.

Almost half of the antimicrobials prescribed were second-line choices, with substantial inter-facility variation. In LTCF that provided education on antimicrobial prescribing, significantly less second-line antimicrobials were prescribed.

**Conclusion:** With limited additional data, important risk factors of AMU and HAI could be identified which help to target AMU stewardship and infection prevention and control programs in LTCF.

**Disclosure of Interest**: None declared

### P111 ANTIMICROBIAL PRESCRIBING PATTERN IN HEALTHCARE-ASSOCIATED INFECTIONS IN 26 BRAZILIAN HOSPITALS: 2017 AND 2018 POINT PREVALENCE SURVEYS

#### A. P. Matos Porto^1^, on behalf of Brazilian Global PPS group, I. Pauwels^2^, A. Versporten^3^, H. Goossens^3^, S. F. Costa^1^, on behalf of Brazilian Global PPS group: Thais Guimarães (Instituto Central-HC-FMUSP and Hospital do Servidor Publico Estadual de São Paulo, São Paulo, Brazil), Evelyne Girão (Hospital Universitário Walter Cantídio UFC, Hospital Regional Unimed Fortaleza and Hospital

##### ^1^Faculty of Medicine, University of São Paulo, São Paulo, Brazil; ^2^Faculty of Medicine and Health Science, University of Antwerp; ^3^Faculty of Medicine and Health Sciences, University of Antwerp, Antwerp, Belgium

###### **Correspondence:** I. Pauwels

**Introduction:** Healthcare-associated infections (HAI) are a common indication for the prescription of antimicrobials (ATM) and the inappropriate use of ATM is a key driver of ATM resistance.

**Objectives:** The aim of this study was to evaluate the ATM prescribing pattern in HAI in 26 Brazilian hospitals using the Global Point Prevalence Survey of Antimicrobial Consumption and Resistance (Global PPS) 2017 and 2018 data (www.global-pps.com).

**Methods:** 18 Brazilian hospitals conducted the PPS in 2017 and 17 in 2018 (9 hospitals participated in both years). The study included inpatients on antimicrobials on the day of the PPS. Data collection included details on the ATM prescriptions and a set of quality indicators. A web-based program was used for data-entry, validation and reporting. The Global PPS was developed by the University of Antwerp and b*ioMérieux* provided funding support.

**Results:** 1801 patients were evaluated in 2017 and 2433 in 2018, of which 941 (52.2%) and 1168 (48%) were on ATM. HAI was the indication for ATM in 328 (34.8%) and 377 (32.3%) patients in 2017 and 2018, respectively. Surgical site infection and device related infection together accounted for 50.6% of the prescriptions in 2017 and 44.6% in 2018. Pneumonia (2017-24.3%; 2018-25.1%) was by far the most common specific diagnosis in the surveys. Both in 2017 and 2018, the most frequent ATM prescribed were meropenem (2017-22.1%; 2018-21.9%), vancomycin (2017-16.6%; 2018-14.8%) and piperacillin-tazobactam (2017-11.2%; 2018-11.7%). About 95% of the drugs were given parenterally. The use of biomarkers to guide therapy was observed in 33.3% of the prescriptions in 2017 and 39.9% in 2018. Empiric use accounted for 65.7% and 69.7% of all ATM prescriptions for HAI in 2017 and 2018, respectively. Out of all the targeted therapies, most were aimed at multidrug-resistant (77.7 to 82.6%), mainly gram-negative bacteria.

**Conclusion:** HAI were the indication for ATM use in about one third of patients and pneumonia was the most common diagnosis. ATM were prescribed mainly empirically and there was a low use of biomarkers to guide ATM therapy. Three broad-spectrum ATM accounted for about half of the prescriptions for HAI showing that reinforcement of de-escalation strategy is needed in Brazilian hospitals.

**Disclosure of Interest**: None declared

### P112 THE BASIC PRINCIPLES OF ANTIMICROBIAL STEWARDSHIP IN DUTCH LONG-TERM CARE FACILITIES

#### A. Eikelenboom-Boskamp^1,2^, M. van Loosbroek^2^, E. Lutke-Schipholt^3^, M. Nelissen^4^, M. Verkaaik^2^, P. Geels^4^, S. Natsch^5,6^, A. Voss^1,7^

##### ^1^Department of Medical Microbiology and Infectious Diseases, Canisius Wilhelmina Ziekenhuis; ^2^ZZG Zorggroep; ^3^Canisius Wilhelmina Ziekenhuis, Nijmegen; ^4^Dutch Institute for Rational Use of Medicine, Utrecht; ^5^The Dutch Working Party on Antibiotic Policy, Leiden; ^6^Department of Pharmacy; ^7^Department of Medical Microbiology, Radboud University Medical Center, Nijmegen, Netherlands

###### **Correspondence:** A. Eikelenboom-Boskamp

**Introduction:** As residents in long-term care (LTC) settings increasingly suffer from multiresistant microorganisms, adequate use of antibiotics is of outmost importance.

**Objectives:** Adaptation and implementation of 'hospital' Antimicrobial Stewardship (AMS) into the LTC setting.

**Methods:** A project was launched within one of the providers of LTC in the South-east of the Netherlands to set up an AMS program. The antibiotic treatment protocol for urinary tract infection (UTI), lower respiratory tract infections and skin infections have been revised. Score-lists for data collection were created. New rules about applying urine dipstick test and culturing were implemented. The A-team monitored all antibiotic descriptions during a period of 5 months in 4 nursing homes. For nurses, e-learning was developed and tested. A focus group for patients/family members was organized to find out more about their needs.

**Results:** In total, 75 antibiotics prescriptions were reviewed of which 73.3% were assessed as correct. The e-learning needed minor adjustments after testing in 29 nurses. For the most important item UTI indicated by patients/family members, we contributed to an informative flyer developed by the association of elderly care physicians.

**Conclusion:** We recommend collecting periodic retrospective data for discussion in the A-team, prior to pharmacotherapeutic consultations to achieve a learning effect for future quality of the antibiotic prescriptions overall. In addition to the A-team activities, it's essential to involve nurses and patients/family members in the AMS program. As our project was limited to 4 locations, the generalizability and expected workload need further evaluation.

**Disclosure of Interest**: None declared

### P113 PROPHYLACTIC ANTIMICROBIAL USE IS COMMON IN FINNISH LONG-TERM CARE FACILITIES: RESULTS OF A POINT PREVALENCE SURVEY, 2017

#### S. Toura, D. Arifulla, E. Sarvikivi, J. Ollgren, O. Lyytikäinen

##### National Institute for Health and Welfare, Helsinki, Finland

###### **Correspondence:** S. Toura

**Introduction:** Antimicrobials are commonly used in long-term care facilities (LTCFs) and may contribute development of antimicrobial resistance. Particular concern is inappropriate use of prophylactic antimicrobials.

**Objectives:** We investigated current use of antimicrobials in LTCFs in Finland, with special emphasis in prophylaxis.

**Methods:** Antimicrobial use of LTCFs was investigated as a part of the third European point prevalence survey (HALT) coordinated by European Centre for Disease Prevention and Control. All residents present in LTCFs and receiving at least one systemic antimicrobial on the day of the survey were included. Local data collectors completed questionnaires including data on resident characteristics, antimicrobials and their indications. Information on antimicrobial policy was gathered using institutional questionnaire. To identify factors associated for prophylactic antimicrobial use, we conducted a multivariate beta-binomial regression model with logit link.

**Results:** In total, 175 LTCFs with 6762 eligible residents participated in the survey during September-November 2017. On the day of the survey, 462 residents received at least one antimicrobial agent (6.8%; range by LTCFs, 0–42%). Antimicrobials were more frequently prescribed for prophylaxis than for treatment (62% vs. 38%) and vast majority (88%) for prophylaxis of urinary tract infection (UTI). Methenamine (43%) was the most common UTI prophylaxis, followed by trimethoprim (38%) and nitrofurantoin (11%). There was no documented end date for 77% of prophylactic agents; 72% were prescribed in the current facility. Presence of written guideline for appropriate antimicrobial use (odds ratio (OR), 0.34; 95% confidence interval (CI), 0.16–0.73) and therapeutic guideline for UTIs (OR, 0.69; 95% CI, 0.47–0.99) were associated with less use of prophylactic antimicrobials.

**Conclusion:** Prophylactic antimicrobial use was common in Finnish LTCFs and most were prescribed for UTI prophylaxis. Increasing awareness and easy access to the national Current Care Guidelines for UTI could decrease inappropriate UTI prophylaxis and the use of methenamine.

**Disclosure of Interest**: None declared

### P114 PARTICIPATORY-ACTION RESEARCH FOR DESIGNING A GLOBAL ANTIBIOTIC STEWARDSHIP NETWORK IN THE PORTUGUESE-SPEAKING COUNTRIES' CONTEXT: A MIXED-METHOD STUDY IN CAPE VERDE

#### M. R. Maia, L. V. Lapão

##### Global Health and Tropical Medicine, NOVA IHMT, Lisboa, Portugal

###### **Correspondence:** L. V. Lapão

**Introduction:** The antibiotic stewardship services (ABS) contribute to healthcare-associated infections and antibiotic resistance (AR) global monitoring, providing clinical decision-support, through multidisciplinary team-work.

**Objectives:** Aiming at co-implement an ABS network within the Portuguese-speaking setting (CPLP), by enabling the evidence translation and second opinion between the countries, we surveyed Cape Verdean (CV) health professionals' (HP) perceptions about AR prevention and control, assessing ABS opportunities.

**Methods:** A Design Science Research Methodology under a Participatory Action Research approach establishes a contextualized ABS participatory process^1^. This mixed-method study addressed the first 2 stages, the problem and objectives. Quantitative study considered the HP’s answers (56 HP; 2 hospitals) to a questionnaire, about their perception on AR and ABS. Qualitative study set-up 10 open-ended structured interviews, clinical shift observation in 2 pilot-services (1 hospital), and meetings with key-elements for ABS, including leadership.

**Results:** Key-stakeholders for ABS multidisciplinary teamwork were identified. Preliminary results are:

1. HP reveal some knowledge but lack of awareness on AR and ABS (eg. 34% don’t recognize the AR local threat);

2. The absence of guidelines and lack of access to key-information affect prescribing confidence (eg. only 46% HP consider microbiology results in deciding antibiotherapy);

3. Priorities for a pilot service are the lack of qualified HP, the need to optimize material resources management and stock procurement, and the need for better access to patient's clinical and prescription information;

4. Digital resources and telemedicine system can be facilitators. Top-down communication and support are essential for the ABS process sustainability.

**Conclusion:** A Global (Portuguese-speaking) ABS network can be important in promoting prevention and effective control of AR, reducing differences between the CPLP countries. In CV, an educational program to support the co-design of ABS service and a decision-support information system are identified priorities. Digital health, like telemedicine, can be ABS facilitators.

**References**

1. Simões AS, Maia MR, Gregório J, Couto I, Asfeldt AM, Simonsen GS, et al. Journal of Hospital Infection 2018. doi:10.1016/j.jhin.2018.07.034.

**Disclosure of Interest**: M. Maia Grant/Research support from: FCT-POCH, L. Lapão Employee of: NOVA IHMT

### P115 ANTIMICROBIAL USE GUIDELINES IN LARGE HOSPITALS IN SWITZERLAND: AN ANALYSIS OF DIFFERENCES AMONG CENTERS

#### V. Naef^1^, G. Catho^2^, A. Ranzani^2^, B. Huttner^2^, S. Tschudin-Sutter^3^

##### ^1^University of Geneva; ^2^Hôpitaux Universitaires de Genève, Geneva; ^3^University Hospital Basel, Basel, Switzerland

###### **Correspondence:** V. Naef

**Introduction:** Clear and consistent guidelines for the improvement of the antibiotic use are a key element for antibiotic stewardship (ABS). International ABS guidance documents recommend that all hospitals should regularly update the treatment guidelines.

**Objectives:** In this study we aimed to review the recommendations for antibiotic use for 3 commons infectious pathologies in Swiss academic hospitals and to explore the differences among these guidelines.

**Methods:** We reviewed the treatment recommendations for community-acquired pneumonia requiring hospitalization (CAP), hospital-acquired pneumonia and uncomplicated urinary tract infections (UTI). We analyzed the guidelines from the 6 largest hospitals in Switzerland, which consider all the university hospitals and the largest non-university hospital. Further, we have take into account aspects such as molecules recommended, dose, administration mode and duration.

**Results:** The preliminary analysis of our data reveals significant differences among the guidelines. Although the antibiotic regimens are often similar, other parameters, such as the recommended dose, duration and the degree of detail of the recommendations (e.g. differentiating between different subsets of pneumonia or taking into account allergies) show considerable variability. Furthermore, the number of subcategories within an infectious syndrome varied widely. With regard to CAP, we have observed 13 different categories (ranging from 3 to 7) increasing the complexity of the comparison. With the exception of lower uncomplicated UTI, institutional guidelines are not always aligned with the corresponding Swiss and/or Europeans guidelines.

**Conclusion:** Our data show that there is a significant heterogeneity in the antibiotic treatment recommendations in large hospitals in Switzerland. This feature is not justified by differences in the epidemiology of infectious diseases. Efforts should be undertaken to harmonize the treatment recommendations in Swiss hospitals.

**Disclosure of Interest**: None declared

### P116 USE OF ANTIBIOTICS AMONG END-OF-LIFE HOSPITALIZED PATIENTS WITH ADVANCED CARE DIRECTIVES: INVESTIGATION OF FACTORS RELATED TO PHYSICIANS BEHAVIORAL INTENTIONS

#### R. Fedorowsky^1^, Y. Bachner^2^, A. Borer^2^, P. Ciobotaro^3^, T. Kushnir^2^

##### ^1^Infection Control Unit, Rabin Medical Center, Hasharon Hospital, Petach Tikva; ^2^Ben-Gurion University, Beer-Sheva; ^3^Infection Control Unit, Kaplan medical center, Rehovot, Israel

###### **Correspondence:** R. Fedorowsky

**Introduction:** over-use of antibiotics by physicians at the end of life (EOL) in patients with advance care directives (ADs) contributes to the evolution of antibiotic resistance bacteria (ARB) which increases mortality, and healthcare costs. The present study investigated factors related to physician use of antibiotics, based on the Theory of Planned Behavior, which predicts an individual's intention to engage in a behavior.

**Objectives:** 1. to investigate several factors (socio-demographic/organizational, motivational, infectious disease physicians (IDP) consultation, burnout) associated with physician behavioral intentions (PBI) to withhold antibiotics in EOL patients with ADs 2. to compare study variables across medical specialties and professional status.

**Methods:** a descriptive-correlational research in which 213 physicians were sampled in 27 wards, in acute and post acute-care hospitals.

**Results:** PBI to withhold antibiotics in EOL patient with and without ADs was rated "sometimes"/"rarely" respectively. Mean positive attitudes towards withholding antibiotics in EOL patients with ADs, awareness of ARB, system-related barriers (lack of guidelines) and mean pressure from families to prescribe antibiotic, were above the average. Physicians reported fewer IDP consultations in EOL patients than in other patients. The overall rate of burnout among all physicians was 44.5%.

Predictive/related factors for PBI were: managerial positions, seniority, board certification, type of hospitalization, system-related barrier, positive attitudes and consultations with IDP demanded by physicians, in EOL patient.

IDP reported the highest levels of: awareness of ARB, positive attitudes, PBI, barriers and burnout. Physicians in post acute-care reported the lowest levels of awareness of ARB, and PBI. Physicians in oncology reported the lowest positive attitudes levels. Attending compared to other physicians, reported the highest level of patient pressure, barriers and respectively the lowest positive attitudes and PBI.

**Conclusion:** IDPs are the potential change agents to improve antibiotics use in EOL patients with ADs. Intervention programs should be focused on oncology, post acute-care hospitals and attending physicians.

**Disclosure of Interest**: None declared

### P117 THEORETICAL APPROACHES IN THE DEVELOPMENT AND EVALUATION OF BEHAVIOUR CHANGE INTERVENTIONS THAT IMPROVE CLINICIANS’ ANTIMICROBIAL PRESCRIBING: A SYSTEMATIC REVIEW

#### H. Talkhan^1^, D. Stewart^1^, T. McIntosh^1^, M. Al Hail^2^, P. Abdulrouf^2^, H. Ziglam^2^, S. Cunningham^1^

##### ^1^School of Pharmacy and life sciences, Robert Gordon University, Aberdeen, United Kingdom; ^2^Hamad Medical Corporation, Doha, Qatar

###### **Correspondence:** P. Abdulrouf

**Introduction:** Antimicrobial resistance (AMR) and its threats have long been recognised. Many countries have developed antimicrobial stewardship programmes with strategies to optimise antimicrobial prescribing, minimise AMR and improve outcomes. There remains a need for behaviour change interventions at clinician level to promote appropriate prescribing practice.

**Objectives:** The aim of this review was to critically appraise, synthesise and present the available evidence for theoretical approaches in the development and evaluation of behaviour change interventions that improve clinicians' antimicrobial prescribing.

**Methods:** Eleven electronic databases and search engines were searched for peer-reviewed, English language studies investigating theoretically based behaviour change interventions that improve clinicians’ antimicrobial prescribing in any healthcare setting. The Theory Coding Scheme was utilised to evaluate the methods by which theories have been used. Clinical and methodological heterogeneity limited data synthesis.

**Results:** The searches resulted in 4227 potentially relevant papers after duplicates removal. Screening of titles/abstracts led to retrieval and dual assessment of 38 full-text papers. Of those, 12 studies met the inclusion criteria and were included in the systematic review. Most of studies included were from the UK (n = 8) and most were published in 2012 (n = 3); none was published before 2008. The majority of studies were carried out in primary care settings (n = 10) targeting upper respiratory tract infections (n = 9). Theoretical approaches used to inform the design and choice of intervention varied across included studies. The most common included: Theory of Planned Behaviour, Social Cognitive Theory and Operant Learning Theory.

**Conclusion:** This systematic review is the first to investigate theoretically based behaviour change interventions for antimicrobial prescribing. Only a small number of primary research studies involving theory in intervention development and evaluation were identified. There is a need for further research in this area.

**Disclosure of Interest**: None declared

## Poster session: Hand hygiene: compliance

### P118 SELF REPORTED COMPLIANCE TO THE 5 MOMENTS OF HAND HYGIENE REPORTS FROM THREE TERTIARY CARE HOSPITALS IN GERMANY

#### T. S. Kramer^1,2,3^, T. Holzmann^4^, A. Ambrosch^5^, W. Schneider^4^, H. Niesalla^6^, S. Diefenbacher^7^

##### ^1^Institute for hygiene and environmental medicine; ^2^National reference center for the surveillance of nosocomial infections; ^3^"Aktion Saubere Hände", Charité university medicine Berlin, Berlin; ^4^Institute for microbiology and hygiene, University hospital Regensburg; ^5^Instituts für Labormedizin, Mikrobiologie und Krankenhaushygiene, Krankenhaus Barmherzige Brüder, Regensburg; ^6^BODE SCIENCE CENTER, Bode Chemie GmbH, Hamburg; ^7^IDepartment of social psychology, University Ulm, Ulm, Germany

###### **Correspondence:** T. S. Kramer

**Introduction:** Hand hygiene is one of the most effective measures to prevent infections and transmission of pathogens in health care settings. However improvement of compliance can only be achieved through contious efforts. Feedback of results in compliance and consumption of alcoholic hand rub to healthcare workers can help to improve their compliance and increase awareness to hand hygiene.

**Objectives:** Objective of the study is to investigate self reported compliance to hand hygiene in comparison to directly observed compliance and annual hand rub consumption in health care workers.

**Methods:** A questionnaire was designed to ask for compliance to the 5 moment of hand hygiene during daily practice. Wards that finished direct observation of hand hygiene compliance within 8 month prior to questioning were included. Healthcare workers from three tertiary care medical centers were questioned.

**Results:** Data from 15 wards (n=4 ICUs ; n=1 IMC; n=1 4 regular wards) were included. Overall 186 HCW participated in the survey. Their self reported compliance was 78.2% (IQR:79.5-81.25). 2570 moments were directly observed with median compliance of 80% (IQR:67-84). Median alcoholic handrub consumption was 73.8ml/patient day (IQR: 41.3-127.55) in the year during direct observation. Nine wards self-reported their average compliance rate within a 5% margin of the directly observed compliance, while 5 wards underestimated their performance by more than 5%, and one ward overestimated their compliance by more than 5%. Overall a significant positive correlation between self-reported and observed compliance was found (*r* = .549, *p* = .034).

**Conclusion:** Self reported does correlate with directly observed compliance on a ward level, but might offer additional information on awareness of importance of hand hygiene.

**Disclosure of Interest**: T. Kramer: None declared, T. Holzmann: None declared, A. Ambrosch: None declared, W. Schneider: None declared, H. Niesalla Employee of: Bode Science Center, S. Diefenbacher Grant/Research support from: Bode Science Center

### P119 ADHERENCE TO HAND HYGIENE AND ITS ASSOCIATED FACTORS IN MATERNITY SETTINGS IN A TERTIARY REFERRAL HOSPITAL IN BLANTYRE, MALAWI: AN OBSERVATIONAL CROSS-SECTIONAL STUDY

#### C. Cohen^*^^1^, C. Dunlop^2^, A. Blennerhassett^2^, P. Bonongwe^3^, L. Gadama^3^, D. Lissauer^4^

##### ^1^University of Birmingham, Birmingham, United Kingdom; ^2^Institute of Metabolism and Systems Research (IMSR), University of Birmingham, Birmingham, United Kingdom; ^3^Malawi College of Medicine, Blantyre, Malawi; ^4^Institute of Metabolism and Systems Research (IMSR, University of Birmingham, Birmingham, United Kingdom

###### **Correspondence:** C. Cohen

**Introduction:** Hand hygiene (HH) in maternity settings in low income countries is often low, despite its importance in preventing peripartum infection and sepsis. Malawi has a high maternal mortality ratio, and to date no study has adequately ascertained the baseline HH adherence and its associated factors in maternity settings at a tertiary level hospital.

**Objectives:** To determine the baseline adherence to WHO standards of HH in maternity settings, assess ward infrastructure and assess the knowledge and behaviour of healthcare professionals.

**Methods:** Data was collected on labour, gynaecology, antenatal and postnatal wards for 4 weeks, and antenatal clinic for 2 weeks. Adherence was determined using a modified version of the WHO Observation Tool and adherence rates calculated. Ward Infrastructure was assessed using the modified WHO Ward Infrastructure Survey and compared graphically over time and by ward. Behaviour was assessed using the WHO Healthcare Worker Knowledge Questionnaire that was modified to include questions assessing knowledge and behaviour in terms of capability, opportunity and motivation in keeping with the COM-B model.

**Results:** HH Adherence to WHO guidelines in inpatient wards was 12.2%, and 57.1% in antenatal clinic. Adherence was lowest on labour ward (7.55%) and highest on antenatal (16.5%). Doctors had higher adherence than nurses (20% versus 13.5% p=0.160). Soap and water availability was higher in antenatal clinic (100% and 94.4% respectively) compared to inpatient wards (65.1% and 25.2% respectively). The majority of HH actions were hand rubbing (71.9%). Adherence did not correlate with soap and water availability by inpatient ward or over time. Healthcare worker knowledge was low, and doctors scored higher than nurses (61.4% versus 52.2% p=0.032). COM-B targets identified for behavioural change were psychological capability, physical opportunity and reflective motivation.

**Conclusion:** Adherence to WHO standards of HH in maternity settings is low. Ward infrastructure does not correlate with adherence. Knowledge is poor but is greater among doctors than nurses, who also had better adherence rates. Physical opportunity, reflective motivation and psychological capability should be addressed to change hand hygiene behaviour. Improving education could therefore improve adherence.

**Disclosure of Interest**: None declared

### P120 HAND HYGIENE COMPLIANCE IN THE INTENSIVE CARE UNIT: A SYSTEMATIC REVIEW

#### A. Vellinga, K. Lambe, S. Lydon, C. Madden, A. Hehir, M. Walsh, P. O'Connor

##### Irish Centre for Applied Patient Safety and Simulation, National University of Ireland, Galway, Galway, Ireland

###### **Correspondence:** A. Vellinga

**Introduction:** Hand hygiene (HH) is acknowledged as the most effective safeguard against HAI and is of particular concern in the Intensive Care Unit (ICU). The WHO five moments for hand hygiene has been widely adopted but HH compliance in the ICU is reported to be low.

**Objectives:** To derive HH compliance according to the WHO guidelines in the ICU and explore differences by region, ICU type, healthcare worker (HCW), method of observation, and each ‘moment’.

**Methods:** Electronic searches were conducted using Medline, CINAHL, PsycInfo, Embase, and Web of Science. Information was extracted and compliance recorded. Mean compliance, effect size and heterogeneity was calculated overall and for each category.

**Results:** More than 5,480 papers were screened of which 61 met the inclusion criteria for data extraction.

The median number of opportunities observed per study was 903 (mean = 3,026). Overall compliance ranged from 1.4% to 100% and the weighted mean compliance was 60% (n=184,597 opportunities). The effect size for all studies was 0.47 (CI=.04-.05), but heterogeneity was extremely high (I²=99.7).

HH compliance varied across geographic region from 65% in high-income countries to 9% in low-income countries. HH compliance also showed distinctive differences by ICU type; neonatal 67%, paediatric 41%, and adult 58%, and HCW; nursing staff 43%, physicians 33%, other staff 54%.

**Conclusion:** HH compliance in the ICU appears notably lower than international targets. The data collated offers a benchmark when evaluating or improving HH compliance in ICUs internationally.

**Disclosure of Interest**: None declared

### P121 HAND HYGIENE IN LONG-TERM CARE FACILITIES (LTCFS): EVERY MOMENT MATTERS!

#### A. Haenen^1^, M. Hulscher^1^, J. Liefers^1^, A. Voss^1^, A. Huis^1^, S. de Greeff^2^

##### ^1^IQ Healthcare, RADBOUDUMC, Nijmegen; ^2^Centre for Infectious Disaese research, RIVM, Bilthoven, Netherlands

###### **Correspondence:** A. Haenen

**Introduction:** The WHO ‘five moments for hand hygiene ‘are the key moments for hand hygiene and provide the fundament to any hand hygiene improvement strategy.

**Objectives:** We studied the baseline compliance and whether our tailored intervention is equally effective at all five moments.

**Methods:** Taking into account the barriers and facilitators we developed tailor-made interventions for 23 LTCF in the Netherlands. In a stepped wedge design we observed the five moments of hand hygiene, using the WHO “my five moments” methodology, before and after this tailored intervention.

**Results:** We observed 6365 opportunities for hand hygiene, of which moment 3 “after body fluid exposure risk” (n=2585) was the most common seen and moment 2 “before clean/aseptic procedures” (n=328) the least . The overall hand hygiene compliance increased with 17.5% from 18.2% before to 35.7% directly after the intervention. All five moments of hand hygiene improved following the intervention, however the compliance of moment 4 “after touching a patient” was the highest before the intervention (27.2%), highest after the intervention (54.5%) and increased the most (27.3%). The compliance “after body fluid exposure risk” was the lowest before (12.1%) and lowest after the intervention (25.2%) and also increased the least (13%).

**Conclusion:** Our preliminary results show that our tailored intervention appears to improve overall hand hygiene compliance, however, not at all moments a substantial increase was achieved. Hand hygiene “after body fluid exposure risk” is the most common opportunity observed, but compliance is the lowest as is compliance improvement after the intervention. Although the overall hand hygiene compliance increased after the intervention, the five moments of hand hygiene should be considered separately when looking at the effectiveness of the intervention.

**Disclosure of Interest**: None declared

### P122 ASSESSMENT OF HAND HYGIENE COMPLIANCE AMONG 4 WARDS AT THE UNIVERSITY OF SIERRA LEONE TEACHING HOSPITAL

#### R. E. Ngauja^1,1^, C. A. Conteh^1^, A. Maruta^2^

##### ^1^National Infection Prevention and Control Unit, Ministry of Health and sanitation; ^2^Health security and emergencies, World Health Organisation, Freetiown, Sierra Leone

###### **Correspondence:** R. E. Ngauja

**Introduction:** Although hand hygiene remains the most important strategy to prevent infections in the health care setting, compliance still remains unsatisfactory.. Many years after the multimodal strategy to improve hand hygiene compliance was introduced, hand hygiene compliance still remains unacceptably low across the globe. During the Ebola response period hand hygiene played an integral part of the response phase. Sierra Leone is among the countries that signed the pledge with WHO on Hand Hygiene Campaign to improve patient safety by reducing healthcare-associated infection. There is continuous need to scale up hand hygiene practices in health care facilities to reduce healthcare associated infections and combat AMR.

**Objectives:** The aim was to assess hand hygiene practices among Healhcare workers in four main wards of the hospital.

**Methods:** A situational analysis was conducted in 4 wards at the University of Sierra Leone Teaching Hospital Complex to assess compliance to hand hygiene. The observational form which is used to audit hand hygiene compliance was used in 4 wards at University of Sierra Leone Teaching Hospital, these are ward 1 (Paediatrics ward), Ward 4 (female Surgical), Ward 6 (male medical ward) and Accident and Emergency ward (Trauma). The WHO hand hygiene observational tool hand hygiene audit tool was used to collect data in the four wards; this tool involved the direct observation of hand hygiene practices among health care providers.

**Results:** Hand hygiene practices were below the normal standards in the 4 wards observed. The results were noted as Paediatric ward, 38.9%, Female Surgical ward 49.2%, Male Medical Ward 55% and Accident and Emergency 48.1%. The overall compliance for the four wards was 48%.

**Conclusion:** Healthcare workers still believe hand hygiene is not relevant to them and they always relate it as an Ebola reduction measure and had no interest on it since Ebola era ended. There is need to set accurate targets for hand hygiene compliance and as the local production of alcohol-based hand rub has started using WHO formulation in two major hospitals in the country. More support is needed from our implementing partners.

**Disclosure of Interest**: None declared

### P123 HAND HYGIENE IN BENIN SURGERY CARE UNITS: OPPORTUNITIES AND CHALLENGES

#### C. L. Yehouenou^1,2^, A. DOHOU^1,3^, A. D. FIOGBE^3,4^, C. DEGBE^5^, A. SIMON^6,7^, O. DALLEUR^8^

##### ^1^clinical Pharmacy Research Group (CLIP), Louvain Drug research Institute (LDRI), Brussels, Belgium; ^2^Faculté des Sciences de la Santé (FSS), Université d'Abomey calavi (UAC); ^3^Université d'abomey Calavi, faculté des sciences de la santé, Cotonou, Benin; ^4^Clinical Pharmacy Research Group (CLIP), Louvain Drug Research Institute, Brussels, Belgium; ^5^Clinique d'hygiène hospitalière (CNHU), Institut regional de santé publique , cotonou, Benin; ^6^Institut de Recherche Expérimentale et Clinique (IREC), Université Catholique de Louvain UClouvain; ^7^Microbiologie, Clinique Universitaire de saint Luc, Brussels; ^8^Louvain drug research institute, CLIP/ Uclouvain, Bruxelles, Belgium

###### **Correspondence:** C. L. Yehouenou

**Introduction:** Hand hygiene (HH) has been described as the cornerstone and starting point in all infection control programs.

**Objectives:** This study aimed to evaluate HH compliance among Healthcare Workers (HCWs) in Benin surgery care units.

**Methods:** We conducted an observational study for two months through six public hospitals in obstetrics and visceral surgery. The World Health Organization HH observation tool was used. HH compliance was calculated by dividing the number of hand hygiene action by the total number of opportunities.

**Results:** We observed 432 HCWs: 55,6% of nurses, 25,7% of auxiliaries, 13,4% of physicians and 5,3% of others. Among the 1315 observed opportunities for HH, average compliance was 33,3%. The compliance rates differed (p<0,001) between obstetrics (49,4%) and abdominal surgery (24,3%).The assumption that HCWs prefer to protect themselves to a greater extent than patients was noted: HH compliance was better after body fluid exposure (54,5%) and after patient contact (37,5%) than for the other opportunities.(p<0,001)**.**

Among 438 hand hygiene actions, only 122 (27,9%) were attributed to the hand rubbing while 316 (72,1%) were to hand washing. Hand washing remains the most preferred HH action for all of WHO five moments. When HH was applied, technique and duration were often inappropriate. Median duration of HH was respectively 10 ± 7 seconds for hand washing and 9 ± 6 seconds for the hand rubbing. Hand hygiene duration was appropriate in< 6% for ABHR and 0,5% for hand washing. Fortunately, most HCWs respected the prerequisites of HH (91% had natural short and without polished fingernails). We observed lack of infrastructures required for HH (i.e. sink, clean water, provision of alcohol solution) in all of hospitals.

**Conclusion:** HCWs missed two opportunities out of three to apply HH and didn’t apply the good techniques. HH practices education should be a priority to improve patient safety in Benin.

**Disclosure of Interest**: None declared

### P124 HAND HYGIENE PROGRAM IN A TERTIARY CARE TEACHING HOSPITAL IN WEST BANK:2017-2019

#### S. Belkebir, A. Kanaan, R. Jeetawi

##### Preventive medicine, An Najah National University Hospital, 00000, Palestinian, State of

###### **Correspondence:** A. Kanaan

**Introduction:** For the implementation and monitoring of Hand Hygiene practice among healthcare workers, the Infection control program at An-Najah National University Hospital, a tertiary care referral teaching hospital in the North of Palestine, uses Direct Observation as recommended by the World Health Organization.

**Objectives:** This descriptive study aims at reporting the prevalence of HH across the institution from January 2017 to April 2019

**Methods:** To monitor improvements, WHO toolkit for direct observation of HH compliance was used by IPCP team and anonymously by trained observers. Compliance was assessed and reported on monthly basis. PDSA, root cause analysis and groups discussions were used as improvement tools. Training was reinforced by the use of ultraviolet light and fluorescent alcohol hand rub. Yearly, staff was engaged in HH day activities. Leadership support was constant by securing annual budget for the HH program and the enforcement of HH policy.

**Results:** The overall compliance rates during the period of study were: 44%(R:31-57%) in 2017, 53%(R:30-72%) in 2018 and 58%(R:55-60%) from January to April 2019.During 2018,an increase in 23% in HH compliance was observed among Nursing staff and 14% among doctors. The most frequent missed opportunity was the compliance with Moment 1“before patient” by 59%, which was mainly due to a misuse of gloves. Qualitative analysis of the results reflected barriers to an optimal hand hygiene rate (set at 85%) such as: high turnover among nursing and residents, shortage of alcohol sanitizer in some months, reported skin irritation problems related to alcohol gel and poor engagement to HH.

**Conclusion:** A steady increase in HH compliance was observed among the institution from 2017 to 2019.Efforts in improving the commitment of the staff to HH is needed as well administrative support and training (especially in the good use of gloves).

**Disclosure of Interest**: None declared

### P125 CAUSES OF NON-COMPLIANCE OF HAND HYGIENE USING A COVERT OBSERVATION METHODOLOGY

#### A. El-Saed^1^, M. Alshamrani^1^, S. Noushad^1^, H. Balkhy^2^

##### ^1^Infection control, King Abulaziz Medical City, Riyadh, Saudi Arabia; ^2^Antimicrobial Resistance, WHO, Geneva, Switzerland

###### **Correspondence:** A. El-Saed

**Introduction:** Covert hand hygiene (HH) observation was suggested as a tool to overcome the Hawthorne bias, which tends to overestimate the routinely observed compliance of HH.

**Objectives:** As HH observation done secretly at our institution showed a low HH compliance, we thought to examine the professional and healthcare causes of non-compliance of HH.

**Methods:** A cross-sectional study design was conducted among healthcare workers (HCWs) at King Abdulaziz Medical City, Riyadh, Saudi Arabia between October 2012 and July 2013. HH observation was done secretly by temporarily-hired professionally-trained observers. Non-compliance was defined as failure to perform hand rubbing (with alcohol-based formulation) or hand washing (with soap and water) during one of the WHO five-moment HH opportunities. HH observation was followed by a questionnaire about the possible causes of non-compliance of HH filled by the HCW and assessment of HH infrastructure at the service location reviewed by the observer.

**Results:** A total 502 HCWs with a total of 5680 opportunities were observed in 28 service locations. The overall non-compliance was 54.1%. The factors that were significantly associated with non-compliance included lack of HH education/training in last 2 years, long working hours, lack of job recognition, lack of HH auditing, lack of alcohol hand rub pocket bottle, lack of conveniently placed water sinks and alcohol hand rub wall dispensers, non-frequent replacement of empty wall dispensers, and lack of nearby promoting posters. Incorrect HH technique was significantly associated with professional category, attending HH education/training, presence of HH auditing at the service location, and the place of alcohol hand rub wall dispenser.

**Conclusion:** The current findings list a number of professional and healthcare factors associated with non-compliance of HH. The covert observation of HH done before the questionnaire is likely to avoid bias in estimating non-compliance and its causes. The findings may potentially help decision maker to identify specific work-environment factors that need to be modified to improve the compliance of HH. Further research may be required to assess the impact of these suggested changes on the compliance of HH.

**Disclosure of Interest**: None declared

### P126 NEW CONCEPT OF HAND HYGIENE SURVEILLANCE IN NURSING HOMES - CANTON DE VAUD

#### M. Attinger Querzoli, C. Petignat, I. N. Tessemo

##### Direction générale de la santé, unité HPCi, Lausanne, Switzerland

###### **Correspondence:** M. Attinger Querzoli

**Introduction:** Health care-associated infections constitute a concern for the safety of patients and are considered by the experts a major issue in public health.Hand hygiene (HH) is the most important measure to avoid the transmission of harmful germs and to prevent cross transmission of such germs. The promotion of HH, according to the five WHO indications is integrated into the cantonal strategy of infection control in nursing homes introduced by Cantonal Unit for Prevention and Infection Control (unité HPCi). Since 2004, in nursing homes, the promotion has been applied through the yearly surveillance of hand rub solution consumption and in 2010 began a surveillance of HH compliance via audits. While audits reveals a 80% HH compliance, the consumption of hand rub solution is only 2.5 disinfections/day/resident (target = 5). There was no correlation between HH observations and the volume of hand rub used, when comparing the two surveillances.

**Objectives:** The unité HPCi developed a new tool for nursing homes, to evaluate the consumption of hand rub solution, taking into account, nursing homes missions and the number of delivered cares per day (degree of dependency of residents).

**Methods:** Elaboration of the form survey for data collection; tutoring; calculation of the average number of opportunities of HH for the nursing home; determination of the expected averages of consumption of hand rub solution; quarterly calculation of the consumption of hand rub solution before collecting the number of delivered cares per day (base line); determination of the minimum personalized target of consumption of hand rub solution, which the nursing home has to reach ; new quarterly calculation of the consumption of hand rub solution.

**Results:** In 2018, a test phase allowed the adjustment of the tool, in collaboration with healthcare workers involved in this new concept.

**Conclusion:** This new concept aims to promote HH by means of a personalized indirect tool. The method can help healthcare workers to adhere to correct HH procedures and favors a better adoption of HH surveillance. It allows the determination of personalized objectives of progress regarding HH compliance. This tool has been implemented in 2019 in all nursing homes of canton de Vaud.

**Disclosure of Interest**: None declared

### P127 COMPLIANCE WITH HAND HYGIENE (HH) PRACTICES UNDER STANDARD (SP) VS. CONTACT PRECAUTIONS (CP) IN A TERTIARY CARE HOSPITAL

#### C. Fankhauser, F. Timurkaynak, D. Pires, E. Tartari, A. Peters, C. Guitart, J. Sztajzel, A. Iten, D. Pittet

##### Infection Control Program, HUG, Geneva, Switzerland

###### **Correspondence:** C. Fankhauser

**Introduction:** The impact of glove use on HH practices is not well known. Some reports describe low adherence with the use of gloves, gowns, and HH while CP are applied, others show noncompliance with HH when donning or removing gloves, or absence of glove change in between HH indications. Understanding noncompliance can improve HH and the judicious use of gloves.

**Objectives:** To compare HH compliance when caring for a patient under CP vs. SP. To assess the impact of glove use on HH compliance.

**Methods:** Trained, validated observers performed HH monitoring using the WHO 5 moments for HH. HH observations were categorized for three conditions: **Overall** (total HH observations); **CP**: when CP was indicated; **SP:** (Overall - CP). Results were compared across 4 healthcare (HC) sectors, periodic HH observation sessions conducted from 2014 to 2017 hospital-wide at HUG (**A)**, Geriatric **(B**), and Orthopedic (**C**) wards, and from 01/06/2017 to 30/03/2018 among volunteer HC workers in Geriatric wards (**D).**

**Results:** Proportions of HH opportunities observed when CP was indicated were: **A)** 4.57% (1075/23541), **B)** 9.09% (257/2827), **C)** 2.11% (47/2232) and **D)** 4.12% (165/4006).

**Conclusion:** HH compliance was significantly lower for opportunities identified as CP. Both SP and CP showed lower HH compliance while using gloves**.** The impact is higher under CP.

**Disclosure of Interest**: None declared

**Table 1 (abstract P109).** See text for description

Variables	Total n=316	Department with antimicrobial stewardship n=89	Department without antimicrobial stewardship n=227	p
Age (in years)	Mean - 62,78 SD - 18,07	Mean - 62,24 SD - 16,30	Mean - 62,99 SD - 18,75	0,725
Male Gender (%)	56,3	59,6	55,1	0,529
Hospitalization days	Median - 7 IQR - 13	Median - 8 IQR - 21,5	Median - 7 IQR - 12	0,049
Antimicrobial prescription (%)	37,7	41,6	36,1	0,370

### P128 IMPROVING COMPLIANCE WITH HAND ANTISEPSIS WITHOUT DECREASED EFFICACY BY SHORTENING THE RUB-IN TIME OF ALCOHOL-BASED HANDRUBS TO 15 SECONDS

#### J. Harnoss^1^, S. Dancer^2,3^, C. Kaden^4^, R. Baguhl^5^, T. Kohlmann^6^, R. Papke^5^, M. Zygmunt^7^, O. Assadian^8^, M. Suchomel^9^, D. Pittet^10^, A. Kramer^5^

##### ^1^University of Heidelberg, Heidelberg, Germany; ^2^Hairmyres Hospital, Hairmyres; ^3^Edinburgh Napier University, Edinburgh, United Kingdom; ^4^Greifswald; ^5^University Medicine Greifswald, Greifswald, Germany; ^6^Institute for Community Medicine ; ^7^Gynaecology and Obstetrics, University Medicine Greifswald, Greifswald, Germany; ^8^Medical University of Vienna & Hospital Landesklinikum Neunkirchen; ^9^Medical University of Vienna, Vienna, Austria, ^10^University of Geneva, Geneva, Switzerland

###### **Correspondence:** A. Kramer

**Introduction:** A previous study among neonatal intensive care unit (NICU) nurses showed that the antibacterial effectiveness of alcohol-based handrubs (ABHR) can be achieved in 15s instead of 30s with a significant increase in the frequency of hand antisepsis.

**Objectives:** To examine 15s vs 30s antisepsis performance by measuring microbial load on fingertips and compliance among nurses in a low-risk gynaecological ward.

**Methods:** An independent trained observer monitored the frequency and compliance with hand antisepsis during shifts in a crossover design. Fingertips including thumbs were rinsed in soy broth before hand rubbing at the beginning of a shift and then hourly to determine the bacterial load. Performance activity was assigned to the contamination class of the Fulkerson scale. Immediately before the lunch break, volunteers cleaned their hands for a randomly determined exposure time of 15 or 30 seconds.

**Results:** For participants rubbing for 15 seconds, both the frequency of hand antisepsis and compliance were significantly higher (p

**Conclusion:** The observed improvement in compliance resulting from shortening the ABHRs application time confirms that time acts as a psychological barrier for optimal compliance with hand antisepsis. Therefore, shortening the application time to 15 s should be considered within the critical components of a successful multimodal intervention strategy to improve compliance with hand hygiene in clinical practice.

**Disclosure of Interest**: None declared

## Poster session: Hand hygiene: Monitoring at large level

### P129 BACK TO BASICS: USING NEW GLOBAL DATA ON HEALTH CARE WASTE MANAGEMENT AND HAND HYGIENE FACILITIES TO TARGET INVESTMENTS, PREVENT INFECTIONS AND SAVE LIVES

#### M. Montgomery^1^, R. Johnston^1,2^, J. Storr^2^, C. Kilpatrick^1^

##### ^1^Water, Sanitation, Hygiene and Health Unit; ^2^WHO, Geneva, Switzerland

###### **Correspondence:** J. Storr

**Introduction:** In April 2019, the WHO/UNICEF Joint Monitoring Programme for Water Supply, Sanitation and Hygiene published harmonized baseline estimates for water, sanitation, hand hygiene, health care waste management, and environmental cleaning (WASH) services in health care facilities. The new JMP global database on WASH in health care facilities includes national data from 125 countries drawing upon assessments of over 560,000 health care facilities.

**Objectives:** To describe the current global status on WASH in healthcare facilities focusing on waste management and hand hygiene

**Methods:** A compilation and analysis of existing monitoring data that countries have already collected was undertaken. Data were extracted from 260 nationally-representative facility assessments and mapped to a standardized set of global indicators.

**Results:** Globally, 42% of health care facilities have neither alcohol-based handrub nor soap and water at points of care. Availability varies widely with at least one country in every region having less than 50% coverage. In some middle- and upper-income countries, where arguably there should be sufficient resources to ensure that universal coverage, less than half of health care facilities had hand hygiene facilities. Globally 40% of health care facilities lack systems to segregate waste. Waste segregation in least developed countries ranges from above 90% to less than 5%. Practices are generally better in hospitals compared to smaller health facilities.

**Conclusion:** These estimates comprise the most comprehensive assessment to date, drawing upon nationally representative surveys covering over half a million health care facilities, and confirm an unacceptable WASH situation. Addressing these major shortfalls can be done with relatively few resources and would result in large cost savings through averting infections and improving the quality of care. WHO and UNICEF are leading global efforts to ensure that all countries and health care facilities look to strengthen collaboration between WASH and IPC, adopting innovative and sustainable approaches to achieve improvement.

**References**

WHO/UNICEF. WASH in health care facilities. Global baseline report 2019. https://www.who.int/water_sanitation_health/publications/wash-in-health-care-facilities-global-report/en/

**Disclosure of Interest**: None declared

### P130 HAND HYGIENE IN HOSPITALS – THE EVOLUTION OF A GLOBAL CAMPAIGN

#### C. Kilpatrick^1^, B. Allegranzi^1^, D. Piitet^2^

##### ^1^World Health Organization; ^2^WHO collaborating centre for patient safety and infection control, University of Geneva Hospitals and Faculty of Medicine, Geneva, Switzerland

###### **Correspondence:** C. Kilpatrick

**Introduction:** In the last 20 years, a paradigm shift has occurred in hand hygiene. In order to put this evolution into context, it is important to understand how a World Health Organization (WHO) global annual hand hygiene campaign has played its role and reached healthcare settings in all corners of the globe. WHO launched the *SAVE LIVES: Clean Your Hands* global campaign on 5 May in 2009, marking the first international day of hand hygiene in healthcare.

**Objectives:** To examine how a ubiquitous but local set of ideas led to a global phenomenon in part supported by a global campaign, and to outline how committed organizations are championing hand hygiene improvement stimulated by the WHO campaign.

**Methods:** A desk exercise was undertaken to review the WHO web pages in order to summarise the themes and calls to action presented for each year of the global campaign, mapping their relevance to other global health agendas. A preliminary review of 10 years of campaign registrations using data from the WHO web ‘sign up’ page was also conducted, alongside an analysis of supporting organizations over time.

**Results:** One of the key findings from the campaign themes highlighted an evolution of focus, from pure hand hygiene to wider global health issues. These health issues were considered in some way to be interconnected with the need for hand hygiene improvement and prevention of avoidable harm. Healthcare facility campaign registration numbers have continued to increase year on year, with the current status being 22 144 health-care facilities in 182 countries. A range of national and global organizations have continually promoted the campaign.

**Conclusion:** While a clear target and denominator for demonstrating global campaign success should be employed, these preliminary results outline a useful starting point in understanding a global campaign's reach. Research has begun to be published showing the upward trend in hand hygiene improvement linked to promotional campaigns. The continued annual commitment of organizations, in part, demonstrates sustainability of campaign messaging framing. However, more analysis of the impact of the global campaign is needed to understand the curve of commitments, which would be of interest to any other global health care campaign.

**Disclosure of Interest**: None declared

### P131 CURRENT STATUS OF HAND HYGIENE MONITORING AND PERFORMANCE FEEDBACK ACROSS LOW-, MIDDLE- AND HIGH-INCOME COUNTRIES

#### E. Tartari^1^, M. Borg^2^, E. Castro-Sánchez^3^, W. Zingg^1^, B. Allegranzi^4^, D. Pittet^1^

##### ^1^University Hospital of Geneva, Geneva, Switzerland; ^2^Mater Dei Hospital , Msida, Malta; ^3^Imperial College, London, United Kingdom; ^4^WHO, Geneva, Switzerland

###### **Correspondence:** E. Tartari

**Introduction:** Hand hygiene (HH) monitoring with performance feedback is considered a key quality indicator; however, differing practices exist in healthcare facilities worldwide.

**Objectives:** To obtain a snapshot of HH monitoring and performance feedback practices internationally.

**Methods:** We developed a survey based on the WHO Hand Hygiene Self-Assessment Framework (HHSAF) and invited a convenience sample of infection preventionists (ICPs) from more than 100 countries attending the International Conference on Prevention and Infection Control (ICPIC) in June 2017 to complete it. The survey included questions on the following HHSAF subcategories: 1) availability of HH products 2) direct monitoring of HH compliance; 3) monitoring consumption of alcohol-based handrub (ABHR); 4) feedback to key stakeholders.

**Results:** 198 ICPs from 71 countries across all income levels completed the questionnaire (22 low-income countries [LIC], 71 middle-income countries [MICs] and 105 high-income countries [HIC). All four HHSAF subcategories were reported to be in place in 0/22 hospitals in LIC, 31/71 in MICsand 38/105 in HIC. Overall 55% reported a monitoring system to ensure that HH products were available at point of care (60% HIC; 35% MIC; 5% LIC). 50% of respondents monitored consumption of ABHR as a proxy measure of HH compliance (69% HIC; 30% MIC; 1% LIC). 47% of hospitals performed direct monitoring of HH compliance in all wards (71/105 HIC; 22/71 MIC; 0/22 LIC) with another 27% doing so only for specific wards (21/105 HIC; 23/71 MIC; 9/22 LIC). Of these, 46% used a combination of ICPs and ward staff to perform HH compliance audits, 40% used only ICPs, 10% relied only on ward staff while 4% used a system of ‘shoppers’/video cameras. HH monitoring was primarily performed overtly after informing ward managers (45%). Covert audits with no notification to ward leadership were done by 16% and after informing observed staff in 14%. HH audit results were fed back to key stakeholders in 67% of institutions.

**Conclusion:** Significant challenges remain in implementing the HHSAF recommendations, even in HIC. However the problem is greatest in LMIC where increased infrastructures, resourcing and stability to implement robust IPC programmes are urgently needed.

**Disclosure of Interest**: None declared

### P132 HAND HYGIENE ENABLERS AND PRACTICES AMONG HEALTH CARE WORKERS IN UGANDA; FINDINGS FROM A NATIONAL INFECTION PREVENTION AND CONTROL (IPC) SURVEY

#### W. Omuut^1^, R. Walwema^1^, M. Lamorde^1^, M. Kesande^1^

##### ^1^Global Health Security Program, Infectious Diseases Institute, Makerere University, Kampala, Uganda

###### **Correspondence:** W. Omuut

**Introduction:** The World Health Organizations’(WHO) annual global campaign to promote hand hygiene (HH) aims to renew global commitment to a low-cost lifesaving infection prevention control (IPC) standard. Ahead of the 2018 national HH celebrations in Uganda, a survey was conducted to establish the status of HH practices in Ugandan health care facilities (HCFs).

**Objectives:** To: Determine HH capacity in Ugandan HCFs. Establish HH compliance of health workers.

**Methods:** A cross sectional survey was conducted in April 2018 at 14 regional referral hospitals (RRHs). 14 IPC leaders were interviewed to assess HH capacity and 412 health care workers observed for compliance to WHO 5 moments of HH. WHO standard tools: HH self-assessment frame work 2010 and HH observation tool 2009, were adopted and assessors trained before data collection. HH capacity was assessed using five components of the WHO multi-modal HH improvement strategy: system change, education and training, evaluation and feedback, reminders at the work place and institutional safety climate. Data were analysed using excel and epi info and summarised using scores, colour schemes and percentages.

**Results:** Most RRHs attained the basic level (126 - 250 out of 500) of hand hygiene capacity. Lowest performance was seen in the ‘evaluation and feedback’ component (24.3 out of 100). The best performing facility (Mubende RRH) had an intermediate level (330 out of 500), attributed to committed leadership, a vibrant IPC committee and innovations like local production of alcohol-based hand sanitizer.

Overall HH compliance using WHO 5 moments was (mean, range) 21% (7% - 54%). Highest compliance was seen for the measures ‘after patient contact’ (36%) and ‘after body fluid exposure risk’ (33%). Compliance to ‘before touching a patient’ and ‘before clean/aseptic procedure’ were lowest (16%). Reasons for inadequate practice were; inadequate supplies, lack of awareness of standard policies of hand hygiene, lack of locally adapted monitoring and evaluation frameworks and poor health worker attitudes.

**Conclusion:** Inadequate HH practice of health workers in Uganda is attributed to gaps in supplies, inadequate knowledge and poor attitudes. Scale up innovations such as local manufacture of alcohol based hand sanitizer and increased leadership commitment should be harnessed to improve practice.

**Disclosure of Interest**: None declared

### P133 SELF-REPORTED HANDWASHING COMPLIANCE IN THE GENERAL POPULATION IN GERMANY: AN IN-DEPTH ANALYSIS OF THE FIRST BZGA-SURVEY ON HYGIENE AND INFECTION CONTROL

#### T. von Lengerke, A. A. Mardiko

##### Department of Medical Psychology, Hannover Medical School, Centre of Public Health and Healthcare, Department of Medical Psychology, Hannover Medical School, Centre of Public Health and Healthcare, Hannover, Germany

###### **Correspondence:** T. von Lengerke

**Introduction:** Promoting handwashing in the general population is a key public health area not only in pandemics but also perennial infection prevention.[1] Thus, it is crucial to know how compliant the general population is, and perceives themselves to be. The German Federal Centre for Health Education (BZgA) has conducted thre relevant representative surveys.[2] Regarding single parameters of handwashing, self-reported compliance rates of up to 96% (indication "after using the toilet") were found.[2] However, comprehensive compliance (in terms of correct indications, duration ≥20 sec., AND technique using soap, washing between fingers, and drying off) has not been reported.

**Objectives:** To provide comprehensive handwashing compliance rate estimates for German adults, and report associations with recalling handwashing instruction signs in public restrooms.

**Methods:** In 2012, N=4483 residents living in a household in Germany aged 16-85 years were surveyed by computer-aided telephone interviews (response: 49.7%).[3] Handwashing \parameters were coded as (non-)compliant based on BZgA-recommendations. Respondents indicated whether they had ever seen instruction signs in public restrooms. Statistics were calculated and regression analyses conducted via IBM® SPSS® v24.

**Results:** The rate of compliance defined as a pattern of self-reported duration of handwashing of ≥20 sec., correct technique, and washing hands in more than 6 of 9 indications, was 8% (men: 6%, women: 9%), while 31% were totally non-compliant (29%/33%). Compared to non-compliance, the odds of total compliance were more than twice as high among respondents recalling handwashing instruction signs in public restrooms (men: OR=2.15) and women (OR=2.08; p<.0001), however with rates still being low (e.g. 10% in women).

**Conclusion:** In 2012, self-reported handwashing compliance in the general population in Germany was low when defined by a pattern of compliant duration, technique, and situational indication. Intensified promotion is obviously needed, and may use instruction signs (e.g. in public restrooms) as a starting point.

**References**

1. Meilicke et al. Hygiene perception changes during the influenza A H1N1 pandemic in Germany. BMC Public Health 2013; 13: 959

2. BZgA. [Infection control via hygiene]. Cologne: BZgA; 2018

3. BZgA. [Infection control 2012. doi:10.4232/1.5175] Mannheim: GESIS Data Archive; 2015

**Disclosure of Interest**: None declared

### P134 HYPOTHESIS - APPLYING NEW METHODS OF MODERN COMMUNITY-BASED HEALTH EDUCATION IS THE WAY TO SUCCEED IN A HAND HYGIENE EDUCATIONAL PROGRAM IN THE HOSPITAL ENVIRONMENT

#### M. Dadgarmoghaddam^1^, A. Alipour Tabrizi^2^, N. Lotfinejad^3^

##### ^1^Assistant professor of community medicine, Community medicine department, Research deputy of school of medicine, Mashhad University of Medical Sciences, Mashhad; ^2^Iranian legal medicine research center, Legal medicine organization, Tehran; ^3^Department of research, Mashhad University of Medical Sciences, Mashhad, Iran, Islamic Republic Of

###### **Correspondence:** M. Dadgarmoghaddam

**Introduction:** Despite various efforts in the field of infection prevention and control regarding hand hygiene (HH) at hospitals, the level of HH compliance among healthcare workers (HCWs) is still not acceptable. In one study conducted in one of the Mashhad's hospitals in Iran, even warning signs of hand-borne infection in the elevators were more noticeable to the audience and statistics revealed that this was more effective than pamphlets.

**Objectives:** We formulated a hypothesis about the best approach to succeed in HH educational program in hospital environment.

**Methods:** A qualitative study identified the effects of HH education in the population.

**Results:** Considering the history of HH, infectious diseases and Ignaz Philipp Semmelweis’s practices, HH has been a challenging human behavior although it is very simple. Reasons for failing in health-related education, such as HH in hospitals, are categorized as: culture-related issues, inappropriate information system to achieve educational goals, non-realistic behavioral changes, inappropriate target population and inadequacy of teaching methodology. Educators of the health system believe that health-related education is only achievable by holding classes and providing brochures and guides. Perhaps it should be noted that the traditional teaching methods need to be transformed and they should be replaced by novel strategies such as using technological advances including emojis, cartoons, and games.

Social behaviors hinge on a larger social system, and sustainable changes in health-related behaviors such as HH require altering behavior starting from lower age groups and supportive changes across a community as a system by using modern learning methods.

**Conclusion:** Based on our findings, we recommend this hypothesis to be be tested in a community trial and results observed in a prospectively followed cohort subject to a community intervention.

**Disclosure of Interest**: None declared

### P135 NATIONAL GROUP FOR HAND HYGIENE - PRACTICE AND EXPERIENCES

#### L. Hobzova^1^, D. Vaculikova^2^

##### ^1^Hygiene, epidemiology and infection control department , University Hospital Hradec Králové, Hradec Králové; ^2^Hygiene, epidemiology and infection control department , Masaryk City Hospital , ÚSTÍ NAD LABEM, Czech Republic

###### **Correspondence:** L. Hobzova

**Abstract video clip:** The National workong group for hand hygiene is a self-governing and voluntary association bringing together people working or related to work in the field of public health with a focus on promoting hand hygiene or training health and non-health workers in this area to pursue and fulfill common interests and goals, namely:· Support and maintain the development of the highest possible standards of hand hygiene· setting up the system of education of employees of individual professions in the area of health care in the hands· supporting scientific research activities and putting new knowledge into practice

· by submitting proposals to the authorities of state authority and other organizations and institutions.

Group was based in 2018, the goal is to present practice, experiences, achievements, options.

In 2018, as part of the World Day of Hand Hygiene, we focused on septic conditions, where hand hygiene is a key prevention factor in order to alert the professional and lay public to the importance of hand hygiene and to influence not only these serious conditions through good hand hygiene practice. We held a press conference on this subject.

**Disclosure of Interest**: None declared

### P136 TRAIN-THE-TRAINERS PROGRAM AS A TOOL TO IMPROVE HAND HYGIENE PROMOTION CAPACITY IN PUBLIC MEXICAN HOSPITALS

#### H. Marquez Villarreal^1^, M. Hernández De Mezerville^2^, O. Gálvez De la Cruz^1^, D. Pittet^3^

##### ^1^Universidad de Guadalajara, Guadalajara, Jalisco, Mexico; ^2^Hospital Nacional de Niños de Costa Rica Dr. Carlos Sáenz Herrera, San José, Costa Rica; ^3^The University of Geneva Hospitals, Geneve, Switzerland

###### **Correspondence:** H. Marquez Villarreal

**Introduction:** Hand hygiene (HH) is fundamental to prevent healthcare-associated infections (HAI), is promoted by WHO through the Multimodal HH Improvement Strategy (MHHIS) implementation. A useful tool to identify the promote hospitals capacity to HH is the HH Self-Assessment Framework (HHSAF), that serves as a diagnostic to identify key issues for improvement and to facilitate action plans formulation. A very useful tool to standardize training and intensify HH actions and interventions is the Train-The-Trainers (TTT) program, which contains promotional, conceptual and practical elements of HH implementation

**Objectives:** To determine the level of HH promotion capacity of Mexican public hospitals through the HHSAF application and to monitor the TTT program impact

**Methods:** It´s a prospective multicenter study using a mixed, quantitative and qualitative approach in 2017 and 2018. With a convenience sample of 23 public hospitals. TTT program was replicated in Nov/17 and May/18 to IPC teams members of the hospitals participating. Data were obtained through the HHSAF, that addresses 5 MHHIS elements, each with a maximum value of 100 points. The statistical analysis was descriptive and inferential, included measures of central tendency, frequencies and dispersion and the application of the Shapiro-Wilk and Student's T tests

**Results:** According to HHSAF, the institutions capacity to promote HH increased by a average of 21.74 points between 2017 & 2018, from 306.43+96.3 to 328.17+78.9, p<.05. Two MHHIS components were strengthened to a greater extent were "Reminders in the workplace" and "Institutional safety climate". Regarding the overall capacity of hospitals to promote HH, 8-34.8% hospitals were classified as intermediate, 8-34.4% as basic, 6-26.1% as advanced, and 1-4.3% as inadequate in 2017. In 2018, 15-65.2% as intermediate, 5-21.7% at advanced and 3-13% at basic

**Conclusion:** TTT program based on the WHO HHMIS proved to be effective in improving HH promotion, in particular to influence the safety culture in hospitals. It is important to highlight that programs must be implemented to replicate the courses within each hospital, since continuous training favors HH actions among all health workers, not only those participating to the TTT

**Disclosure of Interest**: None declared

## Poster session: Promoting hand hygiene

### P137 IDENTIFYING WHAT CONTRIBUTES TO THE SUCCESS OF INTERVENTIONS TO PROMOTE HAND HYGIENE IN PATIENT CARE: FINDINGS OF A SYSTEMATIC LITERATURE REVIEW AND THEMATIC SYNTHESIS

#### D. Gould, D. Gould

##### Cardiff University, Cardiff, United Kingdom

###### **Correspondence:** D. Gould

**Introduction:** Hand hygiene is the most important way of preventing healthcare-associated infection but is poorly undertaken by health workers. A Cochrane systematic review published in 2017 demonstrated that interventions to improve adherence to hand hygiene protocols are moderately effective but impact varies between organisations and sites.

**Objectives:** Undertake thematic synthesis to identify what contributes to a successful hand hygiene intervention

**Methods:** We undertook thematic synthesis to analyse interpretive information and commentary contained in the Cochrane-included publications to explore how hand hygiene interventions operate their effectiveness and suggest reasons for variations in success.

**Results:** Twenty one papers were reviewed: eleven randomised trials, one non-randomised trial and nine interrupted times series studies. In twenty papers methodological limitations were perceived to affect ability of the intervention to exert its effects. The Hawthorne effect arising when hand hygiene is documented manually was the most important methodological challenge, followed by other sources of bias. In eleven papers need for the intervention to align with local context was perceived to be important. In eleven papers need for organisational support was perceived to be important.

**Conclusion:** Thematic synthesis identified factors likely to affect the uptake of hand hygiene interventions and suggested new directions for research. More accurate and detailed reporting of contextual information in epidemiological studies could help explain how hand hygiene interventions operate in specific settings and help to identify messages to inform sustainability and transferability. Much could be learnt from the emerging disciplines of implementation science and quality improvement. Combining traditional systematic reviews with thematic synthesis maximised understanding of how hand hygiene interventions operate. This approach could improve understanding of how other infection prevention interventions exert their effects.

**Disclosure of Interest**: None declared

### P138 IMPLEMENTATION OF THE WORLD HEALTH ORGANIZATION MULTIMODAL HAND HYGIENE IMPROVEMENT STRATEGY IN A UNIVERSITY HOSPITAL IN MOROCCO

#### S. ELHASSOUNI^1^, M. C. BENJELLOUN^2^, B. OUMOKHTAR^1^

##### ^1^laboratory of microbiology and molecular biology., faculty of medicine and pharmacy; ^2^Department of pneumology, Hassan II University Hospital of Fez, fez, Morocco

###### **Correspondence:** S. ELHASSOUNI

**Introduction:** Hand hygiene (HH) is the most effective practice for health associated infections (HAI’s) prevention; however, this simple gesture is not always respected by healthcare workers (HCW’s)

**Objectives:** aimed to assess the effect of a multimodal improvement strategy on hand hygiene compliance in the Hassan II University Hospital in Fez, Morocco

**Methods:** We conducted a before-and-after interventional study between January and June 2018, in four wards of the University Hospital; two medicine wards, a chirurgical ward and a neonatal intensive care unit (NICU), direct observation was used to evaluate the effectiveness of a multimodal improvement strategy on the compliance of HH according to the “My 5 Moments of Hand Hygiene” defined by the World Health Organization (WHO). The intervention consisted of providing alcohol-based handrub dispensers at points of care, designing educational and training sessions on HH for all HCW’s, placement of general and individual reminders in the workplace and providing feedback on HH performance. Statistical analysis was performed using SPSS 21 for Windows (SPSS, Chicago, IL), and differences between variables were analyzed using the χ2 test

**Results:** A total of 1837 opportunities for HH were observed, with 921 opportunities at baseline and 916 opportunities at follow-up. The overall HH compliance improved significantly from 33,3% at baseline to 49,8% at follow-up (p<.05). Compliance of nurses increased from 33,9% to 53,5% (p<.05), and compliance of physicians improved from 30,3% to 50% (p<.05), however, compliance of nursing assistants (35,9% to 43,5%, p= 0,3) was low and did not improve significantly.

When stratified by indication, compliance with HH improved for all indications after intervention (P<.05) except for “before touching patient” and “after touching patient’s surroundings”

**Conclusion:** The implementation of a multimodal improvement strategy has significantly improved the HH compliance of HCW’s in the Hassan II University Hospital

**Disclosure of Interest**: None declared

### P139 SAVING COSTS BY REDUCING THE DENSITY OF CENTRAL- LINE ASSOCIATED BLOODSTREAM INFECTION BECOMES FEASIBLE THE IMPLEMENTATION OF BEST QUALITY ALCOHOL IN PEDIATRIC INTENSIVE CARE UNITS

#### H. I. G. Giamberardino^1^, P. Krys^2^, J. Y. Kawagoe^3^, on behalf of Hospital Pequeno Principe

##### ^1^Epidemiology and Infection Control Department, Hospital Pequeno Prinicpe; ^2^Epidemiology and Infection Control Department, Hospital Pequeno Principe, Curitiba; ^3^Prof.Master's Degree Nursing, Albert Einstein School of Health Sciences, Sao Paulo, Brazil

###### **Correspondence:** H. I. G. Giamberardino

**Introduction:**

Central- Line associated Blood Stream Infection (CLABSI) is the main quality indicator for pediatric hospitals, especially in neonatal units, and Hand Hygiene (HH) is the primary prevention measure for Health Care-associated Infections (HCAI), but guaranteeing its implementation in the various assistance situations, has been a great challenge. Analyzes of costs of are still little practiced by infection control teams, but their reduction can make investment in prevention possible.

**Objectives:** Analyze the impact of the improvement in the quality of the alcohol-based handrub in CLABSI desinties in three Intensive Care Units of a pediatric hospital and to calculate the possible savings costs that this prevention may bring.

**Methods:** The densities of IPCS were compared in the three ICUs: Cardiac, Pediatric and Neonatal. After the post-implantation (2016-2018) of better quality alcoholic solution in pediatric reference hospital. The cost of the IPCS was composed by the analysis of the number of extra days of hospitalization multiplied by the average cost / day of the patient in each ICU.

**Results:** Comparing the period before and after implantation of the new alcohol-based handrub we observed a reduction in the densities of CLABSI: 57.2% Cardiac ICU, 47.7% Pediatric ICU and 43.5% Neonatal ICU. The alcohol-based handrub consumption showed an average increase of 10% when compared with the pre-implantation and post-implantation period. The mean cost of CLABSI in these ICUs ranged from US $ 2500 to US $ 9000.00 and the average number of extra days of hospitalization was 10.4 days.

**Conclusion:**

Although there are other factors that interfere with the occurrence of IPCS, as a technique for insertion and manipulation of the central catheters, we observed that with the improvement of alcohol-based handrub quality, there was better adhesion to HH in these units and it was associatedwith the reduction in CLABSI desinties. Cost analysis and its preventive potential should also be considered and may helpin the allocation of resources that are already scarce in the health area, for the incorporation of better products and medical-hospital materials.

**Disclosure of Interest**: H. Giamberardino: None declared, P. Krys: None declared, J. Kawagoe Employee of: Patient Safety Consultant BBraun

### P140 CAN THE CONSUMPTION OF ALCOHOL-BASED HANDRUB (ABHR) BE AN INDICATOR OF HAND HYGIENE (HH) COMPLIANCE?

#### A. Iten, J. Sztajzel, A. Peters, D. Pittet

##### Infection Control Program, HUG, Geneva, Switzerland

###### **Correspondence:** A. Iten

**Introduction:** WHO recommends regular assessment of hand hygiene (HH) compliance by healthcare workers during their care activities. Such assessment of the HH behavior of caregivers is essential for evaluating the success of an institution’s HH promotion strategy. Quantifying the consumption of the alcohol-based handrub (ABHR) formulation(s) is often considered a surrogate marker of HH compliance in[IA1] hospitals that don’t have the means to observe sufficiently.

**Objectives:** We compared HH compliance data and ABHR consumption collected from 2005 to 2018 at HUG

**Methods:** HH compliance is assessed by observers trained according to WHO recommendations (“My 5 Moments for HH”). The ABHR consumption is measured by the volume of ABHR delivered each year by HUG hospital pharmacy to the different wards and departments, expressed in L/1000 patient-days. We divided the overall time period into 3 phases (2005-2007; 2008-2010; 2011-2018) to account for the 2009 H1N1 pandemic typically associated with increased ABHR worldwide.

**Results:**

**Table:** 

	Years (n)	Compliance rate at HH (%)	ABHR distribution (L/ 1000 day-patients)
**2005-2007** Implementation phase	1	57	25.4
	1	65	30.3
	1	66	35.0
**2008-2010** Pandemic 2009	1	61	35.9
	1	62	50.3
	1	64	40.2
**2011-2018** Stabilization phase	1	67	43.3
	3	69	47.7 Min. 47 ; Max. 48.1
	4	70	44.2 Min. 43.2 ; Max 45.6

**Conclusion:** According to our results, the consumption/distribution of ABHR should not be considered as an indicator for HH compliance. It reflects the distribution of ABHR in a healthcare facility and should be interpreted according to the circumstances and the stage at which the HH promotion strategy is.

**Disclosure of Interest**: None declared

### P141 MEASURING HAND HYGIENE PERFORMANCE BASED ON ALCOHOL-BASED HAND RUB USAGE IN HOSPITALS: A CROSS- BORDER PROJECT (I-4-1 HEALTH)

#### M. Verelst^1^, I. Willemsen^2^, V. Weterings^2^, A. Schuermans^3^, I. Leroux-Roels^4^, J. Kluytmans^5^ on behalf of i-4-1 Health study group

##### ^1^Infection control, UZ Leuven, Leuven, Belgium; ^2^Infection control, Amphia hospital, Breda, Netherlands; ^3^Infection control, University hospital, Leuven; ^4^Infection control, University hospital, Gent, Belgium; ^5^Julius Center for Health Sciences and Primary Care, UMC Utrecht, Utrecht, Netherlands

###### **Correspondence:** M. Verelst

**Introduction:** Observation of healthcare workers is considered the golden standard to evaluate hand hygiene compliance (HHC). However, HHC can be threefold higher in eyesight of an auditor. Another indicator can be the consumption of alcohol based hand rub (ABHR).

**Objectives:** The goal of this study was to get more insight in the hand hygiene performance in different hospitals and countries by using the ABHR indicator.

**Methods:** Nine hospitals were asked to collect data on ABHR usage and patient days. The number of disinfection moments performed by patient day was calculated by dividing the volume of ABHR per patient days by the volume of ABHR per disinfection moment (approximately 2.5 ml).

**Results:** ABHR consumption data was collected over a period of time of at least 6 months, in 3 university hospitals (12 wards) and 3 general hospitals (12 wards). Three hospitals were not able to deliver data. The median number of disinfection moments per patient day was 10, with a range of 4 to 20. The median number of disinfection moments per day was significantly higher in university hospitals (13) than in general hospitals, 6 (p<0.001). The median amount of disinfection moments per patient day differed per medical specialty, however not significantly (cardiology and internal medicine 6; orthopaedics 10; surgery and urology 11; pulmonary diseases 13 and geriatrics 14).

**Conclusion:** This study showed that the majority of the hospitals (6) were able to collect consumption data of ABHR and gain objective information about HH performance. Large differences in ABHR use and number of disinfection moments between hospitals were found, with a significant difference between university and general hospitals.

**Disclosure of Interest**: None declared

### P142 IMPLEMENTATION OF HAND HYGIENE PROGRAM IN TRNAVA UNIVERSITY HOSPITAL DURING PERIOD 2013-2019

#### J. Prnova^1,2,3^, V. Rusnakova^1^, J. Brnova^2,3^

##### ^1^Department of Public Health, School of Health Sciences and Social Work, Trnava University, Slovakia; ^2^Department of Hospital Hygiene and Epidemiology, University Hospital Trnava, Slovakia; ^3^Centre of Microbiology and Infection Prevention, Trnava University, Slovakia, Trnava, Slovakia

###### **Correspondence:** J. Prnova

**Introduction:** Hand hygiene is a primary strategy to prevent healthcare-associated infections and the spread of antimicrobial resistance. The application of hand hygiene improvement program is required in Slovakia, but results are still not enough.

**Objectives:** Our objective was to evaluate the impact of implementing World Health Organization’s Multimodal Hand Hygiene Improvement Strategy in University Hospital Trnava.

**Methods:** We conducted before and after study in 638 beds University Hospital Trnava during 2013-2019. Key strategies included providing and revisiting alcohol-hand rub dispenser at the department, designed educational program, reminders for hand hygiene was placed and evaluated for hand hygiene compliance and alcohol hand rub consumptions.

**Results:** Alcohol hand rub consumptions significantly increased during 2013 to 2018 from 15.7 l/1,000 patient day up to 24.3 l/1,000 patient day (p<0.05). There was significant improvement of hand hygiene compliance from 39.9% (95% CI: 37.4 – 41.9) to 61.2% (95% CI: 58.2 – 65.4), (p<0.05).

**Conclusion:** Even though alcohol hand rub consumption has been increasing, compliance of hand hygiene among health care workers was found to be still low, but during interventions increased. The findings suggest continuous monitoring, education and immediate feedback are critical to success hand hygiene program.

**This work was supported by a research grant from the MŠVVaŠ SR.**

**Disclosure of Interest**: None declared

### P143 SUCCESSFUL IMPROVEMENT OF HAND HYGIENE COMPLIANCE RATE BY IMPLEMENTING MULTIMODAL INTERVENTIONS AT TERTIARY HEALTHCARE CENTER IN SAUDI ARABIA

#### N. A. Bouafia^1^, W. A. Mazi^1^, M. A. Hamdi^1^, S. H. Alwagdani^1^, H. AlSofieni^2^, A. M. Dahlawi^3^, M. R. ALYAMI^4^

##### ^1^INFECTION PREVENTION AND CONTROL, KING FAISAL MEDICAL COMPLEX; ^2^DELIVERY DEPARTMENT; ^3^MEDICAL DIRECTOR, MATERNITY TOWER - KING FAISAL MEDICAL COMPLEX; ^4^HOSPITAL DIRECTOR, KING FAISAL MEDICAL COMPLEX, TAIF, Saudi Arabia

###### **Correspondence:** N. A. Bouafia

**Introduction:** Hand hygiene (HH) is a growing concern among populations and is a crucial element in ensuring patient safety and prevention of healthcare associated infection.

**Objectives:** To reach a HH compliance rate of 80% within 3 months

**Methods:** Maternity Tower is a new building opened on January 2018 and annexed to King Faisal Medical complex – Taif, KSA. HH compliance rate monitored, during the first trimester 2018 (pre-interventional period), by direct observation of health care workers (HCW), using WHO Observational Tool, was about 63%. A quality improvement project based on FOCUS Plan-Do-Check-Act (PDCA) strategy was decided to be implemented particularly in area with low compliance rates. Delivery ward (DW) had the lowest rate with a pooled mean of 50% (61% nurses, 42% doctors).

An anonymous questionnaire was distributed to HCW (n=51) in order to understand barriers to HH compliance and guide the quality improvement team to implement the appropriate interventions.

**Results:** 40 HCW completed the questionnaire. Lack of knowledge (78.8%), skepticism of the value of hand hygiene (77.5%), simple forgetfulness (72.5%) and lack of role model (58.8%) were the most frequent factors affecting HH compliance.

Interventions implemented were: HH Campaign based on daily HH awareness in-service walk round, weekly educational sessions using WHO simulation scenario, monthly feedback about HH compliance, competitive creativity and educational messages (video, posters, brochures, etc.) with monthly awardees of HCW and involvement of patients in the project.

Thus, the H.H compliance rate reached 80 % in June 2018 with respectively 81 % and 78% for Nurses and doctors.

**Conclusion:** The objective of our project was achieved for nurses. Continuous training education is undergone hospital wide, mainly for doctors to achieve the MOH standard (80%)

**Disclosure of Interest**: None declared

### P144 HAND HYGIENE EXCELLENCE AMERICA LATINA(HHEA-LATAM): EXPERIENCE OF A PEDIATRIC HOSPITAL

#### H. I. G. Giamberardino^1^, J. Y. Kawagoe^2^, A. P. O. Pacheco^3^, B. D. Matucheski^1^, L. P. Carvalho^1^ on behalf of Hospital Pequeno Principe - Department of Epidemiology and Infection Control

##### ^1^Epidemiology and Infection Control Department, Hospital Pequeno Principe, Curitiba; ^2^Professional Master's Degree in Nursing, Albert Einstein Israelite School of Health Sciences, Sao Paulo; ^3^Epidemiology and Infection Control, Hospital Pequeno Principe, Curitiba, Brazil

###### **Correspondence:** H. I. G. Giamberardino

**Introduction:** Hand hygiene (HH) is the main pillar for the prevention of *Health Care*-*Associated Infections* (HCAIs) and in the Pequeno Principe Hospital (HPP) is considered a strategic institutional goal.

**Objectives:** Describe the actions to apply for HHEA-LATAM 2018.

**Methods:** The HPP is the largest pediatric hospital in Brazil, Curitiba-PR.It is philanthropic and university hospital, with 360 beds, 66 of which distributed in four intensive care units In September of 2017 we submited form to the HHEA-LATAM, a platform to identify hospitals and groups who improving patient safety, this process was developed by WHO and the Collaborating Centre on Patient Safety, University of Geneva Hospitals . In August 2018 experts in infection control evaluated hospital performance.The HPP has progressively implemented the five key components of the WHO multimodal HH improvement strategy The preparation for this evaluation involved all hospital staff, within 11 months period.

**Results:** Several actions were implemented and intensified: 1) relocation of HH products 2) improvement in the quality of the alcohol-based handrub;3) training in the admission of new employees, for multidisciplinary teams, academicians and residents of medicine, pharmacy, nursing, biomedicine, volunteers and family members ;4) improvement of HH indicators, consumption of alcohol-based handrub by patients-day and units,adherence to the five moments of HH, degree of family satisfaction with HH by HCWs; 5) created the "vigiometer" specific form to share the indicators with each unit / team, 6) periodic updates on HH reminders and educational materials 6)innovative approaches such as global icon (Pelé) involvement in video showing that every time the HCW practices HH is making a "goal" for the prevention of HCAI.

**Conclusion:** Being one of the winning hospitals was a great learning opportunity, reinforcing the importance of continuous self-evaluation over the time, and more future responsibility in the continuous search for excellence in HH.

**References**

Guide to Implementation of the WHO Multimodal Hand Hygiene Improvement Strategy(https://www.who.int/gpsc/5may/Guide_to_Implementation.pdf)

**Disclosure of Interest**: H. Giamberardino: None declared, J. Kawagoe Employee of: Patient Safety Consultant BBraun, A. P. Pacheco: None declared, B. Matucheski: None declared, L. Carvalho: None declared

### P145 IMPROVING HAND HYGIENE COMPLIANCE AND REDUCING HEALTHCARE-ASSOCIATED INFECTIONS: LEADERSHIP AND MANAGERIAL SUPPORT CREATES THE TIPPING POINT

#### B. Du Toit, on behalf of Corporate Hand hygiene Committee

##### Clinical, Mediclinic Southern Africa, Stellenbosch , South Africa

###### **Correspondence:** B. Du Toit

**Introduction:** Hand hygiene is one of the most important interventions to prevent healthcare-associated infections (HAIs). Compliance is often suboptimal, contributing to the spread of multidrug resistant pathogens and the development of HAIs. Improving compliance is ongoing and requires leadership and managerial support.

**Objectives:** To reduce HAIs by improving hand hygiene compliance.

**Methods:** Improving compliance has been seen as the sole responsibility of the IPC practitioner. In 2017 a project was initiated to improve hand hygiene compliance and clinical outcomes in 52 hospitals in Southern Africa. A task team, consisting of the Chief Operating Officer, Chief Clinical Officer and senior managers developed a strategy, based on the multimodal hand hygiene strategy of the World Health Organisation (WHO). Interventions included a baseline knowledge and facility assessment utilising the tools provided by the WHO. A system change ensured that alcohol handrub is available at the point of care and training focused on practical scenarios to ensure theory practise integration. An electronic tool was developed to capture compliance and the institutional safety climate was enhanced by adding hand hygiene as a key performance indicator, displaying compliance rates and discussing it at meetings. The strategy and a position paper were circulated to doctors. A Pearson’s correlation and simple linear regression test was performed, utilising STATA software to establish whether there was a statistical significant correlation between improvement in hand hygiene compliance and reduction in HAI rate.

**Results:** Since the commencement of the project 6053 healthcare workers were trained on hand hygiene. Compliance amongst healthcare workers improved with 33.4% from 56% in June 2013 to 74.4% in April 2019. A statistical significant inverse correlation between hand hygiene compliance and reduction in HAI rate was furthermore observed (R2 = 0.14, p=0.001). No statistical significant shift has been observed in the use of alcohol handrub compared to hand washing.

**Conclusion:** It is not sufficient to only improve the knowledge of staff and to provide alcohol at the point of care to improve compliance. Management and leadership support are critical factors for sustainable improvement in compliance.

**References**

**Disclosure of Interest**: None declared

### P146 THE RIGHT ACTION AT THE RIGHT TIME: ANALYSIS OF SPECIFIC HAND HYGIENE CONDUCTS AND ITS INFLUENCE IN THE IMPLEMENTATION OF LONG-TERM HAND HYGIENE BUNDLES IN MEXICO

#### O. Y. Bello-Chavolla^1^, M. A. Guedez^2^, M. E. Navarrete-Romero^2^, J. A. Pacheco-Martínez^2^

##### ^1^Universidad Nacional Autónoma de México; ^2^B.Braun Mexico, Mexico City, Mexico

###### **Correspondence:** O. Y. Bello-Chavolla

**Introduction:** Hand hygiene bundles (HHBs) are strategies to facilitate implementation of the WHO multimodal strategy to increase HH compliance (HHC). The hand-rub (HR) to handwashing (HW) ratio (HHr) as recommended by WHO consists of 5 actions of HR per action of HW to decrease time dedicated to HH and increase HHC.

**Objectives:** To demonstrate that HHr compared to HHC rates is a better index of resistance to changes in HH conducts and thus a better marker of the efficacy of HHBs implementations, using evidence from real-life implementation of HHBs in Mexico.

**Methods:** We conducted a 3-year follow-up in three medical facilities in which HHBs were implemented. Monthly direct observation measurements were carried out by trained personal according to WHO’s HH moments and health personnel. HH actions were registered as HR with alcohol solution, HW, omission and inappropriate use of gloves. The HHr was calculated as well as HHC rates during follow-up.

**Results:** During the first 12 months of follow-up we observed a significant increase in HHC with steady increases from baseline HHC between 10-20% to rates ranging 45-55% (R^2^=0.6579) with an average HHr of 1.8. The second period of assessment from months 12-24 saw a decrease in the rate of HHC increases from 45-55% to 55-65% (R^2^=0.1099, an 18% increase); in contrast, the HHr had an average of 3.5 actions of HR per HW actions, representing a 94% increase from the previous period. Finally, during the third year HHC rates remained steady, oscillating between 55-70% in all facilities (R^2^=0.0034); nevertheless, we observed a steady increase in the HHr (R^2^=0.5538) with an average of 4.5 HR per HW actions, representing a 28.6% increase. When assessed by personnel category, nurse personal had steadier increases in HHC rates but not HHr (p<0.001), indicating higher resistance to change compared to physicians and in-training personnel. We observed no direct relationship between HHC rates and the HHr in any of the evaluated facilities.

**Conclusion:** HHBs for the implementation of the multimodal strategy of WHO to increase HHC has the potential to offer strategic recommendations per service, personnel category and HH moment using the HHr as an indicative of resistance to change instead of HHC rates.

**Disclosure of Interest**: O. Y. Bello-Chavolla Consultant for: B. Braun Mexico, M. Guedez Employee of: B. Braun Mexico, M. Navarrete-Romero Employee of: B. Braun Mexico, J. A. Pacheco-Martínez: None declared

### P147 NEW ZEALAND HAND HYGIENE PROGRAMME: REQUIREMENTS TO SUSTAIN IMPROVEMENT IN A CHANGING ENVIRONMENT

#### S. Roberts, N. Grae

##### Infection Prevention & Control Programme, Health Quality & Safety Commission, Wellington, New Zealand

###### **Correspondence:** S. Roberts

**Introduction:** A national hand hygiene programme (HHNZ) across all publicly-funded hospitals started in 2011. Public reporting of performance started in July 2012. HHNZ provided coordination, communication expertise, clinical leadership and auditor training. In February 2016 it was felt that the necessary structure was in place for HHNZ to be self-sustaining and funding was reduced. Three years later we reflect on the outcome of this decision.

**Objectives:** We reflect on the essential requirements to sustain a successful national hand hygiene programme.

**Methods:** Qualitative information was gathered from webinars held with hand hygiene coordinators in 2017, follow-up interviews with individual hand hygiene coordinators in late 2017-2018, feedback at a national workshop in 2018, discussions at regional meetings, and through discussion with hand hygiene coordinators unable to sustain nationally agreed auditing requirements. This allowed the key challenges to sustaining a successful programme at a local level to be identified.

**Results:** The success of the regional networks was variable. Outside of the main centres the geographical distance between some hospitals was an impediment to participating in regional networks. As a result the support for training of auditors by the regional network was limited. Equally, there were limited auditor training opportunities throughout the year and the demand exceeded the capacity for auditor training. For smaller hospitals, if key staff left, they often struggled to maintain the programme.

Without national coordination, oversight of gold auditors was not optimal; annual validation was not always undertaken, nor did newly trained gold auditors remain active auditors suggesting that the selection of auditors may not be optimal.

Public reporting and national leadership for activities such as World Hand Hygiene Day and Patient Safety Week were seen as key drivers for changing behaviour.

**Conclusion:** The key elements required to support the HHNZ programme were identified. These include a nationally-led process to support the training of gold auditor trainers and of gold auditors and also to ensure annual validation by gold auditors. Regular updates, either via online meetings or face-to-face workshops, are necessary to ensure hand hygiene remains a priority regardless of staff turnover.

**Disclosure of Interest**: None declared

### P148 A SUCCESSFUL HAND HYGIENE PROGRAM IN SAUDI ARABIA

#### B. Molaeb

##### Infection Prevention and Control, Almoosa Specialist Hospital, Al-Ahsa, Saudi Arabia

**Introduction:** Hand hygiene (HH) is the primary measure to reduce infections. ​Improving HH compliance and implementing sustainable interventions constitute major challenges in healthcare.

**Objectives:** To provide an overview of a successful HH program that improved HH compliance for healthcare workers (HCW) and increased HH awareness in community.

**Methods:** Beginning in 2016, new multimodal strategies for HH improvement based on the World Health Organization (WHO) initiatives were implemented. These included adopting the WHO HH surveillance tool, introducing interactive HH training tools, developing goals for compliance, using visual HH reminders, ensuring leadership support, providing performance feedback, rewarding compliant HCWs, conducting massive HH awareness campaigns and introducing mobile app technology for direct observations in all units. Hospital-wide HH observations were collected and analyzed. Patient feedback on HCWs’ HH practices was obtained through Press Ganey surveys. Z-test was used to compare the percentage change and a p-value less than 0.05 was considered significant. Community HH activities included booths in the facility, visits to schools, universities and parks. In May 2018, an attempt to achieve Guinness World Record (GWR) for the largest HH lesson was made. In May 2019, the campaign was concluded by forming a large human image of a hand.

**Results:** The overall HH rate increased from 76% in 2015 to 87% in 2018 (14.5% increase, p-value<0.05) and has been maintained above that level till date. HH compliance increased by 24% for both doctors and nurses, 32% for technicians and 39% for other HCWs. Patient satisfaction with HCW’s HH practice increased from 75% in 2015 to 89% in 2018 (19% increase, p- value<0.05). Community campaigns covered 6,300 individuals, achieved GWR with 714 participants and included 300 persons forming the largest human image of a clean hand in Saudi Arabia.

**Conclusion:** Multimodal innovative approaches to HH measurement, monitoring and education have been successful in improving HH compliance rates, maximizing community coverage and resulting in sustained improvement.

**Disclosure of Interest**: None declared

### P149 IMPLEMENTATION OF THE WHO HAND HYGIENE STRATEGY IN FARANAH REGIONAL HOSPITAL, GUINEA

#### A. Diallo^1^, S. Müller^2^, SA. Müller^1^, AOK. Diallo^2^, R. Wood^1^, O. Tounkara^2^, M. Arvand^1^, M. Diallo^2^, M. Borchert^1^

##### ^1^PASQUALE, PASQUALE, Faranah, Guinea; ^2^PASQUALE, RKI, Berlin, Germany

###### **Correspondence:** A. Diallo

**Introduction:** Nosocomial infections are the most frequent adverse events in healthcare worldwide, with limited available evidence suggesting highest burden in resource-limited settings. Recent epidemics emphasize the disastrous impact that spread of infectious agents within healthcare facilities can have, accentuating the need for improvement of infection control practices. Hand hygiene (HH) measures are considered to be the most effective tool to prevent nosocomial infections. However, HH knowledge and compliance are low, especially in vulnerable settings such as Guinea.

**Objectives:** To assess knowledge and compliance with HH, improve HH and strengthen hospital performance by incorporating the WHO HH Strategy including the local production of alcohol-based hand-rub (ABHR).

**Methods:** All currently contracted healthcare workers in the Faranah Regional Hospital (HRF) were invited to participate. A baseline assessment of HH knowledge, perception and compliance was performed 12 months before the intervention. The tailored intervention consisted of a training adapted to the needs identified in the baseline assessment and the reintroduction of local production of ABHR. A first follow-up assessment was conducted directly after the intervention, with a second planned at the six month interval. Effectiveness of the implementation was assessed via before-and-after comparison.

**Results:** The survey included a vast majority of all health care workers. Baseline knowledge and compliance were low (52.4%; 23.4%, respectively), but showed a significant increase (75.6%; 71.3%; respectively) upon follow-up. The reintroduction of ABHR production resulted in a four-fold increase of monthly hospital consumption. The WHO HH Strategy was rated to be effective in perception surveys throughout.

**Conclusion:** This study demonstrated that the WHO HH strategy is an adaptable and effective method to improve HH knowledge and compliance in the resource-limited setting of HRF. Local production is a feasible method for providing self-sufficient supply of ABHR to regional hospitals such as HRF. Participatory approaches, such as hygiene committee ownership of regular trainings and project-specific tasks, enhance prospects of sustainability.

**Disclosure of Interest**: None declared

### P150 IMPROVEMENT OF HAND HYGIENE (HH) COMPLIANCE, USING THE WHO MULTIMODAL STRATEGY: RESULTS FROM A ROMANIAN HOSPITAL

#### T. Ionescu^1^, N. Georgescu^1^, D. Bandrabur^1^, E. Tartari^2^, L. Stingaciu^1^, D. Rusu^1^, M. Muscalu^1^, F. Staicu^1^, M. Florica^1^, N. Nitu^1^, C. Mocanu^1^, D. Dunca^1^, M. Vengher^1^, H. Rahimian^1^, C. Palade^1^, D. Pittet^2^

##### ^1^Baneasa Hospital, Regina Maria Private Healthcare Network, Bucharest, Romania, ^2^The University of Geneva Hospitals and Faculty of Medicine, Geneva, Switzerland

###### **Correspondence:** T. Ionescu

**Introduction:** The hospital has enrolled in an international accreditation system for quality and patient safety. To date no HH compliance data have been published in Romania.

**Objectives:** We describe our HH improvement programme and compliance trends between 2013-2018.

**Methods:** Between 2013 and 2016, we developed checklists for the quality audits, including a programme for HH. In 2017, HH compliance data were collected regularly based on the WHO HH observation form by trained practitioners. Alcohol based hand rub (ABHR) was introduced at the point of care at the start of the HH programme and the number of ABHR dispensers has increased progressively. In 2018, the Ministry of Health pledged to join the WHO *Clean Care is Safer Care* to promote best HH practices in the country. The hospital registered for the WHO *Save Lives: Clean Your Hands* campaign and has applied for the Hand Hygiene Excellence Award (HHEA).

**Results:** The Joint Commission International (JCI) accredited the hospital in 2014 and 2017. Reducing the risk of healthcare-associated infections through hand hygiene is one of the six mandatory International Patient Safety Goals of JCI. A total of 400 HH opportunities were observed in 2013, while a three-fold increase was recorded in 2018 (1500). We developed educational materials and HH courses for healthcare workers. Audit sessions showed increased compliance in 2014 (90%), the year of an international accreditation, and lower rates in the following years. At the introduction of WHO observation form, in 2017, HH compliance averaged 74%, peaking at 87.5% before a re-accreditation visit. The highest compliance rate (95%) was achieved in 2018, at the time of the HHEA experts visit. ABHR has increased to more than 40L/1000 pt-days. The programme at our hospital has extended throughout the entire network (hospitals and outpatient).

**Conclusion:** The international accreditation process and the HHEA enrollment have been instrumental in improving HH compliance, but sustainability is a continuous challenge. Continuous initiatives and leadership commitment are significant.

**Disclosure of Interest**: None declared

### P151 EFFECTIVE IMPLEMENTATION OF THE WORLD HEALTH ORGANISATION MULTIMODAL HANG HYGIENE IMPROVEMENT STRATEGY IN A INTENSIVE CARE UNIT OF A UNIVERSITY HOSPITAL IN MADAGASCAR

#### S. Gaimard^1^, P. Nerrant^1^, T. Rakotovao^2^, C. Andrinalimanana^2^, C. Guittard^3^, L. Di Trapani^1^

##### ^1^PAH, pharmaciens humanitaires, Paris, France; ^2^CHU Tambohobe, Fianarantosa, Madagascar; ^3^spci, HUG, Genève, Switzerland

###### **Correspondence:** S. Gaimard

**Introduction:** Each year, hundreds of millions of patients around the world are affected by health care-associated infections (HAI), according to the World Health Organisation (WHO). Adequate hand hygiene (HH) is considered the most effective measure to prevent HAI^1^. Lack of hygiene products and access to drinking-water are the main reasons for failure in HH compliance in Madagascar.

**Objectives:** We assessed the effectiveness of the WHO multimodal HH improvement strategy (MHHSI) in an Intensive Care Unit (ICU) in a university hospital in Madagascar.

**Methods:** We conducted a before and after study from January to March with a pre and post-intervention period of 2 weeks. The intervention was an adaptation of the WHO MHHSI to the local requirements. It consisted of introducing a locally manufactured alcohol-based handrub (ABHR), designing an educational program for health care workers (HCW), providing monitoring and feedback and posting reminders at workplaces. We evaluated HCW knowledge and perceptions; ABHR access; HH ‘WHO 5 moments’ and prerequisite compliances and ABHR consumption.

**Results:** HCW knowledge increased with an average score of 61% before and 83.5% after the educational program. Even if HCW have a good understanding of HH theoretical prerequisites (average mark of 82%), only 44% apply it correctly after our intervention. HH compliance increased from 18% before to 35% after our intervention (p<0.0018). In March 89% of HCW said they had better access to ABHR due to 30% reduction in price for patients and a monthly free endowment of 100mL per HCW. ABHR consumption enhanced from 0.95L/month to 6L/month.

**Conclusion:** Setting up WHO’s MHHSI promotion campaign allowed us to make HCW aware of HH, to increase ABHR’s provision in the ICU, and also their compliance to HH.However we need to continue to promote HH and extend this project to the entire hospital. To maintain and pursue this project, we must introduce an institutional safety climate with regular teaching courses and audits throughout the hospital.

**References**

^1^ *Pittet D, et al. Effectiveness of a hospital-wide programme to improve compliance with hand hygiene. Infection Control Programme’ Lancet. 2000;356(9238):1307–1312.*

**Disclosure of Interest**: None declared

### P152 IMPACT OF HAND HYGIENE COMPLIANCE IN VARIOUS WARDS IN KABALE REGIONAL REFFERAL HOSPITAL

#### E. John, B. LILIAN, M. Namutebi, S. Namasopo, F. Kizito

##### SURGERY, MINISTRY OF HEALTH UGANDA, KABALE REGIONAL REFERRAL HOSPITAL, KAMPALA/KABALE, Uganda

###### **Correspondence:** E. John

**Introduction:** The WHO guide lines 2017 posed question, how significant is the problem of infection s in the health care across the world and suggested that the problem is huge, and estimated that about 1.4 million cases of hospital acquired infections among others surgical site infections and said it was likely to be low due to lack of proper surveillance. Most cases these infections are attributed to poor hand hygiene among other causes. Hand hygiene compliance is still a challenge in Kabale Regional Referral Hospital, with 24% compliance rates in 2016 to 2018.

**METHOD**: Introduction of quality improvement project on hand hygiene compliance by staff and students which identified the following tested changes at various service delivery points. Training of the appointed staffs on how to manufacture locally Alcohol Based Hand Rub (ABHR), Installation of the ABHR Dispensers, monthly continuous medical educations, monthly monitoring of hand hygiene compliance using the World Health Organization (WHO) standard checklist and Monthly feedback to the various wards.

**RESULTS**: Proper hand hygiene compliance among staff and students has resulted in reduced levels of hospital acquired infections and reduced risks of contracting contagious infections in wards with 50% improvement in 2019.

**DISCUSION**: Hand hygiene compliance is the first and easiest way of addressing the risks of contracting contagious infections reduction on the levels of cross infections and also has reduction on anti-microbial resistance in Kabale Regional Referral Hospital.

**CONCLUSION**: Hand hygiene compliance is the first and easiest way of reducing the spread of the infections among staffs, students and patients at large only if absorbed everywhere in addressing gaps in infection prevention and control in various health care settings.

**Disclosure of Interest**: None declared

### P153 ADAPT TO ADOPT: SPREADING THE MESSAGE OF HAND HYGIENE THROUGH A SONG

#### A. Peters, C. Guitart, T. Borzykowski, F. Timurkaynak, M. De Kraker, D. Pittet

##### SPCI, HUG, Geneva, Switzerland

###### **Correspondence:** A. Peters

**Introduction:** Finding new ways to increase compliance with hand hygiene (HH) and avoid campaign fatigue is central to sustain improvement. Implementation of interventions that target healthcare worker behavior change is challenging. As the largest patient safety initiative in the world, the global HH promotion campaign *SAVE LIVES: Clean Your Hands* has been a leader in creative approaches to keeping a worldwide audience engaged.

**Objectives:** To create a song for promoting HH worldwide and to implement an assessment approach to compare several HH campaign communication tools.

**Methods:** This year, in honor of World HH Day on May 5th, the WHO Collaborating Center on Patient Safety at the University of Geneva Hospitals composed an original song incorporating key messages and slogans defined by WHO. We produced a video and shared it on YouTube with our contacts around the world. We studied the diffusion of the video on several social media and compared it to prior initiatives and tools we developed in the past.

**Results:** The video for “It’s in Your Hands” was posted online on 24 April 2019, and has been seen over 28,000 times (28,342 as of 25 May; www.tinyurl.com/ItsInYourHands). Numerous countries have already requested the translations; and a hospital team in Vietnam even translated and recorded the song and made a music video in Vietnamese. Currently, we are working on a promotion strategy and on making subtitles in different languages so that people around the world can adapt the message to fit their own social context. Data will be presented to highlight the spread of the song, as a marker of the HH campaign endorsement, and compared to other indicators of the *SAVE LIVES: Clean Your Hands* campaign spread at 1, 2, 3, 4 months, and include predictions at 6, 12, 18 and 24 months. Comparisons will be made between the spread of the song “It’s in Your Hands” launched in 2019 and the HH dance (> 0.5 million view on YouTube; launched on the first World HH Day, 5 May 2009).

**Conclusion:** Incorporating innovative tools such as songs can be an effective way to help engage healthcare workers and institutions. A possible next step would be to quantify the impact that such an engagement.

**Disclosure of Interest**: None declared

### P154 FIVE MOMENTS TO CARE: ASSESSING ROLE OF SEQUENTIAL MMI ON HCWS’ 5 MOMENTS FOR HAND HYGIENE IN BANGLADESH

#### L. Ara, F. Hossain, M. E. H. Tamal, M. Ibrahim, M. R. H. Khan Abir, M. N. H. Alam, M. S. A. Sarker

##### Nutrition and Clinical Services Division, icddr,b, Dhaka, Bangladesh

###### **Correspondence:** L. Ara

**Introduction:** Adequate adherence to hand hygiene (HH) is a global healthcare challenge. To enhance this fundamental infection prevention and control (IPC) practice among healthcare workers (HCWs), WHO recommended a multimodal design and five prime moments (FPMs) for HH.

**Objectives:** Our study aimed to evaluate the role of individual components of multimodal intervention (MMI) in improving HCWs’ HH compliance (HHC) to the FPMs in Bangladesh.

**Methods:** A quasi experimental study was conducted in 2 intervention and 1 control hospitals of Bangladesh. Data were collected at baseline, endline and the consecutive weeks following implementation of each MMI component (system change, education and training, visual reminder, feedback/incentive) in sequence. Doctors and nurses were directly observed practicing HH in the FPMs: (1) before touching a patient, (2) before aseptic procedure, (3) after risk of fluid exposure, (4) after touching a patient and (5) after touching patient surroundings. Additionally, the feedback component of MMI was replaced with incentive provision (certificate) in one intervention hospital.

**Results:** A total of 6223 HH opportunities were observed from both arms. At intervention arm, doctors’ HHC exhibited maximum improvements to Moments 2 (from 4.7% to 76.6%, OR:66.9, CI:25.3-176.8, *p*<0.01) and 3 (from 8.0% to 81.6%, OR:50.3, CI:23.1-111.6, *p*<0.01) after education and training (ET) intervention. Similarly, nurses adhered to HHC more in Moments 2 (from 1.9% to 38.9%, OR:33.8, CI:10.3-110.9, *p*<0.01) and 3 (from 2.5% to 42.2% OR:29.0, CI:10.2-82.3, *p*<0.01) following implementations of visual reminders (VR) and ET interventions respectively, while the control arm remained unaltered. HHC was more complaint in incentive (13.3%) than the feedback arm (8.8%).

**Conclusion:** Study findings substantiate the success of ET and VR components of MMI in enhancing HCWs’ HHC to Moments 2 and 3. Therefore such evidence-based strategies can be further incorporated in healthcare policies of Bangladesh and other resource-constrained settings for effective IPC.

**Disclosure of Interest**: None declared

### P155 HANDHYGIENE COMPLIANCE WITH THE FIVE MOMENTS BY HEALTH WORKERS IN BENIN HOSPITAL’S

#### T. A. Ahoyo^1^, S. ASSAVEDO^2^, P. F. D. FONTON^1^

##### ^1^BIOLOGIE HUMAINE, EPAC/UAC, ABOMEY CALAVI; ^2^MINISTERE SANTE CABINET, MINISTERE SANTE, COTONOU, Benin

###### **Correspondence:** T. A. Ahoyo

**Introduction:** In the context of outbreak of LASSA fever in Benin 2014, WorldHealth Organization and other partners, have initiated and supported the implementation of a program to prevent and control infection. A national hand hygiene promotion campaign based on WHO multimodal strategy was launched in 2015.

**Objectives:** We assessed the feasibility and compliance with the five moments for hand hygiene by healthworkers in Benin hospitals

**Methods:** The study was carried out between June 2015 to September 2018 in ten (10) tertiary hospitals included in IPC progam. Data were collected in 10 intense care units using methods and tools provide by WHO, a particular attention was paid to handling devices such as intravenous catheter, urinary catheter and dressing. We used R version 3.5.2 for statistics

**Results:** HCW’S compliance with the five moments for hand hygiene increased from baseline **16.83 %** CI 95% (16.32- 17.36) to **40. 2**% CI 95% (39.6 - 41.1), the indication 3 was the most observed from baseline **31%** CI 95% (29.49 - 32.64) to **55.7** % CI 95% (54.21 - 57.3), The most commonly observed handlings were the manipulation of venous catheters 63,2%, the higtestest compliance was noticed with the manipulation of dressing 75, 3 %. Bloodstream infection decrease from **18%** CI 95% (17.09- 18.26 to **4.8%** CI 95% (4.49- 5.04)

A wide variation in handhygiène compliance among hospitals was observed, the most important result was the lower incidence of blooostream during these 3 years

**Conclusion:** Efforts to involve more hospitals in hand hygiene program should be intensified,with local financing

**Keywords: Patient safety, World Health Organisation, Behavior change, Hand hygiène**

**References**

1 Allegranzi B, Sax H, Bengaly L, et al. Successful Implementation of the world health organization hand hygiene improvement strategy in a referral hospital in Mali, Africa. Infect Control Hosp Epidemiol 2010; 31(2): 133-141

2 Abdulsalam M, Ibrahim A, Michael G, et al. Hand washing practicesand techniques among health professionals in a tertiary hospital in Kano. J Med Investig Pract 2015; 10(1): 8-12

**Disclosure of Interest**: None declared

### P156 INVESTIGATING THE EFFECT OF SHORT DAILY HAND HYGIENE PRACTICE ON ACHIEVING PROFICIENCY: A PROSPECTIVE COHORT STUDY

#### L. Price, L. Gozdzielewska, K. McAloney

##### School of Health & Life Sciences/Institute for Applied Health Research, GLASGOW CALEDONIAN UNIVERSITY, Glasgow, United Kingdom

###### **Correspondence:** L. Price

**Introduction:** Hand hygiene training aims to develop participants’ proficiency in performing the World Health Organization technique without prompts. In this study we explored the impact of short daily hand hygiene practice with feedback (deliberate practice) in achieving proficiency.

**Objectives:**

**Methods:** Staff and students in a university volunteered to practice hand hygiene using the SureWash® app on their phone or tablet on a daily basis for four weeks. App data provided information on the frequency of practice and the achieved level of performance. In addition, once a week participants were observed, by the researchers, performing hand hygiene using the SureWash® ELITE system to assess their level of performance.

The assessment at which proficiency was achieved was translated into the number of training sessions required, and investigated in relation to data gathered on the participants’ gender, age group, previous training experiences, manual dexterity, and adherence with the protocol, using T-tests.

**Results:** Data from 47 participants demonstrated that 38 (81%) achieved proficiency. The mean number of sessions to achieve proficiency was 24.3 (sd = 17.8). Daily practice decreased from 28% (N = 13) in week 1 to 15% (N = 7) in week 4. There was no significant difference in age, previous training or manual dexterity but males required fewer training sessions to achieve proficiency (15.3 v 27.83, t (35.92_)_ = -2.914, p = .006). The analysis was power calculated for moderate effects.

**Conclusion:** Our results suggest that the use of short daily hand hygiene practice shows promise in promoting proficiency. They challenge the current culture of single training sessions repeated annually or biannually as being sufficient to achieve proficiency. We recommended that infection prevention and control teams consider the use of deliberate practice for use in hand hygiene training along with concurrent longitudinal evaluation of its effectiveness in comparison to current practice.

**Disclosure of Interest**: None declared

### P157 ACCEPTABILITY AND TOLERABILITY OF ALCOHOL-BASED HAND HYGIENE PRODUCTS FOR ELDERLY RESIDENTS IN LONG-TERM CARE; A CROSSOVER STUDY

#### M. O’Donoghue^1^, J. M. Ho^1^, D. Pittet^2^, L. K. Suen^1^

##### ^1^Squina International Centre for Infection Control, School of Nursing, The Hong Kong Polytechnic University, Hong Kong, Hong Kong; ^2^Infection Control Program and WHO Collaborating Centre on Patient Safety, University of Geneva Hospitals, Geneva, Switzerland

###### **Correspondence:** M. O’Donoghue

**Introduction:** Efforts to improve hand hygiene in healthcare settings have largely targeted healthcare worker (HCW) compliance but its importance for patients, including those in long-term care facilities (LTCFs), is receiving increased attention. Alcohol-based hand rub (ABHR) can lead to improved compliance.

**Objectives:** To determine acceptability and tolerability of two ABHRs for hand hygiene of elderly LTCF residents using a modified version of the WHO protocol.

**Methods:** 36 elderly LTCF residents participated in this crossover study. A modified and translated (Chinese) version of the WHO protocol for evaluation of two or more ABHRs was used to evaluate product acceptability and tolerability for one gel (bottle with reclosable cap) and one foam (pump). During each 3-day testing period, participants received their own portable bottle of ABHR. A research nurse objectively assessed hand skin integrity at baseline and throughout the study. Skin moisture content was determined using a Moisture Checker (STR, Ca, USA). Participants rated ABHR tolerability and acceptability using the WHO checklist at the end of each test period.

**Results:** Both products passed the WHO criteria for acceptability and tolerability. The foam (86%) scored higher than the gel (51%) for ease of use possibly because some participants found the cap of the gel bottle difficult to open due to finger stiffness. No evidence of damage to skin integrity was observed. Overall, skin moisture content improved by the end of the study. Residents preferred either test product to the ABHR rinse currently in use by the LTCF.

**Conclusion:** Elderly LTCF residents were willing to use ABHR for hand hygiene. Both ABHRs were well tolerated and preferred over the usual product. Forgetfulness and difficulties in hand rubbing due to finger stiffness was a barrier for some residents. HCW-assisted hand hygiene at specified times daily and reminders to perform hand hygiene could offer a feasible and sustainable strategy to overcome these challenges.

**Disclosure of Interest**: None declared

## Poster session: Behaviour

### P158 IMPROVING WASTE MANAGEMENT PRACTICES AMONG HEALTH CARE WORKERS IN A LARGE PUBLIC HOSPITAL IN UGANDA.

#### M. S. Kesande^1^, W. Omuut^1^, J. Agwang^2^, J. Nanyondo^1^, M. Lamorde^1^

##### ^1^Global Health Security Project, Infectious Diseases Institute, Makerere University, Kampala, Kampala; ^2^IPC focal person, Jinja Regional Referral Hospital, Jinja, Uganda

###### **Correspondence:** M. S. Kesande

**Introduction:** Waste management is one of the IPC standards with poorest performance in developing countries; this can be attributed to deficiencies in supplies, poor attitude of health workers and limited involvement of facility leadership. The Infectious Diseases Institute (IDI) Global Health Security project designed a model to improve waste management at Jinja Regional Referral Hospital.

**Objectives:** To demonstrate a model to improve waste management practices among health workers.

**Methods:** In a bid to improve Infection Prevention and Control (IPC) in health facilities, IPC committees have been formulated to lead the process. IDI trains these committees on standard precautions, roles and responsibilities of committee members and institutes routine waste quantification and feedback exercises in regional referral hospitals. Waste handlers are also trained on use of Personal Protective Equipment (PPE), waste collection, transportation and disposal.

Engaging IPC committees as drivers of the process; the committee members have trained 170 fellow health workers and 41waste handlers on waste management components. Waste quantification and feedback exercises have been instituted as well as involvement of the hospital leadership in planning meetings of the waste management subcommittee.

**Results:** More CMEs targeting waste management components have been conducted quarterly. Improved waste segregation demonstrated by increase in percentage of non-infectious waste from 0% in July to 69% in September 2018. This demonstrated less mixing of waste though the facility has not yet reached the expected standard of 85% for non-infectious waste. The training and routine CMEs have instigated improved use of Personal Protective Equipment (PPE) and proper waste disposal among waste handlers.

**Conclusion:** Use of IPC committees should be utilized as change agents to improve compliance to waste management practices. Improved practices will help to leverage cost savings that can be redirected for further improvements.

**Disclosure of Interest**: None declared

### P159 DEVELOPING A PROTOCOL TO DEAL WITH DISINFORMATION AND FAKE NEWS IN INFECTION PREVENTION AND CONTROL

#### A. Peters^1^, R. Martischang^1^, C. Guitart^1^, N. Lotfinejad^2^, D. Pittet^1^

##### ^1^University Hospitals of Geneva, Geneva, Switzerland, ^2^Mashhad University of Medical Sciences, Mashad, Iran, Islamic Republic Of

###### **Correspondence:** A. Peters

**Introduction:** Although misinformation has always existed, the scope and speed at which fake news can reach even the most remote corners of the globe is a modern phenomenon. In the field of infection prevention and control (IPC), we like to believe that our dedication to the field and the process of conducting science protects us from the ravages of 'bad buzz' and fake news. This misconception leads medical professionals to underestimate the negative effects of misinformation on public health.

**Objectives:** To create a protocol for evaluating and categorizing misinformation.

**Methods:** We conducted a literature search of the role of fake news in general and in IPC, and of ways that is being addressed by governments, the media, and the scientific community. We then thoroughly analyse different instances of bad buzz in IPC that affected us directly, and began developing a standardized protocol of systematically evaluating analysing, reporting, and reacting to misinformation.

**Results:** The resulting protocol has two parts, one that helps readers analyse all types of media around a certain piece of misinformation and includes steps to analyse the hypotheses put forward, assertions made, implied associations, causality, clinical relevance, etc. in a systematic way. The second part is a reporting template that would help homogenize the resulting analyses in a form that could be compiled and organized easily, even if written by different people. Multiple examples will be given, ranging from misinformation to intentional fake news, and their analysis presented.

**Conclusion:** We would like to validate this protocol and make it an implementable tool for IPC researchers to use when trying to combat the detrimental effects of bad buzz and fake news. In the future, it would be desirable to create an online repository for this information, where IPC researchers from all over the world could upload their work and have it be organized by subject. We believe that this would greatly strengthen the response to disinformation and help to reduce the potential damage to patients.

**Disclosure of Interest**: None declared

### P160 COMPARATIVE ASSESSMENT OF INFECTION PREVENTION AND CONTROL PRACTICE AMONG MATERNITY UNIT HEALTH WORKERS IN PUBLIC AND PRIVATE SECONDARY HEALTH FACILITIES IN KADUNA STATE, NIGERIA

#### J. Sunday^1^, M. B. Sufiyan^2^, C. L. Ejembi^2^, B. N. Natie^1^, A. A. Olorukooba^2^, C. J.-C. Igboanusi^3^, M. Onoja-Alexander^4^, Y. Musa^2^, E. E. Ajumuka^2^

##### ^1^Department of Planning Research and Statistics, Ministry of Health and Human Services, Kaduna State; ^2^Department of Community Medicine, College of Health Sciences, Ahmadu Bello University, Zaria; ^3^Department of Public Health, Headquarters, 2 Division Medical Services/Hospital, 2 Division, Nigerian Army, Adekunle Fajuyi Cantonment, Ibadan; ^4^Department of Community Medicine, College of Health Sciences,Kogi State University, Anyigba, Lokoja, Nigeria

###### **Correspondence:** M. Onoja-Alexander

**Introduction:** Infection Prevention and Control (IPC) practice in health facilities is abysmally low in developing countries, resulting in significant preventable morbidity and mortality.

**Objectives:** This study assessed and compared health workers’ practice of IPC strategies in public and private secondary health facilities in Kaduna State.

**Methods:** An analytic cross-sectional comparative study was employed. Multistage sampling technique was used to select 227 participants each comprising of doctors, midwives and nurses drawn from public and private health facilities. Data was collected using interviewer-administered questionnaire and observation checklist. Data was analysed using both bivariate and multivariate analysis. Statistical significance was determined at p value < 0.05.

**Results:** The practice of infection prevention is poor. Overall, 48% of the HW used plain soap to wash their hands, and 35.1% of them used hand operated faucet to turn water on and off during hand washing. These practices were significantly poorer in public health facilities (40.1% and 30.0% respectively) compared to private health facilities (55.9% and 40.5% respectively) (p < 0.001 and P<0002 respectively). Overall, 42.3% of the health workers did not change their gowns in-between seeing patients, with the significantly higher rates in 73.1% of private compared to 42.3% of public health facility workers (p < 0.001). Additionally, 30.5% and 10.1% of health workers do not use face mask and eye goggle respectively when conducting procedures likely to generate splash of body fluids, however, there was no significant difference in these poor practices in public compared to private health facilities. The mean IPC practice was 51.6%±12.5, this was significantly lower among public (48.8%±12.5) compared to private (54.5%±11.9) health facility workers (p value <0.0001). Private HF workers were three times more likely to implement IPC interventions compared to public health facility workers.

**Conclusion:** IPC practice especially among public health facility workers was poor.

**Disclosure of Interest**: None declared

### P161 RAISING AWARENESS ABOUT HAND WASHING WHILE CONTACTING WITH NEWBORN PATIENT - WHAT APPROACH IS BETTER FOR MEDICAL STAFF COMING OUTSIDE NICU?

#### O. Turcanu, M. Guzun

##### Neonatal Reanimation Unit no.1, Clinical Municipal Hospital no.1 / Perinatal Center, Chisinau, Moldova, Republic of

###### **Correspondence:** O. Turcanu

**Introduction:** Besides the NICU staff, who is trained/monitored tightly on hand hygiene, babies in our unit are contacting with general "visiting" staff: - laboratory workers (taking WBC & blood gases), USG specialists (same as for adult ICU), engineers (contacting with the circuits of ventilators).

Starting with 14.01.2018 we have a registry of nosocomial culture results. Our spectrum (St.epidermidis) of nosocomial bacterias is different than adult ICU (P.aeruginosa & A.baumanii), but most difficult cases, dangerous to neonatal ICU were with last 2 bacterias.

**Objectives:** Bimensual cultures from surfaces, staff hands, equipement etc. didn't show that the source of Pseudomonas & Acinetobacter is in our unit. Anonimous observations showed insufficient compliance with hand washing procedures by "visiting" staff. So, we tested a few interventions - what will better motivate them to change this risky behavior.

**Methods:** a) Periodic evaluation meetings, usually relying on professional reprimands (still a tactique in former soviet countries).

b) Using a "paper requests" with inserted nice reminder about hand washing at the end (for every "visiting" staff in unit.

c) 15th October action "a flower for clean hands".

**Results:** *Qualitative measurement by provider:* - Colleagues from "visiting" scaled the interventions they considered more willing to be subjected - the best was "paper requests" & the action "a flower for clean hands", and worst - unpleasant meetings.

*Observational results:* - The fastest improvement both in remembering when to wash hands while contacting with baby/equipment and hand washing technique - middle level personnel (65% of lab workers) & technical personel (70% engineers) and slowest (35%) among physicians.

*Indirect results:* - Blood/traheal aspirates - since 10.11.2018 we don't have any +culture of P.Aeruginosa or A.baumanii (no proved cases of their transmission from other hospital units), all cases of bacterial growth being St.epidermidis (main nosocomial bacteria of our unit).

**Conclusion:** Although visual information on hand washing (ex. WHO posters) is useful in promoting hand hygiene; when speaking about targeting medical staff from different hospital settings - a more personal approach in raising awareness is likely to have a better outcome & efficient feedback. Especially if this is done in a user-friendly way (not punishment tactic)

**Disclosure of Interest**: None declared

### P162 CONSTRUCTION AND CONTENT VALIDATION OF A QUESTIONNAIRE TO EVALUATE THE NURSES’ KNOWLEDGE, ATTITUDES AND PRACTICES TOWARDS ORAL CARE OF CRITICALLY PATIENTS

#### I. D. T. A. Amaral, A. Felix, B. R. Paz, J. A. Silva, B. R. Santos, M. S. Coyado, L. A. Gomes

##### ^1^Enfermagem, Faculdade de Ciências Médicas da Santa Casa de São Paulo, São Paulo, Brazil

###### **Correspondence:** A. Felix

**Introduction:** Oral care of critically patients is a standard part of the daily nursing care and is one of the strategies of lowering the incidence of ventilator-associated pneumonia (VAP). In Brazil, there is no tool that measure the nurses’ knowledge, attitudes and practices towards oral care of critically ill patients.

**Objectives:** To describe the process of elaborating and validating a questionnaire about the nurses’ knowledge, attitudes and practices towards oral care of critically ill patients.

**Methods:** Methodological study, performed from May 2018 to December 2018. The content of items that composed the questionnaire was based on present evidence-based recommendations for preventing VAP. The questionnaire was submitted to the content validation of eight nurses specialized in Infection Control and four nurses specialized in Intensive Care who gave their opinions on clarity, pertinence and relevance of the items, in two rounds of the Delphi Technique. Content Validity Index (CVI) was used to verify consistency and items with CVI ≥ 0.80 were considered valid.

**Results:** The questionnaire included 29 items (Knowlede=13 items; Attitudes and Practices=15 items) and was considered relevant, clear and pertinent by the specialists, with an average agreement of 0.94, 0.90 and 0.92 respectively. The overall CVI was 0.92, evidencing a satisfactory level of agreement among the specialists.

**Conclusion:** The questionnaire was considered valid by the specialists; thus, it can be implemented to evaluate the nurse’s knowledge, attitudes and practices towards oral care of critically ill patients.

**Disclosure of Interest**: None declared

### P163 PLANNING IS DISTINCT FROM MOTIVATION AND CAPABILITIES IN EXPLAINING SURGICAL SITE INFECTION-PREVENTIVE COMPLIANCE: RESULTS FROM A SINGLE-CENTRE, SMALL SAMPLE BUT HIGH RESPONSE SURVEY OF ORTHOPAEDIC PHYSICIANS IN HANNOVER, GERMANY

#### T. von Lengerke^1^, I. Tomsic^1^, F. Gossé^2^, E. Ebadi^3^, I. Hartlep^4^, P. Schipper^4^, B. Schock^4^, I. F. Chaberny^4^

##### ^1^Department of Medical Psychology, Hannover Medical School, Centre of Public Health and Healthcare; ^2^Department of Spinal Surgery and Conservative Orthopaedics, Clinic of Orthopaedics of Hannover Medical School at DIAKOVERE Annastift, Hannover; ^3^Institute of Medical Microbiology and Hospital Epidemiology, Hannover Medical School, Centre for Laboratory Medicine; ^4^Institute of Hygiene, Hospital Hygiene and Environmental Medicine, Leipzig University Hospital, Leipzig, Germany

###### **Correspondence:** I. Tomsic

**Introduction:** Preventing surgical site infections (SSI) implies compliance with many pre-, intra- and postoperative measures [1]. Evidence is lacking on psychological determinants of comprehensive compliance, i.e. with as many measures as possible. Among others, orthopaedic surgery physicians represent an underresearched group.

**Objectives:** To identify associations between self-reported SSI-preventive compliance and determinants as specified by the COM-B- (capability, opportunities, motivation and behaviour-) model [2].

**Methods:** In a pretest to the "WACH"-trial (funding: German Federal Ministry of Health, grant-ID: ANNIE2016-55-038; German Clinical Trials Register-ID: DRKS00015502), N=52 orthopaedic surgeons and anaesthetists of an orthopaedic clinic participated in a questionnaire survey. The response rate was 73.2%. Compliance regarding 26 SSI-preventive measures respondents felt responsible for was assessed by self-reports as well as COM-determinants (18 items). Statistically, factor analysis and linear regression models were used.

**Results:** Using oblimin rotation on the COM-items, 4 factors emerged: capabilities (4 items/Cronbachs alpha=0.88), motivation (4/0.83), opportunities (3/0.90), and planning (2/0.82). In the final backward linear regression model of self-reported compliance rate across all 26 measures (mean: 89.8%), the number of measures responsible for had a negative regression weight (beta=-0.31/p=0.04), while capabilities (0.35/0.02) and planning (0.26/0.09) showed positive effects.

**Conclusion:** Action and coping planning of SSI-preventive measures may be distinct from motivation and capabilities as a construct and determinant of self-reported compliance. Like in the realm of hand hygiene interventions [3], promotion of SSI-prevention should stress planning skills and social support.

**References**

[1] WHO. Global Guidelines for the Prevention of Surgical Site Infection. Geneva: WHO; 2018[2] Michie et al. The Behaviour Change Wheel. Implement Sci 2011;6:42[3] von Lengerke et al. Impact of Psychologically Tailored Hand Hygiene Interventions on Nosocomial Infections with Multidrug-Resistant Organisms. Antimicrob Resist Infect Control 2019;8:56

**Disclosure of Interest**: None declared

### P164 IMPLEMENTATION INTERVENTIONS TO IMPROVE COMPLIANCE WITH SURGICAL SITE INFECTION PREVENTIVE MEASURES IN ABDOMINAL SURGERY: A SYSTEMATIC REVIEW

#### I. Tomsic^1^, N. R. Heinze^2^, I. F. Chaberny^3^, C. Krauth^2^, B. Schock^3^, T. von Lengerke^1^

##### ^1^Department of Medical Psychology; ^2^Institute of Epidemiology, Social Medicine and Health Systems Research, Hannover Medical School, Centre of Public Health and Healthcare, Hannover; ^3^Institute of Hygiene, Hospital Hygiene and Environmental Medicine, Leipzig University Hospital, Leipzig, Germany

###### **Correspondence:** I. Tomsic

**Introduction:** Despite several guidelines that recommend measures to prevent surgical site infections (SSIs) [1], there is a high prevalence in abdominal surgery [2]. Also, compliance with these measures is often insufficient [3]. At the same time, there exist several implementation interventions to promote compliance with preventive measures [4].

**Objectives:** The aim of this systematic review is to identify implementation interventions that are used in the field of abdominal surgery to prevent SSIs, and determine associations between implementation interventions and reductions in SSI-rates.

**Methods:** Literature was searched in April 2018. Implementation interventions were classified using the Cochrane Review Group Effective Practice and Organisation of Care’s “EPOC Taxonomy” [3]. In addition, an effectiveness analysis was conducted on the association between number of implementation strategies and reduction of SSI-rates.

**Results:** Forty studies were included. Implementation strategies used most frequently were audit and feedback (80% of the studies), organisational culture (70%), monitoring the performance of healthcare delivery (65%), reminders (53%), and educational meetings (45%). Twenty-nine studies used a multimodal strategy with ≥3 implementation interventions. There was a tendency for strategies with 3-5 strategies to be most effective in reducing SSI rates, however starting from a comparably high baseline level.

**Conclusion:** In abdominal surgery, mostly standard multimodal implementation strategies are applied to improve compliance with SSI preventive measures. Further research is needed on which implementation strategies, or bundles of these, are most effective in promoting compliance in abdominal surgery.

**References**

1. World Health Organization (WHO). Global Guidelines for the Prevention of Surgical Site Infection, 2nd ed. Geneva:WHO;2018.

2. Azoury et al. Postoperative abdominal wound infection - epidemiology, risk factors, identification, and management. Chronic Wound Care Manage Res 2015;2:137-148.

3. Leaper et al. Surgical site infection: poor compliance with guidelines and care bundles. Int Wound J 2015;12:357-362.

4. Effective Practice and Organisation of Care (EPOC). EPOC Taxonomy;2015. (https://epoc.cochrane.org/epoc-taxonomy).

**Disclosure of Interest**: None declared

### P165 GAMIFICATION – AN INNOVATIVE INTERVENTION TO INCREASE HAND-HYGIENE COMPLIANCE

#### G. Regev-Yochay^1^, O. Berezovsky^1^, I. Tal^1^, E. Konan^2^, O. Barnet^3^, E. zimlichman^4^

##### ^1^Infection Prevention & Control; ^2^Radiology department, Sheba Medical Center, Ramat Gan; ^3^Entropy, Tel Aviv; ^4^Sheba Medical Center, Ramat Gan, Israel

###### **Correspondence:** G. Regev-Yochay

**Introduction:** Full adherence to infection control measures is frequently difficult to achieve. The Radiology department, which is a central intersection, with large patient movement and difficult to measure infection outcomes, is a particular challenge. Gamification and positive psychology have been previously successfully used to modify organizational culture in other settings.

**Objectives:** To engage the Radiology team to IC culture.

**Methods:** The Radiology department team of the Sheba Medical Center includes 120 technicians, 60 physicians and 12 nurses. Hand hygiene compliance was low (70%) despite routine IC measures (observation & feedback, annual training, etc) before the intervention. A positive-feedback game was carried out during 4 months, focusing on hand-hygiene compliance, adherence to cleaning and isolation policy guidelines.

Intervention-agents were recruited from different HCW sectors of the department. They received a short IC creative training (2h) using quizzes and games. During 3 months, they gave positive feedback and marked HCW who they observed adhering to IC measures. From the second month of the game, patients waiting for a radiologic procedure, were also recruited to mark HCW whom they observed performing hand hygiene. Weekly honours & prizes where distributed by the department director to HCWs with highest marks.

**Results:** Rapidly all HCW in the department were engaged to the game. Hand hygiene compliance increased to 90% in all the different units of the Radiology Departent. A 'whatsup' group was established within the game and positive feedbacks with pictures and slogans were posted.

Currently, one year after the formal game has ended, the hand hygiene compliance is still >90% in all department units and pictures & slogans are continuously posted.

**Conclusion:** Engaging teams by positive feedback and gamification is an efficient methodology to achieve IC adherence, particularly in challenging circumstances.

**Disclosure of Interest**: G. Regev-Yochay: None declared, O. Berezovsky: None declared, I. Tal: None declared, E. Konan: None declared, O. Barnet Shareholder of: Entropy group, E. zimlichman: None declared

### P166 THE “PATIENT ZONE” AND ITS CONCEPTUAL HURDLES FOR HEALTHCARE PROVIDERS – A QUALITATIVE CONTENT ANALYSIS

#### J. Bogdanovic^1,2^, S. Petralito^1^, S. Passerini^1^, H. Sax^1^, T. Manser^3^, L. Clack^1^

##### ^1^Department of Infectious Diseases and Hospital Epidemiology, University Hospital Zurich, Zurich, ^2^Institute of Nursing Science, University Basel, Basel, ^3^School of Applied Psychology, University of Applied Sciences and Arts Northwestern, Olten, Switzerland

###### **Correspondence:** S. Petralito

**Introduction:** The “patient zone” concept was introduced in 2007 to guide infection prevention and control (IPC) efforts and to anchor indications for hand hygiene.

**Objectives:** The present study aims to understand how the patient zone is interpreted and applied by healthcare providers (HCP).

**Methods:** Ten nurses and physicians allocated 30 items from the healthcare environment to “inside” or “outside” the patient zone with simultaneous verbal think-aloud. Allocations were subsequently compared with those of two IPC-experts. Utterances from the think-aloud process were included in a content analysis to understand HCPs’ interpretations of the patient zone.

**Results:** A total of 68% of item allocations were consistent with expert consensus and seven out of thirty items achieved a 100% agreement among participants. Our content analysis revealed ambiguity and variation in participants’ interpretations of the patient zone and its practical implications***.*** Participants often interpreted that items with direct patient contact should be allocated to “inside” the patient zone or they interpreted the zone as a physical perimeter, where all objects inside, i.e. a patient room, were allocated to “inside” the patient zone. Furthermore, participants often deduced allocation based on the observed need to disinfect hands and objects. Furthermore, participants interpreted that an item’s allocation could change depending on the context of the item’s use.

**Conclusion:** Variation in HCPs’ interpretations of the patient zone led to low agreement with expert consensus and discrepancies among participants. HCPs’ interpretations were based on simplifying concepts, such as equating the patient zone to a physical perimeter or to items with patient contact, and thus failed to accurately capture the concept’s subtleties. Our results provide first insights into how the patient zone is interpreted and applied by HCPs. Our findings suggest that a revised concept and didactic strategies may be promising approaches to improve understanding and application of the patient zone.

**Disclosure of Interest**: None declared

### P167 KNOWLEDGE AND SELF-REPORTED COMPETENCE IN STANDARD PRECAUTIONS (SP): A SURVEY AMONG HEALTHCARE WORKERS (HCW) IN A MULTI-SITE HOSPITAL

#### D. Vuichard-Gysin^1^, R. Fulchini^1^, P. Kohler^2^, M. Schlegel^2^

##### ^1^Infectious Diseases & Hospital Epidemiology, Thurgau Hospital Group, Muensterlingen; ^2^Infectious Diseases & Hospital Epidemiology, Cantonal Hospital St. Gallen, St. Gallen, Switzerland

###### **Correspondence:** D. Vuichard-Gysin

**Introduction:** SP are fundamental to reduce the risk of transmission of bloodborne and other pathogens from identifiable and unrecognized sources. The Thurgau hospital group comprises two acute-care hospitals, a rehabilitation and a psychiatry clinic. The concepts of SP are outlined in the local infection control guidelines but levels of knowledge and competence in implementation remain largely unknown.

**Objectives:** To increase awareness on the importance of SP and to identify knowledge gaps, a questionnaire was addressed to HCW involved in patient care.

**Methods:** A 15-item survey with single and multiple-choice questions was distributed by e-mail. Two reminders were sent out to enhance participation. Depending on the importance of the respective question, one to three points were awarded for each correct answer (3 points = highly important). HCW were also asked to rate their overall competence in SP on a scale from 1 to 4 (1 = feeling overburdened, 4 = competent). After completion, the correct answers along with the total score (max. 35 points) and a feedback about the overall performance were automatically displayed.

**Results:** From July to August 2018, a total of 898 HCW (37% response rate) participated in the survey. Of these, 63% were nurses, 18% were doctors and 19% were others. Of 754 participants answering the knowledge questions, 355 (47%) had good knowledge (> 26 points). The following proportions of HCW correctly identified these measures as component of SP: hand hygiene (98%), wearing of personal protective equipment (78%), cough etiquette (64%) and vaccinations (23%). Only 46% considered SP indicated in patients under transmission-based precautions. Of 177 respondents in the lowest total score category (<23 points), 154 (89%) judged themselves as (rather) competent.

**Conclusion:** Knowledge of SP among HCW was moderate and revealed important gaps in the understanding of the basic concepts of SP. The discrepancy between knowledge scores and self-reported competence suggests that HCW overestimate themselves in their competence. These results serve as basis for planning multi-faceted educational interventions.

**Disclosure of Interest**: None declared

### P168 INPUT FROM IMPLEMENTATION SCIENCE: REVISION OF THE WORLD HEALTH ORGANIZATION MULTIMODAL HAND HYGIENE IMPROVEMENT STRATEGY IN LIGHT OF THE BEHAVIOUR CHANGE WHEEL

#### P. Muthukumaran ^1^, A. Peters^2^, C. Guitart^2^, D. Pittet^2^

##### ^1^University of Geneva; ^2^University Hospitals of Geneva, Geneva, Switzerland

###### **Correspondence:** P. Muthukumaran

**Introduction:** Hand hygiene (HH) remains the most important measure to prevent healthcare associated infections (HAI). The World Health Organization (WHO) Multimodal Hand Hygiene Improvement Strategy (MMHHIS) is currently the most effective documented method for implementing HH practices in hospital settings worldwide. While this model has had great success in clinical settings, other models and tools available from the field of implementation science, such as the Behaviour Change Wheel (BCW), incorporate some potentially useful elements that are not explored in the MMHHIS.

**Objectives:** The objective of this research is to analyse and compare the MMHHIS and the BCW, and identify elements from the latter that might be used to improve the MMHHIS.

**Methods:** A literature review was performed using Google Scholar in order to explore behaviour change frameworks applicable to healthcare settings. The BCW was chosen because it is the most comprehensive of the models available (built using 19 other frameworks), and has already been applied in healthcare settings. The review was followed by a qualitative analysis of the suitability of the BCW to help refine the MMHHIS.

**Results:** Elements from the BCW can be incorporated into the MMHHIS and may result in a more comprehensive approach to designing interventions for improving HH compliance. The MMHHIS focuses on healthcare workers (HCW) in a hospital setting, doesn’t comprise elements of early stages of HCWs’ education, or emphasis on the importance of role-models, which are addressed in the BCW. The BCW also offers a more *visually* comprehensible model that is easily adaptable to suit each setting, facilitating the adoption of the framework.

**Conclusion:** The BCW offers a new lens through which to review the WHO MMHHIS; it uncovers new avenues to potentially improve HH practices. Thus, the current research suggests that an updated multimodal strategy should be developed by incorporating elements of the BCW. This model should then be tested in a clinical setting in order to compare it with the existing strategy.

**Disclosure of Interest**: None declared

### P169 Withdrawn

### P170 ACCEPTABILITY OF WHO HAND HYGIENE REMINDERS IN MATERNITY SETTINGS; PRELIMINARY RESULTS OF A MIXED METHODS STUDY

#### C. L. Dunlop^1^, C. Kilpatrick^2^, M. Bonet^3^, V. Brizuela^3^, W. Graham^4^, A. Thompson^5^, L. Jones^6^, D. Lissauer^1^

##### ^1^Institute of Metabolism and Systems Research, University of Birmingham, Birmingham; ^2^Consultant, The Soapbox Collaborative, London, United Kingdom; ^3^UNDP/UNFPA/UNICEF/WHO/World Bank Special Programme of Research, Development and Research Training in Human Reproduction (HRP), Department of Reproductive Health and Research, World Health Organisation, Geneva, Switzerland; ^4^The Soapbox Collaborative, London; ^5^College of Medical and Dental Sciences; ^6^Institute of Applied Health Research, University of Birmingham, Birmingham, United Kingdom

###### **Correspondence:** C. L. Dunlop

**Introduction:** Hand hygiene is fundamental to prevent healthcare associated infections. Currently there are no World Health Organisation (WHO) hand hygiene reminders specific to maternity settings. Maternity settings may benefit from these due to: WHO recommendations that reminders be part of a multimodal improvement strategy; clinical circumstances unique to pregnancy and childbirth; and to support prevention of maternal sepsis.

**Objectives:** We sought to investigate if healthcare workers find the current WHO hand hygiene reminders acceptable for use in maternity settings and if adaptations could improve their relevance.

**Methods:** An integrative mixed-methods approach was used to assess acceptability of WHO hand hygiene reminders in maternity settings. Informed by a valid acceptability domains framework, a multi-language survey was launched, sampling participants via a WHO multi-country study network and snowballing. Purposively sampled qualitative interviews and a focus group were conducted with maternal health clinicians and public health practitioners with experience working in high and low resource settings.

**Results:** The survey (n=342), interview and focus group (n=19) responses represented 48 countries. Preliminary results demonstrate that acceptability of hand hygiene reminders was high, with 76% scoring ‘agree’ or ‘strongly agree’ across pooled domains. Campaign posters from World Sepsis Day 2018 were deemed equally or more acceptable for maternity settings than the original reminders, across all domains. Acceptability could be improved by including: images that represent maternity settings; clear explanations of the importance of hand hygiene; and infographic examples of the 5 moments of hand hygiene in this setting.

**Conclusion:** Preliminary results demonstrate that the WHO hand hygiene reminders were well received by healthcare workers, but acceptability for maternity settings could be improved with adaptions. Work is underway to address this, in a reminder specific to maternity care that would be acceptable in high and low resource settings.

**Disclosure of Interest**: None declared

### P171 BUILD IT, TEACH IT, CHECK IT, SELL IT, LIVE IT – A CONVERSATION ABOUT WRITING YOUR PLAYBOOK FOR IPC IMPROVEMENT

#### J. Storr^1^, C. Kilpatrick^2^

##### ^1^Global Health, S3 Global, MORETON IN MARSH; ^2^S3 Global, Glasgow, United Kingdom

###### **Correspondence:** J. Storr

**Introduction:** Implementation of infection prevention and control (IPC) evidence is challenging and complex. The global multimodal improvement approach that is now a cornerstone of World Health Organization (WHO) implementation documents aims to overcome these challenges and to support and empower end users to take a systematic approach, applicable to their settings. With the goal of improving outcomes and behaviours, it is one of the evidence-based IPC core components.

**Objectives:** To present a synthesis of intelligence and practical learnings on the application of a multimodal improvement strategy (MMIS) in a range of countries. To outline how understanding of the MMIS can be enhanced by the use of a novel ‘mantra’.To stimulate reflection and discussion on the current and future utility of the MMIS, using country and healthcare facility examples.

**Methods:** A review of published IPC literature and resources was undertaken. Personal interactions, including peer-to-peer feedback and social media engagement, were also used to determine perceptions of the current understanding and use of all five elements of the MMIS.

**Results:** The MMIS can be summarised as follows; system change (build it), training and education (teach it), monitoring and feedback (check it), reminders and communication (sell it), institutional safety climate and culture change (live it). The current situation suggests that its elements are used but that comprehensive and widespread understanding and acceptance of the approach in its entirety and the ability to clearly articulate its value remains sub-optimal. At the global level, the approach has already been applied to hand hygiene, IPC generally, surgical site infection surveillance and carbapenem-resistantorganism control.

**Conclusion:** The MMIS is an IPC evidence-based recommendation. The approach still needs to be promoted for full adoption in all countries, in all health care settings and adaption of global documents into local ‘playbooks’ is needed.

**Disclosure of Interest**: None declared

### P172 SUCCESSFUL APPLICATION OF NATIONAL PATIENT SAFETY INITIATIVE PROGRAM STRATEGY TO REDUCE HEALTHCARE ASSOCIATED INFECTIONS IN KING FAISAL MEDICAL COMPLEX-TAIF, KINGDOM OF SAUDI ARABIA

#### W. A. Mazi^1^, M. H. Abdulwahab^1^, M. A. Al-Ashqar^1^, N. A. Bouafia^1^, N. A. Hamoda^1^, Y. S. Aldecoa^1^, Z. R. Bhat^1^, S. H. Al Wagdani^1^, O. S. Yasin^2^, S. A. Al Sayali^2^, W. S. Ahmed^3^, A. A. Al Talhi^4^, S. Thomas^4^, A. M. Dahlawi^5^, S. A.-D. M. Mahfouz^6^, M. R. Al Yami^7^

##### ^1^Infection Prevention and Control; ^2^Intensive Care Unit; ^3^Gynecology Department; ^4^Operation Room Department; ^5^Maternity Medical Director; ^6^Surgey Department; ^7^Hospital Director, King Faisal Medical Complex, Taif, Saudi Arabia

###### **Correspondence:** W. A. Mazi

**Introduction:** National Patient Safety Initiative Program 2018 was introduced in King Faisal Medical Complex-Taif as a part of national transition program 2020 for achievement the national vision 2030.

**Objectives:** To reduce HAIs benchmarking to the National Healthcare Safety Network (NHSN), USA.

**Methods:** A prospective study was conducted in the 27-bed intensive care unit and maternity tower of King Faisal Medical Complex (KFMC)-Taif, Kingdom of Saudi Arabia. Central line-associated bloodstream infection (CLABSI), catheter-associated urinary tract infection (CAUTI), ventilated associated pneumonia (VAP) and CS-SSI were identified using the Centers for Disease Control and Prevention and National Healthcare Safety Network (NHSN, USA) criteria. Incidence rate, utilization ratio, benchmarking, and statistical analysis were carried out using the NHSN recommendations. NPSIP was introduced in January 2018 using three steps strategy; 1) identify indicator based on NHSN benchmark, 2) assess risk factors using Society Healthcare Epidemiology of America/Infectious Diseases society of America (SHEA/IDSA) basic practice recommendations, and 3) perform gap analysis strategy for intervention.

**Results:** CLABSI incidence rate is between 50th-75th percentile benchmarking to NHSN despite of higher in utilization ratio. CAUTI incidence rate is between 25th-50th percentile benchmarking to NHSN with lower in utilization ratio . VAP incidence rate was declined from 5.0- to 3.19/1000 ventilated days but still above 90th percentile with same in utilization ratio 0.28.

The CS-SSI incidence rate is lower than the NHSN using re-admission surveillance method.

**Conclusion:** KFMC-Taif successfully achieving the NHSN, USA benchmark using the three strategy method.

**References**

Not applicable.

**Disclosure of Interest**: None declared

## Poster session: Surveillance 1

### P173 SIX YEARS OF POINT PREVALENCE SURVEY OF HEALTHCARE-ASSOCIATED INFECTIONS AND ANTIMICROBIAL USE AT A TERTIARY ACUTE-CARE UNIVERSITY HOSPITAL IN NORTH-WEST ITALY

#### F. Tassinari^1^, M. Zacconi^1^, I. Barberis^1^, M. Astengo^1^, V. Tisa^1^, S. Schenone^1^, D. de Florentiis^2^, V. Daturi^2^, R. Ziferro^2^, A. M. Di Bella^2^, A. Talamini^2^, M. K. Mikulska^1^, C. Viscoli^1^, G. Icardi^1^

##### ^1^University of Genoa; ^2^Ospedale Policlinico San Martino, Genova, Italy

###### **Correspondence:** F. Tassinari

**Introduction:** Healthcare-associated infections (HAI) point prevalence surveys (PPS), repeated periodically, provide an effective epidemiological tool for evaluations of preventive and control strategies.

**Objectives:** To estimate HAI prevalence and antimicrobial use prevalence, (ii) to describe the characteristics of patients and HAIs, (iii) to evaluate for HAI risk of factors.

**Methods:** During February-April of 6 consecutive years (2014-2019), and in November 2016, we conducted 7 PPS adopting the protocol of the European Center for Disease Prevention and Control (ECDC), Versions 4.3 – 5.3, at the 1300 acute-care beds San Martino Policlinic Hospital, a teaching hospital located in Genoa, North-West Italy. Multivariate linear regression was used to assess risk of factor for HAI insurgence.

**Results:** Overall, 6499 patients were enrolled, with a median age of 72 years (IQR: 57 – 81), a male:female ratio = 0.99:1 and a median HAI onset time of 15 days (IQR: 7 – 29). 50.0% of the patient had a Non-fatal McCabe score; 31.2% had an Ultimately-fatal, 17.1% had a Rapidly-fatal and 2.7% had an Unknown score. HAI prevalence decreased from 15.5% in 2014 to 11.2% in 2019 (Chi Square for linear trend, p < 0.01), while patients in antibiotic therapy decreased from 44.0% in 2014 to 42.4% 2019. Bloodstream and low-respiratory tract infections were the most frequent HAI. Staphylococcus aureus (12.4%) Staphylococcus epidermidis (12.0%) and Klebsiella pneumoniae (10.0%) were the most frequent microorganisms isolated overall bloodstream infections. In the multivariate logistic regression analysis, ultimately or rapid McCabe score, surgery, hospitalization in intensive care unit, central line catheter use and urinary catheter use were associated with a higher risk of developing an HAI.

**Conclusion:** PPS repeated over time have proved to be a valid tool to evaluate the trend of HAI and the use of antibiotics as an effect of prevention strategies adopted by the hospital health management.

**Disclosure of Interest**: None declared

### P174 POINT PREVALENCE SURVEY OF HEALTHCARE-ASSOCIATED INFECTIONS IN SLOVAKIA: COMPARISON DATA FROM SURVEY IN 2012 AND 2017

#### M. Stefkovicova^1^, S. Litvova^1^, M. K. Garabasova^1^, J. Brnova^2^

##### ^1^National Reference Centre for HAI, Public Health Authority , Trenčín; ^2^Centre of Microbiology and Infection Prevention, Trnava university, Trnava, Slovakia

###### **Correspondence:** J. Brnova

**Introduction:** Inadequate compliance to surveillance systems and lack of infection control professionals still exist in Slovakia, making it difficult to accurately assess the present burden of health care-associated infections (HAI).

**Objectives:** We compared data from European Centre for Disease Prevention and Control (ECDC) point prevalence study of HAI in Slovakia between year 2012 vs 2017.

**Methods:** Point prevalence survey of HAI was carried out according to a standardized methodology developed by the ECDC in Slovakia providing acute health care in June 2012 (40 hospitals) and June 2017 (50 hospitals). Data were collected at the country level, hospital level and the patient level according to standard protocol. Overall 8397 patients were included in the survey in 2012 and 9145 in 2017.

**Results:** Prevalence of HAI were similar in both survey (3,5 %/2012 vs. 4%/2017). The highest prevalence of HAI was found on the intensive care units. The most common types of HAI were urinary tract infections (25,8%/2012 vs. 26,2%/2017). The most frequent microorganisms were *Escherichia coli* (15,0 %) *in* 2012, but in 2017 *Clostridium difficile* (17,4%). We observed significant increasing consumption of alcohol hand disinfection from 9 L per 1000 patients’ day in 2012 to 18 L per 1000 patient’s day in 2017. Proportion of single bed hospital room increased from 5% to 17,2%, but only 10,2% single bed hospitals rooms have own hygiene facilities. Increasing proportion of Infection Control Practitioner in Slovak hospitals was observed (0,15/2012 vs. 0,3/2017). No change in administration of antibiotic prophylaxis between two periods was observed (80/2012 vs. 76/2017).

**Conclusion:** In Slovakia, we urgently need to establish infection control teams in health care facilities according ECDC standards and improving compliance to surveillance systems and antibiotic stewardship.

**Disclosure of Interest**: None declared

### P175 ANNUAL SURVEYS FOR POINT-PREVALENCE OF HEALTHCARE-ASSOCIATED INFECTION IN A TERTIARY TEACHING HOSPITAL IN MALAYSIA, 2016-2018

#### S. Ponnampalavanar^1,2^, S. Saaibon^2,3^, R. Zhazali^2,3^, S. S. Samsudin^2,3^, H. Che Hamzah^2,3^, N. Ghazali^2,3^, T. M. D. Tengku Mat^2,3^, R. Hassan^2,3^, J. Sarijo^2,3^, N. Razali^2,3^, S. N. Zainon^2,3^, M. Ishak ^2,3^, E. S. Y. Liew ^2^, S. S. L. L. Goh^2^, H. C. Ong ^1^, A. Kukreja^1^

##### ^1^Medicine, University of Malaya; ^2^Infection Control, ^3^Nursing, University Malaya Medical Centre, Kuala Lumpur, Malaysia

###### **Correspondence:** S. Ponnampalavanar

**Introduction:** Health care-associated infection (HAI) cause increased in morbidity and mortality in hospitalized patients. Little has known to the prevalence of HAIs in Malaysia.

**Objectives:** to investigate the point-prevalence of HAI in University Malaya Medical Centre (UMMC).

**Methods:** Three cross sectional studies was performed from 2016-2018 of hospitalized patients who had been diagnosed with HAI in UMMC. The data were collected using a structured data collection form by infection control nurses. Data was analysed using Statistical Package for the Social Sciences (SPSS) version 20.

**Results:** Out of 2465 hospitalized patients, 135(5.5%) were diagnosed with HAI. The rate if HAI was 4.5%, 5.1% and 6.9% in 2016, 2017 and 2018 respectively. The medical department had the highest rate of HAI (24.4%) followed by surgical (21.5%) and the intensive care unit (15.9%). The most common HAI was pneumonia (37.8%), followed by bloodstream infection (BSI) (19.3%) and surgical site infection (SSI) (10.4%). The prevalence of hospital acquired pneumonia (HAP) and BSI were found reducing from 2016 to 2018. The rate of HAP reduced from 40% to 35.6% in 2016 and 2018 respectively; BSI decreases from 31.4% in 2016 and 10.9 % in 2018. HAI was higher in males (57.8%), prolonged hospitalization (65.2%) and those who had intravenous lines (74.8%). *Pseudomonas Aeruginosa-non multidrug resistant* (12.6%) was the most common pathogen followed by extended-spectrum β-lactamase (ESBL) producing *Klebsiella pneumonia* (8.9%).

**Conclusion:** The study found the prevalence of HAI in our hospital is similar to previous studies across Asia, especially due to HAP, BSI and SSI. Interventions focusing on reduction of these HAIs in healthcare facilities should be prioritised to ensure patient safety while receiving care in acute care hopsitals.

**Disclosure of Interest**: None declared

### P176 THE PROCESS TRAINING FOR THE PILOT POINT PREVALENCE SURVEY OF HEALTHCARE-ASSOCIATED INFECTIONS AND ANTIMICROBIAL USE IN SELECTED ACUTE CARE HOSPITALS IN ALBANIA

#### A. Vasili, Z. Sulejmani, R. Daja, S. Bino, A. Fico

##### Institute of Public Health, Tirane, Albania, Tirane, Albania

###### **Correspondence:** A. Vasili

**Introduction:** Surveillance of HAI and antimicrobial use is an essential part of infection prevention and antimicrobial stewardship. It drives action by planning and implementing more effective, evidence based policies, surveillance and strategies

**Objectives:** Prepare hospital teams for the best knowledge of case definition for HAI and prepare for setting up and strengthening the Infection Prevention and Control programs.

**Methods:** The PPS will follow the standardized protocol and methodology devised by ECDC. The selection of hospitals in Albania, at least one general hospital will be enrolled. Hospital participation is voluntary.

**Results:** The consultant in close cooperation with the WHO Local Offices in Albania and a representative of the Institute of Public Health identified a national coordinator and a coordination center that will managed the PPS rollout in Albania.

The national coordinator in cooperation with the consultant identified the trainees at the national level and at the national level. Coordinator assisted in providing training.

Two hospitals, Tirana University Hospital Center and the Durres Regional Hospital were selected. The national coordinator in cooperation with the coordinators of each hospital appointed the team of the hospital. The Durres hospital team consisted of 6 people, 3 physicians and 3 nurses. The Tirana hospital team consisted of 6 physicians and 5 nurses, in total 11 persons.

During the training, people learned to look at participating hospitals: the prevalence of HAI and antimicrobial use in acute care; describes patients, invasive procedures, infections (sites, microorganisms including antimicrobial resistance markers) and the described antimicrobials. Much important in training was the standardization of HAI definition of cases and the recognition of participants with these definitions.

**Conclusion:** The information in this survey will help create policies and procedures to reduce HAIs, to improve methods to collect data on HAIs, support appropriate use of antimicrobial drugs and strength the implementation of Infection Prevention and Control programs at national and local levels.

The results obtained with this survey will assist national and local IPC teams to identify series of priorities for the prevention of HAIs and antimicrobial resistance in the selected hospitals.

**Disclosure of Interest**: None declared

### P177 FOUR NATIONAL POINT PREVALENCE SURVEYS OF HEALTHCARE-ASSOCIATED INFECTIONS IN SERBIA; WHAT WE HAVE LEARNED?

#### L. Markovic-Denic^1^, V. Suljagic^2^, G. Dragovac^3^, V. Mioljevic^4^, B. Mijovic^5^, I. Cirkovic^1^

##### ^1^University of Belgrade, Faculty of Medicine; ^2^Military Academy, Belgrade; ^3^University of Novi Sad, Faculty of Medicine, Novi Sad; ^4^Clinical Center of Serbia, Belgrade, Serbia; ^5^Faculty of Eastern Sarajevo, Foca, Bosnia and Herzegovina

###### **Correspondence:** L. Markovic-Denic

**Introduction:** Healthcare-associated infections (HAIs) represent an important public health challenge worldwide, especially in less developed counties. National HAI point prevalence surveys (PPS) allow identification of priorities and strategies for HAI prevention and control.

**Objectives:** To investigate prevalence of HCIs and to evaluate changes over time.

**Methods:** Four national PPSs of HAI were undertaken in Serbia; in 1998, 2005, 2010 and 2017. The first three national PPS applied CDC definitions, while the 4^th^ PPS applied ECDC definitions and methods. Trained infection control staff reviewed medical records to identify HAI active at the time of the survey. Data were collected in a single day in one ward with a maximum time frame of 2 weeks in one hospital and within one month for whole national survey.

**Results:** In first PPS participated 27 acute hospitals, 56 in second, 59 in the third, and 65 in 4^th^ survey. Increased number of included hospitals reflected increased awareness and recognition of the importance of HAI. The prevalence of patients with at least one HAI and the overall prevalence of HAI were 6.3% and 7.5% in 1998, 3.1% and 3.5% in 2005, 4.9% and 5.3% in 2010, and 4.3% and 4.6% in 2017. While three PPSs were conducted in autumn season, the second survey was conducted in May, and the lowest prevalence was recorded in this study, which pointing to the possible seasonal variation of HAI. The most frequent were surgical site infections (SSI) in first study (34.1% of all HAIs), and urinary tract infections (UTI) in the other three surveys (29.0%, 25.9% and 21.3%). After first study, first national HAI guidelines were prepared and started to implement in the practice. Validity survey which was organized along with 4^th^ PPS, showed that sensitivity of HAI detection was 85.1% and specificity 99.1%.

**Conclusion:** Repeated national PPSs provide data on the effectiveness and progress of infection control measures.

**Disclosure of Interest**: None declared

### P178 POINT PREVALENCE OF HOSPITAL ACQUIRED INFECTION IN THREE REGIONAL HOSPITALS IN BURKINA

#### Z. Gansane^1^, S. COULIBALY^1^, S. Yao^1^, A. Ouedraogo^1^, H. Hien^2^

##### ^1^Burkinabe Observatory for Healthcare Quality and Safety, ^2^Institut National de la Sante Publique, OUAGADOUGOU, Burkina Faso

###### **Correspondence:** Z. Gansane

**Introduction:** Healthcare-associated infections are a major public health problem, threatening the safety and well-being of patients. In Burkina Faso, HAI are common in health care facilities but their epidemiology is poorly described namely in regional hospitals.

**Objectives:** to perform a situational analysis through an epidemiological description in country's regional hospitals

**Methods:** We conducted in September 2018, a cross-sectional survey in three regional hospitals. All eligible inpatients admitted for at least 48 hours on the day of the survey were included. HAI was diagnosed based on CDC Atlanta's definition. The information was collected by physicians using the paper CRF and the patients' folder served as sources of information. Bacteriological samples were taken for the isolation of the germ and the sensitivity testing if needed. Data were analyzed with STATA 13 and R software. Univariate analyzes and a multi-varied logistic regression were performed to determine associated factors.

**Results:** During the survey, 216 patients were included in our sample. The median age of the patients was 23.6 years. Thirty-eight (38) cases of HAI were reported in our study, leading to an estimated prevalence of 17.59%, IC95 = [13.04-23.30]. Most of patients had received antibiotic therapy on the day of the survey (90.23%). Surgical site infections were the most frequent (34.21%). In multivariate analysis, patients over 55 years (OR = 0.13, CI = 0.02-0.65), pediatric patient (OR = 0.27, IC = 0.05-0.95) gynecological / obstetrical patient (OR = 0.13; 0.02-0.65), Koudougou hospital (OR = 0.19, IC = 0.06-0.52), intravenous catheter presence (OR = 4.06, IC = 1.15-15.45), length of stay over than 7 days since admission (OR = 2.35, CI = 1.01-5.48) were independent factors associated with the occurrence of HAIs. The Culture’s results showed that Staphylococcus aureus (57.8%) and Escherichia coli (26.3%) were most commonly isolated HAI-causing pathogens.

**Conclusion:** HAI’s prevalence remains high in our context and could be explained by the persistence of several factors related to the organization, the availability of human and material resources, human factors, the hospital environment

**Disclosure of Interest**: None declared

### P189 NATIONAL PREVALENCE SURVEY OF HEALTHCARE ASSOCIATED INFECTIONS: LESSONS LEARNED IN SENEGAL IN 2017

#### B. Ndoye^1^, N. N. KONATE^2^

##### ^1^ICAN, WHO Consultant; ^2^Coordonnatrice PRONALIN, MSAS, Dakar, Senegal

###### **Correspondence:** B. Ndoye

**Introduction:** Healthcare Associated Infections (HAIs) is a global public health scourge for which there is little reliable data available in Africa. The prevalence survey is currently the most accessible and realistic method in Africa, to have quantified data on the impact and put in place appropriate preventive measures.

**Objectives:** The objectives were to have quantified estimates of the prevalence rates of the main HAIs, the main bacteria responsible and resistance levels, but also to evaluate the practices in the antibiotics prescription.

**Methods:** The 2017 CDC and ECDC protocols were used as benchmarks and were adapted to the local context by a local technical working group, supported by experts from WHO and CDC. Ten hospitals were selected on the basis of criteria of representativeness, but also of functionality.

**Results:** Overall prevalence: 6%, although the collection of suspected cases by referring physicians gives 23%. Predominance of surgical site infections (52%) and low laboratory input with only 28% of cases where a sample for bacteriological diagnostic purposes was performed. Bacteriological aspects marked by the multi-resistance, even if the number of strains was low (11 strains of *Klebsiella pneumoniae* of which 20% resistant to carbapenems). Antibiotic patients on the day of the survey or the day before: 65% of patients included, of whom 22% had no documented infection or substantiation, third-generation cephalosporins being the most prescribed class. The 48th / 72nd hour assessments were not documented in 84% of cases and the justification for continued treatment beyond the first week was not documented in 75% of cases. Surgical antibiotic prophylaxis exceeded 24 hours in 65% of cases.

**Conclusion:** The prevalence is broadly comparable to previous studies, with rates underestimated, due to lack of information in patient records. The innovation of collecting the suspected cases by the treating physicians has allowed getting a clearer idea, and better reflecting the reality of the scourge in the African context, hence the importance of involving these staff in the survey, but also improving the quality of patient records.

The study also confirmed the predominance of infections in surgery, the low contribution of bacteriological diagnosis, as well as the misuse of antibiotics and the tendency towards bacterial multi-resistance, even with respect to carbapenems.

**References**

**Disclosure of Interest**: None declared

### P180 ESTABLISHING HEALTH CARE ASSOCIATED INFECTION (HCAI) SURVEILLANCE SYSTEMS IN PUBLIC REGIONAL HOSPITALS IN UGANDA

#### W. Omuut ^1^, M. Lammorde^1^, S. Jacob ^2^, J. Okware ^3^

##### ^1^Global health Security Program, Infectious Diseases Institute, Makerere University, Kampala, Uganda; ^2^Medicine , Liverpool School of Tropical Medicine, Liverpool , United Kingdom; ^3^Quality Assurance and Inspection Department, Ministry of Health, Uganda , Kampala , Uganda

###### **Correspondence:** W. Omuut

**Introduction:** The prevalence of HCAIs is arguably the most plausible indicator to measure effectiveness of health facility Infection Prevention and Control (IPC) programs. However, HCAI burden remains largely unknown in Uganda because no national system is in place for comprehensive and routine investigation.

**Objectives:** To establish HCAI surveillance systems in Public hospitals in Uganda.

**Methods:** In partnership with Ministry of Health a project was initiated to establish HCAI surveillance systems at select health care facilities. We reactivated and trained IPC committees at 8 regional referral hospitals (RRHs) to support HCAI investigation and drive necessary improvements. In April 2018, the first national HCAI Point prevalence survey (PPS) was conducted in 14 RRHs. Assessments were conducted using a World Health Organisation (WHO) 2002 PPS tool. Survey teams were pre-trained and site assessments conducted for 1 day per HCF for admitted patients.

HCAIs were classified as 1. Systemic defined as presence of two or more of the following: Heart Rate > 90 beats/min, Fever >38°C, Hypothermia < 36°C, hypotension < 90/60 mmHg, altered mental status, tachypnea > 25 breaths/min at least 48 hours after admission 2. Surgical Site Infections (SSIs); defined as contaminated, dirty or infected wounds for a surgery performed during the current hospitalization.

Survey results including prescription findings were summarized using descriptive statistics and disseminated at facility and national level to inform improvement. IPC Committees used results to design and implement improvements at respective health facilities.

**Results:** In the 14 RRHs,1100 patients were included in the survey. Mean prevalence (range) of HCAIs was 14% (6% - 24%). SSIs occurred most frequently at 44% then line related infections 13%, pneumonia 6%, blood stream infections 5%, urinary tract infections 2%. Microbiology investigation was not conducted. Most of the HCAIs (38%) were treated with a third or fourth generation cephalosporin; mostly intravenous ceftriaxone.

**Conclusion:** The project demonstrates feasibility of setting up HAI surveillance systems in a low-income country. We recommend integrating HCAI surveillance indicators into national monitoring and evaluation frameworks and to develop linkages with microbiology testing.

**Disclosure of Interest**: None declared

### P181 HEALTHCARE ASSOCIATED INFECTION IN RIBAT TEACHING HOSPITAL , SUDAN

#### M. Gamar Elanbya

##### Sudan, Khartoum, Sudan

**Introduction:** Healthcare associated infections in limited resources countries is a major patient safety problem, it is essential to understand the magnitude of the problem and address it.

**Objectives:** To assess the rate of health care associated infection and pathogen associated with it.

**Methods:** Active surveillance was carried for all inpatient who had been in the hospital for at least 48 hours, trained nurses were assigned for data collection, blood, urine and wound swabs were collected to determine the causative microorganism. Sample size was 504, calculated using the equation of z2 p q/d2.

**Results:** The rate of infection was 9.7%. 53% of infection are SSI, 32.7 % BSI and 14.3 % UTI (91% of UTI in female) 56.5 % of the sample no growth is detected. Klebsiella species causes 66.7 % of the infection, Staphylococcus species causes 22.2% of infection and pseudomonas species causes 11.1 % of the infection

**Conclusion:** The health care associated infection is considered high and surgical site infection is the most common one, further investigation is needed for the causes of no growth media

**References**

1- Benedetta Allegranzi,Julie Storr,Gerald Dziekan, Agnes leotsakos, liam Donaldson, Didier Pittet.The First Global Patient safety challenge" Clean Care is Safer Care":from Launch to current progress and achievements. Journal of hospital infection (2007)65(S2)115-123 p2

2-  WHO, Department of communicable disease surveillance and response: Prevention of hospital acquired infections, practical guide, second edition.

3-  SURGICAL SITE INFECTIONS, INTERNATIONAL NOSOCOMIAL INFECTION CONTROL CONSORTIUM REPORT, DATA SUMMARY OF 30 COUNTRIES, 2005–2010

IINFECTION CONTROL AND HOSPITAL EPIDEMIOLOGY june 2013, vol. 34, no. 6

4- May.O.Gamar Elanbya, an Assessment of Implementation of the Infection Control Program in six main Sudanese federal hospitals during the period from March-April 2006.

**Disclosure of Interest**: None declared

### P182 HEALTHCARE- ASSOCIATED INFECTIONS HAI SURVEILLANCE IN A PRIVATE HOSPITAL. A SUCCESS STORY OF SURVEILLANCE IN A CAIRO, EGYPT

#### J. A. A. Elkholy

##### Cairo University Hospitals, Dar Al Fouad Hospitals, Cairo, Egypt

**Introduction:** Health care-associated infections are the most frequent adverse event in health-care delivery worldwide. Limited data are available from low- and middle-income countries [1-2].

**Objectives:** To describe the results of the surveillance system from August 2018 till 27 May 2019.

**Methods:** Standardized active prospective surveillance system was conducted HAI definitions used were the same 2008 NHSN case definitions [4]. Antimicrobial susceptibility testing was performed using CLSI guidelines.

**Results:** Intensive care units ICUs contributed to 19 HAIs. Of these 6 (31.6%) are ICU acquired and 13 (68.4%) are infections present on ICU admission. Of the infections BSI represented 50% (primary BSI: LCBSI- CLABSI), UTI represented 33% (ABUTI- CAUTI) and pneumonia represented 17% (pneumonia type 1). The incidence of HAI were 2.1/ 1000 patient- days, CLABSI was 1.6/ 1000 central line days and CAUTI was 1/ 1000 urinary catheter days. Mechanical ventilation utilization ratio was 0.3 and central line and urinary catheter utilization ratio was 0.7 each. Culture of microorganisms showed that gram negative pathogens constituted 52.6% of the total pathogens, mainly Klebsiella spp. constituted (36.8%) and Acinetobacter spp. (15.8%).

**Conclusion:** Having a continuous and sustainable surveillance system is a success. Surveillance is fundamental to have benchmark of infections, to plan for prevention strategies, to record the antimicrobial resistance pattern and to plan for an antimicrobial stewardship program[4].

**References**

(1) Zimlichman E, Henderson D, Tamir O, et al. Health Care–Associated Infections: A Meta-analysis of Costs and Financial Impact on the US Health Care System. JAMA Intern Med. 2013;173(22):2039-2046. doi:10.1001/jamainternmed.2013.9763.

(2) Magill S., Jonathan R. Edwards, M.Stat., Bamberg W., Beldavs Z. Multistate Point-Prevalence Survey of Health Care–Associated Infections N Engl J Med 2014;370:1198-208

(3) See I., Lessa F., Incidence and Pathogen Distribution of Healthcare-Associated Infections in Pilot Hospitals in Egypt. Infection Control and Hospital Epidemiology, Vol. 34, No. 12 (December 2013), pp.1281-1288

(4) Gordts B, Vrijens F, Hulstaert F, Devriese S, Van de Sande S. The 2007 Belgian national prevalence survey for hospital-acquired infections. J Hosp Infect 2010; 75:163-167..

**Disclosure of Interest**: None declared

### P183 THE STATUS OF HOSPITAL INFECTION CONTROL PROGRAMS IN GOVERNMENT HEALTH FACILITIES IN METRO MANILA, PHILIPPINES

#### R. P. Berba, A. V. Dy Echo, M. A. Lansang, P. M. Pagkatipunan, R. Caro, A. Mejia

##### ^1^College of Medicine, University of the Philippines, Manila, Philippines

###### **Correspondence:** R. P. Berba

**Introduction:** Infection prevention and control (IPC) programs constitute one of the pillars of patient safety. Evaluation of existing IPC programs is key to planning educational and program interventions for quality improvement. To date, there has been no published report on the status of IPC in any group of hospitals in the Philippines.

**Objectives:** To evaluate the current status of IPC among 10 government healthcare facilities in Metro Manila Philippines.

**Methods:** This cross-sectional descriptive study evaluated 10 government hospitals in Metro Manila, Philippines. The pre-validated public domain instrument called the Infection Control Assessment Tool (ICAT)^1^ was used. This includes 22 modules, each focused on a specific IPC topic. Each hospital was surveyed by trained ICAT assessors using in-depth interviews with key staff, observation of hospital practices and record review. At the end of each hospital visit, a summary score of either A, B, or C was given according to the criteria set by the ICAT. Hospital performances were summarized and compared.

**Results:** Using the ICAT, the 10 health facilities were comprehensively evaluated. The common strengths were: (1) presence of an organized,recognized and active Infection Control Committees (ICC) in all the surveyed hospitals; (2) The ICC functions were broad and expansive. Most have IPC policies in place; (3) Significant portion of time and resources of the ICC committees was spent on IPC education of staff. The three IPC areas which need improvement are: (1) Surveillance of HAI is almost uniformly incomplete; (2) Antimicrobial stewardship is lacking; (3) Critical portions of isolation precautions particularly implementation of proper personal protective equipment (PPE) is poor.

**Conclusion:** Survey on IPC in 10 government hospitals in Metro Manila using the ICAT identified the top 3 strengths and weaknesses in their practice of IPC. It became evident that the need for IPC programs in these Philippine hospitals is recognized and valued; and that in general the implementation of IPC have a strong foundation and able to perform within expectations. Gaps identified include need for technical support and skills-building activities, training and financing of relevant materials, infrastructure and manpower.

**References**

1. https://www.cdc.gov/infectioncontrol/pdf/icat/hospital.pdf.

**Disclosure of Interest**: None declared

### P184 DEVICE ASSOCIATED INFECTION RATE AND BACTERIAL RESISTENCE IN AN EGYPTIAN UNIVERSITY HOSPITAL

#### M. M. Abdelhalim, on behalf of Mona Mohamed El_Khlousy**, Radwa Ahmed Rabea** Clinical Pathology Department - Faculty of medicine- Beni-Suef University** - Egypt

##### Clinical Pathology Department, Faculty of medicine- Cairo University, Cairo, Egypt

###### **Correspondence:** M. M. Abdelhalim

**Introduction:** Device-associated (DA)health care–associated infection(HAI) DA-HAIs are considered the principal threat to patient safety in the ICU and are among the main causes of patient morbidity and mortality.Standardized surveillance method allows for the determination of DA-HAI rates,comparable among health care centers,and provides infection control practitioners with a detailed picture of the institutional problems that they face, allowing them to devise effective solutions.

**Objectives:** To determine DAI rates, and the microbiological and antibiotic resistance profiles of infecting pathogens in ICUs of Beni-Suef University Hospital-Egypt

**Methods:** Prospective surveillance of HAIs was performed for adult and newborn patients admitted to adult and neonatal ICUs (NICU)of Beni-Suef university hospital from June 2012 to May 2013.DAIs rates were registered based on definitions applied by the Centers for Disease Control andPrevention᾽s National Healthcare Safety Network.

**Results:** The study included 303 patients were followed in ICUs for a total of 2,636 patient days.DAIs rate was88.5/1000 device days.VAP posed the greatest risk (68.7/1,000 ventilator days in the adult ICU, and 77.7/1,000 ventilator days in the neonatal ICU), followed by catheter associated urinary tract infections(CAUTI), then central line-associated bloodstream infections(CLABSI).The most frequently isolated pathogens inVAP were *Acinitobacter Spp.* (75%) in adultICU and *Klebsiella Spp.*(55%) in NICU. *Candida Spp.*were the leading pathogens in patients with CAUTI.InCLABSI, *Enterococcus Spp.*was the most frequently isolated pathogens (33%)in adultICUand*Klebsiella Spp.*(45%)inNICU.*All Staphylococcus aureus* infections were caused by methicillin-resistant strains, 88.9% of *Pseudomonusaeruginosa* were resistant to piperacillin-tazobactam, 45.6% were resistant to imipenem and flouroquinolones.Crude extra mortality was 10% among patients withVAPin adult ICU while66.7%in NICU,20% among patients with CLABSI in adult ICU while 26.7%in NICU, and 20% in patients with CAUTI.

**Conclusion:** The overall rate of DAIs was88.5/1000 device inICUs of our hospital.VAP was the most common DAI showing *Acenitobacter Spp.* as the commonest isolated organisms in adult ICU and *Klebsiella Spp.*in NICU. Establishment of active infection control programs that involve infection surveillance and implement guidelines for infection prevention can improve patient safety and quality of medical service

**Disclosure of Interest**: None declared

### P185 INCIDENCE OF BLOOD STREAM INFECTION (BSI) ASSOCIATED WITH ENDOSCOPIC RETROGRADE CHOLANGIOPANCREATOGRAPHY IN A TERTIARY HOSPITAL; 3 YEARS PROSPECTIVE SURVEILLANCE STUDY

#### Y. Leharova^1^, R. Rodrigues^1^, C. Frenette^2^

##### ^1^MONTREAL UNIVERSITY HEALTH CARE CENTER, Montreal, Canada; ^2^Infection Control & Prevention, MONTREAL UNIVERSITY HEALTH CARE CENTER, Montreal, Canada

###### **Correspondence:** Y. Leharova

**Introduction:** Our center is a 350 beds tertiary care referral center for hepatobiliary diseases and liver transplantation. Surveillance of health care associated BSI revealed that a significant proportion (12%) of HA-BSI were related to invasive procedures and the most frequent associated procedure being endoscopic retrograde cholangiopancreatography (ERCP) from 2016 to 2018.

**Objectives:** The goal of this study was to determine the incidence of bacteremia associated with ERCP, identify associated risk factors, complications and evaluate use of antibiotic prophylaxis regimen.

**Methods:** All BSI are reviewed to identify if associated with procedure within 7 days prior. The following variables were collected: primary diagnosis, types of hepatobiliary procedure performed indication and usage of antibiotic prophylaxis, dose, timing, complications and identified pathogen. The indications for the antibiotic prophylaxis were immunocompromised patient, liver transplant, biliary tree infection, neutropenic patient, etc.

**Results:** Over the three years, 44 BSI occurred following 1517 ERCP, at a rate of 3.0%. Indications were underlying Diagnosis: 18% cases were liver transplant cases, 9% cases were Gallbladder cancer, 61% cases were pancreatic or liver cancer, 30% cases were on chemotherapy; Symptoms prior ERCP procedure: Cholangitis 16%, liver obstruction 50%, stent stenosis 16%, liver abscess 7% and liver diseases. The most prevalent bacteria isolated were Klebsiella sp. 25%, *Escherichia coli* 25%and *Enterobacter sp.* 10%; *Enterococcus sp.* represented 11%. Prophylaxis antibiotic was not given prior to the procedure in 72% of cases; 14% cases received Ciprofloxacin and other antibiotics 14%. 72 % were hospitalized prior the procedure, 62 % required hospitalization, including 14 % to ICU. #30 day mortality was 9%.

**Conclusion:** The study reveals that many patients with ERCP associated BSI did not receive recommended antibiotic prophylaxis. This study indicates the need to improve prophylaxis through a standardized preprinted order for antibiotic prophylaxis that was developed, along with a high risk patient evaluation tool to evaluate patient’s risk factors prior to ERCP. These post-preventative measures implemented need to be evaluated for the outcome.

**Disclosure of Interest**: None declared

### P186 Withdrawn

### P187 CENTRAL LINE ASSOCIATED BLOOD STREAM INFECTION & CATHETER ASSOCIATED URINARY TRACT INFECTION : EPIDEMIOLOGY FROM A TERTIARY CARE HOSPITAL IN INDIA

#### M. P. Pillai^1^, A. Warrier^2^, S. Joy^3^, R. Babu^3^, S. Bernadit^1^, K. Charles^1^ on behalf of Aster Medcity

##### ^1^Infection Control; ^2^Infectious Diseases and Infection Control, ^3^Microbiology, Aster DM Healthcare LTD, Kochi, India

###### **Correspondence:** R. Babu

**Introduction:** Heath care associated infections (HAI) are important causes of morbidity and mortality world wide. In India, these infections are often under-reported and there are few reliable data. Given the paucity of data the objective of this study was thus to describe the epidemiology of HAIs, mainly CLABSI (Central line Associated Blood Stream Infection) & CAUTI (Catheter Associated Urinary Tract Infection) in our hospital - a quaternary care centre in India.

**Objectives:** To describe the epidemiology of HAIs, mainly CLABSI (Central line Associated Blood Stream Infection) & CAUTI (Catheter Associated Urinary Tract Infection) in our hospital - a quaternary care centre in India.

**Methods:** A retrospective study of case files of patients identified with a HAI during the period of January 2018 to December 2018 was conducted. HAIs were identified as part of routine infection control and prevention surveillance using the Centre for Disease control & National Healthcare Safety Network Criteria (CDC NHSN 2018) definitions. Rates were calculated and analyzed. The microorganism isolated and their antibiograms were also analyzed.

**Results:** Of 7484 patients included in the study, 2652 patients had a central line and 6711 had an indwelling urinary catheter. The rate of CLABSI was 3.8 per 1000 catheter days. The age group most affected was less than 20years. Gram Negative bacilli was the most common isolate of which *Klebsiella pneumonia* accounted for 30.7% . 41.66% of the *Klebsiella pneumoniae* strains showed multidrug resistance (MDR) and were resistant to the Carbapenems.

62 CAUTIs were identified during the study period with an incidence rate of 2.94/ 1000 catheter days. The most common age group was over 60 years (41.9%) and male sex was more commonly affected (51%). 51.6% of the patients were admitted with neurological diseases of which 24 required neurosurgery and had long duration of catheterization. The most common pathogen isolated was *Klebsiella pneumoniae* with around 59% being resistant to the Carbapenems and MDR

**Conclusion:** Key factors like competency assessment for staff involved in insertion and care of devices, and strict adherence to preventive bundles of care remain a challenge.

**Disclosure of Interest**: None declared

### P188 SEASONAL INCIDENCE TRENDS OF SURGICAL SITE INFECTION - THE RESULT OF ACTIVE SURVEILLANCE PROGRAM OF POLISH SOCIETY OF HOSPITAL INFECTIONS

#### A. Różańska^1^, J. Wójkowska-Mach^1^, A. Jarynowski^2^, M. Wałaszek^3^ on behalf of Polish Society of Hospital Infections

##### ^1^Jagiellonian University Medical College, Kraków; ^2^Interdisciplinary Research Institute in Wroclaw, Wrocław; ^3^State Higher Vocational School in Tarnów, Tarnów, Poland

###### **Correspondence:** J. Wójkowska-Mach

**Introduction:** Surgical site infections (SSI) surveillance and registration are necessary to determine the epidemiological situation and to launche effective preventive strategies. European Centre for Diseas Control and Prevention (ECDC) HAI-Net SSI program recommends the registration of SSI after selected surgical procedures for a period of at least three months.

**Objectives:** The aim of the study was to analyze the incidence trends for SSI registered as part of the active surveillance program run by Polish Society of Hospital Infections in accordance with the ECDC HAI-Net SSI protocol.

**Methods:** Definitions, infections detection criteria and registration forms in the Polish version were developed on the basis of the ECDC protocol.

SSI registration was carried out using the active method in various types of surgical procedures. In the years 2013-2018 eight hositals participated in the program, for the period time not shorter than one year.

**Results:** The study involved 33467 patients undergoing surgery (numer of operations), in which 477 cases of SSI were registered. Most of the SSI cases form of SSI was not given. The average incidence was 1.4% and ranged from 1.1% to 3.3% depending on the type of surgery. Incidence rates were significantly different depending on the month of infection detection. The highest rates were recorded in the winter months (December, January) and summer (June, July, August) in which fewer operations were performed than in other periods. No seasonality was observed for the distribution of etiological factors, among which Gram-negative rods from the *Enterobacteriacae* were dominant (24%).

**Conclusion:** The study has certain limitations. They are for example a small number of hospitals, lack of effective post-discharge surveillance or classification based on the systems, not specific operating procedures choosen for analysis. However, observation of the seasonally significant variability of incidence has practical implications. Firstly, the need for detailed control and validation of SSI prevention procedures during winter and summer holidays of staff. Secondly, the need of continuous targeted suveillance longer than three months period as a necessary condition of obtaining reliable epidemiological data and effective infection prevention.

**Disclosure of Interest**: None declared

### P189 VALIDATION OF AN INSTRUMENT FOR POSTOPERATIVE SURVELLANCE IN SURGICAL SITE INFECTION

#### G. B. Guatura, V. Poveda

##### PROESA, University of Sao Paulo, São Jose dos Campos, Brazil

###### **Correspondence:** G. B. Guatura

**Introduction:** Surgical site infection (SSI) represents the third cause of infectionrelated to health care. Considering hospital discharges that are becoming more precocious, post-discharge surveillance is extremely necessary, since its failure to perform can lead to underreporting, making it difficult to prevention and control actions. There is a lack,until now, of tools that have been validated for the identification of potential cases of SSI during post-discharge surveillance.

**Objectives:** To create and validate an instrument for the post-discharge detection of potential cases of surgical site infection through post-discharge surveillance.

**Methods:** Methodological study, using psychometric analysis, for the elaboration and validation of an instrument for the post-discharge surveillance of surgical site infection.

**Results:** The instrument had coefficient of validity of total content equal to 0.87. In the criterion and construct validation, it was applied to a sample of 100 patients and compared to the medical and nursing physical examination to detect surgical site infection resulting in Cohen´s kappa (0.83), Cronbach´s alpha (0.87) and Comparative Fit Index (0.998). The sensitivity was 76.4%; specificity of 100%; negative predictive values ​​of 92.5% and positive of 100%, and; accuracy of 94%.

**Conclusion:** The instrument was validated in the content, criteria and construct stages.

**References**

1. Berríos-Torres S, Umscheid C, Bratzler D, Leas B, Stone E, Kelz R et al. Centers for Disease Control and Prevention Guideline for the Prevention of Surgical Site Infection, 2017. JAMA Surgery. 2017;152(8):784. DOI: http://dx.doi.org/10.1001/jamasurg.2017.0904

2. World Health Organization (WHO). Global Guidelines for the Prevention of Surgical Site Infection. [Internet]. 2016. Available from:http://apps.who.int/iris/bitstream/10665/250680/1/9789241549882-eng.pdf?ua=1

3. United States of America. Centers for Disease Control and Prevention. Procedure associated module: Surgical Site Infection. [Internet]. Atlanta; 2018. [cited 2017 Oct 25]. Available from: https://www.cdc.gov/nhsn/pdfs/pscmanual/9pscssicurrent.pdf

6. National Institute for Health and Clinical Excelence/NICE. Guideline for Preventing, Identifying and Managing Wound Infection. [Internet]. 2017 [cited 2017 Oct 25]; Available from: https://www.nice.org.uk/guidance/CG74/chapter/1-Guidance#preoperative-phase

**Disclosure of Interest**: None declared

### P190 ASSESSING INFECTION PREVENTION, CONTROL, AND SURVEILLANCE IN KENYAN HOSPITALS PROVIDING CAESAREAN SECTIONS

#### S. Senglaub^1^, A. Sway^1^, A. Wanyoro^2^, A. Oburu^3^, J. Solomkin^1^ on behalf of World Surgical Infection Society

##### ^1^World Surgical Infection Society, Cincinnati, OH, United States; ^2^Obstetrics and Gynaecology, Kenyatta University, Nairobi; ^3^ACE Research, Kisumu, Kenya

###### **Correspondence:** S. Senglaub

**Introduction:** Caesarean section (CS) is a commonly performed major surgical procedure in Kenya; however, there is limited data available that describes infection prevention and control (IPC) readiness and surveillance in hospitals that perform this procedure.

**Objectives:** To assess IPC capacity including health care-associated infection (HAI) surveillance in Kenyan hospitals that perform CS.

**Methods:** We conducted a cross-sectional survey on IPC practices by adapting the World Health Organization IPC Assessment Framework. Surveys addressed four areas: IPC guidelines, surveillance, workload, and environment. Purposive sampling was used to identify 23 level-4 and -5, primary and secondary hospitals across 7 counties in southwest Kenya that provide CS. County-level permissions were obtained and data was collected from March through May 2019.

**Results:** IPC programmes were present in all 23 hospitals with 21 reporting leadership support and 14 with dedicated IPC budgets. While laboratory support was available in 21 hospitals, only 16 could support HAI surveillance. On-site labs were present in 8 of these hospitals while the remaining sent specimens off-site. Surveillance was conducted for: surgical site infections (11/23; 48%), device-associated infections (7/23; 30%), multidrug resistant pathogens (5/23; 22%), and infections in vulnerable populations (8/23; 35%). Antimicrobial drug resistance was analyzed regularly in 17% of hospitals and surveillance data was used for quality improvement in 44%. Functioning hygiene stations were available in 65% of hospitals. Water services and power supply were sufficient in 91% and 95% of hospitals, respectively.

**Conclusion:** Critical components of IPC programmes were lacking in the majority of hospitals including financial support, dedicated surveillance resources, and laboratory infrastructure. Moving forward, hospitals should consider identifying local champions, developing educational campaigns, and utilizing the IPC Core Component Guideline for self-assessment and recommendations to improve programme capacity.

**Disclosure of Interest**: None declared

### P191 SURVEILLANCE OF SURGICAL SITE INFECTIONS IN INDIA- ACTIVE VERSUS PASSIVE METHOD

#### D. Sureshkumar^1^, S. Saravanakumar^2^, J. Hemalatha^3^, A. Jennifer^4^

##### ^1^Infectious Diseases, Best of IDs, Chennai; ^2^Medicine, KMC Manipal, Mangalore; ^3^Pharmacy, Best of IDs; ^4^Infectious Disease, Madras Medical Mission, Chennai, India

###### **Correspondence:** D. Sureshkumar

**Introduction:** Surgical site infections (SSIs) are one of the most common health care associated infections (HAIs) in the developing world as well as most feared HAIs. Despite the availability of standard definitions and surveillance methods there are major concerns are there in the consistency & completeness of SSIs surveillance in the developing world.

**Objectives:** The main objective of this study was to describe & to compare the surveillance methods used by two accredited hospitals in South India in the estimation of SSIs burden.

**Methods:** Retrospective analysis of the SSIs case records prepared by infection control nurses working in 2 tertiary referral & accredited hospitals in South India between January 2017 and September 2018. The key parameters analyzed were total number of surgeries performed, SSI rate, timing of SSIs (before hospital discharge or after hospital discharge) and surveillance method (passive- microbiology positive cultures or active- case finding by daily bed side rounds, outpatient follow ups telephonic follow up of discharged patients).

**Results:** Totally 8042 surgical procedures with 149 SSIs (1.85%) occurred during the study period. 2899 surgeries happened in the first hospital with 26 SSIs (0.89%). However, in second hospital 5143 surgeries happened at the same time with 123 SSIs (2.39%). Majority of the SSIs happened after the discharge from both the hospitals (103/149, 69.12%). Passive surveillance method (culture positive reports from microbiology laboratory) identified 143 of 149 SSIs (95.97%). Very few cases surgeon identified the SSIs and informed the infection control nurses in this study.

**Conclusion: :** Most of the SSIs happened after discharge and passive surveillance method was the main method used to identify SSIs in the Indian hospitals. Urgent measures are required to improve the active SSIs surveillance and to find the real SSIs burden in India.

**Disclosure of Interest**: None declared

## Poster session: Outbreak

### P192 FUSARIUM SPP OUTBREAK IN ONCOHEMATOLOGIC UNIT

#### W. Cornistein^1^, A. Novau^1^, L. Fabbro^1^, L. Paulosky^1^, G. Pineda^2^, M. L. Pereyra^3^

##### ^1^Prevention and Infection Control; ^2^Microbiology; ^3^Infectious Disease, Austral University Hospital, Buenos Aires, Argentina

###### **Correspondence:** W. Cornistein

**Introduction:** *Fusarium spp* can disseminate causing an invasive fusariosis (IF) with mortality rate of 75% in immunocompromised (IC) hosts. F*usarium* sp are ubiquitous molds which can be found in the soil, water, and air. In our hospital, the incidence of IF increased from 0.56 ( 2° semester of 2018) to 5.3 (1° trimester 2019) 1000 IC patiens day

**Objectives:** To describe a *Fusarium spp* outbreak in oncohematological patients and the strategies to control it.

**Methods:** This study was performed at a 210-bed private tertiary-care university hospital located in Buenos Aires. The Bone Marrow Transplant (BMT) ward has 8 individual rooms, HEPA filters and positive pressure airflow. The hematology ward has 8 private rooms with no controlled air. All patients with hematologic malignancies were hospitalized in this ward.

The Infection Control Department performed:1-Case analysis and possible source of infection; 2-Inspection of the hospital infrastructure and rooms; 3-Environmental samples from air, surfaces and water; 4- Examination of cleaning and disinfection policies; 5. Specific measures on patients regarding shower and use of drinking water.

**Results:** From Jun 2018 to january 2019, we detected 5 cases of IF caused by *Fusarium solanii.* Most patients presented neutropenia, fever with cutaneous portal entry. All patients were treated with a combination of antifungal drugs. Mortality was 40%.

72% of the rooms had either visible fungi in bathroom walls or uneven surfaces which made imposible an adequate cleaning. We remodeled each room and bathrooms.

Air samples were negative. All showers and sink surfaces samples in BMT unit, hematologic and general wards were positive to *Fusarium spp.*proving the hypothesis of waterborne dissemination. External faucets were uncoupled and both pieces and surfaces were cleaned with hyperchlorination and 20% quaternary ammonium. A water filter was installed in one room.

The patients' bathroom and the use of water were suspended. No more cases have been detected.

**Conclusion:** The environmental study of an outbreak identifies risk factors for IC patients making it possible to improve conditions and reduce the incidence of serious and potentially lethal cases.

**Disclosure of Interest**: None declared

### P193 INFLUENZA A OUTBREAK IN A HEMODIALYSIS UNIT AT A TERTIARY CARE CENTER IN LEBANON

#### J. Tannous^1^, N. K. Zahreddine^1^, R. M. Attieh ^2^, H. Mezher ^3^, Z. Kanafani^4^, S. Kanj^4^

##### ^1^Infection Prevention and control; ^2^internal medicine; ^3^nursing services; ^4^infectious diseases , AUBMC, Beirut, Lebanon

###### **Correspondence:** J. Tannous

**Introduction:** Patients undergoing hemodialysis (HD) are at an increased risk for Influenza and its complications. Annual influenza immunization is recommended for eligible HD patients and Healthcare Workers (HCWs) working in Hemodialysis units (HDUs). The vaccine is presumed to induce immunity despite reports of insufficient effectiveness in HD patients.

**Objectives:** The aim of the study is to describe an outbreak of influenza A among patients undergoing dialysis.

**Methods:** An outbreak caused by Influenza A virus occurred between February 19 and March 14, 2019 in the 24-chairs HDU at the American University of Beirut Medical Center. Influenza infections were confirmed by either *Rapid Influenza* Diagnostic *or* Reverse Transcription Polymerase Chain Reaction (RT-PCR) tests. All patients, staff and visitors were required to wear face masks when accessing HDU as directed by the Infection Control (IC) team. All exposed patients received prophylactic oseltamivir. Exposure management for HCWs was also initiated. Exposed HCWs were referred to Employee Health Clinic (EHC) for medical assessment and work restriction when indicated.

**Results:** A total of 11 patients out of the 100 HD enlisted patients (11%) were confirmed to have Influenza A infection during the outbreak period. 10 out of the 11 patients were hospitalized (91%) and all patients recovered. 5 patients gave a positive history of flu vaccination. The flu vaccination uptake rate for the 100 patients was 66%. One patient developed influenza despite prophylactic oseltamivir. Moreover, 6 of the 8 exposed nurses developed flu like illnesses, but none were tested. None of them had received vaccination and all were restricted from work until cleared by EHC.

**Conclusion:** The IC measures that were implemented were effective in halting the transmission to other patients. A mandatory Influenza vaccination targeting HD staff and patients was adopted for the coming seasons. Additional research is needed to resolve dosage (high-dose or booster) requirements of the flu vaccine in HD patients. Furthermore, screening HD patients at the point of entry for flu-like illnesses is essential during the Influenza season for proper implementation of IC measures.

**Disclosure of Interest**: None declared

### P194 EMERGENCE OF ELIZABETHKINGIA MENINGOSEPTICA AND RALSTONIA PICKETTI IN CRITICALLY ILL PATIENTS – INNOCENT BYSTANDERS OR POTENTIAL THREAT?

#### M. C. Dimabuyu^1^, S. Santos^2^, S. Abad Santos^3^

##### ^1^Medicine, St. Luke's Medical Center; ^2^St Luke's Medical Center, Taguig; ^3^St. Luke's Medical Center, Taguig City, Philippines

###### **Correspondence:** M. C. Dimabuyu

**Introduction:** *Elizabethkingia meningoseptica* and *Ralstonia picketti* are both gram-negative bacilli of low virulence that is ubiquitously found in hospital environments, hence associated with various nosocomial infections. Immunocompromised individuals are particularly at increased risk for developing severe infections with high mortality rates partly due to multidrug resistance.

**Objectives:** We described here 2 cases of *E. meningoseptica* bacteremia and 2 cases of hospital acquired pneumonia with *R. picketti* showing resistance to multiple antibiotics, ensuing treatment and investigation in our institution.

**Methods:** Over a period of 4 months, two critically ill patients were positive for *E. meningoseptica* from blood cultures. Isolates were identified using rpoB sequence cluster analysis. Another two patients both intubated and on hemodialysis were identified with *R. picketti* isolates from respiratory tract.

**Results:** *E. meningosepticum* isolates has shown resistance to most antibiotics useful against Gram-negative bacteria (aminoglycosides, beta-lactams, chloramphenicols) but it is susceptible to Vancomycin and Ciprofloxacin. Resistance may be due to both class A extended spectrum beta lactamases (ESBL) and class B metallo-β-lactamases (MBLs). Both cases were given Vancomycin which showed clearance from repeat blood cultures done after 7 days.

Resistance of *R. pickettii* infections could be due to the presence of mobile genetic elements: blaOXA-22 which has activity against benzylpenicillin, cloxacillin and restricted-spectrum cephalosporins and blaOXA-60 which has activity against imipenem. Both were given Tigecycline based on sensitivity results. Repeat cultures were negative.

Infection control had been alerted and investigation revealed no commonalities between patients other than having been inpatient on the same unit at different times. Hemodialysis machine used were different. Water samples from outlet were negative.

**Conclusion:** When the isolation of unusual microorganisms occurs, an active search should be initiated that includes microbiological examination of administered fluids, medications, hemodialysis water, mechanical ventilators and culture materials. Such cases must be reported to the committee for infection control to prevent and control outbreaks.

**Disclosure of Interest**: None declared

### P195 RAPID CONTROL OF SERRATIA MARCESCENS OUTBREAK IN NICU, OMAN, SEPTEMBER-OCTOBER 2018

#### Z. A. Al Maskari^1^, M. AL Hinnai^2^, K. AL Riyami^3^, L. AL Ghabshi^4^, A. AL Rashdi^4^, A. K. AL Jardani^4^

##### ^1^Infection prevention & control, Royal Hospital, Muscat; ^2^Infection prevention & control; ^3^NICU, Royal Hospital; ^4^Medical Microbiology, Central Public Health Laboratory, Muscat, Oman

###### **Correspondence:** Z. A. Al Maskari

**Introduction:** *Serratia marcescens* is an important opportunistic pathogen combining a propensity for healthcare-associated infection and antimicrobial resistance. Outbreaks are frequently reported in neonatal intensive care units.

**Objectives:** The aim of this study to describe the outcome of infected neonates, emphasize on the importance of enhancing various infection control practices to control the outbreak and describe the role of molecular typing.

**Methods:** from september to October 2018, the NICU of our hospital experienced an outbreak of *Serratia marcescens.* The screening for Serratia was initiated for all neonates at risk after the second case detected positive, a total of 9 cases involved (5 had bacteremia, 4 were colonized). Extensive environmental microbiological sampling conducted, and five clinical isolates were typed using PFGE. Cohorting of infected/colonized neonates done, healthcare workers awareness and hand hygiene practices were enhanced, frequent environmental and non-critical medical equipment disinfections were carried out.

**Results:** During outbreak surveillance, 90 neonates were screened by rectal swab and other swabs taken based on the case (ET for ventilated neonates, wound swabs). 5 neonates detected by screening,1 of them had bacteremia. Three of the cases that had bacteremia had negative screening. Unfortunately, 3 of the bacteremia cases died with sepsis being the direct cause. Factors contributed to the outbreak were overcrowding, low compliance among the unit staff and among external medical staff visiting the unit with infection control practices after working hours and late start of appropriate antibiotics in septic neonates. 86 environmental samples were cultured results of which came negative. Molecular typing showed only two strains were identical, one was un-typable, and the other two were closely related.

**Conclusion:** Serratia marcescens bacteremia has a high mortality. Enhancing various infection control practices is very vital for rapid control of Serratia marcescens outbreak.

**Disclosure of Interest**: None declared

### P196 CANDIDA AURIS OUTBREAK EXPERIENCE IN A MEDICAL INTENSIVE CARE UNIT OF A TERTIARY, GENERAL HOSPITAL IN QATAR, 2019

#### F. Altura - Visan^1^, N. Al Ansari^2^, W. Al Wali^1^, A. Zakaria^3^, E. Karic^4^ on behalf of Critical Care Outbreak Team members, J. Al Ajmi^5^, M. Al Jonidi^4^ on behalf of Critical Care Outbreak Team members, O. Al Hasanat^6^, J. Castro^6^, G. Hudaib^6^, M. Asuncion^6^, U. Ummer^6^, D. Hamdani^7^ on behalf of Candida auris Outbreak Group, Al Wakra Hospital

##### ^1^Infection Prevention and Control, Al Wakra Hospital - Hamad Medical Corporation; ^2^Infection Prevention and Control, Al Wakra Hospital; ^3^Infection Prevention and Control, AL Wakar Hospital - Hamad Medical Corporation; ^4^Critical Care Division, Al Wakra Hospital - Hamad Medical Corporation, Al Wakra; ^5^Corporate Infection Prevention and Control, Hamad Medical Corporation, Doha; ^6^INFECTION PREVENTION AND CONTROL OFFICE, Al Wakra Hospital - Hamad Medical Corporation, Al Wakra Hospital - Hamad Medical Corporation, Al Wakra; ^7^INFECTION PREVENTION AND CONTROL OFFICE, Al Wakra Hospital - Hamad Medical Corporation, Ministry of Public Health, Doha, Qatar

###### **Correspondence:** F. Altura - Visan

**Introduction:** *Candida auris* is known to be invasive, multidrug resistant and can cause outbreaks in hospitals. Mode of transmission is through contaminated hospital items i.e. fomites and intervention of staff. The outbreak of *Candida auris* affecting four patients at the Medical Intensive Care Unit (MICU) either in the form of infection or colonization is the first documented outbreak occurring in the State of Qatar.

**Objectives:** The objectives for this presentation are to identify the risks of patients involved, discuss interventions for outbreak control and add knowledge to IPC community regarding *Candida auris.*

**Methods:** First case identified on 11/2018 in a patient colonized in respiratory tract. Candida auris biweekly tests were conducted. Outbreak cases were identified as second case colonized in the decubitus ulcer. Third case as bloodstream infection, treated accordingly with recovery from septicemia. Fourth case colonized in the axillae.

**Results:** Root cause analysis suggests, index case imported from Pakistan (known *Candida auris* infection), followed by subsequent transmission to other patients in MICU. Geographically a particular room number 2 where all four patients have occupied is the potential source of transmission. Multiple evidence-based infection prevention and control actions, were pursued including stricter monitoring programme, decolonizing by using topical antifungals, environmental sampling, terminal cleaning, and increasing awareness.

**Conclusion:** The outbreak is not yet terminated, there's continuous IPC interventions. Outbreak control was conducted within a strong MDT using latest scientific evidence. We recommend it is imperative that everybody complies with IPC practices. Unfortunately there are still colleagues even senior consultants refusing to comply. The need for Hydrogen Peroxide vapor disinfection machine to kill highly contaminated environments.

**Disclosure of Interest**: None declared

### P197 OUTBREAK AND CONTROL OF ACHROMOBACTER DENTRIFCANS INFECTIONS AT A TERTIARY HOSPITAL IN PRETORIA, SOUTH AFRICA

#### M. Said^1^, Y. Dangor^2^, R. Naidoo^2^, B. Mitton^2^, N. Mbelle^2^, M. Naicker^2^, K. Katlego^2^, V. Amutenya^2^

##### ^1^NATIONAL HEALTH LABORATORY SERVICES; ^2^Medical Microbiology, University of Pretoria, Pretoria, South Africa

###### **Correspondence:** M. Said

**Introduction:** From June-October 2018, the Tshwane Academic Microbiology lab cultured 38 *Achromobacter dentrificans* patient isolates (baseline was 1-2 isolates per month) from the Steve Biko Academic Hospital (SBAH). The majority of the isolates were cultured from blood (n=23).

**Objectives:** To determine the source of the *Achromobacter dentrificans* outbreak

**Methods:** A line list was developed for each case. Each case had an interview conducted with the attending clinician, the patient and the person who collected the lab specimens. A common feature was that for all procedures, the antiseptic used was chlorhexidine and water solution prepared at the hospital pharmacy. The chlorhexidine and water solutions from various wards were collected for investigations. The pharmacy was visited and swabs were taken from empty bottles and caps of the bottles used for chlorhexidine and water solutions, water used for the preparation, the stock chlorhexidine solution, the solution colourant, utensils used in the preparation, the dispenser nozzle used to dispense the solution into the bottles and the final product. The chlorhexidine and water solutions as well as the swabs were plated on to 5% blood agar, chocolate agar and MacConkey agar and incubated at 35-37^o^C for 48 hours. Final identification and antimicrobial susceptibilities (AST) of colonies was done using Vitek 2 (Biomerieux, France). REP-PCR was performed to determine molecular relatedness of the isolates.

**Results:** Growth of *Achromobacter dentrificans* was noted from all chlorhexidine and water solutions tested from the wards as well as the dispenser nozzle from the pharmacy. According to REP-PCR, two strains of *Achromobacter dentrificans* were identified from the pharmacy. Both these strains were identified in patient samples. The majority of patients were not clinically septic.

**Conclusion:** The hospital pharmacy was advised to recall chlorhexidine and water solutions from all wards at the end of October 2018. It was advised that 4% chlorhexidine gluconate be used in future for skin antisepsis and no further chlorhexidine and water preparations be made in pharmacy. Following this intervention, the number of clinical isolates decreased dramatically to the baseline of 1-2 isolates per month from November to May 2019.

**Disclosure of Interest**: None declared

### P198 A CLUSTER EPIDEMIC CAUSED BY E.COLI INFECTION AMONG PATIENTS WHO WERE UNDERGOING URODYNAMIC TESTING IN A TEACHING HOSPITAL IN CHINA

#### L. Li

##### Department for HAI control, West China Hospital of Sichuan university, Chengdu, China

**Introduction:** As a valid tool to evaluate the urinary incontinence, the urodynamic testing is usually applied in clinical practice. However, it was frequently associated with nosocomial infections due to lack of standard precautions.

**Objectives:** To confirm the cluster epidemic and explore the source of the epidemic, we investigated a cluster epidemic of *E.coli* infection after the urodynamic testing in a teaching hospital.

**Methods:** Field investigation was used to collect the demographic and clinical data through HIS system. Case control study was implemented to assess the risk of getting infection. Environmental samples from urodynamic device and related wards were tested by PCR. ERIC-PCR was used to test the similarity between urinary specimens and environmental samples. Medical records were reviewed finally to identify new infections in one month afterwards.

**Results:** Totally there were 25 inpatients underwent urodynamic testing during the epidemic period. Case control study showed urodynamic testing was a risk factor for the patients to get urinary infection(OR=2.44, *P*=0.003).5 specimens of 5 patients, which mainly came from ward A and ward B, and one environmental sample were tested positive of *E.coli*. and the ERIC-PCR result between the *E.coli* strain isolated from the 4 specimens of 4 patients and one environmental sample from a urinary funnel showed highly similarity. Field investigation showed bleach of infection control policy during the testing.

**Conclusion:** Undertaking urodynamic testing is a risk factor of getting *E.coli.* infection, and bleach of infection control policy during Urodynamic testing is commen. Strict infection control measures must be followed and the active surveillance for nosocomial infection in hospital played a key role to identify the unexpected event.

**Disclosure of Interest**: None declared

### P199 INVASIVE FUNGAL INFECTION IN AN INTENSIVE CARE UNIT RELATED TO STRUCTURAL COMPONENTS AND HEALTHCARE WORKERS BEHAVIOURS

#### D. Hilliquin, A. Regard, N. Khanafer, E. Marion, P. Cassier, T. Rimmelé, M. Bertin-Maghit, O. Martin, P. Vanhems

##### Hospices civils de Lyon, Lyon, France

###### **Correspondence:** D. Hilliquin

**Introduction:** Nosocomial infection can occur from care or environmental contamination like airborne pathogens. To reduce this risk, air treatment is set up in units which admit high-risk patients of invasive fungal infections

**Objectives:** Evaluate relationship between serious fungal infection and structural components and healthcare workers (HCW) behaviours

**Methods:** In December 2018, the burn intensive care unit (ICU) of a French university hospital alerted the infection control unit of 3 nosocomial invasive fungal infections. Two Mucorales *(1 Rhizopus microsporus and 1 Lichtheimia spp.)* and 1 *Aspergillus fumigatus* infections were reported in 3 patients hospitalized in 3 different rooms. Investigations by the infection control team (ICT) were conducted including air samples, structural (environmental and technical constituents) and HCW behaviours

**Results:** Burn-ICU is composed of 10 rooms with positive air flow. Two of them have a flow reversal system. Each room is composed of two doors (1) corridor - airlocks door and (2) airlock - room door. Two 500L air samples were performed in each room of patients with Mucorales infection. In one room, 4 CFU/m^3^ *Aspergillus versicolor* and 12 CFU/m^3^ *Penicillium sp.* were isolated in one sample and 6 CFU/m^3^ *A. versicolor* were found in the second one. Inspection of structural components showed: dust in some supply grids and inappropriate fixing of some false ceiling panels. Front doors without windows were opened frequently because of the lack of visibility. Regarding organizational components, removing cardboard were performed in the corridor with opened front doors. Cardboard were also found in airlock rooms. Moreover at least 1 of the 2 doors was opened in the majority of rooms conducting to an ineffective air treatment or a flow inversion. Following the investigations, the technical unit intervened to create windows for front doors and to fix ceiling panels. Moreover, ICT achieved training session for HCW about the importance of air treatment

**Conclusion:** This investigation shows the importance of air treatment in unit with patient at high-risk of developing severe invasive infection linked to environment. HCW behaviour should be considered as a principal actor to improve control measures against the spreading of airborne infection in hospital

**Disclosure of Interest**: None declared

### P200 CONTROL OF CARBAPENEM RESISTANT ACINETOBECTER BAUMANII INFECTIONS IN AN ENDEMIC HOSPITAL SETTING

#### K. Labay^1^, I. Aharon^1^, I. Asael^2^, O. Nitzan^1,3^, A. Peretz^4^, S. Soboh^2^, H. Zayyad^1^

##### ^1^Infectious Disease Unit; ^2^Internal Medicine B, Poriya medical center, Poriya; ^3^Faculty of medicine, Bar Ilan university, Zefat; ^4^Microbiology Unit, Poriya medical center, Poriya, Israel

###### **Correspondence:** K. Labay

**Introduction: Introduction:** Carbapenem resistant Acinetobacter baumanii (CRAB) is one of the most common resistant pathogens causing hospital acquired infections.

**Objectives:** Following an outbreak of CRAB infections in an internal medicine ward, we used a multimodal approach to implement guidelines and reduce the incidence of CRAB acquisition at the hospital level.

**Methods: Methods:** We conducted a study of the incidence of CRAB infections during the years 2017-2019 at our 350 bed hospital. An intervention was implemented during July-September 2018 after an outbreak of CRAB infections in an internal medicine ward. Before intervention, infection control measures included contact precautions and environmental disinfection. Our intervention included cohorting or single room isolation, monitoring environmental disinfection, screening cultures of at-risk patients. Patients screened positive for CRAB were isolated as well. To facilitate implementation of the intervention we engaged the hospital management, department leaders, teams in charge of environmental disinfection and the microbiology laboratory.

The primary outcome was CRAB infection rate (Incidence rate) per 100,000 patient days each year, before, during and after the intervention.

**Results: Results:** During the baseline period (2017), the rate of CRAB infections was 57 cases per 100,000 patient-days; 20 cases per 100,000 patient days were acquired during the year of intervention, 2018, relative risk reduction (RRR) 0.65 (p<0.0001); and during the first 5 months of 2019 rates declined further to 6 cases per 100,000 patient-days (RRR 0.89, p<0.0001).

**Conclusion: Conclusion:** We believe the outbreak served as a booster for implementing infection control measures. An intervention involving the ward medical staffs, ward leaders, cleaning teams, and the hospital management prevented outbreak escalation, and caused a sustained decline in CRAB acquisition at the hospital level. CRAB outbreaks can be controlled using a multimodal approach of infection control measures.

**Disclosure of Interest**: None declared

### P201 INFECTION PREVENTION AND CONTROL (IPC) IN NATURAL DISASTERS AND OUTBREAKS – EXPERIENCES FROM HUMANITARIAN RESPONSE TO TROPICAL CYCLONE (TC) IDAI IN MOZAMBIQUE, FOLLOWED BY AN OUTBREAK OF CHOLERA

#### D. Peter^1^, N. Stücke^2^, F. Mattner^1^

##### ^1^Institute of Hospital Hygiene, Kliniken Köln, Lehrstuhl für Hygiene und Umweltmedizin, Universität Witten-Herdecke; ^2^Arbeiter-Samariter Bund Deutschland e.V. (ASB), Köln, Germany

###### **Correspondence:** D. Peter

**Introduction:** In the aftermath of TC Idai an outbreak of cholera occurred in the district of Beira, Mozambique.

**Objectives:** Embedded in the humanitarian response ASB’s WHO-classified Emergency Medical Team (EMT) focused on the implementation of IPC measures to support health centers (HC) in Beira District during the outbreak. Here we report our approach, experiences and lessons learnt from this deployment, implementing an IPC-tool that has been developed and pilot-tested in Zambia in 2018/2019.

**Methods:** A multidisciplinary team consisting of WASH-experts (water, sanitation and hygiene) and IPC professionals conducted a structured assessment of 4 health centers, each providing care for 120-150 patients per day. Contents for an IPC-training (software) and construction needs to facilitate the implementation of IPC-measures (hardware) were identified. A training was tailored to the needs and local circumstances in close collaboration with local health care workers, health authorities and the WHO.

**Results:** Sings of infectious diseases, transmission of pathogens and transmission-based precautions, safe handling of personal protective equipment (PPE), relevant diseases and reporting pathways were part of the training in which representatives from 12 (of 17) HC in Beira District participated. Hardware installed in 4 HC included hand wash facilities, safe waste management areas, water filters and additional waiting areas for infectious patients.

**Conclusion:** A relatively short intervention in the midst of an outbreak cannot aim at sustainably implementing IPC-measures. Whilst the threat is immanent however a bundle of needs-based and context-adapted soft- and hardware measures can help increase the safety of HC when managing infectious patients. This may help to protect the valuable resource of health professionals, preserve the population’s trust in the local health system and prevent the spread of infections between patients during humanitarian crises. A complementary approach that combines training and necessary materials and structural preconditions to put theory into practice is crucial.

**Disclosure of Interest**: None declared

### P202 DEVELOPMENT AND PILOT TESTING OF AN INFECTION PREVENTION AND CONTROL (IPC) TOOL FOR HUMANITARIAN RESPONSE TO OUTBREAKS AND NATURAL DISASTERS

#### D. Peters^1^, N. Stücke^2^, F. Mattner^1^

##### ^1^Institute of Hospital Hygiene, Kliniken Köln, Lehrstuhl für Hygiene und Umweltmedizin, Universität Witten-Herdecke; ^2^Arbeiter-Samariter Bund Deutschland e.V. (ASB), Köln, Germany

###### **Correspondence:** D. Peters

**Introduction:** Outbreaks occur worldwide independent of or in the aftermath of natural disasters. The WHO Emergency Medical Teams (EMT) initiative has contributed to quality assurance in humanitarian response. The response to outbreaks has recently gained relevance for EMTs.

**Objectives:** Here we report the development and pilot testing of an IPC-tool by ASB’s WHO-classified EMT.

**Methods:** 1. CDC’s Infection Prevention and Control Assessment Tool1 was adapted to the context of rural health centers (RHC) in Mumbwa District, Zambia. 2. A structured focus group discussion was conducted to identify relevant contents of an IPC-curriculum with representatives of the district health office (n=2) and local health care workers (HCW, n=5). 3. A workshop was held to identify preferred didactic methods. 4. A table-top exercise (TTEX) was developed to measure HCWs’ performance when managing infectious patients.

**Results:** A training curriculum was developed followed by a 3-day training held in Feb. 2019. 15 participants of 13 RHC participated. 13 participants completed a pre-/post-training multiplechoice IPC-questionnaire (MCQ). 7/13 RHC, providing care for a population of over 36.000, completed a full evaluation cycle consisting of: 1. Structured assessment of the RHC1 2. Pre-/post-/post-post (3 months after training) MCQ of training participants 3. Pre-/post-post MCQ of one non-participant health worker per RHC to evaluate multiplication activities of participants 4. Pre-/post-post TTEX to evaluate participants’ performance when managing infectious patients in the RHC Participants of these 7 RHC included 4 environmental health technicians, 2 clinical officers and 1 nurse. Preliminary results show an improvement of HCWs’ performance when managing infectious patients in their RHC. The final data are currently being analyzed.

**Conclusion:** A structured IPC-assessment of a RHC can be performed within hours, allowing IPCprofessionals to identify risks of pathogen transmission. A tailored training can help prepare RHC in Zambia for the management of infectious patients.

**References**

1) CDC, Version 2.3 – September 2016, https://www.cdc.gov/infectioncontrol/pdf/icar/outpatient.pdf

**Disclosure of Interest**: None declared

## Poster session: Long-term care facilities & nursing homes

### P203 ANTIMICROBIAL USE AND URINARY TRACT INFECTIONS IN FINNISH LONG-TERM CARE FACILITIES: RESULTS FROM TWO DIFFERENT SURVEILLANCE SYSTEMS

#### S. Toura, D. Arifulla, E. Sarvikivi, M. Mäkelä, O. Lyytikäinen

##### National Institute for Health and Welfare, Helsinki, Finland

###### **Correspondence:** S. Toura

**Introduction:** Prevalence of healthcare-associated infections (HAIs) and antimicrobial use (AMU) in Finnish long-term care facilities (LTCFs) were investigated recently as a part of the third European point prevalence survey (HALT) coordinated by European Centre for Disease Prevention and Control. In Finland, the RAI Long Term Care (LTC) and Home Care Instruments have previously been considered feasible tools for collecting AMU and HAI data from LTCF residents.

**Objectives:** We explored the prevalences of AMU and HAIs in Finnish LTCFs and compared the results in two different surveillance systems.

**Methods:** In Finland, HALT survey was conducted during September-November 2017, gathering information on resident characteristics, active HAIs and AMU on the day of the survey. HAIs had to meet the criteria of the standardized case definitions. Second, we used data on characteristics, AMU and urinary tract infections (UTI) of all residents for whom RAI-LTC form was completed by LTCFs during October 2017-March 2018. RAI collected AMU within 7 days prior to the assessment and infection data was based on a checklist. Descriptive statistics were used to summarize and to compare the data.

**Results:** In total, HALT survey covered 175 LTCFs with 6762 residents and RAI 1369 LTCFs with 21 943 residents. The proportion of residents over 85 years of age, females and usage of urinary catheter did not differ between the two surveillance systems. In HALT and RAI, 5% and 6% of the residents received at least one antimicrobial (other than methenamine) and the proportion of residents using methenamine was 2% and 3%, respectively. In HALT the most common infection type was UTI. UTI prevalence was higher in RAI than in HALT (5% vs. 1%).

**Conclusion:** Although the data collection methods differed, the two surveillance systems provided rather similar estimates for AMU. AMU and UTI prevalences were lower than in the previous study which was also based on RAI data. RAI-LTC could be used as an alternative tool for data collection on AMU and HAI in LTCFs. However, adding or implementing case definitions/or implementing case definitions for HAIs would improve the data.

**Disclosure of Interest**: None declared

### P204 Withdrawn

### P205 SCABIES IN NURSING HOMES : EXPERIENCES IN CANTON DE VAUD, SWITZERLAND?

#### D. Hequet, B. Sobgoui, C. Petignat

##### Unité cantonale vaudoise HPCI, Lausanne, Switzerland

###### **Correspondence:** B. Sobgoui

**Introduction:** Cases of scabies in nursing homes (NH) can be problematic to manage, mainly because diagnosing scabies is difficult. Indeed, the elderly can be paucisymptomatic and the identification of the parasite is complex and requires expertise. Because diagnosis is often delayed, additional measures are not immediately implemented.

**Objectives:** The main purpose of this review is to demonstrate the importance of rapid identification and management of cases and contacts

**Methods:** We reviewed scabies cases reported in four NH between June 2017 and March 2019.

**Results:** Among residents, sixteen cases were identified with three close contact cases. Moreover, one case engendered two contact cases in healthcare workers. A treatment was given to the sixteen cases, the three close contacts among residents and the two healthcare workers. We describe the special situation of a NH with seventy-two residents on four floors and two healthcare workers teams (floors 1-2 and floors 3-4). In December 2018, a case of Norwegian scabies in a hospital was confirmed. The case and two healthcare workers as secondary cases were diagnosed and treated accurately. All the residents and healthcare workers of the same floor (second floor) received a treatment. In January 2019, three residents (to the first and second floor) were diagnosed with Norwegian scabies; none contact case was identified. Unfortunately, the treatment of the forty-four contact residents was not concomitant (time differential from 1 to 5 days due to the availability of drugs). In March 2019, four cases were diagnosed (first and third floor), without reported contact cases. The treatment was once again given to all the forty-four contact residents and to all the healthcare workers of the institution. Within three months, all residents and healthcare workers had to take the treatment at least two times, which represents more than 150 treated people.

**Conclusion:** This analysis demonstrates the importance of applying the PCI recommendations (identifying contact cases and the concomitant treatment of residents and healthcare workers) are essential to eradicate scabies in NH, in particular in case of Norwegian scabies.

**Disclosure of Interest**: None declared

### P206 FIRST NATIONAL PREVALENCE STUDY OF HEALTHCARE-ASSOCIATED INFECTIONS AND ANTIBIOTIC USE IN SERBIAN LONG-TERM CARE FACILITIES

#### Z. Djordjevic^1^, G. Dragovac^2^, G. Krtinic^3^, I. Janicijevic^4^, V. Rakic^5^, L. Markovic-Denic^6^

##### ^1^Clinical Center of Kragujevac, Kragujevac; ^2^University of Novi Sad, Faculty of Medicine, Novi Sad; ^3^General Hospital, Subotica; ^4^Institute of Public Health, Nis; ^5^Institute of Public Health of Serbia; ^6^University of Belgrade, Faculty of Medicine, Belgrade, Serbia

###### **Correspondence:** L. Markovic-Denic

**Introduction:** Although less invasive medical care is provided in long-term care facilities (LTCF) than in hospitals, healthcare-associated infections (HAIs) and antimicrobial (AM) use are common in residents of these institutions.

**Objectives:** To identify institution and resident associated risk factors of HAI and AM use.

**Methods:** Six LTCF from different regions in Serbia were included in a 1-day point prevalence survey in July 2017, applying ECDC definitions and methods. Data collecting staff were previously trained physicians and infection control (IC) nurses from LTCFs and IC doctors from the regional institutes of public health. Data on LTCF organization and resources, HAIs, and AM use were collected.

**Results:** A total of 1168 eligible LTCF full-time residents were included (male-female ratio was 0.40; 32.1% >85 yrs). The prevalence of residents with at least one HAI was 3.2% (95% CI 1.5-4.6), and prevalence of all HAI was 3.5% (95% CI 1.6-5.4). Urinary tract infections and respiratory tract infection were more frequent with prevalence 1.5% and 1.0%, respectively, following with skin infections, than the eye, ear, nose, mouth infections and unexplained fever (prevalence 0.3% vs. 0.2% vs. 0.1%, respectively). At higher risk of HAI were residents with an indwelling urinary catheter (OR=5. 949, 95%CI), pressure sores (OR=14.208, 95% CI 5.838-34.577; p<0.001), impaired mobility (OR=3.395, 95%CI 1.518-7.591; p=0.003), and disorientation in time and/or space (OR=4.288 95%CI 2.057-8.938; p<0.001). At least one antimicrobial received 4.9% (95%CI 3.7-6.3) of residents; prevalence of total AMs was 6.0% (95%CI 3.4-15.4). AMs were mainly administered orally (77%). Most frequent AM were quinolones (30.5%), cephalosporins, carbapenems (22.0%) and aminoglycosides (16.9%).

**Conclusion:** The prevalence of HAIs and AM use at LTCF residents in Serbia were similar to LTCF residents in EU.

**Disclosure of Interest**: None declared

### P207 INFECTION CONTROL MEASURES IN BAVARIAN LONG-TERM CARE FACILITIES – A SURVEY

#### T. Kolberg^1^, C. Naß^1^, S. Fries^1^, G. Todorovic^1^, G. Valenza^2^, V. Lehner-Reindl^2^, A. Zeckey^1^, N. Frank^1^, C. Höller^1^

##### ^1^Hygiene, BAVARIAN HEALTH AND FOOD SAFETY AUTHORITY, Oberschleißheim; ^2^Hygiene, BAVARIAN HEALTH AND FOOD SAFETY AUTHORITY, Erlangen, Germany

###### **Correspondence:** N. Frank

**Introduction:** Infection control is becoming more and more important in long-term care facilities (LTCF) like nursing homes and residential homes. This is not only due to the demographic development and the associated changes in the health situation of the population, but also because changes in hospital care with earlier discharge of patients, increasingly complex treatments as well as the emergence of antibiotic-resistant bacteria outside of hospitals lead to higher demands on infection control in these facilities.

**Objectives:** In order to evaluate the infection control measures in Bavarian LTCFs and to derive possible necessary actions a questionnaire was developed and applied in 40 LTCFs, which were inspected by the project-team.

**Methods:** Considering number of rooms, rural or urban surroundings, private or public ownership the selection of LTCFs was representative for institutions that can be found in Bavaria.

**Results:** Infection control staff with a corresponding comprehensive training could be contacted by 73% of LTCFs, 9% had someone even available in-house. The local staff had a rather limited training in infection control and felt often insecure in special situations like management of residents colonized with antibiotic-resistant bacteria. 20% of the LTCFs had no standard to deal with an outbreak situation, for instance caused by norovirus or influenza virus. In 30% of the facilities laundry from infectious residents was not disinfected and only 52% stored the laundry in an adequate way. Storage areas were often not sufficiently available so that work according to infection control rules was impeded. More than half of the LTCFs had only a water-pressure-dependent decentralized disinfectant dosing unit, which may cause problems and was therefore considered as unreliable.

Subsequent to our survey a questionnaire asking about risk factors and more details about infection control training was distributed to all 1.500 LTCFs in Bavaria in order to evaluate if a common training course has to be established. In addition, educational films are currently being produced that will be made available to all LTCFs free of charge.

**Conclusion:** Infection control is an important issue. Although the staff was involved, it became clear that more needs to be done in this area.

**Disclosure of Interest**: None declared

### P208 UNIFORM PREFERENCE FOR HEALTHCARE WORKERS BY RESIDENTS AND HEALTHCARE WORKERS IN NURSING HOMES

#### N. Kenters^1^, A. Eikelenboom^1^, M. de Leeuw^1^, K. Saris^1^, M. Hulscher^2^, A. Huis^2^, A. Voss^1,3^

##### ^1^Medical Microbiology, CWZ; ^2^IQ Healthcare; ^3^Medical Microbiology, Radboudumc, Nijmegen, Netherlands

###### **Correspondence:** A. Eikelenboom

**Introduction:** In Dutch nursing homes it is found of great importance that residents feel at home. Therefore, white uniforms or service clothes for the healthcare workers (HCWs) have been abolished in most nursing homes. The choice for uniforms or private clothing lies solely with the organization. Private clothing for infection control measures are far from ideal to prevent transmission of microorganisms. No research has yet described which care-givers attire is preferred by residents in nursing homes.

**Objectives:** The aim of the study was to research the preference for HCWs clothing during care giving by HCWs and residents in nursing homes.

**Methods:** A quasi-experimental cross-sectional study was conducted in which two propositions regarding the clothing of health care personnel are presented to nursing home residents and HCWs. In total 20 residents and 13 HCWs were questioned in an intramural somatic care organization. Other nursing homes will follow in the upcoming months.

Two questions were asked, on preference by care provided, and on were residents/HCWs felt most comfortable with. The nursing home resident / HCWs had to choose between 4 different clothing outfits with the test – retest principle. The choices were casual, only a professional polo, only a professional nursing jacket and a complete white uniform.

**Results:** The primary outcome measure of the study is the preference of residents / HCWs for a specific clothing combination. Overall 38% had a preference for a professional nursing jacket with their own trousers. With a difference of 10% between residents and HCWs. In total the casual outfit was chosen the least, 9% of the times, with a minor difference of 3% between the HCWs and residents.

**Conclusion:** Most of the HCWs and residents in the nursing home both choose for a HCWs to dress in a professional outfit. The least chosen outfit was the casual outfit. With a professional outfit the chance of transmission of microorganisms via clothes of HCWs would be reduced.

Results of the additional nursing homes will be included in the scientific poster.

**Disclosure of Interest**: None declared

### P209 INFECTION PREVENTION IN NURSING HOMES: A QUALITATIVE STUDY OF FAMILY PRACTITIONERS’ AND RESIDENTS’ SUBJECTIVE NORMS, ATTITUDES AND BEHAVIORS WHILE VISITS

#### J. Hammerschmidt, L. Heier, N. Ernstmann

##### Institute for Patient Safety , University Hospital Bonn, Bonn, Germany

###### **Correspondence:** J. Hammerschmidt

**Introduction:** The post-antibiotic era poses increasing economic and ethical challenges for society.^1^ 426. 277 cases of infections with multi drug resistant (MDR) bacteria in health care were registered in the EU.^2^ The role of general practitioners (GPs) in the treatment of nursing home residents in counselling to prevent infections is not sufficiently understood.^3^ Although there are guidelines for hand hygiene in German nursing homes, for GPs, it is still unclear to what extent these are implemented, and if so, whether they are communicated to the residents. ^4^

**Objectives:** The aim of the study is to gain a first understanding of the perspectives and attitudes of residents and visiting GPs towards counselling health literacy regarding the prevention of MDR infections.

**Methods:** Qualitative semi-structured interviews with 12 GPs and thematically focused conversations with 12 nursing home residents (cognitively not impaired) were conducted and content analysed.

**Results:** GPs (Øage 44, 3 fem.) reported deviant behaviour in consultation from the guidelines. Nursing home residents are rarely given advice on improving their health literacy. They (Øage 84, 10 fem.) reported that GPs do not perform this advisory function, although many residents have already had experiences with MDR.

**Conclusion:** Health literacy in infection prevention in nursing homes during GPs visits has hardly been researched to date. The research is mainly focused on results and recommendations for existing infections.

**References**

1) **Tacconelli, E.; Pezzani, M. D.** Public health burden of antimicrobial resistance in Europe, The Lancet Infectious Diseases. (2018), DOI:10.1016/S1473-3099(18)30648-0.

2) **Cassini, A. et. al**, Attributable deaths and disability-adjusted life-years caused by infections with antibiotic-resistant bacteria in the EU and the European Economic Area in 2015: A population-level modelling analysis. The Lancet Infectious Diseases. (2018), DOI: 10.1016/S1473-3099(18)30605-4.

3) **Nicolle, L. E.** Infection prevention issues in long-term care, Current opinion in infectious diseases. (2014), DOI:10.1097/QCO.0000000000000071.

4) **Fassbender B., Rösing C., Weckbecker K.** MRSA-eine Handreichung für Hausärzte Teil 1-3: Altenpflegeheime AWMF-Registernr. 053/034c Klasse S1; DEGAM, 2013.

**Disclosure of Interest**: None declared

### P210 RESPIRATORY SYNCYTIAL VIRUS, A THREAT FOR NURSING HOMES RESIDENTS?

#### D. Hequet, A. Rochat, C. Petignat

##### Unité cantonale vaudoise HPCI, Lausanne, Switzerland

###### **Correspondence:** A. Rochat

**Introduction:** Nursing home residents live confined, in close contact with one another and staff, increasing opportunities for viral spread. During winter season, Respiratory Syncytial Virus (RSV) might affect the residents as much as influenza virus. However, data estimating RSV morbidity and mortality in nursing homes remain scarce.

**Objectives:** Describe epidemiology of RSV in nursing homes during influenza season

**Methods:** In influenza seasons 2016-2017 and 2017-2018, a study on the burden of influenza was led in nursing homes of canton de Vaud. Nasal swabs were collected in residents symptomatic with an influenza-like illness. The samples were analyzed with a coupled PCR diagnosing influenza A/B as well as RSV. As a result, the diagnosis of RSV was underlined, even though it was not the purpose of the study.Demographic characteristics of resident were recorded (clinical data, hospitalization, mortality).

**Results:** 509 residents with influenza-like illness (ILI) symptoms were included. 38 RSV infected residents (7.5%) were diagnosed in 15 different nursing homes. 10 of these nursing homes experienced an epidemic situation with ≥2 residents diagnosed and 2 of them with 5 and 6 simultaneous cases respectively. Median age was 87 years (SD 7). Median temperature at diagnosis was 37.9°C (SD 1.1). The three most represented symptoms were cough (89.5%), malaise (73.7%) and fever (71.1%). 12 residents (31.2%) required oxygen therapy and 26 residents (68.4%) were treated with an antibiotic. 3 residents were hospitalized within 30 days after diagnosis (7.9%). 8 residents died within the first 30 days (21%) and a total of 14 residents died within 90 days (37%).

**Conclusion:** This study revealed that RSV infections in the institutionalized elderly is an underestimated threat. Residents with a RSV infection encounter a risk for hospitalization and mortality. Although the study was conducted on a relatively small number of residents, it reveals the need to take into account this pathogen in case of influenza-like illness outbreak in nursing home. Moreover, RSV outbreaks can occur in nursing homes. In case of influenza-like illness epidemic in a nursing home, if samples are negative for influenza, RSV should be looked for.

**Disclosure of Interest**: None declared

## Poster session: Infection Control & Prevention in developing world

### P211 Withdrawn

### P212 Withdrawn

### P213 BEHAVIORAL AND ENVIRONMENTAL RISK FACTORS ASSOCIATED WITH NEONATAL SEPSIS IN UGANDAN HEALTHCARE FACILITIES

#### H. Yakubu on behalf of Center for Global Safe WASH, Emory University

##### Center for Global Safe Water, Sanitation and Hygiene, Hubert Department of Global Health, EMORY UNIVERSITY, Cantonments, Ghana

###### **Correspondence:** H. Yakubu

**Introduction:** Neonates’ exposure to pathogens in the healthcare facility environment can cause healthcare associated infections including sepsis. Sepsis is estimated to be responsible for 15%>20% of neonatal deaths globally and about 30%>40% occur at the time of birth. Low resource setting are disproportionally affected by the burden of neonatal deaths. In Uganda, new born deaths constitute 38% of the all infant mortality and an estimated 31% of new born deaths are attributed to sepsis. However, little evidence exists on behavioral and environmental risk factors that cause sepsis

**Objectives:** The objective of this study is to assess environmental and behavioral risk factors associated with neonatal sepsis in maternal and post natal wards

**Methods:** Two public healthcare facilities (HCF) in Kampala, Uganda with contrasting differences in water, sanitation and hygiene (WASH) conditions and infrastructure were purposively selected. They were classified as WASH poor and WASH good HCF. A qualitative approach was adopted to understand what patients, caregivers and health care staff come into daily contact with that may lead to neonatal exposure to pathogens in the health facility environment. Structured and unstructured observation were conducted to understand who frequently come in touch with neonates and their frequency of contact with surfaces and equipment’s to inform environmental sampling and exposure pathways. Structured observation was subsequently conducted using the checklist for 3 hours each in the morning, afternoon and evening for 2 weeks by trained enumerators

**Results:** In the post natal wards, bedsheets (26%) and the walls (21%) were the most frequently touched in the WASH good HCF whilst in the WASH poor HCF, the most frequently touched were mobile phones (10%) and delivery kits (9%). In the delivery and labor ward, fetal scope (16%) and bedrail (13%) were the most frequently touched in the WASH good HCF whilst in sink faucet (7%) and bed lining (6%) were the most frequently touched in the WASH poor HCF

**Conclusion:** This information was used to target environmental sampling to determine pathways of exposure that may have the greatest risk to neonates. Analysis of the sampling results in conjunction with the behaviors observed will be useful in planning evidence based interventions in the healthcare facilities.

**Disclosure of Interest**: None declared

### P214 PROFILE OF PSEUDOMONAS INFECTIONS DIAGNOSED IN AN INFECTIOUS CLINIC IN DAKAR (SENEGAL)

#### N. A. LAKHE, K. DIALLO MBAYE, A. MASSALY, V. M. P. CISSE DIALLO, N. M. FALL, D. THIOUB, A. BADIANE, D. KA, L. FORTES DEGUENONVO, C. T. NDOUR, M. SEYDI

##### SERVICE DES MALADIES INFECTIEUSES/ CENTRE HOSPITALIER DE FANN, DAKAR, Senegal

###### **Correspondence:** N. A. LAKHE

**Introduction:** *Pseudomonas* infections are opportunistic infections marked in some situations by their great severity and their nosocomial character. The resistance to carbapenems is actually a great issue by reducing therapeutic options.

**Objectives:** The objectives of this study were to determine the epidemiological, clinical, resistance and therapeutic profile of *Pseudomonas* infections.

**Methods:** A descriptive retrospective study was performed, based on hospitalized patients’ records in the department of infectious and tropical diseases, for whom Pseudomonas infections was diagnosed from 1 January 2013 to 31 December 2017.

**Results:** Forty-three cases (2.1%) of Pseudomonas infections were collected among 2,061 inpatients. The sex ratio was 0.95. The mean age was 43 ± 16. Nearly half of the patients (21 cases) had HIV infection. Bacteremia and urinary tract infections were the most common with 18 cases, followed by lower respiratory tract infections (5 cases) and skin and soft tissues (4 cases). The isolated strains were *Pseudomonas aeruginosa* (68.8%), *Pseudomonas spp* (28.8%) and one strain for *Pseudomonas oryzyhabitans* (2.2%). Antibiotic resistance was higher for Aztreonam, piperacillin-tazobactam and fosfomycin with 19.4%, 12.9% and 9.7% respectively. One strain producing carbapenemase (3.2%) and another one resistant to colistin (3.2%) were isolated. Also, only one strain was resistant to ciprofloxacin. All strains tested were sensitive to amikacin. More than 6 out of 10 patients (61.2%) had received probabilistic antibiotic therapy. The lethality was 30.2%.

**Conclusion:** *Pseudomonas* infections are common in our context and have high mortality. They pose the problem of their management with the appearance *Pseudomonas* to imipenem and colistin. Strategies for rational use of antibiotics must be implemented.

**Disclosure of Interest**: None declared

### P215 PREVALENCE AND EPIDEMIOLOGICAL PROFILE OF HEPATITIS B VIRAL INFECTION AMONG PREGNANT WOMEN IN TAIF GOVERNORATE, SAUDI ARABIA

#### N. M. Alzahrani^1^, F. S. Alghamdi^2^ , Y. H. Alam-Eldin^3,4^ , M. Bahaa Eldin Foda^5^

##### ^1^Communicable Diseases & Vector Control Administration, Ministry of Health, Taif, KSA; ^2^Blood Banks Administration, Ministry of Health, Taif, KSA

###### **Correspondence:** N. M. Alzahrani

**Introduction:** Hepatitis B virus (HBV) infection is the most predominant type of hepatitis infection in Saudi Arabia. HBV-infected mothers are important source of infection in children.

**Objectives:** This study aims to estimate the magnitude of HBV infection and to identify the most important risk factors among the pregnant female in Taif city, Saudi Arabia.

**Methods:** We performed a cross-sectional study on Taif governorate from January 2018 to January 2019. Blood samples were collected from pregnant women who followed up for antenatal care clinics at primary healthcare centers (PHC) and hospitals of Taif. Samples were processed by Enzygnost® Hepatitis B surface Antigen (HBsAg) 6.0 enzyme immunoassay for qualitative detection of HBsAg. Positive results were confirmed by neutralization of HBsAg and Hepatitis B core Antibody (HBcAb).

**Results:** The seroprevalence of positive HBsAg among 11370 pregnant females was 0.88%. The age range of seropositive pregnant females was from 20 to 49 years (yrs) with an average of 34 + 4.86; 10% from 20-29 yrs, 79% from 30-39 yrs and 11% from 40-49 yrs. Seroprevalence in rural areas was 61% and 39% in urban areas. 47% of seropositive cases were diagnosed during antenatal screening, 27% during premarriage screening, and 8% during peripartum screening. 5% of the seropositive pregnant females were vaccinated and 65% were unaware about their vaccination status. The most important risk factors of HBV infection in this study were attributed to history of dental procedures 36.8% and contact with infected-family member 32.9%.

**Conclusion:** The seroprevalence of HBV infection among the pregnant females in Taif was lower than other regions of KSA, reflects the success of the HBV vaccination program in Taif. Consequently, awareness about importance of antenatal screening and routes of transmission of HBV infection especially in rural areas should be raised.

**Disclosure of Interest**: None declared

### P216 KNOWLEDGE OF HEALTHCARE ASSOCIATED INFECTIONS AND RISKS FACTORS IN THE INFECTIOUS DISEASES SERVICE IN FANN HOSPITAL (DAKAR-SENEGAL)

#### N. A. Lakhe, B. A. Niang, K. Diallo Mbaye, D. Ka, A. Massaly, V. M. P. Cisse Diallo, L. Fortes Deguenonvo, C. T. Ndour, M. Seydi

##### SERVICE DES MALADIES INFECTIEUSES/ CENTRE HOSPITALIER DE FANN, DAKAR, Senegal

###### **Correspondence:** N. A. Lakhe

**Introduction:** Healthcare-associated infections are a major public health concern. These infections are marked by an increase frequency, their gravity, not to mention the medico-legal aspect raised, as well as the economic increase generated.

**Objectives:** The main objective was to assess the state of knowledge of medical and paramedical personnel on health care-associated infections and their risk factors.

**Methods:** A cross-sectional and observational survey was conducted from 12 to 14 December 2018 among staff of the infectious diseases department of CHNU Fann. A pre-established questionnaire was administered to the participants by direct interview. The questions were mostly closed.

**Results:** A total of 76 people was surveyed during this study. Two-thirds were women and the median age of the study population was 31 [19; 64]. As for their qualification, half of the respondents were nurses, 19.7% doctors and 13.2% trainees. Almost 6 out of 10 (59.2%) had less than 5 years of seniority in the service. Only 35.5% of respondents were trained in infection prevention and control (IPC). A correct definition of HAI’s was provided by 49 participants (64.5%). Three-quarters of the participants were not knowledgeable about hygiene’s standard precautions and 9 of 10 about additional hygiene precautions. More than half of the respondents (51.3%) were unable to cite a HAI’s. The main factors favoring HAI’s identified by the respondents were urinary catheter (98.7%), vascular catheter (94.7%), insufficient surgical asepsis (93.4%) and nasotracheal aspiration (81.6%).

**Conclusion:** Overall knowledge of healthcare-associated infections and their contributing factors is insufficient. Regular training program of health personnel with a well-established schedule should be undertaken.

**Disclosure of Interest**: None declared

### P217 SMALL IS BEAUTIFUL: HOW A TINY NGO BROUGHT ABOUT A BIG CHANGE IN INFECTION PREVENTION AT BIRTH

#### C. Kilpatrick^1^, G. Gon^2^, E. Morrison^1^, S. Woodd^2^, S. Virgo^2^, W. Graham^2^

##### ^1^The Soapbox Collobrative, Aberdeen; ^2^London School of Hygiene and Tropical Medicine, London, United Kingdom

###### **Correspondence:** C. Kilpatrick

**Introduction:** Launched in 2012, The Soapbox Collaborative’s primary goal was to inform and influence action by policy-makers, managers and the healthcare workforce to reduce the recently reported global burden of healthcare associated infections (HAI), particularly in mothers and newborns in hospitals in low and middle-income countries (LMIC).

**Objectives:** To outline how a small NGO can affect change in knowledge and behaviours towards infection prevention and control (IPC).

**Methods:** Between 2013 and 2017, needs assessments were undertaken with country partners in eight LMICs. A mixed methods approach using validated tools assessed the state and drivers of hygiene, measured by visual cleanliness inspection, surface microbiology to detect potential pathogens, hand hygiene audits, document reviews, structured interviews and focus group discussions.

**Results:** Inadequate training and supervision, insufficient resources and poor infrastructure were found to be limiting facility cleaners from maintaining a safe and hygienic health care environment. Soapbox's participatory training package (TEACH CLEAN) was developed to address this gap, and first pre-tested in The Gambia. It has since been acknowledged by major international agencies, and applied in several countries, including Cameroon, Myanmar and the United Republic of Tanzania. Recent inclusion of indicators in global best practice and monitoring platforms has resulted in TEACH CLEAN being recognised as a ready resource. Increasing concern of the antimicrobial resistance burden and the need to improve water, sanitaion and hygiene in healthcare facilities has meant that this NGO's novel contribution is informing action to prevent HAI in maternity units and exceeding reasonable expectations that it can play a crucial influential role in global and country partner work.

**Conclusion:** The role of NGOs in supporting global IPC initiatives is often not dependent on size of the organization but rather the timeliness of its activities and the opportunity to align with local and global partners. The Soapbox Collaborative has highlighted the neglected role of cleaners and cleaning in the prevention of HAI in maternity units in LMICs and provided a sustainable training package, accepted at global and nationla level, to improve hygiene practices which will result in an impact on HAI.

**Disclosure of Interest**: None declared

### P218 REGISTER OF INFECTION PREVENTION AFTER PROFESSIONAL ACCIDENTAL EXPOSURE TO BIOLOGICAL AGENTS AND SEXUAL ABUSE IN THE REGIONAL PUBLIC HOSPITAL OF BANFORA (BURKINA FASO)

#### K. C. C. Sawadogo^1^, I. F. BAKO^2,3^

##### ^1^Medical information, Public regional hospital, Banfora; ^2^Health science department, Joseph KI-ZERBO University; ^3^Neurosurgery, Yalgado OUEDRAOGO University hospital, Ouagadougou, Burkina Faso

###### **Correspondence:** K. C. C. Sawadogo

**Introduction:** Post-exposure prophylaxis (PEP) relies on procedures allowing quick access to treatment in case of accidental exposure to viral risk (AEV). There are 2 types of AEVs:

- occupational blood exposure concerns mainly health-care professionals
- sexual exposure for which there is currently no monitoring [1].

The register of the hospital of Banfora has been created since 2013 in order to collect these data.

**Objectives:** The main objective was to evaluate the use of the paper register for the prevention of accidental exposure to infections.

Secondary objectives were:

- to determine the types of cases notified in the register;

- to determine the occupational profiles most at risk of infectious accidents;

- to determine the most implicated material in infectious accidents.

**Methods:** A retrospective study had been conducted from January 1^st^ to April 1^st^ 2019 on the paper-based registry of infections exposures in the Cascades region (Burkina Faso). An exhaustive inclusion had been made and paper questionnaires were completed. The study was approved by the hospital administration. Data were analyzed using EPI INFO 3.5.3.

**Results:** From 2013 to May, 1^st^ 2019, 39 cases of AEV have been reported, including 34 cases of exposure accident to biological fluid, four cases of sexual assault and one case of condom rupture. The average age of our patients was 30.57 years. Nurses and biomedical technicians are the most affected occupational profiles, with 35.9% and 23.1% of notified cases respectively. Care and handling constituted the majority of the accident occurrences, with 68.6% and 17.1% of the notified cases respectively. The syringe needle was the most implicated material with 67.6% of cases. Sexual assault involved women whose average age was 19. No patients had reported positive HIV and hepatitis markers.

**Conclusion:** In 6 years of using the register, it is clear that it is under notified. It is appropriate to make health staff and the population of the Cascades region aware of the need to consult for adequate management in the event of an accident of exposure.

**References**

1. Rouveix, E., Bouvet, E., Vernat, F. et al. (2014). Prise en charge des expositions accidentelles au VIH : rapport d’activité 2011 des COREVIH, Médecine et maladies infectieuses, 44, 112– 16.

**Disclosure of Interest**: None declared

### P219 ABOUT DIFFICULTIES TO MAKE SUSTAINABLE IPC PROJECTS IN AFRICA: EXAMPLE OF FANN HOSPITAL IN SENEGAL

#### B. Ndoye^1^, N. A. LAKHE^2^, C. T. Diop^3^

##### ^1^ICAN, WHO Consultant; ^2^Fann Hospital,IPC Coordinator; ^3^Fann Hospital,General Director, Dakar, Senegal

###### **Correspondence:** B. Ndoye

**Introduction:** Healthcare-associated infections (HAIs) are currently a global public health problem. However, there are very few concrete and effective actions to sustainably improve the situation in African hospitals. We report here the example of the Fann hospital in Senegal where an initiative was taken in this direction

**Objectives:** After many previous attempts to improve current IPC program in situation of lethargy, the objective was to provide the hospital with a functional IPC committee and to develop a concrete and sustainable action plan, adapted to local needs.

**Methods:** The methodological approach was very inclusive, with the participation of all stakeholders, both technical services and the administration. The following activities were carried out with the technical and financial support of partners (WHO and CDC):

- Information and awareness meeting chaired by the Hospital General Director to inform all staff

- Evaluation of the PCI situation using WHO's IPCAF tool [1], including an analysis of strengths and weaknesses, but also opportunities and threats

- Inclusive and participatory development of an operational action plan based on core components recommended by WHO, and a follow-up plan on the activities to be carried out

**Results:** A concrete and realistic operational action plan was available since July 2018, but no planned activity has been implemented so far, one year after.The hospital is waiting for potential partners to fund the activities.

**Conclusion:** This IPC project, as many of others, stops at the withdrawal of partners, and goals were not achieved. The causes are undoubtedly numerous and intricate in the context of limited resources. In any case, at the hospital level, serious consultation between the technical services and the administration is needed, while at the national level it is time for African countries to make IPC a national priority for public health. This must result in the development of a national IPC policy validated by the authorities and implemented in concrete terms by a program equipped with the necessary means of action. The WHO-CDC Africa project to develop recommendations on minimum standards for IPC will certainly have to bring a new dimension to the fight against the scourge in the continent.

**References**

[1] http://www.who.int/infection-prevention/tools/core-components/en/

**Disclosure of Interest**: None declared

## Poster session: Occupational Health

### P220 OCCUPATIONAL RISK OF BLOOD-BORNE VIRUSES IN HEALTHCARE WORKERS: A 20-YEAR SURVEILLANCE

#### F. A. Van Laer^1^, E. Coenen^2^, H. Jansens^1^

##### ^1^Infection Control; ^2^Occupational Medicine Department, Antwerp University Hospital, Edegem, Belgium

###### **Correspondence:** F. A. Van Laer

**Introduction:** This study presents the results of a 20-years surveillance program involving the prospective follow-up of health-care workers (HCWs) in the Antwerp University Hospital exposed to blood-borne viruses (hepatitis C virus (HCV), hepatitis B virus (HBV) and human immunodeficiency virus (HIV)).

**Objectives:** To evaluate the risk of transmission of blood-borne viruses to health-care workers.

**Methods:** From 1998 through 2018, all HCWs reporting an occupational exposure (through sharp objects, bites, mucous membrane exposure or damaged skin) to blood-borne viruses were included in the surveillance. After the exposure a serum sample from the patient whose blood caused the contamination was tested for viral markers: HIV 1&2 As/Ag, HBsAg and anti-HCV. Only if one these markers was positive the HCW was also tested and a postexposure follow-up for six months was done. No postexposure follow-up was done for HBV of HCW with anti-HBs levels higher than 10 IU/ml. Postexposure prophylaxis for HIV (a co-formulation of elvitegravir, cobicistat , emtricitabine and tenofovir disoproxil fumarate) was offered to the HCW if the source was positive for HIV as recommended by the Belgian Superior Health Council. All HCWs with anti-HBs levels of less than 10 IU/mL were vaccinated against hepatitis B virus infection.

**Results:** A total of 2495 exposures were reported: 7 (0,3%) through bites, 290 (11,6%) through mucous membrane exposure, 2122 (85,1%) through needles and other sharp objects and 76 (3,0%) through damaged skin. Among the patients involved 105 (4,2%) were positive for HCV, 48 (1,9%) were positive for HBV, 125 (5,0%) were positive for HIV and 2217 (88,9%) were negative for all viral markers. During the serologic follow-up no transmission of HBV, HCV or HIV was observed among the HCW.

**Conclusion:** Our postexposure follow-up among HCW revealed a lack of transmission of HBV, HCV and HIV. These results are confirmed by other published surveillance programs.

**Disclosure of Interest**: None declared

### P221 DECREASING EXPOSURE OF HEALTH CARE WORKERS TO INFECTIOUS DISEASES: A STEP TOWARDS EMPLOYEE HEALTH AND SAFETY

#### R. Roshanali, N. K. Virani, Z. Rafique, R. Khowaja, S. F. Mahmood

##### Aga Khan University Hospital Karachi Pakistan, Aga Khan University Hospital Karachi Pakistan, Karachi, Pakistan

###### **Correspondence:** R. Roshanali

**Introduction:** Safety of employees should be one of the top most concern for any organization. Accidental exposure to any pathogens carries risk of infection and is responsible for transmission of fatal diseases. Health care workers (HCWs) occupational exposure to various communicable diseases must be an area of concern for all infection preventionists.

**Objectives:** The objectives of this study were as follows:

- To reduce health care workers exposure rate to infectious/communicable diseases atleast by 60% from 6.7 to to 2.5 in next one year.

- To decrease the cost of investigation incurred by the organization after an employee is exposed to infectious disease. To minimize risk of acquiring infectious diseases to the employees.

- To reduce the cost of investigation incurred by the organization after an employee is exposed to the infectious diseases

- To identify causes of noncompliance to institutional policies

**Methods:** Define Measure Analyze Improve Control (DMAIC) six sigma methodology of quality Improvement was utilize. Each step were done systematically, after defining the problem, data was measured and analyzed to target the interventions. In improve phase major actions were taken and implementation of each action was done on ground and finally controls were set in the systems to ensure sustainability of the project.

**Results:** Results showed significant reduction in the overall rate of exposure of health care workers to infectious diseases i.e. from 6.7 to 1.5 %. Particularly TB exposure decreased from 16 to 3.3, Measles from 3.6 to 2.4, Chickenpox from 10 to 3.6, CCHF from 3.8 to 0.3, Pertussis from 3.5 to 1.5 etc. Ultimately reduced the cost incurred by organization after employee exposure also decreased.

**Conclusion:** Health care workers exposure were decreased significantly, employees safety was optimized by minimizing the risk of acquiring infectious diseases. In addition the cost incurred post exposure for post exposure prophylaxis also decreased.

**References**

Steege, A. L., Boiano, J. M., & Sweeney, M. H. (2014). NIOSH health and safety practices survey of healthcare workers: training and awareness of employer safety procedures. American journal of industrial medicine, 57(6), 640-652.

**Disclosure of Interest**: None declared

### P222 REDUCING THE RISK OF SHARP INJURY AT TERTIARY CARE HOSPITAL (AKUH.K) A STEP TOWARDS HEALTH CARE WORKER SAFETY

#### Z. Rafique, R. Roshanali, N. Virani, R. Khowaja, S. F. Mahmood

##### Infection Prevention and Hospital Epidemiology, Aga Khan University and Hospital, Karachi, Pakistan

###### **Correspondence:** Z. Rafique

**Introduction:** Sharp injuries are a major occupational health and safety issues globally. Health care workers (HCW) are on highest risk of getting blood borne pathogen due to contaminated sharp objects.

**Objectives:** It has been observed that there was a sudden rise of sharp injuries in Q2 2017 from 59 to 72 and the NSI rates were high form benchmark i.e. >1.Thus Infection Control Department (IC) aimed to:

- To reduce number of exposure from sharp injuries i.e. from 72 to **60**
- To decrease sharp injuries rates and target was set below the bench mark >1
- Reduction in number of NSI exposures i.e. 50% in service line were rates are higher.

**Methods:** The PDSA model, a continuous quality improvement (CQI) tool, was used to decrease the preventable exposure to sharp injuries. P**lan**: AKUH’s IC department analyzed that number of sharp injuries among HCW has drastically increased in few service lines i.e. laboratory (0 to 11), GI and GS (05 to 16) and mind brain (01 to 06). **Do**: educational sessions were conducted for HCW through case based scenario activity, reinforcement was done to avoid recapping, flyers circulated to all patient care areas, disseminate pocket guide to HCW, fixed danger box in brackets to avoid spillage, introduce safe lancet to check blood glucose, explore retractable butterflies **Check**: Observed staff practices, malpractices were notified to supervisors. **Act:** Increase frequency of audits to ensure safe disposal of sharps and checked for overfilled danger box. Monthly sharing of exposure data with supervisors.

**Results:** A significant decline was observed in Q2 2018 after implementing all strategies. The number and rates of sharp injuries exposure decreased i.e. from 72 to 57 and 1.19 to 0.94 respectively. The number of NSI were decreased in the respective service line i.e. laboratory (11 to 06), GI and GS service line (16 to 18) and mind brain service line (06 to 02).

**Conclusion:** IC department continue to worked with individual department/service lines to decrease NSI and promote staff safety.

**Disclosure of Interest**: None declared

### P223 EVALUATION OF THE MEASLES, MUMPS, RUBELLA ANTIBODY IN ONE SOUTHERN HOSPITAL EMPLOYEE

#### H. Chao ^1^, C. Huang^2^, C. Tsou^3^, T. Chang^4^, H. Chung ^5^

##### ^1^Infection Control Unit, E-Da Cancer Hospital; ^2^Division of Infectious Diseases / Department of Medicine; ^3^Occupational Safety and Health office, E-Da Hospital; ^4^Occupational Safety and Health office; ^5^Division of Infectious Diseases / Department of Medicine, E-Da Cancer Hospital, Kaohsiung, Taiwan

###### **Correspondence:** H. Chao

**Introduction:** There are outbreak episodes of measles, mumps in Taiwan in recent years because of frequent International interactions, new immigrant groups, a decline of childhood vaccine efficacy in the 20-40 year old groups, et al. Similar outbreaks also occurred in different countries. The healthcare workers are at higher risk than the normal populations of acquiring diseases due to occupational exposure to measles, mumps and rubella (MMR) patients.

**Objectives:** we analyze the trend of seropositive prevalence within 2 years and in different occupations and age groups. This study gives a reference to develop a health policy in the future in this hospital.

**Methods:** MMR data in annual health checkups of healthcare workers in 2016 and 2017 is collected. Seropositive cases define all Immunoglobulin G(IgG) are positive in MMR. Seronegative cases defined anyone of MMR IgG is negative or low titer. We separate occupations to doctors, nurse, medical laboratory technicians and administrative assistant groups. Descriptive statistics and Chi-square test are used.

**Results:** In 2016, a total of 2,106 subjects(78.1%) received exams of MMR, and the seropositive rate was 42.3%. In 2017, a total of 2,440 subjects (88.4%) received exams of MMR, and the seropositive rate was up to 63.9% which contributed to 509 subjects (30%) received one booster vaccine in the prior year. The compared 2-year seropositive prevalence was significant (P<0.001).The seropositive rate of mumps and measles increased from 63.8%,61.7% to 80.7%,80.0% respectively.The 2-year prevalence comparison is significant (P<0.001). The seropositive rate of measles was 80.5% which was best in the nurse group with aged 20 to 40-year-old.

**Conclusion:** A policy of providing vaccines constructs a healthy and safe occupational environment and a preventive effect of outbreaks. After one dose of MMR vaccine in the seronegative group, a total seropositive rate was 63.9%. The seropositive rates of measles and mumps were both up to 80% and significant. The seropositive rate was up to 80% in the aged 20~40-year-old nurse group.

**References**

**Disclosure of Interest**: None declared

### P224 PREVALENCE AND PERCEPTION OF NEEDLE STICK INJURY AMONG HEALTH CARE PROFESSIONALS AT A TERTIARY CARE HOSPITAL, KARACHI, PAKISTAN

#### R. Nisar^,^ on behalf of Shahid Saleem ,Saima Bashir, Sunil Dodani, Asma Nasim

##### Infection control department, Sindh Institute of Urology and Transplantation, Karachi, Pakistan

###### **Correspondence:** R. Nisar

**Abstract video clip:** Introduction: Needle stick injury (NSIs) is a percutaneous injury occurs through sharp objects among health care professionals (HCP). Sharp injury can happen due to mishandling, recapping, passing of sharps or leaving the sharps on surfaces. World Health Organization (WHO) reported an average of 4 NSIs /year/HCP. Our study objective is to find the knowledge, prevalence of NSIs and current status of hepatitis B vaccination (HBV) among HCP.

Methods: A cross sectional survey conducted through questionnaire among HCP. Data collection was done between December 2018 and January 2019. Data analysis was done using SPSS version 20.

Results: A total of 117 participants were surveyed and majority was females. There were 23% doctors, 35% nurses and 46% technicians. Around 64% experienced NSIs in the last one year. Out of whom 8% got NSIs 3 times and 12% >4 times. Around 78% NSIs was unreported. NSIs were considered harmful by 97%. A total of 65% believe that HBV, Hepatitis C Virus (HCV) and Human Immunodeficiency Virus (HIV) can be transmitted by NSIs. Mostly the cause of NSIs was rush hours 33%. Around 47% NSIs were percutaneous and gloves were used by 79%. Only 12% washed their hands with soap and water after NSI. Around 77% HCP received health safety training. HBV vaccine series was completed in 71%, however, only 39% know about their antibodies titers.

Conclusion: NSIs has high prevalence in our setting. The reporting system, improvement in vaccination status, regular educational sessions with emphasis on standard precautions can decrease NSI rates in our hospital.

**Disclosure of Interest**: R. Nisar Employee of: Do not have any conflict of interest.

### P225 NEEDLE STICK INJURIES AMONG HEALTH CARE WORKERS IN A TERTIARY CARE TEACHING HOSPITAL IN PALESTINE, 2016-2018

#### S. Belkebir, A. Kanaan, R. Jeetawi

##### Preventive medicine, An Najah National University Hospital, 00000, Palestinian, State of

###### **Correspondence:** A. Kanaan

**Introduction:** Injuries among healthcare workers (HCW) from needles(NSI) and sharps remain a serious problem due to high risk of transmission of blood-borne pathogens (BBP)

**Objectives:** This study aims at reporting the numbers of NSIs from 2016 to 2018 and the main characteristics of affected HCWs at NNUH

**Methods:** An-Najah National University hospital(NNUH), a tertiary care hospital located in the north of Palestine, implemented since 2016,a strategy to prevent and surveille NSI among HCWs including the enforcement of evidence based policy, annual training about safety injection, waste management and universal precautions. Incidents are self-reported after what HCWs and the source are screened for BBP and appropriate management (Counseling, education, dose of HBV vaccine or Specific Ig) is applied according to setting policy and in collaboration with the Palestinian Ministry of health (MoH)who provides the HBV vaccine.In 2018, vacutainers were provided and intensive training and education were applied across the institution.

**Results:** The total number of NSI reported was 139(29,62 and 48 cases for 2016,2017 and 2018 respectively). 56%(78) were male and 44%(61) were female. The mean age was 26.5years (SD 7.4).All HCWs were VHC and HIV non-reactive. Nurses had the highest percentages 51.1%(71) of NSIs, followed by students 23%(32),doctors 12.2%(17), Cleaners 11.5%(16) and others such as lab technicians 2.2%(3).The most common mode of injury was during blood sampling 32.4% (45), followed by injuries at the moment of needle disposal 20.1%(28) , cleaning or waste disposal 10.8%(15), cannula insertion/removal 9.4%(13), glucose level testing 7.9%(11) and accidentally by other co-worker 4.3%(6).Recapping was the cause in only 2.2%(3). Regarding the management, 70.5%(98) did not need post-exposure prophylaxis (PEP), 28.1%(39) were referred to MoH to receive a single dose of VHB vaccine and only 1.4%(2) needed the specific Immunoglobulin+ vaccine.PEP for HIV is not available.

**Conclusion:** HCWs at NNUH are at great risk of contracting blood-borne infections. It is crucial then reinforce both the reporting system and the training program and the provision of PEP and adequate equipment.

**Disclosure of Interest**: None declared

### P226 OCCUPATIONAL EXPOSURE TO BLOOD AND BODY FLUIDS AMONG HEALTHCARE WORKERS AT MARMARA UNIVERSITY EDUCATIONAL AND RESEARCH HOSPITAL IN ISTANBUL, TURKEY.

#### D. N. M. Al-Khalili^1^, A. A. I. Muhsen^1^, M. Karavuş^2^, N. S. Al-Kayyali^3^

##### ^1^Medicine, Marmara University, Amman, Jordan; ^2^Medicine, Marmara University, Istanbul, Turkey; ^3^Actuarial Science, King Fahd University of Petroleum & Minerals, Amman, Jordan

###### **Correspondence:** A. A. I. Muhsen

**Introduction:** While healthcare workers are focused on tending to their patients, they become vulnerable to occupational exposure to blood and body fluids in the workplace. This poses and puts them in high risk of attaining blood-borne pathogens including Hepatitis B and HIV.

**Objectives:** The aim of the study was to investigate the exposure, awareness, and practices of healthcare workers related to occupational exposure to blood and/or body fluids in a teaching hospital in Istanbul, Turkey.

**Methods:** The study was cross-sectional in design. The residents and nurses (n=384) of the hospital were the target population of this study. Data were collected anonymously using a predesigned self-administered structured questionnaire consisting of 23 questions pertaining to different categories including demographic characteristics, awareness and precautions used by HCWs, frequency and exposure routes to blood and body fluids.

**Results:** The participants comprised 48.2% residents and 51.8% nurses. 86.7% had encountered an occupational exposure. Needle-stick injury was the most common mode of occupational exposure being 72.4% of total exposures. On average, residents generally encounter a needle-stick injury (NSI) at least 2.30 times per year, while nurses on the other hand, encounter a total of 2.67 needle-stick injuries per year. Residents tend to encounter a NSI the most “while handling contaminated needles” 73.8%, (*P*<0.018); in contrast to nurses, who were found to be exposed to a NSI more often “before using the needle” 54.6%, (*P*<0.001). 70.3% of the participants were vaccinated with three doses of hepatitis B vaccine. Residents experienced more exposure to blood and body fluids of a patient with Hepatitis B than nurses did (*P*<0.019). Out of the exposed 56 (30.2%) residents, 9 (4.9%) received post-exposure prophylaxis (PEP) against hepatitis B. 55.2% of the affected individuals reported the occurrence of an accident, with more nurse compliance to the notification process (*P*<0.013).

**Conclusion:** There was a high prevalence of occupational exposure among the sample population. This emphasizes the need of promoting training and education for HCWs, stressing on the importance of adherence to precautionary measures against occupational exposures.

**Disclosure of Interest**: None declared

### P227 ASSESSMENT OF PREVALENCE, FACTORS RELATED TO PERCUTANEOUS INJURIES AND ADHERENCE TO ‘OCCUPATIONAL EXPOSURE AND POST EXPOSURE PROPHYLAXIS’ NACO GUIDELINES AMONG NURSING PERSONNEL

#### S. Kaur, S. Kaur A. Guleria, J. Kaundal, P. Patil, S. Pathania, N. Vir Singh, R. Kaur

##### ^1^National Institute of Nursing Education, Post Graduate Institute of Medical Education and Research Chandigarh, Chandigarh, India

###### **Correspondence:** S. Kaur

**Introduction:** Percutaneous injuries constitute the major health hazards among the nursing personnel. NACO has provided guidelines for prevention of ‘Occupational exposure and post exposure prophylaxes.

**Objectives:** To estimate the prevalence and assess factors related to percutaneous injuries and adherence to “Occupational exposure and post exposure prophylaxis” guidelines among nursing personnel.

**Methods:** This descriptive study was conducted among bedside nurses working in a tertiary care hospital. The data was collected over a period of one month using a pre-validated survey proforma, questionnaire and a checklist. Descriptive and inferential statistics was used to analyze the data.

**Results:** Seven hundred and forty seven nursing personnel were enrolled in the study. Around one fourth (26%) nurses were ever exposed to percutaneous injuries. Mean age of the participants was 32±7.219 years with the range of 22-50 years. Nurses working in the general wards and emergency wards had highest prevalence of percutaneous injuries. Recapping of needle (86.8%) was the main factor identified, 24% had percutaneous injuries with infected device and all of them adhered to NACO guidelines. There was no statistically significant association of percutaneous injury with age, work experience, attending workshop, shift of duty, number of patients assigned and state of mind before injury.

**Conclusion:** Training programme on percutaneous injuries and its treatment regimen must be conducted to increase the effectiveness of adherence to post exposure prophylaxis.

**References**

1. NACO guidelines.National_guidelines_for_HIV_testing_21 April 2016.pdf. Accessed on 20 Dec,201

2. Acharya AS, Khandekar J, Sharma A. Awareness and practises regarding needle stick injuries among nurses in a tertiary care hospital of Delhi. Indian Journal of Community health. 2014 Dec 15;26 (4) :390-5

**Disclosure of Interest**: None declared

### P228 IMMUNITY EVALUATION AGAINST MEASLES IN EMERGENCY PERSONNEL IN A TERTIARY HOSPITAL.

#### G. CABRERA_TEJADA, C. VILLANUEVA_RUIZ, J. BARRENENGOA_SAÑUDO, M. HERNÁNDEZ_MALDONADO, V. SOLER_MOLINA, J. G. MORA_MURIEL, J. SÁNCHEZ_PÁYA

##### PREVENTIVE MEDICINE, GENERAL HOSPITAL OF ALICANTE, ALICANTE, Spain

###### **Correspondence:** G. CABRERA_TEJADA

**Introduction:** In Europe,since 2016 there has been an increase in the number of cases of measles,many of them related to the healthcare environment. It is necessary to evaluate the protection of health workers against measles;trying to reach heights above 95%.

**Objectives:** Evaluate the protection of health workers against measles in emergency room.

**Methods:** A descriptive study of the immunity against measles in the emergency services workers of a tertiary hospital between May 2018 and May 2019 was carried out. Clinical vaccination records and serology were reviewed.A worker was considered immune if they were born before 1971,had two registered doses of vaccines, had clinical history of measles or positive serology.Workers who were not immune or had unknown immunity were contacted for screening.A description of the workers and labor characteristics was made. For the association study,the Chi-square test was used. The Mc Neymar test was used to measure the changes between the immunity status (pre-assessment/post-assessment).The level of statistical significance was 0.05.

**Results:** 239 workers were evaluated, 72.4% worked in the General Emergency Service. The mean age was 46.12 +/-10.1. 71.1% were women,who worked as nurses and female attendants. Vaccination data was obtained of 13.4% of the workers.Serology data was obtained of 55.6% of the workers 98.5% were positive for measles. 75.3% of the workers were immune. All workers with a clinical history of measles were born before 1978,year of measles vaccine introduction in Spain(p <0.03). 24,7% of the workers were contacted for screening;only 42.4% went for assessment: serology was requested in 20.3% and 15.3% were vaccinated.18.7% did not perform final screening analyzes.13.8% of the workers did not want to be evaluated. Significant differences were observed (p <0.001) in the prevalence of immunity pre-assessment(75.3%) and post-assesment (81.2%).

**Conclusion:** The data of workers obtained was insufficient; probably the accessibility of records and motivation/perception of risk could be influencing. The percentage of protected workers is lower than that recommended (>95%),so is the importance of reviewing these data in all health personnel and promoting vaccination among these workers becomes more important.

**Disclosure of Interest**: None declared

### P229 ASSESSING THE ATTITUDES OF HEALTHCARE WORKERS TO THE

**INFLUENZA VACCINE**

#### N. Damani^1^, M. Borg^2^

##### ^1^Mater Dei Hospital, Msida, Malta; ^2^Infection Prevention and Control , Mater Dei Hospital, Msida, Malta

###### **Correspondence:** N. Damani

**Introduction:** The global disease burden of seasonal influenza is estimated to include up to 650,000 respiratory deaths annually and decreased healthcare productivity and as result vaccination of healthcare workers (HCWs) is recommended. However, influenza vaccine uptake globally amongst HCWs remains limited.

**Objectives:** The purpose of this survey is an attempt to ascertain the attitudes and reservations held by HCWs towards taking the vaccine.

**Methods:** A face-to-face survey of 50 HCWs in the form of a questionnaire consisting of 20 closed-questions was conducted over a period of 2 weeks. In order to encourage honest and open responses, the anonymity of the survey participants was preserved. The questions themselves were modelled upon and categorized according to the major factors outlined in the theory of planned behaviour put forward by Icek Ajzen and Martin Fishbein,

**Results:** Of the major variables aforementioned, the greatest factor influencing influenza vaccine uptake pertained to normative beliefs and subjective norms i.e. the extent to which environment and peer influences motivate subject intention to engage in the target behaviour. Within the context of attitudes surrounding behavioural beliefs, some of our participants were doubtful about the efficacy and side effect profile of the vaccine. There also appears to be a broad misunderstanding among some HCWs regarding the need to take the vaccine, with a third saying that if they did contract influenza, it would not be severe enough to warrant immunization.

**Conclusion:** In conclusion, interventions to address this issues would involve increasing social pressure on HCWs by the means of encouragement by senior staff members, roadshows, education and the display of posters and promotional material, and increased availability of vaccines in the wards.

**Disclosure of Interest**: None declared

### P230 5 YEAR SURVEY ON SHARP INJURIES AMONG HEALTHCARE WORKERS IN AN ACUTE CARE HOSPITAL IN SINGAPORE

#### H. M. L. Oh^1^, Q. S. Meng^2^

##### ^1^Infectious Disease; ^2^Infection Prevention & Control, Changi General Hospital, Singapore, Singapore

###### **Correspondence:** H. M. L. Oh

**Introduction:** Healthcare workers (HCW) are especially exposed to injury by sharp instruments in the course of their work. It is estimated that 2 million injuries cause 66,000 HBV, 16,000 HCV and about 1,000 HIV infections among 35 million HCWs each year [1].

**Objectives:** To determine the prevalence of sharp injuries and associated factors among HCWs in Changi General Hospital.

**Methods:** A retrospective analysis of 5 year surveillance study on sharps injuries (SIs) among HCWs in Changi General Hospital, a 1000 bed acute care hospital was conducted. The occupational groups, the type of sharps involved and the procedures during SIs were studied.

**Results:** There was a total of 427 SIs from 2014 to 2018. Among occupational groups, doctors had the highest SI rate (53.9%), followed by nurses (33.5%) and other allied health professionals (8.2%). There was an increasing proportion of doctors sustaining SIs, 32 doctors (47.05%) in 2016; 52 (58.4%) in 2017 and 61 (61.6%) in 2018. 77% of SIs occurred among junior doctors.

The majority of SIs were caused by hollow bore needles (42%). The source patient was identified in 396 (72.7%) of all SIs. From known sources, 46 were seropositive; 20 for HRV, 18 for HCV and 8 for HIV. No seroconversion occurred. 190 (45%) SIs were sustained during surgical procedures; 69 (16%) during blood collection; 28 (7%) during IV cannulation; 27 (6.3%) while clearing trolley, 26 (6%) during injection administration and 16 (4%) during recapping used needle. Most SIs among doctors occurred during surgical procedures (53.9%).The exact reasons for the comparatively high SI rate among doctors could be due to heavy clinical workload, insufficient training to prevent occupational exposures and the lack of safety culture. Awareness and sharps safety culture should be actively promoted in OT and among doctors in our hospital

**Conclusion:** The exact reasons for the comparatively high SI rate among doctors could be due to heavy clinical workload, insufficient training to prevent occupational exposures to blood borne pathogens and the lack of safety culture. Awareness and sharps safety culture should be actively promoted in OT and among doctors in our hospital

**References**

1. Pruss-Ustan A, Rapiti E, Hutin Y. Estimation of the global burden of disease attributable to contaminated sharp injuries among health-care workers. Am J Ind Med2005; 48:482-490.

**Disclosure of Interest**: None declared

### P231 A NATIONWIDE SURVEY ON THE HOSPITAL VACCINATION POLICIES IN SOUTH KOREA

#### S. H. Park^1^, M. Lee^2^, S. R. Kim^3^, Y. G. Kwak^4^

##### ^1^Division of Infectious Diseases, Department of Internal Medicine, College of Medicine, The Catholic University of Korea; ^2^Division of Infectious Diseases, Department of Internal Medicine, Kyung Hee University School of Medicine; ^3^Infection Control Office, Korea University Guro Hospital, Seoul; ^4^Division of Infectious Diseases, Department of Internal Medicine, Inje University Ilsan Paik Hospital, Ilsan, Korea, Republic Of

###### **Correspondence:** S. H. Park

**Introduction:** Healthcare personnel (HCP) are at risk of being exposed to or transmitting infections in hospitals and vaccination against vaccine-preventable diseases (VPD) is a well-known preventive strategy. However, there has been no information on the current hospital vaccination policies for HCP in South Korea.

**Objectives:** To investigate the current status of hospital vaccination policies for HCP in South Korea

**Methods:** We conducted a nationwide survey in 2018. Surveyed items were target VPDs and target HCP, influenza vaccination coverage, and barriers to implementing recommended vaccination polices. The online survey questionnaire was distributed to 652 hospitals, and 200 of them responded.

**Results:** Among 200 hospitals, 151 (75.5%) had the VPD screening program for new HCP and 196 (98%) had their vaccination policies including at least one vaccine. Influenza vaccine was most commonly included in their policies (97.5%, n=195), followed by HBV vaccines (69%, n=138). However, less than a quarter of hospitals included other vaccines in their policies a (MMR 24.5%, varicella 18.5%, Tdap 11%). Only 13 hospitals had all five vaccines (Influenza-HBV-MMR-Varicella-Tdap) recommended. Influenza vaccination coverage was mean 89.9% (95% CI, 88.0-91.9) and it was significantly higher in hospital supporting the full vaccination cost (91.8 % v 81.7%, p<0.001). The majority of hospitals answered that hospitals’ financial burden for the costs of vaccines and serologic tests was the most important barrier to implementing vaccination polices because these are not covered by the Korea National Health Insurance (78.6%, 121/154). A total of 42 exposure events occurred in unvaccinated HCP: varicella 15, HBV 11, influenza 9, HAV 4. measles 2, pertussis 1.

**Conclusion:** Despite the vaccination guidelines for HCP being published in 2012, the vaccination practices differed by hospitals and appeared to be insufficient to protect HCP and prevent nosocomial transmission. Strong leadership of each hospital and financial support from the government are required to implement appropriate vaccination policies in hospitals.

**Disclosure of Interest**: None declared

### P232 ANIMAL-ASSISTED INTERVENTIONS IN A PRIVATE HOSPITAL IN SOUTHERN BRAZIL: HOW INFECTION PREVENTION AND CONTROL CAN CONTRIBUTE TO IT?

#### C. C. Ponzi, N. V. Polezze, B. V. Biavatti, K. Catapan, L. J. C. de Mendonça on behalf of Programa Cão Amor, Unimed Chapecó

##### Health Care Associated Infections Control, Hospital Unimed Chapecó, Chapecó, Brazil

###### **Correspondence:** C. C. Ponzi

**Introduction:** Animal-assisted activities are complementary healing modality,and safety both for patients and animals is important for success of AAA, and the infection prevention and control (IPAC) is crucial for this to happen.

**Objectives:** to describe the implementation of a AAA program in a hospital in Southern Brazil.

**Methods:** The first action was to select adequate trainned canines, as well as to educate their tutors concerning safe behaviour in hospital (hand hygiene, compliance with hospital policies, confidentiality). Animals were screened for MRSA and received anti-parasitic medication before their first visit to the hospital and every three months. Bathing within 24 hours before visiting is compulsory as well as normatization of where animals can circulate in the institution, what kind of patiens was stated by IPAC, as well as hygiene of the canines´paws and body with chlorexidine-soaked disposable tissues.

Visits occur both in common areas of the hospital, as well as in patients wards, both for adult and pediatric patients. Immune-supressed individuals, those in contact precautions for MDRO and intensive-care patietns do not receive animal visits, as well as agitated or fobic patietns.

Formal written consent is obtained from patient or his/her tutor, as well as from the assistant physician.

All records are kept by IPAC (patients, visiting canine, pathology, age, physician, animal screening for MRSA and parasite control).

**Results:** 188 patients were visited by a canine since August, 2017 (38,3% male), age varied from 0 to 94 years of age (mean 44,8). Patients in Internal Medicine wards were 22, 81%, Orthopedics and Pediatrics 17,5% each and Neurosurgery 15,8%. No AAA events were detected (allergies, agitation, animal bad behaviour, transmition of zoonosis or MDRO´s). None of the 7 canines that participate in the program ever tested positive for MRSA or parasites.

**Conclusion:** IPAC role in normatizing AAA in a hospital setting is crucial for safety, and it is advisable to IPAC professionals to keep records of these activities, even if there is no academic or research purpose involved. AAA can be a complementary healing modality and can offer comfort both for patietns, families and staff. We kindly thank and acknoledge all the tutoers, canines and voluntary hospital staff that participate in this program.

**Disclosure of Interest**: None declared

## Poster session: Building environment

### P233 MINIMUM BUILDING REQUIREMENTS OF HOSPITAL ISOLATION ROOMS FOR PATIENTS WITH HIGHLY CONTAGIOUS AND SEVERE INFECTIONS

#### M. J. Tielemans^1^, R. de Groot^1^, J. Immerzeel^2^, M. C. Vos^1^

##### ^1^Department of Medical Microbiology and Infectious Diseases; ^2^Programma Integrale Bouw , Erasmus University Medical Centre Rotterdam, Rotterdam, Netherlands

###### **Correspondence:** M. J. Tielemans

**Introduction:** Globalization has increased the risk of spread of highly contagious and very severe diseases, such as viral hemorrhagic fever. There is a lack of studies and guidelines how to design hospital isolation rooms for these patients and what their technical requirements should be.

**Objectives:** The aim of this study was to create building requirements for hospital isolation rooms with anteroom to optimize hospital care for patients with these infectious diseases combined with ensuring safety for health care workers and admitted patients.

**Methods:** We performed a literature search to find existing national and international guidelines, reports, laws and scientific articles on building requirements for hospital isolation rooms suitable and safe to take care for patients with highly infectious diseases. Additionally, we checked the references of these reports, and guidelines on level 3 and 4 biosafety laboratories. The following information was extracted: type of material of floor, wall and ceiling covering; size of the room and anteroom; requirements and design of sanitation; airflow control including prevention of in- and outdoor air contamination; requirements for an automated room decontamination device (ARDD); security measures; clinical waste handling, and observational equipment.

**Results:** Several national and international institutions have published recommendations on building requirements. However, details of these recommendations differ considerably within and per target population. We found detailed requirements on airflow control, prevention for air contamination and clinical waste handling. Recommendations for sanitation, requirements for an ARDD, security measures and observational equipment were mentioned by several institutions but were less abundant and less detailed. Little information was given on type of material of floor, wall and ceiling covering and optimal size for the room and anteroom.

**Conclusion:** Studies on the impact of design on safety were absent and on technical requirement were sparse. Using published national and international recommendations on isolation suites, we proposed minimum building requirements for isolation suites for patients with highly infectious diseases. The result of this study will be shown in a comprehensive overview of requirements.

**Disclosure of Interest**: None declared

### P234 COMPARATIVE ANALYSIS OF BACTERIOLOGICAL AND REAL TIME POLYMERASE CHAIN REACTION (RT-PCR) TESTS IN ASSESSING THE COLONIZATION OF OBJECTS OF HOSPITAL ENVIRONMENT

#### O. Orlova^1^, M. Zamyatin^1^, N. Umzunovа^1^, Y. Savochkina^2^, V. Akimkin^3^

##### ^1^Pirogov National Medical and Surgical Center, ^2^Central Research Institute of Epidemiology, ^3^2Central Research Institute of Epidemiology, Moscow, Russian Federation

###### **Correspondence:** O. Orlova

**Introduction:** According to bacteriological tests (BTs), the level of colonization by nosocomial microorganisms of hospital facilities is from 5 to 36%, however, these tests do not always help to determine the pathways of infection. Additional information can be obtained from RT-PCR tests, but its place in the system of epidemiological survey has not yet been determined.

**Objectives:** to conduct a comparative study of BTs and RT-PCR tests results in assessing the colonization of hospital environment objects.

**Methods:** 215 samples of washout specimens from various sources of hospital environment were processed by BTs and RT-PCR tests.

**Results:** Positive results of BTs were found in 54% of samples, RT- PCR at the same objects in 80.0%, with strong correlation (r=0.92) in content. The coincidence of the results in most cases was noted at high values of DNA copies (800-10000), and mismatch of the results at low values of DNA copies or in the presence of microbial associations. BTs revealed 129 microorganisms with a predominance of coagulase-negative staphylococci (CoNS) – 70.5%. RT- PCR discovered the DNA of 288 pathogens: CoNS – 55.6%t, Streptococcus spp. – 19.8% and Enterococcus spp. – 16.7%. The frequency of pathogens from ESCAPE group was 22.7% in the BTs and 41.3% in RT- PCR(p

**Conclusion:** Although the PCR method doesn’t answer the question whether pathogens that are found on the objects of the hospital environment remain alive, but the detection of their DNA indicates that they have contaminated the surfaces for some time and could be a source of nosocomial infection of patients.

**Disclosure of Interest**: None declared

### P235 MICROBIAL CONTAMINATION OF OPERATING THEATERS AND INTENSIVE CARE UNITS AT A SURGICAL SPECIALTY HOSPITAL IN IRAQ

#### S. T. Baban, P. Akram, D. Jalal

##### Infection control and prevention, Surgical Specality Hospital, Erbil, Iraq

###### **Correspondence:** S. T. Baban

Microbial contamination of operating theatre and intensive care unit is themost frequent cause of nosocomial infections in patients. The aim of this study was to evaluate the prevalence level and variety of microbal contamination in these high risk areas in the surgical specialty hospital in Iraq. Of the 87 samples collected form surfaces, medical equipment , sterilization supplies, air and water, 48.3% yielded positive microbial growth. The most common isolates were Gram-positive bacteria (83.1%), of which S. aureus accounted for 78.6% of bacteiral pathogens isolated, followed b Streptococci (33.3%) and Enterococci (28.6%). Wherease, lower rate of Gram-negative bacterial contamination (16.9%) was observed, including E. coli (19%) adn molds (14.3%)were observed, respectively. The highest rate of microbial contamination was observed in the operating theatre rooms (35.6%)where 50% of environmental hygiene was practiced. These findings emphasize the important role of infection controlsystem to prevent the cross-transmission of nosocomial pathogens to cause contamination and infection in the critically ill hospitalized patients.

**Disclosure of Interest**: S. Baban Employee of: No conflict of interest, Grant/Research support from: No conflict of interest, Speaker's bureau of: No conflict of interest, Shareholder of: No conflict of interest, Consultant for: No conflict of interest, Paid instructor for: No conflict of interest, Other conflict with: No conflict of interest, P. Akram Employee of: No conflict of interest, Grant/Research support from: No conflict of interest, Speaker's bureau of: No conflict of interest, Shareholder of: No conflict of interest, Consultant for: No conflict of interest, Paid instructor for: No conflict of interest, Other conflict with: No conflict of interest, D. Jalal: None declared

### P236 MULTIDRUG RESISTANT BACTERIA (MDR) IN THE HOSPITAL ENVIRONMENT: CONTAMINATION OF SURFACES IN THE HEMODIALYSIS UNIT

#### L. Chaoui^1,2^, R. Ait Mhand^2^, F. Mellouki^2^, N. Rhallabi^2^

##### ^1^ Laboratory of Epidemiological Diagnosis and Environmental Hygiene, Provincial Health Delegation; ^2^Biology department, LVMQB/EB, University Hassan II Casablanca, FST Mohammedia, Mohammedia, Morocco

###### **Correspondence:** L. Chaoui

**Introduction:** The environment of hemodialysis, exposed frequently and continuously to various contaminants, the immune deficiency of patients and the multitude of coetaneous entry doors are all factors that could favor nosocomial infections. Multidrug-Resistant Organisms (MDROs) are important causes of health-related infections (HAI) and exacerbate the prognosis for hemodialysis patients.

**Objectives:** In order to control the environmental infectious risk, we conducted microbiological surface monitoring in a public hemodialysis center, aimed at assessing the contamination rate, identifying the bacteria isolated, and determining their antimicrobial resistance.

**Methods:** Ninety samples were collected during the study hemodialysis service of the Mohammedia Provincial Hospital (Morocco). Samples were collected from floors, generators, tables, beds, trolley, remotes and closets, by swabs according to ISO/DIS 14698. Cultures and identification were determined and the susceptibility patterns of the isolates were determined using Kirby Bauer disc diffusion method following the standards of CLSI.

**Results:** Among all the items sampled 44.2% (62/90) represented the overall bacterial contamination, with 69 isolates. *Staphylococcus* was the most frequently found (55.1%), with a predominance of *coagulase negative staphylococci* (CoNs) at 31.9%. *Streptococcus* was isolated at a frequency of 13% and 8.1% for *Bacillus* sp. Gram-negative isolates consisted of *Enterobacter* sp with 7.2%, *Pseudomonas* sp, *Burkholderia cepacia, Acinetobacter sp,* and *E.coli* 8.7%, 5.8%, 2.9 and 1.4% respectively. The positivity threshold ranges from 33.3% at closets to 91.7% at floor. The results of the antibiotics susceptibility testing illustrated that 25% (4/16) of *S.aureus* isolates were MRSA, Among Gram negative bacteria 25% were MDRs (Organisms were deemed MDR if they were resistant to 3 or more classes of antibiotics).

**Conclusion:** The presence of MDR bacteria in the environment is a serious risk for the health of hemodialysis patients. Hence, it is necessary to comply with current guidelines and recommendations for cleaning and disinfection of surfaces.

**Disclosure of Interest**: None declared

### P237 IMPACT OF A NOVEL E-MOTION GESTURE SENSING LIGHT SWITCH (EMGSLS) ON MICROBIAL LOAD AND CLINICAL WORKFLOW IN AN ACUTE CARE MEDICAL WARD: AN INNOVATION TOWARDS REDUCING BIOBURDEN ON HIGH TOUCH SURFACES

#### M. Cheng^1^, R. Baggs^1^, L. Ward^1,2^, C. Yang^1^, S. Johnson^1^, D. Hunter^1^, G. Hallihan^1^, R. Geransar^1^, T. Louie^1,2^, J. Conly^1,2^

##### ^1^W21C, Cumming School of Medicine, University of Calgary; ^2^Alberta Health Services, Calgary Zone, Calgary, Canada

###### **Correspondence:** J. Conly

**Introduction:** Light switches can contribute to the hospital pathogen reservoir and act as a vector for transmission to healthcare workers and patients.

**Objectives:** The touchless functionality of a novel eMGSLS was evaluated to determine user acceptance and reductions of microbial load in an acute care medical ward.

**Methods:** eMGSLS switches were installed as replacements of toggle switches with accompanying signage and information on use, but without active enforcement or monitoring of use. User perceptions were assessed through qualitative semi-structured stakeholder interviews. A visual analogue scale (VAS) was used to provide a score ranging from 0-10 for each of three attributes of technology acceptability – safety, efficiency, and overall satisfaction. Interview results were analyzed using deductive thematic analysis and scores from VASs were verified using a Kolmogorov-Smirnov statistical test. Semi-quantitative microbiological load testing was performed by culturing swab samples from eMGSLS and toggle light switches (TLS) to determine the presence of skin flora and hospital pathogens in standard single-bed patient rooms.

**Results:** User interviews indicated that stakeholders generally perceived the eMGSLS as safe and efficient with an overall score of 6.81 out of 10, but a learning curve was encountered by 72% of users. Feasibility analysis revealed that the bathroom light switches had the most pathogenic microbes including *Staphylococcus aureus* and *Enterococcus*. Prior to daily surface cleaning, skin flora and potential pathogens in approximately 8% of samples were recovered in 93% (68/73) of TLSs and 69% (45/65) of eMGSLS. In a subset of eMGSLS cultures with prior intensified education, contamination was reduced by 55% (11/20). Post cleaning results showed both types of switches were easily cleaned.

**Conclusion:** The safety and efficiency of this innovation in healthcare workflow depends on familiarity, education and adaptation. An eMGSLS has the potential to reduce nosocomial pathogen transmission from a high touch surface. Initial results support further investigation into the use of eMGSLS in acute care settings.

**Disclosure of Interest**: None declared

### P238 IMPLEMENTATION OF AN OPERATING ROOM PROGRAM FOR HYGIENE COMPLIANCE IMPROVEMENT IN 4TH MILITARY HOSPITAL IN WROCLAW

#### Z. Rucińska, A. Rucinski

##### Operating Room department, 4th Military Clinical Hospital with Policlinic Rudolfa Weigla 5, Wroclaw, Poland

###### **Correspondence:** Z. Rucińska

**Introduction:** Upon opening of a new integrated Operating Room(OR) suite at the 4^th^ Military Hospital in Wroclaw in November 2018, a new process for cleaning was introduced. The aim was to have a process to reduce the risk of transmission of pathogens in the environment that was simple, fast and effective.

**Objectives:** The OR suite has eleven ORs, with eight hundred procedures performed per month. Efficiency of cleaning was the primary focus, recognising that staff engagement and satisfaction would be key to success.

**Methods:** The OR program for cleaning was introduced in July 2018 with:

pre-impregnated single use mops for cleaning floors

introduction of cleaning equipment: cleaning carts with mops and sweeping sets

timing between case and terminal cleans

observational audits of cleaning

cleaning regimes for staff

microbial contamination of surfaces using swabs

cleaning audits with UV markers on High Touch Objects (HTOs)

training for staff

Six months post-implementation, assessment of the program was conducted with:

surveys to determine the satisfaction levels of cleaning and medical staff

timing between case and terminal cleans*

microbial contamination of surfaces using swabs*

cleaning audits with UV markers on HTOs*

comparison of disinfectant use before and after program introduction

*as baseline

**Results:**

**Table:** 

**Improvements in cleaning OR**			
	**Pre**	**Post**	**Overall impact**
Cleaning effectiveness	33%	85%	158% improvement
Positive swabs	13.9%	4.5%	9.4% reduction
Cleaning Time per OR	48:28	14:10	34:18
Cost of disinfectant			31% reduction
**Staff Satisfaction – staff feedback**			
Program helped structure OR cleaning			> 80%
Carts & mops good/very good			100%
Mops relieved physical burden on back & muscles			73%
Better than previous cleaning system			91%
Training helpful in improving skills & effectiveness of OR cleaning			78%
More satisfied with work			89%

**Conclusion:** The OR cleaning program, with pre- and post-implementation measurements for cleaning effectiveness, microbial burden on surfaces, cleaning time and disinfectant use provided an objective way to assess cleaning in the OR suite. Significant improvements were seen in all areas, following the introduction of training of staff, cleaning regimes and user-friendly cleaning equipment. These improvements allowed staff to have more time for other activities. This resulted in a high level of staff engagement, which has positive benefits in staff satisfaction and retention.

**Disclosure of Interest**: None declared

## Poster session: Disinfection / sterilization / environment 1

### P239 Withdrawn

### P240 EVALUATION OF THE MAXIMAL INTERVAL STORAGE OF FLEXIBLE ENDOSCOPES AND TRANSOESOPHAGEAL ECHOCARDIOGRAPHIC (TEE) PROBES BEFORE REPROCESSING AT A TERTIARY CARE CENTER IN LEBANON

#### J. TANNOUS^1^, N. K. Zahreddine^1^, T. KARDAS^1^, Z. KANAFANI^2^, S. KANJ^2^

##### ^1^Infection Control and Prevention Program; ^2^INFECTIOUS DISEASES, AMERICAN UNIVERSITY OF BEIRUT MEDICAL CENTER, Beirut, Lebanon

###### **Correspondence:** J. TANNOUS

**Introduction:** Proper storage of flexible endoscopes and TEE probes is an essential step to prevent contamination with standardized reprocessing using high level disinfection. The maximum interval time of endoscope storage before reprocessing remains unresolved. Internationally, the optimal storage cabinet features have not been determined yet.

**Objectives:** The objective of this study was to evaluate the effectiveness and safety of the adopted interval time storage of endoscopes and TEE probes at the American University of Beirut Medical Center (AUBMC).

**Methods:** Microbiological surveillance cultures from manually reprocessed TEE probes stored in a newly dedicated storage cabinet designed with positive pressure HEPA-filter was started in January 2018. The maximal interval storage time was set at 7 days. Five samples were obtained between January and August 2018 from 5 different TEE probes randomly selected. Swabs from the distal parts were obtained on day 7 before reprocessing.

Surveillance cultures from reprocessed endoscopes stored in regular clean cabinets were obtained between 2015 and February 2019. The maximal interval storage time was set at 72 hours. A total of 23 endoscopes were randomly cultured at 72 hours and samples were obtained from the distal end including the elevator mechanism, and from the flushed instrument channel.

**Results:** Four surveillance cultures taken from the TEE probes between January and August 2018 were negative. However, 4 colonies of Coagulase-negative Staphylococcus were detected in one TEE probe. On the other hand, no growth of organisms was detected from the 19 samples taken from the flexible endoscopes.

**Conclusion:** No growth of pathogenic bacteria was detected from the reprocessed flexible endoscopes and TEE probes at 72 hours and 7 days respectively verifying the effectiveness of the adopted interval time of storage. Maintaining the maximal interval storage time with ongoing surveillance cultures is essential to validate the effectiveness and safety of the reprocessing methods.

**Disclosure of Interest**: None declared

### P241 EVALUATION OF HIGH LEVEL DISINFECTION OF GASTROINTESTINAL ENDOSCOPES

#### N. N. Nawar

##### Clinical Pathology, Kasr Al Aini Cairo university, Mohandesene, Giza, Egypt

**Introduction:** Flexible endoscopes may become heavily contaminated with blood, secretions and microorganisms during use. These instruments are difficult to clean and disinfect because of their complex design and narrow channels.

**Objectives:** Our study aim was to compare two different cycle duration of High Level Disinfection (HLD) process (20 minutes versus 40 minutes)using ( >2% glutaraldehyde) , and determine whether adequate bacterial decontamination of Gastrointestinal (GI) endoscopes was achieved after complete reprocessing cycle. Also, simultaneous samples were taken from Automated endoscope reprocessors (AERs) and decontaminated Gastrintestinal endoscope, to compare efficacy of both sampling methods in GI endoscopy surveillance cultures.

**Methods:** This evaluation was conducted in “GI Endoscopy Unit of Kasr Al Aini Teaching Hospital” Cairo, Egypt. Six upper endoscopes and 3 AERs were in service during the study. A total of 320 samples were collected, 160 samples were obtained by rinsing the Biopsy channels of Gastroscopes, and 160 swab samples of residual water collected from the internal surfaces of AERs after a complete reprocessing cycle. Of these samples 160 were obtained after 20 minutes and 160 after 40 minutes of High- level Disinfection .

**Results:** A total of (4.4%) (7/160) Gastroscope biopsy channel (BCs) were found positive. No culture positive samples were obtained from Automated endoscope reprocessor samples (AERs) ( 0% [0/160]). A statistically significant difference in the number of positive samples collected from the BCs and AERs was observed (p <0.0001). Also, by comparing two different cycle duration of High Level Disinfection (20 minutes and 40 minutes of High Level Disinfection), we found that longer cycle duration yielded less positive cultures, yet, surprisingly it was not statistically significant.

**Conclusion:** our study suggests that culturing samples from GI endoscopes biopsy channel is more effective for monitoring the effectiveness of reprocessing after High Level Disinfection than culturing swab samples from AERs. Also, by comparing two different cycle duration of High level disinfection ( 20 minutes and 40 minutes of HLD), we found that longer cycle duration yielded less positive cultures, yet it was not statistically significant.

**Disclosure of Interest**: None declared

### P242 CHARACTERIZATION OF THE 'HOSPITAL MICROBIOME' AND THE SPREAD OF ANTIMICROBIAL RESISTANCE AND BIOFILM ON THE INTENSIVE CARE UNITS FROM DIFFERENT REGIONS.

#### K. Aljohani^1^, K. Vickery^2^, D. Costa^3^, S. Dancer^4^, D. Melo^3^, A. Tipple^3^, A. Devaa^2^, I. Gosbell^5^, S. Jensen^5^, G. Whiteley^6^, H. Hu^7^

##### ^1^Central Military Laboratories and Blood Bank, Prince Sultan Military Medical City, Riyadh , Saudi Arabia; ^2^Faculty of Medicine and Health Sciences, Macquarie University, SYDNEY, Australia; ^3^Faculty of Nursing, Federal University of Goias, Goias, Brazil; ^4^NHS Lanarkshire, NHS Lanarkshire, Glasgow, United Kingdom; ^5^Antimicrobial Resistance and Mobile Elements Group, Ingham Institute of Applied Medical Research, sydney; ^6^Whiteley Corporation, Whiteley Corporation, Nth Sydney; ^7^Surgical Infection Research Group, Macquarie University, SYDNEY, Australia

###### **Correspondence:** K. Aljohani

**Introduction:** Healthcare associated infections are a significant burden on health service providers, in terms of cost, morbidity and mortality. Organisms causing these infections can be sourced from the inanimate environment around patients. Could the difficulty of eradicating these organisms be because they reside insurface biofilms?

**Objectives:** Investigate the microbial structure and diversity of biofilms associated with healthcare infections

**Methods:** Following environmental cleaning and disinfection, samples from Brazil (n=40), Australia (n=44), Scotland (n=8) and Saudi Arabia (n=20) were obtained by aseptically cutting fragments from hospital furnishings and equipment. Bacterial load/cm^2^was determined by quantitative PCR of the 16S rRNA gene. Culturable MDRO were detected by sonication in tryptic soya broth followed by culture on HBA plates and specific methicillin-resistant Staphylococcus aureus(MRSA), vancomycin-resistant enterococci(VRE) and extended spectrum beta-lactamase(ESBL) agar plates. Biofilm presence was visually confirmed by live/dead BacLight staining combined with confocal laser scanning microscopy.

**Results:** Sequencing of 16S rRNA genes identified *Proteobacteria, Firmicutes, Actinobacteria*and *Bacteroidetes*as the most abundant bacterial phyla, with 48 different genera across the samples. the mean of the microbial load,as determined by PCR, being 4.4x10^3^, 5.5x10^5^, 2.6x10^4^and 3.4x10^4^/cm^2^for Brazilian, Australian, Scottish and Saudi hospitals respectively. Overall 59% of the 112. MDRO were isolated from 4/26 (15%), 13/23 (57%), 2/4 (50%) culture positive samples from Brazilian, Australian and Scottish hospitals respectively, MDRO were not isolated from Saudi samples.

**Conclusion:** Biofilms containing pathogens, including ESKAPE species, are widespread in hospitals worldwide. As bacteria within biofilms are tolerant to disinfection, this may be one mechanism through which ESKAPE organisms persist in the environment, and are subsequently transmitted to patients.

**Disclosure of Interest**: None declared

### P243 OPTIMAL TIME INTERVAL FOR ENHANCED TERMINAL DISINFECTION OF AN OPEN-BAY ICU TO MINIMIZE ACQUISITION OF EXTENSIVELY-DRUG RESISTANT ACINETOBACTER BAUMANNII

#### R. A. Moghnieh^1^, M. Jadayel^2^, H. Kadri^1^, D. Abdallah^1^, R. Kassem^1^, L. Ajjour^1^, H. Hafez^1^, S. Al-Hassan^1^, T. Jisr^1^, H. Tamim^3^

##### ^1^Makassed General Hospital; ^2^Beirut Arab University, ^3^American University of Beirut, Beirut, Lebanon

###### **Correspondence:** R. A. Moghnieh

**Introduction:** Enhanced Terminal Disinfection (ETD) is reported to control extensively-drug resistant Acinetobacter baumannii (XDR-AB) outbreaks in intensive care units (ICU). At Makassed General Hospital’s 5-bed open-bay ICU, ETD using vaporized H_2_O_2_ is performed only upon intensivists’ requests.

**Objectives:** We determined the incidence of XDR-AB/1000 ICU days (d), risk factors of acquisition (RFA), and the optimal time interval to routinely perform ETD post-ICU evacuation.

**Methods:** This is a retrospective chart review of patients (pt) admitted to ICU between January 2016-May 2018. They were divided to 3 groups(G): G1: pt who acquired XDR-AB 48 hours after admission till d-7 post-ICU discharge, G2: pt who did not (control), G3: pt who previously harbored XDR-AB before ICU admission. RFA were determined through comparing G1 and G2 through univariate and multivariate (MV) analysis. The dates where ETD was performed were plotted on a calendar and the days of the study were divided into weeks according to ETD dates. In each week post-ETD, we determined the incidence of XDR-AB acquisition/1000 ICU d. The incidence versus weeks was plotted on a graph and a trend was determined. We set hypothetical breakpoints (week) where we observed a visual increase in the incidence of acquisition. These breakpoints were included in the analysis of RFA to determine the optimal time for ETD.

**Results:** 335 patients were included and 13% of the them acquired XDR-AB(G1). Independent RFA were: mechanical ventilation >2d(OR=4.11,P=0.003), use of urinary catheter >5d(OR=3.72,P=0.05), wound presence (OR=3.72,P=0.05), and XDR-AB contact pressure >3d(OR=5.51,P<0.0001). As for the longitudinal variation in the incidence of XDR-AB acquisition, we observed an ascending trend(*P*=0.003) with a potential breakpoint at week-7 post-ETD. Patients who resided in ICU after week-7 had a higher chance of acquiring XDR-AB compared to those who resided before it through MV model(OR=1.48,P=0.35).

**Conclusion:** The optimal schedule for performing ETD is every 7 weeks. Adherence to Infection Control guidelines and bundles of care would help decrease the rate and risk of XDR-AB acquisition in our ICU.

**Disclosure of Interest**: None declared

### P244 EPIDEMIOLOGY MONITORING OF ENVIRONMENTAL MICROBIAL CONTAMINATION IN THREE ACUTE HOSPITALS IN EMILIA ROMAGNA REGION

#### M. F. Pedna^1^, M. Sanchi^2^, F. Congestri^1^, M. Fantini^1^, M. Mazzotti^2^, I. Melandri^2^, V. Sambri^1^

##### ^1^Microbiology, Area Vasta Romagna, Pievesestina; ^2^Formula Servizi, Forlì, Italy

###### **Correspondence:** M. F. Pedna

**Introduction:** The role of surface contamination in the transmission of health care-associated pathogens is being recognized increasingly. Environment may facilitate transmission of pathogens, including vancomycin-resistant enterococci (VRE), *Clostridium difficile*, *Acinetobacter spp*., *Methicillin-ResistantStaphylococcus aureus*(MRSA) and Norovirus. Unrecognized environmental reservoirs may be responsible of outbreaks or ongoing sporadic transmission.

**Objectives:** Objective of the study was to monitor environmental microbial contamination after implementation of a new enzymatic sanitizer(StayClean) using Surface Recovery kit (SRK, COPAN Italia) sampling method.

**Methods:** Microbial contamination survelliance was performed for four weeks on Geriatry and Dyalisys departments through sampling of items, equipment and general sites in bed spaces and rooms for a total of 900 points after sanitization with the new enzymatic detergent (t=0) and after 24 h. The first week was used to define the baseline of microbial contamination on surface pretreated with the routine detergent. After collection, the FLOQSwab was broken within the transport tube containing 1 mL of diluent solution and sent to the laboratory for analysis.The whole 1 ml was used to inoculate Columbia Agar+5% sheep blood plate at 35°C up to 48h. Total Colony Count (TCC) at t=0 and after 24h have been monitored. The bacteria identification was performed by MALDI-TOF mass-spectrometry.

**Results:** in the first week of monitoring inGeriatry the average of TCC at t=0 and after 24h was 30 and 201 CFU respectively while, after three weeks of treatment with the new detergent the average of TCC at t=0 and after 24h was 17 and 151 CFU respectively. During the first week of monitoring in Dialysis the average of TCC at t=0 and after 24h was 11 and 31 CFU respectively. After three weeks of new treatment the average of TCC at t=0 and after 24h was 6 and 27 CFU respectively. *Staphylococcus aureus*, MRSA, *Enterococcus faecalis* and *Aspergillus fumigatus*have been identified only before sanitization.

**Conclusion:** The enzymatic detergent was associated with a stable decrease of surface pathogens compared to conventional sanitation system used.

**Disclosure of Interest**: None declared

### P245 STUDY ON THE CORRELATION OF SMALL VOLUME NEBULIZER CLEANING FREQUENCY AND BACTERIAL COLONIZATION

#### H. C. Liang^1^, H. Mei Luan^2^

##### ^1^Infection control department; ^2^Nursing, National Taiwan University Hospital Hsin-Chu Branch, Hsin-Chu, Taiwan

###### **Correspondence:** H. C. Liang

**Introduction:** In order to avoid respiratory infections caused by the use of small volume nebulizer(SVN) , it’s important to maintain the sterilized of the nebulizer device. Many studies have shown that the rate of bacterial colonization by the use of SVN at home is more than 50%. The pathogen is Pseudomonas aeruginosa,Staphylococcus aureus, the literature review found that most of the cleaning methods for patients at home, there are not many studies in the hospital. Some studies have pointed out that it should be immersed and then cleaned to achieve the effect, but it’s also cannot be implemented clinically.

**Objectives:** The purpose of this study was to compare the difference in bacterial colonization after every 6 hours of use and after every 24 hours of use.

**Methods:** The study was conducted in a regional hospital with a chest ward.The period of acceptance was 2019/1/1-2019/12/31, and the patients who were admitted to COPD or Pneumonia ,and were excluded influenza and tuberculosis. It is estimated that 60 participants.

The SVN is usually treated every 6 hours. After each use, the device needs to be disassembled and rinsed with sterilized water, and then covered with sterilized towel. We will simplify the cleaning process and inject it with sterilized water after every 24 hours. After the SVN is shaken and rinsed, the SVN device is directly placed upside down and covered with the sterilized towel, which is performed by trained nurses.

**Results:** This research is still in progress.

**Conclusion:** This study based on a randomised controlled trial, it’s an objective result and will provide a new method of improving the hospital to simplify workflow and ensure the patient safety.

**References**

Jarvis, S., Ind, P. W., Thomas, C., Goonesekera, S., Haffenden, R., Abdolrasouli, A., ... & Shiner, R. J. (2014). Microbial contamination of domiciliary nebulisers and clinical implications in chronic obstructive pulmonary disease. *BMJ open respiratory research*, *1*(1), e000018.

Device Cleaning and Infection Control in Aerosol Therapy

Tai, C. H., Lin, N. T., Peng, T. C., & Lee, R. P. (2011). Cleaning small-volume nebulizers: the efficacy of different reagents and application methods. *Journal of Nursing Research*, *19*(1), 61-67.

**Disclosure of Interest**: None declared

### P246 ASSESSMENT OF THE MICROBIOLOGICAL QUALITY OF THE MEDICO-TECHNICAL EQUIPMENT, SURFACES, PREMISES AND STAFF HANDS IN THE DEPARTMENT OF NEONATOLOGY OF THE DEPARTMENTAL UNIVERSITY HOSPITAL CENTER OF OUÉMÉ-PLATEAU IN BENIN IN 2018

#### C. C. Degbey^1,2^, A. Challa^3^, J. Todedji ^4^, C. Koudjo^5^, A. Dossou^6^, M. MAKOUTODE^7^

##### ^1^Clinique Universitaire d'Hygiène Hospitalière , CNHU-HKM, Cotonou; ^2^Santé Environnement, IRSP CAQ/ UAC, Ouidah; ^3^Laboratoire Médical, Centre de Santé de Houinmè , Porto-novo; ^4^Santé Environnement , IRSP CAQ /UAC, Ouidah; ^5^Centre Hospitalier Universitaire Départemental de l'Ouémé/Plateau, , Porto-Novo; ^6^Centre Hospitalier Universitaire Départemental de l'Ouémé/Plateau, Porto-Novo; ^7^Santé Environnement , IRSP CAQ/UAC, Ouidah, Benin

###### **Correspondence:** C. C. Degbey

**Introduction:** Hospital hygiene is a set of preventive measures essential to ensure the quality of care in health facilities

**Objectives:** The objective of the present study was to evaluate the microbiological quality of the medico-technical equipment, the surfaces, the premises and the hands of the personnel in the neonatology department of the Ouémé-Plateau’s Departmental University Hospital Center (CHUD-O/P)

**Methods:** This was a descriptive cross-sectional study that included 130 swab samples divided into four batches: Batch No 1 (Medico-technical equipment); Batch No 2 (surfaces); Batch No 3 (Premises) and Batch No 4 (Staff Hands) and ran from April 14 to August 31, 2018. Each sample was also examined macroscopically, microscopically, consisting of the fresh state, the Gram stain, and then a selective medium inoculation in petri dishes in order to determine some characteristics of the bacteria. The identification of the bacteria was made on the Le Minor gallery, which was seeded and incubated in an oven at 37°C for 24 hours for the identification of enterobacteria (Gram-negative bacilli). Gram-positive cocci were identified by a series of biochemical tests that were catalase and DNAse.

**Results:** The results showed that 105 samples out of 130 gave a positive culture, 80.76% (high risk of contamination). A total of 369 germs of all species were identified. The isolated microorganisms were *Staphylococcus aureus* (21.95%), *Staphylococcus spp* (20.32%), *Klebsiella pneumoniae* (18.97%), *Escherichia coli* (17.07%), *Candida spp* (12.73%) and *Candida albicans* (8.94%). The majority of identified bacteria had resistance to commonly used antibiotics (only 3 antibiotics sensitive on the 7 tested).

**Conclusion:** : It is useful to organize bio-cleaning and high-level disinfection in a short time in order to provide the care in hygienic conditions recommended by the World Health Organization.

**Disclosure of Interest**: None declared

### P247 EVALUATION OF BACTERIAL SURVIVAL ON INERT SURFACES IN A HYPERBARIC ENVIRONMENT

#### L. Hendier^1^, H. Soule^2^, M. Abbas^2^, M.-A. Magnan^3^, D. Pittet^2^, R. Pignel^3^

##### ^1^Faculty of Medicine, University of Geneva; ^2^Infection Control Programme, University of Geneva Hospitals and Faculty of Medicine; ^3^Diving and Hyperbaric Unit, University of Geneva Hospitals, Geneva, Switzerland

###### **Correspondence:** L. Hendier

**Introduction:** The effects of hyperbaric oxygen on bacteria biological tissues are either bactericidal or bacteriostatic, depending on the type of bacteria. However, the effects on bacteria on inert surfaces is unknown. This may have implications for terminal cleaning methods and potentially cross-transmission of pathogens in hyperbaric chambers.

**Objectives:** The aim of this study is to evaluate the effect of hyperbaric environments on the bacterial survival on inert materials as compared to normal pressure environment.

**Methods:** We deposited 10^6^ cfu of either *Staphylococcus aureus* (NC 10788) or *Escherichia coli* (ATCC 10536) on 1 cm^2^ strips of inert materials commonly found in hyperbaric chambers (plastic, metal and seat upholstery [imitation leather]). Three of each contaminated tiles were placed inside the hyperbaric chamber (Comex1200Alu) and exposed to a standard hyperbaric compression of 95 minutes duration (2.5 atm; 253.31 kPa). Corresponding control strips were placed outside the chamber (1 atm; 101.33 kPa) for 95 minutes. At the end of each experiment, the contaminated tiles were placed in 100 ml tryptone soy mixture, shaken vigorously, and then the recovering solution was inoculated on agar for 48 hours. The number of colonies for each tile was counted, and we compared the average logarithmic reductions between tiles inside and outside the chamber for each material and each bacterium using Student’s *t*-test. All experiments were inspired by the application standard stage 2/step 2 NF EN 14561.

**Results:** Mean log reductions of *S. aureus* and *E. coli* do not show statistically significant differences with exposure to either a normobaric (1 atm) or a hyperbaric (2.5 atm) environment in ambient air, regardless of the media used.

**Conclusion:** Standard pressurization of air (2.5 atm for 95 minutes) has no proliferative or bactericidal action on *S. aureus* or *E. coli* colonies deposited on inert supports compared to those present outside a hyperbaric chamber. Further studies using different species and serial compressions are required to broaden our knowledge of bacterial survival in hyperbaric chambers.

**Disclosure of Interest**: None declared

### P248 HEALTHCARE FACILITY ENVIRONMENTAL CLEANING IN RESOURCE-LIMITED SETTINGS (RLS): DEFINING BEST PRACTICES AND DEVELOPING TOOLS FOR IMPLEMENTATION

#### M. Patrick^1^, S. Mehtar^2^, B. Park^1^, R. Smith^1^

##### ^1^CDC, Atlanta, United States; ^2^Stellenbosch University, Stellenbosch, South Africa

###### **Correspondence:** B. Park

**Introduction:** Robust environmental cleaning is a critical infection prevention and control (IPC) practice in healthcare facilities, but implementation is complex and can be expensive. An online survey administered to key stakeholders across Africa in 2018 identified significant gaps and weaknesses, including a lack of accessible and appropriate technical guidance. In response, we developed guidance specific for RLS to improve environmental cleaning practices.

**Objectives:** To define best practices for healthcare facility environmental cleaning programs in resource-limited settings.

**Methods:** A committee of experts in environmental cleaning in resource-limited settings was convened to review current available best practices and standards from selected high-resource settings. Using a consensus-driven strategy, the expert committee identified and compiled the most relevant and feasible best practices for resource-limited settings.

**Results:** A best practices manual was developed and includes three major sections. The first describes best practices for environmental cleaning supplies and equipment, including guidance on the preparation and use of the most widely available and appropriate chemicals and equipment used in cleaning. The second section addresses environmental cleaning procedures in all major facility areas, including outpatient, general inpatient and specialized care wards. Detailed guidance and emphasis is placed on wards with increased healthcare-associated infection risk, such as maternity wards and intensive care units. The final section addresses implementation of environmental cleaning programs, including the importance of integration within IPC programs and the use of defined multi-modal strategies. A toolkit was developed which will guide the step-wise implementation of the best practices guidance.

**Conclusion:** Key resources for environmental cleaning in RLS were developed. They will be refined and updated through pilot evaluations, which will document usability, feasibility and impact on processes and health outcomes.

**Disclosure of Interest**: None declared

### P249 EVALUATION OF OPERATING ROOM SURFACES CLEANING AND DISINFECTION BY VISUAL INSPECTION, MICROBIOLOGICAL ANALYSIS AND ADENOSINE TRIPHOSPHATE

#### E. A. Silva Nascimento^1^, V. De Brito Poveda^2^

##### ^1^Medical and surgical nursing, University of São Paulo (Universidade de São Paulo-USP); ^2^Medical and surgical nursing department, UNIVERSITY OF SÃO PAULO, São Paulo, Brazil

###### **Correspondence:** V. De Brito Poveda

**Introduction:** The surface cleaning and disinfection in Operating Rooms (OR) is a main factor for environmental contamination control. In spite of this, the efficiency evaluation of environmental cleanliness is often based on visual inspection. Recently, quantitative methods for evaluating environmental cleanliness entered in debate, among them the bioluminescence Adenosine Triphosphate (ATP).

**Objectives:** To evaluate the presence of organic or biological material between cleaning and disinfection (CD) of the operating room by different monitoring methods: visual inspection, ATP and microbiological analysis.

**Methods:** This is a descriptive-exploratory quantitative study using visual inspection, ATP samples and microbiological culture as indicators for the evaluation of CD. The collection was performed before and after the CD of the following areas: anesthesia cart, electric scalpel, surgical table, circulating nurse table and infusion pump.

**Results:** 90 samples of ATP, 90 microbiological samples and 45 surfaces underwent visual inspection after CD on five surfaces from nine operating rooms were evaluated. There was a statistically significant reduction (p <0.0001) of the ATP values ​​in the periods before and after OR CD, with a mean reduction percentage of 92.6%. Regarding the visual inspection, 42 (93.3%) of the evaluated surfaces were considered clean and only three (6.7%) surfaces, namely electric scalpel, anesthesia car and surgical table were considered to be faulty. In the microbiological analyzes there was a reduction in the number of colonies of microorganisms identified in the evaluated surfaces.

**Conclusion:** The CD process reduced the microbial load and organic matter of the evaluated surfaces, demonstrated by the results obtained by the ATP and microbiological evaluation, but the visual inspection as a unique tool to evaluate the effectiveness of the cleanliness of surfaces, may generate a false impression of clean environment. Although bioluminescence ATP is an interesting tool for assessing the quality of cleanliness, as well as an educational measure by demonstrating results that are quickly understandable to the team, its high cost may be an inhibiting factor for its implementation in many Brazilian hospitals.

**Disclosure of Interest**: None declared

### P250 THE USE OF ATP SURFACE TESTS TO EVALUATE THE CLEANLINESS OF TOILETS IN HOSPITAL ROOMS

#### P. De Waegemaeker, R. Ablorh, E. De Brabandere, I. Leroux-Roels

##### Hospital Infection Control, Ghent University Hospital, Ghent, Belgium

###### **Correspondence:** P. De Waegemaeker

**Introduction:** Outbreaks with intestinal bacteria, such as *Clostridium difficile* or carbapenamase producing *Enterobacterales*, suggest that toilets can play a role in the spread of these microorganisms (patient to patient transmission). In that respect, toilet cleanliness is very important and should be evaluated by the hospital infection control team.

**Objectives:** With this study, we aim to evaluate the cleanliness of the toilets in our hospital by using ATP surface testing.

**Methods:** Within the context of the European consortium project i-4-1 health, ATP tests (3M) on patient care materials and environmental surfaces were performed and revealed a very high bioburden in the toilets of our hospital. This observation incited us to further evaluate the cleanliness of all patients toilets on 4 patients wards using ATP surface tests. We compared the effect of cleaning products, age of the toilets and sampling location within the toilet bowl.

**Results:** We sampled 90 toilets below the rim, since that’s the hardest reachable area for cleaning. 98% of the samples showed ATP values of more than 10.000 RLU, classified as heavily soiled. Further investigation showed no difference between cleaning products (10 samples). There was also no difference between older or more recent toilets (10 samples). The results were mainly determined by sampling location, with very high values below the rim and, in contrast, good results on other surfaces of the bowl. Evaluation of 1 rim free toilet in parallel with a standard toilet clearly showed the benefits of the first.

**Conclusion:** We demonstrated the usefulness of ATP tests in evaluating the cleanliness of toilets. Overall the toilets were clean (low ATP value), with the exception of the rim of the toilets which had a very high bioburden. This can probably be explained by biofilm development and contamination on the rough surface below the rim, which can pose a risk to patients. The use of rim free toilets should be considered.

**Disclosure of Interest**: None declared

### P251 PRACTICE-LIKE VIRUCIDAL EFFICACY EVALUATION OF DISINFECTANT WIPES: USA VS. EUROPE

#### S. Pahl^1^, J. Steinmann^1^, F. Brill^1^, D. Paulmann^1^, B. Becker^1^, B. Bischoff^1^, E. Steinmann^2^

##### ^1^Dr. Brill + Partner GmbH, Hamburg; ^2^Molecular and medical virology, Ruhr University Bochum, Bochum, Germany

###### **Correspondence:** F. Brill

**Introduction:** Application of disinfectant wipes in hospitals are a major measurement for infection prevention. These wipes should be able to inactivate viruses on environmental surfaces and to prevent virus transfer.

**Objectives:** The aim of this study to present a practice-like alternative to the ASTM E2967-15 wiperator method based on EN 16615, a method evaluating the bactericidal and yeasticidal efficacy of disinfectant wipes, in order to present a practice-relevant method evaluating the virucidal efficacy of wipe products.

**Methods:** Tests based on EN 16615 were performed with four commercially available RTU disinfectant wipes and a standardized wipe material impregnated with the respective impregnation liquids of the tested RTU wipes. Murine norovirus (MNV), adenovirus type 5 (AdV) and polyomavirus SV40 (SV40) were chosen as test viruses. Pass criteria for efficacy evaluation were a four log10 reduction in virus titre on the contaminated surface and prevention of eventual virus spread on treated surfaces as a consequence of the wiping procedure.

**Results:** The PAA-based wipe A sufficiently inactivated all three test viruses, whereas the QAC-based product B failed to reach such reduction against AdV, but sufficiently inactivated MNV and SV40. The second QAC-based wipe C was not sufficiently active against both AdV and MNV, however, sufficiently inactivated SV40. The 2-propanol-based wipe D was not able to demonstrate sufficient efficacy against all tested viruses. Furthermore, a spread of test viruses on the treated surface was observed for the 2-propanol-based product only.

**Conclusion:** Obtained data of tests based on EN 16615 against viruses demonstrated that the method can be transferred to determine the virucidal efficacy of disinfectant wipes on a surface in a more practice-relevant way compared to ASTM E2967-15. The data resulting in more reliable data compared to existing test methods for virucidal efficacy evaluation.

**Disclosure of Interest**: None declared

### P252 STORAGE TIME EFFECTS ON THE BACTERICIDAL ACTIVITY OF QUATS DISINFECTANT-IMPREGNATED WIPES

#### X. Song^1^, A. Zille^1^, L. Vossebein^2^

##### ^1^University of Minho, 2C2T – Centro de Ciência e Tecnologia Têxtil, Guimarães, Portugal; ^2^Faculty of Textile and Clothing Technology, Niederrhein University of Applied Sciences, Mönchengladbach, Germany

###### **Correspondence:** X. Song

**Introduction:** An efficient cleaning and disinfection practice plays a crucial role in preventing cross-contamination in nosocomial environment. The binding of quaternary ammonium compounds (QACs) on cellulosic material which may fail the disinfection process hinder the broad use of pre-impregnated disinfecting wipes (ready-to-use disinfectant wipes) in hospital. Moreover, ageing of disinfectant-impregnated wipes may affect the products’ disinfection performance later in practice but very little research was performed regarding this issue.

**Objectives:** The purpose of this study is to evaluate the adsorption change and antimicrobial activity due to the ageing of the disinfecting wipe over storage time with and without atmospheric Double Barrier Dielectric (DBD) plasma pre-treatment. The main questions to be answered are: i) How does the adsorption of active ingredients onto textile substrate change with storage time; ii) How does the antimicrobial efficacy vary by time. The study of the adsorption of QACs in storage is important to ensure hospitals daily workflow and to complement the products’ user manual of disinfectant and wipes in the market.

**Methods:** Plasma–treated and untreated commercial wiping materials of polyester (PET), 55% cellulose/45%PET and 100% cotton were immerged into QACs solution with a certain liquor ration for 30 min, 3, 7, 15 and 30 days. The absorption and adsorption of quats onto wiping material were measured by UV spectrophotometer. Standards ASTM E 2149-13a was carried out to assess the antimicrobial efficacy.

**Results:** There was evident increase of QACs adsorption in all wipes including plasma treated ones in function of the storage time. Plasma–treated PET showed a small increase in adsorption (~5%) while cotton wipes display a decrease in QACs adsorption (~5%). Surprisingly, the plasma-treated blend wipes are able to adsorb almost the double of the untreated ones (from 15% to around 30%). Cotton sample shows the lowest antimicrobial effect in all the cases. In PET and blend wipes the storage time has a significant influence on the Log reduction of *E.coli*. in both untreated and plasma treated samples.

**Conclusion:** The use of blend wipes is recommended due to the best performance between adsorption and antimicrobial effect. Moreover, plasma treatment can significantly improve Gram-negative bactericidal effect if necessary.

**Disclosure of Interest**: None declared

### P253 DEVELOPMENT OF THE PROTOCOL FOR STANDARDIZATION OF ENVIRONMENTAL CLEANING PRACTICE IN A KOREAN HOSPITAL

#### E. S. Lee^1^, M. J. Shin^1^, H. K. Seo^1^, D. R. Lee^1^, J. H. Hong^1^, H. B. Kim^2^, S. M. Moon^2^, H. R. Kim^3^, J. S. Joo^3^, Y. J. Eim^3^, H. J. Yoon^3^, S. Y. Lee^3^, H. N. Youn^3^, Y. S. Oh^3^, E. S. Kim^1,2^

##### ^1^Infection Control Team; ^2^Division of infectious Disease; ^3^SSG(SNUBH Sterile&clean environment Guarantee) TFT, Seoul National University Bundang Hospital, Seongnam, Korea, Republic Of

###### **Correspondence:** E. S. Lee

**Introduction:** An environmental cleaning program on hightouch surfaces in the hospitals is recommended, considering that the spread of microorganisms causing healthcare-associated infections is related to contamination of near-patient surfaces and equipments.

**Objectives:** We have developed activities to improve the environmental cleaning program.

**Methods:** A multidisciplinary team for standardization of environmental cleaning practice was formed and carried out as part of manual, education and evaluation system. The manual was produced in the form of a checklist of 7 places by determining the hightouch surfaces and cleaning frequencies through a literature review. Customized education contents for each job group were developed. After terminal cleaning, its effectiveness were evaluated using adenosine triphosphate (ATP) monitoring method. Below 500 RLU (Relative Light Units) was judged as “Pass”, and the result was fed back to the environmental cleaning service department.

**Results:** A total of 81 hightouch surfaces were evaluated. The pass rate increased from 21.0% to 51.9% after intervention, and the average ATP value decreased (2636 vs. 1089, P <0.0001). The ATP pass rate for all departments has increased and the average ATP value has significantly decreased for the ward, intensive care unit and operation/procedure rooms. Among major hightouch surfaces, the average ATP value decreased most strongly for lamp switch and handwash sink. The level of knowledge of the environmental cleaning service staff has also risen after intervention.

**Conclusion:** Implementation of the standardized protocol for environmental cleaning and an evaluation for its effectiveness using ATP monitoring showed an increase in the degree of environmental cleaning and the level of knowledge of the performers in the hospital.

**Disclosure of Interest**: None declared

## Poster session: Device associated blood-stream infections 2

### P254 OUTCOME OF MRSA CATHETER-RELATED INFECTION IN PATIENTS ON HEMODIALYSIS WITH CENTRAL VENOUS CATHETERS

#### V. Gerasimovska^1^, B. Gerasimovska Kitanovska^2^

##### ^1^Vascular access unit; ^2^University Clinic of Nephrology , Skopje, Macedonia, The Former Yugoslav Republic Of

###### **Correspondence:** V. Gerasimovska

**Introduction:** The incidence of invasive MRSA infection among patients(pts) undergoing chronic dialysis is >100 times higher than in the general population. Increased risk of MRSA infections in dialysis patients is related to repeated vascular access for hemodialysis patients through central venous catheters (CVCs).

**Objectives:** Outcome of patients on hemodialysis with central venous catheters with confirmed MRSA catehter related infections

**Methods:** We collected epidemiological and laboratory data on all cases of MRSA bacteremia at pts on hemodialysis with CVC , from December 31, 2010 to December 31, 2017. Demographic data, medical comorbidities (diabetes),duration of CVC, duration of hospitalization and antibiotic therapy, function and complications were recorded

**Results:** We identified 52 episodes of MRSA bacteremia from 46 patients (24 males, 22 females, aged 57 years).Thirthy nine pts had temporary CVC, and 7 permanent CVC. More than a half of the patients have diabetes, and one third of the pts were on Chronic Hemodialysis Program more than 3 years. There were no differences in age, gender or severity of bacteraemia and comorbidities In logistic regression analysis, variables were duration time of CVC, type of previous venous access, previous use of antimicrobials, and previous hospitalization related to CRB. Previous hospitalization increased the chance of developing CRB, 6.6-fold (CI 95%: 1.9–23.09) All CVC were removed and new ones were inserted. Only one patient died, and two had complications- Spondylodiscites. Vancomycin was most frequently administered antibiotic.

**Conclusion:** All MRSA catheter-related bacteraemia were successfully resolved by changing CVC and appropriate antibiotic therapy. Therefore, prevention activities should focus on improving CVC maintenance.Infection prevention measures for bloodstream infections related to central venous catheter use should be intensified.

**Disclosure of Interest**: None declared

### P255 DECREASING CENTRAL LINE ASSOCIATED BLOOD STREAM INFECTIONS BY REDUCING THE USE OF FEMORAL CENTRAL VENOUS CATHETERS

#### L. Kozita^1^, I. Aharon^1^, A. Peretz^2,3^, O. Nitzan^1,3^, H. Zayyad^1^

##### ^1^Infectious Disease Unit; ^2^Microbiology Lab, Poriya Medical Center, Poriya, ^3^Faculty of Medicine, Bar Ilan university, Zefat, Israel

###### **Correspondence:** L. Kozita

**Introduction:** Insertion of a central venous catheter (CVC) is a common invasive procedure performed in hospitals in order to provide intravenous treatment and hemodynamic monitoring. One of the possible complications of a CVC is a central line associated blood stream infection (CLABSI), which is associated with substantial morbidity and mortality. The CVC insertion site is one of the factors that impacts the incidence of CLABSI-as the femoral site is associated with higher CLABSI rates than the subclavian or jugular sites, especially in obese patients.

**Objectives:** Our aim was to decrease the rate of femoral site insertion of CVCs and the ensuing CLABSI events at our medical center.

**Methods:** This prospective study was conducted from 2015 to 2018 in the 2 internal medicine wards, general intensive care unit (ICU) and cardiac ICU (ICCU) at the Padeh-Poria medical center, a 350 bed general hospital in northern Israel. We constructed a program for decreasing femoral CVCs that included prospective monitoring of CVC insertion, educational staff meetings, instruction of medical staff concerning insertion techniques of subclavian and jugular catheters and use of ultrasound guided CVC insertion, and quarterly yearly feedbacks of femoral CVC insertion rates and CLABSI rates.

**Results:** During the study period there was a decrease in the total rate of femoral CVC insertion: from 92 femoral CVCs out of a total of 173 CVCs in 2015 (53.2%) to 18 out of 92 CVCs in 2018 (19.6%) (p<.001). In internal medicine ward A: from 25 out of 35 (71.4%) femoral CVCs to 3 out of 10 (30%) (p=.05), internal medicine ward B: 38/62 (61.3%) to 12/41 (29%) (p=.02), ICU: 25/58 (43.1%) to 1/16 (6.25%) (p<.001), and in the ICCU from 4/18 (22.2%) to 2/29 (6.9%). The CLABSI rate in the ICU decreased from 5.7 in 2015 to 1.3 in 2018 (a 77% risk reduction) and in the ICCU from 2.7 to 1.5 events per 1000 catheter days (44% risk reduction).

**Conclusion:** A comprehensive plan that included monitoring, instruction of medical staff and periodic feedback resulted in a significant decrease in the rate of femoral CVC insertion and CLABSI rates.

**Disclosure of Interest**: None declared

### P256 INFLUENCE OF THE NUMBER OF CATHETERS PLACED BY A DIALYSIS CENTER ON THE OCCURRENCE OF CATHETER BACTEREMIA ON HEMODIALYSIS

#### N. David^1^, M. Anaïs^1^, R. IAN^1^, B. Vincent^2^, D.-P. SILVINA^3^, G. SOPHIE^4^, L. GILLES^5^, M.-G. XAVIER^6^, S. ANNE^1^

##### ^1^CPIAS AUVERNGE-RHONE-ALPES, SAINT-GENIS-LAVAL; ^2^CH ANNONAY, ANNONAY; ^3^ATIR, AVIGNON; ^4^CH LYON SUD, LYON; ^5^AIRBP, VERNOUILLET; ^6^AGDUC, LA TRONCHE, France

###### **Correspondence:** N. David

**Introduction:** A national surveillance network for acquired hemodialysis infections was set up in 2005. It targets infections and bacteremia associated with access sites. A review of 10 years of surveillance was conducted in 2016 showing that access site infections were globally controlled, while bacteremia was not. Given these results, we hypothesized a possible center effect depending on the number of catheters placed in the occurrence of a bacteremia.

**Objectives:** The aim of this study is to test this hypothesis.

**Methods:** Study based on the national database from 2005 to 2017, including 63 centers with at least one catheter. For the study of the risk factors, we chose to discretize the quantitative variables retained, in binary form on the basis of the cut off thresholds determined by their respective modified ROC curves. The variable "center effect" was constructed by taking into account the number of catheters placed among all access sites (catheters and fistulas) per center. Stepwise logistic regression analysis was performed.The results obtained were presented in adjusted OR form (aOR) and their 95% confidence interval (CI).

**Results:** After adjustment, the factors associated with the occurrence of bacteremia on catheter are:- History of MSSA or MRSA infection (aOR = 1.92 (95% CI [1.35-2.71])- Chinstring (vs. femoral) access site (aOR = 3.31 (95% CI [2.04-5.37])- Recent site change (aOR = 0.69 (95% CI [0.51-0.93]- Installation of a membrane closure plug at laying (aOR = 1.97 (95% CI [1.40-2.77])- At least one site manipulation unrelated to a session (aOR = 1.60 (95% CI [1.21-2.10])- Be in a center with a high catheter rate, defined as having more than 46 catheters per 100 access sites (aOR = 2.24 (95% CI [1.72-2.92]).

**Conclusion:** Our work confirms the results of previous studies concerning the factors associated with the risk of hemodialysis catheter infections. However, we highlight a center effect that confirms the interest of limiting and / or reevaluating indications for catheter placement.

**Disclosure of Interest**: None declared

### P257 BIOFILMS AS A RISK FACTOR FOR THE OCCURRENCE OF HEALTHCARE ASSOCIATED INFECTIONS

#### A. V. Tutelyan, N. G. Sedykh, I. V. Chebotar, V. G. Akimkin

##### Central Researche Institute of epidemiology, Moscow, Russian Federation

###### **Correspondence:** A. V. Tutelyan

**Introduction:** Catheter associated bloodstream infections (CABSI) - occupy the third place among all healthcare associated infections (HAIs) and the first place among the causes of primary bacteremia.

**Objectives:** At present, the most common colonization and infection of the central venous catheter (CVC), occurs through the migration of bacteria from the skin, less often through the external opening of the catheter and through the transfusion of contaminated infusion solutions. The formation of a biofilm on a vascular catheter can occur as early as 24 hours after its production.

**Methods:** At the same time, the introduction of various medicinal substances through CVC, in particular catecholamines, can accelerate the formation of biofilms. The chemical nature of the material from which the catheter is made also plays an important role in biofilm formation. Thus, catheters made of polyethylene and polyvinyl chloride are much more susceptible to the adhesion of microorganisms and the formation of biofilms than catheters made of silicone, teflon and polyurethane.

**Results:** After penetration into the vessel lumen, microorganisms, interacting with the catheter surface, form a biofilm consisting of two phases: a stationary one, consisting of an intercellular polysaccharide matrix and slowly dividing bacterial cells, which after dispersion from the biofilm are in a free-weighted, planktonic form (second phase), are responsible for the development of the clinical symptoms of the infection.

Studying the physical properties of the catheter material, the adhesion processes of microorganisms on them, as well as the dispersion of bacteria from the biofilm composition, the genotype and phenotypic profile of the bacteria forming the biofilm, will reduce the number and severity of infectious complications and reduce the length of hospital stay for patients.

**Conclusion:** The development of a regulatory framework to combat and prevent the formation of biofilms of causative agents of HAIs on abiotic surfaces, as well as clinical standards for catheterization and care for vascular catheters, training medical personnel in these issues will reduce the number of CABSIs that arise and, consequently, reduce the cost of their treatment.

**Disclosure of Interest**: None declared

### P258 A CLUSTER OF SERRATIA MARCESCENS BLOODSTREAM INFECTIONS AMONG HEMODIALYSIS PATIENTS

#### A. Vaturi^1^, S. Masarwa^1^, E. Gavrieli^1^, D. Ben-David^1^, E. Solter^1^, D. Schwartz^1^, J. Lellouche^1^, R. Rov^1^, Y. Carmeli^1,2^, M. J. Schwaber^1,2^

##### ^1^National Center for Infection Control, Israel Ministry of Health; ^2^Tel Aviv University Sackler Faculty of Medicine, Tel Aviv, Israel

###### **Correspondence:** A. Vaturi

**Introduction:** *Serratia marcescens* is an opportunistic pathogen with potential to cause severe nosocomial infections with significant morbidity and mortality. In March 2019, the Ministry of Health received notification about 5 dialysis patients who developed *Serratia marcescens* bloodstream infection (BSI) within the previous two weeks..

**Objectives:** To describe the investigation and outbreak control

**Methods:** The epidemiologic investigation included a site visit in the nephrology clinic and interviews with staff. Microbiological testing was performed on samples taken from medications and fluids, surfaces, water systems and the involved health care professionals. Clonality was assessed with repetitive sequenced-based PCR (rep-PCR) and FT-IR spectroscopy.

**Results:** Between March 10 and March 22, 45% of patients (5/11) with tunneled central lines developed *Serratia marcescens* BSI. None of the patients who underwent dialysis via graft or fistula developed bacteremia. Interview of staff members revealed that central lines are routinely flushed with a heparin lock solution. The heparin flush is withdrawn from a common saline bag into which the contents of a multi-dose heparin vial are injected. *Serratia marcescens* was isolated from the sink. Cultures from medications, fluids and healthcare workers’ hands did not grow *Serratia*. All 5 human isolates belonged to a single clonal type. The sink isolate belonged to a different serotype. Following intervention, which included cleaning and disinfection of the dialysis unit, discarding of open solutions and implementation of a recommendation to use single-use heparin lock solution for line flush, the outbreak ceased

**Conclusion:** An outbreak of *Serratia marcescens* in hemodialysis patients was associated with multi-patient use of a heparinized saline solution, highlighting the risk of using multi-dose flush solutions and the importance of adherence to strict aseptic technique when preparing intravenous drugs. An environmental isolate of *Serratia marcescens* was proven unrelated to the outbreak, highlighting the importance of genotypic analysis in outbreak investigations.

**Disclosure of Interest**: None declared

### P259 COMPARATIVE STUDY OF LEAKAGE FROM MALE LUER CONNECTORS ON INFUSION SETS AFTER DISCONNECTION FROM STANDARD FEMALE HUBS AND NEEDLE FREE CONNECTORS

#### C. Mide^1^, E. Lingaas^2^, J. G. Schön^1^

##### ^1^Product Development, ConceptoMed AS, Ballstad; ^2^Head of Department of Infection Prevention, Oslo University Hospital, Oslo, Norway

###### **Correspondence:** C. Mide

**Introduction:** Unintended fluid spill during connection to and disconnection from hubs/connectors is well known in different clinical settings. Leakage from connectors may increase the risk of fluid pathway contamination and catheter related bloodstream infections, as well as exposure of personnel and the environment to drugs, e.g. antibiotics or other potentially harmful substances.

**Objectives:** The purpose of the study was to evaluate fluid leakage from clamped infusion gravity sets with male luer connectors during and after disconnection from different female luer hubs in a controlled setting.

**Methods:** The study compared a male luer lock connector designed to close flow prior to disconnection (ThorQ™ Mini CTRL FlowStop™) with standard male lock luer connectors on infusion sets with roller-clamp, after disconnection from ordinary stop-cock hubs and three different needle-free connectors. Measurement of leakage was performed as follows: 1. Closing off the stop-cock downstream. 2. Closing the roller clamp upstream. 3. Disconnection. 4. Collection of spontaneous leakage over 30 seconds. 5. Finalizing with a standardized hammer punch on the male connector to release excess fluid into the sample bag. Collected fluid leakage was weighed and analyzed with Paired T-test, 95%CI, between the test/control group pairs.

**Results:** 640 samples were collected and weighed. Gravity infusion sets with standard male luer lock connectors allow fluid to leak into the female hub and out of the tube during and after the disconnection (average 0.106 mg). Two of the three needle-free connectors caused significantly increased leakage, compared to the female stop-cock hub. When using a male luer lock connector designed to close flow prior to disconnection in combination with needle-free connectors, there was a statistically significant 99.94% reduction of fluid spill compared to using the needle-free connectors in combination with a standard male luer lock connector.

**Conclusion:** With variation in clinical habits related to clamping and inherent male connector design issues, it is reasonable to conclude that the test device has potential to prevent most spontaneous fluid spill after luer disconnection. Thus, it may significantly reduce overall spill of antibiotics and other harmful infusates as well as potentially reduce the risk of intraluminal contamination.

**Disclosure of Interest**: C. Mide Employee of: ConceptoMed AS, Shareholder of: ConceptoMed AS, E. Lingaas: None declared, J. Schön Employee of: ConceptoMed AS, Shareholder of: ConceptoMed AS

### P260 RISK OF CENTRAL VENOUS VERSUS HEMODIALYSIS CATHETER-RELATED BACTEREMIA IN INTENSIVE CARE UNITS 2011-2017

#### A. Savey^1^, A. Machut^1^, A. Berger-Carbonne^2^, F. L'Hériteau^3^, I. Russell^1^, V. Stoeckel^4^, J.-F. Timsit^5^, P. Vanhems^6^, A. Lepape^6^ on behalf of REA-RAISIN study group

##### ^1^CPias Auvergne-Rhône-Alpes, Saint-Genis-Laval; ^2^Santé publique France, Saint-Maurice; ^3^CPias Ile-de-France, Paris; ^4^CPias Grand-Est, Reims; ^5^Hôpital Bichat - Université Paris Diderot - IAME : UMR1137, Paris; ^6^Hospices Civils de Lyon, Lyon, France

###### **Correspondence:** A. Savey

**Introduction:** Since 2011, the French national surveillance network of intensive care units (ICU)-acquired infections added venous hemodialysis catheters (HC) to central venous catheters (CC) to measure the respective risk of catheter related bacteremia (H-CRB or C-CRB).

**Objectives:** The objective is to compare the rate of both types of CRB and their trends over 7 years.

**Methods:** Two databases were constructed with Patients "with one or more HC" or "with one or more CC" from 2011 to 2017. A descriptive analysis was performed, then a Stepwise logistic regression to determine the independent risk factors (IRF) of C-CRB or H-CRB, and to compare them.

**Results:** 208,353 patients with CC and 36,668 with HC were included.

Patients with HC were more severe (higher SAPSII, longer stay, more transferred from another ICU, immunocompromised, medical reason for admission). The mean duration of exposure was the same for CC and HC (11.2 vs 11.3d).

We observed 1295 C-CRB and 268 H-CRB after a mean time of catheter insertion of 13.9d vs 11.9d (p = 0.004).

The most common micro-organisms of the H-CRB vs C-CRB were: *S. aureus* (21.1 vs 16.9%), *P. aeruginosa* (12.9 vs 8.6%) and *S. epidermidis* (11.0 vs 19.0%). *E. coli* was more common on H-CRB (7.6 vs 3.9%), and *C. albicans* on C-CRB (7.8 vs. 5.7%).

In the final model, CRB-IRF were:

- favoring: duration of catheterization (>4d.), transfer from another ICU.

- protector: antibiotic at admission, male (for H-CRB), age>77y, surgical patient (for C-CRB).

Over 7 years, after adjustment on these IRF, the rate of H-CRB was higher (2.77/1000HDC-d) than C-CRB (2.36 /1000CVC-d) (OR = 1.18 [1.04-1.35]).

Both annual adjusted rates significantly decreased between 2011 and 2017 (H-CRB: 3.48 to 2.23/1000HC-d; C-CRB: 2.91 to 1.98/1000CC-d). It was higher for H-CRB in 2014 (p = 0.05) and 2015 (p = 0.04).

**Conclusion:** Patients with HC were more severe, but the CRB-IRF little differed between CC and HC. The onset of H-CRB was earlier, with a higher adjusted rate. A significant decrease in CRB incidence over 7 years was observed for both CC and HC. The use of a hierarchical model taking into account the characteristics of catheters and units will complete this study.

**Disclosure of Interest**: None declared

### P261 CATHETER SECUREMENT IMPACT ON PICC-RELATED CLABSI: DOES SECUREMENT EFFECT RISK?

#### M. S. Rowe^1^, T. Spencer^2^

##### ^1^Vascular Access, University of Arkansas for Medical Sciences, Little Rock; ^2^Director, Global Vascular Access, LLC, Cary, United States

###### **Correspondence:** T. Spencer

**Introduction:** Can a PICC securement device provided a significant lower risk of overall harm in terms of catheter dislodgment, migration or malposition, alleviating the potential risks to develop catheter-related thrombosis and device-related infection?

**Objectives:** CLABSI is a large global concern that often leads to higher morbidity and mortality in hospitalized patients and finding ways to further reduce risk and identify causal factors is crucial to improve quality of healthcare. All factors including patient diagnosis and insertion related factors, were scrutinized to rule out insertion related risk factors. Factors related to care and maintenance of lines was also assessed and equivalent in the two groups over the 4-year period with exception of the securement type.

**Methods:** A single center, 4-year, retrospective review performed at a large academic medical center with the use of a subcutaneous securement device and adhesive stabilization in 7,779 PICC procedures to establish the correlation and impact of catheter stabilization and securement methods to central line associated bloodstream infection (CLABSI).

**Results:** This quality data analysis process found that a subcutaneous securement device offered a reduction of overall risk of 49-51% for central line associated bloodstream infection rates assessment over the adhesive securement devices. Risk reduction was consistent over the four years, highlighting that improved catheter stability can lead to lower infection rates by reducing intravascular bacterial ingress through subcutaneous tissue when the catheter is stabilized from the point of implantation to explant without catheter movement.

**Conclusion:** The use of a subcutaneous securement device also provided a significant lower risk of overall harm in terms of catheter dislodgment, migration or malposition, alleviating the potential risks to develop catheter-related thrombosis and device-related infection.

**Disclosure of Interest**: M. Rowe Speaker's bureau of: Interrad Medical, Inc, Consultant for: Interrad Medical, Inc / Smiths Medical, Inc, T. Spencer Speaker's bureau of: Teleflex, LLC / FujiFilm SonoSite, Inc, Consultant for: Interrad Medical, Inc

### P262 SECULAR TRENDS AND EPIDEMIOLOGY OF PERIPHERAL VENOUS CATHETER-RELATED BACTEREMIA AT A UNIVERSITY HOSPITAL OVER A 15-YEAR PERIOD

#### A. Hornero^1^, E. Jimenez-Martinez^1^, P. Saliba^2^, G. Cuervo^3^, I. Grau^3^, D. Berbel^4^, C. Ardanuy^4^, J. Carratala^3^, M. Pujol^3^

##### ^1^Infection Control, BELLVITGE UNIVERSITY HOSPITAL; ^2^ICoordinating Center , VINCAT PROGRAM; ^3^Infectious Diseases; ^4^Department of Microbiology, BELLVITGE UNIVERSITY HOSPITAL, Hospitalet Llobregat, Spain

###### **Correspondence:** A. Hornero

**Introduction:** Peripheral venous catheter (PVC) is the device most often used in hospitalized patients. The ECRI institute has listed PCV-related bacteremia among the top ten causes of concern regarding patients’ safety. PVC-related bacteremia is often ignored, underreported, and frequently underrecognized as a cause of serious infection.

**Objectives:** To determine secular trends and changes in the epidemiology of PVC-related bacteremia over a 15 year-period in a hospital with a comprehensive vascular-catheter infection control program.

**Methods:** Surveillance of PVC-related bacteremia was carried-out through daily meetings with members of the microbiology department and the infection control team in a 700-bed university hospital in Barcelona, Spain. Patients with positive blood cultures were visited to assess the source of bacteremia and to administrate antibiotic treatment. Changes in the epidemiology of PVC-related bacteremia were determined comparing 2 periods: 2004-2011 (first period) and 2012-2018 (second period).

**Results:** A total of 264 episodes were included; 193 (73%) during the first period and 71 (27%) during the second one. Cumulative incidence was 0.10/1.000 patient-days during the first period and 0.04/1.000 patient-days during the second (p<0.001). No significant differences between periods were observed regarding gender, median age, and median days from PVC insertion to bacteremia, causative agents, median Charlson score, and 30-days mortality.

**Conclusion:** PVC-related bacteremia is a significant cause of health-care related infection. Through a comprehensive infection control program, we have achieved a significant reduction in the incidence of PVC-related bacteremia, although epidemiology, etiology, and outcomes did not vary significantly between the study periods.

**Disclosure of Interest**: None declared

### P263 NOSOCOMIAL BLOODSTREAM INFECTION RATES: EXPLORATION OF A QUALITY INDICATOR FOR INFECTION PREVENTION

#### I. Spijkerman^1^, B. Laan^2^, W. de Rond^1^

##### ^1^Medical Microbiology; ^2^Internal Medicine, Amsterdam UMC, Amsterdam, Netherlands

###### **Correspondence:** I. Spijkerman

**Introduction:** Outcome indicators for the quality of infection prevention in hospitals are scarce and time consuming to collect. In the Netherlands, the proposed rate of bloodstream infections (BSI) by highly resistant microorganisms (HRMO) is so low that it has no use as a tool for focussing interventions and comparison between hospitals and wards.

**Objectives:** To monitor nosocomial BSI rates during a 3-year surveillance period and evaluate its use as an indicator for infection prevention.

**Methods:** Between January 2016 and January 2019, we conducted an observational, prospectieve surveillance in a tertiary teaching university hospital. We collected data on positive blood cultures in patients admitted to the hospital for more than two days from the Laboratory of Bacteriology. Data on source of BSI, ward specialism and other patient characteristics were collected from the electronic medical records. Rates of BSI were calculated as number of BSI per 1000 patient days.

**Results:** The overall prevalence of nosocomial BSI was 2.33 per 1000 patient days. The rates varied between 2.04 in 2016, 2.14 in 2017 and 2.92 nosocomial BSI in 2018. The increased rate in 2018 is probably due to a quality improvement project to increase adherence of taken blood cultures before starting antimicrobial therapy, and due to a decrease in patient days for the hospital. The most common sources of BSI were (central) line-associated bloodstream infections (35.9%) and abdominal infections (18.2%). Coagulase-negative staphylococci (27.4%) and Enterobacteriaceae (23.7%) were the most common pathogens. The rate of HRMO was stable over time (5.1%). Rates of nosocomial BSI varied considerably between wards and specialisms. In addition, the distribution between sources of BSI is variable for different wards. Time trends of nosocomial BSI are shown per ward and can be used monitoring. The department of Infection Prevention spent 2.5 hours per week on data collection.

**Conclusion:** Nosocomial BSI rate is a solid, inexpensive and easy to perform outcome indicator for infection prevention in our hospital. Surveillance of nosocomial BSI revealed objective data on clinically relevant infections concerning all specialisms and wards. Variation of nosocomial BSI rates between wards and over time can focus infection prevention interventions.

**Disclosure of Interest**: None declared

### P264 CATHETER-RELATED BLOODSTREAM SURVEILLANCE AND A PATIENT SAFETY PROGRAM: EARLY JOINERS AND UNOBSERVED IMPROVEMENT.

#### T. van der Kooi, E. Smid, J. Wille, S. de Greeff

##### Centre for Infectious Disease Control, National Institute for Public Health and the Environment (RIVM), Bilthoven, Netherlands

###### **Correspondence:** T. van der Kooi

**Introduction:** Dutch hospitals can voluntarily monitor central venous catheter-related bloodstream infection (CRBSI) in the PREZIES national surveillance since 2002. A patient safety (‘VMS’) program, including a CRBSI bundle, started in 2009, which boosted participation and was associated with a CRBSI reduction.

**Objectives:** Evaluation of trends in CRBSI rates during and after the program.

**Methods:** The surveillance includes patients > 18 years, with short term CVCs. We evaluated trends in data from 2009 up to 2018 (not complete) with Cox regression, with hospital as fixed variable and accounting for clustering at patient level by using robust covariance estimation. We stratified for year of entry.

**Results:** Participation increased from 9 in 2009 to 43 in 2014, to decrease thereafter to 28 in 2017, in total 58 (75% of all Dutch hospitals).When evaluating all hospitals the hazard ratio (HR) for 2010 compared to 2009 (reference year) was 0.39 (95% confidence interval (CI) 0.22-0.68) and only slightly less thereafter (range 0.23-0.43). A closer evaluation demonstrates that hospitals that started in 2009 (‘early joiners’) had a high average CRBSI rate (5.0/1000 CVC days), which decreased directly thereafter whereas hospitals that started one or more years later had lower CRBSI rates (2.2-3.5/1000) from the start. Here the HR associated with one year of participation ranged between 0.57 (95%CI 0.29-1.10) and 2.15 (1.13-4.07).

**Conclusion:** During 2009-2018 the incidence of CRBSI in The Netherlands declined. Hospitals that joined the surveillance one or more years after the start of the patient safety program, had relatively low rates already. Although we cannot attribute the decline to the national patient safety program based on these data, the findings might indicate that hospitals already improved their practices while being aware of and preparing for the program(1).

**References**

1. Bion J, Richardson A, Hibbert P, Beer J, Abrusci T, McCutcheon M, et al. 'Matching Michigan': a 2-year stepped interventional programme to minimise central venous catheter-blood stream infections in intensive care units in England. BMJ quality & safety. 2013;22(2):110-23.

**Disclosure of Interest**: None declared

### P265 VALIDATION OF THE CATHETER-RELATED BLOODSTREAM INFECTIONS´ DATA AMONG THE HOSPITALS OF THE VINCAT PROGRAM

#### P. Saliba^1^, O. Gasch^2^ on behalf of Group of bacteremia and infections of vascular catheters-VINCat, E. Calbo^3^, J. contreras^4^, E. Limon^5^

##### ^1^VINCat, Institut Català d'Oncologia; ^2^VINCat, Hospital Parc Taulí de Sabadell; ^3^VINCat, Hospital Universitari Mutua Terrassa; ^4^VINCat, Hospital de la Santa Creu i Sant Pau; ^5^VINCat, Institut Català d'Oncologia, Barcelona, Spain

###### **Correspondence:** P. Saliba

**Introduction:** During the last three decades, the surveillance of healthcare associated infections (HAIs) has been recognized as the cornerstone of an effective program of prevention and control of HAIs. Catheter-related bloodstream infections (CRBSIs) are one of the most common types of HAIs. In 2018, the VINCat program (vigilància de les infeccions nosocomials als hospitals de Catalunya) conducted a national validation of CRBSIs´ data to validate the actual recorded rates and assess the data concordance between VINCat and the participating hospitals.

**Objectives:** To validate the declared CRBSIs data of the hospitals participating in the VINCat program

**Methods:** This validation included 44 participating hospitals divided into 3 main groups according to bed number. All hospitals were asked to submit microbilogical data lists of all registered cases of CRBSIs with Staphylococcus aureus (S.aureus) and Coagulase-negative staphylococci (CoNS) reported to the VINCat during the cut period (March to December ) and were subject for validation. Main outcomes were false negative cases of CRBSIs that should have been declared to VINCat and false positive cases that do not comply VINCat criteria for a CRBSI.To evaluate the agreement between the two validators statistically, the Cohen's Kappa value is used with “1” being the highest value of concordance.

**Results:** The total number of validated cases of bacteremia was 2327, of which 585 were cases of CRBSIs. In total, 89 (5%) were discordant cases of which 2 cases (2%) were not declared, 4 cases (5%) did not meet the VINCat criteria and 83 cases (93%) were not shown in the list of microbiology provided by hospitals. The Kappa value between the two validators was 0.92.

**Conclusion:** This validation shows a high level of concordance between the CRBSIs´data declared to the VINCat program and the participating hospitals up to 96% (almost perfect). Therefore, the CRBSIs ´data within the VINCat program are reliable and eligible for the benchmarking and for public declaration.

**References**

**Disclosure of Interest**: None declared

## Poster session: Surgical site infection: Intervention to reduce the burden 2

### P266 SUSPECTED OUTBREAK OF POST-CESAREAN SURGICAL SITE INFECTION: INVESTIGATION, INTERVENTION AND CONCLUSIONS

#### A. S. LAMEIRAS_ AZEVEDO, C. M. BENITO_MIRALLES, M. J. MOLINA_GOMEZ, G. CABRERA_TEJADA, N. ALGADO_SELVES, V. SOLER_MOLINA, M. FUSTER_PÉREZ, J. SANCHEZ_PAYA

##### PREVENTIVE MEDICINE, GENERAL HOSPITAL OF ALICANTE, ALICANTE, Spain

###### **Correspondence:** A. S. LAMEIRAS_ AZEVEDO

**Introduction:** The surgical site infection is one of the most frequent healthcare associated infections, including post-cesarean wound infection which is a cause of prolonged hospital stay and a burden to the healthcare system.

In july 2018, our department was notified of a suspected increase in the number of post-cesarean surgical infections, in which we took action.

**Objectives:** Describe the evaluation of the situation, the intervention carried out and the outcomes.

**Methods:** An epidemiological investigation was conducted. Simultaneously, an intervention was performed, by providing informative sessions regarding the prevention and control of infections programs, and by presenting a preliminary report. Finally, the periods pre and post-intervention (1 April to 20 July and 21 July to 31 October) were compared to detect risk factors.

**Results:** A total of 229 cesareans were included (115 in the pre-intervention period; 114 in the post-intervention period), and no outbreak was detected. Nevertheless, there was a decrease in the surgical site infections in the post-intervention period from 11,3% to 4,4% (OR 0.4, 95% CI 0.1 – 1.0), with the verification of the change to chlorhexidine at 2% for the preoperatory skin preparation. The lack of antibiotic prophylaxis when indicated was identified as a risk factor for infection (OR 7.3, 95% CI 1.5 – 37.0).

**Conclusion:** Although no outbreak was detected, we identified modifiable risk factors related to healthcare activities. By improving the compliance of the control and prevention of infections programs, we were able to diminish the impact of these factors and decrease the surgical site infections. Nonetheless, scope to improve remains and, as shown by this study, relies on the contribution of the healthcare professionals.

**Disclosure of Interest**: None declared

### P267 PROLONGED ANTIBIOTICS PROPHYLAXIS AFTER CARDIOVASCULAR SURGERY AND ITS EFFECT ON SURGICAL SITE INFECTIONS

#### S. S. Samsudin^1,2^, S. S. L. Goh^1^, S. Saaibon^1,2^, R. Zhazali^1^, R. Ramli^2^, N. Hashim^3^, F. Zainal ^3^, A. Amin^4^, M. Kumaran^4^, I. Azmi^4^, Z. Zulkifli ^4^, S. Krishnasamy^4^, S. Hashim^4^, A. Mokhtar^4^, S. Ponnampalavanar^1,5^

##### ^1^Infection Control; ^2^Nursing; ^3^Anesthesia; ^4^Surgery, University Malaya Medical Centre; ^5^Medicine, University Malaya, Kuala Lumpur, Malaysia

###### **Correspondence:** S. S. Samsudin

**Introduction:** Surgical site infections (SSIs) following coronary artery bypass graft (CABG) procedures pose additional burden on patients and healthcare system. Evidence supports that prolonged surgical antibiotic prophylaxis (SAP) ≥48 hours is ineffective in reducing SSI, it is still a common practice.

**Objectives:** To determine the incidence of SSIs and associated risk factors in patient who underwent CABG in University Malaya Medical Centre (UMMC).

**Methods:** Patients who had undergone CABG in UMMC from January 2017 to December 2018 were included and were prospectively followed up by the infection control nurse for 90 days post operation using electronic medical records (EMR). Data was collected using a standardized form and analyzed using SPSS version 20.

**Results:** A total of 260 patients were reviewed. Majority were male (84.2%) with a median age of 61 years. Patients diagnosed with SSIs were 45(17.3%). Of these, 22(48.9%), 18(40.0%), 4(8.9%) and 1(2.2%) had saphenous vein graft (SVG), sternum, both SVG and sternum and radial graft infections respectively. On univariate analysis, older age, smokers, primary surgeon A and duration of SAP ≥48 hours were associated with increased risk of SSI infections after CABG surgery. On multivariate analysis, only duration of SAP ≥48 hours was associated with increased risk of SSI (OR 7.979, 95% Cl 0.029-0.537). 75.7% (197/260) patients were on prolonged SAP.

**Conclusion:** The rate of SSI post CABG was high and prolonged used of SAP is frequently practiced in UMMC. Our study found that prolong SAP did not prevent SSI. Interventions directed towards increasing awareness and education of prescribes on appropriate SAP use is needed to ensure compliance to guidelines.

**Disclosure of Interest**: None declared

### P268 SURGICAL ANTIBIOTIC TREATMENT IN ORTHOPAEDIC PROTHETIC SURGERY : OVERWEIGHT VERSUS SEVERE OBESITY, SAME DOSE OF CEFAZOLIN ?

#### P. Fascia^1^, D. Narbey^1^, L. Azoeuf^2^, M. E. Gengler^1^, A. Savey^1^, R. Badet^3^

##### ^1^CPIAS Auvergne Rhône-Alpes, CHU LYON, HOPITAL H. GABRIELLE, Saint Genis Laval; ^2^EOH; ^3^Orthopaedic surgery unit, Clinique St Vincent de Paul, Bourgoin Jallieu, France

###### **Correspondence:** P. Fascia

**Introduction:** Because of an increase in the number of surgical site infections (SSI) in a surgical clinic, a case control study was conducted to determine risk factors of those SSI. These SSI concerned orthopedic surgery of hip and knee.

**Objectives:** Describe SSI and Identify risk factors of SSI that occured in the surgical clinic.

**Methods:** Total hip (THP) or knee prosthesis (TKP) infections occurring between 1^st^ January, 2013 and 31^st^ December, 2017 were included. For each case, 2 controls were randomly paired by year of surgery and type of surgery. Risk factors were determined by stepwise conditional logistic regression including variables after univariate analysis with a p-value less than 0.20. Univariate analysis determined Odds Ratios (OR) and conditional logistic regression determined adjusted Odds Ratios (AOR). Weight is analyzed equally as a continuous variable (Body Mass Index, BMI) or as a discontinuous variable (obesity, defined as a BMI over than 30).

**Results:** A total of 34 SSI were identified, 15 THP and 19 TKP. Sixty eight controls were carried out randomly. Among the 38 bactericidal agents isolated, 29 were *Staphylococci* (22 *Staphylococcus aureus, SA*). All the SA strains were serotyped and were different. Risk factors of SSI are male gender (OR=4.9, 95% CI [1.77–13.46], p=0.0022), BMI (OR=1.12, 95% CI [1.02-1.23], p=0.016), obesity (OR=3.11, 95% CI [1.25-7.73], p=0.014), rank of surgery (OR=1.4, 95% CI [1.08-1.86], p=0.001) and loco-regional anesthesia (OR=3.05, 95% CI [1.12-8.28], p=0.029). Logistic regression identified male gender (AOR=4.21, 95% CI [1.48-11.96], p=0.007), BMI (AOR=1.12, 95% CI [1.01-1.23], p=0.028) or obesity (AOR=3.09, 95% CI [1.11-8.6], p=0.03) as risk factors of SSI.

**Conclusion:** French guidelines of surgical prophylactic antibiotic treatment recommend that the dosage of cefazolin needs to be doubled in the case of BMI over 35 or weight over 100 kg. This case control study conjectures about doubling the dosage of cefazolin as soon as BMI is over 30 or possibly even 25, or adequately adapting surgical antibiotic treatment to weight.

**Disclosure of Interest**: None declared

### P269 BRINGING TO LIFE THE PREVENTION OF SURGICAL SITE INFECTIONS IN HEALTH CARE FACILITIES – A NEW IMPLEMENTATION APPROACH

#### C. Kilpatrick^1^, T. Weiser^2^, J. Solomkin^3^, B. Allegranzi^1^

##### ^1^World Health Organization, Geneva, Switzerland; ^2^Stanford University School of Medicine, Stanford; ^3^World Surgical Infection Society, Cincinnati, United States

###### **Correspondence:** C. Kilpatrick

**Introduction:** The World Health Organization (WHO) strongly recommends multimodal improvement strategies for effective implementation of infection prevention and control (IPC) interventions. This builds upon the scientific evidence gathered about hand hygiene improvement, focusing on the integration of five elements (system change, education and training, monitoring and feedback, reminders and communications, and institutional safety climate) that are adaptable to other IPC contexts. This approach was adapted for the implementation strategy of the WHO Global guidelines on the prevention of surgical site infection (SSI).

**Objectives:** To outline the steps taken to develop a WHO SSI prevention implementation manual.

**Methods:** A desk exercise was undertaken to critique the relationship between the WHO improvement strategy for hand hygiene with SSI interventions. Collation of experiences from the field and a review of the concept was undertaken by a virtual engagement exercise with 18 experts between July and December 2019.

**Results:** This first of its kind global implementation manual features an approach used for each one of the WHO 29 SSI prevention recommendations, framed within the five-element multimodal strategy and within the context of a broader surgical safety climate. It outlines a range of implementation and practical steps that reflect real-life experiences. It proposes a cyclic framework for rolling out a SSI prevention programme to ensure that the entire facility is ready to undertake the intervention/change required based on a preparation step, a baseline situation analysis, planning, impact measurement and sustainability evaluations.

**Conclusion:** This operational, adaptable manual is a comprehensive resource for countries and healthcare facilities and fills an implementation gap in this field. It has the potential to influence improvement of IPC practices and SSI reduction over time. Other important achievements will be improved SSI knowledge and awareness among the key players.

**Disclosure of Interest**: None declared

### P270 Withdrawn

### P271 WHICH IS A BETTER SOLUTION FOR OPERATING ROOM VENTILATION - LAMINAR AIRFLOW OR MIXING VENTILATION?

#### G. Cao^1^, C. Pedersen^1^, A. Radtke^2,3^, H. Langvatn^4^, L.-I. Stenstad^5^, J. G. Skogås^5^

##### ^1^Department of Energy and Process Engineering; ^2^Department of Clinical and Molecular Medicine, Norwegian University of Science and Technology; ^3^Unit for Infection Control; ^4^Clinic for Surgery; ^5^Operating Room of the Future, St. Olavs hospital, Trondheim University Hospital, Trondheim, Norway

###### **Correspondence:** A. Radtke

**Introduction:** The air quality of the operating microenvironment may be an important contributor to prevent surgical site infections (SSIs). Both mixing ventilation (MV) systems and laminar air flow (LAF) systems have been used in operating rooms (ORs) to tentatively reduce the CFU (Colony Forming Unit) density. The recently published WHO guideline suggests that LAF systems should not be used to reduce the risk of SSI for patients undergoing total arthroplasty surgery, but the conclusion is disputed and based on low to very low quality of evidence. The OR contains numerous transient phenomena that may cause significant changes to the time resolved indoor air distribution patterns.

**Objectives:** The objective of this study was to compare the performance of laminar airflow systems and mixing ventilation in operation rooms.

**Methods:** Measurements of CFU and experimental measurements of airflow distribution were performed in different orthopedic operating rooms equipped with a LAF and MV systems at St. Olavs hospital.

**Results:** Under many operating conditions, the airflow distribution from the laminar airflow system was disrupted more than with mixing ventilation method. **Lower CFU levels** may be achieved in ORs with Mixing ventilation when using proper surgical clothing with lower activity intensity.

**Conclusion:** This study showed a complex relationship between the local indoor air quality and airflow distribution in the operating microenvironment of a lying patient. Air distribution changed significantly under various conditions involving the presence of different heat sources, including surgical lamps, the patient, surgical staff and various monitors in the orthopedic OR. Our study indicates that LAF may be disturbed under operating conditions. However, this does not seem to increase CFU levels around the surgical site.

**Disclosure of Interest**: None declared

### P272 SEARCHING THE IDEAL SPOT: AN AERODYNAMIC SIMULATION STUDY FOR THE POSITIONING OF A DECONTAMINATION-RECIRCULATION SYSTEM IN THE OPERATION ROOM

#### G. Messina^1^, G. Spataro^2^, L. Catarsi^3^, F. De Marco^4^, A. Grasso^4^, G. Cevenini^2^

##### ^1^Department of Molecular and Developmental Medicine, University of Siena Italy; ^2^Department of Medical Biotechnologies, University of Siena, Italy; ^3^Department of Molecular and Developmental Medicine, Post Graduate School of Public Health, University of Siena, Italy, ^4^Medical Management, “Le Scotte” Teaching Hospital, Siena, Italy

###### **Correspondence:** G. Messina

**Introduction:** Surgical Site Infections (SSI) are associated with a rise in mortality, hospital stay duration, and medical costs. In Operating Rooms (OR), air ventilation and filtration are important factors in preventing SSIs, but to identify how precisely variations in the ventilation and filtration systems influence air quality in the OR requires specific and tailor-made measures and analyses.

**Objectives:** The purpose of this study is to explore how two different positions of a mobile air filtering system affect air quality in the OR.

**Methods:** We conducted a descriptive study in April-May 2018 in the Teaching Hospital of Siena (Italy). The inflow and outflow air ventilations of an OR were measured, together with a novel mobile air decontamination-recirculation system with a high efficiency particulate air filter and a patented crystalline ultraviolet C reactor. The Solidworks 2017 3D software was used to simulate the OR and the airflows interactions based on the measured experimental data. The device was simulated: 1) next to the main OR entrance with its airflow facing the surgical bed; 2) next to the surgical bed, with its airflow facing the main OR entrance.

**Results:** Based on our simulation results, the device interferes with the main flow generated by the ceiling vents and creates a low pressure effect, dragging the air also towards possibly contaminated surfaces. To locate the device with its air intake next to the OR door means to facilitate the entrance of particulate into the OR, enhancing the risks of contamination due to the opening of the door and reducing the effects of the OR positive pressure. We modeled an orientation of the device intake of air towards the surgical table, in order for the device airstream to not cause any possible contamination and to hamper the entry of contaminants in a door opening simulation.

**Conclusion:** Extra flows of a mobile air filtering device can significantly affect the air dynamics in an OR. Ad hoc environmental measures are needed in order to determine its ideal positioning to maximize the effects in potentially preventing SSIs.

**Disclosure of Interest**: G. Messina Grant/Research support from: Aerobiotix, G. Spataro: None declared, L. Catarsi: None declared, F. De Marco: None declared, A. Grasso: None declared, G. Cevenini: None declared

### P273 AIRBORNE PARTICLES AND A SURGICAL SITE INFECTIONS: EVALUATION OF A DECONTAMINATION-RECIRCULATION DEVICE AS A PREVENTIVE MEASURE

#### G. Messina^1^, G. Spataro^2^, L. Catarsi^3^, F. De Marco^4^, A. Grasso^4^, G. Cevenini^2^

##### ^1^Department of Molecular and Developmental Medicine, University of Siena Italy; ^2^Department of Medical Biotechnologies, University of Siena, Italy; ^3^Department of Molecular and Developmental Medicine, Post Graduate School of Public Health, University of Siena, Italy, ^4^Medical Management, “Le Scotte” Teaching Hospital, Siena, Italy

###### **Correspondence:** G. Messina

**Introduction:** Hospital Acquired Infections (HAI) are a severe public health problem and also represent a high economic burden. About $40 billion have been spent annually for HAIs in hospitals in the US and EU, and Surgical Site Infections (SSI) in particular are a relevant part of this spending. It is well known that bacteria responsible for SSIs can also be carried by air particulate in the room.

**Objectives:** Our goal in this study is to investigate whether a mobile unit for air particle filtering in the operating room (OR) can improve the environmental airborne conditions.

**Methods:** A cross sectional study was conducted in March 2018 in Siena’s Hospital (Italy). We tested a novel air decontamination-recirculation system unit provided with a patented crystalline ultraviolet C (C-UVC) reactor and HEPA filter, that could be moved around in an OR. In order to evaluate the environmental contamination, air samples were taken in 4 spots of an OR’s periphery during a surgical intervention, and at each spot 4 different phases have been considered: I) Illuvia off and OR at rest; II) Illuvia off and OR in operational; III) Illuvia on and OR in operational; IV) Illuvia off and OR in operational. The evaluation was performed through particle counting of six different sizes: 0.3, 0.5, 1.0, 3.0, 5.0, and 10 μm. The statistical analysis used the Wilcoxon rank test, and was carried out with the statistical software Stata 12, setting the significant level at p<0.05.

**Results:** Particulate matter of all sizes had fallen significantly (p<0,05) passing from phase II (Illuvia off and OR in operational) to phase III (Illuvia on and OR in operational). Switching the device off, particles of 3,0 μm or smaller were statistically significantly higher (p<0,05).

**Conclusion:** Particulate matter stayed significantly lower during the functioning of the device in an OR in operational. This could reduce the risk of SSI.

**Disclosure of Interest**: G. Messina Grant/Research support from: Aerobiotix, G. Spataro: None declared, L. Catarsi: None declared, F. De Marco: None declared, A. Grasso: None declared, G. Cevenini: None declared

### P274 EFFICACY OF TEMPERATURE CONTROLLED AIRFLOW (TCAF) VENTILATION IN THE OR TO REDUCE SURGICAL SITE INFECTIONS

#### S. Vasiuk^1^, Y. Vasylchyshyn^1^, V. Vasyuk^2^, C. Bulitta^3^

##### ^1^“Swedish-Ukrainian clinic “Angelholm”” Ltd; ^2^Bukovinian State Medical University, Chernivtsi, Ukraine; ^3^Institute of Medical Engineering, Technical University of Applied Sciences Amberg-Weiden, Weiden, Germany

###### **Correspondence:** C. Bulitta

**Introduction:** The current recommendations of the WHO and the German KRINKO for the prevention of surgical site infections do not see a compelling proof for the use of ultraclean airflow ventilation technologies like low-turbulence displacement flow (TAV). However, for this technology and innovative solutions like temperature controlled airflow (TcAF) there still is a lack of data to assess the impact on infection rates.

**Objectives:** The aim of the study was to evaluate the impact of operating room ventilation technology on clinical outcome parameter.

**Methods:** A retrospective analysis of 1,000 consecutive cases of primary total joint arthroplasty (hip, knee) before and 1,000 consecutive cases after the installation of an ultraclean airflow ventilation system (temperature controlled Airflow TcAF System Opragon AB, Avidicare Sweden), in the same operating room was performed. Clinical outcome was evaluated using length of stay and infection rates as endpoints. The proper function of the TcAF system was checked by intraoperative measurement using active air sampling (blood agar plates, Klotz Impactor FH6).

**Results:** The intraoperative airborne contamination was always below 5 cfu/m^3^ of air, which is the threshold demanded according to the Swedish SIS standard for ultraclean air in operating theatres demonstrating proper function of the system. Ultraclean air provided by the TcAF system was associated with a decrease in mean postoperative hospital stay from 11.0 to 8.64 days, a decrease in percentage of patients who stayed inpatient over 14 days after surgery from 7.3 to 2.2 %, and a decrease of infectious complications from 3.3 to 1.1 %.

**Conclusion:** The study shows positive impact on key clinical outcome parameters using ultraclean airflow ventilation systems in the OR. This is in line with previous research by Charnley and Lidwell. There are however limitations to the study that warrant further research to prove clinical impact and efficacy by prospective clinical studies.

**Disclosure of Interest**: S. Vasiuk: None declared, Y. Vasylchyshyn: None declared, V. Vasyuk: None declared, C. Bulitta Consultant for: Avidicare AB

### P275 CLINICAL VALIDATION OF A TEMPERATURE-CONTROLLED VENTILATION SYSTEM (TCAF) IN THE OR

#### S. Buhl^1^, L. Nilsson^2^, R. Guttenberger^1^, C. Bulitta^3^

##### ^1^Technical University of Applied Sciences Amberg-Weiden, Weiden, Germany; ^2^Avidicare AB, Lund, Sweden; ^3^Institute of Medical Engineering, Technical University of Applied Sciences Amberg-Weiden, Weiden, Germany

###### **Correspondence:** C. Bulitta

**Introduction:** Microbiological burden of room-air in operating theatres is a known risk factor for surgical site infections. However, it is unclear how to best evaluate the different ventilation technologies regarding efficacy and efficiency under routine clinical conditions with respect to reduction of this risk factor.

**Objectives:** The aim of the study was to evaluate active air sampling methodology for performance qualification of operating room ventilation technology.

**Methods:** 10 installations of the temperature-controlled ventilation system (TcAF) Opragon (Avidcare AB, Sweden) were assessed during live surgery according to the Swedish SISTS 39: 2015 standard using active air sampling (blood agar plates, Klotz Impactor FH6). The spectrum of procedures included general surgical interventions and trauma / orthopedic procedures. Colonies were counted as colony forming units per cubic meter (cfu/m³).

**Results:** There were on average 6 persons in the room with a median (M) 6, mean (MW) 6.2 and standard deviation (SD) 1.3. Measurements showed values ​​of median (M) 0 cfu/m³ over all measuring points in the room, mean value (MW) 1.8 cfu/m³, standard deviation (SD) 4.5 cfu/m³. In detail, the following germ counts ​​were obtained: In the area of ​​the surgical field median (M) 0 cfu/m³, mean value (MW) 0.4 cfu/m³, standard deviation (SD) 0.8 cfu/m³, in the range of the instrument table median (M) 0 cfu/m³, mean (MW) 1 cfu/m³, standard deviation (SD) 1.9 cfu/m³ and in the periphery median (M) 2 cfu/m³, mean (MW) 4 cfu/m³, standard deviation (SD) 6.7 cfu/m³.

**Conclusion:** Active air sampling according to the Swedish SISTS 39: 2015 standard is suitable to assess the efficacy of ventilations systems for operating rooms in a routine clinical setting. Our results show that the requirements of the Swedish standard were met or significantly excelled by the TcAF system. The temperature-controlled airflow reliably and robustly ensures "ultra-clean" air (<10 cfu/m³) in the entire operating theatre and therefore is capable to reduce the risk of airborne microbial transmission under routine clinical conditions.

**Disclosure of Interest**: S. Buhl: None declared, L. Nilsson Employee of: Avidicare AB, R. Guttenberger: None declared, C. Bulitta Consultant for: Avidicare AB

## Poster session: Ventilator-associated pneumonia / Hospital-Acquired Pneumonia / Other devices

### P276 Withdrawn

### P277 INTERVENTION TO REDUCE VENTILATOR ASSOCIATED PNEUMONIA IN ADULT INTENSIVE CARE UNIT AT A TERTIARY TEACHING HOSPITAL IN RURAL EASTERN CAPE PROVINCE, SOUTH AFRICA

#### T. Apalata, L. Venn, S. Vasaikar

##### Medical Microbiology, Walter Sisulu University and National Health Laboratory Services, Mthatha, South Africa

###### **Correspondence:** T. Apalata

**Introduction:** Ventilator-associated pneumonia (VAP) occurs among admitted patients in intensive care units (ICUs) following endotracheal intubation.

**Objectives:** The study sought to determine the aetiological agents, risk factors and rates of VAP during pre- and post-implementation of a multifaceted infection prevention and control (IPC) intervention (“VAP bundle”) in adult ICU.

**Methods:** A before-and-after study design was conducted over 9 months from 1^st^ May 2018 to 31 January 2019. All patients admitted in adult ICU were evaluated daily for the diagnosis of VAP using clinical, radiological and microbiological criteria. Data were collected using an identical tool in the pre-intervention and post-intervention periods. The “VAP bundle” consisted of a multifaceted intervention as defined by the South African “Best Care Always” quality improvement campaign. All microbiological samples were processed for microscopy, cultures on routine solid media and VITEK 2 automated system (Biomérieux, USA).

**Results:** 66 and 64 patients were part of the pre-intervention and post-intervention surveys, respectively. The baseline VAP rate showed an incidence of 36.0 per 1000 ventilator-days as compared to 22.6 per 1000 ventilator-days post-intervention. Risk factors associated with VAP in pre-intervention period consisted of patients aged ≥35 years (p = 0.003), who had an endo-tracheal tube in place (p = 0.001), and whose length of stay in ICU was ≥6 days (p = 0.028). A reduction of VAP rate was observed, from 34.8% (23/66 patients) in the pre-intervention period to 20.3% (13/64 patients) in the post-intervention period with a relative risk (RR) of 1.7 (with 95% CI:1 – 3.1; p = 0.07). The commonest aetiological agents causing VAP in pre- and post-intervention periods were mainly Gram negative bacteria mainly *Acinetobacter baumannii*, followed by *Escherichia coli*, *Klebsiella pneumoniae,* and *Pseudomonas aeruginosa*. Among the Gram-positive bacteria, *Coagulase negative Staphylococci* was the main organism isolated, although its true implication as a pathogenic agent for VAP remains controversial.

**Conclusion:** Results indicated that the incidence of VAP is high in the adult ICU, Gram negative bacteria were the most prevalent causing pathogens, and that “VAP bundle” intervention is effective in reducing VAP rate, hence improving the quality of care.

**Disclosure of Interest**: T. Apalata Other conflict with: There is no conflict of interest, L. Venn: None declared, S. Vasaikar: None declared

### P278 EVALUATION EFFECT OF VAP CARE BUNDLE ON VENTILATOR-ASSOCIATED PNEUMONIA AT INTENSIVE CARE UNITS, CHO RAY HOSPITAL

#### T. M. Phung^1^, T. T. H. Vo^1^, T. P. Nguyen^1^, V. T. Le^1^, L. A. Nguyen^1^, L. T. K. Nguyen^1^, C. T. Le^2^, O. T. Nguyen^3^, H. Kurosu^4^, V. Q. Tran^5^, X. T. Phan^6^, T. T. N. Pham^6^, T. T. A. Le^1^

##### ^1^Infection Control; ^2^Internet Technology; ^3^Nursing , CHO RAY HOSPITAL, Ho Chi Minh city, Viet Nam; ^4^Expert of Infection Control , Japan International Cooperation Agency, Tokyo, Japan; ^5^Neurosurgery ICU; ^6^ICU, CHO RAY HOSPITAL, Ho Chi Minh city, Viet Nam

###### **Correspondence:** T. M. Phung

**Introduction:** Current situation of hospital associated infection (HAIs) in Vietnam is still high alarm for infection. A lot of researches in worldwide as well as Vietnam showed HAIs increased hospitalized time, antibiotics consumption, medical fees, and high mortalities. Current Vietnam has not been systemic researches of role HAIs surveillance and giving suitable interventions. Therefore, we applied systemic approaches for HAIs surveillances, including longitual and continuous HAIs surveillances. We found that ventilator-associated pneumonia (VAP) rate was significant higher than those blood stream infection (BSI), urinary tract infection (UTI), and surgical site infection (SSI). Hence, we would like to assess the efficient VAP care bundle for VAP patients in ICUs.

**Objectives:** Assessment the effect of VAP care bundle on VAPs patients in ICU at Cho Ray hospital

**Methods:** Patients with intubation in ICUs from April 2018 to December 2018

Using intervention study by apply VAP care bundle

**Results:** Result of HAIs surveillance showed VAP rate of hospital was significantly decreased from 28.4 to 24/1000 mechanic ventilator (MV)-day (*P* = 0.0004); VAP rate of ICU from 32.8 to 24.4/1000 MV-day (*P* = 0.0055); and VAP rate of NICU from 29.5 to 16.3/1000 MV-day (*P* = 0.0001) after conducting VAP intervention. In addition, the result of study also revealed that antibiotics consumption rate of inpatients in 2018 was decreased by 15.9% compared to 2017. Futhermore, the average antibiotics consumption expense for each patient was also reduced by 4.1%.

**Conclusion:** VAP care bundle intervention induced significantly decreasing VAP rate and antibiotics consumption expense in ICUs.

**Disclosure of Interest**: None declared

### P279 MAJOR RISK FACTORS OF VENTILATED-ASSOCIATED PNEUMONIA IN MEDICAL/SURGICAL INTENSIVE CARE UNIT IN ACUTE CARE HOSPITAL

#### W. A. Mazi^1^, M. H. Abdulwahab^1^, M. A. Al Ashqar^1^, Y. S. Aldecoa^1^, Z. R. Bhat^1^, S. A. Al Sayali^2^, O. S. Yasin^2^

##### ^1^Infection Prevention and Control; ^2^Intensive Care Unit, King Faisal Medical Complex, Taif, Saudi Arabia

###### **Correspondence:** W. A. Mazi

**Introduction:** Healthcare associated infections (HAIs) increase mortality, length of hospital stay, cost of care, bacterial resistance antibiotic use and other adverse events. Ventilated associated pneumonia (VAP) is the most common HAI reported in intensive care units (ICUs) worldwide.

**Objectives:** To reduce VAP benchmarking to National Safety Network (NHSN, USA).

**Methods:** A prospective study was conducted in 27-bed medical/surgical intensive care unit of King Faisal Hospital, Taif-Kingdom of Saudi Arabia from January 2015 to August 2018. Two prevention models were introduced to reduce VAP. The first one is Institute for Healthcare Improvement (IHI) implemented in 2016. The second one is the basic recommendations of Society Healthcare Epidemiology of America/Infectious Diseases Society of America (SHEA/IDSA) used in 2018. VAP was identified using the NHSN criteria. Incidence rate, ratio, benchmarking, and statistical analysis were carried out using the NHSN recommendations. VAP Bundle program was implemented during the study period. *P*-value <0.05 (2 tailed) was considered statistically significant. Bacterial identification and antimicrobial susceptibility of isolates were determined according to Clinical Laboratory Standards Institute (CLSI) guidelines.

**Results:** VAP incidence rate was declined from 4.81- to 3.62 /1000 ventilated-days with utilization ratio 0.31 to 0.27 and from 4.87- to 3.67/1000 ventilated days with utilization ratio from 0.31 to 0.26 using IHI and SHEA/IDSA prevention models, respectively. There was no significant difference between two prevention models (*p*-value is 0.915). VAP bundle compliance rate was above 90% through-out interventions. Two major risk factors were contributed VAP; pair spontaneous breathing trials with spontaneous awakening trials and provide endotracheal tubes with subglottic secretion drainage ports.

Multi-drug resistance *Acinetobacter baulmannii* was the most common causative HAI-VAP.

**Conclusion:** Pair spontaneous breathing trials with spontaneous awakening trials and provides endotracheal tubes with subglottic secretion drainage ports are very important factors to reduce VAP.

**References**

Not applicable.

**Disclosure of Interest**: None declared

### P280 RISK FACTORS FOR NON-VENTILATOR-ASSOCIATED HOSPITAL-ACQUIRED PNEUMONIA (NVHAP)

#### A. Wolfensberger^1^, V. N. Kachalov^1^, W. Jakob^2^, S. P. Kuster^1^, P. W. Schreiber^1^, R. Kouyos^1^, H. Sax^1^

##### ^1^Department of Infectious Diseases and Hospital Epidemiology; ^2^Department of Medical Data Management Systems, Medical Directorate, UNIVERSITY HOSPITAL ZÜRICH, Zürich, Switzerland

###### **Correspondence:** A. Wolfensberger

**Introduction:** Pneumonia and lower respiratory tract infections are the most common hospital-acquired infections and the burden of disease among inpatients is higher in non-ventilated than in ventilated patients. While multiple aspects - including risk factors - of ventilator-associated pneumonia are well studied, literature on non-ventilator-associated hospital-acquired pneumonia (nvHAP) is scarce.

**Objectives:** Understanding of modifiable and non-modifiable risk factors for nvHAP is key for the development of effective prevention measures and to tailor prevention strategies to patients at highest risk. Therefore, we aimed to identify risk factors for nvHAP.

**Methods:** In this retrospective cohort study we included patients ≥ 18 years of age who were discharged during a 2 year period from the University Hospital Zurich, Switzerland. A total of 37 potential risk factors - both constant and time-varying - were extracted from electronic medical records, including demographic data, comorbidities, signs and symptoms (including daily nursing assessments), procedures, and devices. Hazard ratios for nvHAP were derived from univariable and multivariable Cox proportional hazard models.

**Results:** We included 69,717 cases of whom 481 (0.07%) had nvHAP. Median age was 57 years (IQR: 39-72), and 34,657 (49.7%) of patients were male. Independent risk factors were male gender (Hazard ratio (HR) = 1.63, 95% Confidence interval (CI)= 1.33 - 1.99), age >65 years (HR = 1.43, CI = 1.16 - 1.76), impaired consciousness (HR = 1.78, CI = 1.23 - 2.57)), impaired activity and mobility (HR = 3.37, CI = 2.37 - 4.78), acute moderate to severe pain (HR = 1.83, CI = (1.43 - 2.35), breathing problems (HR = 1.95, CI = 1.45 - 2.61), swallowing difficulties (HR = 1.56, CI = 1.13 - 2.15), vomiting (HR = 1.68, CI = 1.14 - 2.46), history of intubation or tracheostomizing (HR = 1.44, CI = 1.05 - 1.97), and monitored anaesthesia care (HR = 2.66, CI = 1.06 - 6.68).

**Conclusion:** We found impaired activity and mobility to be the strongest independent risk factor for nvHAP. This condition is often present in multimorbid, postoperative and medically sedated patients. It is a potentially targetable condition that can be approached by purposeful mobilization by physiotherapy, nursing, or patient participation.

**Disclosure of Interest**: None declared

### P281 PNEUMONIA ACQUIRED IN INTENSIVE CARE UNITS IN PATIENTS WITHOUT MECHANICAL VENTILATION: FIFTEEN YEARS PROSPECTIVE MULTICENTRIC STUDY IN A FRENCH UNIVERSITY HOSPITAL

#### C. De Bastiani^1^, N. khanafer^1^, T. Rimmele^2^, C. Guérin^2^, L. Argaud^2^, A. Friggeri^2^, S. Arnal^2^, C. Monard^2^, A. Lepape^2^, E. Marion^1^, P. Vanhems^1^

##### ^1^Hygiène hospitaliere; ^2^Réanimation, Hospices Civils de Lyon, Lyon, France

###### **Correspondence:** C. De Bastiani

**Introduction:** Nosocomial pneumonia (NP) is the most common infection in Intensive Care Units (ICU). Invasive mechanical ventilation is known as the major/principal risk factor but other risk factors cannot be excluded.

**Objectives:** The objectives of this study were to describe NP in non-intubated patients and to identify associated risk factors.

**Methods:** Data from the REA-RAISIN surveillance network collected between 2003 and 2018 in 11 ICU’s in a French university hospital were analyzed. Only patients hospitalized at least 48 hours and who had not invasive mechanical ventilation before a possible NP were included. Descriptive analysis and univariate logistic regression were done with SPSS 21^®^.

**Results:** A total of 49,348 patients were included in the network. Of whom, 13,457 (35%) patients didn’t have invasive mechanical ventilation and 123 (0.9%) patients developed NP. The median age was 65 (51-75) years and there were more men than women (62% *vs* 38%; P=0.015). The median length of stay was significantly different between patients with NP and patients non-infected (10 and 4 days respectively; p<0.001). The most frequently isolated micro-organisms were *S. aureus* (n=22, 19.3%) with 37% resistant to methicillin, *P. aeruginosa* (n=20, 17.5%) with 55% resistant to Carbapenem and/or Cephalosporin’s third generation.

Male gender (OR: 1.64,; P=0.02), trauma (OR: 2.45; P<0.001), scheduled surgery (OR: 1.97; P=0.004), exposure to central venous catheter (1.99; P<0.001) or indwelling catheter (OR: 2.15; P=0.002), previous ICU hospitalization (OR: 3.52; P<0.001) were the independent risk factors for NP in non-intubated patients.

**Conclusion:** Identifying risk factors of NP in non-intubated patients in ICU may help to optimize appropriate preventive measures. The major risk factor of NP remains Mechanical ventilation.

**Disclosure of Interest**: None declared

### P282 RISK FACTORS FOR ACQUISITION HEALTHCARE ASSSOCIATED INFECTIONS AND /OR COLONIZATIONS IN INTENSIVE CARE UNITS. A META-ANALYSIS OF CASE CONTROL AND COHORT STUDIES.

#### L. M. Parra Ramírez^1^, M. Cantero Caballero^1^, J. Sierra Marticorena^1^, Á. Asensio Vegas^1^, F. J. García López^2^

##### ^1^Preventive Medicine Department, Puerta de Hierro Majadahonda University Hospital; ^2^National Epidemiology Centre, Carlos III Institute of Health, Madrid, Spain

###### **Correspondence:** L. M. Parra Ramírez

**Introduction:** Water systems act as an important reservoir of microorganism in ICUs.

**Objectives:** To conduct a systematic review to identify risk factors associated with acquisition of waterborne healthcare-associated infection (HAI) and/or colonization.

**Methods:** A systematic review and meta-analysis was conducted. PubMed, Web science and Embase were searched to identify cohort and case-control studies that had reported on the risk factors associated with acquisition of waterborne-HAI and/or colonization from 1966 to 10 July 2018. Three investigators independently screened titles and abstracts for relevant articles, and two screened full-text articles. Summary estimates were obtained using random effect model.

**Results:** Ten relevant studies met the inclusion criteria for the systematic review (5 cohort studies and 5 case control studies). We identified no point-of-use filter installed on the sink faucet (OR: 5.79, 95%CI=1.29-30.62) and exposure to invasive ventilation (OR, 5.79; 95% CI: 1.29-30.62) as risk factors in neonatal population. Be admitted to a room with a contaminated tap (HR=1.76, 95% CI=1.09-2.84) or contaminated sink (OR:11.2, 95% CI= 1.92-65.68), exposure to invasive ventilation >10 days (HR: 2.56, 95% CI= 1.46-4.50) and require hemofiltration (OR: 22.9, 95% CI= 1.63-203.91) were identified as risk factors in adult population. The case-control studies contributed to a meta-analysis. Waterborne-HAI and/or colonization was positively associated in neonatal population with CVC exposure (pooled OR: 8.02, 95% CI= 2.19-29.31, p=0.002). Umbilical catheter exposure (pooled OR: 2.41, 95% CI= 0.20-28.33, p=0.49) and total parenteral nutrition exposure (pooled OR: 11.52, 95% CI= 0.55-240.94, p=0.12) were not associated with acquisition. Length of stay before colonization was positive associated with an increased risk of waterborne-HAI and/or colonization acquisition in adult population. Duration of antibiotic treatment in ICU was identified as a protective factor in adult population.

**Conclusion:** In this meta-analysis, CVC exposure in neonates and length of stay in adult population were associated with an increased risk of waterborne-HAI/colonization.

**Disclosure of Interest**: None declared

### P283 REDUCTION OF DEVICE–ASSOCIATED INFECTIONS IN INTENSIVE CARE UNITS USING PREVENTION MODELS IN TAIF REGION, KINGDOM OF SAUDI ARABIA

#### W. A. Mazi^1^, M. A. Shah^2^, M. H. Abdulwahab^1^, S. S. Ghiaty^3^, A. M. Al-Qarni^4^, S. Y. Al Zahrani^2^, Y. S. Aldecoa^1^, N. J. Helali^3^, A. M. Tan^3^, S. F. Al Hamadi^4^, S. S. Al Mones^5^

##### ^1^Infection Prevention and Control, King Faisal Medical Complex; ^2^Regional Directorate for Infection Prevention and Control, Directorate of Health Affairs; ^3^Infection Prevention and Control, King Abdul Aziz Specialist Hospital; ^4^Infection Prevention and Control, Children Hospital; ^5^Director Administration, Directorate of Health Affairs, Taif, Saudi Arabia

###### **Correspondence:** W. A. Mazi

**Introduction:** Healthcare associated infections (HAIs) increase mortality, length of hospital stay, cost of care, bacterial resistance, antibiotic use and other adverse events in intensive care units (ICUs).

**Objectives:** To reduce HAIs in ICUs.

**Methods:** The study was conducted in three medical/surgical intensive care units of three hospitals from January 2017 to September 2018. Unified surveillance was considered using Centre for Diseases Control and Prevention (CDC) and National Healthcare Safety Network (NHSN, USA) criteria. Incidence rate, utilization ratio, benchmarking, and statistical analysis were carried out using the NHSN recommendations. Different prevention models were implemented.

**Results:** Ventilated associated pneumonia (VAP) was the most common of HAIs followed by central line-associated bloodstream infection (CLABSI) in King Abdul Aziz Specialist (KAAS) and Children Hospitals and catheter associated urinary tract infection (CAUTI) in KAAS hospital.

King Faisal Medical Complex (KFMC) implemented Society Healthcare Epidemiology of America/Infectious Diseases Society of America (SHEA/IDSA) prevention model. VAP incidence rate was declined from 5.19 to 3.55/1000 ventilated-days (*p*-value 0.36) with lower in utilization ratio (0.31 *vs* 0.29).

Children Hospital and KAASH implemented Institute for Healthcare Improvement (IHI) prevention model. CLABSI and VAP incidence rates in Children hospital were declined from 11.18- to 4.02/1000 central line-days (*p*-value is 0.003) and from 4.02- to 2.12/1000 ventilated-days (*p*-value is 0.14) despite of higher in utilization ratio (0.14 *vs* 0.21 and 0.65 *vs* 0.82, respectively).

In KAASH, VAP incidence rate was declined from 6.27 to 4.85 per 1000 ventilated-days (*p*-value 0.51) despite of higher in utilization ratio (0.42 *vs* 0.50). No significant reduction of CLABSI incidence rate with same in utilization ratio.

**Conclusion:** There is reduction of CLABSI and VAP incidence rates despite of high in utilization ratio. However, VAP incidence rate is still above 90th percentile benchmarking to NHSN.

**References**

Non Applicable.

**Disclosure of Interest**: None declared

## Poster session: Catheter-associated urinary tract infections

### P284 THE IMPACTS OF CATHETER-ASSOCIATED URINARY TRACT INFECTION ON PROGNOSES OF 11,163 INTENSIVE CARE UNIT PATIENTS WITH INDWELLING URINARY CATHETER: AN ANALYSIS USING PROPENSITY SCORE MATCHING MODEL

#### S. Zhu^1^, Z. Zong^1^, L. Cai^2^, W. Yin^1^

##### ^1^Infection Control Department; ^2^Intensive Care Unit, West China Hospital of Sichuan University, Chengdu, China

###### **Correspondence:** S. Zhu

**Introduction:** The clinical impacts of catheter-associated urinary tract infection (CAUTI) were uncertain because of lacking of high quality evidences in China.

**Objectives:** This study was performed to identify the clinical impacts of CAUTI on patients with indwelling urinary catheter in the intensive care unit (ICU) settings in China.

**Methods:** Using the prospectively collected ICU nosocomial infection target monitoring database, a 3-year prospectively cohort study design from April 2015 to March 2018 was applied in all the 5 adult ICUs in a 5000-bed tertiary-care hospital in China. Adult patients with indwelling urinary catheter > 2 days in the ICUs were included. The exposure factor was CAUTI in ICU. The outcomes were duration of indwelling urinary catheter, length of stay (LOS) in ICU and in hospital, hospitalization costs, and all-cause deaths in ICU and in hospital. Propensity score matching (PSM) model (with the caliper value of 0.02 and matching ratio of 1:4) was used to assess the impacts of CAUTI. The potentially confounding factors included demographic characteristics, disease score, combined diseases and treatment operations.

**Results:** A total of 11,163 patients with indwelling urinary catheter > 2 days (with 107,566 catheter-days) were included, 185 patients developed 192 CAUTI episodes (1.7 cases per 100 patients, 1.8 cases per 1,000 catheter-days). After PSM a new cohort with 185 cases in the exposed group and 671 cases in the non-exposed group was developed. Patients with CAUTI had 23-day longer duration of indwelling urinary catheter, 23-day increased LOS in ICU, 22-day increased LOS in hospital and 11.1-thousand yuan excessed hospitalization costs than those without (P<0.05). CAUTI was an independent risk factor for each of the 4 outcomes above (P<0.05), respectively. However, CAUTI did not increase the risk for all-cause death in ICU and in hospital.

**Conclusion:** Although CAUTI could not increase the risk for hospitalization death, it is significantly associated with prolonged duration of indwelling urinary catheter, LOS in ICU and hospital and increased hospitalization costs, which lead to the waste of the limited medical resources in China. Therefore, it is necessary to take appropriate intervention measures to prevent and control CAUTI.

**Disclosure of Interest**: None declared

### P285 COMPARISON OE ESCHERICHIA COLI AND KLEBSIELLA PNEUMONIAE IN HEALTHCARE-ASSOCIATED ACUTE PYELONEPHRITIS

#### M. Hyun, S. Y. Ryu

##### Keimyung University Dongsan Medical Center, Daegu, Korea, Republic Of

###### **Correspondence:** M. Hyun

**Introduction:** *Escherichia coli (E. coli)* and *Klebsiella pneumoniae (K. pneumoniae)* is one of the most common cause of healthcare-associated urinary tract infection (UTI).

**Objectives:** The purpose of this study is to compare clinical characteristics and antimicrobial susceptibility of healthcare-associated acute pyelonephritis (APN) between *E. coli* and *K. pneumoniae*.

**Methods:** We reviewed medical records of healthcare-associated APN with *E. coli* and *K. pneumoniae* between February 2014 and October 2017. Severe APN was defined as severe sepsis or septic shock due to APN.

**Results:** A total 114 patients were enrolled with 64 of *E. coli* and 50 of *K. pneumoniae*. Mean age of *E. coli* was older than *K. pneumoniae* (74.22 ± 12.24 vs. 65.76 ± 14.73, *p*=0.001). Neurogenic bladder, obstructive uropathy and urinary tract stone were more associated with *E. coli*. Catheter associated APN and complicated APN were more observed in *K. pneumoniae*. Bacteremia and severe APN were more accompanied by *E. coli*. Extended-spectrum β-lactamase (ESBL) production was more observed in *E. coli*. Among ESBL, *E. coli* was more susceptible to piperacillin/tazobactam (75.0% vs. 46.9%, p=0.01) and *K. pneumoniae* was more susceptible to ciprofloxacin (10.4% vs. 37.5%, p=0.005). Thirty-day mortality was higher in *K. pneumoniae*.

**Conclusion:** *E. coli* was tended to be more associated with anatomical or functional urogenital problem, and *K. pneumoniae* was more associated with urinary catheter. The distribution of causative strains of catheter associated UTI was different from other studies. Even high rate of ESBL production, piperacillin/tazobactam can be used empirical treatment of healthcare-associated UTI. Still, ciprofloxacin and trimethoprim/sulfamethoxazole can be good option for *K. pneumoniae* UTI.

**Disclosure of Interest**: None declared

### P286 RISK FACTORS AND 30-DAY MORTALITY OF SEPTIC SHOCK DUE TO COMPLICATED URINARY TRACT INFECTION. A MULTICENTER STUDY

#### A. Gomila^1^, J. Carratalà^1^, C. Vuong^2^, I. Addy^2^, I. Wiegand^2^, N. Eliakim-Raz^3^, E. Shaw^1^, L. Vallejo-Torres^4^, J. M. Vigo^5^, S. Morris^4^, S. Grier^6^, C. Vank^2^, N. Cuperus^7^, L. van den Heuvel^7^, A. MacGowan^6^, L. Leibovici^3^, M. Pujol^1^ on behalf of RESCUING Study Group and Study Sites

##### ^1^Infectious Diseases Department, Bellvitge University Hospital, Barcelona, Spain; ^2^AiCuris Anti-infective Cures, Wuppertal, Germany; ^3^Beilinson Hospital, Tel Aviv, Israel; ^4^University College London, London, United Kingdom; ^5^Fundació Institut Català de Farmacologia, Barcelona, Spain; ^6^Southmead Hospital, Bristol, United Kingdom, ^7^University Medical Center Utrecht, Utrecht, Netherlands

###### **Correspondence:** A. Gomila

**Introduction:** Complicated urinary tract infections (cUTI) are one of the most frequent infections, and are associated with significant health costs and antimicrobial resistance.

**Objectives:** To analyse risk factors and 30-day mortality of patients with cUTI presenting with severe sepsis or septic shock.

**Methods:** Multicentre, observational study at 20 hospitals in 7 South European countries and Israel. Data was retrospectively collected from patients with cUTI hospitalised between 2013 and 2014.

**Results:** Of 1007 patients, 785 were evaluated for complete information on sepsis status at diagnosis. Of them, 141 (17.9%) had severe sepsis or septic shock (SS-SS), 32.6% being urinary catheter-associated. Patients’ characteristics independently associated with SS-SS compared to sepsis were having haematological malignancy (Odds ratio [OR] 3.31, 95% confidence interval [95%CI] 1.08-10.15), higher Charlson score (OR 1.16, 95%CI 1.01-1.25), obstructive uropathy (OR 1.88, 95%CI 1.17-3.01) and bacteraemia (OR 2.9, 95%CI 1.9-4.41). No particular microbiology, including presence of multidrug-resistant Gram-negative bacteria (MDR-GNB), was associated with SS-SS. Overall 30-day mortality was low (8%), significantly higher in SS-SS group than in sepsis group (26.2% vs 5%, p<0.001). Among those who died (n=69), there was less percentage of infection source control measures than among those who did not (5.4% vs 10.6%, p=0.35), although not statistically significant. Independent risk factors for 30-day mortality in patients with SS-SS were age, having a haematological malignancy, admission for a different reason than cUTI and a dependent functional capacity.

**Conclusion:** Almost 18% of patients with cUTI presented with SS-SS. Significantly, no particular microbiology, including MDR-GNB, was associated with SS-SS. Mortality of patients with SS-SS was high, more related to the basal condition of patients than to an adequate antibiotic management and source control.

**Disclosure of Interest**: A. Gomila: None declared, J. Carratalà: None declared, C. Vuong Grant/Research support from: belong to EFPIA (European Federation of Pharmaceutical Industries and Association) member companies in the IMI JU and costs related to their part in the research were carried by the respective company as in kind contribution under the IMI JU scheme, I. Addy Grant/Research support from: belong to EFPIA (European Federation of Pharmaceutical Industries and Association) member companies in the IMI JU and costs related to their part in the research were carried by the respective company as in kind contribution under the IMI JU scheme, I. Wiegand Grant/Research support from: belong to EFPIA (European Federation of Pharmaceutical Industries and Association) member companies in the IMI JU and costs related to their part in the research were carried by the respective company as in kind contribution under the IMI JU scheme, N. Eliakim-Raz: None declared, E. Shaw: None declared, L. Vallejo-Torres: None declared, J. M. Vigo: None declared, S. Morris: None declared, S. Grier: None declared, C. Vank Grant/Research support from: belong to EFPIA (European Federation of Pharmaceutical Industries and Association) member companies in the IMI JU and costs related to their part in the research were carried by the respective company as in kind contribution under the IMI JU scheme, N. Cuperus: None declared, L. van den Heuvel: None declared, A. MacGowan: None declared, L. Leibovici: None declared, M. Pujol: None declared

### P287 RISK FACTORS RELATED TO CATHETER-ASSOCIATED URINARY TRACT INFECTIONS AT A TERTIARY CARE CENTER IN LEBANON

#### A. IBRAHIM^1^ on behalf of IC TEAM , N. K. Zahreddine^1^, J. TANNOUS^1^, R. AHMADIEH^1^, T. KARDAS^1^, Z. KANAFANI^2^, S. KANJ^2^

##### ^1^Infection Control and Prevention Program, ^2^INFECTIOUS DISEASES, AMERICAN UNIVERSITY OF BEIRUT MEDICAL CENTER, Beirut, Lebanon

###### **Correspondence:** J. TANNOUS

**Introduction:** Catheter-Associated Urinary Tract Infections (CAUTI) are a leading cause of Healthcare Associated Infections with increased morbidity, mortality and cost. CAUTIs can be prevented through the application of the CAUTI prevention bundle. However, the likelihood of CAUTI increases with patient related risk factors such as age, gender, critical care stay, duration of the urinary catheter (UC), bed-bound patients resulting in urine stasis, antimicrobial treatment or documentation of multiple insertion trials.

**Objectives:** This study was conducted to determine the CAUTI risk factors and the resulting causative organisms.

**Methods:** A prospective hospital-wide surveillance was conducted between January 2018 and March 2019 at the American University of Beirut Medical Center. CAUTI was diagnosed using the CDC/NHSN (Centers for Disease Control and Prevention/National health and Safety Network) definition. All patients who developed CAUTI were recruited. A checklist was adopted which included most common risk factors for CAUTI.

**Results:** 66 patients developed CAUTI during the study period of which 41 (62%) were females. 30 (45%) patients were above 65 years of age. The average duration of UC placement prior to CAUTI was 14 days with 53 (80%) of the cases occurring after day 5 of UC insertion. The majority of patients were bed ridden 58 (88%). 57 (86%) patients underwent one or more transfers for diagnostic procedures such as CT and MRI. CAUTI cases were most commonly identified in the neurosurgical ICU (15 [23%]). Antimicrobial treatment of **>** 5 days prior to CAUTI was identified in 46 (70 %) of the cases. Admission to a critical care unit before CAUTI was noted in 50 (76 %) cases. CAUTI organisms were predominantly Gram-negative bacilli (91%), with *Escherichia coli* (38%) being the most common causative organism (40% ESBL, 28% MDR); followed by *Klebsiella pneumoniae* (29%) with 21% ESBL and 16% MDR.

**Conclusion:** Understanding CAUTI risk factors plays a major role in reducing CAUTI rates and can improve patient safety outcomes. Maintaining the UC bundle at all times can be instrumental in preventing CAUTI with strict implementation of all its elements and in particular the daily assessment of the need to keep the UC.

**Disclosure of Interest**: None declared

### P288 REDUCING CATHETER ASSOCIATED URINARY TRACT INFECTION RATE: EXPERIENCE FROM RIYADH, SAUDI ARABIA

#### H. A. Amer, H. A. Abdalla, F. A. Alaklobi

##### Infection Control Dept, King Saud Medical City, Riyadh, Saudi Arabia

###### **Correspondence:** H. A. Amer

**Introduction:** Urinary tract infection has long been considered the most common healthcare-associated infection (HAI). In USA, Catheter Associated Urinary Tract Infections (CAUTIs) are associated with an additional cost of $676 per admission and being preventable it was the first hospital-acquired conditions selected for non-payment by Medicare as of October 2008, and have been further targeted for complete elimination as a “never event,”.

High CAUTI rate was reported in first quarter of 2017 (6.2 / 1000 urinary catheter days) at Intensive Care Unit (120 bed ICU) at King Saud Medical City (KSMC).

**Objectives:** ≥20% reduction of CAUTI rate annually till being ≤ National Healthcare Safety Network (NHSN) pooled mean (2.2 /1000 catheter days).

**Methods:** Multidisciplinary team formulated. Implementation of Evidence based practices listed in urinary catheter bundle of care was initiated and checklist was approved as part of medical record. Strategies conducted include: focus group discussion with nurses and physicians’ team members, education about Foley’s catheter care maintenance bundle, review process of urine sample collection to avoid contamination, empower nurses to review catheter necessity daily and removal of unnecessary catheter, and continuous auditing of bundle compliance by infection control staff. Ensure availability of Folly’s catheter insertion kit with single used lubricant gel and availability of catheter type that has sample collection port was important to maintain closed system. Recommendation for improvement circulated categorized as high and low impact. Updated CAUI surveillance criteria based on Centre of Disease Control (CDC) was reviewed.

**Results:** Around 50 % reduction of CAUTI rate was achieved by end of 2017

**Conclusion:** Implementation of urinary catheter bundle of care proved to be effective in reducing CAUTI. Continue education and bundle monitoring are essential for further rate reduction and sustaining the improvement. Involvement of front line staff in initiatives planning and implementation are critical to achieve the target. A project of applying simulation training for Folly’s catheter insertion and care initiated in collaboration with nursing, it is impact on CAUTI prevention is expected to be evaluated soon.

**Disclosure of Interest**: None declared

## Poster session: Influenza 2

### P289 NOT SICK ENOUGH TO WORRY? THE FLU AND WORK-RELATED BEHAVIOUR AMONG HEALTHCARE WORKERS AND OTHER PROFESSIONALS WORLDWIDE

#### E. Tartari^1^, K. Saris^2^, N. Kenters^2^, A. Widmer^3^, P. Collignon^4^, V. Cheng^5^, S. C. Wong^5^, T. Gottlieb^6^, K. Marimuthu^7^, E. Perencevich^8^, P. Tambyah^7^, A. Dramowski^9^, M. Edmond^8^, A. Voss^2^

##### ^1^University Hospital of Geneva, Geneva, Switzerland; ^2^Department of Medical Microbiology and Infectious Diseases, Nijmegen, Netherlands; ^3^University hospital , Bassel, Switzerland; ^4^Australian National University, Canberra, Australia; ^5^The University of Hong Kong, Hong Kong, Hong Kong; ^6^University of Sydney, Sydney, Australia; ^7^National University of Singapore, Singapore, Singapore, ^8^University of Iowa Hospitals, Iowa, United States; ^9^University of Cape Town, Cape Town, South Africa

###### **Correspondence:** E. Tartari

**Introduction:** Healthcare workers (HCWs) working while sick, with influenza-like symptoms and/or returning too early to work may play a role in the transmission of the influenza virus to susceptible patients. While this problem has been recognized, there are few data on its extent internationally.

**Objectives:** To explore the beliefs and attitudes of HCWs on influenza virus infection and vaccination practices internationally.

**Methods:** Between October 2018 and January 2019 we conducted a cross-sectional international online survey on behaviour of HCWs around influenza virus. The survey was distributed through the International Society of Antimicrobial Chemotherapy (ISAC), Infection Prevention and Control (IPC) Working Group to its members and networks and also via social platforms including: LinkedIn, Twitter and the IPC Blog.

**Results:** A total of 557 responses were collected, of which 44.7% were HCWs and 51.2% other professionals (50% North Europe, 16.2% South-east Europe, 18.3% Australia, 8.8% North America, 1.6% South America). The top symptoms reported by respondents as reasons to stay at home and report sick from work were: fever (75.4%), cold chills (49%), and a pounding headache (44.3%). However, respondents reasons to stay at home, did not match with what they considered to be the most relevant influenza symptoms: fever (81.7%), muscle aches (61.4%), and cold chills (39.7%). Only 65% would get a flu vaccine annually. 37% of respondents claimed not to have had the flu in the past two years. 56% indicated that they would still go to work when experiencing flu-like symptoms and 62.8%, would stay at work while feeling sick. 62.6% would return to work despite still having some symptoms.

**Conclusion:** To achieve successful strategies to prevent influenza transmission future awareness campaigns should be broadened to include policies on sick leave for HCWs with flu-like symptoms, while encouraging the uptake of influenza vaccine.

**Disclosure of Interest**: None declared

### P290 PREVENTION OF NOSOCOMIAL INFLUENZA (NOSO): A MULTIMODAL INTERVENTION FOCUSED ON HEALTHCARE WORKERS IS INSUFFICIENT

#### A. Iten^1^, C. Bonfillon^2^, C.-A. Siegrist ^3^, L. Kaiser^4^, D. Pittet^1^

##### ^1^Infection Control Program; ^2^Hospital Health Service; ^3^Pediatrics; ^4^Laboratory of Virology, HUG, Geneva, Switzerland

###### **Correspondence:** A. Iten

**Introduction:** Vaccination is the cornerstone for the prevention against seasonal influenza (SI). In countries where vaccination cannot be made mandatory by law, an alternative exists for hospitals: during SI epidemics, obligation for healthcare workers (HCW) to be vaccinated or to wear a mask. In case of a high incidence of patients hospitalized with SI, mask wear may be temporarily made mandatory for all HCW and visitors. This strategy, called “zoning”, has been adopted by HUG since 2009.

**Objectives:** To describe SI epidemics (inclusive NOSO) and observance to recommendations in our university hospital from 2011/12 to 2018/2019

**Methods:** Suspected cases of SI (respiratory symptoms, fever with chills, muscular pain, or prostration) were screened using nasopharyngeal samples, which were analyzed by RT-PCR. Cases were defined as NOSO when symptoms occurred >72 h after admission. Regular audits were performed to assess HCW and visitors’ compliance with recommendations. Hospital teams were regularly informed about the evolution of SI epidemics and the results of the audits.

**Results:** From winter 2011/12 to winter 2018/19, 3036 patients (min: 148 in winter 2011/12; max: 575 in winter 2016/17) were positive for influenza virus. Droplet precautions with single room isolation were implemented whenever possible, i.e. for 2729 patients (89.9%; min. in winter 2011/12: 73/148, 49.3%). The vaccination rate of hospital staff increased by 17.5% (winter 2011/12: 33.0%; winter 2018/19: 50.5%) and HCW compliance with recommendations (vaccine or mask wear during SI epidemics) increased by 23.7% (winter 2011/12: 55.4%; winter 2018/19: 89.0%). During the winter of 2018/19, only 16.8% of the visitors in care units wore masks. At the same time, the rate of NOSO declined by 31.6%, but was still 24.4% in winter 2018/19).

**Conclusion:** The combination of HCW vaccination and infection control procedures for patients and HCW contributes to preventing nosocomial transmission of the influenza virus, but are not sufficient. Additional measures are necessary and should involve patients and visitors

**Disclosure of Interest**: None declared

### P291 LARGE VARIABILITY OF THE EFFECTS OF PREVENTIVE MEASURES ON THE OCCURRENCE OF NOSOCOMIAL INFLUENZA INFECTION (NOSO-INF) ACROSS HOSPITAL SECTORS

#### A. Iten^1^, C. Bonfillon^2^, C.-A. Siegrist^3^, L. Kaiser^4^, D. Pittet^5^

##### ^1^Infection Control Program; ^2^Hospital Health Service; ^3^Pediatrics; ^4^Laboratory of Virology; ^5^Infection Control Program, HUG, Geneva, Switzerland

###### **Correspondence:** A. Iten

**Introduction:** Vaccination (V) is the cornerstone of the prevention against seasonal influenza (SI). In countries where V cannot be made mandatory, the strategy for hospitals to prevent NOSO-INF during SI epidemics combines the obligation for healthcare workers (HCW) to be vaccinated or to wear a mask, with the promotion of hand hygiene. Additionally, infection control procedures (droplet precautions with single isolation whenever possible) are implemented for suspected cases of influenza (I). This strategy, called “zoning”, is adopted by HUG since 2009.

**Objectives:** To describe SI epidemics (inclusive NOSO-INF) and compliance with recommendations at HUG during winter 2018/19.

**Methods:** Suspected cases of SI (respiratory symptoms, fever with chills, muscular pain, or prostration) were tested for I virus using nasopharyngeal samples and confirmed by RT-PCR. Cases were defined as NOSO-INF when symptoms occurred >72 h after admission. Regular audits were performed to assess HCWs’ compliance with recommendations (V; wearing of a mask if not vaccinated; adherence with hand hygiene practices).

**Results:** During winter 2018-2019, 571 I cases were hospitalized; the table includes the data of 460 patients who have already been reviewed.

**Table:** 

Preliminary data	HOSPITAL AREAS				
	Hospital	Pediatrics	Medicine	Geriatrics	Rehabilitation
Patients [n]	460	8	207	141	24
Age [y]	73.2	5.9	67.5	85.4	81.6
Sex [F %]	52.4	0.12	50.2	58.1	50.0
Antiviral treatment [%]	64.1	50.0	64.7	59.6	70.8
Complications [%]	73.3	75.0	87.9	71.6	45.8
Vaccinated [%]	21.5	25.0	27.0	20.6	16.6
Dropplets measures installed [%]	93.9	100.0	95.6	97.2	19.2
Hospital staff {n]	12157	1031	418	563	375
Vaccinated [%]	39.0	50.9	54.1	46.3	41.1
Respecting the recommandations [%]	92.2	90.2	93.7	90.3	89.5
**Nosocomial cases [%]**	**21.1**	**0.0**	**3.8**	**26.9**	**70.8**

**Conclusion:** These preliminary results show that the rate of NOSO-INF varies greatly from one hospital sector to another (range 0% to 70.8%). These initial data suggest that the lack of appropriate measures for patient installation and a lower V rate for patients and hospital staff contribute to the occurrence of NOSO-INF. Following the review of patient records, additional analyses are planned.

**Disclosure of Interest**: None declared

### P292 CLINICAL SIGNS AND TREATMENT OUTCOMES OF CHILDREN WITH H1N1 INFLUENZA IN TWO OUTBREAKS, SOUTHEASTERN IRAN

#### A. Hosssininasab

##### Pediatrics, Kerman University of Medical Sciences, Kerman, Iran, Islamic Republic Of

**Introduction: Influenza is an acute respiratory infection that has a new presentation every year due to a viral mutation. The aim of this study was to evaluate the clinical signs and outcomes of treatment of children with H1N1 influenza in the fall of 2015 and 2017 in Southeastern of IRAN.**

**Objectives: The aim of this study was to evaluate the clinical signs and outcomes of treatment of children with H1N1 influenza in the fall of 2015 and 2017 in Southeastern of IRAN.**

**Methods: This descriptive cross-sectional study was performed and samples of the nasopharynx in patients with flu-like symptoms in Afzalipour Hospital were investigated using RRT-PCR method And the data collected are analyzed based on the frequency of data and the Chi-square.**

**Results: The findings of the study indicate in the year 2017 of 37 PCR positive cases 21 were male and 16 were female. In 2015 of the 16 positive PCRs, 7 were female and 9 were male. The most frequent age was 5 to 8 years. The most common symptoms were fever, respiratory symptoms, gastrointestinal symptoms, weakness, myalgia, and neurological symptoms consequently.**

**In 2017, all PCR patients were discharged without complication. In 2015, one patient with positive PCR was dead. In 2017, among the samples in which their PCR was positive 89% of were received the antiviral drug, and this figure was 88% for 2015.**

**Conclusion: There was no significant relationship between clinical manifestation, treatment outcome, age, sex and vaccination of the patients.**

**Disclosure of Interest**: None declared

### P293 EVALUATION OF THE EFFECTIVENESS OF THE INFLUENZA VACCINE TO PREVENT SEVERE CASES OF INFLUENZA IN A TERTIARY HOSPITAL. SEASONS 2012-2013 TO 2018-2019

#### V. SOLER_MOLINA, A. LAMEIRAS_AZEVEDO, P. GRAS_ VALENTÍ, S. BALBOA_ESTEVES, J. G. MORA_MURIEL, P. CHICO_SÁNCHEZ, S. CANOVAS_JAVEZA, J. SÁNCHEZ_PÁYA

##### PREVENTIVE MEDICINE, GENERAL HOSPITAL OF ALICANTE, ALICANTE, Spain

###### **Correspondence:** V. SOLER_MOLINA

**Introduction:** Influenza is a major public health challenge worldwide assuming a special risk within health facilities to have a population of susceptible patients who require more care, the flu is more lethal in this area.The main strategy for the prevention and control of influenza during the last 60 years has been vaccination; It is important to evaluate the effectiveness of the influenza vaccine every year.

**Objectives:** Estimate the effectiveness of the influenza vaccine to prevent cases of severe influenza in patients treated at a tertiary hospital during the 2012-2013 to 2018-2019 seasons.

**Methods:** A case-control study was conducted to analyze the effectiveness of the influenza vaccine to prevent severe cases of influenza (SCI) in the seasons 2012-2013 to 2018-2019 from the data obtained from the surveillance system in the General Hospital from Alicante. All admitted patients were included as cases, with a positive result for A / B influenza confirmed by laboratory and with diagnosis of SCI. As controls, those patients diagnosed with influenza who do not meet the criteria of a severe case. As explanatory variables, those collected in the SCI survey will be included. For all patients, the SCI percentage with its 95% confidence intervals was calculated. For the association study, the Odds Ratio (OR) and its 95% confidence intervals were calculated. For the calculation of vaccine effectiveness the formula was used: (1-OR) x 100.

**Results:** 1953 cases of influenza were included. 396 were diagnosed with SCI (20.27%). The effectiveness of the vaccine to prevent SCI was 38.4% (-33.4 - 71.9) in the 2012-13 season, 45% (-4.4 - 71.5) in 2013-14 5.5% (-74.4 -48.9) in 2014-15, 9.7% (-66 -51.2) in 2015-16, 5% (-91.7-52.9) in 2016-17 , 7% (-31-36.7) in 2017-2018 and in 2018-19 52.3 (8.3-75.2).

**Conclusion:** The effectiveness of the vaccine for the prevention of SCI is low and varies by season. These differences in the effectiveness of the vaccine over the seasons studied can be related to the concordance of the strain contained in the vaccine and the circulating strain. The distribution of other known risk factors due to lack of data could not be assessed

**Disclosure of Interest**: None declared

### P294 INFLUENZA VIRUS A(H1N1)PDM09 INDUCES LETHAL OUTCOMES MORE FREQUENTLY AND IN YOUNGER PEOPLE THAN INFLUENZA VIRUS A(H3N2).

#### S. Yatsyshina, M. Elkina, V. Akimkin

##### Rospotrebnadzor, The Central Research Institute for Epidemiology of Rospotrebnadzor (CRIE), Moscow, Russian Federation

###### **Correspondence:** S. Yatsyshina

**Introduction:** One hundred twenty seven lethal cases of influenza were reported in 2017-2018 season in Russia. Etiology and epidemiology knowledge helps to prevent fatal outcomes.

**Objectives:** Investigation the etiology of Influenza and analysis of risk factors for fatal outcomes due to Influenza.

**Methods:** Ninety autopsy lung samples from lethal influenza cases were examined for the presence of Influenza viral RNA and bacterial DNA that could cause pneumonia. Total RNA and DNA were extracted using the “RIBO-prep nucleic acid extraction kit”, RNA was used to prepare cDNA by reverse-transcription with the “REVERTA-L RT reagents kit” (AmpliSens, CRIE, Russia). Influenza viral infection was confirmed by two-step real-time RT-PCR with the “AmpliSens Influenza virus A/B-FRT PCR kit”, the “AmpliSens Influenza virus A/H1-swine-FRT PCR kit” and “AmpliSens Influenza virus A-type- FRT PCR kit” (CRIE, Russia) according to the manufacturer’s instructions on a Rotor-Gene 6000 Instrument (Corbett Research, Sydney, Australia). DNA was used for detection of *Streptococcus pneumoniae, Haemophilus influenzae, Staphylococcus aureus, Streptococcus pyogenes, Acinetobacter baumannii, Pseudomonas aeruginosa, Klebsiella pneumoniae* DNA in quantitative real-time PCR with the “AmpliSens” reagents kits (CRIE, Russia).

**Results:** Ninety three percent were non-vaccinated from influenza, 89 % had comorbid conditions (arterial hypertension, coronary heart disease, diabetes, obesity or overweight, chronic respiratory diseases). According to laboratory surveillance data influenza lethal cases were caused by *Influenza A(H1N1)pdm09 virus* more frequently than *Influenza A(H3N2) virus* and Influenza B virus (72, 12 and 6 cases, respectively, p<0.0001). The mean age of adult patients with fatal outcomes of Influenza A(H1N1)pdm09 was less than of Influenza A(H3N2) (54.2±14 age vs 68.4±15 age, p<0.0001) fatal outcome. An examination of lung samples did not reveal bacterial superinfections in 72.2% of A(H1N1)pdm09 cases versus 16.7% of A(H3N2) cases (p<0.001).

**Conclusion:** The short duration (8.1±3.8 days) of infections together with laboratory data suggests primary viral damage rather than secondary bacterial pneumonia in lethal cases of Influenza A(H1N1)pdm09. Influenza vaccination is extremely needed for middle-aged people with above mentioned comorbid conditions.

**Disclosure of Interest**: None declared

### P295 VALIDATION OF MATHEMATICAL EQUATIONS THAT USE TO QUANTIFY THE RELATIONSHIP BETWEEN AIR AND SURFACE MICROBES

#### W. Hiwar, M.-F. King, L. Fletcher, C. Noakes, on behalf of Hospital Environment Control, Optimisation and Infection Risk Assessment (HECOIRA)

##### Civil engineering, University of Leeds, Leeds, United Kingdom

###### **Correspondence:** W. Hiwar

**Introduction:** Healthcare-associated infections (HCAIs) represent a constant threat to patients’ life and the treatment of these infections can be extremely expensive. The hospital environment itself is thought to be responsible for up to 20% of all HCAIs. The relationship between airborne pathogens, surface contamination and HCAIs remains undisclosed. Therefore, the aim of this study to validate mathematical equations that use to quantify the relationship between air and surface microbes.

**Objectives:** To validate mathematical equations that are used to quantify the relationship between air and surface microbes.

**Methods:** Two equations that connect the deposition rate of microorganisms to their concentration in the air were identified from related literature (Omelyansky, 1940; Whyte and Eaton, 2016). Both equations were derived to be given Y: deposited microbes on surface (CFU/m^2^/min) and have been validated using the given data of (Wong et al., 2011), who conducted both active and passive sampling for different parameters.

**Results:** According to Omeliansky’s equation, the predicted value of deposited microbes on the surface depends on the values of airborne microbes with a constant deposition rate of 20%. On the other hand, Whyte’s equation suggests that the deposition rate is not linear (the change in deposition rate is not constant). Using bland-Altman plot, there is a significant difference (*P*=.017) between the two measurements. The actual data follows a pattern of Whye’s equation. It gives lower values of deposition rate at a higher concentration of airborne increases; however, the standard deviation is very high. Physical and human factor could cause that fluctuation of deposition rate via the effect on survival rate, e.g. the evaporation of droplet that surrounds the microbes and air dynamic.

**Conclusion:** The equations were found to show a positive regression relationship between the number of ACC in air and the number of ACC on surface. However, the field needs a more accurate mathematical model that characterizes the impact of the physical and human factors on the correlation between air and surface microbes.

**References**

**Disclosure of Interest**: None declared

### P296 PREVALENCE OF RESPIRATORY VIRUSES CAUSING INFLUENZA-LIKE ILLNESS IN PATIENTS REFFERED TO CLINICS AND HOSPITALS IN SISTAN-BALUCHISTAN IN THE AUTUMN AND WINTER 2015-2016

#### F. Sharify Mood^1^, B. sharifimood^2^, M. metanat^2^

##### ^1^gonabad university of medical sciences, gonabad; ^2^Zahedan university of medical sciences, zahedan, Iran, Islamic Republic Of

###### **Correspondence:** F. Sharify Mood

**Introduction:** Acute respiratory viral infection is one of the commonest infection in cold seasons especially in the winter. Respiratory viruses are one of the most important causes of hospitalization and death in developing countries.

**Objectives:** Here we determined the frequency of the respiratory viruses including Influenza Viuses( A,B), Respiratory Syncytial virus type A and B (RSV-A and RSV-B), Human parainfluenza virus type 1, 2, and 3 (HPIV-1, HPIV-2 and HPIV-3) among patients with Influenza like illness( ILI) who referred to hospital and outpatient clinics.

**Methods:** This cross-sectional study was conducted to investigate frequency of some of the respiratory viruses infection in patients presenting with ILI ( fever, runny nose, cough, sore throat, body aches, fatigue). These patients were referred to clinics and hospitals of Zahedan university of medical sciences in Sistan and Baluchestan province during autumn and winter of year 2015. All samples were tested by RT-PCR and those who were negative for Influenza virus, tested for other viruses which cause the same syndrome using to Multiplex- PCR.

**Results:** Among 240 patients**,** 115 were male(47.9%) and 125 were female(52.1%). In 196 patients(81.7%), the sample was positive for influenza A. The highest number of influenza A patients was in the age group of 16-30. In 9 patients, the B-type influenza virus was detected (3.8%) and the highest number of cases was in the age group of more than 46 years. In the remaining 35 samples, none of the viruses were found.

**Conclusion:** The results showed that the higher prevalence of influenza viruses, especially type A, in respiratory diseases. This study suggests continuing surveillance, infection control and annual vaccination for Influenza.

**Disclosure of Interest**: None declared

## Poster session: Burden of healthcare-associated infection

### P297 A COLLABORATIVE APPROACH REDUCE HOSPITAL-ACQUIRED INFECTION AND COSTS IN HOSPITAL

#### I. Neves, F. Vieira, I. Devesa, B. Leite, D. Peres, A. Durães, V. Alves on behalf of “Stop Hospital Infection Project!” Group

##### Infection Control and Antimicrobial Resistance Unit, Matosinhos Local Health Unit, Matosinhos, Portugal

###### **Correspondence:** I. Neves

**Introduction:** In 2015, the Calouste Gulbenkian Foundation’s with Ministry of Healthy, support by Institute for Healthcare Improvement, proposed a collaborative approach in Portuguese Hospitals to reduce Hospital-Acquired Infections (HAI), named “Stop Hospital Infection Project!”

**Objectives:** The aim of the project is to reduce the following infections by 50% in 3 years: surgical site infection (SSI), namely hip and knee prosthesis (HKP), cholecystectomy and colon surgery in surgical wards, central line associated bacteremia (CLAB) and ventilator-associated pneumonia in intensive care and catheter associated urinary tract infection (CAUTI) in general and surgical wards. Our hospital was one of the 12 healthcare facilities that were selected to participate.

**Methods:** In order to achieve the “Stop Hospital Infection Project!” goals was carried out: Senior hospitals leadership commitment; Selected and adapted teams for each area; Learning Sessions: bundles use and PDSA construction, storyboard rounds and benchmarking; Extranet network availability where everyone reported the measures, with automatic graphing results.

**Results:** After 2 years the evaluation showed a global 50% reduction of all HAI at participating hospitals, except in colon surgery (none facility was able to reduce infection in colon surgery). In ours facility, HAI reduction was: 84% in SSI – HKP (orthopedics ward), 74% in CAUTI (two general wards), 40% in VAP and we kept in 0% de CLAB (intensive care). This allowed us to save 783.610€ at this time. Mortality, absenteeism and other adverse effects costs were not studied.

**Conclusion:** This collaborative, applying learning methodology with bundles and PDSA usage, disclosure of monthly results associated with intra/interinstitutional benchmarking, allowed to reduce HAI and associated costs. Other measures should be considered and studied to promote infection reduction in colon rectal surgery.

**References**

Reducing Hospital-Acquired Infection in Portuguese Hospitals: A collaborative approach toward Quality Improvement. Paulo Sousa; José Artur Paiva. In Health Systems Improvement across the globe. Editors Jeffrey Braithwaite et al. Taylor & Francis Editors, 2017.

**Disclosure of Interest**: None declared

### P298 THE GLOBAL POINT PREVALENCE SURVEY OF ANTIMICROBIAL CONSUMPTION AND RESISTANCE (GLOBAL-PPS): 2018 RESULTS OF ANTIMICROBIAL PRESCRIBING FOR HEALTHCARE-ASSOCIATED INFECTIONS IN TUNISIA

#### B. MNIF^1^, I. Pauwels^2^, A. Versporten^2^, H. Amri^3^, F. Medhioub^4^, A. Chtourou^5^, A. Hammami^1^, H. Goossens^2^

##### ^1^Laboratoire de Microbiologie, CHU Habib Bourguiba , Sfax, Tunisia; ^2^Laboratory of Medical Microbiology, University of Antwerp, Antwerp, Belgium; ^3^Medical Biology Laboratory, Jebeniana Hospital; ^4^ICU, Mahres Hospital, Sfax; ^5^Medical Biology Laboratory, Mtorrech regional hospital, Gabes, Tunisia

###### **Correspondence:** B. MNIF

**Introduction:** Healthcare-associated infections (HAI) are a great threat to public health.

**Objectives:** The purpose of the Global-PPS in Tunisia was to use a uniform and standardized method to assess antimicrobial (AM) use in hospitals, especially for HAI.

**Methods:** A PPS was conducted in 2018, in 4 Tunisian public hospitals. The survey collected details on AM and reasons for treatment as well as a set of quality indicators for all inpatients receiving an AM on the day of the PPS. BioMérieux provided unrestricted funding support for the survey.

**Results:** 468 patients were surveyed in 30 adult wards, of which 48.8% were on AM treatment (43% in medical wards, 47% in surgical wards and 71% in ICU). The top 3 antibiotics were amoxicillin and enzyme inhibitor (25.9%), cefotaxime (17.1%) and imipenem (10.3%). 45.1% of AM were used for community-acquired infections, 29.2% for HAI and 25.6 % for prophylaxis. The most frequently reported diagnoses for therapeutic use were pneumonia (22%) and intra−abdominal sepsis (21.3%). Reason for treatment and stop/review date were documented in 16,2% and 4.7% of cases respectively. Guidelines were missing in 90.8% of cases and 88.2% of the AM treatment was empiric. Prolonged prescribing for surgical prophylaxis (>24 hours) reached 98%.

Of all patients, 11.1% (N=52) received AM treatment for a HAI, with imipenem (29.2%), ciprofloxacin (8.3%), metronidazole (7.3%) and amikacin (7.3%) as the most common antibiotics. 34.3% (N=33) of HAI prescriptions were targeted, of which 93% were against one or more multidrug-resistant (MDR) microorganisms, including 8 ESBL- and 5 carbapenemase-producing Enterobacteriaceae. The most frequently reported HAIs were skin and soft tissue infections (25%) and pneumonia (22.9%).

**Conclusion:** This Global-PPS showed the need to implement an Antimicrobial Stewardship Program in Tunisian hospitals to improve antibiotic prescribing (e.g. by reducing carbapenem use), thus controlling MDR microorganisms.

**Disclosure of Interest**: None declared

### P299 INCREASED FINANCIAL BURDENS AND LENGTHS OF HOSPITAL STAY IN HEALTHCARE-ASSOCIATED INFECTIONS IN INTENSIVE CARE UNITS DUE TO MULTIDRUG-RESISTANT ORGANISMS AS COMPARED WITH THOSE DUE TO NON-MULTIDRUG-RESISTANT ORGANISMS: A PROPENSITY-SCORE MATCHED COHORT STUDY

#### S. Lihsiang^1^, C. I-Ling^2^, T. Ya-Fen^1^, L. Jien-Wei^1^, L. Jen-Sin^3^

##### ^1^Infection control; ^2^Department of Pharmacology, Gung Memorial Hospital, Kaohsiung; ^3^Department of Finance, I-Shou University, Kaohsiung, Taiwan

###### **Correspondence:** S. Lihsiang

**Introduction:** ICUs feature admission of critically ill patients increased chances of healthcare-associated infection (HAI), and high incidence of multidrug-resistant organism (MDRO) colonizations /infections. The impacts of HAIs due to multidrug-resistant organisms (MDRO-HAIs) have not yet been clarified.

**Objectives:** The objective of this study was to evaluate the impacts of MRDO-HAI as compared to non-MRDO-HAIs at ICUs in a large medical centre in Taiwan.

**Methods:** A 2680 facility in southern Taiwan. There were totally 12 adult ICUs. Patients aged ≧ 18 years admitted at one of these ICUs between January 2010 and December 2017 suffering either a MRDO-HAI or non-MDRO-HAI were included for propensity-score matching (PSM) for evaluation.

MDROs include MRSA, VRE, extended spectrum β-lactamases (ESBLs)-producing Entertobacteriaceae, carbapenem-resistant Entertobacteriaceae, multidrug-resistant (MDR)-*Pseudomonas aeruginosa*, and MDR-*Acinetobacter* spp. By Magiorakos et al with modifications. Mortality referred to all-cause death during hospital stay. The PSM was performed for patients of the MDRO-HAI group and those of the non-MDRO-HAI group at a 1:1 ratio. Statistical analyses were performed using the SAS software package, version 9.0 (SAS Institute Inc., NC).

**Results:** Among the overall 60 312 admissions to adult ICUs during the study period, total 1011 cases for potential PSM. Eventually, 368 pairs, significant differences were found in antibiotic cost (*P*<0.0001), overall hospitalization cost (*P*<0.0001), length of ICU stay (*P*=0.0005), and length of overall hospital stay (*P*=0.044). As compared to those in the non-MDRO-HAI group, we found that in the MRDO-HAI group, medical antibiotic cost was increased by 43%, overall hospital cost and length of ICU stay each by 15%, and length of overall hospital stay by 11%. The mortality rate between the 2 groups did not significantly differ (*P*=0.189).

**Conclusion:** These data shed light on the negative impacts of MDRO-HAIs in ICUs; the quantified financial burdens might be helpful in allocating resources for minimizing MDRO-HAIs in ICUs.

**Disclosure of Interest**: None declared

### P300 INCIDENCE PROPORTION OF HEALTHCARE-ASSOCIATED INFECTIONS FROM A NATIONWIDE SWISS POINT PREVALENCE SURVEY AFTER CORRECTING THE LENGTH BIAS

#### S. Doerken^1^, W. Zingg^2^, A. Metsini^2^, M. Wolkewitz^1^

##### ^1^Faculty of Medicine and Medical Center – University of Freiburg, Freiburg, Germany; ^2^University of Geneva Hospitals, Geneva, Switzerland

###### **Correspondence:** S. Doerken

**Introduction:** Point prevalence surveys (PPS) of healthcare-associated infections (HAIs) are length-biased whereby patients with longer hospitalization times are overrepresented.

**Objectives:** We demonstrate weighting methods to correctly estimate incidence proportions from PPS data.

**Methods:** To estimate the incidence proportion, we weight patients inversely to their length of hospital stay in a logistic regression model. We apply these methods to PPS data from Geneva (n = 1314) where additional follow-up information was available. We validate our methods using simulation data.

**Results:** For the Geneva PPS sample, naive vs. weighted estimates resulted in 47 vs. 12 days overall length of stay, 29 vs. 13 days duration of infection and an incidence proportion of 17.0% vs. 6.1%. Also the estimates of risk factors differed. For instance, an ultimately fatal McCabe score had an effect on the prevalence odds ratio of 0.90 vs. 2.26 on the incidence odds ratio. This can be explained by the McCabe score's strong effect on the discharge rate for patients without HAI but weaker effect after patients acquire infection.

**Conclusion:** Incidence estimates from a PPS can be grossly wrong when neglecting the length bias. Weighted regression models are a simple but powerful tool to allow incidence proportion estimation from prevalence data. Using imputation methods, results can be extrapolated to obtain results for the nationwide Swiss PPS study (n = 12931) where the necessary follow-up information was not available.

**References**

Fluss R et al. Correction of sampling bias in a cross‐sectional study of post‐surgical complications. Statistics in Medicine. 2013;32(14):2467-2478.

Frantal S et al. Length bias correction in one-day cross-sectional assessments–The nutritionDay study. Clinical Nutrition. 2016;35(2):522-527.

Wolkewitz M et al. Methodological challenges in using point-prevalence versus cohort data in risk factor analyses of nosocomial infections. Annals of Epidemiology. 2018;28(7):475-80.

**Disclosure of Interest**: None declared

### P301 HEALTHCARE ASSOCIATED INFECTION IN HOME HEALTHCARE; WHAT, WHY AND HOW?

#### A. Hoxha^1,2^, E. Duysburgh^2^, L. Mortgat^2^

##### ^1^European Programme for Intervention Epidemiology Training (EPIET), ECDC, Stockholm, Sweden; ^2^Department of Epidemiology and Public health, Sciensano, Brussels, Belgium

###### **Correspondence:** A. Hoxha

**Introduction:** The number of patients and clinical conditions treated in home healthcare (HHC) is increasing. Care in home setting presents many challenges, including healthcare associated infections (HAI). Currently, in Belgium, data and guidelines on the topic are lacking.

**Objectives:** Our objective was to investigate a definition of HAI in HHC, their associated risk factors and recommendations for infection prevention and control (IPC).

**Methods:** The study included three consecutive steps: i) a scoping literature review; ii) in-depth interviews with individuals involved in the field of HHC; and iii) a two-rounds Delphi survey online to reach consensus among experts on the results of the previous steps.

**Results:** The review included 47 publications. No standard definition was broadly accepted, risk factors identified were limited by methodological flaws and recommendations were therefore inconsistent. The overall evidence was weak.

Nineteen in-depth interviews were conducted, and supported the literature findings, highlighting the lack of agreed definitions, risk factors and guidelines.

The Delphi survey included 21 and 23 participants in each round, respectively, and agreement was reached on the following:

Definition: Any infection specifically linked with providing care, that develops in a patient who receives HHC from a professional healthcare worker, and occurs ≥48 hours after starting HHC.

Risk factors: hand and patient’s hygiene; untrained patients and caregivers; presence and management of invasive devices.

Recommendations: adapt and standardise existing IPC guidelines to HHC; perform a national point prevalence study to measure the burden of HAI in HHC in Belgium.

**Conclusion:** This study offers an overview of the evidence available and the field knowledge of HAI in HHC. It provides a framework to set-up a prevalence study, and drive future monitoring policies and guidelines on IPC in Belgium.

**References**

**Disclosure of Interest**: None declared

## Poster session: Methicillin-resistant Staphylococcus aureus

### P302 ANALYSIS OF MRSA SCREENING AND ITS CORRELATION WITH CLINICAL MRSA INFECTION IN ACUTE ADMISSIONS

#### C. Nye

##### Medicine, North Bristol Trust, Bristol, United Kingdom

**Introduction:** MRSA screening is a core part of infection control and is common practice for patients with risk factors for MRSA colonisation in UK hospitals. This study had the following aims:

**Objectives:** 1. To quantify the proportion of MRSA screens carried out which have positive results

2. To assess the correlation between patients who have positive screens who are found to have clinically relevant MRSA infection, with MRSA isolated from samples such as blood cultures, urine, wound swabs, or surgical specimens

3. To assess the usefulness of the current screening protocol in identifying patients who had clinical MRSA infection

**Methods:** A search was carried out using pathology software to identify all MRSA screens carried out in the Emergency Department, Medical Admission Unit and Surgical Admission Unit, over 3 months from January to March 2019. A second search was conducted to identify patient specimens from the same departments which isolated MRSA over the same period. Results were compared to patient data drawn from ICE reporting software and patient notes.

**Results:** A total of 1097 patients were screened for MRSA, of which 26 were positive (2.4%). Of the 26 MRSA positive patients, 7 patients (26.7%) were found to have MRSA in clinical specimens.

Conversely, 20 patients in total had MRSA positive specimens. Of these patients, 7 had been screened and were positive (as above), 4 had been screened and were negative, and 9 had not been screened. 6 of the 9 patients who were not screened did not meet hospital criteria for screening and were therefore appropriately omitted from screening – this accounts for 30% of the total 20 patients.

**Conclusion:** Several interesting conclusions can be drawn from these results. Firstly, the proportion of patients who screen positive to MRSA is low, but the relative percentage of these patients who have clinically relevant MRSA infection in the same admission is significant (26.7%). However, 65% patients with clinical MRSA infection were not identified on screening, due to negative screens or either inappropriate or appropriate omission from screening. Even if screening criteria had been implemented correctly throughout, 50% of patients would not have been identified. This could highlight both a problem with the sensitivity of the screen itself and the screening inclusion criteria. These findings are worth further consideration in our ongoing approach to MRSA control.

**Disclosure of Interest**: None declared

### P303 Withdrawn

### P304 EVALUATION OF MRSA SCREENING STRATEGY IN A BELGIAN TERTIARY CARE HOSPITAL

#### A. Aerssens^1^, J. Boelens^1,2,3^, P. De Waegemaeker^1^, R. Ablorh^1^, E. De Brabandere^1^, I. Leroux-Roels^1,2,3^

##### ^1^Deparment of Infection Control; ^2^Laboratory for medical Micorbiology; ^3^Department of Diagnostic Sciences, Ghent University Hospital, Ghent, Belgium

###### **Correspondence:** A. Aerssens

**Introduction:** Multidrug resistant micro-organisms (MDRO) are a constant threat to public health. They often cause more serious infections with a longer hospitalization duration and higher costs. The past few years methicillin-resistant *Staphylococcus aureus* (MRSA) incidence is decreasing worldwide. As resources are limited the current MRSA screening strategy can be questioned.

**Objectives:** Goal of this study is to evaluate the current surveillance strategy in the light of the declining trend of nosocomial MRSA infections in our hospital and the recently revised guidelines of the Belgian Superior Health Council.

**Methods:** Since 2005, a targeted screening policy for MRSA is in place in our tertiary hospital with 1062 beds.

Screening is done on pooled samples of nose, throat and perineum or on 3 different samples.

Descriptive data were collected on the MRSA screening samples and MRSA infections during a period of 1 year.

**Results:** In 2018, a total of 6411 screening samples were tested for MRSA, thereby screening 3907 patients. Of these samples, 207 were positive (3.2%), counting for 144 patients (3.7%).

Out of 118 patients with clinical samples positive, 48 (40.7%) had this sample positive within 48 hours after admission and were considered community-acquired infections. Twenty-five patients (21.2%) were missed: 7 (5.9%) should have had a screening sample according to hospital guidelines and 15 patients (12.7%) had negative screening samples despite positive clinical samples > 48 hours after admission. Only 3 patients (2.6%) were missed because they were not in the target group of the screening policy.

**Conclusion:** In 2018, 3.7% of all patients screened for MRSA were positive. With the current targeted screening strategy, only 3 patients were truly missed because they were correctly not screened. Of special concern are the 15 patients who never had a positive screening sample.

In order to reduce the number of screening samples and thereby the costs and burden for the laboratory, further investigation of the positive patients on screening and those with clinical infection is needed to see if the target group can be further narrowed.

**Disclosure of Interest**: None declared

### P305 SCREENING FOR MRSA, VRE AND CARBAPENEMASE PRODUCING ENTEROBACTERIACEAE IN TUNISIAN INTENSIVE CARE UNITS

#### H. Ghali, A. Ben Cheikh, H. Hannachi, S. Khefacha-Aissa, M. Ben Rejeb, H. Said-Latiri

##### Prevention and Care Safety, Faculty of Medicine, University of Sousse, Sousse, Tunisia

###### **Correspondence:** M. Ben Rejeb

**Introduction:** Following a growing number of patients infected by emergent highly resistant bacteria (eHBR) in our intensive care units (ICUs), we have set up a screening system for their control.

**Objectives:** Describe outcomes of eHBR screening in ICUs of Sahloul university hospital of Sousse (Tunisia)

**Methods:** Rectal and nasal swab cultures were collected to detect meticillin resistant *Staphylococcus aureus* (MRSA), Vancomycin resistant *enterococcus* (VRE) and Carbapenemase producing *Enterobacteriaceae* (CPE) among patients admitted in six ICUs of Sahloul university hospital of Sousse (Tunisia) and more than three times, at least one week apart, between 1 June and 1 December 2018.

**Results:** During the study period 174 patients were screened. Of them, 69.5% were male and 73.6% were admitted in surgical ICU. In total, 161, 152 and 35 samples were realized respectively for the detection of CPE, VRE and MSRA. These samples were positive in 15%, 8.5% and 14.3% respectively for CPE, VRE and MSRA. *Klebsiella pneumoniae* OXA 48 was the most isolated CPE (80%).

**Conclusion:** Our high rates testify the need for a sensitive, specific and cost-effective screening programm that may help in infection control by early identification of patients, thereby facilitating an informed decision about infection control interventions. However, screening cannot be substitute for the basic infection prevention and control measures such as clean hospitals, microbiologically satisfactory water and food quality, rational use of antibiotics and strict enforcement of preventative measures wherever indicated.

**Disclosure of Interest**: None declared

### P306 CARRIAGE OF METHICILLIN-RESISTANT STAPHYLOCOCCUS AUREUS AMONG HEALTHCARE WORKERS: CASE OF EMERGENCY DEPARTMENT IN A TEACHING HOSPITAL IN ABIDJAN, COTE D’IVOIRE, 2019

#### M. A. E. N'guiachi^1^, I.A.D. Yapi^1^, G.A.J. Bahan^1^, Y.M. Coulibaly-Diallo^1^, K. Horo^2^, A. Kacou-N’Douba^1,3^

##### ^1^BIOLOGIE MEDICALE-bactériologie, Centre Hospitalier et Universitaire d'Angré, ABIDJAN, Côte d'Ivoire

###### **Correspondence:** M. A. E. N'guiachi

**Introduction**. - Hospital staff can act as a source for transmission of methicillin-resistant *Staphylococcus aureus* (MRSA) when transient, or permanent colonization occurs. The aim of this study was to determine the prevalence of MRSA carriage among emergency staff.

**Material and methods**. - The study was conducted from February to April 2019 in the emergency department of the Hospital and University Center of Cocody. Two samples were taken per healthcare worker (HCWs) from hand and nose using sterile swabs. Isolation and identification of *S. aureus* was carried out following a standard microbiological method. MRSA was detected using Cefoxitin (30 μg) disc by the Kirby-Bauer method. Other antibiotics have also been tested. The zone sizes were measured and interpreted according to CA-SFM 2019.

**Results**. - From a total of 146 swabs collected from healthcare workers, 102 *Staphylococcus aureus* isolates were recovered, of which 53 at the nasal carriage (36.3%) and 49 at the hand carriage (33.6%). The prevalence of MRSA among nasal carriage and hand carriage were 60.37% and 67.3%, respectively.

From nasal carriage, high rate of resistance of MRSA isolates were also resistant to sulfamethoxazole-trimethoprim in 56.2% (p = 0.003), to ciprofloxacin in 54.8% (p = 0.08), to erythromycin 65.6% (p = 0.85) and to Kanamycin, Tobramycin and Gentamycin (KTG) in 35% (p = 10^-4^). Resistance patterns of MRSA hand carriage strains were as follows: sulfamethoxazole-trimethoprim (69.7% ; p = 10-6), ciprofloxacin (54.5% ; p = 0.005), erythromycin (63.6% ; p = 0.92) and KTG (87.9% ; p = 10^-6^).

**Conclusion**. - The high rate of MRSA carriage among healthcare workers found in this study

highlights the need to prevent MRSA transmission from HCWs to the vulnerable patient.

**Disclosure of Interest**: None declared

### P307 DIFFERENCES IN METHICILLIN RESISTANT STAPHYLOCOCCUS AUREUS NASAL CARRIAGE AMONG HEALTH CARE WORKERS OF INFECTIOUS DISEASE HOSPITAL, IN KANO-NIGERIA AND COMMUNITY MEMBERS ATTENDING THE HOSPITAL FOR THE FIRST TIME

#### I. Yusuf^1^, M. I. Getso^2^, M. Haruna^3^, N. M. Doguwa^4^, A. H. Badawi^5^

##### ^1^Microbiology; ^2^Medical Microbiology, Bayero University, Kano; ^3^Biology, Kano University of Science and Technology; ^4^Microbiology, Aminu Kano Teaching Hospital; ^5^Microbiology, Infectious Disease Hospital, Kano, Nigeria

###### **Correspondence:** I. Yusuf

**Introduction:** *Staphylococcus aureus* has been indicated to cause serious community and health care-associated infections, especially in developing countries where infection prevention and control practice is inadequate. Even though the bacteria colonizes the nasal and other human surfaces, risk of invasive type is high and it sometimes difficult to differentiate the source of its transmission between health care workers (HCW) that handle patients and members of community that attend the Infectious Disease Hospital (IDH) in Kano, Nigeria.

**Objectives:** The study aim was to compare the prevalence of methicillin-resistant *S. aureus* (MRSA) carriage among HCW of IDH and 4 categories of community members that attend the hospital before having contact with HCW.

**Methods:** A cross-sectional study of 250 participants, which comprised of HCWs, food vendors, rural dwellers, children and students. Questionnaires were given to the participants to fill and nasal swabs were collected with their consent. Swabs were cultured on mannitol salt agar for *S. aureus isolation*, and isolates were screened for MRSA using phenotypic and genotypic methods.

**Results:** Of the total cultures made, 193 (77.2%) yielded growth. The overall prevalence of nasal carriage of *S. aureus* among the participants was 35.7% while other *Staphylococcus* species accounted for 64.3%. MRSA was detected in 46 participants (18.4%) phenotypically with 8 detections in HCW, 6 among students, 18 among rural dwellers, 12 among food vendors and 2 among children. MecA gene was detected in 31 out of 46 strains. The MRSA showed high resistance (>60%) to commonly used antibiotics, but were relatively susceptible to colistin (73%) and flouroquinolones (≈60%)

**Conclusion:** In situation where infection control protocol is not followed, chances of acquiring and/or disseminating MRSA between HCW and community members, especially those attending the hospital for the first time and visitors is high. There is need for regular screening of new patients and implementation of infections prevention strategies to reduce acquisition and cross contamination to others.

**Disclosure of Interest**: None declared

### P308 SCREENING FOR METHICILLIN RESISTANT STAPHYLOCOCCUS AUREUS AND EXTENDED SPECTRUM BETA LACTAMASE PRODUCING ESCHERICHIA COLI IN RESTROOM FLOORS OF MALE AND FEMALE HOSTELS OF A PUBLIC UNIVERSITY IN KANO, NIGERIA

#### A. S. Umar^1^, I. Yusuf^2^

##### ^1^Applied Chemistry, Federal University Dutsin Ma, Katsina State, Nigeria, Dutsin Ma; ^2^Microbiology, Bayero University Kano, Kano, Nigeria

###### **Correspondence:** A. S. Umar

**Introduction: Background**: The poor conditions of restrooms in over populated hostels in most Nigerian public universities can serve as source of acquiring and transmiting infectious agents.

**Objectives:** The objective of this study is to screen the floors of restrooms for Methicillin resistant Staphylococcus aureus (MRSA) and Extended spectrum beta lactamase (ESBL) producing *E. coli* before and after routine cleaning and disinfection process and to determine the suscebtibility of the isolated organisms to commonly prescribed antibiotics.

**Methods: Methods**: For 7 days, two selected locations of restroom floors from male and female hostels were swabbed in the morning before and one hour after cleaning/disinfection. Culture, identification, antibiotic suscebtibility tests were performed using standard techniques. MRSA and ESBL-*E.coli* were detected using cefoxitin susceptibility and double disc synergy tests respectively.

**Results: Result**: *S. aureus* and *E. coli* were isolated in the restroom floors. Restroom floors near the doors were more contaminated with *S. aureus* (60% in males; 52% in females) when compared with floors near the base of the toilet seats (36% in male hostels; 32% in female hostels). Overall prevalence of *E. coli* isolated in the study was 24%, and were mostly resistant to ampicillin (87.5%), co-trimoxazole (75.0%) and amoxicillin-cloxacillin (54.1%). About 96%, 80%, 75 and 60% of *S. aureus* were resistant to ampicillin, amoxicillin-clavulanic acid, co-trimoxazole and cefuroxime. The total number of MRSA among the isolated *S aureus* was 25 (36.2%), about 64% of which are from male hostels. No ESBL production was detected among the *E. coli*. Restrooms floors were still contaminated with *S. aureus*, *E. coli* and MRSA one hour after cleaning and disinfection.

**Conclusion: Conclusion**: The study showed high level of bacteria contamination of restroom floors before and after cleaning, which indicates ineffective disinfection. Risk of acquiring and transmitting the infectious and antibiotic-resistant agents among users is high, therefore adherence to and implementation of good sanitation and infection prevention measures are needed.

**Disclosure of Interest**: None declared

### P309 QUICK DETECTION OF THE STAPHILOCOCCUS AUREUS CARRIERS BEFORE THE IMPLANT OF AN ARTICULAR PROSTHESIS DUE TO A FEMUR FRACTURE: DOES IT CONTRIBUTE TO DECREASE PERIPROSTHETIC JOINT INFECTIONS?

#### N. Benito^1^, M. L. Galvez^1^, E. Garcia^2^, J. De Caso^3^, M. Puig^4^, E. Sanchez^3^, M. Jordan^3^, T. Nolla^3^, A. Cotura^1^, E. Fernandez^1^, A. P. Cortes^1^, V. Pomar^1^, M. Gurgui^1^, P. Coll^2^, X. Crusi^3^, J. Lopez-Contreras^1^

##### ^1^Infectious Diseases Unit; ^2^Microbiology; ^3^Orthopedics and Traumatology; ^4^Emergency Department, Hospital de la Santa Creu i Sant Pau, Barcelona, Spain

###### **Correspondence:** J. Lopez-Contreras

**Introduction:** The efficacy *S. aureus* (SA) carriers decolonization has not been assessed in patients with femur fracture candidates to biarticular hemiarthroplasty (HAB).

**Objectives:** To determine, if to decolonize SA carriers is useful to prevent prosthetic joint infection (PJI) in these patient with femur fractures treated with HAB implants.

**Methods:** In 2016 a program to detect *S. aureus* carriers before HAB was implemented. Patients coming from chronic care hospitals (CCH) or nursing homes (NH) were selected. At Emergency Room, a nasal test with PCR was performed. If positive, decolonization started (daily bath with chlorhexidine and nasal mupirocin BID for 5 days) without delay of surgery. Prophylaxis with cefazolin changed to teicoplanin in SARM carriers. Prophylaxis was cefazolin (or vancomycin in allergics) until 2014; from 2015 gentamicin was added.

**Results:** Results of 6 years are shown: 4 before and the 2 after the program. In 2012-2014, *S. aureus* caused 36-50% of PJI (25-100% were SARM). In 2016-2017, 22-26% of patients with femur fracture who were treated with HBA came from CCH or NH. The *S. aureus* PCR was positive in 25-29% of tested (SARM 33-57%). The PJI decreased in 2016-2017 (5; 1.3%) compared with 2014-2015 (18; 4.8%) (p=0.004), and the PJI by S. aureus (0% vs 1.3% [5]) (p=0.027). The PJI by SARM decreased (0% vs 0.5% [2]), but not statistically significant (p=0.237).

**Table:** 

	2012	2013	2014	2015	2016	2017
**HAB n**	186	208	178	200	192	206
CCH/NH patients					42 (21.9)	54 (26.2)
PCR *S. aureus* (CCH/NH patients)					36 (87.5)	48 (88.9)
Positive *S. aureus* PCR (PCR performed)					9 (25)	14 (29.2)
PCR SARM *(S. aureus)*					3 (33.3)	8 (57.1)
**PJI**	8 (4.3)	10 (4.8)	11 (6.2)	7 (3.5)	2 (1)	3 (1.5)
*S. aureus*	4 (2.2)	5 (2.4)	4 (2.2)	1 (0.5)	0 (0)	0 (0)
SARM	4 (2.2)	3 (1.4)	1 (0.6)	1 (0.5)	0 (0)	0 (0)

**Conclusion:** The *S. aureus* carriers detection by PCR, before the HAB treatment due to femur fracture, it is related to a decrease in the PJI and PJI caused by *S. aureus.*

**Disclosure of Interest**: None declared

### P310 STAPHYLOCOCCUS AUREUS OPHTHALMIC INFECTIONS IN POLISH PATIENTS – EPIDEMIOLOGY AND VIRULENCE FACTORS

#### M. Kłos, D. Romaniszyn, A. Chmielarczyk, J. Wójkowska-Mach

##### Department of Microbiology, Jagiellonian University Collegium Medicum, Kraków, Poland

###### **Correspondence:** M. Kłos

**Introduction:** Untreated staphylococcal ocular infections may cause injuries in the ocular structure and lead to visual impairments, lesions in the anatomical ocular surface, and blindness.

**Objectives:** The aim of the study was to describe the epidemiology and virulence factors of 90 *Staphylococcus aureus* (SA) ocular infections strains with a special emphasis on ability of biofilm formation and drug resistance.

**Methods:** Antimicrobial susceptibility testing was performed by disc diffusion or the E-test method according to the current guidelines of the EUCAST. The MRSA, and MLSB phenotypes were detected. Polymerase Chain Reaction (PCR) was used to detect the *mec*A, and *mup*A genes. Erythromycin resistance genes (*erm*A, *erm*B, *erm*C, and msr) were detected by multiplex PCR. Using PCR, the presence of virulence genes were checked: *luk*E, pvl, tsst-1, *et*A, and *et*B. Biofilm formation was examined by the method with Congo red agar (CRA). On CRA, slime-producing strains formed black colonies, whereas non-producing strains developed red colonies.

**Results:** A positive result of the CRA test was accomplished in 66.2% cases; significantly more often in hospitalized strains (73.4% vs 45.4% OR 3.3, 55% CI 1.2-9.3). The most frequent virulence genes were *luk*E (72.2%) and *erm*A (15.6%). 73.4% isolates were fully susceptible. In hospitalized patients the level of resistance to at least one antimicrobial category has been identified as 40.9% and 27.2% in outpatients. Among the tested strains: 5 (6.0%) had the resistance phenotype MRSA and 22 (26.5%) the resistance phenotype MLSB. Four strains manifested both mechanisms. Erythromycin’s resistance equalled 25.3%, in those resistant to fluoroquinolones. Resistance to fluoroquinolones was 5 times more often found in ambulatory patients. All the tested isolates were vancomycin sensitive.

**Conclusion:** Biofilm formation is an important risk factor for developmental staphylococcal hospital-acquired ocular infections. Our results indicates the high efficacy of chloramphenicol and fluoroquinolones treatments, as well as the need to implement new solutions due to the aforementioned bacteria’s high resistance to neomycin and anatomic barriers difficulties.

**Disclosure of Interest**: None declared

### P311 DISTRIBUTION OF HOSPITAL-ASSOCIATED MULTI DRUG-RESISTANT STAPHYLOCOCCUS AUREUS HARBORING PVL GENE IN HOSPITALS

#### W. A. Mazi^1^, J. Yu^2^, E. Dickson^3^, G. Sandstrom^4^, A. Saeed^5^

##### ^1^Infection Prevention and Control, King Faisal Medical Complex, Taif, Saudi Arabia; ^2^Strathclyde Institute of Pharmacy and Biomedical Sciences, University of Strathclyde; ^3^Microbiology, Scottish MRSA Reference LAboratory, Glasgow, United Kingdom; ^4^Department of Laboratory Medicine, Karolinska Institutet, Stockholm, Sweden; ^5^Microbiology, University of Medical Science and Technology, Khartom, Sudan

###### **Correspondence:** W. A. Mazi

**Introduction:** Although methicillin resistant *Staphylococcus aureus* infections impose a huge risk to public health in healthcare and community settings worldwide, there remains a lack of data on antibiogramas well as genotypes in Saudi Arabia isolates. The prevalence of community and hospital-associated MRSA infections is also unclear.

**Objectives:** To assess antibiogram and genotyping of single locus *spa* and multi-locus sequencing typing (MLST) and detect Panton Valentine Leukocidine (PVL) gene, a marker for CA-MRSA infection in the isolates in Taif city of Saudi Arabia.

**Methods:** Twenty-eight clinical MRSA isolates were collected randomly from three referral hospitals in Taif, Saudi Arabia. Isolates were cultured and minimum inhibitory concentration of 17 antimicrobial agents were determined according to the British Society for Antimicrobial Chemotherapy standards using Vitek2 system (BioMerieux, USA). Polymorphic region of the *spa* and seven housekeeping genes were amplified and sequenced. *spa*-typing and MLST were analyzed using BioNumerics v.5.1 (Applied Maths). All samples were screened for presence of PVL and mecA genes using polymerase chain reaction (PCR). CA- MRSA and HA-MRSA infections were defined according to centre for disease control and prevention/National healthcare safety network (CDC/HNSN, USA) criteria.

**Results:** All isolates were susceptible to chloramphenicol, rifampicin, nitrofurantoin, teicoplanin, daptomycin and vancomycin. *T*4573/ST22 strains are most prevalent in Taif city (N=6, 21%). Three isolates (*t*363/ST240 strain) were resistant to eight antimicrobial agents. Alarmingly, most of *t*4573/ST22 strains were PVL positive, resistant to ciprofloxacin and related to HA-MRSA infections.

**Conclusion:** Evidence of emerging multi drug resistant *S. aureus* strains carrying the PVL gene circulating within hospitals, highlights the urgent need for continuous active surveillance and implementation of prevention measures.

**References**

Not applicable.

**Disclosure of Interest**: None declared

### P312 OUTCOME OF MRSA OUTBREAK INVESTIGATIONS IN MAASSTAD HOSPITAL, 2015-2018

#### M. Bolleman, I. Pruis, P. Smit, A. Sandijck, M. Damen

##### Department of Medical Microbiology and Infection Prevention, Maasstad hospital , Rotterdam, Netherlands

###### **Correspondence:** M. Bolleman

**Introduction:** In order to evaluate outbreak investigations for Methicillin Resistant Staphylococcus Aureus (MRSA) in Maasstad Hospital, Rotterdam, data from 1-1-2015 until 1-1-2019 were analysed.

**Objectives:** The goal of the analysis was to evaluate the practice of outbreak investigations as currently used in Maasstad Hospital Rotterdam.

**Methods:** In the Netherlands, a search and destroy policy for MRSA is employed. The finding of a new MRSA positive patient results in an outbreak investigation. Caretakers and other patients are at risk of acquiring or transmitting MRSA within the hospital and are included in an outbreak investigation through screening.

Samples of caretakers (nose and throat swab) or patients (nose, throat and perineum swab) were incubated in an enrichment broth and screened with PCR. PCR positive samples were subsequently cultured according to national guidelines. Additionally, s*pa* typing was performed on every first positive patient sample.

**Results:** Based on routine screening, 293 MRSA positive patients were diagnosed. These resulted in 177 outbreak investigations involving each 1 to 15 departments, 1 to 113 caretakers and 1 to 132 patients.

In total, 3341 caretakers were screened due to MRSA outbreak investigations. Of these, 9 (0,3%) were diagnosed with MRSA. Based on *spa* typing, 2 out of 9 (22,0%) caretakers were linked to the outbreak strain.

In total, 678 patients were screened due to MRSA outbreak investigations. Of these, 10 (1,5%) were diagnosed with MRSA. Based on *spa* typing, 9 out of 10 (90,0%) were linked to the outbreak strain.

**Conclusion:** The percentage positive findings among caretakers and patients is low. The majority (77,8%) of MRSA positive findings in outbreak investigations among caretakers were considered incidental findings. However, the majority of MRSA positive findings among patients were considered to be linked to the outbreak (90,0%). This research suggests that the practice of outbreak investigations as currently used in Maasstad Hospital might be interpreted as excessive and the size of outbreak investigations could be reduced.

**Disclosure of Interest**: None declared

### P313 “EVIDENCE OF NOSOCOMIAL INFECTION INCLUDING MRSA IN INDOOR DUST AND PARTICULATE MATTER”

#### Z. Athar, K. L. Yeung

##### ENVR, Hong Kong University of Science and Technology, Kowloon, Hong Kong

###### **Correspondence:** Z. Athar

**Introduction:** Today, this is well recognized that environment may facilitate transmission of several important nosocomial pathogens including drug resistant, methicillin-resistant *Staphylococcus aureus* (MRSA).The dust particles in our living environment can provide surviving and growing environment to pathogens. Hence, it is important to figure out how and to what extent these disease causing agents are present in dust particles in working environment.

**Objectives:** This study aimed; [1] Investigating the bacterial load of nosocomial infections in dust particles, [2] Identifying type of clinical isolates using different selective media, [3] Comparing the biochemical properties and effects of dust particles from different indoor environments and [4] Improving health quality for those who are exposed to contaminated dust particles.

**Methods:** The project involved dust samples from different living environments for inoculation and enumeration for total viable aerobic bacterial count and drug resistant strains (i.e. MRSA). Testing of toxicity levels of dust particles for micro-organisms as well as human cells with the help of different Bioassays including Acute toxicity, Yeast Estrogenic Screen (YES), Genotoxicity and MTT Assay for cell viability after exposure to dust samples.

**Results:** In results, particles contaminated with pathogens 5x 10^6^cfu/g of dust showed more acute toxicity. For YES, estrogenic activity was reduced due to breakdown of estrogen. In Gentoxicity and MTT Assay for A-549 the Human Alveolar Epithelial cells, dust treated with pathogens showed higher toxicity. Both treated and untreated dust samples were containing significant load of viable bacteria and MRSA showing the low efficacy of bleach as disinfectant.

**Conclusion:** As a conclusion, there is need to optimize the concentration of household cleaning products to improve safety guidelines, and give us directions to decide which cleaning products need to be abandoned completely for indoor use in order to minimize or eradicate the negative environmental health impacts. In future, the study results will also be helpful for developing new, less harmful disinfectants/cleaning products for indoor settings without any potential risk to human health.

**References**

Dancer, S.J., 2008. Importance of the environment in meticillin-resistant Staphylococcus aureus acquisition: the case for hospital cleaning. *The Lancet infectious diseases*, *8*(2), pp.101-113.

**Disclosure of Interest**: None declared

## Poster session: Vancomycin-resistant enterococci

### P314 SETTING DATABASE OF GENES OF VANCOMYCIN-RESISTANT ENTEROCOCCUS FAECIUM BY USING MULTILOCUS SEQUENCE TYPING

#### Y.-F. Tang^1^, Y.-S. Lin^1^, L.-H. Su^1^, K.-C. Kuo^2^, J.-W. Liu^1^

##### ^1^Committee of Hospital Infection Control; ^2^Department of Pediatrics, Kaohsiung Chang Gung Memorial Hospital, Kaohsiung, Taiwan

###### **Correspondence:** Y.-F. Tang

**Introduction:** *Enterococcus faecium* are normal inhabitants of the gastrointestinal tract of humans. The relative importance of *Enterococcus faecium* as a pathogen has increased with the occurrence of more immuno-compromised patients, invasive procedure utilization, and multiple antimicrobial drugs in hospital. Vancomycin-resistant *Enterococcus faecium* (VREfm) have caused hospital-case associated infections and outbreaks worldwide. Some previous studies demonstrated that sequence types(ST) 17 and 78 were the epidemic strain causing VREfm infections in Taiwan after 2007.

**Objectives:** Cases of VREfm increased significantly in our hospital. For understanding the role of VREfm sequence type, we determined the population structure of VREfm isolates in our hospital.

**Methods:** The isolates were identified by conventional methods and MALDI-TOF(MALDI Biotyper System Microflex LT; Bruker Daltonik GmbH, Bremen, Germany). MIC values of vancomycin were determined in vancomycin resistant strains by Phoenix 100 BD automated system (Becton Dickinson Diagnostic Systems, USA). Clonal relationship between strains was determined by pulsed-field gel electrophoresis (PFGE). Sequence analysis was performed for examples selected for multilocus sequence typing (MLST) of each pulsotype and subtype.

**Results:** We collected 45 isolates of VREfm from November 2018 to April 2019. VREfm were recovered from blood in 51% (n=23), from ascites in 42% (n=19) and in 7% (n=3) from pleural effusions.PFGE identified 36 unique PFGE types among the 45 isolates; MLST identified 10 unique sequence types. The sequence types are the most ST17 (44%) and the ST78 (36%).

**Conclusion:** These 45 VREfm strains were characterized into 36 different PFGE types with less similarity. The sequence types of VREfm were dominated by ST17 and ST78 in our hospital. VREfm has been in our area for some time and has accumulated genetic variation.

**Disclosure of Interest**: None declared

### P315 IMPACT OF REGIONAL CHANGES IN VANCOMYCINE-RESISTANT ENTEROCOCCUS PREVENTION AND CONTROL OF INFECTION PRACTICES ON PROVINCIAL INFECTION RATES

#### S. Masson-Roy^1^, C. Garenc^2^, J. Villeneuve^2^, D. Moisan^3^ on behalf of Surveillance provincial des infections nosocomiales - Entérocoques résistants à la vancomycine (SPIN-ERV)

##### ^1^Hotel-Dieu de Levis; ^2^Institut National de Santé Publique du Québec, Québec, ^3^CISSS du Bas-St-Laurent, Rivière-du-Loup, Canada

###### **Correspondence:** S. Masson-Roy

**Introduction:** The surveillance of vancomycin-resistant enterococcus (VRE) infections and colonizations is mandatory in hospitals in the province of Quebec since April 2011. In addition to this systematic VRE screening at admission, contact precautions are implemented for carriers. However, in 2015-2016, hospitals in one region opted to stop screening patients for VRE with the exception of patients in intensive care units (ICUs).

**Objectives:** The objective of this study is to describe the impact of regional changes in VRE prevention and control of infection practices on provincial rates.

**Methods:** Data were extracted from the mandatory surveillance of VRE on May 1^st^, 2018.

**Results:** Before the changes in screening practices, no infection had ever been observed in this region. Compared to the previous years, a decrease of 35% of patients screened at admission in 2015-2016 and of 70% in 2016-2017 was observed in this region while the provincial numbers were stable. A transitory drop in the number of colonized patients was observed. While this region represents only 3.3% of the provincial number of admissions, we observed an increase in VRE nosocomial infections corresponding to 40% of the provincial numbers in 2018-2019. Furthermore, the number of primary and secondary bacteraemia and lethality increased in this region while a drop of VRE infections and deaths were observed in the province. The increased incidence rate of VRE in this region resulted in increased provincial incidence rate compared to the previous year.

**Conclusion:** In the context of a province-wide surveillance, the cessation of VRE screening at admission, in hospitals of one region in the province of Quebec, resulted in the increase of VRE infections. While the number of admissions in this region accounts for a small proportion of provincial admissions, we observed a significant increase in the incidence rate of VRE infections due to changes in colonization screening measures in only one region.

**Disclosure of Interest**: S. Masson-Roy Consultant for: For Braaun in january 2019, C. Garenc: None declared, J. Villeneuve: None declared, D. Moisan: None declared

### P316 EFFICACY OF ALCOHOLIC PREPARATIONS FOR HAND HYGIENE AND SURFACE DISINFECTION AGAINST DIFFERENT CLINICAL / NON-CLINICAL ENTEROCOCCUS STRAINS

#### T. Koburger-Janssen

##### Hygiene Nord GmbH, Greifswald, Germany

**Introduction:** Apart from the dramatic challenges of antibiotic resistance, adaption of potentially pathogenic microorganisms to antiseptics and disinfectants is increasingly and controversially discussed, as most prominently exemplified by a publication on the tolerance of *Enterococcus faecium* VRE hospital strain ST 796 to alcoholic preparations (Pidot *et al.*, 2018).

**Objectives:** The European Standard methods for testing the bactericidal efficacy of antiseptics typically employ the related species *Enterococcus hirae* (ATCC 10541) as a reference test organism. Additionally, and primarily focussing on thermal tolerance, the *Enterococcus faecium* reference strain ATCC 6057 is tested in the context of the chemical-thermal instrument- or textile disinfection only.

In order to evaluate the above-mentioned findings on *E. faecium* ST 796 in the context of current hygiene and test standard requirements, we therefore compared the *in vitro* efficacy of commercially available alcoholic preparations against *E. hirae*, *E. faecium* ATCC 6057 and *E. faecium* in accordance with the European Standard EN 13727 (2015).

**Methods:**

Simulating surface disinfection with mechanical action (wiping), preparations were additionally analysed in the 4-field-test according to EN 16615 (2015) for their efficacy and its dependency for those three reference strains.

**Results:** Results are presented and discussed with regard to the controversy mentioned above.

**Conclusion:** Results suggest that susceptibility to antimircrobials might primarily be strain- and species dependent quite naturally already and that it rather is thourough compliance to stablished and ever-evaluated hygiene procedures that therfore matters most for infection control.

**References**

Pidot *et al.*, Sci. Transl. Med. 10, eaar6115 (2018)

**Disclosure of Interest**: T. Koburger-Janssen Grant/Research support from: B.Braun Medical AG, Switzerland

### P317 VANCOMYCIN RESISTANT ENTEROCOCCCUS: DO WE REALLY NEED CONTACT PRECAUTIONS? A MULTICENTER STUDY EXPERIENCE

#### M. I. Staneloni^1^, W. Cornistein^2^ on behalf of IACS, Y. Nuccetelli^3^, A. Garces^4^, J. Farina^5^

##### ^1^Infection Control, Hospital Italiano de Buenos Aires, Ciudad de Buenos Aires; ^2^Hospital Austral; ^3^Hospital La Plata; ^4^AMEBPA; ^5^Hospital Moreno, Buenos Aires, Argentina

###### **Correspondence:** M. I. Staneloni

**Introduction:** Recent studies have questioned the impact of contact precautions (CPs) for Vancomycin Resistant Enterococcus (VRE), demonstrating that through universal preventive measures such as hand hygiene, daily patient bath, environmental hygiene and antimicrobial control program it is possible to discontinue contact precautions without increasing the infection risk. Adverse events associated with CPs such as less contact with healthcare personnel, the psychological impact for the patient, difficulties in patient flow and higher costs question this measure even more. In this study we share the experience of 7 healthcare instituions in Argentina that no longer apply this measure for VRE.

**Objectives:** To evaluate the clinical and economic impact of discontinuing CPs for VRE.

**Methods:** Multicenter study, quasi-experimental pre-post CPs discontinuity, one year pre and year post, in adults with colonization or infection with VRE, in 7 medical surgical hospitals of Argentina with an Infection Control program and without changes in preventive measures suchs as hand hygiene compliance or enviromental hygiene.VRE infection was defined as a positive culture other than surveillance culture and clinical signs compatible for infection <48 hours after cultures for VRE were taken. We compared VRE infection episodes/1000 discharges during the first period and the second period (significant difference p 0.005). An estimate of the costs associated with CPs in the first period was made.

**Results:**

**Table:** 

Discharges 1st period	Discharges 2nd period	Patient days 1st period	Patient days 2nd period	Colonized patients 1st period	Infections 1st period	Infections 2nd period	Chi^2^
72618	73609	273758	286834	71	38	39	0.95

Cost savlsing: 11445€

**Conclusion:** In this experience we were able to discontinue CPs for patients colonized or infected with VRE without an increase in VRE infections, through the implementation of an Infection Control and Surveillance program. Additionally, the discontinuation of CPs resulted in an estimated annual savings of 11445€.

**Disclosure of Interest**: None declared

### P318 DO INFECTION CONTROL MEASURES CONTRAST THE SPREAD OF VANCOMICIN-RESISTANT ENTEROCOCCUS FAECIUM IN LONG-TERM CARE FACILITIES?

#### A. Anesi^1^, V. Rognoni^1^, S. Bracco^1^, R. Di Martino^1^, I. M. Sciabica^1^, S. Forlani^2^, G. Saba^2^, R. Accetta^3^, E. Sudati^3^, M. Ferrari^3^

##### ^1^Diagnostic Department, Laboratory of Microbiology, ASST of Lodi; ^2^Low Intensity R.I.C.C.A. Department, Low Intensity Medicine; ^3^Hygiene Department, Hygiene Unit, Lodi, Italy

###### **Correspondence:** A. Anesi

**Introduction:** Although the cost-benefit of universal screening programs for VRE colonization remains controversial, targeted VRE screening of “high risk” patients, and in “high risk” units could be implemented to protect the frailest patients from VRE infections.

**Objectives:** This study reports the evaluation of surveillance and control measures adopted to contrast VRE colonization/infection in a rehabilitation unit of Territorial Socio-Sanitary Authority (ASST) of Lodi.

**Methods:** Since August 2018, in the 40-bed rehabilitation unit of ASST of Lodi all patients are screened for VRE with an active weekly surveillance. Different standard precautions have been implemented. Microbiological surface control was performed using SRK Hygiene monitoring kit system (Copan). Disinfection with hydrogen peroxide and silver cations micronebulization (HyperDRYMist, 99Technologies) after patient discharge was introduced. The trend in colonization was measured using the coefficient of transmission, which expresses the number of transmissions/1000 days for patient at risk.

**Results:** A total of 371 patients (93% bedridden) underwent screening for VRE during the initial 9-month of observation: 278 (75%) of them had a negative rectal swab, while 93 (25%) were colonized by VRE at admission. Colonization by VRE after admission could be identified in 92 (33%) patients, with a median admission-to-colonization time of 12 days (3-80) days. Since December 2018, organizational isolation instead of spatial isolation resulted only in a slight increase of transmission. The coefficient of transmission varied from 1.32 in August raising to 1.86 in January (χ^2^=0.48, p=0.49) and subsequentely decreased to 1.17 in April (χ^2^=1.86, p=0.17). VRE was isolated in urine of 17 patients and in blood coltures of only one patient, without a clear clinical meaning.

**Conclusion:** Our data highlight the strong survival and diffusion capacity of VRE in healthcare settings. The value of VRE screening in a LTCF remains contentious: VRE colonization is of no concern, VRE are “wimpy” pathogens, VRE control is difficult and costly, however in the absence of control measures, VRE colonization rates increase.

**Disclosure of Interest**: None declared

### P319 EFFICACY OF SCREENING AND ADDITIONAL PRECAUTIONS FOR PREVENTING INTER-HOSPITAL SPREAD OF VRE.

#### M. Eyer, D. Obi, N. Troillet

##### Infectious Diseases, Valais Hospital-Central Institute, Sion, Switzerland

###### **Correspondence:** M. Eyer

**Introduction:** A large outbreak of vancomycin-resistant *Enterococcus faecium* (VRE) *vanB* ST796 is affecting several hospitals in the Canton of Bern since December 2017. As patients are regularly transferred from the affected hospitals to a 110-bed rehabilitation clinic in the Canton of Valais, this institution is regularly exposed to this clone. Therefore, the prevention policy was reinforced.

**Objectives:** To assess whether our prevention policy is effective to prevent dissemination of VRE.

**Methods:** Transferred patients were categorized as follows based on information provided by the affected hospitals: A. Known to be colonized or infected; B. Known to have been in contact with a colonized or infected patient (same room); C. Other patients were considered possible contacts. Patients in category A were isolated at admission. Those in category B were isolated preemptively at admission until 3 consecutive negative rectal swabs were obtained. Those in category C underwent 3 screening swabs as well, but preemptive isolation was stopped if the 1^st^ swab was negative. In September 2018 and January 2019 respectively, 12 and 13 patients staying in the rehabilitation clinic for >30 days on wards that had housed or were housing VRE-patients were screened. Swabs were plated on chromogenic agar plates (CHROMID VRE, bioMérieux) incubated for 48 hours. Colonies were identified as *E. faecium* using MALDI-TOF (VITEK MS, bioMérieux) and tested for *vanA* and *vanB* genes (Xpert vanA/vanB, Cepheid) before phenotypic susceptibility testing on an automated system according to EUCAST (Vitek 2, bioMérieux).

**Results:** From December 2017 to May 2019, 205 patients were transferred from the affected hospitals. Nineteen belonged to category A and 13 to category B. A total of 189 screening swabs were cultured. Three patients had clinical specimens positive for VRE; all were already known for carriage and isolated. No other inpatient screened during his hospital stay was found positive for VRE.

**Conclusion:** A rigorous policy coupled with efficient communication between hospitals could prevent intra- and inter-hospital dissemination of VRE despite numerous admissions of colonized or infected patients.

**Disclosure of Interest**: None declared

### P320 SUCCESSFUL COLLABORATION BETWEEN SWISSNOSO AND THE FEDERAL OFFICE OF PUBLIC HEALTH (FOPH) DURING A NATIONAL VANCOMYCIN RESISTANT ENTEROCOCCUS FAECIUM (VRE) OUTBREAK

#### D. Vuichard-Gysin^1^, S. Harbarth^2^, N. Troillet^3^, A. Widmer^4^ on behalf of VRE taskforce and Swissnoso, V. Masserey^5^, C. Gardiol^5^ on behalf of Federal Office of Public Health

##### ^1^Research & Development, Swissnoso, Bern; ^2^Infection Prevention & Control, University Hospital Geneva, Geneva; ^3^Infectious Diseases, Central Institute Valais Hospital, Sion; ^4^Infection Prevention & Control, University Hospital Basel, Basel; ^5^Infectious Diseases, Federal Office of Public Health, Bern, Switzerland

###### **Correspondence:** D. Vuichard-Gysin

**Introduction:** In 2018, an ongoing outbreak of VRE at the University Hospital Bern, Switzerland, disseminated to other cantons.

**Objectives:** Managing such a situation in a federal state is a challenge. The FOPH mandated Swissnoso, a group of leading experts in infection prevention and control in Switzerland, to conduct investigations.

**Methods:** To act in the most efficient way, Swissnoso set up a Task Force (TF). Since notification of VRE is not mandatory, the TF obtained data on VRE from the Swiss Centre for Antibiotic resistance (anresis.ch) and 2 main microbiology laboratories, who shared their whole genome sequencing (WGS) data. Hospitals experiencing outbreaks reported directly to the TF.

**Results:** The collaborative investigation revealed small-scale VRE outbreaks in several cantons of Switzerland. WGS proofed useful in identifying various circulating VRE clones and spatiotemporal relationships. The TF detected major shortcomings in the system: lack of pro-active screening in hospitals, lack of a consistent national surveillance and lack of communication between healthcare institutions and health authorities.

**Conclusion:** The financial and administrative support provided by the FOPH facilitated a coordinated national VRE outbreak investigation and enabled identification of deficits in epidemiological surveillance and communication. As a result, the FOPH is introducing mandatory reporting of VRE outbreaks. Cantonal Officers of Health, who are in charge of infectious disease control in Switzerland, will receive electronic updates on all outbreaks in order to pro-actively inform hospitals and contain further dissemination of VRE between facilities. In addition, a center of excellence for epidemiological investigations of nosocomial outbreaks will be launched.

**Disclosure of Interest**: None declared

### P321 ENTEROCOCCAL BLOODSTREAM INFECTIONS IN SWITZERLAND: A NATIONWIDE SURVEILLANCE STUDY

#### V. Piezzi^1^, A. Atkinson^1^, A. Kronenberg^2^, J. Marschall^1^, N. Buetti^1,3^ on behalf of Swiss Centre for Antibiotic Resistance

##### ^1^Department of Infectious Diseases, Bern University Hospital; ^2^Institute for Infectious Diseases, University of Bern, Bern, Switzerland; ^3^IAME, DeSCID team, INSERM, Université Paris Diderot and Sorbonne Paris Cité, Paris, France

###### **Correspondence:** V. Piezzi

**Introduction:** Vancomycin-resistant enterococci (VRE) are multi-drug resistant microorganisms that may cause healthcare-associated infections. Compared to bloodstream infections (BSI) due to susceptible enterococci, VRE BSI are associated with longer hospital stays and increased mortality.

**Objectives:** The aim of this study was to characterize the epidemiology of enterococcal BSI in Switzerland with a focus on VRE.

**Methods:** We retrospectively analyzed enterococcal BSI from 90 healthcare institutions from January 2013 to October 2018 using data from the Swiss antibiotic resistance centre (anresis.ch). The accuracy of anresis.ch in reporting BSI was previously verified. Only the first blood isolate with *E. faecium* or *E. faecalis* from an individual patient was considered.

We calculated the proportion of VRE among all BSI and estimated trends over time using Poisson regression models. A multivariable logistic regression was used to compare the proportion of VRE occurrence among gender, age group, hospital type (community hospital *vs* university hospital) and size (<200, 200-400, >400 beds), hospital departments (non-ICU *vs* ICU departments) and geographical regions.

**Results:** We identified 5,344 enterococcal BSI: 2,200 (41.2%) were due to *E. faecium,* whereas 3,144 (58.8%) to *E. faecalis*. Twenty-eight (1.27%) *E. faecium* BSI and one *E. faecalis* BSI (0.03%) showed vancomycin resistance. 3,566 (66.7%) BSI were observed in male patients and 2,912 (42.9%) occurred in patients >65 years. The majority of the samples (2,762, 51.5%) were observed in hospitals with > 400 beds.

The total number of cases of enterococcal BSIs slightly increased from 2013 to 2017 (p=0.06). The proportion of VRE isolates increased significantly (p<0.001), particularly in 2018.

The multivariate analysis revealed that university hospitals (Odd Ratio, OR 2.1 [1.1, 5.1], p=0.03) and ICU departments (OR 2.8 [1.2, 6.1], p=0.01) were associated with higher proportions of VRE BSI. However, neither age group nor geography had an influence on VRE BSI prevalence.

**Conclusion:** We detected an increasing proportion of BSI due to vancomycin-resistant *E. faecium* in Switzerland between 2013 and 2018. Higher VRE proportions were predominantly seen in university hospitals and ICUs departments. Prevention strategies should focus on these healthcare settings in the future.

**Disclosure of Interest**: None declared

### P322 SUCCESSFUL CONTROL OF AN OUTBREAK OF VANCOMYCIN-RESISTANT ENTEROCOCCUS FAECIUM CLONE ST796 AT THE UNIVERSITY HOSPITAL OF LAUSANNE

#### L. Senn, B. Clarivet, F. Boiron, M. Hannane, B. Grandbastien, D. S. Blanc

##### CHUV - centre hospitalier universitaire vaudois, Lausanne, Switzerland

###### **Correspondence:** L. Senn

**Introduction:** The VREfm clone ST796 has emerged as a major nosocomial clone in Australia and New Zealand. Switzerland was the first European country to report a nosocomial outbreak with this clone, affecting several hospitals in the Canton of Bern since December 2017.

**Objectives:** To describe a VREfm ST796 nosocomial outbreak that occurred at Lausanne University Hospital (CHUV) in December 2018.

**Methods:** Since the occurrence of several nosocomial VREfm outbreaks at CHUV in 2015-2016, we introduced weekly VRE screening in ICU and twice monthly in visceral and septic surgery units. In 2018, those systematic screenings highlighted an unsuspected outbreak in December. Whole genome sequencing (WGS) was performed routinely on all isolates. Whole genome multilocus sequence typing (wgMLST) was used to compare the genetic similarity between isolates (Bionumerics, v. 7.6.3).

**Results:** On December 11^th^ 2018, the systematic weekly screening in ICU revealed a new patient colonized with VREfm. Investigation of contact patients and weekly screenings of units identified 10 other cases in a surgical intermediate care unit and in two surgical units, the last case being identified on January 1^st^ 2019. Contact isolation precautions were prescribed for all cases and contacts and weekly screenings were performed. WGS revealed that the 11 cases carried the VREfm clone ST796. Moreover, all isolates were grouped within the same cluster with high genetic similarities (0 to 2 loci differences) and were very similar to isolates from Bern (< 7 loci differences), highly suggesting a link between the two outbreaks. The index case remained undetermined so far: none of the 11 patients had an obvious link with Bern, all but one lived in the canton of Vaud. However, one known ST796 patient from Bern attended our ambulatory pain clinic during the same period and we cannot excluded a transmission through healthcare personal.

**Conclusion:** A year after its first identification in Switzerland, the ST796 VREfm clone is circulating between Swiss hospitals. We showed that prompt detection and strong infection control measures, as described in recently published Swiss national guidelines, are efficacious to contain VRE outbreaks.

**Disclosure of Interest**: None declared

### P323 USING DATABASE VALIDATION TO IMPROVE SURVEILLANCE OF VANCOMYCIN-RESISTANT ENTEROCOCCI IN SWITZERLAND

#### V. Piezzi^1^, M. Gasser^2^, A. Kronenberg^2^, J. Marschall ^1^, N. Buetti^3^ on behalf of Swissnoso and anresis.ch

##### ^1^Department of Infectious Diseases, Bern University Hospital, University of Bern; ^2^Institute for Infectious Diseases, University of Bern, Bern, Switzerland; ^3^IAME, DeSCID team, INSERM, Université Paris Diderot and Sorbonne Paris Cité, Paris, France

###### **Correspondence:** M. Gasser

**Introduction:** The incidence of Vancomycin-resistant enterococci (VRE), although relevant for infection control purposes, is not systematically monitored in Switzerland. However, antibiotic resistance data from hospitals, representing at least 75% of annual inpatient days, are collected on a weekly or monthly basis by the Swiss Centre for Antibiotic Resistance (anresis.ch). Recently, due to a regional outbreak of a VRE *Enteroccocus faecium* clone ST796, a one-time nationwide survey on VRE epidemiology was conducted on behalf of the National Centre for Infection Control (Swissnoso).

**Objectives:** The aim of the current study was to compare the anresis.ch VRE data with the results from the Swissnoso survey, in order to identify gaps of detection and hereby improve the national surveillance system anresis.ch.

**Methods:** VRE data from 1 January 2015 to 31 March 2018 from 55 healthcare institutions, each represented in the anresis.ch and the Swissnoso database, were compared. Moreover, we performed a sub-analysis of VRE bloodstream infections (BSI). Wilcoxon signed rank tests were applied.

**Results:** Among the 55 healthcare institutions included in the analysis, 332 and 567 VRE detections were observed in the anresis.ch and in the Swissnoso database, respectively, resulting in a significantly higher number of VRE detections per hospital in the Swissnoso database (p=0.036). In three hospitals (5.45%) an identical number of samples was found in both databases, in 14 hospitals (25.45%) a difference of one sample was observed, in 18 institutions (32.72%) the difference was >1 sample, and from 20 hospitals (36.36%) no samples were detected in either database.

Regarding VRE BSI per hospital the difference between the two databases was not significant (p=0.28). Two hospitals (3.64%) showed consistently positive results, nine (16.36%) showed a discrepancy of one sample, three institutions (5.45%) a discrepancy of >1 sample, and 41 hospitals (74.55%) showed consistently negative results.

**Conclusion:** Discrepancies between the VRE databases of anresis.ch and Swissnoso were mainly due to missing screening samples in anresis.ch. For VRE BSI congruency was high. In order to prepare anresis.ch for a possible future role in VRE outbreak investigation we have since increased anresis.ch coverage and enforced the data transfer of screening samples.

**Disclosure of Interest**: None declared

## Poster session: Antimicrobial resistance in Low and Middle Income Countries 2

### P324 KNOWLEDGE OF ANTIMICROBIAL RESISTANCE AMONG UNDERGRADUATE STUDENTS OF AHMADU BELLO UNIVERSITY, ZARIA,NORTH-WEST NIGERIA

#### M. O. Onoja-Alexander^1^, A. A. Olorukooba^2^, B. Nwankwo^3^, K. H. Liman^2^, A. D. Onoja^4^, B. M. Matthew^5^

##### ^1^Department of Community Medicine, Kogi State University,Anyigba, Kogi State ,Nigeria, Lokoja; ^2^Department of Community Medicine, Ahmadu Bello University,Zaria,Kaduna,State, Nigeria, Zaria; ^3^Kaduna State University,Kaduna , kaduna; ^4^Department of Chemistry, Kogi State University,Anyigba, Kogi State ,Nigeria, Lokoja; ^5^Ahmadu Bello University Teaching Hospital Zaria , Zaria , Nigeria

###### **Correspondence:** M. O. Onoja-Alexander

**Introduction:** Antimicrobial resistance is one of the biggest health challenges facing the world today. Estimates suggest that 700,000 lives are lost annually to antimicrobial Resistance, and it is projected to lead to mortality of at least 10 million lives by 2050. ABR leads to increased health care costs, longer hospital stays, and limited drug options for treatment, most especially in developing countries. One of the leading causes of antimicrobial resistance is self-medication with antimicrobials.

**Objectives:** The aim of this study was to assess the knowledge of antimicrobial resistance among undergraduate students of Ahmadu Bello University, Zaria.North-Western Nigeria

**Methods:** A cross-sectional descriptive study was coducted among 416 respondents using a multi-stage sampling method and data was collected using a pre-tested, coded interviewer administered questionnaire deployed on the Open Data Kit (ODK) software version 1.16.1 installed on an android device. The data was analysed using STATA software version 13.0 (Stata Corp., College Station, TX, USA).

**Results:** Majority (76.44%) of the respondents had good knowledge of antimicrobial resistance. The Mean Knowledge score (± S.D.) of antimicrobial resistance was found to be 9.18 ± 2.61 on a scale of 16points. Self-Medication with Antimicrobials was found to be the main factor affecting the knowledge of antimicrobial resistance.

**Conclusion:** Majority of the respondents had good knowledge of antimicrobial resistance. There is still therefore the need for more educational interventions to curb the growing threat posed by antimicrobial resistance

**Disclosure of Interest**: None declared

### P325 SURVEILLANCE OF ANTIMICROBIAL RESISTANCE IN SOUTHERN CHINA IN 2017

#### J. Wang^1^, M. Zhou^2^, Z. Guo^2^, G. Huang^2^, W. Zingg^1^

##### ^1^Infection Control Program and WHO Collaborating Centre on Patient Safety, University of Geneva Hospitals and Faculty of Medicine, Geneva, Switzerland; ^2^Dong Guan Nosocomial Infection Control and Quality Improvement Centre, Dong Guan City, China

###### **Correspondence:** J. Wang

**Introduction:** Many international publications on antimicrobial resistance (AMR) in China addressed molecular characterization rather than incidence and prevalence on a broader scale.

**Objectives:** The objective of this study aimed at summarizing AMR in seven hospitals of Southern China in 2017, and to specifically calculate the incidence density of AMR in clinical context within three tertiary-care hospitals.

**Methods:** All data on antimicrobial susceptibility testing being reported to the Chinese “WHONET” were included for analysis. Resistance data of three tertiary-care hospitals were linked with patient’s data, providing specific information on healthcare-associated infections (HAIs) and community-associated infections (CAIs).

**Results:** Two secondary and five tertiary hospitals reported a total of 2,775 and 13,773 bacterial strains to WHONET, respectively. The proportions of 3^rd^ generation cephalosporin-(3GC) resistant *Escherichia coli* and *Klebsiella pneumoniae* were 43.9%(95%CI: 42.4% -45.5%) and 30.0%(27.9% -32.2%), respectively. The proportions of carbapenem (CAR)-resistant *Pseudomonas aeruginosa* and *Acinetobacter baumannii* were 20.7%(18.7% -22.9%) and 49.5%(46.0% -52.9%), respectively. The proportion of methicillin-resistant *Staphylococcus aureus* (MRSA) and vancomycin-resistant *Enterococcus faecalis* were 26.2% and 0.5%, respectively. In the three tertiary hospitals, the incidence density of HAI and CAI due to 3GC-resistant *E. coli* was 0.14(0.12-0.16) per 1,000 patient-days, and 0.35(0.32-0.38) per 100 admissions, respectively. The incidence density of HAI and CAI due to 3GC-resistant *K. pneumoniae* was 0.047(0.036-0.060) per 1,000 patient-days, and 0.089 (0.073-0.106) per 100 admissions, respectively. The incidence density of HAI and CAI due to CAR-resistant *P. aeruginosa* and *A. baumannii* together was 0.03 infections per 1,000 patient-days, and (0.016 VS 0.022) per 100 admissions, respectively. The incidence density of HAI and CAI due to MRSA was 0.023(0.015-0.032) per 1,000 patient-days, and 0.067(0.054-0.082) per 100 admissions, respectively.

**Conclusion:** Antimicrobial resistance in the seven hospitals of this study was lower than that in Chinese report, but higher than in Switzerland and Germany. However, given the findings on HAI due to AMR, transmission in the hospitals should be followed up carefully, particularly for Enterobacteria and non-fermentative bacilli.

**Disclosure of Interest**: None declared

### P326 CHARACTERIZATION OF ANTIBIOTIC RESISTANCE PROFILES OF VIBRIO PATHOGENS RECOVERED FROM TREATED WASTEWATER EFFLUENTS IN THE EASTERN CAPE PROVINCE, SOUTH AFRICA

#### C. A. Osunla^1^, A. O. Okoh^2^ on behalf of Applied and Environmental Microbiology Research Group (AEMREG) and SAMRC Microbial Water Quality Monitoring Centre, University of Fort Hare, Alice

##### ^1^Microbiology, Adekunle Ajasin University, Akungba, Nigeria; ^2^Department of Biochemistry and Microbiology, University of Fort Hare, Alice, South Africa

###### **Correspondence:** C. A. Osunla

**Background:** Treated wastewater effluent has been known to contain high loads of contaminants, including disease-causing bacteria such as *Vibrio* pathogens. The aim of this study was to evaluate the diversity and antimicrobial signatures of clinically important *Vibrio* pathogens isolated from treated effluents of two sub-urban wastewater treatment plants over a period of 12 months (December 2016 – November 2017).

**Methods:** Presumptive isolates were recovered by processing the samples with thiosulfate-citrate-bile salts-sucrose agar and later incubated at appropriately. The presumptive isolates were identified using PCR methods with emphasis on four *Vibrio* species of public health importance. The phenotypic susceptibility patterns of the confirmed isolates to 18 antibiotics were determined using the standardized Kirby Bauer disc diffusion method.

**Results:** Of the total 206 presumptive *Vibrio* species, 124 were confirmed belonging to the *Vibrio* genus. Four clinically important of *Vibrio* pathogens (*Vibrio cholerae, V. parahaemolyticus, V. mimicus* and *V. fluvialis*) were detected in 43, 16, 11 and 4% of the confirmed *Vibrio* species respectively. All the isolates were resistant to Polymixin B and Tetracycline. Multiple antibiotic resistances were observed among the confirmed isolates and genetic profiling of resistance genes identified t*et*A 36 %, *par*C 32 %, t*et*M 28 %, t*et*C 25 %, gyrA 23 %, gyrB 18 %, t*et*D 15 %, *bla*OXA-48 10 %, and *bla*IMP 8 % genes among the antimicrobial resistance determinants examined.

**Conclusion:** Based on our findings, we conclude that treated wastewater effluents are potential reservoirs of *Vibrio* species antibiotic resistance determinants. Hence, there is need for regular monitoring of the wastewater treatment plants for compliance in order to safeguard public health.

**Disclosure of Interest**: C. Osunla Other conflict with: No conflict of Interest, A. Okoh Other conflict with: No conflict of Interest

### P327 HIGH RATE OF GUT COLONIZATION WITH CTX-M-15 -NON-ST131 ESCHERICHIA COLI AMONG HEALTHY INDIVIDUALS IN RURAL NEPAL

#### S. Hosuru Subramanya^1^, I. Bairy^2^, N. Nayak^1^

##### ^1^Manipal College of Medical Sciences, Pokhara, Nepal; ^2^Manipal Academy of Higher Education, Manipal, India

###### **Correspondence:** S. Hosuru Subramanya

**Introduction:** Gut colonization with antibiotic-resistant bacteria such as extended-spectrum β-lactamase (ESBL) and/or carbapenemase-producing Enterobacteriaceae (EPE and CPE respectively) jeopardize infections.

**Objectives:** The aim of this study was to determine the EPE and CPE colonization rate and genotype prevalence among healthy individuals in rural Nepal.

**Methods:** A total of 237 rectal swabs were collected by systematic random sampling from healthy adults and children in the Kaski district of Western Nepal. Volunteers without a history of antibiotic consumption or hospitalization three months prior to recruitment were included in the study. Specimens were screened for EPE and CPE by selective plating and confirmed by phenotypic methods. The β-lactamase genes; *bla*_TEM_/*bla*_SHV_/*bla*_OXA-1_ and *bla*_CTX-M_ (phylogenetic groups 1, 2 and 9) were detected by PCR and sequencing. All ESBL positive *E. coli* isolates were screened for sequence type 131 clades (A, B, and C) by multiplex PCR.

**Results:** A total of 187 (79.2%, 95% CI 60, 86) EPE isolates were obtained from 237 specimens, which included 95 EPE (74.8%) from 118 adults, 100 EPE (78.74%) from 118 children. No CPE was isolated. Of the 187 ESBL positive strains, 172 (91.9%) were identified as *E. coli*, eleven as *Klebsiella* species (5.8%), and four (2.1%) were of *Enterobacter* and *Citrobacter* species. More than 50% of the isolates were MDR. Among non-cephalosporin antibiotics, the resistance rates were observed higher in nitrofurantoin, tetracycline, and fluoroquinolones. The bla_*TEM*_ and bla_*SHV*_ gene was detected in 13.6% of the isolates each, and bla_*OXA*_*-*_*1*_ gene in 6.5% of isolates. Twenty four percent of total strains co-harbored multiple ESBL genes. Among bla_CTX-M_ ESBL types (70.8%), bla_*CTX-M-15*_ genes were found in 95.8% isolates. Out of 172 *E. coli* isolates, 16 (9.3%) were belonging to ST131 and of clade C.

**Conclusion:** This is the first study in Western Nepal, demonstrating the EPC genotypes and clonal prevalence among gut flora of healthy individuals in community settings. Our data indicates that CTX-M-15 was the most prevalent ESBL enzyme mainly associated with *E. coli* belonging to non-ST131clones.

**Disclosure of Interest**: None declared

### P328 IMPROVE AWARENESS AND UNDERSTANDING ANTIBIOTICS RESISTANT THROUGH PARTICIPATION IN PUBLIC CULTURAL FORUMS

#### A. Ismail

##### Microbiology, National Public Health Laboratory, Khartoum, Sudan

**Introduction:** Antibiotics saving the lives of millions of people around the world, and have been a critical public health tool since the discovery of penicillin 1928.

Today, the development of resistance of antibiotics well recognized around the world which means simple infections that are treatable today may become untreatable tomorrow. Antibiotic resistance starts at the individual level and impacts the population

More attention needed where the access to antibiotics could be without prescriptions

**Objectives:** The aim of the study to increase the awareness among the public communities and create effective communication tools

**Methods:** Most active 10 Public cultural forums in Khartoum State were participate in this study, these Public cultural forums used to conduct weekly event concerning about the Sudanese music and songs for two hours

The time for music and songs reduced up to one hour and filled the remaining hour as ½ hour power point presentation about the use of antibiotics and the other ½ for questions and discussion. The key points in the presentation definition, type &classification, mode of action, method of uses guidelines and recommendations

**Results:** The idea accepted by the participants and interacts through questions, comments and discussion as well they enjoyed the music and songs

Awareness improved among the participants and many invitations from other Public cultural forums received showing they are interested to participate in the program

**Conclusion:** The idea accepted by the participants and interacts through questions, comments and discussion as well they enjoyed the music and songs

Awareness improved among the participants and many invitations from other Public cultural forums received showing they are interested to participate in the program

**Disclosure of Interest**: None declared

### P329 FOSFOMYCIN SUSCEPTIBILITY AMONG EXTENDED SPECTRUM Β LACTAMASE-PRODUCING MDR UROPATHOGENS

#### N. N. Nawar

##### Clinical Pathology, Kasr Al Aini Cairo university, Mohandesene, Giza, Egypt

**Introduction: Introduction:** The Urinary tract infection caused by Extended Spectrum β Lactamase (ESBL) producing multidrug resistant (MDR) organisms is considered an issue of great concern. Multidrug resistance results into limited therapeutic options urging the clinicians to reconsider the use of old antibiotics as Fosfomycin.

**Objectives: Objectives:** This work aimed at studying the invitro activity of fosfomycin against the ESBL MDR uropathogens

**Methods: Methods**: The ESBL production among the MDR urinary isolates was screened by Chromogenic agar and confirmed using Cephalosporin/ Clavulanate combined disk method (CAZ and CAZ/CAL) as well as by double disk synergy test. The confirmed ESBL MDR producers were selected for invitro susceptibility testing against Fosfomycin using Disk diffusion Kirby Bauer method. Statistical Analysis was done using Microsoft Excel 2010 and Statistical Package for the social Science ,version 23 for Microsoft Windows.

**Results: Results**: The ESBL producing MDR uropathogens were confirmed in 44.4 % of the total collected urinary isolates (472/1063). It was found that 36.9% (174 / 472) of the urinary isolates were resistant to all antibiotics other than Fosfomycin. The susceptibility rate to Fosfomycin among these isolates was 50% (87 / 174) which was significantly lower than that among other isolates that were not resistant to all antibiotics showing a sensitivity of 80.2 % ( 238 /298 ). The E-Coli showed a highest susceptibility incidence (93.1%) to fosfomycin.

**Conclusion: Conclusion**: The fosfomycin showed a high invitro activity against ESBL producing MDR uropathogens. E-Coli showed a significantly higher susceptibility rate to Fosfomycin than other other tested species

**Disclosure of Interest**: None declared

### P330 DETERMINATION OF ANTIBIOTIC RESISTANCE PATTERN AND PREVALENCE OF OXA-TYPE ENZYMES IN MULTIDRUG RESISTANCE ACINETOBACTER BAUMANNII CLINICAL ISOLATES, TEHRAN, IRAN.

#### F. Fallah

##### Pediatric Infections, Research Center, Research Institute for Children Health, Shahid Beheshti University of Medical Sciences, Tehran, Iran, tehran, Iran, Islamic Republic Of

**Introduction:** The appearance and spread of carbapenem-resistant *Acinetobacter baumannii* (CRAB) pose an important challenge for optimization of antibiotic therapies and outbreak preventions. Ability to produce OXA-type carbapenemases is the most prevalent mechanism for resistance to carbapenem in this opportunistic pathogen.

**Objectives:** We aimed to evaluate the prevalence of OXA-type carbapenemases among clinical isolates of *A. baumannii* in a one of the referral hospital in Tehran.

**Methods:** In this cross sectional study 90 *A. baumannii* was collected from different clinical specimens in Milad hospital, Tehran. Collected strains were identified by conventional microbiological methods. Antibiotic susceptibility testing was done according to CLSI. Oxa- 23, Oxa-48, Oxa- 58, Oxa- 40 and Oxa- 24 genes as most common Oxa type genes in *A. baumannii* were detected buy PCR. The sequencing was used to confirm PCR results.

**Results:** 90 collected *A. baumannii* was confirmed according to phenotypic methods. Colistin remains the most effective antibiotic in MDR *A. baumannii* and all of strains were susceptible to that. The most of resistance was observed against cefotaxim, ceftazidim and imipenem. 88%, 67%, 38% and 9% Oxa- 23, Oxa- 24 , Oxa-48 and Oxa- 40 were identified by PCR and sequencing, respectively.

**Conclusion:** the results of this study indicated that the presence of high rate of Oxa- 23 and Oxa- 24 in MDR *A. baumannii* and they are the most prevalent of OXA type enzyme in this cause of nosocomil infection in Iran. These genes are located on transferable genetic elements and can spread rapidly in hospital. Thus, nosocomial infection committee of hospital should consider this high prevalence of plasmid resistance gene.

**Disclosure of Interest**: None declared

### P331 PLASMID MEDIATED CEPHALOSPORIN RESISTANCE AMONG BACTERIAL ISOLATES FROM A MAJOR HEALTHCARE FACILITY IN KANO - NIGERIA

#### M. Yusha'u^1^, M. H. Musa^2^

##### ^1^Bayero University Kano, Nigeria, ^2^Bayero University, Kano, Nigeria

###### **Correspondence:** M. Yusha'u

**Introduction:** Resistance to antimicrobial agents especially extended spectrum cephalosporins is increasing globally. Plasmids are key vectors of horizontal gene transfer from one bacteria to another and enable a competent bacteria to take up naked DNA which facilitates the rapid dissemination of genes through bacterial population.

**Objectives:** Testing the sensitivity of the isolated bacteria to third and fourth generation cephalosporins.

Curing the cephalosporin-resistant isolates in order to destroy any present plasmid.

Re-testing the cured isolates to determine the loss of resistance or otherwise.

Plasmid analysis to confirm the presence of plasmid.

**Methods:** The Gram negative bacterial (301) isolates were subjected to standard biochemical tests to confirm their identity. The identified isolates were subjected to sensitivity to standard discs of Cefoperazone, Ceftazidime, Ceftriaxone, Cefotaxime and Cefepime using disc diffusion method according to Clinical Laboratory Institute criteria. Resistant isolates were further subjected to curing experiment using acridine orange by modified technique of Winkler et al. (1976) followed by re-testing the isolates for loss of antibiotic resistance.

**Results:** Of the 301 bacterial isolates tested for susceptibility, resistance was observed in 143(47.51%), 299(99.34%), 268(89.94%), 301(100.9%) and 247(82.06%) to Cefoperazone, Ceftazidime, Ceftriaxone, Cefotaxime and Cefepime respectively. On curing the resistant isolates and re-testing for sensitivity to the same antibiotics, 31(21.68%), 9(3.01%), 24(8.96%), 21(6.98%) and 23(9.31%) were found to be susceptible which indicated that their initial resistance was plasmid mediated.

**Conclusion:** The overall plasmid mediated resistances of 10.30%, 3.00%, 7.97%, 6.98% and 7.64% to the respective cephalosporins discs used in this study is a major public health concern as these antibiotics are the drugs of choice for most bacterial infections at the healthcare facility patronized by patients from the northern part of Nigeria and many neighbouring West African countries.

**References**

Winkler, U., River, W.and Wacjernagel , E. (1976): Bacterial phage and molecular genetics. Springer- Verlag, Berlin. Pp 127-132

**Disclosure of Interest**: None declared

### P332 POLYCLONALITY OF CARBAPENEM- RESISTANT KLEBSIELLA PNEUMONIAE IN EGYPTIAN HOSPITALS

#### A. R. Elmanakhly^1^, A. Elkholy^2^

##### ^1^Infection prevention and control department, Dar Alfouad hospital; ^2^Clinical Pathology, Microbiology and immunology, Faculty of medicine, Cairo university , Cairo, Egypt

###### **Correspondence:** A. R. Elmanakhly

**Introduction:** Critically ill ICU patients frequently require invasive medical devices which compromise normal skin and mucosal barriers, predisposing them to infection by carbapenem-resistant bacteria.

**Objectives:** This study aimed to, phenotypically and genotypically, screen for carbapenem-resistant Gram-negative bacteria (GNB) isolated from bloodstream infections in 4 ICUs in Egypt, to examine the genetic relatedness of the common resistance genes.

**Methods:** The study was conducted over two years in four ICUs in tertiary-care hospital in Egypt during 2017-2018. It included 130 (GNB) isolates from blood stream infection. Identification of isolates was performed using MALDI-TOF. Antimicrobial susceptibility was tested by the disk diffusion method following CLSI guidelines. Phenotypic detection of carbapenemases enzymes activity determined by modified Hodge test and Carba-NP method. Isolates were investigated for most common carbapenemases encoding genes *bla*_KPC_, *bla*_NDM_, and *bla*_OXA-48_ using multiplex PCR assay. Molecular typing of carbapenem-resistant isolates occurred by ERIC–PCR followed by sequencing for the common resistant genes.

**Results:** Bloodstream infections were primary in 63.91% and secondary in 36.09%. The most common isolates were Klebsiella pneumoniae. All isolates were ESBL producers, 38.65% of isolates were positive for modified Hodge test and 45.86% for Carba-NP detection. Multiplex PCR assay detected the *bla*_NDM_ in 44.44% of the isolates and *bla*_KPC_ in 19.44%, *bla*_NDM_ and *bla*_KPC_ were detected together in the same isolate in 5.55% while *bla*_OXA-48_ were detected in 8.54% of the isolate. ERIC PCR ruled out genetic relatedness, which excludes horizontal transmission of isolates.

**Conclusion:** High resistance rates were observed for all antibiotics including carbapenems. Though ERIC PCR showed that the resistant isolates were polyclonal, yet strict adherence to aseptic technique is essential during insertion and maintenance of central lines, as well as the implementation of antimicrobial stewardship.

**Disclosure of Interest**: None declared

### P333 AN INDIAN INITIATIVE TO SAVE CARBAPENEMS: AN INVITRO ASSESSMENT OF ANTIBIOTIC COMBINATIONS AGAINST MULTIDRUG RESISTANT GRAM NEGATIVE BACILLI

#### R. C. Tellis^,^ on behalf of yenepoya medical college

##### Hospital infection control, Yenepoya Medical College Hospital, MANGALORE, India

###### **Correspondence:** R. C. Tellis

**Introduction:** Infections caused by MDR gram negative bacilli (MDR-GNB) are a therapeutic challenge to clinicians and Combination antimicrobial therapy with empirically selected antibiotics is often used to treat these infections. This approach is poorly guided, as the antibiotics selected may not be optimal because of different killing activity. This study aims to determine in-vitro efficacy of antibiotic combinations devoid of carbapenems to explore potential synergy between antibiotics of different chemical classes.

**Objectives:** - To isolate & speciate MDRGNB

- To study MIC of ceftazidime, amikacin, imepenem and ciprofloxacin by micro-broth dilution

- To test & compare *invitro* effect of ceftazidime- amikacin, ceftazidime-ciprofloxacin, imepenem-amikacin, imepenem- ciprofloxacin combinations

**Methods:** Prospective, experimental descriptive study of 85 MDR-GNB isolated from clinical samples. MIC of ceftazidime, amikacin, imepenem and ciprofloxacin was determined by broth micro-dilution. *Invitro* effect of CAZ- AMK, CAZ-CIPRO, IMP-AMK & IMP-CIPRO combinations studied by **checker- board assay**.

**Results:** 62.35%, 27.05% & 44.70% of the MDR-GNB were ESBL, AmpC & MBL producers respectively. 27.05% co-produced multiple β-lactamases. MIC_90_ ranges for CAZ: 16- ≥1028μg/ml, Amk: 0.25 - ≥256μg/ml, CIPRO: 0.25-12μg/ml and IMP: 0.125-512μg/ml. CAZ- AMK and IMP-AMK combinations showed synergistic effect in >85% of MDR-GNB with FICI ≤0.5. Higher rates of indifference & antagonism observed with combinations having fluroquinolones.

**Conclusion:** In-vitro antimicrobial activity of antibiotic combinations having 3^rd^ or 4^th^ generation cephalosporin with aminoglycosides was comparable to that of imepenem mono or combination therapy. Combinations devoid of carbepenems to be advocated to prolong the clinical usefulness of this antibiotic group.

**References**

C Arias CA, Murray BE. Antibiotic resistant bugs in the 21st century- a clinical super challenge. N Engl J Med 2009; 360 (5) 439-43.

1. American Thoracic Society, IDSA. Guidelines for the management of adults with hospital acquired ventilator associated and healthcare associated pneumonia. Am J Respir CritCare Med 2005; 171 (4); 388-416.

2. Tamma PD, Cosgrove SE, Maragakis LL. Combination therapy for treatment of infections with gram-negative bacteria. Clin Microbiol Rev. 2012, 25 (3): 450–470.

**Disclosure of Interest**: None declared

### P334 INFECTIOUS RISK RELATED TO SALMONELLA SPP IN IMPORTED MEAT IN ALGERIA

#### A. Deriet^1^, M. berrazeg^2^, F. Mouffok^2^, R. Drali^2^

##### ^1^Microbiology; ^2^Pasteur institut of Algeria, Algiers, Algeria

###### **Correspondence:** A. Deriet

**Introduction:** Non-typhoidal *Salmonella* (NTS) remains one of the most important food-borne pathogen worldwide and presents a big challenge to public health and food safety. Globalization and the import of food, especially meat, have increased the risk linked to NTS.

**Objectives:** the infectious risk related to imported meat.

**Methods:** In the context of microbiological surveillance of imported food products, 33 NTS strains were isolated during the period 2013- 2015 according to “ISO 6579: 2002” in Pasteur Institute of Algeria. Six strains were chosen belonging to three different countries and five different serotypes to perform genomic investigations, the six strains were isolated from the frozen meats.

A paired-end 2x 250bp sequencing run was performed using the MiSeq illumina. The Nextera XT DNA library Preparation kit was used to construct libraries. Raw sequence reads were trimmed by “Trimmomatic v0.36.4”. Assembly was carried out using “Spades v1.3.1”. AMR gene presence was investigated with “Resfinder v3.1”. The presence of plasmid replicons was explored by “PlasmidFinder v2.0”. Virulence was investigated using the virulence factors database: http://www.mgc.ac.cn/VFs. Samtools SNP Phylogeny pipeline was used to calculate SNP number and build the tree of genetic distance.

**Results:** concerning AMR genes, *aac (6')-Iaa* was found in all the strains, while *qnrB19* was found only in the strain 6. Strains contained an average of 161 +/- 9 virulence factors and all contained the SPI-1 and SPI-2. Two plasmids with 100% identity were identified using BlastN on the NCBI database. A large genetic distance was detected between strains.

**Conclusion:** This study tried to highlight the infectious risk related to imported meat, and provided the first genomes of *Salmonella* spp in Algeria which should allow comparison and monitoring in *Salmonella* infections.

**Disclosure of Interest**: None declared

### P335 MOLECULAR CHARACTERIZATION OF CARBAPENEM RESISTANT ENTEROBACTERIACEAE IN TERTIARY CARE CENTER

#### S. N. Alyami, S. Aljohani

##### King Abdulazia Medical City, Riyadh, Saudi Arabia

###### **Correspondence:** S. N. Alyami

**Introduction**

Carbapenem resistant *Enterobacteriaceae* is a major challenge for microbiology laboratory to identify, given the wide variability in the capacity of clinical and public health laboratories to perform testing for the detection of carbapenemases. That’s mainly due to multiple mechanisms of resistance, including carbapenemase production ,extended spectrum beta-lactamases (ESBL)orAmpC enzymes together with porin changes,or efflux and permeability mechanisms.

In our study we evaluate the commonest gene detected at our institution; King Abdulaziz Medical City in Riyadh, Saudi Arabia; A tertiary care center that include two hospitals(Main Hospital & Children Hospital)with total capacity of 1600 beds.

**Material & Method**

A Total of 1769 Carbapenem Resistant *Enterobacteriaceae* tested using Gene Xpert® Carba-R which is a qualitative,on-demand real-time PCR test for the detection and differentiation of *bla*KPC,*bla*NDM,*bla*VIM,*bla*OXA-48,and *bla*IMP gene sequences since 2014 to up to date. We tested all samples following the manufacture’s instruction.

All suspected multi-drugs resistant *Enterobacteriaceae* tested using this method.

**Result**

The Carbapenem resistant *Enterobacteriaceae* strains were isolated from urine, respiratory tract, blood, cerebrospinal fluid and wound samples. Out of 1769 Carbapenem Resistant Enterobacteriaceae isolates are(46.8% isolates are *bla*OXA-48, 20.2%isolates are *bla*NDM ,0.73%isolates are *bla*KPC,0.39%isolates are *bla*VIM and 0.05%isolates are *bla*IMP).

The results presented suggest the rapid emergence of *blaKPC* isolates. Data shows dissemination of multiple carbapenem resistance genes as *blaOXA-48*+*blaNDM* in 6.8% of the isolates and there are 0.11%isolates exhibit both *blaOXA-*48*+ blaKPC*.

**Conclusion**

Carbapenem resistant *Enterobacteriaceae* is a global concern. Adherence to Infection Control practice, rising the awareness and rapid identification of patient once admitted to a healthcare setting are very critical tools to eliminates it’s spread in health care setting. Antimicrobial stewardship program must be activated in all institutions as carbapenem resistance start to reach to an alarming level globally.

**Disclosure of Interest**: None declared

### P336 EVALUATING THE LEVEL OF DISINFECTION AND EFFECTIVENESS OF DISINFECTANTS USED AT KABALE REGIONAL REFERRAL HOSPITAL.

#### C. Ngabirano^1^, A. M. NAMUTEBI^2^, on behalf of kabale regional referral hospital /infectious disease institute uganda

##### ^1^Internal medicine, ministry of health Uganda(Kabale Regional Referral Hospital; ^2^Internal medicine, kabale regional refferal hospital, KAMPALA/KABALE, Uganda

###### **Correspondence:** C. Ngabirano

**Background**: Disinfectants are used to destroy microorganisms that are living on the objects by destroying the cell wall or interfering with the microbe’s metabolism. At Kabale Regional Referral Hospital (KRRH), disinfectants such as Alcohol, Sodium Hypochlorite, and Chlorhexidine are used. Limited data is however available on evaluating the level of disinfection and effectiveness of the disinfectants in Hospital settings. The study was to determine the level of disinfection and effectiveness of the disinfectants used at KRRH.

**Methodology**: Working surfaces and Door handles from fifteen (15) units in the hospital were swabbed during the morning after disinfection. The samples were cultured. The isolates were gram stained and identified using biochemical tests. The disinfectants from these wards were collected and qualitative Suspension tests performed. Results were expressed as ‘growth’ or ‘no growth’.

**Results**: Of the 15 units swabbed, 80% (12/15) showed growth for *staphylococcus aureus* and *Escherishia Coli.* 66% (10/15) of the working areas exhibited growth, 6% (1/15) of the door handles sampled also showed growth. The suspension test showed 100% clearance of the bacterial cultures with Sodium Hypochlorite and Alcohol. *Escherishia coli* suspensions with Chlorohexadin showed 99.9% clearance.

**Conclusion:** Disinfectants used at Kabale regional referral Hospital are effective in killing microorganisms with Sodium Hypochlorite and Alcohol being more effective than Chlorohexadin. The Growth of microgranisms in 80% of the hospital units was probably attributed to poor dilution of disinfectants and inadequate cleaning/hand hygiene.

**Recommendation**. To Strengthen training of cleaners and other hospital staff on proper cleaning/hand hygiene techniques including proper dilution of disinfectants and more comprehensive study should be done to determine the relationship between the isolated organisms after disinfection and the common organisms causing hospital acquired infections, hence prevention.

**Disclosure of Interest**: None declared

## Poster session: Antimicrobial use and stewardship 2

### P337 KNOWLEDGE, ATTITUDE AND PERCEPTION SURVEY OF DOCTORS REGARDING ANTIBIOTIC USE AND RESISTANCE IN KARACHI, PAKISTAN

#### I. Khanum^1^, H. Ahmed ^2^, S. bhimani^3^

##### ^1^section of infectious diseases , department of medicine, Aga khan university hospital; ^2^DOW university of health sentience; ^3^JPMC, karachi, Pakistan

###### **Correspondence:** I. Khanum

**Introduction:** The increasing issue of Antimicrobial Resistance (AMR) has emerged as a major health crisis in almost all countries of the world including Pakistan.

**Objectives:** The objective of current study was to establish a better understanding of physician knowledge and beliefs, and compare distinctions in knowledge, attitude and perception of junior and senior doctors, regarding the rational use of antibiotics in a tertiary care hospital in Karachi, Pakistan.

**Methods:** A 26-question survey, divided in three sections to test knowledge, attitude and perception, was distributed among doctors in a tertiary care hospital in Karachi, Pakistan. IBM Statistical Package for Social Sciences (SPSS) was utilized for analyses.. All data was in the form of categorical values and presented as percentages. No imputation techniques were used for missing values. A p value of <0.05 was considered statistically significant.

**Results:** A total of 200 questionnaire**s** were completed. The greatest response was from the department of General Medicine (32.5%). Although majority of physicians (91.0% ) realized that antibiotic resistance is a pressing issue but significant percentage (n=94 ,47.0%) of participants admit that they over prescribe antibiotics . A substantial number (69.1% ) of junior doctors felt that patients’ demands influenced their decision of unrequired antibiotic prescription to patients but only 50.0% of seniors agreed (*p* = 0.01) on it . Physicians were not educating their patient about proper use of antibiotics , not aware of the antibiotics available in the hospital formulary and harmful effect of antibiotics use in agricultural industry .

They did not realize the importance of local antibiotic guidelines and antibiotic policy for logical use of antibiotics .

**Conclusion:** There were many gaps in knowledge and perception about AMR .Obtaining information on the knowledge and diffèrent perception of physicians about antibiotics helps in planning future intervention to improve antibiotics prescription practices and hencé decreasing AMR .This study also highlighted the importance of formulation and implementation of antibiotic stewardship program ( ASP ) and infection prevention and control program in healthcare facilities on urgent basis .

**Disclosure of Interest**: None declared

### P338 KNOWLEDGE, ATTITUDES, AND PRACTICES OF HEALTHCARE PROFESSIONALS TOWARDS ANTIMICROBIAL STEWARDSHIP AND THEIR PREDICTORS AT A TEACHING HOSPITAL, SUDAN

#### H. M. M. Azrag

##### Expanded Program on Immunization, Federal Ministry of Health, Khartoum, Sudan

**Introduction:** Antibiotic resistance is rising to dangerously high levels in all parts of the world. In 2016, a multi-sectoral international external evaluation team conducted a joint assessment of the International Health Regulation core capacities of Sudan. By the end of the assessment, antimicrobial resistance was found to score low. Antimicrobial Stewardship is an effective program used by healthcare institutions to reduce inappropriate antimicrobial use.

**Objectives:** This study was conducted to assess the knowledge, attitudes, and practices of healthcare professionals towards antimicrobial stewardship and their predictors at a Teaching Hospital in Sudan.

**Methods:** A prospective cross-sectional study was designed based on an endorsed anonymous self-administered questionnaire. The study included a sample of 128. The inclusion criteria for this study are all healthcare professionals who are willing to participate in the study excluding laboratory technicians and radiologists. Data was collected from March 1 to March 31, 2019, compiled, analyzed (using descriptive statistics and binary logistic regression) and presented using frequency table, figures, and charts.

**Results:** There were a total of 128 participants. 73 % of the respondents were females. The majority of participants had good knowledge (87.5%), 31 % had positive attitude towards antimicrobial stewardship and 33 % of Health Care Professionals had good practices related to antimicrobial stewardship. The main source of information used in dealing with infectious diseases was asking a colleague (41%) followed by standard treatment guidelines and smart phones internet (26%) and Wikipedia and medical encyclopedia (7%).

**Conclusion:** Despite the fact that the practice of Antimicrobial Stewardship is not implemented and well developed in the hospitals and healthcare facilities in Sudan, the level of Knowledge Attitude Practice towards the Antimicrobial Stewardship was good. However, in spite of the casual knowledge of our study participant’s, attitude and practice with regards to antibiotic use were found to be unsatisfactory. Therefore, we recommended to develop and adopt guidelines and policies to implement and monitor antimicrobial stewardship in all government and private health institutions countrywide.

**Disclosure of Interest**: None declared

### P339 IMPACT OF EDUCATIONAL ACTIVITIES IN YOUNG PHYSICIAN'S KNOWLEDGE ABOUT INFECTION CONTROL AND ANTIMICROBIAL PRESCRIPTION

#### R. Duro^1^, A. Silva-Pinto^1^, N. Rocha-Pereira^1^, P. Andrade^1^, J. Mourato-Torres^1^, L. Graça^1^, P. Palma-Martins^1^, D. Mota^2^, C. Lima-Alves^1^

##### ^1^Infection Control and Antimicrobial Stewardship Unit; ^2^Medical Residency Coordination, Centro Hospitalar Universitário de São João, Porto, Portugal

###### **Correspondence:** A. Silva-Pinto

**Introduction:** The knowledge transmitted in Med School does not adequately prepare young physicians in the infection control and antimicrobial use topics. Educational activities in the beginning of residency are essential to fill this lack of knowledge in clinical practice.

**Objectives:** To assess the knowledge of young physicians regarding infection control and antimicrobial prescription in the beginning of residency and to assess the impact of designed educational activities on such knowledge.

**Methods:** We designed an 8 hours theoretical and practical course on infection control and antimicrobial use practices for physicians in the first year of residency in our hospital (either foundation year or first year of specialization). We applied a 20 multiple choice questions quiz before and after that course concerning 4 topics: infection control (4 questions), microbiology (2 questions), infectious syndrome diagnosis (4 questions) and practical clinical cases about antimicrobial use (10 questions), graded from 0 to 20 (1 point per correct answer). We compared performance (global and for each topic) of young doctors before and after the 8 hours course.

**Results:** We performed this course to 164 young physicians (95 in the foundation year and 68 in the first-year specialization), splited in 5 different days. There were no differences between pre and post course questionnaires comparing foundation year and first-year specialization physicians. Compared with the pre course questionnaire, the grades of the post course were significantly higher (p<0,001; median, IQR: 16, 3 vs. 19, 1), and this improvement was consistent through the different topics (p<0,001 in all the four topics).

**Conclusion:** One year of clinical practice does not seem to improve knowledge in infection control and antimicrobial use topics. This theoretical and practical course had a positive impact in the immediate knowledge of physicians about infection control and antimicrobial use, highlighting the importance of education on these particular subjects.

**Disclosure of Interest**: None declared

### P340 ANTIMICROBIAL STEWARDSHIP IN OBSTETRIC INPATIENTS : A SYSTEMATIC REVIEW AND NARRATIVE SYNTHESIS

#### W. Elkassem^1^, B. Thomas^1^, P. Abdulrouf^1^, H. Alsoub^2^, M. Alhail^1^, D. Stewart^3^

##### ^1^Pharmacy Executive Directors Office; ^2^Infectious Disease, Hamad Medical Corporation; ^3^College of Pharmacy, Qatar University, Doha, Qatar

###### **Correspondence:** W. Elkassem

**Introduction:** Hospital Antimicrobial Stewardship Programs (ASPs) aim to optimize the antimicrobial therapy by reducing inappropriate use of antibiotics, and thereby reducing antimicrobial resistance and improving patient’s clinical outcomes and quality of care. An estimated 40-60% of pregnant women worldwide receive antibiotics during pregnancy or prior to delivery to prevent infection in both the mother and fetus that warrants having a formal ASP framework in obstetrics. A scoping review has identified scarcity of literature around ASP among the obstetric population.

**Objectives:** To describe the antimicrobial stewardship programs among different obstetric setting and to determine the impact of ASPs conducted in obstetric setting, specifically around the consumption of antibiotics, prescribing outcome and patients’ clinical outcomes.

**Methods:** We systematically searched the following databases, Cochrane, Embase and Medline (PubMed), CINAHL for studies that provided data on ASP from the inception until May 29, 2019. Studies were included for the final review only if they reported at least one if they presented data on any of these three outcomes, antibiotic consumption, prescribing outcome or clinical outcomes of patients (e.g. length of stay).

**Results:** Whilst the initial database search retrieved 392 relevant unique citations, only 11 studies were identified as potentially eligible for review and analysis. Majority of the studies originated from Nigeria (27%). Most of the studies (72%) collected data from multiple centers and involved hospitals over 500 beds. Majority of the studies were prospective in nature (54%) and the data collection period varied from three months to five years. Most of the studies reported data on prescribing outcomes (72%) followed by antibiotic consumption (63%); two studies estimated the economic impact and the compliance rate to the guideline. Owing to the heterogeneity in method and the data collection it was difficult to pool the data for analysis, however 90% of the studies reported significant impact either in terms of prescribing (72%),

**Conclusion:** Based on the findings, it is evident that obstetric settings with ASPs have achieved significant improvement in terms of rational prescribing, consumption and overall costs

**Disclosure of Interest**: None declared

### P341 EXPLORING MALTESE WOMEN’S EXPERIENCES OF URINARY TRACT INFECTION: A QUALITATIVE STUDY

#### E. Cutajar^1^, K. Currie^1^, P. Flowers^2^, A. Dickson^1^ on behalf of SHIP research Group - Safeguarding Health through Infection Prevention

##### ^1^Nursing Department - School of Health and Life Sciences, Glasgow Caledonian University; ^2^University of Glasgow, Glasgow, United Kingdom

###### **Correspondence:** K. Currie

**Introduction:** Public knowledge, attitudes and behaviours relating to Urinary Tract Infection - a highly common self-limiting infection (1) - remain under-explored, particularly in countries such as Malta. From a Health Psychology perspective, it is important to explore the perceptions and views people hold towards infections as well as the way they behave in the management of their symptoms.

**Objectives:** This study aims to better understand the views and drivers of behaviours in relation to the management of UTI in Maltese women. The secondary aim is to gain a better understanding of their knowledge and understanding of antibiotics and the drivers of antimicrobial resistance.

**Methods:** A qualitative methodology was utilised involving the conduction of semi-structured face-to-face or telephone interviews with Maltese women recruited via the dissemination of hard- and soft- copy adverts online and around the Maltese Islands. The data gathered from these interviews was analysed thematically following Braun & Clarke's (2006) guidelines.

**Results:** 19 participants were included in the analysis, which is currently underway. However, overarching themes have emerged so far with regards to the management of UTI in the Maltese context. The themes examine: understanding and experience of UTI; participant response to UTI; Help-seeking and management strategies; Interactions with and accessibility of health-care professionals; knowledge about antibiotic resistance and its result on antibiotic behaviour; UTI prevention strategies.

**Conclusion:** A general aversion towards antibiotics as well as a relative openness to alternative methods of managing UTI show promising avenues for interventions aimed at educating and empowering Maltese women in Antimicrobial stewardship with regards to their UTI management. The accessibility of Maltese healthcare can be harnessed in order to encourage women to engage in healthier antibiotic behaviours.

**References**

Colgan, R., & Williams, M. (2011). Diagnosis and treatment of acute uncomplicated cystitis. American family physician, 84(7), 771.

Braun, V., & Clarke, V. (2006). Using thematic analysis in psychology. *Qualitative Research in Psychology*, *3*(2), 77–101. https://doi.org/10.1191/1478088706qp063oa

**Disclosure of Interest**: None declared

### P342 ANTIBIOTIC AWARENESS AND PRACTICES AMONG THE PUBLIC AND PATIENTS SEEKING HEALTHCARE IN SOUTHEAST ASIA: A REVIEW

#### M. Tan

##### Medicine, NATIONAL UNIVERSITY HOSPITAL, SINGAPORE, Singapore, Singapore

**Introduction:** Southeast Asia is a known hotspot for antimicrobial resistance. However, there is limited information on public and patient knowledge on antibiotics in this region.

**Objectives:** To review current information on knowledge, attitudes and practices on antibiotics among the public and outpatients seeking health care in Southeast Asia.

**Methods:** Studies published in Medline until 01/01/2019 were identified using combinations of terms for the concepts “antibiotics”, “knowledge” and “awareness”. We also screened article reference lists. We included qualitative and quantitative English-language studies conducted in Southeast Asia on the public’s and outpatients’ knowledge on antibiotics. Southeast Asian countries were defined as Association of Southeast Asian Nations (ASEAN) member states.

**Results:** 14 studies met the inclusion criteria and were analysed. The search yielded studies from Singapore, Malaysia, Thailand, the Philippines, Laos, and Indonesia. Most were prospective, descriptive studies which used structured questionnaires that were self-administered or administered by an interviewer. Study recruitment ranged from statistical to convenience sampling in various settings, including residential communities (rural and urban) as well as outpatients seeking healthcare at hospitals, primary healthcare clinics and pharmacies. Statements used to evaluate subjects’ knowledge included: whether antibiotics are effective for bacterial and/or viral infections, whether antibiotics can have side effects and whether antibiotic misuse is linked to resistance. Studies reported varying levels of knowledge on antibiotics. Interestingly, several studies highlighted that despite public and patient awareness that antibiotics are ineffective for viral infections, there was the expectation that antibiotics be prescribed for viral infections such as upper respiratory tract infections. Some studies mentioned self-reported antibiotic misuse practices, such as patients switching doctors or buying antibiotics without a prescription to obtain antibiotics.

**Conclusion:** Southeast Asia is a heterogenous region with regard to development and healthcare standards. Despite varying levels of public and patient knowledge on antibiotics, there are worrying antibiotic use practices, which may contribute to antimicrobial resistance. Further research is required in countries with little information on public knowledge on antibiotics.

**Disclosure of Interest**: None declared

### P343 EFFECT OF MEDIA STRATEGY FOR PUBLIC AWARENESS-RAISING ACTIVITIES ON ANTIMICROBIAL RESISTANCE IN JAPAN

#### Y. Gu, Y. Fujitomo, R. Takahashi, N. Ohmagari

##### AMR Clinical Reference Center, National Center for Global Health and Medicine Hospital, Tokyo, Japan

###### **Correspondence:** Y. Gu

**Introduction:** The government of Japan published the National Action Plan (NAP) on Antimicrobial Resistance (AMR) in 2016 with a key policy of raising public awareness, and the AMR Clinical Reference Center (AMRCRC) was set up in 2017 to implement the NAP under the Ministry of Health, Labour and Welfare. The AMRCRC Information and Education Division started promotion efforts including press releases and media seminars to attract media attention for an effective campaign, with an attention peak planned for November (antibiotic awareness month in Japan).

**Objectives:** This study aimed to evaluate this media strategy for raising public awareness of AMR.

**Methods:** We counted the number of AMRCRC exposures in newspapers and magazines, television and radio programs, and web media on a monthly basis from September 2017 to March 2019 to evaluate media relations. We also measured the advertising value equivalency (AVE) as a key performance indicator to estimate the impact of media exposure. The AMR information website was launched in September 2017, and the number of page views was counted to estimate increasing interest in AMR among the general public.

**Results:** Total number of media exposures during the 19-month study period was 715 (newspapers and magazines 246, television and radio 16, web media 453). Although the monthly number of media publications tended to fluctuate, the highest number was recorded in November 2018 at 100 publications, including 6 television and radio programs. Total AVE over the study period was 116,728,000 JPY (898,000 EUR), for which the peak was also in November 2018 at 45,916,000 JPY (353,000 EUR). The total number of page views over the study period was 1.87 million with an increase from baseline to more than 140,000 page views per month. Monthly page views peaked in November 2018 (205,674 page views).

**Conclusion:** Both AVE and the number of media exposures reached record highs in November 2018 as intended. The number of page views on the AMR website indicated public attention to AMR had increased since AMRCRC activities were started. An aggressive media strategy is effective in attracting appropriate attention to the issue of AMR.

**Disclosure of Interest**: None declared

### P344 FIGHTING ANTIBIOTIC RESISTANCE: A NARRATIVE REVIEW OF PUBLIC KNOWLEDGE, ATTITUDES, AND PERCEPTIONS OF ANTIBIOTICS USE

#### A. N. Antwi^1^, A. Stewart^2^

##### ^1^Education, Health and Wellbeing, Miss; ^2^Education, Health and Wellbeing, University of Wolverhampton, Wolverhampton, United Kingdom

###### **Correspondence:** A. N. Antwi

**INTRODUCTION**: The increasing threat of Antibiotic Resistance (ABR) is, inevitably, the consequence of antibiotic use. This is largely influenced by the knowledge and perceptions of people; and their socio-economic environment.

**OBJECTIVE:** The aim of this review is to ascertain the comprehensive knowledge, perceptions, and attitudes of people from varying socio-economic regions towards antibiotic use; identify the misperceptions and malpractices; and inform health policy and practice.

**METHOD**: EBSCO Host databases, Pubmed and Google Scholar were searched to obtain relevant primary research papers within the years, 2010-2018. Search phrases included, “antibiotics use,” “community perceptions,” “public opinion, knowledge, behaviour, practices, perceptions.” Initially selected papers were screened, using the PQRS model. Methodologies of selected papers were critically analyzed. Themes were derived from the papers and analysed.

**RESULTS:** Twenty papers were selected for review. These twenty papers were a mix of both qualitative and quantitative research. Review of the twenty articles selected was based on six identified themes. It was found that insufficient knowledge and awareness of antibiotics use; self-medication and the use of left-over antibiotics; treating viral diseases with antibiotics or used as painkillers; expecting antibiotic prescription as a culmination of consultation; and the credibility of information obtained, are issues that cut across the different countries.

**CONCLUSION:** Evidence from this review suggests that misconceptions of antibiotic use are similar in different countries. Therefore, the need for the development and implementation of transferable policies as well as educating the public is necessary for the fight against ABR.

**Disclosure of Interest**: None declared

### P345 PUBLIC KNOWLEDGE, ATTITUDES, AND BEHAVIORS AROUND ANTIBIOTIC USE IN SELF-LIMITING INFECTIONS: A SCOPING REVIEW.

#### E. Cutajar^1^, K. Currie^1^, P. Flowers^2^, A. Dickson^3^ on behalf of SHIP Research Group - Safeguarding Health Through Infection Prevention

##### ^1^Nursing Department - School of Health and Life Sciences, Glasgow Caledonian University; ^2^University of Glasgow; ^3^Glasgow Caledonian University, Glasgow, United Kingdom

###### **Correspondence:** K. Currie

**Introduction:** ‘Antibiotic resistance’ (AR) is the ability of bacteria to become resistant to the action of antibiotic agents, threatening the effective prevention and treatment of an ever-growing range of infections and rendering AR an issue of local, national, and international concern (1). An exploration of public and patient perspectives of, attitudes towards, and behaviours around the use of antibiotics in self-limiting infections is necessary for discovering suitable areas for targeted action in alleviating AR through reducing antibiotic misuse.

**Objectives: To** explore existing literature pertaining to public awareness and knowledge of, attitudes towards, and behaviours around antibiotic use and resistance in SLI.

**Methods:** A scoping review was conducted on 7 electronic databases from inception until May 2017. For included studies, quantitative and qualitative data was extracted, charted and synthesized.

**Results:** 143 studies were included. Symptoms and infections of the respiratory tract were most frequently studied, followed by those of the urinary tract, and the eye. A number of key common factors that may lead to the unnecessary use of antibiotics in SLI were uncovered, including a number of misunderstandings and inappropriate attitudes towards antibiotic use in SLI. Furthermore, sociodemographic and experiential factors have also been found to influence these outcomes, including but not limited to gender; level of education; area of residence or nationality; perceived symptom severity; and past antibiotic use. The use of theoretical frameworks to satisfy study aims was observed in a small minority of included studies.

**Conclusion:** Narrowing our research focus unto areas that are relatively under-explored can help us uncover some important infection-, population-, and context- specific factors that influence inappropriate antibiotic use. The use and application of theory must be further explored if we are to give predictive and robust value to behavior change interventions.

**References**

1. Ventola CL. The antibiotic resistance crisis: part 1: causes and threats. P T A peer-reviewed J Formul Manag [Internet]. 2015;40(4):277–83. Available from: http://www.ncbi.nlm.nih.gov/pubmed/25859123%

**Disclosure of Interest**: None declared

### P346 KNOWLEDGE, ATTITUDE AND PERCEPTION SURVEY OF PHYSICIAN REGARDING ANTIBIOTIC USE AND RESISTANCE IN KARACHI, PAKISTAN

#### I. Khanum

##### Aga khan university hospital, karachi, Pakistan

**Introduction: Introduction**

The increasing issue of Antimicrobial Resistance (AMR) has emerged as a major health crisis in almost all countries of the world including Pakistan

**Objectives:** The objective of current study was to establish a better understanding of physician knowledge and beliefs, and compare distinctions in knowledge, attitude and perception of junior and senior doctors, regarding the rational use of antibiotics in a tertiary care hospital in Karachi, Pakistan.

**Methods:** A 26-question survey, divided in three sections to test knowledge, attitude and perception, was distributed among doctors in a tertiary care hospital in Karachi, Pakistan. IBM Statistical Package for Social Sciences (SPSS) was utilized for analyses.

**Results:** A total of 200 questionnaire**s** were completed. The greatest response was from the department of General Medicine (32.5%). Although majority of physicians (91.0% ) realized that antibiotic resistance is a pressing issue but only 65.5% felt confident about their antibiotic use , as a significant percentage (n=94 ,47.0%) of participants admit that they over prescribe antibiotics . A small group of young physician (19.1% ) believed that antibiotics do not cause side effects even if patients do not need them, compared to 9.1% seniors (*p* = 0.043). A substantial number (69.1% ) of junior doctors felt that patients’ demands influenced their decision of unrequired antibiotic prescription to patients but only 50.0% of seniors agreed (*p* = 0.01) on it . Physicians were not educating their patient about proper use of antibiotics , not aware of the antibiotics available in the hospital formulary and did not realize the importance of local antibiotic guidelines and antibiotic policy for logical use of antibiotics. Only 43 % of physician participated in the study were aware of the harmful effect of antibiotics use in agricultural industry . Majority of the participant favors implementation of antibiotic stewardship program and infection prevention and control policies in healthcare settings

**Conclusion:** Obtaining information on the knowledge and diffèrent perception of physicians about antibiotics helps in planning future intervention to improve antibiotics prescription practices and hencé decreasing AMR

**Disclosure of Interest**: None declared

### P347 A STUDY PROTOCOL TO ENHANCE THE KNOWLEDGE OF ANTIMICROBIAL RESISTANCE AMONG PHARMACISTS AND PHARMACY STUDENTS IN SRI LANKA

#### S. Hameem^1^, A. Bennett^2^, A. McLachlan^3^

##### ^1^University of Peradeniya, Peradeniya, Sri Lanka; ^2^NSW Therapeutic Advisory Group; ^3^The University of Sydney, Sydney, Australia

###### **Correspondence:** S. Hameem

**Introduction:** Pharmacist are experts in medicines and their education and training can have a direct impact on the quality of services they provide to patients. Antimicrobial stewardship (AMS) competencies for pharmacy students have been developed and implemented in the developed world. However, in developing countries like Sri Lanka, AMS competencies do not as yet exist for pharmacy students.

**Objectives:** Aim of this study was to prepare a protocol that includes learning outcomes and content to explore a national consensus on AMS competencies that will improve the knowledge about antibiotics and AMR for pharmacists and pharmacy students in Sri Lanka.

**Methods:** The first draft of the proposed curriculum was developed through a literature review, informed by extensive investigations of pharmacy students’ current knowledge and understanding and ensuring suitability in the Sri Lankan context. A process of liaising with academics and stakeholders in Sri Lanka and discussions with academics from developed countries (such as Australia), was used to prepare the final draft.

**Results:** The resulting curriculum covers six domains: infection and surveillance, antibiotics and antimicrobial resistance, infection and prevention control, quality use of antimicrobial agents, patient education and counselling, and inter-professional collaborative practice. This curriculum was prepared to fit the local context, aligning with recently published World Health Organization (WHO) guidelines on antimicrobial resistance (AMR) education for healthcare professionals [1].

**Conclusion:** The competencies developed will be applicable to pharmacy undergraduate programs in Sri Lankan universities and for practicing Sri Lankan pharmacists as a continuous professional development program. This protocol, once implemented, will help to strengthen AMS education amongst pharmacists in Sri Lanka and will ultimately benefit Sri Lankan consumers and assist other health care professionals addressing AMR.

**References**

1. WHO Competency Framework for Health Workers' Education and Training on Antimicrobial Resistance. Available: https://www.who.int/hrh/resources/WHO-HIS-HWF-AMR-2018.1/en/ (accessed 20 November 2018)

**Disclosure of Interest**: None declared

### P348 OVERVIEW OF ANTIMICROBIAL STEWARDSHIP AT A TERTIARY CARE OBSTETRIC ACADEMIC HOSPITAL IN THE STATE OF QATAR

#### B. Thomas^1,2^, H. Alsoub^3^, P. Abdulrouf^1^, W. Elkassem^1^, S. Muqarrabeen^1^, M. Alhail^1^

##### ^1^Pharmacy Executive Directors Office, Hamad Medical Corporation, Doha, Qatar; ^2^School of Pharmacy and Life Sciences, Robert Gordon University, Aberdeen, United Kingdom; ^3^Infectious Diseases, Hamad Medical Corporation, Doha, Qatar

###### **Correspondence:** B. Thomas

**Introduction:** Approximately 40-60% of pregnant women worldwide are prescribed with antibioticsduring prenatal, antenatal and postnatal period to prevent infection in both the mother and fetus. Antimicrobial stewardship interventions such as restriction of drugs, pre-authorization of certain antimicrobials, multidisciplinary rounds and implementation of guidelines and education have shown significant outcomes in patients.

**Objectives:** The current study aimed to demonstrate the impact (more specifically in terms of antibiotic consumption and clinical outcome) of recently implemented Antimicrobial stewardship program at a tertiary care obstetric setting in Qatar.

**Methods:** The study took place at a 300 bedded tertiary care obstetric academic center in the state of Qatar. Data was collected for three different pharmacy specific stewardship interventions, daily defined doses (DDDs) (for 11 months) of intravenous use only, quality outcome such as intravenous to oral switch (>72hrs) and patient specific outcome such as de-escalation, escalation or discontinuation of an antibiotic (2 months each).

**Results:** From May 2018 through March 2019, the DDD for all antibiotics used ranged from (253 – 293)/1000 patient days. Majority of the antibiotics were used in postnatal wards, anticipated mostly due to the post-surgical or to prevent infection. Ampicillin was used the highest followed by ceftriaxone, gentamycin, clindamycin and cefazolin. Of 188 patient files reviewed 124 patients (66%) were successfully switched to oral medications, while 62 (35%) patients had substantial justification for not switching, the remaining 2 patients (<1%) were transferred and lost follow up. Most common indication for antibiotic use was chorioamnionitis followed by urinary tract infections. In terms of patient specific outcomes, we extracted the data for 2 months specifically for restricted antibiotics (5), of 73 patients reviewed 16 (21%) patients had discontinuation of antibiotic, 14 (19%) were deescalated to a smaller antibiotic.

**Conclusion:** Antimicrobial stewardship program at the obstetric setting has shown a significant improvement in terms of antimicrobial prescribing and patient outcome. Further long-term studies including cost analysis are warranted to understand the actual impact.

**Disclosure of Interest**: None declaredpt?>

### P349 NEW PERSPECTIVE OF ANTIMICROBIAL STEWARDSHIP: CHANGING SURGEON'S PARADIGM

#### N. Kori^1^, R. Jarmin^2^, P. Periyasamy^1^, C. L. Lau^3^

##### ^1^Department of Medicine; ^2^Department of Surgery, ^3^Department of Pharmacy, UKM Medical Centre, Kuala Lumpur, Malaysia

###### **Correspondence:** N. Kori

**Introduction:** A one month observational study done in March-April 2016 found that compliance to surgical prophylaxis 2012 guideline is only 56.8%. Majority (66.1%) continued antibiotics post operatively beyond 24 hours. Many procedures in the 2012 UKMMC Anti-Infective Guideline listed third generation cephalosporins as a choice of surgical prophylaxis.

**Objectives:** To improve surgeons' compliance and antibiotic prescribing practice.

**Methods:** UKMMC AMS committee called for meetings with surgeons from all departments and disciplines, with pharmacists, infection preventionist and anaesthesiologists, chaired by head of surgery.

The multidisciplinary meetings were held in Jan, April and July 2017. Representatives were appointed to each surgical related unit to prepare the first draft according to the procedures listed in the hospital electronic system. Choices and duration of antimicrobials were reviewed by pharmacists with reference to international and local guidelines. In cases where references were not found, consensus was reached during the discussion.

Evidence for preoperative timing, intraoperative redosing and postoperative duration were presented at each discussion. Before the last draft was finalized, a meeting was held with hospital therapeutic committee, IT department and hospital OT committee to draft the implementation strategies to ensure compliance to the guideline.

**Results:** Surgical antimicrobial prophylaxis guideline was launched in September 2018. Compared to year 2012, only 10 surgical disciplines and 20 procedures were listed, in which 70% stated third generation cephalosporins as primary choice. In the revised guidelines, the number of surgical disciplines involved increases to 15, including newly added paediatrics and interventional radiology, with 351 procedures being added. Third generation cephalosporins is no longer put as choice of surgical prophylaxis but replaced with either penicillin group or cefazolin or cefuroxime, according to procedures.

**Conclusion:** In year 2019, the format of the electronic postoperative notes was revised so that the information regarding antibiotic choice, timing of administration and post operative antibiotic plan was made mandatory for regular audit and feedback purposes.

**Disclosure of Interest**: None declared

### P350 EFFICACY OF CLINICIAN-TARGETED REFRESHER COURSES FOR MANAGEMENT OF RESPIRATORY TRACT INFECTIONS TO PROMOTE APPROPRIATE USE OF ANTIBIOTICS IN OUTPATIENT SERVICES IN JAPAN

#### Y. Fujitomo^1^, S. Yamamoto^2^, Y. Gu^1^, R. Takahashi^1^, N. Ohmagari^1^

##### ^1^AMR Clinical Reference Center, National Center for Global Health and Medicine Hospital, Tokyo; ^2^Department of Infectious Diseases, Kyoto City Hospital, Kyoto, Japan

###### **Correspondence:** Y. Fujitomo

**Introduction:** The Japanese government published the National Action Plan (NAP) on Antimicrobial Resistance (AMR) in 2016. We held clinician-targeted refresher courses on management of respiratory tract infections (RTIs) in order to improve physicians’ clinical practice based on the Manual of Antimicrobial Stewardship, which was published by Ministry of Health, Labour and Welfare in 2017.

**Objectives:** To evaluate the efficacy of the clinician-targeted refresher courses for treating RTIs.

**Methods:** The refresher courses were held four times between January 2017 and December 2018. The program comprised lectures on diagnosis of and medications for RTIs and interactive training with role-play to improve communication skills. We administered pre- and post-course questionnaires and performed a vignette-based evaluation of changes in the knowledge and attitudes of the course participants in relation to prescribing antibiotics for RTIs. The questionnaires also included course feedback.

**Results:** Of the 137 participants, 122 completed the questionnaire. About half of them worked at a clinic and 70% had more than 20 years of clinical experience. We found statistically significant reductions in the number of physicians who responded that they prescribed antibiotics for 5 conditions: acute bronchitis (-34.3%; 95% confidence interval [CI] -43.4 to -25.2%), common cold (-4.6%; 95% CI -8.6% to -0.6%), acute pharyngitis (-24.8%; 95% CI -33.0% to -16.5%), acute rhinosinusitis (-22.2%; 95% CI -30.2% to -14.3%), and acute bronchitis (-3.7%;95% CI -7.3% to -0.1%). The feedback from the course participants was good.

**Conclusion:** Clinician-targeted refresher courses for the management of RTIs based on the Manual are considered effective in improving physicians’ skills for the management of acute RTIs in daily clinical practice and for promoting appropriate use of antibiotics in outpatient care.

**Disclosure of Interest**: None declared

### P351 THE ROLE OF WEB-BASED ELECTRONIC ANTIMICROBIAL PRESCRIPTION AND MONITORING SYSTEMS ON ANTIMICROBIAL STEWARDSHIP PROGRAM: A CASE STUDY IN A LARGE TEACHING HOSPITAL

#### S.-C. Pan^1^, C.-H. Tai^2^, K.-L. Tien^3^, C.-C. Chu^2^, J.-T. Wang^1^, Y.-C. Chen^1^, S.-C. Chang^1^

##### ^1^Department of Internal Medicine; ^2^Department of Pharmacy; ^3^Center for Infection Control, National Taiwan University Hospital, Taipei, Taiwan

###### **Correspondence:** S.-C. Pan

**Introduction:** An electric antimicrobial prescription system (EPS) has started since 2014 in National Taiwan University Hospital. Through the antimicrobial stewardship program (ASP), we noticed the use of teicoplanin increased dramatically.

**Objectives:** We aimed to curb improper prescription of glycopeptides by auditing prescriptions and evaluating trends of antimicrobial resistance

**Methods:** We analyzed the baseline prevalence of drug-resistance GPC and the usage of teicoplanin (DDD per 1000 inhabitants per day (DID)) since 2016. The intervention started since Sep. 1, 2018. For patients who received teicoplanin for more than 7 days, the pharmacologist (Rx) checked the prescription reason from the EPS. The assigned ID specialists reviewed clinical response and discussed with the prescription physicians. We evaluate the usage of glycopeptides by interrupted time-series analysis. (Before intervention: Jan, 1, 2016 to Aug, 31, 2018 and post intervention: Oct. 1, 2018 to March. 31, 2019)

**Results:** Before intervention, the proportion of MRSA among all clinical isolated *S. aureus* per month decreased significantly from 23.1% to 16.5% (P=0.01). The proportion of MRCNS/CNS and penicillin-resistant enterococcus/*Enterococcus spp.* also decreased significantly (both P<0.001), but the DID of teicopanin increased from 29.9 to 41.2 (P< 0.001). During intervention, there were mean 47 cases per month were reviewed. 47.0% were considered as non-culture proven by Rx and 38.1% were recognized as non-clinical indicated by ID. After feedback, 63.6% of cases stopped the teicoplanin. The DID of teicoplanin decreased after intervention (coefficient -0.4, P=0.37), even though not significantly yet, compared with before-intervention (coefficient +0.61, P<0.001). There were no balloon effect on the usage of vancomycin (coefficient=-0.06 before and -0.11 after intervention).

**Conclusion:** The intervention successfully reverse the increasing trend of teicoplanin usage. Under the current EPS, a check list is provided to identify the clinical symptoms and laboratory finding. However, individual consideration and bidirectional discussion are still needed in ASP to provide a tailored treatment for the patients.

**Disclosure of Interest**: None declared

### P352 DETERMINANTS OF THE USE OF COMPUTERIZED DECISION SUPPORT SYSTEMS AND ADHERENCE TO GUIDELINES BY PHYSICIANS FOR ANTIBIOTIC PRESCRIPTION IN HOSPITALS: A QUALITATIVE DESCRIPTIVE STUDY IN THREE HOSPITALS IN SWITZERLAND AND FRANCE.

#### G. Catho^1^, N. S. Centemero^2^, H. Catho^3^, V. Zanichelli^4^, C. Landelle^3^, P. Pavese^3^, C. Balmelli^2^, V. Prendki^1^, M. Hulscher^5^, B. Huttner^1^

##### ^1^Geneva University Hospitals and Faculty of Medicine, Geneva; ^2^Ente Ospedaliero Cantonale, Bellinzona, Switzerland; ^3^Grenoble Alpes University Hospital and Faculty of Medicine, Grenoble, France; ^4^Lady Davis Institute for Medical Research, Jewish General Hospital, Montreal, Canada; ^5^Radboud university medical center, Radboud Institute for Health Sciences, IQ healthcare, Nijmegen, Netherlands

###### **Correspondence:** G. Catho

**Introduction:** Computerized decision support systems (CDSSs) offer new perspectives for semi-automated antimicrobial stewardship interventions. Despite recent findings demonstrating the potential of local guidelines and CDSSs to improve healthcare, both are often poorly accepted by physicians.

**Objectives:** Before implementing aCDSS that helps physicians choose the appropriate antimicrobial treatment (i.e. the COMPASS tool (1)), we sought to gain an in-depth understanding of the barriers and facilitators to the use of antimicrobial guidelines and adoption of CDSS by in-hospital physicians.

**Methods:** We conducted a qualitative study using semi-structured interviews among in-hospital physicians in three public hospitals in Switzerland and France. We developed an interview guide with open-ended questions using a comprehensive checklist for determinants of healthcare professional practice (2). Physicians were recruited by convenience sampling and snowballing until data saturation was achieved. We analysed the data using Thematic Content Analysis with a mixed approach, a) identifying themes deductively from a framework and b) generating themes inductively from the data.

**Results:** We interviewed 30 physicians. The following themes emerged (a) CDSSs were seen as essential tools for our future health systems, (b) CDSSs were felt to potentially compromise physicians’ thinking, autonomy and make them lose time (c) both CDSSs and guidelines in general were felt not to be able to replace physician’s decision making in complex situations (d) CDSSs were felt to help making guidelines more accessible, (e) CDSSs uptake is influenced by software features and physician resistance to change.

**Conclusion:** Understanding determinants of guidelines use and CDSSs adoption is key before implementing such intervention. Findings concerning physicians' views and experiences about guidelines and CDSSs can be used for designing new tools and hopefully increase their adoption.

**References**

(1) Catho G et al. BMJ Open. 2018 Jun

(2) Flottorp SA et al. Implement Sci IS. 2013 Mar

**Disclosure of Interest**: None declared

### P353 BARRIERS AND ENABLERS TO IMPLEMENTING A NATIONAL ANTIMICROBIAL STEWARDSHIP PROGRAMME IN HOSPITALS: THE BEAMS PROJECT

#### K. Currie^1^, V. Ness^1^, B. Laidlaw^1^, P. Flowers^2^

##### ^1^GLASGOW CALEDONIAN UNIVERSITY; ^2^Glasgow University, Glasgow, United Kingdom

###### **Correspondence:** K. Currie

**Introduction:** Davey *et al’s.* (2017) Cochrane review reported that targeted interventions to improve antibiotic prescribing to hospital in-patients are effective in increasing compliance with antibiotic policy and reducing duration of antibiotic treatment, thereby influencing key drivers to antimicrobial resistance (AMR). Davey *et al.* concluded that future research should be directed towards exploring barriers and enablers to the implementation of antimicrobial stewardship (AMS) interventions.

**Objectives:** To identify barriers and enablers to the implementation of a national hospital-based antimicrobial stewardship (AMS) programme in Scotland.

**Methods:** Qualitative design: individual telephone interviews with 27 AMS implementation leads (infection specialist consultants, specialist pharmacists and nurses) from 14 out of 15 Scottish health regions. Fifteen focus groups with doctors, nurses, and clinical pharmacists (n=72) from five purposively selected health regions. Thematic analysis identified broad themes in the data.

**Results:** Six themes were found to be influential in implementation: ‘people matter’; ‘context, time and resources matter’; ‘knowledge, experience and confidence matters’; ‘prioritisation matters’; ‘technology matters’; and ‘feedback matters’. Key barriers and enablers related to these themes were identified and specific recommendations to address these were developed in collaboration with participants. The use of theoretical frameworks from the social sciences provided an explanation of mechanisms which support the integration of AMS in everyday practice and potential targeted intervention elements for behaviour change.

**Conclusion:** Future attention should be directed towards the organisational context and resource requirements for AMS implementation. Developing AMS leadership skills, as well as practitioner confidence and knowledge, and capitalising on the potential contribution of nurses should be considered. Technological solutions, such as electronic prescribing and other systems which provide behavioural prompts, in addition to feedback mechanisms to reinforce positive change, would be beneficial. AMR is a global health challenge; these findings have international relevance for all healthcare providers seeking evidence based recommendations to implement or strengthen AMS in the acute hospital sector.

**Disclosure of Interest**: None declared

### P354 DEVELOPMENT OF A HEALTH INFORMATION LEAFLET ON ANTIMICROBIAL RESISTANCE FOR LOW- TO SEMI-LITERATE COMMUNITY HEALTH WORKERS

#### S. Sharma^1^, R. Tandlich^1,2^, S. Srinivas^1^

##### ^1^Faculty of Pharmacy, Rhodes University, Grahamstown, South Africa; ^2^Faculty of Health Sciences, Technical University of Liberec, Liberec, Czech Republic

###### **Correspondence:** S. Sharma

**Introduction:** Antimicrobial resistance (AMR) has a major negative impact on global public health, especially in low- and middle-income countries. AMR is projected to cause up to 10 million deaths annually by 2050, with up to 4,730,000 and 4,150,000 annual deaths in Asia and Africa, respectively. South Africa is a middle-income country facing a quadruple burden of disease. With a shortage of trained practitioners able to expand on the Antimicrobial Stewardship programme, approximately 84% of the population relying on resource-limited public sector healthcare facilities, and a low life expectancy, a treatment-based approach alone is insufficient and ineffective. Culture-sensitive and context-specific educational material can reduce the literacy barrier and facilitate behavioural changes of low- to semi-literates, resulting in increased practice of health interventions, decreased health disparities and enhanced health outcomes.

**Objectives:** To design, test and implement a context-specific, end-user literacy aligned and culture-sensitive health information leaflet (HIL) on AMR for low- to semi-literate community health workers (CHWs) from seven Primary Health Care (PHC) clinics in Grahamstown, South Africa.

**Methods:** A HIL on AMR was developed based on the key areas identified from 15 semi-structured interviews and six focus group discussions with healthcare professionals and CHWs from seven PHC clinics in Grahamstown. The HIL was subjected to readability testing using seven readability formulae. It was tested for content comprehension and culture-sensitivity using the Suitability Assessment Material instrument and Patient Education Materials Assessment Tool and modified based on peer-assessment and feedback from CHWs. The HIL will be implemented through collaborative workshops with CHWs, which will be evaluated using pre- and post- workshop questionnaires.

**Results:** A thematic analysis of the data supported the need for a holistic health promotion initiative on the prevention and control of AMR. The development of the HIL resulted in aligning to the desired readability of the target group. Suitability assessment results showed that the HIL was suitable for the target population.

**Conclusion:** The HIL was suitable for the CHWs and obtained an average readability score which fell within the target range of this study.

**Disclosure of Interest**: None declared

### P355 EFFECTIVENESS OF SWITCHING FROM VANCOMYCIN TO DAPTOMYCIN AMONG PATIENTS WITH METHICILLIN-RESISTANT STAPHYLOCOCCUS AUREUS (MRSA) BLOODSTREAM INFECTIONS (BSI)

#### M. Schweizer, K. Richardson, M. V. Sarrazin, M. Goto, D. Livorsi, R. Nair, B. Alexander, B. Beck, M. Jones, M. Puig-Asensio, E. Perencevich

##### Iowa City VA Hospital, Iowa City, United States

###### **Correspondence:** M. Schweizer

**Introduction:** Vancomycin is the standard treatment in the US for MRSA BSIs. Patients may be switched from vancomycin to daptomycin if the patient is not responding well to vancomycin (i.e., persistent bacteremia or adverse reactions).

**Objectives:** To compare the effectiveness of switching to daptomycin with the effectiveness of remaining on vancomycin using propensity-score matching.

**Methods:** We evaluated patients admitted to 124 VA hospitals who had MRSA BSIs and were treated with vancomycin from 2007-2014. A logistic regression model was used to evaluate the to create a propensity score. Separate models were created for switching any time during the index admission and for early switching (i.e. within 3 days). Patients who switched to daptomycin were 1:1 matched to patients who had a similar propensity score on the day of the switch but who remained on vancomycin. The association between switching to daptomycin and 90-day mortality was assessed using chi-square tests and logistic regression models.

**Results:** 7,868 patients received vancomycin for MRSA BSIs. 610 patients switched to daptomycin during the admission, and 115 switched from vancomycin to daptomycin within 3 days of starting vancomycin. The patients who switched to daptomycin within 3 days were less likely to die compared with their matched pairs who remained on vancomycin (19% vs. 31%, p-value=0.03). This protective effect of daptomycin remained after adjusting for creatinine on the day of admission and comorbidities, in a logistic regression model (odds ratio=0.43; 95% confidence interval: 0.23, 0.83). When the 610 patients who switched to daptomycin during the admission were compared with matched controls, there was no significant association between switching to daptomycin and 90-day mortality (25% vs. 23.4%, p=0.5).

**Conclusion:** Switching to daptomycin within 3-days of receiving vancomycin may be protective against mortality. Randomized trials comparing daptomycin and vancomycin have struggled to enroll sufficient numbers of patients. Our findings suggest that early switching from vancomycin to daptomycin may improve patient outcomes.

**Disclosure of Interest**: None declared

### P356 THE EFFECT OF AN ANTIBIOTIC STEWARDSHIP PROGRAM ON THE USE OF TIGECYCLINE IN A TERTIARY CARE HOSPITAL, A BEFORE AND AFTER STUDY

#### R. A. Moghnieh^1^, D. Abdallah^1^, L. Awad^1^, M. Jadayel^2^, A. Zaiter^3^, D.-C. Awwad^3^, L. Sinno^1^, S. Al-Hassan^1^, R. Khalil^1^, S. Droubi^1^, N. Droubi^1^

##### ^1^Makassed General Hospital; ^2^Beirut Arab University; ^3^Lebanese University, Beirut, Lebanon

###### **Correspondence:** R. A. Moghnieh

**Introduction:** An antibiotic stewardship program (ASP) was established in Makassed General Hospital in September 2016. A key performance indicator of this program was a drug-oriented intervention. It consisted of restricting tigecycline (TGC) use to FDA-approved indications.

**Objectives:** We checked the effect of this ASP on TGC consumption, indications, and clinical outcome in patients (pt) treated with it during 2 years after its initiation, in comparison to 2 years before.

**Methods:** This is a retrospective chart review of all adult pt who received TGC>3 days between Jan.2012-Dec.2013 (before ASP) and Oct.2016-Dec.2018 (after ASP).

**Results:** 153 pt received TGC before ASP [26 defined daily doses (DDD)/1000 pt days (PD)] and 116 patients after it(11 DDD/1000 PD). We observed 56% decrease in the level of TGC consumption after ASP. FDA-approved indications significantly increased after ASP compared to the period before (78% vs. 19% respectively, *P*<0.001). There was a true restriction for TGC use in off-label indications: ventilator-associated pneumonia:(26% before vs. 3% after,*P*<0.001), hospital-acquired pneumonia (20% before vs. 5% after,*P*=0.001), and sepsis (9% before vs. 3% after,*P*=0.028). Prolonged TGC use >15 days significantly decreased after ASP (18% before vs. 6% after, *P*=0.005). Combination of TGC and carbapenems significantly decreased after ASP, as well as its combination with colistin. Regarding patient outcome, clinical success rates increased significantly from 48% before ASP to 66% after it (*P*=0.005). Regarding mortality, we observed a statistically significant decrease in overall mortality from 45% before ASP to 21% after it (*P*< 0.0001). Precisely, the 7-day post treatment mortality, which reflects tigecycline failure to treat the primary infection, significantly dropped from 33% before ASP to 15% after it (*P*< 0.0001).

**Conclusion:** The ASP reached its main goal through increasingly using TGC in FDA-approved indications without compromising patient outcomes. In parallel, off-label use significantly decreased in critically ill patients and those with high severity of illness scores.

**Disclosure of Interest**: None declared

### P357 DECREASING THE BROAD SPECTRUM ANTIBIOTICS UNIT SOLD:

**THE PROSPECTIVE ANTIMICROBIAL STEWARDSHIP OF RASPRO INDONESIA MODEL**

#### R. I. Natadidjaja^1,2,3^, Y. Fitra^4^, Y. B. Saroyo^4^, A. Matatula^4^, R. W. Sundariningrum^4^

##### ^1^Internal Medicine, Trisakti School of Medicine; ^2^Indonesian Society of Infection Control (INASIC); ^3^Pondok Indah-Puri Indah Hospital, ^4^Hermina Bekasi Hospital, Jakarta, Indonesia

###### **Correspondence:** R. I. Natadidjaja

**Introduction:** A copyright of Ronald Irwanto Antimicrobial Stewardship Program (RASPRO) was created as an idea for running the prospective antimicrobial stewardship in hospital setting. This was synthesized from a few kinds of studies and references with a primary concept of “The Rule of 3 PIE”. RASPRO predicted the possibilities of microorganism resistance character as a caused of infection based on the disease severity, immune status, and previous antibiotics taking, hospitalization or medical instrument exposure for antibiotic empiric using.

**Objectives:** Aim of this study was to analyze RASPRO as a tool in running antimicrobial stewardship, for reducing the ineffective antibiotic prescriptions in daily practice showed by decreasing number of broad spectrum unit sold.

**Methods:** RASPRO was developed and inspired by the selective pressure theory, a few academic journals, Carmeli Stratification for predicting any possibilities of infection caused by the multi sensitive or MDR microorganism.

RASPRO first antibiotic used flowchart form, called as RASPRO-RASAL. In the first antibiotic prescription, clinicians should answer top down-closed question in this flowchart and stop when it categorized into one type of stratification. Through this flowchart form, we tried to guide clinicians when to use broad or narrow spectrum empirically while waiting the culture result for a definitive treatment. We observed the broad spectrum antibiotics unit sold in hospital X and made some 3 months comparison before (July-September 2018) and after (October-December 2018) RASPRO-RASAL flowchart implemented.

**Results:** Three months observation and comparison before-after RASPRO-RASAL flowchart implemented, 0.5g Meropenem unit sold decreased 63.83%, 1g Meropenem decreased 75.42% while Imipenem showed 100% reduction. A 93.80% decreasing of Ceftazidime and 70.05% Cefepime unit sold also reported. Overall, we noted 76.10% broad spectrum reduced before-after RASPRO-RASAL implemented.

**Conclusion:** Decreasing of broad spectrum antibiotics unit sold was reported in 3 months after RASPRO-RASAL used.

**Disclosure of Interest**: None declared

### P358 APPROPRIATENESS OF CEFTRIAXONE USE IN MEDICAL WARDS OF UKMMC - HOW COMPLIANT ARE WE?

#### A. Ibrahim^1^, I. Naina Mohamed ^2^, C. L. Lau^3^, R. Ramli^4^, N. Kori^5^, P. Periyasamy^5^

##### ^1^Department of Medicine, UKM Medical Centre, Cheras; ^2^Department of Pharmacoepidemiology and Drug Safety Unit; ^3^Department of Pharmacy; ^4^Department of Medical Microbiology and Immunology; ^5^Department of Medicine, UKM Medical Centre, Kuala Lumpur, Malaysia

###### **Correspondence:** N. Kori

**Introduction:** In year 2010 to 2018, cephalosporin usage was the highest among all other antimicrobials group in medical wards, with ceftriaxone being the most prescribed agent.

**Objectives:** To determine the prescribing pattern of ceftriaxone in medical wards and to evaluate the appropriateness of ceftriaxone usage.

**Methods:** Inpatient records of patients receiving ceftriaxone were screened. Ceftriaxone appropriateness was evaluated based on 5 criteria: according to type of therapy (empirical,prophylactic,targeted), source of infection, total daily dose, dosing frequency, and culture & sensitivity tests based. Therapy was considered appropriate if the study participants fulfilled all the 5 criteria according to local and international antibiotic protocols. Deviation in any was regarded as inappropriate. Patients were followed-up until discharge. Clinical success was considered when there is resolution of signs and symptoms. Clinical failure was considered when there is escalation of antibiotics due to clinical worsening.

**Results:** 325 patients were included. Ceftriaxone use was empirical in 92.9% of patients and one third was indicated for pneumonia (35.4%). Majority was prescribed 2g total daily dose (94.5%) and once daily (96%). Median duration of treatment was 5.0 (IQR 4.0-7.0) days. Blood culture and sensitivity test was done in 97.8% of patients. Overall, inappropriate use of ceftriaxone was 55.7%, mostly attributed by inappropriate indication (41.8%) and type of therapy (41.8%). Factors associated with appropriateness are: concomittant antibiotics, (AOR 2.73, 95% CI (1.55-4.80), P <0.001), length of stay (P<0.023) and duration of therapy (P<0.026). 59.7% (194/325) achieved good clinical outcome, but 13.2% had clinical failure and 1.2% had bacteraemia related mortality.

**Conclusion:** Inappropriate use of ceftriaxone was high and almost half had no indication to initiate antibiotic. Measures like diagnostic stewardship need to be taken to improve on the antibiotics presribing practice.

**Disclosure of Interest**: None declared

### P359 DOSAGE ADEQUACY OF SODIUM COLISTIMETHATE PRESCRIPTION

#### J. M. Torres^1^, L. Graça^2^, A. Pinto^2,3^, F. Almeida^2^, N. Pereira^2,3^, P. Andrade^2,3^, R. Duro^2,3^, C. Alves^2,3^

##### ^1^Infectious Diseases, Hospital Egas Moniz, Lisboa; ^2^Infectious Diseases; ^3^Infection Control and Antibiotic Stewardship Unit, Centro Hospitalar e Universitário de São João, Porto, Portugal

###### **Correspondence:** J. M. Torres

**Introduction:** Sodium Colistimethate is one of the last therapeutic options for the treatment of some multidrug resistant organisms. Correct posology is critical for an adequate pharmacokinetics and pharmacodynamics parameters (PK/PD), avoiding therapeutic failure and emergence of resistance.

**Objectives:** To evaluate the adequacy of Sodium Colistimethate prescriptions concerning loading dose and maintenance dose adjusted to creatinine clearance.

**Methods:** Retrospective cohort study of Sodium Colistimethate prescriptions in a single Tertiary Care Hospital from 2015 to 2018. Data were collected through clinical process evaluation and revision. Adequate loading dose was defined as 9 million internation units (MIU) irrespective of estimated creatinine clearance (CrCl). Adequate daily maintenance dose was defined as: 9 MIU for CrCl > 50 mL/min; 5.5 to 7.5 MIU for CrC 30 - 50mL/min; 4.5 to 5.5MIU for ClCr 30 - 10mL/min; 3.5 MIU for CrCl <10mL/min and for patients in hemodyalisis 2.25 MIU, except for dyalisis days when it should be administered 3 MIU after dyalisis. (1)

**Results:** We evaluated 171 Sodium Colistimethate prescriptions between 2015 and 2018. The majority of patients were male (n=114; 67%) and mean age in years was 61 ± 16. Only 17 patients (10%) had chronic kidney disease previous of Sodium Colistimethate use. Sodium Colistimethate was prescribed as targeted therapy in 131 patients (77%), and *Pseudomonas aeruginosa* was the most frequent targeted identified microorganism (n=96; 73%). Data regarding loading dose was available from 140 patients. From these, 79 (56%) did not receive any loading dose and 5 (8%) received an adequate loading dose. Regarding maintenance doses, only 74 patients (51%) received adequate dose when adjusted to creatinine clearance. Ninety per cent of inadequate maintenance doses were subtherapeutic.

**Conclusion:** In our cohort we found that the majority of patients did not receive a loading dose and only half received an adequate maintenance dose. It proves critical that more efforts are put in implementing strategies that can educate and reinforce the need for an adequate dosage prescription.

**References**

1 - European Medicines Agency completes review of polymyxin-based medicines EMA/785229/2014 (Annex III)

**Disclosure of Interest**: None declared

### P360 AN EVALUATION OF ANTIFUNGAL USE IN INTENSIVE CARE: OPTIMISING STEWARDSHIP IN GREATER GLASGOW & CLYDE

#### L. Cottom, B. Jones

##### Department of Medical Microbiology, Glasgow Royal Infirmary, Glasgow, United Kingdom

###### **Correspondence:** L. Cottom

**Introduction:** Invasive candidiasis in critically ill patients has continued to challenge, with infection being associated with significant morbidity and mortality.

The criteria for initiating empirical therapy for suspected invasive candidiasis in non-neutropenic high-risk patients remains poorly defined. Early initiation and the widespread use of antifungal agents must always be balanced against the risk of toxicity and emergence of resistance.

Over the last decade there has been a growing awareness that improvements to stewardship and robust surveillance is needed to ensure the judicious use of antifungal agents.

This study supports the importance of our Microbiology led ICU stewardship programme in promoting effective antifungal usage.

**Objectives:** The aim of this study was to evaluate antifungal use in intensive care and assess the impact of our stewardship programme.

**Methods:** A retrospective analysis was performed over a 12 month period (July 2017 to August 2018). Data on antifungal usage was provided by the lead Pharmacist for our intensive care unit at Glasgow Royal Infirmary, a large teaching hospital within Greater Glasgow and Clyde.

Only patients who were identified as being initiated on antifungal therapy were included for anaylsis. The indication/criteria, diagnosis, antifungal management and clinical advice was reviewed for each patient. For each case the microbiological culture results were assessed. The 30-day, 90-day and all-cause mortality rate was determined for the study population.

**Results:** A total of 52 patients were identified as receiving antifungal therapy. In 83% (43/52) of cases, empirical antifungal therapy was considered appropriate based on clinical or radiological evidence. Of these, 53% (23/43) of patients received targeted antifungal therapy based on positive culture results (a *Candida spp* cultured from a sterile site specimen in keeping with invasive candidiasis). For the 7 cases where antifungal therapy been initiated either prior to admission to ICU or review/discussion on our stewardship ward round, following a detailed assessment antifungal therapy was stopped with no adverse outcome (culture negative, no clinical or radiological evidence, all cause mortality was 0%).

**Conclusion:** This study supports the importance of an effective programme of surveillance and audit to promote antifungal stewardship and improve clinical outcome.

**Disclosure of Interest**: None declared

### P361 TRENDS IN NOSOCOMIAL PATHOGEN RATES BY ANTIBIOTIC PHENOTYPE AND PHENOTYPE-SPECIFIC ANTIBIOTIC PRESSURE METRICS

#### M. Jones, V. Stevens, J. Lewis, A. Bostwick, M. Samore, M. Rubin

##### Epidemiology, VA SALT LAKE CITY HCS, Salt Lake City, United States

###### **Correspondence:** M. Jones

**Introduction:** In antimicrobial stewardship, progress on one front is often met by set-backs in others. We investigated whether some pathogens trends are worsening in the US Veterans Affairs (VA) system, as well as whether antibiotic trends portend to problems in the future.

**Objectives:** Estimate trends for several pathogens by prevalent antibiotic phenotypes. Estimate antibiotic phenotype-specific trends in antibiotic pressure.

**Methods:** Data were extracted between 2005 and 2016 from VA medical centers. The top 5 most prevalent antibiotic phenotypes were studied. Nosocomial trends were assessed by counting clinical isolates occurring after hospital day 2 without precedent in the past year, denominated by patient days-at-risk. Antibiotic regimens selecting for a pathogen were those with no predicted activity given the phenotype. Regimens selecting against a phenotype contained at least one active agent. Antibiotic days for and against phenotypes per 1000 days present were measured. Annual trends were estimated using generalized estimating equations, assuming a negative binomial distribution and allowing for clustering at medical centers.

**Results:** Nosocomial trends varied widely, even within pathogen groups. The highest increasing trend was *Escherichia coli* resistant to fluoroquinolones (FQR; 2.1% annual). The steepest declining trend was an FQR *Enterococcus faecium* (-19%)*.* Antibiotic pressure selecting for phenotypes declined almost universally, excepting *Staphylococcus aureus* phenotypes. Trends in antibiotic selection against phenotypes were mixed, increasing consistently only against *Pseudomonal* phenotypes.

**Conclusion:** Different phenotypes can have strong divergent trends, even within pathogens. General decreases in selection pressure for pathogens may be due to a combination of decreased total antibiotic use and increasing breadth of coverage; we have seen evidence for the former (1). Variable trends in antibiotic selection against pathogens argues against universal increasing breadth of coverage.

**References**

1. Kelly AA, et al. A Report of the Efforts of the Veterans Health Administration National Antimicrobial Stewardship Initiative. Infect Control Hosp Epidemiol. 2017 May;38(5):513-520.

**Disclosure of Interest**: None declared

### P362 BINARY DRUGS ARE A PROMISING DIRECTION FOR OPTIMIZING THE TREATMENT OF INFECTIOUS DISEASES, INCLUDING HAIS

#### A. V. Tutelyan^1^, Y. A. Nikolaev^2^, G. I. El-Registan^2^

##### ^1^Rospotrebnadzor, Central Researche Institute of epidemiology; ^2^Russian Academy of Sciences, FIC Fundamental Principles of Biotechnology, Moscow, Russian Federation

###### **Correspondence:** A. V. Tutelyan

**Introduction:** Research on the development of antimicrobial agents that are effective against circulating strains of the main causative agents of HAIs, is developing in several directions, including the search for new antibiotics and the creation of combined preparations from already known compounds. In the last decade, a trend has emerged to search for substances that enhance the action of antibiotics, among a wide range of substances that do not have their own antibacterial activity

**Objectives:** To study the in vitro effect of alkylresorcinol (AR), approved for use in medical practice as adjuvants that enhance the effect of antibiotics.

**Methods:** Test objects non-pathogenic strains of *Escherichia coli*, *Staphylococcus aureus, Candida utilis*; studied drugs: various types of antibiotics, including aminoglycosides, polypeptides, beta-lactams, quinolones, macrolides, phenol derivatives; AR - 4-hexylresorcinol (GR). GR was used at concentrations of ½ the minimum inhibitory concentration (MIC), which was determined individually for each test object. Antimicrobial activity was judged by a decrease in MIC for a single antibiotic or in combination with GR, in which there is no growth of the test organism on liquid media for 24 hours

**Results:** In the presence of ½ MIC GR for *E. coli*, there is an increase in the antimicrobial action of all antibiotics without exception — different types of action and different structure. The effect of polymyxin, azithromycin and levomycetin is most strongly enhanced (MIC decreases 10-50 times), and at least for cyclosporine, vancomycin, rubomycin (2-3 times). In the presence of ½ MIC GR for *S.aureus*, an increase in the antimicrobial action of all the tested antibiotics is also observed. The effect of polymyxin, capremabol, ampicillin increases to the greatest extent (5-10 times). Two antibiotics, ciprofloxacin and naftifine hydrochloride, were tested against fungi. Addition of GR reduced the MIC for both antibiotics 4 times

**Conclusion:** The use of antibiotics of different groups and mechanisms of action in conjunction with microbial auto-regulators of growth, in particular hexylresorcin, allows to reduce the concentration of the antibiotic used.

**Disclosure of Interest**: None declared

### P363 SYSTEMATIC REVIEW OF THE EFFECT OF BACTERIOPHAGE TREATMENT IN HUMANS

#### N. Saperkin

##### Epidemiology, UNIVERSITY OF UTRECHT, Utrecht, Netherlands

**Introduction:** Lytic virulent bacteriophages (specific viruses) are considered to be a promising intervention for the treatment of infections, particularly antibiotic-resistant ones. For successful implementation of this approach, convincing evidence of safety and efficacy is needed.

**Objectives: Objective**: to assess the effect of bacteriophage therapy for the prevention or treatment of bacterial infections in humans.

**Methods: Search methods** We searched MEDLINE (1935- 2018), the CENTRAL (1999-2018), Embase (1935-2018), and Russian-language literature databases e.Library and rsl.ru (1980-2018).

**Selection criteria** We included randomized controlled trials (RCTs) investigating the effects of phage therapy in people with bacterial infections.

**Data collection and analysis** Two review authors independently selected studies, extracted data, and assessed risk of bias. We used random-effects models for meta-analysis. If meta-analysis was not possible, we summarized the results narratively.

The study protocol was recorded in PROSPERO under the registration number CRD42018100813.

**Results: Results** For the evaluation of the frequency of phage-associated adverse events, we summarized the results of six studies that reported this outcome, five countries. For wound infection and surgical site infection, polyvalent phages seemed to be clinically safe when compared with antibiotics. Fewer adverse events were reported in the PhagoBurn trial for the anti-Pseudomonas aeruginosa phage cocktail versus argentic antimicrobial, with relative risk of 0.4 (95% CI 0.1 to 1.3). Similarly, no significant difference was found by Budanov et al between the polyvalent phage and antibiotics, RR=0.3 (95% CI 0.01 to 5.8). In Wright’s study more adverse events were revealed in individuals suffering from chronic otitis and treated with another anti-P. aeruginosa polyvalent bacteriophage, RR=1.2 (95% CI 0.5 to 2.9). The pediatric study of Sarker reported no adverse events attributable to phages vs placebo. Overall, meta-analysis provided us with a pooled estimate of 0.74 (95% CI 0.68 to 1.2).

**Conclusion: Conclusion** Phage-treated individuals reported fewer or not exceeding amount of adverse events compared with placebo or antibiotics. However, the conduct of well-designed and sufficiently powered RCTs would facilitate registration issues and wide accepting of bacteriophage therapy.

**Disclosure of Interest**: None declared

## Poster session: Hand hygiene for the surgeon

### P364 SYSTEMATIC REVIEW ON MICROBIOLOGICAL EFFICACY OF WHO ALCOHOL-BASED HAND RUB FORMULATIONS FOR SURGICAL HAND PREPARATION

#### H. Saito^1,2,3^, M. Abbas^4^, S. Mai^2^, A. J. Emecheta^5^, S. Tomczyk^2,3,6^, D. Pires^4^, H. Soule^4^, B. Allegranzi^2^, D. Pittet^4^

##### ^1^St. Marianna University School of Medicine, Yokohama City Seibu Hospital, Yokohama, Japan; ^2^Infection Prevention and Control Global Unit, WHO; ^3^University of Geneva, Institute of Global Health; ^4^Infection Control Programme, Geneva University Hospitals, Geneva, Switzerland; ^5^University of Iowa, Iowa City, Iowa, United States; ^6^Robert Koch Institute, Berlin, Germany

###### **Correspondence:** H. Saito

**Introduction:** Effective surgical hand preparation (SHP) is required prior to surgical procedures. World Health Organization (WHO) recommended two formulations for local production of alcohol-based hand rub (ABHR), composed of alcohol, glycerol, and hydrogen peroxide. However, recent evidence suggests that WHO formulations may not comply with the efficacy requirements of international norms (eg. EN 12791).

**Objectives:** The objective of this systematic review was to assess the efficacy of WHO ABHR formulations and/or any modified version of their composition to international norms for SHP.

**Methods:** The following databases were searched: MEDLINE, EMBASE, CINAHL, Cochrane Central Register of Controlled Trials, and Google Scholar. Title-abstract screening, full-text screening, and data extraction were conducted by at least two reviewers. The review was registered to PROSPERO (CRD42018112697).

**Results:** The search yielded 2909 studies for title-abstract screening of which 161 studies were reviewed in full-text. Five studies were identified for the final data extraction. The evidence suggests that neither WHO formulations met noninferiority of the norms, and that increased alcohol concentration by using mass rather than volume percent concentrations and reducing glycerol (either halving from 1.45% to 0.725% vol/vol or eliminating completely) improved both immediate and sustained microbiological efficacy. Generally, 5-minute rub showed better efficacy than 3-minute. None of the included studies provided data on tolerability and acceptability of the modified formulations. Four of the five studies were performed by the same research group.

**Conclusion:** Available evidence suggests that the microbiological efficacy of the WHO ABHR formulations is not fully compliant with international norms for SHP. However, the clinical implication of these findings is unclear. Some studies indicate that modified formulations could improve compliance with the norms but their tolerability is unclear. Thus, more research is needed to identify more effective and well-tolerated ABHR formulations for local production.

**Disclosure of Interest**: None declared

### P365 COMPARING SURGEONS’ SKIN TOLERANCE AND ACCEPTANCE TO ALCOHOL-BASED SURGICAL HAND PREPARATION VERSUS TRADITIONAL SURGICAL SCRUB: A MATCHED CLINICAL TRIAL

#### A. E. R. Lopes, S. R. M. D. S. Canini, M. G. Menegueti, G. G. Gaspar, R. O. C. Santos, L. R. Ferreira, M. E. L. D. V. Dallora, F. Bellissimo-Rodrigues

##### University of São Paulo, Ribeirão Preto, Brazil

###### **Correspondence:** F. Bellissimo-Rodrigues

**Introduction:** According to the World Health Organization (WHO), handrubbing with alcohol-based handrub (ABHR) and handscrubbing with antimicrobial soap and water are both recommended for surgical hand preparation, as they are considered equally effective for surgical site infection prevention.

**Objectives:** To compare the tolerance and acceptance of surgeons to handrubbing with ABHR and handscrubbing with antimicrobial soap and water.

**Methods:** A matched clinical trial was conducted in a university hospital among cardiac and orthopedics surgical teams, from April 1 to October, 31, 2017. In the first phase, participants performed handscrubbing with either 2% chlorhexidine (CHG) or 10% iodopovidone (PVP-I). In the second phase, participants performed handrubbing with an ABHR (57% ethanol + 22.5% n-propanol). Two trained nurses using WHO-validated scales evaluated surgeons’ skin tolerance and acceptance of the study products. Data was dichotomized in “good” or “bad” and analyzed using the MacNemar’s test within the STATA program. Tolerance to the ABHR was separately compared to CHG and PVP-I containing products.

**Results:** We enrolled 56 surgeons potentially eligible, but 23 medical residents did not joined any operation during one of the study phases. Therefore, 33 participants constituted the “per protocol” population. Regarding tolerance, there was little variation in redness, scaliness, fissures, and visual rating of the skin when handrubbing was compared to handscrubbing with PVP-I or CHX. Regarding acceptance, participants rated better handrubbing than handscrubbing with PVP-I regarding product smell (66.6% x 0%, p=0.002), color (73.3% vs 0%, p=0.001) product texture (60% vs 0%, p=0.004), skin dryness effect (60% vs 0%, p=0.004), application (66.6% vs 0%, p=0.002) and general satisfaction (66.6% vs 6.7% p=0.011). Participants rated similarly handrubbing and handscrubbing with CHX, except for product texture, when ABHR was better rated (71,4% vs. 0%, p=0.002). When asked which method they preferred, 73.3% answered handrubbing.

**Conclusion:** Considering surgeons’skin evaluation, both handrubbing and handscrubbing were well tolerated. Regarding acceptance, handrubbing was preferred by most of the surgeons.

**Disclosure of Interest**: None declared

### P366 SURGICAL HAND ANTISEPSIS WITH ALCOHOL-BASED PREPARATION: PERFORMANCE EVALUATION IN A LARGE PHILANTHROPIC HOSPITAL IN SÃO PAULO, BRAZIL.

#### F. C. Castro^1^, J. A. Caraça^1^, G. T. Ferreira^1^, M. S. Almeida^2^, G. H. Myai^2^, T. Rodrigues^3^, J. Y. Kawagoe^4^, M. Vilins^5^

##### ^1^Hospital Epidemiology, Hospital Santa Marcelina; ^2^Medicine school, Faculty Santa Marcelina; ^3^Infection Prevention / commercial, B. Braun; ^4^Professional Master's Degree in Nursing , Albert Einstein Israelite School of Health Sciences; ^5^ Hospital Epidemiology, Hospital Santa Marcelina, SÃO PAULO, Brazil

###### **Correspondence:** J. Y. Kawagoe

**Introduction:** Alcohol-based preparation (ABP) was implemented in July 2018 for surgical hand antisepsis (SHA) because of its rapid action, time savings, fewer side-effects and cost savings.

**Objectives:** To evaluate the adherence to SHA with ABP, according to World Health Organization and manufacturer recommendations.

**Methods:** Cross-sectional study, performed from September 2018 to March 2019, at 700-bed hospital with 17 operating rooms. Any member of surgical team using ABP for SHA was observed by a trained professional for 1h, 3 times a week using a personal electronical device with checklist in Google Forms divided in two phases: PI - Pre-antisepsis and PII - Antisepsis with ABP - executed with fricction in 3 steps: S1 fingertips and forearms, S2 other side’s fingertips and forearms, S3 both hands, back of the hands, interlaced fingers, back of the fingers, thumbs; duration of technique, ABP volume used and hand drying.

**Results:** 127 SHA were observed. PI: absence of jelwelry in the hands and wrists: 124, 97.6%; hand washing and hand drying: 118, 92.9% and 114, 89.7% respectively and use of surgical mask: 125, 98.4%. PII: S1: fingertips 112, 88.1% and forearms 121, 95.2%, S2: other side’s fingertips 124, 97.6% and forearms 118, 92.9%, S3: both hands 118, 92.9%, back of the hands 93, 73.2%; interlaced fingers: 83, 65.3%; back of the fingers: 43, 33.8%; thumbs: 65, 51.1%. The antisepsis time ≥ 60 seconds: 81, 63.7%; minimum volume recommended or more: 108, 85% and hand drying: 92, 71.4%. Total adherence to PI was 94.6% and PII was 17.2% and compliance to the steps 1, 2 and 3 were 87.6%, 92.3% and 67,3% respectively.

**Conclusion:** Lower adherence occurred in step 3 of antisepsis with ABP. Periodic evaluation of the SHA procedure with ABP is crucial in order to raise awareness and to guarantee surgical patient safety.

**References**

WHO Guide to Implementation. A Guide to the Implementation of the WHO Multimodal Hand Hygiene Improvement Strategy. Geneva. 2009. 48 p.

**Disclosure of Interest**: F. Castro: None declared, J. Caraça: None declared, G. Ferreira: None declared, M. Almeida: None declared, G. Myai: None declared, T. Rodrigues Employee of: B.Braun, J. Kawagoe Employee of: Patient Safety Consultant - B Braun, M. Vilins: None declared

### P367 ADHERENCE TO WORLD HEALTH ORGANIZATION RECOMMENDED TECHNIQUE FOR SURGICAL HAND PREPARATION USING ALCOHOL-BASED HANDRUB: A PILOT OBSERVATIONAL STUDY

#### D. Perréard^1^, C. Ginet^1^, G. Agneta^2^, C. Geslin^3^, I. Banckaert^4^, M. Abbas^1^, D. Pittet^1^

##### ^1^Infection Control Programme; ^2^Operating Theatre; ^3^Operations Branch; ^4^Centre for Training and Skills, Geneva University Hospitals, Geneva, Switzerland

###### **Correspondence:** D. Perréard

**Introduction:** Since 2010 our institution recommends performing surgical hand preparation (SHP) using alcohol-based handrub. Washing hands using soap and water is only performed in specific circumstances. Adherence to the recommended technique had never been measured.

**Objectives:** To evaluate adherence to the World Health Organization (WHO) recommended technique for SHP using a standardized checklist.

**Methods:** We performed direct observations of healthcare workers (HCWs) in the operating theatre to evaluate the quality of the technique in terms of number of steps performed out of the total, correct sequence of steps, and recommended duration. A specific data collection tool was custom-made.

**Results:** During a 3 month period, direct observations were performed by 2 observers on 47 HCWs: theatre nurses and instrument technicians (n=14), and surgeons (n=33). Theatre nurses and instrument technicians performed a mean of 12.8 out of 17 steps (standard deviation [SD] 1.5), on average, whereas surgeons performed a mean of 9.5 out of 17 steps (SD 2.1). Recommended duration of SHP was achieved by 10 nurses and technicians (71%) and 18 surgeons (55%). Among HCWs in whom sequence was observed, 7 (64%) nurses and technicians, and 28 (100%) surgeons performed it incorrectly.

**Conclusion:** There was poor overall adherence to the 3 evaluated components of the WHO recommended technique for SHP. Surgeons’ performance was poorer than that of theatre nurses and technicians. Training and monitoring of SHP is needed to improved practices. Identified needs were a chronometer to time SHP duration and simplification of the illustrated technique, as well as a video tutorial.

**Disclosure of Interest**: None declared

### P368 EVALUATION OF A NEW SURGICAL HAND PREPARATION METHOD USING A NO-TOUCH DISPENSER WITH CONTINUOUS DISPENSING FUNCTION

#### T. Takami^1^, T. Konno^1^, X. Huang^2^, Y. Kumashita^1^, M. Yamamoto^1^, Y. Hirata^1^

##### ^1^Biochemical Laboratory; ^2^Product Development Division, Saraya Co., Ltd., Osaka, Japan

###### **Correspondence:** T. Takami

**Introduction:** According to the surgical hand preparation with alcohol-based handrubs in the *WHO Guidelines on Hand Hygiene in Health Care* (2009), 5 mL of alcohol-based handrubs is taken with one hand and smeared on the other hand up to the elbow. In this procedure, there is a risk that the expected amount of handrubs could not be applied on the entire forearm due to spill of the handrubs. In order to avoid this risk, we have developed a new method that sprays the handrubs directly from the hand to the elbow using a continuous dispensing mode (up to 20ml) of a no-touch dispenser. In addition to the continuous direct application of the handrubs, the new method also focuses on the disinfection of fingertips since this is the most contaminated parts of hands in daily work in healthcare settings.

**Objectives:** To evaluate the effectiveness of a new surgical hand preparation method.

**Methods:** The efficacy test method of surgical hand preparation, EN 12791 was referred to evaluate the efficacy in this study. The volunteers’ hands and forearms were prepared by washing with plain soap, and the resident bacteria on the hands and forearms were sampled to determine the baseline. The hands and forearms were disinfected using the new surgical hand preparation method with a test product (contains ethanol, n-propanol, phosphoric acid and other 3 ingredients). The resident bacteria on one hand and forearm were sampled after disinfection and the number of bacteria was measured to evaluate the disinfection effect.

**Results:** The disinfection effect was observed, and the requirements of EN 12791 were fulfilled.

**Conclusion:** The new method which directly and continuously applies handrubs on hands up to elbows and focuses on the disinfection of fingertips is considered to be effective for surgical hand preparation.

**Disclosure of Interest**: None declared

### P369 SUCCESS IN IMPLANTATION OF SURGICAL HANDS DISINFECTION WITH ALCOHOL SOLUTION: IMPACT ON COST AND ENVIRONMENTAL RESOURCES

#### M. Crema Tobara^1^, C. de Almeida Silva^1^, P. Martins Silva^1^, L. Dias^1^, B. Silva Dalla Dea^1^, V. Moreno Fernandes^1^, G. Pereira Vetuche^1^, T. Teixeira Rodrigues^2^, R. Tamazato^3^, E. Shimura^3^, C. Nagao^3^, R. Richtmann^1^

##### ^1^Infection Control, Grupo Santa Joana; ^2^Infection Control Comercial, B.Braun; ^3^Surgical center, Grupo Santa Joana, São Paulo, Brazil

###### **Correspondence:** M. Crema Tobara

**Introduction:** use of alcohol-based solutions for surgical hands disinfection is justified by its antimicrobial efficacy, ease of application, less damage to the skin and time saving. In Brazil, surgical brushing of the hands with chlorhexidine is still the most widespread technique among surgeons. A well-structured implementation process is critical to ensuring culture change and expected benefits.

**Objectives:** To describe the steps of implantation process of surgical hands disinfection with alcohol-based solution (Softalind® Pure, B.Braun), to compare the use of the alcohol-based solution versus scrubbing with chlorhexidine brushes. Environmental impact and cost-effectiveness were compared.

**Methods:** Retrospective, descriptive study performed in two private maternity hospitals in São Paulo, Brazil. Compared the period of use of scrub with chlorhexidine brushes (P1- January 1, 2013 to December 31, 2015) with the post-implantation period of the alcohol-based product (P2- January 1, 2016 to December 31, 2018), related to the amount of waste generated, water consumption and costs.

**Results:** The implementation process developed in three stages: evaluation and acceptance of the new product; training and validation of the surgical team and follow-up. P1 period (scrub): 102,776 surgical procedures. The amount of residue with brushes used 4.1 tons; the consumption of water for hand rinsing was 791,432 liters and brush costs US $ 127,000. P2 period (alcohol-based solution): 98,050 surgical procedures. The cost of the alcoholic product was US $ 67,783.80. The total cost of the technique with the alcohol-based product was 46% lower compared to chlorhexidine brushing, generating savings in the period of US $ 59,500. There was a reduction in water consumption of 686,350 liters and a reduction of 3.9 tons of waste in the period studied after the implementation.

**Conclusion:** The well-planned implementation of this process proved to be effective. The use of the alcoholic product proved to be more economical, also generating great impact in relation to environmental resources.

**Disclosure of Interest**: M. Crema Tobara: None declared, C. de Almeida Silva: None declared, P. Martins Silva: None declared, L. Dias: None declared, B. Silva Dalla Dea: None declared, V. Moreno Fernandes: None declared, G. Pereira Vetuche: None declared, T. Teixeira Rodrigues Employee of: B.braun, R. Tamazato: None declared, E. Shimura: None declared, C. Nagao: None declared, R. Richtmann: None declared

### P370 COMPLIANCE WITH DRESSING CHANGE IN PRE-POST INTERVENTION COMPARISON AFTER INTRODUCTION OF A NEW DRESSING CHANGE CONCEPT IN THE CONTEXT OF A PROSPECTIVE COHORT STUDY ON INFECTION PREVENTION

#### M. M. Strybos^1^, R. Otchwemah^1^, S. Akca^2^, C. Kugler^2^, R. Galante^1^, I.-K. Dombrowski^1^, J. Hoffmann^1^, M. Frauke^1^

##### ^1^Institute for Hygiene, Kliniken der Stadt Köln gGmbH, Cologne; ^2^Institute of Nursing Science, University of Freiburg, Freiburg, Germany

###### **Correspondence:** M. M. Strybos

**Introduction:**

The prospective cohort study funded by the Federal Ministry of Health (project "HygArzt" ZMVI1-2516FSB111) aims to demonstrate the effectiveness of hygiene measures introduced by an in ICP trained physician of the department.

**Objectives:**

We investigated the introduction of a new dressing change (DC) concept (DCC), and general hand hygiene compliance (HHC) according to WHO in three orthopedic/trauma surgery normal wards.

**Methods:**

The DC HHC were recorded with a modified checklist based on the recommendations of the German Commission for Hospital Hygiene and Infection Prevention. Four required hand disinfections were defined as indications ("preparation of DC", "immediately before DC", "before clean phase", "after DC"). In the pre-intervention phase (pre) 337 DC were observed to assess the initial situation. Based on the results, a new DCC was introduced in the intervention phase (int). This included the introduction of additional bed dispensers for hand disinfectants at each patient's bed, taking the dressing trolley into the patient's room to ensure a low-germ storage area, introduction of sterile disposable trays for the transport of dressing material into isolation rooms (into which the dressing trolley may not be taken), theoretical and practical DC-training on and off the job. During the intervention phase, 60 observations were performed and the DCC adapted iteratively. In the post-intervention phase (post) 317 DC have been observed so far. A χ²-test was performed for the analysis.

**Results:**

The introduction of the DCC has significantly increased the HHC of all indications. The strongest increase in HHC occurred in the indication "preparation of DC" (34% pre, 91% int to 85% post; *p*<.001). The HHC also increased in the other indications ("directly before DC"; 32% pre, 73% int, 85% post; *p*<.001); "before clean phase" (42% pre, 81% int, 96% post; *p*<.001); "after DC" (74% pre, 100% int, 99% post; *p*<.001); "after DC" (74% pre, 100% int, 99% post; *p*<.001).

**Conclusion:**

The introduction of the new DCC has significantly increased HHC in all indications of the DC. Through continuous observation and an iterative improvement process, the intervention could already be adapted in the int.

**Disclosure of Interest**: None declared

## Poster session: Hand hygiene: perception and knowledge

### P371 STUDYING MISINFORMATION AND FAKE NEWS IN HAND HYGIENE: A SYSTEMATIC APPROACH

#### A. Peters^1^, C. Guitart^1^, N. Lotfinejad^2^, D. Pittet^1^

##### ^1^University Hospitals of Geneva, Geneva, Switzerland; ^2^Mashhad University of Medical Sciences, Mashhad, Iran, Islamic Republic Of

###### **Correspondence:** A. Peters

**Introduction:** Hand hygiene (HH) is a simple and cost-effective measure to reduce the global burden of healthcare-associated infections (HAI) and slow the spread of antimicrobial resistance. Hand hygiene using alcohol-based hand rub/alcohol sanitizer (ABHR) saves millions of lives each year. Numerous studies, including meta-analyses, have demonstrated the safety, microbiological efficacy and clinical effectiveness of hand hygiene in healthcare settings. With so much clinical evidence to support its use, one would imagine that the field of HH is relatively protected from fake news and misinformation. In the contrary, misinformation about the safety and efficacy of ABHR is widespread and can decrease compliance and put patients directly at an increased risk for HAI.

**Objectives:** To identify the instances and trends of misinformation in the field of HH, especially when the safety or efficacy of ABHR was questioned without scientific evidence to support it (as this would be the type of misinformation that would have the greatest effect of patient safety).

**Methods:** Because of our work in the field, we were already aware of numerous instances of misinformation concerning ABHR. We performed a literature search to identify some of the misinformation that could potentially damage healcare worker compliance. Some of the search questions were “dangers of hand sanitizer”, “can hand sanitizer give me cancer” and “reasons to avoid hand sanitizer”

**Results:** A precursory search revealed that there are probably thousands of articles (mostly in the lay press) that misinform the public about ABHRs. These include the ideas that certain bacteria are developing resistance to ABHR, and that most ABHRs can put you at risk for bisphenol-A and triclosan absorption, that ABHR can be toxic to use, or carcinogenic, etc. The effect of this misinformation has not yet been quantified. Based on our research, we will propose a framework to assess the risk for the fake news to spread in HH and link our work to the “European fake news initiative”.

**Conclusion:** More must be done to combat fake news and misinformation in HH, as the outcomes of a reduced healthcare worker compliance translates directly into an increase in HAIs and jeopardizes patient safety.

**Disclosure of Interest**: None declared

### P372 EVALUATING EMOJIS AS CANDIDATES FOR EXPRESSING HAND HYGIENE BEHAVIOUR; PRELIMINARY RESULTS

#### N. Lotfinejad^1^, A. Peters^2^, E. Tartari^2^, M. De Kraker^2^, R. Assadi^1^, D. Pittet^2^

##### ^1^Mashhad university of medical sciences, Mashhad, Iran, Islamic Republic Of, ^2^University hospital of Geneva, Geneva, Switzerland

###### **Correspondence:** N. Lotfinejad

**Introduction:** Hand hygiene (HH) is universally recognized as the cornerstone of healthcare-associated infection (HAI) prevention. Although the WHO “My five moments for hand hygiene” has been used for almost a decade to delineate HH indications and promote action, adherence levels among healthcare workers (HCWs) are still notoriously low and disquieting.

**Objectives:** To compensate for the lack of effective HH communication, we aimed to describe a novel educational strategy by evaluating emojis as possible surrogates for the non-verbal aspects of HH behaviour.

**Methods:** Following a thorough review of the Unicode version 12.0, the most applicable emojis to the terms used in the 5 Moments were extracted. Consequently, we developed a questionnaire to determine infection prevention and control (IPC) experts’ opinions regarding the proposed emojis. Completed questionnaires were collected and analysed to assess the suitability of the existing emojis to illustrate a unified emoji poster of the 5 Moments. IPC experts were not informed that other experts would be asked to complete the questionnaire.

**Results:** According to our preliminary results, 8 emojis demonstrated a scientific appropriateness of 50% or more. There was a 62.5% agreement on five emojis related to “hand”, “hygiene”, “touching”, “risk”, and “surroundings” and 75% agreement on “patient”. Further results of this study will be presented at the conference.

**Conclusion:** Emojis have the potential for promoting HH compliance provided that they clarify information through adding dynamicity to verbal cues that may lead to a more accurate perception of the intentions of the HH process. Using these symbols may be one major step forward in conveying HH concepts. Even though the observations of this study revealed that existing emojis are not completely adequate to express HH moments, using meticulously selected emojis to promote the 5 Moments for HH may result in enhanced attention, memory and behaviour change. Further research and implementation testing is needed.

**Disclosure of Interest**: None declared

### P373 IMPACT OF SOCIAL MEDIA IN DISSEMINATING HAND WASHING INFORMATION TO UNIVERSITY PURE AND SOCIAL SCIENCE STUDENTS

#### H. M. Ibrahim^1^, I. Yusuf^2^

##### ^1^ICT Unit, Hydraulic Equipment Development Institute; ^2^Department of Microbiology, Bayero University Kano, Kano, Nigeria

###### **Correspondence:** H. M. Ibrahim

**Introduction:** Acquisition and dissemination of community and hospital acquired multi drug resistant bacteria (MDRB) remained a public health issue in Nigerian health care delivery systems. Floors of toilets in public schools in Nigeria could be heavily contaminated with all sorts of germs, because they are often characterized by lack of constant water for flushing and hand sanitizers. Possession of smart phones by university students is almost 100%.

**Objectives:** A study was therefore undertaken to determine how social media applications could improve hand washing compliance after use of public toilet between pure science (PS) and social science (SS) students of Nigerian University.

**Methods:** A total of 100 male students, 50 each from PS and SS students with each group sharing same toilets. A chat group for each set of student was opened and information regarding importance of hand wash with water, soap and alcohol based hand rubs was passed to each group on daily basis. Pre and post knowledge and practice was asses via online questionnaire. Voice messages, info graphics and short essays on need to employ hand wash after toilet use was transmitted and discussions was monitored. Nature of response which include questions and clarifications and exhaustion of hand washing soaps and sanitizers placed at a distance from the toilet was used to assess the compliance level.

**Results:** Prior knowledge of MDRB was very low (11%) among SS students when compared with PS students (56%). There is no significant different in the practice of hand wash after toilet use with water and soap among the two groups, but use of hand sanitizer was 13% and 28% among SC and PC students respectively prior to the study. Surprisingly, information passed via smartphone improves the knowledge and practice of PS and SS students by 26% and 42% respectively. Only 12% of the SS were aware of MDRB as problem, but 26% agreed that MDRB could be contacted in dirty toilet but not sure of dissemination. Voice messages recorded high impact on SS, followed by info graphics. A shift in practice of hand sanitizer was observed from initial from 13% to 38% among SS and 28% to 66% among PS.

**Conclusion:** Hand wash campaign via smart phone and social media platforms significantly improves hand wash knowledge, practice and overall compliance among social and pure students of a Nigerian University.

**Disclosure of Interest**: None declared

### P374 DEVELOPMENT OF A QUESTIONNAIRE TO ASSESS THE KNOWLEDGE OF PARTICIPANTS BEFORE AND AFTER A TRAIN THE TRAINERS (TTT) COURSE IN HAND HYGIENE (HH)

#### C. Fankhauser, E. Tartari, F. Timurkaynak, D. Pires, A. Peters, F. Bellissimo-Rodrigues, D. Pittet

##### Infection Control Program, HUG, Geneva, Switzerland

###### **Correspondence:** C. Fankhauser

**Introduction:** A train-the-trainers (TTT) 3-day course is used for education of infection prevention and control (IPC) experts worldwide on improvement of hand hygiene practices. A pre- and-post course questionnaire was designed to measure the acquisition of knowledge during the course.

**Objectives:** To develop and field-test a questionnaire on knowledge about HH practices among IPC experts worldwide.

**Methods:** The questionnaire was developed in English. It contained 22 multiple choice questions based on general IPC principles, the WHO Multimodal HH Improvement Strategy, and monitoring HH compliance using the “My five moments for HH”. It was developed in consultation with HH experts. 27 IPC experts were invited to pretest the TTT questionnaire. For content validity, participants were asked to complete a feedback survey. For reliability testing, participants completed a test-retest with at least one week interval between the tests. Following expert validation, field testing of the questionnaire was performed at TTT courses organized in 2 different countries involving 29 and 85 participants, respectively, mostly nurses and doctors.

**Results:** 20 IPC experts from 13 countries (Netherlands, France, UK, Malta, Turkey, Malaysia, Australia, South Africa, Brazil, Costa Rica, Mexico, Canada and Switzerland) and 18 healthcare facilities participated in both test-retest of the questionnaire and completed the feedback survey. Experts advised on the construct and content that resulted in some modifications, such as removed, reworded, or added questions. Reliability testing showed good internal consistency (Kappa coefficient = 0.81). Field testing with 114 TTT participants was performed. The before and after test mean (CI 95%±) were 43.10% (±8.83) and 67.87%(±7.74), respectively, for an increase of 24.7%.

**Conclusion:** The tested questionnaire proved reliable and field testing demonstrated a significant increase in the knowledge of the participants about hand hygiene practices and promotion.

**Disclosure of Interest**: None declared

### P375 HAND HYGIENE KNOWLEDGE AND PERCEPTIONS AMONG ANESTHESIA PROVIDERS IN PALESTINE

#### A. S. Abutayeh^1^, R. Sajdeya^1^, F. Zaben^2^, M. Zaben^1^

##### ^1^IMET2000-Pal, Ramallah; ^2^IMET2000-Pal, Ramallah, Palestinian, State of

###### **Correspondence:** A. S. Abutayeh

**Introduction:** Hand hygiene is considered the single most important procedure for prevention of hospital acquired infection (HAI) in patients and health care providers. Hand hygiene compliance in anesthesia providers has been poorly studied in Palestine. In the absence of standard protocols for hand hygiene in the Palestinian health care system, compliance may be poor.

**Objectives:** To evaluate compliance of anesthesiologists with hand hygiene practice inside the operating room(OR).

**Methods:** A multi-centre, cross-sectional, descriptive study, using a self-administered questionnaire, was conducted in January-March 2015. Participants’ compliance regarding IC practices and availability of training material and programs policies were examined using 48 items questionnaire. SPSS was used for data analysis.

**Results:** Fifty-seven anesthesia doctors from nine governmental and private hospitals in West Bank responded to our survey. Most participants were male (93%) of them 66.7% were residents, and 29.8% were specialists. 61.4% had a postgraduate degree (master, diploma, PhD). Only one third of the respondents begin their day at the OR with hand washing while only half of them always wash their hands between cases. 36.4% reported that they rarely wash their hands before inducing general anesthesia, one third of them specialists. 20.4% rarely wash their hands in neuroaxial blocks, 35.1% in peripheral blocks, 41.7% in venous cannulation. Surprisingly, 24% or participants reported the absence of alcohol inside the operating room

**Conclusion:** Anesthesia providers’ adherence to hand hygiene practice and guidelines is extremely low. This exposes patients and healthcare providers to serious nosocomial infections. Further research is required to know why they do not comply the infection control (IC) practices. Hand hygiene improvement programs should be prioritized and addressed to help anesthesiologists to employ safe hand hygiene.

**Disclosure of Interest**: None declared

### P376 HAND HYGIENE KNOWLEDGE AND PRACTICE IN POLISH LONG-TERM CARE FACILITIES

#### G. Puto^1^, A. Różańska^2^, J. Wójkowska-Mach^2^

##### ^1^Jagiellonian Medical College; ^2^Jagiellonian University Medical College, Kraków, Poland

###### **Correspondence:** A. Różańska

**Introduction:** Healthcare associated infections (HAI) are an important problem not only in hospitals, but also in long-term care facilities (LTCFs). Infection control in LTCF facilities operates in a different framework than in hospitals - infection prevention procedures probably are not as comprehensive as in hospitals.

**Objectives:** The aim of this study was to acquire knowledge about LTCF medical staff knowledge on WHO recommendations on hand hygiene (HH) , their self-assessment of compliance of the practice with recommendations in the context of the infection control program operating in these facilities.

**Methods:** The study was conducted using the diagnostic survey method, which included questions about the WHO recommendations on hand hygiene, self-evaluation of its practice and questions about the scope of infection control programs. The survey was conducted among 237 medical or social workers, from May to June 2017 in LTCFs in the Małopolskie voivodship.

**Results:** Most of the respondents were women (96.2%), of which more than half worked in the profession for at least 20 years. The majority (90.3%) of respondents declared knowledge of the WHO recommendations on hand hygiene, but only 66.7% of them correctly mentioned the moment before contact the patient and 66.2% after contact the patient. The declaration of the WHO recommendations on 5 moments of HH correlated with the declaration that the supervisor ever checked whether an employee complied with these recommendations, and whether he was correctly applying the Ayliffe technique. 81.1% of the respondents declared that the infection control program was implemented in their unit, and 88.5% that a person was appointed to perform infection control tasks. Almost 70% of respondents declared that HH is the most important hygiene procedure in the context of infection prevention. At the same time, also 70% of respondents said that employees who have contact with patients often overlook the HH, although only 27% positive answers to the question whether they face difficulties related to the HH procedure (lack of funds, etc.) were noticed. In the self-assessment, only 15% of respondents declared that they often overlook hand hygiene.

**Conclusion:** The obtained results indicate that the degree of compliance of HH recommendations with practice in Polish LTCF can be highly unsatisfactory and requires immediate implementation of corrective actions.

**Disclosure of Interest**: None declared

### P377 EFFICACY OF IOT (INTERNET OF THINGS) TO IMPROVE HAND HYGIENE PERFORMANCE

#### H. Shigemi^1^, Y. Ideno^2^, Y. Muroi^3^, C. Matsuyama^3^, M. Hida^3^, Y. Yamashita^4^, T. Ishizuka^1^, H. Iwasaki^3^

##### ^1^Respiratory Medicine, UNIVERSITY OF FUKUI, Fukui; ^2^Global Business, Carecom co. Ltd, Tokyo; ^3^Infection Control and Preventon, ^4^Medical Information, UNIVERSITY OF FUKUI, Fukui, Japan

###### **Correspondence:** H. Shigemi

**Introduction:** Inappropriate usage of antimicrobiotics has easily induced antimicrobial resistance. To decrease the rate of MRSA, it is essential not only to practice the proper usage of antimicrobiotics but also to educate hand hygiene performance (HHP). In 2017, enforcement rate of HHP was low (37%) in Japan. Otherwise, it is reported that HHP are 36%~51% in the US, 33%~65% in Europe.

**Objectives:** Applying through IoT, we analyzed big data to visualize the status of HHP. Compared the data obtained using new IoT and AI methodology, with the data by the direct / indirect observation, we evaluated the effectiveness of our method.

**Methods:** In July 2018, 21 doctors and 40 nurses in gastrointestinal surgery ward(GSW), were subjects of this study. At entering into a patient’s room / patients zone and exit from there, HHP by using disinfection and /or liquid soap were analyzed from the data collected by IoT system. Installed IoT gates on the ceiling can collect the data from each sensor on the pump of disinfect. Mobile phones and mobile disinfect bottle which belong to medical staff, send the wireless signal to IoT gates and enable to integrate the data to show the location. The data of the individual, time course, location, the kind of disinfect, the amount of disinfect were accumulated for 24 hours in the particular area.

**Results:** The average rate of HHP was very low (7.8 %) in doctors and rather high (27.2 %) in nurses. In 5 Moments presented from WHO, the average rate was 8.3 % (Step 1), 2.9 % (Step 2), 21.6 % (Step 3&4), and 10.8 % (Step 5). In our facility, the disinfect volume ( mL / patients・day) was 83.7 in NICU, 17.5 in ICU, 10.1 in hematological ward, and 7.4 in GSW. HHP rate in ICU was 40.1% in doctors and 96.1% in nurses, at entering ICU room.

**Conclusion:** Using IoT, compliance of HHP can be analyzed accurately for 24 hours continuously. The system could collect the data concerned with HHP in real time and all of the medical staff simultaneously. In GSW, it would be necessary to educate medical staff for recognizing the importance of HHP. Applying feedback system will enable us to proceed with HHP. (Supported by Strategic Information and Communications R&D Promotion Programme (SCOPE) Grant from Ministry of Internal Affairs and Communications 181605001)

**Disclosure of Interest**: None declared

### P378 UNINSTRUCTED HANDWASHING BY KINDERGARTEN CHILDREN - THE SIGNIFICANCE OF THE THUMB AND INDEX FINGERTIP

#### P. Pfister

##### GP PRACTICE, Regensdorf, Switzerland

**Introduction:** During the evolution of man from our earliest ancestor *Ardi (Ardipithecus ramidus)* to our own spezies *Homo sapiens* hand skills have developed and become refined (1.). The hand and brain of the modern man interact in a way that allows handling of fine tools and advanced technical equipment like smart phones. The thumb and index finger have become more important and widely used. By their usage they come into contact with microorganisms (2.).

**Objectives:** The aim of this observational study is to unterstand how kindergarten children wash their hands in a natural way without instruction. Of particular interest is whether the thumb and index fingertip get the attention they deserve.

**Methods:** Parents of kindergarten children were asked to film their children with a smartphone while the children wash their hands at home. The children did not get any special instruction in handwashing techniques beforehand. The children knew, that they were being filmed. The first 20 records received (from 12 March to 3 April 2019) were analyzed for this presentation.

**Results:** 20 children aged between 5 years 2 months and 6 years 9 months participated in the study. Only one child washed the index fingertip in a reasonable way. The other children did not rub the index fingertip against another hand part in order to wash the fingertip. The claw hand, which is necessary for such an action, was not formed naturally when washing hands. The thumb was washed well by 2 children and in a rudimentary manner by another 5 children. 13 out of the 20 children did not wash their thumbs.

**Conclusion:** When handwashing children do not give the attention to the thumb and index fingertip which they deserve in view of the wide usage of those hand parts. These findings may explain, why the thumb and fingertips belong to the often missed hand parts during handwashing (3.). The reason for this handwashing behavior probably lies in the anatomy of the hand and in the hand/brain interaction.To wash the thumb and index fingertips properly we need more concentration on these parts. It does not work automatically, but requires concentrated brain energy until it becomes habitual.

**References**

1. Lundborg Göran; The Hand and the Brain, Springer Verlag London 2014

2. McGinley et al.; Composition and density of microflora in the subungal space of the hand; 1988 J. Clin. Microbiol.26:950-3

3. Taylor L. J.; An evaluation of hand washing techniques I+II; Nurs. Times. 1978

**Disclosure of Interest**: None declared

### P379 A JOURNEY TOWARDS IMPROVING PHYSICIANS HAND HYGIENE COMPLIANCE

#### M. L. B. Garcia^1^, C. Simbulan^1^, A. Patel^2^, M. A. El Hassan^2^, M. S. Thomas^3^, E. I. El Magboul^4^

##### ^1^Quality & Patient Safety Department - Infection Prevention & Control; ^2^Cardiology Department; ^3^Nursing Department, Hamad Medical Corporation - Heart Hospital; ^4^Laboratory, Hamad Medical Corporation, Doha, Qatar

###### **Correspondence:** M. L. B. Garcia

**Introduction:** A low hand hygiene compliance of physicians was observed in Coronary Intensive Care Unit (CICU) and remained a challenge for four years. Improvement strategies were initiated and implemented to change culture on physicians’ perspective and to improve compliance.

**Objectives:** To increase hand hygiene compliance of physicians in CICU from 56.5% in 3rd Quarter 2014 to above 90% target by the end of 4th Quarter 2018 and beyond.

**Methods:** A quality improvement “Hand Hygiene Project” was initiated by multidisciplinary team. A driver diagram was developed to implement strategies and to empower staff to remind doctors for any missed opportunities. Trained secret observers were chosen to monitor physicians’ specific compliance based on WHO My 5 Moments of Hand Hygiene and compliance rates were posted monthly by names in CICU Board. Annual recognition was given to physicians with 90% compliance rate.

**Results:** After implementation of initiatives, physicians compliance rate gradually increased but low compliance rates below the 90% target was still observed due to the rotation of new residents. A baseline rate of 56.5% was markedly increased to 86.52% in 4^th^ Quarter 2016 then dropped to 79.19% in 4^th^ Quarter 2017. With continuous reinforcement of practices in 2018, a 90.73% compliance was achieved in 4^th^ Quarter 2018 and a 60.58% increase from baseline rate. A reduction of healthcare associated infections was evident with the significant increase in hand hygiene compliance in CICU.

**Conclusion:** Developing a behavioral culture change from physicians’ perspective was a real challenge and could happen gradually over a period of time. The implementation of interventions and reinforcement of practices will enhance physicians hand hygiene compliance to achieve 90% target. With strong leadership support, cooperation, teamwork, commitment and recognition of physician champions for hand hygiene can make a difference in improving physicians’ hand hygiene compliance.

**References**

WHO (2017). Clean Care is Safer Care. [Online] Available at http://www.who.int/gpsc/5may/background/5moments/en/ [Accessed on 15/02/2017]

Hamad Medical Corporation (2015). CL 7241 Hand Hygiene Policy. [Online] Available at http://intraappsrv01/POLICIES/pdf/CL%207241%20HAND%20HYGIENE%202014.pdf [Accessed on 15/02/2017]

**Disclosure of Interest**: None declared

### P380 HAND HYGIENE WORKSHOP BEFORE STARTING CLINICAL PRACTICE, IS THERE ANY EFFECT ON KNOWLEDGE AND ATTITUDE OF MEDICAL STUDENTS

#### M. H. Aelami^1^, T. Afshari Saleh^2^, H. Zakeri^2,3^, M. Mousavi^3^

##### ^1^Pediatrics& Infection Control and Hand Hygiene Research Center; ^2^MASHHAD UNIVERSITY OF MEDICAL SCIENCES, Mashhad, Iran, Islamic Republic Of; ^3^Emergency Medicine, MASHHAD UNIVERSITY OF MEDICAL SCIENCES, Mashhad, Iran, Islamic Republic Of

###### **Correspondence:** M. H. Aelami

**Introduction:** Hand hygiene is considered the most important measure to reduce the healthcare associated infections.

**Objectives:** The main aim of this study was to evaluate the effect of an educational program among medical students’ knowledge and attitude toward hand hygiene (HH) at Mashhad University of Medical Sciences.

**Methods:** Two groups of medical students of Mashhad university of medical sciences (the first group consisted of the medical students who just passed the physiopathology course and the second group was the students at the beginning of the residency period) were selected to participate in the study during 2017. The Persian version of WHO questionnaire on HH knowledge and attitude was completed by each participant. Then they attended a teaching workshop about the importance and the method of hand hygiene. The same questionnaire was filled by the participants on finishing and 3 months after the workshop. The paired t test was used for analysis of the data.

**Results:** A total of 277 medical students entered in to the study. About 71% completed the study. The knowledge level of the residents was significantly higher than the externs at the beginning of the study (p<0.001). There was a significant difference between the pre-test and the immediate post-test scores on HH knowledge and attitude of the attendants (P<0.001). However, the late post-test scores reduced compare to the immediate post-test scores, but they were still higher than the pre-test scores (P<0.001).

**Conclusion:** Training program can improve the knowledge and attitude toward hand hygiene. Regular course is recommended to maintain the knowledge and attitude levels.

**Disclosure of Interest**: None declared

### P381 THE KNOWLEDGE OF THE PARTICIPANTS OF HAND HYGIENE PHOTO FESTIVAL ABOUT HAND SANITATION IN MASHHAD-IRAN

#### A. Mohajeran^1^, M. Farahi^2^, M. R. Homayouni Fahimi^1^, M. Hooshiar^3^, N. Khosravi ^1^, M. H. Aelami^3^

##### ^1^Infection Control and Hand Hygiene Research Center; ^2^Imam Reza Hospital; ^3^Pediatrics & Infection Control and Hand Hygiene Research Center, MASHHAD UNIVERSITY OF MEDICAL SCIENCES, Mashhad, Iran, Islamic Republic Of

###### **Correspondence:** A. Mohajeran

**Introduction:** Hand hygiene is the most important way to prevent transmissible infections. Increasing Knowledge of people in community about hand sanitation is very crucial. Photo festival is a way to encourage people to think about the role of hand hygiene in their lives.

**Materials and Methods:** During a 6 months period from September 2017 to February 2018 we announced all health care workers in the country and high school students in Khorasan Razavi province to send photos about hand hygiene. Also we asked professional photographers to send photos in this subject. An exhibition of all selected photos was took place in a park in Mashhad, Iran. Questionnaires were filled by some attendants in exhibition area. Questionnaire included demographic information and also questions about hand hygiene products and the duration of hand sanitation.

**Results:** We received 379 photos which of them 20 photos selected and received valuable prizes. In exhibition area 49 people filled the questionnaire. Thirty-two (65. 3%) were female. Seventeen (34.7%) had academic education. Thirty-one (63.3%) preferred soap & water for cleaning hands instead of hand rub. Thirty-three (67.3%) preferred liquid soap instead of solid soap for hand washing. The duration of hand wash and hand rub were correct in 34(69.4%) and 21(42.9%) of responders respectively.

**Conclusion:** Hand hygiene can be promoted by different tools including photo festival. It is very important to increase knowledge of community about hand hygiene.

**Disclosure of Interest**: None declared

### P382 PERCEPTIONS AND BELIEFS IN HAND HYGIENE AMONG NURSES IN TWO HOSPITALS IN KINSHASA

#### N. Muyulu^1,2^, L. Richard^1,3^, R. Borgès Da Silva^4,5^, A. Prud’Homme^4^

##### ^1^Faculté des sciences infirmières, Université de Montréal, Montréal, Canada; ^2^ISSI INSTITUT SUPERIEUR EN SCIENCES INFIRMIERES, Kinshasa, Congo, The Democratic Republic of the; ^3^Institut de recherche en santé publique de l’Université de Montréal (IRSPUM), Université de Montréal; ^4^Institut de recherche en santé publique de l’Université de Montréal (IRSPUM); ^5^Département de gestion, d’évaluation et de politique de santé, École de santé publique, Université de Montréal, Montréal, Canada

###### **Correspondence:** N. Muyulu

**Introduction**

Hand hygiene (HH) is the best way to prevent healthcare-associated infections. The most effective strategies for promoting HH use behavioral changes. Guided by the Precede-Proceed model, the present study aimed to describe the predisposing factors and facilitators of HH among nurses in two hospitals in Kinshasa and to examine associations between these factors and various characteristics of participants.

**Methods**

The quote was of a descriptive correlational type. The convenience sample included 89 nurses who had previously participated in HH training (response rate: 83%). The data were collected by self-administered questionnaire. In addition to participants' characteristics, the questions focused on knowledge, perceptions of HH, and social norms, as well as access to infrastructure that facilitates the adoption of this behavior. Bivariate and multivariate analyzes were performed.

**Results**

The results show lower scores on the scales measuring knowledge and access to infrastructure, compared to the other constructs studied. Significant multivariate associations are obtained in the regression model of perceptions of social norms in hand hygiene. Being in the older age group (50+) and the practice location (hospital and unit) are significant predictors.

**Conclusion**

The findings reveal gaps in HDM knowledge and confirm the importance of the workplace and perceptions of social norms, an important determinant of health behaviors.

**Disclosure of Interest**: None declared

### P383 AN ASSESSMENT OF KNOWLEDGE AND HAND HYGIENE PRACTICES AMONG HEALTHCARE WORKERS AT DODOMA REGIONAL REFERRAL HOSPITAL.

#### E. Venant, E. M. A. Ombaka, V. Ndesendo

##### St.John's University of Tanzania, Dodoma, Tanzania, United Republic of

###### **Correspondence:** E. Venant

**Introduction:** The WHO’s “My 5 moments for hand hygiene” is designed to prevent the spread of infectious organisms in health-care settings which occurs mostly via health-care workers' (HCWs) contaminated hands, items/equipment and the environment. Information on its implementation in Tanzania is needed.

**Objectives:** The main objective of the study was to assess knowledge, access to facilities for hand hygiene and the adherence to the WHO’s five moments of hand hygiene by healthcare workers at Dodoma Regional Referral Hospital. It included an assessment of source of microbial contamination through an examination of bacterial content of cell phones of healthcare workers.

**Methods:** This was a descriptive cross-sectional study. Three sets of data were collected through i) questionnaires to assess knowledge and attitudes and barriers to effective hand hygiene, ii) observation to assess adherence of five moments of hand hygiene, and iii) laboratory examination of samples from cell phones for bacterial contamination. Analysis was performed by using Statistical Package for Social Science (SPSS)*.*

**Results:** Over 75% HCWs had had formal training on hand hygiene and had access to hand washing facilities, but only 63% were aware of the ‘WHO 5 moments’. Doctors, nurses and health care students were observed and of the 270 hand hygiene moments, only 7 (2.6%) complied with expected action. No group was better and no hand hygiene was observed after touching cell phones. Thirty four percent of sampled cell phones were contaminated with *Staphylococci* species which were resistant to penicillin and had varying resistance to Erythromycin, Clindamycin and Gentamycin.

**Conclusion:** Hand hygiene is the most effective way to reduce the risk of health care associated infections. However, this case study has shown that many healthcare workers do not adhere to the five moments of hand hygiene despite having training and availability of hand washing facilities. The low practice of hand hygiene means that cell phones of health care workers can easily act as reservoir of transmissable organisms. This study has highlighted the need to identify factors that prevent the implementation of a known “best practice”. More studies need to be done to confirm if this is a general behavior in Tanzanian hospitals and to identify necessary interventions.

**Disclosure of Interest**: None declared

### P384 FACTORS INFLUENCING HCW’S CHOICE OF HAND HYGIENE PRACTICES IN A DEVELOPING COUNTRY

#### Y. F. Lee^1,2^, W. Zingg^3^, M. McLaws^4^, L. M. Ong^5^, H. H. Chua^6^, S. Y. Wong^6^, D. Pittet^3^

##### ^1^Institute of Global Health, Geneva University, Geneva, Switzerland; ^2^Ministry of Health, Kuala Lumpur, Malaysia; ^3^Infection Control Program and WHO Collaborating Centre on Patient Safety, University of Geneva Hospitals and Faculty of Medicine, Geneva, Switzerland; ^4^School of Public Health and Community Medicine, University of New South Wales, Sydney, Australia; ^5^Clinical Research Centre & Department of Medicine, Penang; ^6^Sarawak General Hospital , Kuching , Malaysia

###### **Correspondence:** Y. F. Lee

**Introduction:** One of the nine key recommendations arising from the World Health Organization (WHO) Guidelines on Hand Hygiene (HH) in Health Care is the provision of a readily accessible alcohol-based handrub (ABHR) at the point of patient care for use by healthcare workers (HCWs).

**Objectives:** To evaluate the perception and beliefs of ward staff towards their choice of HH practices; hand wash (HW) versus locally developed hand rub (HR).

**Methods:** The project was conducted on two acute medical wards in Sarawak, Malaysia. Staff on both wards were educated on correct HH practices by a staff identified as a change agent (CA) who received training by Geneva University Hospital, the WHO collaborating centre for Patient Safety, Geneva. 111 staff received education sessions by CAs during a 56-days intervention period. 12 groups of Question & Answer sessions using a structured questionnaire were then conducted by a local infection prevention and control expert to evaluate staff choice of HH practices. Staff oral responses were clarified whenever necessary. All sessions were recorded, transcribed verbatim and translated to by an expert court interpreter. The average duration of a session was 45 minutes to 1 hour totalling ≈608 minutes.

**Results:** After education on correct HH practices, more than half the staff, from all professions, preferred to HW and were more confident that HW would mechanically remove germs. Preferences for HW including dislike of the smell of ABHR, poor environmental cleaning and that transmission of sporulating microorganism onto surfaces will occur unless HW is performed.

**Conclusion:** Staff prefer and tolerability of locally made ABHR can not be overcome with intensive education. However, staff practice for HW will still provide successful implementation the WHO Multimodal HH Improvement Strategy if staff can commit to the required number of HW per shift. Otherwise industrial ABHR may circumvent the current preference for HW.

**Disclosure of Interest**: None declared

### P385 IMPACT OF SCARE TACTICS ON HAND HYGIENE COMPLIANCE BY NIGERIAN UNIVERSITY STUDENTS THAT SHARED CONTAMINATED RESTROOMS THROUGH FACE-TO-FACE AND WHATSAPP VOICE MESSAGE

#### I. Yusuf^1^, H. I. Muhammad^2^

##### ^1^Microbiology, Bayero University, Kano, Kano; ^2^ICT Unit, Hydraulic Equipment Development Institute, Kumbotso, Kano, Nigeria

###### **Correspondence:** I. Yusuf

**Introduction:** Acquisition of community-associated infectious agents from dirty environments is widely reported in Nigeria. For two consecutive years, restroom floors of congested student hostels and other high-touch surfaces such as corridor floors, toilet and hostel room door handles are frequently contaminated with multidrug resistant (MDR) bacteria before and 1 hour of cleaning and disinfection. More recent isolation of strains of MDR present in contaminated surfaces from the hands of restroom users necessitates this study.

**Objectives:** The objective is to determine how scaring students on the dangers of MDR via face-to-face and through whataspp voice message can impact on their hand hygiene compliance after use of contaminated restrooms.

**Methods:** Two sets of 50 students (A and B) sharing 5 restrooms at different hostel blocks were selected. Practice of hand hygiene with water, ordinary and medicated soaps and alcohol hand rubs after restroom use was assessed before and after scaring voice messages on dangers of MDR and high chance of the acquiring MDR in their restrooms was passed verbally and through whatsapp. Improvement in hand hygiene was determined by the number of participants that used alcohol hand rub placed at about 3 m away from the restroom for 4 weeks.

**Results:** Prior knowledge of danger of MDR was low; it is 16% and 12% in set A and B respectively. The overall practice of hand hygiene with water, ordinary and medicated soap by all the participants were 63, 57 and 23% respectively. Prior use of hand sanitizer was very low (6%). Scaring the participants face-to-face significantly resulted in improving compliance by 56% as against use of whatsapp voice message which only resulted in 11%. There is relative improvement in hand hygiene for up to 4 weeks in set A scared via physical voice, unlike set B which lasted for only 1 week. Follow-up questions on MDR were received from 44 and 18% of participants from set A and B respectively.

**Conclusion:** Incorporation of scaring strategy into teaching of hand hygiene to non-health care workers can encourage participation and compliance. Dissemination of information via face-to-face engagement impact positively in improving hand hygiene practice than using whatsapp.

**Disclosure of Interest**: None declared

## Poster session: Local production of alcohol-based handrub

### P386 LOCAL PRODUCTION OF HYDRO-ALCOHOLIC SOLUTIONS AS A KEY STRATEGY FOR HAND HYGIENE PROMOTION IN BENIN

#### A. K. Aissi^1^, P. Gounadon^1^, L. Affovehoundé^1^, S. Assavedo^2^, M. I. Hotèyi^2^, C. Gnimadi^3^, C. C. Adjidé^4^

##### ^1^National Directorate of Public Health; ^2^Ministry of Health; ^3^Beninese Centre for Scientific Research and Innovation , Cotonou, Benin; ^4^ Laboratory of Biological & Environmental Risk Hygiene, Amiens Picardie University Hospital, Amiens , France

###### **Correspondence:** A. K. Aissi

**Introduction:** In most healthcare facilities in Benin, the indicator of availability of equipment and consumables needed for hand hygiene compliance according to WHO guidelines is less than 20 %.

**Objectives:** Allow healthcare workers to have continuous access to sufficient amounts of hydroalcoholic solution for better Hand Hygiene compliance during care.

**Methods:** -

- Training workshop for 32 hospital lab technicians on the guidelines for local production of hydroalcoholic solutions;

- Logistical support from WHO and Belgian Development Agency for supplying the essential material needed for production of hydroalcoholic solutions in 6 hospitals as an experiment;

- Awareness of IPC Commitee members and of staff in general, respectively on the rational management of hydroalcoolique solutions and their efficient use according to WHO guidelines;

- Administration of WHO standardized questionnaires to of stakeholder on knowledge and perceptions related to hand hygiene and healthcare associated infection risk;

- Organization of the World Hand Hygiene Day with showing to the press of locally produced hydroalcoholic solutions samples.

**Results:** -

- Hydroalcoholic friction is viewed by 75% of IPC commitee managers as an effective, fast-track and less constraining alternative than hand-washing for preventing healthcare associated infections;

- An average monthly requirement of 100 L of hydroalcoholic solution was estimated by Hospital. This quantity would be about 8 times cheaper than the same amount purchased from importers according to hospital managers, of whom 83% didn't know that effective local production of hydroalcoholic solution was possible;

- All of them have committed to allocate a specific budget for maintaining this local production. A mapping of prioritization of the place of care with a high level of risk of manupired transmission of microorganisms was carried out within each facility.

**Conclusion:** The initiative to produce the hydro-alcoholic solution locally is wise, especially in a resource-limited country like Benin. However, a regulatory framework must be established to ensure and maintain quality assurance in each production unit.

**Disclosure of Interest**: None declared

### P387 LOCAL MANUFACTURE OF ALCOHOL BASED HAND RUB TO PROMOTE HAND HYGIENE IN UGANDAN HEALTH FACILITIES.

#### J. S. Nanyondo, W. Omuut

##### Global Health Security, Infectious Diseases Institute, Kampala, Uganda

###### **Correspondence:** J. S. Nanyondo

**Introduction:** Alcohol-based hand rub(ABHR) is the gold standard of care for hand hygiene practice in healthcare settings [i]and proper use can reduce health care associated infections (HCAIs) by as much as 40%.[ii] However, the cost of ABHR ($8-10 per litre for commercial supplies) is considered to be the main impediment for supply.

**Objectives:** 1.To promote local manufacture of ABHR in order to promote hand hygiene in public facilties in Uganda.

2.To establish the cost of local manufacture of ABHR versus commercial ABHR

2.To monitor hand hygiene compliance in public facilities in Uganda

**Methods:** Infection Prevention and Control committees were trained to manufacture ABHR as part of a broader package of interventions including general IPC principles. To initiate the program, facilities were provided with 100 litres of purchased commercially-prepared ABHR. Requisitions for constituents of ABHR were made through the National Medical Stores (NMS) system. Facilities were also provided with tools to distribute ABHR supply and monitor monthly ABHR consumption. Cost inputs were obtained from NMS price lists and IDI procurement invoices Finally, IPC committees were trained to assess hand hygiene compliance based on the WHO 5 moments of hand hygiene framework.

**Results:** Three regional referral hospitals (Mubende, Jinja and Mbale), manufactured 160,80 and 90 litres of ABHR respectively. Mean production cost was $3.70 (13,700/=) per liter versus $6.80 (25,000/=) per liter of commercial ABHR (45% saving). Over time, hand hygiene compliance in Mubende and Jinja increased from a baseline of 22.0%, and 10.9% in 2017, to peaks of 54% and 50% in 2018, respectively.

**Conclusion:** Facilities that benefited from comprehensive IPC support program including local manufacture of ABHR registered improved hand hygiene compliance. Feasibility of scaling up local ABHR production is being explored.

**References**

[1] (WHO 2017)

[2] (Kamph,2009).

**Disclosure of Interest**: None declared

### P388 ALCOHOL BASED HAND RUB LOCAL PRODUCTION IN SIERRA LEONE

#### A. Maruta^1^, B. V. Chaibva^1^, R. E. Williams^2^

##### ^1^IPC, World health organisation; ^2^IPC, Ministry of Health and Sanitation, Freetown, Sierra Leone

###### **Correspondence:** A. Maruta

**Introduction:** Hand hygiene process is an essential and cost-effective way to minimize healthcare associated infections in hospitals. Given the lack of infrastructure in most hospitals in Sierra Leone (lack of sinks with clean running water, soap and disposable towels), alcohol-based hand rub (ABHR) is the best solution to providing the means to hand hygiene when indicated at the point of care.

**Objectives:** Training of healthcare in ABHR production was in line with the strategy to strengthen infrastructures relating to hand hygiene in hospitals. The main objectives were to demonstrate the ABHR production process from primary materials to finished product, the ABHR quality control process from primary materials to finished product and assess each participant's ability to perform ABHR production

**Methods:** Fourteen participants were identified from the pharmacy and nursing departments of Sierra Leone. The participants underwent a theoretical and practical training on alcohol based hand rub production. The theoretical training was conducted over 3-days. A demonstration of production process was conducted followed by supervised production. Each participant managed to work as an operator and as a double check. The recommended World Health Organization Alcohol Based Hand-rub Formulation 1 was used during the process of production. University of Geneva Teaching hospitals record keeping forms were adopted during the manufacturing and quality control. Alcohol content determination quality control was done using a Gay Lussac alcoholmeter, and semi-quantitative determination of hydrogen peroxide using quantofix test strips.

**Results:** Seven groups of two participants (operator and checker) where trained in Sierra Leone. Each group managed to produce 3 batches of 10 litres and 3 batches of 20 litres Alcohol Based Hand-rub solution 80% v/v. The average concentration of first group was 80.4% v/v after adjusting for recorded temperature. Ninety eight percent (41/42) of the batches had hydrogen concentration of 150mg and 2.4% (1/42) had a concentration of 300mg/ml. All produced batches were compliant and released for use after 72 hours of quarantine.

**Conclusion:** Sierra Leone successfully managed to have 14 pharmacy and nursing staff trained in local production of alcohol based hand rub. Sustainability of the program is essential to allow the continuous supply of hand rub to all facilities.

**Disclosure of Interest**: None declared

### P389 FEASIBILITY OF LOCAL PRODUCTION OF ALCOHOL-BASED HANDRUB IN LOW- AND MIDDLE-INCOME COUNTRIES

#### C. Guitart, D. Pittet

##### SPCI, HUG, Geneva, Switzerland

###### **Correspondence:** C. Guitart

**Introduction:** Hand hygiene (HH) remains the most effective action to prevent healthcare-associated infections. According to the WHO multimodal strategy for HH improvement, the first essential element is ‘system change’, i.e. the replacement of soap and water handwashing by alcohol-based handrub (ABHR) at the point of patient care. Unfortunately, commercially-available ABHR agents are seldom available and affordable in low- and middle-income countries (LMIC). To overcome this problem, WHO proposed two ABHR formulations, with specific components and a methodology adapted for their local production.

**Objectives:** We assessed the feasibility of the local production of ABHR in LMIC.

**Methods:** A literature search was conducted (March 2019). Index and free terms text for ABHR, hand hygiene and local production were searched on Medline, Pubmed and Google scholar. All articles in English and French languages are reviewed.

**Results:** The last systematic review was conducted more than 10y ago. Over the past decade, there have been several examples of local production of ABHR as part of multimodal approaches to improve HH, from single hospital pharmacy to national level. The feasibility testing, quality control, and costs of local ABHR production have led to very encouraging results, particularly in terms of cost reduction compared to commercial products available in LMIC. There are, however, barriers to the accessibility of ABHR in LMIC, including difficulties in the procurement of basic ingredients, distribution constraints, and discontinuation of regular budget allocation. One emerging solution for the development of country-based capacity in ABHR production has been promoted through South-North partnership mechanisms. In 2006, such partnership was developed and organized practical ABHR production workshops with quality control. Tools are available online for wide replication in LMIC all over the world. Summaries of tools for change and main implementation barriers as well as reasons for successful models will be presented.

**Conclusion:** Our findings demonstrate that ABHR local production occurs in hospital pharmacies or at national level worldwide. Demonstration of successful models based on local ABHR production would be highly beneficial since the literature remains scarce.

**Disclosure of Interest**: None declared

## Poster session: Patient participation

### P390 PATIENT INVOLVEMENT IN THE HAND HYGIENE PERFORMED BY HEALTHCARE WORKERS AND PATIENTS: PERCEPTIONS AND PREFERENCES USING A VALIDATED QUESTIONNAIRE

#### J. Y. Kawagoe^1^, R. G. Barison^1,2^

##### ^1^Professional Master's Degree in Nursing , Albert Einstein Israelite School of Health Sciences, São Paulo; ^2^Nursing , Hospital do Coracão, Londrina, Brazil

###### **Correspondence:** J. Y. Kawagoe

**Introduction:** The World Health Organization recommends patients involvement to increase hand hygiene (HH) compliance by healthcare workers (HCW) to prevent healthcare associated infections (HAI).

**Objectives:** To develop, validate and apply an instrument to identify patients' preferences on their involvement in active or passive, in the HH performed by HCW and by themselves, and preferences about HH education strategies.

**Methods:** Cross-sectional, mixed and sequential research performed in 4 phases (PI to PIV). PI - focus group (FG) performed with inpatients to develop the instrument in PII. PIII - instrument validation: a) content validation by a panel of 2 groups of judges (expert and general public), using Delphi technique with 0.80 of the content validity index (CVI) and the Gwet AC2 coefficient of agreement (CA) and b) apparent content validation pre-test, using cognitive interview (CE) with inpatients. Hospitalized patients answered the questionnaire in PIV.

**Results:** After two FG sessions with 24 patients (PI), the instrument (46 questions) were developed (PII) and this was considered validated (PIII) after a) two rounds of validation (CVI and CA >0.80): 25 and 16 participants in the first and second-round respectively and b) CE with 20 patients. The instrument applied to 100 inpatients (PIV) showed the preferences in HH participation: active/passive (49%), passive (33%) and active (16%), p <0.001. If there is a HCW’s invitation for the patient participation, they would feel comfortable to remind them to perform HH: physician (55%), nurse (60%) and nursing technician (58%), p >0.99. If there’s no invitation, the willingness to remember the HCWs reduces significantly: physician (26%) nurse / nursing technician (34% / 35%), p<0.035. The preferences of the educational strategy about the importance of HH were: posters (41%) and verbal orientation (24%).

**Conclusion:** Patients tend to participate actively in the practice of HH if there is an invitation from the HCW to remind them; the preferences for information about HH were mainly through posters and verbal guidance by HCWs. The instrument identified patient preferences regarding their participation in the HH program.

**References**

World Health Organization. Guide to Implementation. A Guide to the Implementation of the WHO Multimodal Hand Hygiene Improvement Strategy. Geneva. 2009. 48 p.

**Disclosure of Interest**: J. Kawagoe Employee of: Patient Safety Consultant - B Braun, R. Barison: None declared

### P391 THEN AND NOW (1989-2019): PATIENT EMPOWERMENT AND AWARENESS OF HEALTHCARE-ASSOCIATED INFECTIONS

#### M. McGuckin^1^, J. A. Storr^2^, J. Govednik^1^

##### ^1^McGuckin Methods International, Ardmore, United States; ^2^Global Health, S3 Global, London, United Kingdom

###### **Correspondence:** M. McGuckin

**Introduction:** Our team compared two studies (1989^1^, 2019) on consumer awareness of healthcare-associated infection (HAI) risk, noting differences in consumer perception of being empowered and involved in reducing HAI risk.

**Objectives:** To identify changes in consumer knowledge of HAIs and awareness of risks over time.

**Methods:** Partnering with a consumer research organization with HAI experience, our team surveyed 1,004 consumers in the United States (US) in March 2019 via the internet. Completed interviews were weighted by demographic variables to ensure reliable and accurate representation of the total adult population. Significance testing was done to the 95% confidence level. Results were compared to a 1989 survey conducted by the University of Virginia, US, from a sample size of 976.

**Results:** 65% of respondents were aware of the risks of HAIs prior to our survey. 41% were satisfied with the information they received on HAI risk during their last hospitalization, 7% dissatisfied, 36% did not receive information, 11% didn’t remember. Only 28% believed HAIs were preventable. 40% would have $1-$30 per day added to their hospital bill to fund programs that educate patients on HAI risk; 4% more than $30/day. 29% used a medical professional as a source for HAI information, 29% website/web search, 22% friend/family, 15% TV/radio, 14% social media such as YouTube, 13% government health website, 11% newspaper/magazine. 39% had not used any source.

**Conclusion:** Awareness of HAI risk has not changed in three decades (62% vs. 65%). The percentage of respondents having an HAI remained at 12%. In 1989, 62% were dissatisfied with the information received from their hospital about HAIs compared to 7% in 2019. In 1989, 83% believed HAIs were preventable; now only 28% believe so. Willingness to pay for better HAI patient education decreased (57% vs. 44%). Despite 30 years of education and empowerment, consumers are not more aware of HAIs, less willing to pay more for HAI reduction programs, and more accepting of HAIs as a risk of healthcare. However, the quality of information is better for those who report receiving it. We now have a baseline of HAI information sources: medical professionals and websites emerge as top sources in this study.

**References**

1. Miller PJ, Farr BM. Survey of patients' knowledge of nosocomial infections. Am J Infect Control. 1989 Feb;17(1):31-4.

**Disclosure of Interest**: None declared

## Poster session: Education

### P392 INTENSIVE CARE NURSING TEAM’ KNOWLEDGE, ATTITUDES AND PRACTICES REGARDING ORAL CARE OF CRITICALLY ILL PATIENTS

#### A. Felix^1^, I. D. T. A. Amaral^1^, B. R. Paz^1^, J. A. Silva^1^, B. R. Santos^1^, M. S. Coyado^1^, L. A. Gomes^1^, A. Leal^2^, M. Carmo^3^

##### ^1^Enfermagem, Faculdade de Ciências Médicas da Santa Casa de São Paulo; ^2^Enfermagem, Hospital Santa Isabel; ^3^Enfermagem, Hospital Santa Casa de Misericórdia de São Paulo, São Paulo, Brazil

###### **Correspondence:** A. Felix

**Introduction:** Oral hygiene (OH) is a standard part of a daily nursing care and is a strategy for lowering the incidence of ventilator-associated pneumonia (VAP). Although OH is a fundamental practice nursing team provides their patients, undertaking this practice can be challenging for a range of reasons.

**Objectives:** To assess the intensive care nursing team' knowledge, attitudes and practices regarding oral care of critically ill patients.

**Methods:** Cross sectional study design was used to achieve the aim of the study. The study was conducted in four Intensive Care Units (ICU) at a public hospital in São Paulo, Brazil. A convenience sample of nursing professionals was invited to participate in this study in December 2018. The survey instrument was designed by the investigators and it was previously validated by specialists. The instrument included questions about demographic characteristics; knowledge towards oral hygiene (13 items); attitudes and practices regarding oral care (15 items). The data was analyzed using descriptive statistics methods. This study was reviewed and approved by the Research Ethics Committee (No 3033243) of the medical center where the study took place.

**Results:** Eight-one nursing professionals (nursing technician=60% and auxiliary=7% and nurses=19%) agreed to participate in this study. The majority of the sample was female (n=64, 79%) with an average age of 37 years old. Thirty-nine (48%) participants related that ICU work experience was less than five years and sixty-three (78%) related have no more than one job. Most responded (n=44, 54%) answered no to have received training toward oral hygiene in critically ill patients. The majority of nursing professionals demonstrated positive knowledge, however the majority of them had fair attitude and practice toward oral care in critically ill patients in ICU. Lack of time and materials were pointed out as a barrier to perform OH.

**Conclusion:** These findings suggest the requirement for permanent education for reinforcing the importance of proper OH in ICU and working with hospital managers to allow sufficient time and materials to attend to oral care as a recommended by the literature.

**Disclosure of Interest**: None declared

### P393 EVALUATION OF AN ADVANCED INFECTION PREVENTION AND CONTROL (IPC) TRAINING PACKAGE

#### S. Tomczyk^1^, A. Twyman^2^, B. Hakizimana^2^, J. Storr^2^, N. Damani^2^, K. Wilson^3^, B. Ndoye^4^, A. Baller^2^, N. Dayanghirang^5^, B. Allegranzi^2^

##### ^1^Robert Koch Institute, Berlin, Germany; ^2^World Health Organization, Geneva, Switzerland; ^3^Centers for Disease Control and Prevention, Atlanta, United States; ^4^Infection Control Africa Network, Dakar, Senegal; ^5^Regional Office for Africa, Brazzaville, Congo

###### **Correspondence:** A. Twyman

**Introduction:** IPC training and education is one of the eight core components for effective IPC programmes as defined by the World Health Organization (WHO). However, IPC expertise and opportunities for IPC training are limited, especially in low and middle-income countries.

**Objectives:** We aimed to evaluate a newly developed WHO advanced IPC training package.

**Methods:** An IPC expert consultation identified ten priority training topics which were subsequently developed by global IPC experts in English and French. The training package was first evaluated at ICPIC 2017, then in two field pilots with IPC focal persons in Liberia and Senegal. The evaluation targeted participants, facilitators and observers and included a knowledge test and structured questions on teaching methods, content, time allocation, and general effectiveness using a Likert scale of 1 (strongly agree) to 5 (strongly disagree). Data collection included written evaluations, focus group discussions, pre/post-tests, facilitator briefings, and observer notes. All data collected were analysed descriptively.

**Results:** Participants were 30, 37 and 26 from 35 countries during the initial testing, Liberia and Senegal pilots, respectively. In all three testing occasions, the average scores ranged between 4.1 and 4.7 for all questions, apart from “adequate time allocation for the training” which scored 3.5 at the ICPIC testing. The average pre/post-test scores went from 12.3 to 19.6 (41% to 65%) in Liberia and 17.5 to 21.8 (58% to 73%) in Senegal. Participants considered the IPC content as appropriate although specific aspects missing or unclear were noted. They highlighted the value of practical hospital sessions, videos and demonstrations although some modules were noted as needing additional examples, particularly from low-resource settings. It was suggested that the course length ideally could be increased.

**Conclusion:**

We used a robust evidence-based approach to evaluate the WHO IPC advanced training package and improve its quality to support countries in bridging the gap in IPC expertise.

**Disclosure of Interest**: None declared

### P394 DEVELOPMENT AND EVALUATION OF SIMULATION CLINICAL SCENARIOS TO TEACH INFECTION PREVENTION AND CONTROL

#### R. M. Figueiredo^1^, A. A. L. Dias^2^

##### ^1^Nursing Department; ^2^Post graduate Program , Federal University of Sao Carlos, São Carlos, Brazil

###### **Correspondence:** R. M. Figueiredo

**Introduction:** To teach prevention and control of healthcare-associated infections (HAIs), it is recommended the use of novel methods that stimulate critical thinking, such as simulation. The simulation clinical scenarios to be used should be developed based on scientific evidence, have fidelity, be tested and replicable.

**Objectives:** To develop and validate simulation clinical scenarios to teach methods for the prevention and control of HAIs to nursing students.

**Methods:** Two-stage methodological study conducted at a public university in Brazil: 1) development and content validation of two simulation scenarios; and 2) test of the scenarios and evaluation of their design using the Simulation Design Scale (SDS).

**Results:** The simulation scenarios were developed according to the National League for Nursing Jeffries Simulation Theory. The themes standard precautions, specific precautions, and bloodstream and urinary tract infections were used. Ten experts participated in the content validation and achieved an agreement of at least 80%. In the second stage, 44 students participated. Twenty-eight (63.4%) were mid-course students; 28 (63.6%) actively participated in the scenarios, and 23 (52.3%) had previous experience with simulations. The mean score for the design elements was 4.7/5.0 with the internal consistency of 0.86. The mean score for the item importance was 4.8/5.0, with the internal consistency of 0.93. The SDS items with the smallest means were those that assessed fidelity (4.5 and 4.6). Being a senior (p= .021) and having previous experience with simulation (p=.001) were significantly associated with higher rating scores for the simulation. There were no differences between students that participated or were observers in the simulation.

**Conclusion:** The results of this study demonstrated that the lack of environmental fidelity did not compromise the evaluation of the simulation design. The simulation clinical scenarios developed were shown to be appropriate for the population under study, and feasible as a method to teach prevention and control of HAIs.

**Disclosure of Interest**: None declared

### P395 MEDICAL OFFICERS AND NURSES AS KEY STAKEHOLDERS IN LEVEL 4 AND 5 FACILITIES IN LOW- AND MIDDLE-INCOME COUNTRIES

#### A. Sway^1^, A. Wanyoro^2^, S. Senglaub^1^, A. Oburu^3^, J. Solomkin^1^ on behalf of World Surgical Infection Society

##### ^1^World Surgical Infection Society, Cincinnati, United States; ^2^Obstetrics and Gynecology, Kenyatta University, Nairobi; ^3^ACE Research, Kisumu, Kenya

###### **Correspondence:** A. Sway

**Introduction:** A core component of the WHO IPC recommends that staffing levels be adequate for the patient workload. The lack of healthcare workers in low- and middle-income countries, particularly in sub-Saharan Africa is well documented; one way to alleviate the burden of surgical infections without a large commitment of resources is to better train and educate existing facility staff.

**Objectives:** To understand the current allocation of the surgical workload in district level hospitals in Kenya in order to create targeted task-shifting and education programs.

**Methods:** We developed a cross-sectional survey on IPC practices, adapted from the World Health Organization (WHO) Guideline on Core Components for IPC and the WHO Emergency and Essential Surgical Care Situational Analysis Tool, for use in district level facilities in low- and middle-income countries. Survey components included IPC guidelines, surveillance, workload and environment, hospital characteristics, clinical workforce, obstetrical outcomes, and perioperative care practices. In a pilot feasibility trial of the survey tool, data was collected from 27 district level hospitals in Kenya from March to May 2019. These facilities were selected in part based on their capacity to provide comprehensive maternal and obstetric care, including cesarean operations, as cesareans make up the majority of surgeries in the regions and are associated with high SSI rates.

**Results:** The facilities included in the survey pilot serve an estimated population of 3,615,166. The average number of annual admissions was 7,801 (Range: 1,190-25,783). Thirteen of the 27 total facilities (48.1%) had no certified surgeons, six facilities had one certified surgeon (22.2%), and eight facilities employed two or more certified surgeons. In contrast, almost all facilities (n=25; range: 2-15) employed Medical Officers who performed cesarean operations. The number of nurses in surgical and OB/GYN wards ranged from four to 101, with an average of 27.

**Conclusion:** Dedicating specialized training and education to medical officers and perioperative nurse, and engaging them as key stakeholders in any infection prevention and control projects is key to creating practical and sustainable change

**Disclosure of Interest**: None declared

### P396 VIRTUAL LEARNING FOR CAPACITY BUILDING OF HEALTHCARE WORKERS TO IMPROVE ANTIMICROBIAL STEWARDSHIP OUTCOMES IN RESOURCE-LIMITED SETTINGS

#### L. C. Nalule, J. W. Arinaitwe, A. Bakabweyaka, P. Buyego, M. Ssekitya, R. Kikonyogo

##### Training Department, Infectious Diseases Institute, Makerere University, Kampala, Uganda

###### **Correspondence:** L. C. Nalule

**Introduction:** Uganda has low numbers of health workers, to bridge this gap task shifting has been done with mid-level practitioners providing clinical care even for patients who should ideally be handled by specialists.

**Objectives:** Training opportunities to equip the mid-level practitioners and health workers in general are costly and limited. Through e-learning, affordable and convenient learning opportunities are being provided by the Infectious Diseases Institute (IDI) to equip these health workers with the necessary knowledge and skills to build their technical capacity in clinical management including promotion of Antimicrobial Stewardship(AMS) and handling Antimicrobial Resistance (AMR).

**Methods:** IDI’s open access Virtual Learning Environment(VLE) hosts peer-reviewed case studies on AMS, AMR surveillance reports and guidelines that are freely accessible to health workers, utilization of these resources is evaluated through an online post-test and online certification is subsequently provided. A toll-free phone line is made available to participants in case they have queries related to the cases or other clinical issues.

**Results:** AMS resources were launched in March 2018, so far 5 case studies, 2 surveillance reports and 1 AMS success story were uploaded and accessed by over 160 health workers with 52 certificates downloaded after completion of learning. Low numbers of certificate downloads are attributed to among other reasons, a pass mark which muct be obtained. However, health workers cite internet challenges, ward burn out as some of the barriers to accessing these resources.

**Conclusion:** E-Learning can be an effective capacity building avenue for health workers in AMS and has potential for scale up if barriers are addressed.

**Disclosure of Interest**: None declared

### P397 ENHANCE INFECTION PREVENTION KNOWLEDGE & COMPETENCIES OF HEALTH CARE WORKERS AT AKUH.K

#### Z. Rafique, S. F. Mahmood, R. Roshanali, N. Virani, R. Khowaja

##### Infection Prevention and Hospital Epidemiology, Aga Khan University and Hospital, Karachi, Pakistan

###### **Correspondence:** Z. Rafique

**Introduction:** Staff development refers to the process whereby employees of an organization enhance their knowledge and skills in directions that are advantageous to their role in the organization. Infection control department conduct different on job training to enhance staff knowledge and skills which helps to prevent and decrease hospital acquired infections (HAIs). Their training started from general orientation programme, ongoing in-service education and simulated workshop.

**Objectives:** Infection Control Department (IC) aimed to:

- Increase number of sessions/simulated workshop for all health care workers.
- Explore Online Mandatory Certification for HCW who are directly involved in patient care.

**Methods:** The PDSA Plan-Do-Study-Act model, a continuous quality improvement (CQI) tool, was used to enhance staff knowledge and competencies related to infection control. **Plan**: In Q4 2017 it was identified that the number of sessions conducted by ICNs were drastically decreased from 55 to 35, simultaneously staff engagement decreased from 1326 to 1200. **Do:** The problem identification was done through annual risk assessment at begin of the year and educational planner was developed. This planner includes session for general orientations, in-service sessions, infection control linked nurses session. The modules were revised as per guidelines, different strategies were used.**Study**: It was identified that staff will required to refresh their knowledge thus all the educational material kept on website for easy access. Pocket guide was developed in both versions. Simulated workshops were conducted. **Act**: It was identified that all the staff were not covered through session thatswhy worked with IT and launched mandatory on line certification for all the direct care staff.

**Results:** After the implementation of all the strategies drastic improvement was seen and we were able to achieve our targets. It was observed that our no of session increased more than 50% i.e. from 35 to 71 and staff engagement increased from 1200 to 1844.

**Conclusion:** Health care workers sessions are very essential to decrease hospital acquired infections and

mandatory on line course is essential to enhance staff knowledge related to infection control.

**Disclosure of Interest**: None declared

### P398 INFECTION CONTROL LINK NURSE PROGRAMS IN THE NETHERLANDS: HOW DO THEY WORK?

#### M. Dekker^1^, I. Jongerden^2^, M. de Bruijne^2^, C. Vandenbroucke-Grauls^1^, R. van Mansfeld^1^

##### ^1^Department of Medical Microbiology and Infection Prevention; ^2^Department of Public and Occupational Health, Amsterdam UMC, Vrije Universiteit Amsterdam, Amsterdam, Netherlands

###### **Correspondence:** M. Dekker

**Introduction:** Infection control link nurses (ICLN) are deployed to support implementation of infection prevention and control (IPC) guidelines

**Objectives:** The aim of this study was to explore the occurrence and variation of infection control link nurse (ICLN) programs in Dutch hospitals and to assess determinants that may influence effectiveness of these programs.

**Methods:** In this cross-sectional study one infection prevention expert per hospital was surveyed during a National Congress for Dutch infection prevention experts in 2018. Survey questions were based on literature that reports on ICLN programs, and The Template for Intervention Description and Replication checklist. This checklist is applicable in the reporting of complex interventions in a non-trial setting. Data was analyzed using descriptive statistics. Associations were calculated for variables that represent current best practices.

**Results:** In total, 72 of 86 questionnaires, representing 82 Dutch hospitals, were returned. A ICLN program is in place in 66.7% of the hospitals. The top 3 goals for these programs were to increase awareness for infection prevention, to create a liaison between the wards and the infection prevention and control team, and for ICLN to be a source of information for their peers. In 87.5% programs ICLN receive education, with a mode of ≤ 4 sessions per year. Respondents perceived that they were able to accomplish their IPC goals due to the help of ICLN, when expressed on a 10 point Likert scale, as 7 (IQR 6.0-7.0). Programs that included education on infection prevention topics as well as training in implementation skills were associated with higher perceived effects (median 7.0, IQR 7.0-8.0) than programs without such education (median 5.0, IQR 2.5-6.8) or programs where this education included only infection prevention topics (median 6.0, IQR 6.0-7.5).

**Conclusion:** ICLN programs have been implemented in two thirds of Dutch hospitals. The training of link nurses in implementation skills besides education on infection prevention topics is associated with higher perceived accomplishment of IPC goals by infection prevention experts.

**Disclosure of Interest**: None declared

### P399 COMPETENCY BASED INFECTION PREVENTION AND CONTROL ADVANCED CERTIFICATE TRAINING COURSE IN SIERRA LEONE

#### C. Conteh^1^, A. Maruta^2^, G. Kassa^3^

##### ^1^National Infection Prevention Control Unit , Ministry of Health and Sanitation; ^2^IPC, World Health Organization, Freetown, Sierra Leone; ^3^Quality Improvement, ICAP Columbia University, NewYork, United States

###### **Correspondence:** C. Conteh

**Introduction:** Situational analysis evidence and lessons gathered from the 2014 - 2015 Ebola outbreak highlighted the vulnerabilities of the Sierra Leone healthcare system, specifically the Infection Prevention and Control (IPC) lapses contributed to the ongoing threat to the health and safety of patients and healthcare workers. The Ministry of Health with support from CDC in collaboration with ICAP, introduced advanced IPC competency based training to develop a cadre of IPC experts to improve IPC capacity in Sierra Leone

**Objectives:** To advance healthcare workers’ IPC skills and knowledge and create a pool of IPC experts committed to reducing healthcare-associated infections in Sierra Leone.

**Methods:** A six-month course was designed for clinical practitioners with previous training in basic infection prevention and control. The program structure consists of three contact sessions, each incorporating classroom didactics, and hands – on clinical sessions, field assignments, and a pre – posttest. Face to face and virtual mentorship will be provided between in-person sessions. Competencies will be assessed during contact sessions and mentorship visits

**Results:** Twenty-five HCWs from across Sierra Leone participated in the first cohort of the Competency Based IPC Certificate Course in May 2019. Participants trained in microbiology, standard precautions, and transmission based precautions in the classroom, laboratory and clinical setting. Participants will return to Freetown for the second contact session in July 2019.

**Conclusion:** Competency based training is an effective way of building national capacity, increase access to IPC expertise at the central, district, and hospital levels of the health care system. Participants will Improve implementation and coordination of IPC in healthcare facilities and promote creative thinking and problem-solving skills around IPC issues to improve healthcare delivery.

**Disclosure of Interest**: None declared

### P400 TRANSLATING THE INFECTION CONTROL LINK NURSES ROLE INTO DAILY PRACTICE

#### T. Lauret, M. Dekker, R. van Mansfeld

##### VU MEDICAL CENTER, Amsterdam, Netherlands

###### **Correspondence:** T. Lauret

**Introduction:** Infection control link nurses (ICLN) are trained to disseminate their knowledge on infection prevention and control (IPC) to their peers. Current best practice for an ICLN program includes education on infection prevention topics as well as a clear description of the ICLN profile and training in implementation skills.

**Objectives:** To educate the ICLN in four interactive workshops which will be used to investigate the perception of the ICLN on their role and identify barriers for ICLN that may withhold them to fulfil their role.

**Methods:** A training program was developed to interfuse knowledge on IPC measures with executive and implementation skills. In 4 interactive workshops, situations common to the daily care practice of ICLN were simulated in role-plays. ICLN took part in the play as a patient, as a coworker or as themselves. All others observed the compliance with IPC protocols or the role of the ICLN. Role-plays were evaluated directly afterwards. During the last workshop the ICLN role and the impact of this role on various IPC topics was discussed. ICLN where asked to share experiences and their ideas on how to improve the 8 IPC topics that the role-plays were based upon, in daily practice.

**Results:** A total of 42 ICLN participated in the workshops and 62% of the ICLN attained more than one workshop. Evaluation of the role-plays and the discussion on the role revealed that ICLN interpretation of their role varied, despite the existence of a written role profile. Furthermore ICLN struggled to blend this role into their role as a nurse in daily practice.Ideas to overcome these barriers could be divided into 4 categories: knowledge, skills to fulfill the ICLN role, ideas for short term projects, and ideas for long term projects. To address the barriers identified in the workshops in a playful way, an IPC quartet was developed using experiences and ideas of the ICLN. It contains one instruction card and 32 play cards based on 8 IPC topics.

**Conclusion:** Barriers to fulfill the role of ICLN uncovered during this training program emphasize the need for collaboration between IPC experts and ICLN to translate the ICLN role into their day-to-day work. An IPC quartet could be a suitable tool to fulfill this need.

**Disclosure of Interest**: None declared

### P401 IMPROVING INFECTION PREVENTION AND CONTROL IN RECOVERY USING IN SITU SIMULATION

#### P. Silva^1^, M. Gomes^1^, J. Cotterill ^1^, J. Shackleton ^2^, L. Everett ^2^

##### ^1^Post Anaesthetic Care Unit; ^2^Infection Prevention and Control , Royal Brompton Hospital , London, United Kingdom

###### **Correspondence:** P. Silva

**Introduction:** Infection Prevention and Control (IPC) is a crucial indicator of quality and safety in healthcare settings, monitored to reduce patient infection and improve his outcome. Hand hygiene monthly audits in Recovery has fallen below 75% in three consecutive months, prompting urgent individual and team education to improve staff engagement and compliance with local and national IPC guidelines.

In situ simulation is an effective educational method, allowing teams to review and reflect about their own practice in their actual working environment, identify system weaknesses and develop solutions to overcome those, improving safety and the quality of care provided.

**Objectives:** In association with the IPC team, the aim of this project is to facilitate personalised education through in situ simulation and ultimately improve IPC and patient safety in Recovery.

**Methods:** Two in situ, high fidelity simulations were delivered to the Recovery team in the months of March and April. Those sessions were recorded and posteriorly analysed in relation to hand hygiene and protective equipment with the collaboration of the IPC team. A teaching session was then planned and delivered, including the visualization of the simulation records and facilitation of team reflection and discussion about IPC practices requiring improvement and the review of IPC guidelines and the five World Health Organization (WHO) moments of hand hygiene.

Added to the teaching session, a structural change was also made in the Recovery setting, with the reallocation of alcohol-based handrub foam into each bed space, to reinforce its usage.

**Results:** The results from the project will be ready for poster publication at the ICPIC Conference. Those will include the trend in Hand Hygiene Audit in the months of June to August and feedback forms from the Recovery team.

**Conclusion:** In situ simulation improves individual, team and organizational learning, promotes engagement and increases the opportunity to practice knowledge, skills and attitude. Through in situ simulation and consequent reflection over own practice and change of behaviours, this project endeavours to improve IPC practices and the quality of care provided in Recovery.

**Disclosure of Interest**: None declared

### P402 “INFECTION PREVENTION AND CONTROL IDEA CHALLENGE” CONTEST: A FRESH VIEW ON MEDICAL EDUCATION AND PROBLEM SOLVING

#### A. Arianpoor^1^, A. Zarifian^2^, E. Askari^3^, A. Akhavan Rezayat^2^, M. Dayyani^3^, A. Rahimian^3^, E. Amini^3^, A. Ziaeemehr^1^, W. Zingg^4^, M. H. Aelami^2^, D. Pittet^4^

##### ^1^Surgical Oncology Research Center, ^2^Infection Control and Hand Hygiene Research Center, ^3^Student Research Committee, Mashhad University of Medical Sciences, Mashhad, Iran, Islamic Republic Of, ^4^IPC program & WHO Collaborating Centre on Patient Safety, University of Geneva Hospitals and Faculty of Medicine, Geneva, Switzerland

###### **Correspondence:** A. Arianpoor

**Introduction:** Healthcare-associated infection (HAI) is a major challenge in modern medicine.

**Objectives:** We attempted to provide knowledge on infection prevention and control (IPC) to different audiences, focusing on education as an essential part of multimodal approaches towards IPC.

**Methods:** We sent a nation-wide invitation and gathered medical, nursing and dentistry students from all over Iran to present projects on “How to reduce HAI?” in a predefined structured format. After excluding non-conforming projects, two reviewers assessed all projects using a standardized protocol; the top twelve were selected. The contest was designed in three rounds. In first round, 12 teams were matched one by one and debated in a knockout manner, while the jury reviewed the scientific content and presentation skills to select the top five. In second round, the reviewers selected the top three projects for final presentation in front of a panel including renowned international experts to determine 1st to 3rd ranks.

**Results:** We received 49 projects, of which 11 (22%) were excluded due to non-adherence to the predefined criteria (10) and to plagiarism (1). The overall score of the retained 38 projects averaged 69.38±18.25 (SD; maximum score, 115). The average score of the 12 selected projects was significantly (*p* <0.001) higher than that of the 25 others. In first round, the top 5 projects scored significantly (*p* <0.001) higher than the 7 others, 147.6±9.22 vs. 116.97±9.13, respectively (maximum, 188). The average score of the three projects selected in the second round was 157.06 ± 5.40.

**Conclusion:** Building a powerful motivation combined with interactive educational methods might play an important role in adherence to guidelines to help implementing multimodal approaches and promote innovation in IPC.

**Disclosure of Interest**: None declared

## Poster session: Innovation

### P403 A REVIEW ON USING EMOTICONS TO IMPROVE HEALTH-RELATED BEHAVIOURS IN INFECTION PREVENTION AND CONTROL

#### N. Lotfinejad^1^, E. Tartari^2^, D. Pittet^2^

##### ^1^Mashhad university of medical sciences, Mashhad, Iran, Islamic Republic Of; ^2^University hospital of Geneva, Geneva, Switzerland

###### **Correspondence:** N. Lotfinejad

**Introduction:** Emoticons are ubiquitous symbols that enable us to convey emotional and non-verbal cues. In particular circumstances, emoticons may improve communication by increasing attention and effecting positively on behaviour and health.

**Objectives:** The aim of this review was to identify examples of using emoticons in medical literature, highlighting infection prevention and control (IPC) as a field that is significantly influenced by human behaviour.

**Methods:** We conducted PubMed and Cochrane database search for the keyword “emoticon” with no time limit. All identified original research studies using emoticons in IPC were summarized.

**Results:** Our search yielded only one eligible study published in 2018. By performing a field experiment research, Gaube et al. revealed the significant impact of injunctive norms on hand hygiene behaviour by using an emoticon-based feedback system.

**Conclusion:** Emoticons facilitate effective communication by giving personality to the written text. However, despite the prevalent use of these global symbols, there are currently limited data available concerning the role of emoticons in health behaviours particularly related to IPC as a major component of health systems. According to the satisfactory results of the only evidence existing, further research is necessary in the health system.

**Disclosure of Interest**: None declared

### P404 ENABLING INFECTION PREVENTION IN HEALTHCARE FACILITIES: USING DATA AND SUPERVISION AS LOW-COST TOOLS TO ESTABLISH AND MAINTAIN WATER, SANITATION, HYGIENE, WASTE MANAGEMENT AND CLEANING SERVICES IN LOW AND MIDDLE-INCOME COUNTRIES.

#### J. Lopez^1,2^, S. Sara^1,2^

##### ^1^Global Health, Save the Children; ^2^Maternal and Child Survival Program, USAID, Washington, United States

###### **Correspondence:** J. Lopez

**Introduction:** Mounting evidence highlights the lack of sustainable water, sanitation, hygiene (WASH), waste management and cleaning services in healthcare facilities (HCFs) across LMICs and its effects on infection prevention compliance. USAID’s Maternal and Child Survival Program (MCSP) tested a low-cost approach to increase infection prevention readiness through institutionalizing performance-based monitoring and accountability systems in 145 HCFs across four countries (DRC, Guatemala, Haiti, Nigeria).

**Objectives:**

- Assess whether implementation of the Clean Clinic Approach resulted in increased infection prevention readiness among participating HCFs and within individual wards (outpatient, labor & delivery, maternity wards, sick newborn wards).

- Determine the sustainability of improvements over time

**Methods:** MCSP, in partnership with national ministries of health (MoH), designed national WASH and infection prevention scorecards for HCFs and specific wards. The MoH, alongside MCSP, conducted quarterly observational surveys assessing the availability of WASH services and levels of HCF infection prevention readiness.

**Results:** Results show significant and sustained improvements in WASH and infection prevention readiness across countries. In Guatemala, participating HCFs increased their infection prevention readiness score from an average of 46 to 89 points (100-point scale) while HCFs in DRC improved from 41 to 70 points. The availability of basic WASH services and infection prevention supplies were observed across wards, with little or no external investments.

**Conclusion:** In LMICs, WASH infrastructure investments are often ill maintained and needs outpace funding. Low cost and scale-able interventions are required to increase and maintain WASH services to enable infection prevention practices. Results from Clean Clinic Approach implementation demonstrate that substantial progress can be made to improve infection prevention readiness in HCFs without waiting for expensive, large-scale infrastructure investments. Low-cost system changes can empower managers within wards, HCFs and local governments, to make and sustain improvements independently of external investments.

**Disclosure of Interest**: None declared

### P405 THE IMPACT OF QUALITY IMPROVEMENT METHODOLOGY TO IMPROVE INFECTION CONTROL PRACTICES

#### I. A. Bangura, C. A. Conteh

##### National Infection and Control Unit, Ministry of Health, Freetown, Sierra Leone

###### **Correspondence:** I. A. Bangura

**Introduction:** The National Infection Prevention and Control Unit (NIPCU) established in Sierra Leone post Ebola outbreak with the mandate to oversee IPC implementation in all healthcare facilities, collaborated with ICAP Columbia University to launch Rapid Improvement Methodology (RIM) initiative in 2017. The main focus was on improving compliance to IPC standards on Environmental Cleanliness (EC) Waste Management (WM) and Personal Protective Equipment (PPE) The quarterly National IPC assessment conducted in 2018 revealed a lower compliance rate of the above stated elements. To effectively monitor implementation on IPC performances, NIPCU adapted the RIM model to strengthen evidence based practice and improve quality of care.

**Objectives:** NIPCU scaled up the RIM QI initiative in 2018 in 8 secondary and 5 tertiary hospitals in the western area Freetown the capital city of Sierra Leone to improve compliance in Environmental Cleanliness (EC), Waste Management (WM) and Personal Protective Equipment (PPE)

**Methods:** A rapid improvement model (RIM) version of the QI collaborative with a compressed timeframe of 6 months was applied. Checklist adapted from the RIM project implemented in pilot hospitals. 4 units in each facilities was assessed. Facility staff setup quality improvement teams, 5 days intensive training on QI, weekly coaching and mentorship provided by the National team and weekly data collection from each facility. Monthly learning sessions bringing all 13 QI teams together to review progress, exchange best practices and adapt change packages that worked for other facilities. The final harvest session and presentations was done at facility level to management teams.

**Results:** Compliance across all 13 HCF improved, WM from 69% to 84%, EC from 60% to 76% and PPE from 67% to 76%. Almost all HCF achieved their aim within the 6 months and sustained until the end of the project. The QI teams in each facility are now scaling up the RIM project to all other wards/department in their facilities

**Conclusion:** The RIM QI approach is practicable and realistic and has shown an improvement in the first quarter IPC assessment in 2019.Best practices identified have been scaled up to all 16 district hospitals in the country in April 2019 by the national infection and control unit (NIPCU).

**Disclosure of Interest**: None declared

### P406 ANIMAL-ASSISTED INTERVENTIONS AND INFECTION CONTROL IMPLICATIONS

#### N. Verbraeken, L. Blommaert, P. Bosmans, I. Wybo

##### Microbiology and infection control, UZ Brussel, Brussels, Belgium

###### **Correspondence:** N. Verbraeken

**Introduction:** In 2013 UZ Brussels launched the idea for the patients to see and to hug their pet during their hospital stay. But lack of legal regulation around animals visit to patients was an obstacle. Therefore it was decided to build a House (Villa Samson) next to the hospital, a ' meet and greet ' place where pets and therapy animals can come for a visit.

**Objectives:** The aim is to describe the implementation of infection prevention rules in zootherapy.

**Methods:** As the plans progressed, the need to create an infection prevention framework together with the initiators and the expertise of a vet, became clearer. In addition to exclusion criteria for patients and animals, visit rules and regulations about cleaning and disinfection were drafted and implemented.

**Results:** The staff consists of a coordinator with a nursing background and volunteers. The latter accompanies the patients to and from Villa Samson and guides the patients and animals in the house. Before entering the service and annually, they participate in a training day with instructions on hand hygiene and cleaning/disinfection.

Meanwhile, Villa Samson is open for more than 1 year. To date, no incidents have been reported. On average, there are 60 visits per month, especially from psychiatric, pediatric, geriatric and rehabilitation wards. Because of an increasingly shorter hospitalization, the practical organization of a visit to Villa Samson remains difficult. Everyone tries as much as possible to adhere to the rules but hand hygiene compliance is sometimes difficult, just as it is in the hospital.

**Conclusion:** This experiment proves that the risk remains manageable as long as the setting adheres to a procedure, such as the High Health council (HGR) in Belgium prescribes.

**Disclosure of Interest**: None declared

### P407 AUTOMATED HAND HYGIENE MONITORING INCREASES THE UNDERSTANDING OF POOR COMPLIANCE BEHAVIOUR: A PROSPECTIVE OBSERVATIONAL STUDY

#### M. B. Hansen^1^, A.-M. Iversen^2^, C. R. Lauridsen^2^, R. Hansen^3^, B. Hesselbo^4^, R. Alexander^1^, C. P. Kavalaris^4^

##### ^1^Konduto ApS, Copenhagen; ^2^Department of Oncology, Aarhus University Hospital, Aarhus; ^3^Department of Orthopedic; ^4^Department of Quality and Education, Bispebjerg University Hospital, Copenhagen, Denmark

###### **Correspondence:** M. B. Hansen

**Introduction:** Low hand hygiene compliance (HHC) at hospitals remains a problem and good monitoring methods are needed.

**Objectives:** We aimed to asses HHC and describe our experiences using a real-time automated hand hygiene monitoring system.

**Methods:** An automated HHC monitoring system (Sani nudge™)^1^ was installed at two Danish hospital wards between Feb 2018 and Sep 2018. The system constantly monitored staff hygiene behaviour using sensors located on staff name tags, patient beds and sanitizers. Specially designed algorithms based on the WHO’s ‘5 Moments for Hand Hygiene’ and local hospital guidelines were used to calculate the HHC levels. The system has been validated and compared with direct observations.

**Results:** With 79 staff members (nurses, n=73; doctors, n=6) included, the system registered 2.2 million data points equating to 127,601 direct observations and a mean HHC of 46%. There was a disparity in HHC rates across room types with the lowest HHC in patient bedrooms (mean 36%) and the highest HHC in staff toilets (mean 80%). In the patient bedrooms, the staff more often sanitized hands after (mean 35%) than before (mean 26%) patient contact. Fluctuations in HHC between days became smaller as the HCWs worked with the data from the fully integrated system and a general upward trend could be seen achieving HHC levels as high as 93% in staff toilets.

**Conclusion:** The automated hand hygiene monitoring system proved to be a successful alternative to current observation methods in supplying detailed information about HHC. The data-driven approach provided hospitals with unbiased and real-time compliance data while giving important insights into hygiene behaviour. Importantly, the system helped identifying critical areas with low HHC that was improved.

**References**

[1] Sani nudge: www.saninudge.com (accessed on 21/05/2019)

**Disclosure of Interest**: M. Hansen Other conflict with: Co-founder of the hygiene monitoring system, A.-M. Iversen: None declared, C. Lauridsen: None declared, R. Hansen: None declared, B. Hesselbo: None declared, R. Alexander Employee of: Konduto ApS, C. Kavalaris: None declared

### P408 COMPARATIVE ANALYSIS OF BACTERIOLOGICAL AND REAL TIME POLYMERASE CHAIN REACTION (RT-PCR) TESTS IN ASSESSING THE COLONIZATION OF OBJECTS OF HOSPITAL ENVIRONMENT

#### O. A. Orlova^1^, M. N. Zamyatin^1^, N. Umzunovа^1^, Y. A. Savochkina^2^, V. G. Akimkin^2^

##### ^1^Ministry of Health of the Russian Federation, Pirogov National Medical and Surgical Center; ^2^Rospotrebnadzor, Central Research Institute of Epidemiology , Moscow, Russian Federation

###### **Correspondence:** V. G. Akimkin

**Introduction:** According to bacteriological tests (BTs), the level of colonization by nosocomial microorganisms of hospital facilities is from 5 to 36%, however, these tests do not always help to determine the pathways of infection. Additional information can be obtained from RT-PCR tests, but its place in the system of epidemiological survey has not yet been determined.

**Objectives:** To conduct a comparative study of BTs and RT-PCR tests results in assessing the colonization of hospital environment objects.

**Methods:** 215 samples of washout specimens from various sources of hospital environment were processed by BTs and RT-PCR tests.

**Results:** Positive results of BTs were found in 54% of samples, RT- PCR at the same objects in 80.0%, with strong correlation (r=0.92) in content. The coincidence of the results in most cases was noted at high values of DNA copies (800-10000), and mismatch of the results at low values of DNA copies or in the presence of microbial associations. BTs revealed 129 microorganisms with a predominance of coagulase-negative staphylococci (CoNS) – 70.5%. RT- PCR discovered the DNA of 288 pathogens: CoNS – 55.6%t, Streptococcus spp. – 19.8% and Enterococcus spp. – 16.7%. The frequency of pathogens from ESCAPE group was 22.7% in the BTs and 41.3% in RT- PCR(p<0.05). The RT-PCR tests revealed genes of methicillin-resistant St.aureus 1.9 times more often (p <0,05) and found the genes of metal-β-lactamase (MBL) in 26.0% of washout samples, where the BTs did not find the MBL producers at all (p<0,05).

**Conclusion:** Although the PCR method doesn’t answer the question whether pathogens that are found on the objects of the hospital environment remain alive, but the detection of their DNA indicates that they have contaminated the surfaces for some time and could be a source of nosocomial infection of patients.

**Disclosure of Interest**: None declared

### P409 A ROADMAP FOR DEVELOPING, ADOPTING, ADAPTING AND IMPLEMENTING IPC GUIDELINES – A COUNTRY PILOT TEST

#### C. Kilpatrick, A. P. Coutinho Rehse, D. Lo Fo Wong

##### World Health Organization, Copenhagan, Denmark

###### **Correspondence:** A. P. Coutinho Rehse

**Introduction:** According to WHO, defective infection prevention and control (IPC) practices during every day health care delivery cause harm to hundreds of millions of patients worldwide every year. To achieve successful implementation of the core components (CC) of infection prevention and control (IPC) programmes, WHO is committed to assisting countries with the development and implementation of evidence-based IPC guidelines, as one key component.

**Objectives:** To determine the baseline of one country in the European Region with regards to the current level of implementation of the IPC CC at national level. To test the draft content of a planned, easy to read five-step roadmap for national development, adaption, adoption, implementation and monitoring of evidence-based IPC guidelines in this country.

**Methods:** A desk exercise was undertaken to critique the available information on the WHO five step cycle for implementation and a mapping exercise performed to highlight examples that could form the basis of a roadmap for guideline development. A baseline assessment, using the WHO National IPC Assessment Tool 2 (IPCAT2) was performed. Finally, in-country interviews were conducted and pilot testing of the draft content of the roadmap was performed.

**Results:** The desk exercise resulted in an enhanced outline of the WHO five step cycle, focused on the actions required at the outset of guideline development, to inform the draft roadmap. The pilot test country exercise identified gaps in the content but importantly the need for such a roadmap to allow for a systematic and robust decision making process that is time efficient and has an impact on practice.

**Conclusion:** Further testing of the roadmap content is planned with an additional eight countries in the European Region. A final report including recommendations for the use of the roadmap is expected early 2020, alongside the final visual roadmap. As technical guidelines should provide clear directions on IPC priorities, evidence-based standards and a framework for local adaptation, including training and monitoring of adherence, this roadmap, if adequately developed, can lead to quality improvement and enhanced outcomes. This work will build upon the WHO core components for IPC, supporting prevention of healthcare associated infections and antimicrobial resistance.

**Disclosure of Interest**: None declared

### P410 POSSIBLE EFFICACY LOSSES OF ANTIMICROBIAL SURFACE TECHNOLOGIES AFTER REPROCESSING

#### S. Buhl^1^, A. Stich^1^, J. Peter^1^, R. Brückner^2^, C. Bulitta^1^

##### ^1^Institute of Medical Engineering, Technical University of Applied Sciences Amberg-Weiden, Weiden; ^2^HECOSOL GmbH, Bamberg, Germany

###### **Correspondence:** S. Buhl

**Introduction:** Medical devices may be heavily contaminated with mircoorganisms during daily use and challenge the staff in reprocessing (cleaning and disinfection) them appropriate. Antimicrobial surface technologies could provide great advantages to reduce microbiological contamination. The principal effectiveness of these surface technologies can be demonstrated by standard methods such as the Japanese Industrial Standard (JIS Z 2801). However, there are only a few studies to date on the activity of antimicrobial technologies after reprocessing and permanent use under regular clinical conditions.

**Objectives:** In this work, various manufacturing strategies of common antimicrobial surface technologies were considered and examined regarding their potential impact on their effectiveness. A special focus has been on the possible impact of reprocessing in routine clinical setups on the functionality of the coatings.

**Methods:** Therefore, in laboratory tests, various samples of different materials (glass, plastic, metal, tiles) were antimicrobially coated and the durability of the coatings was evaluated after abrasion tests simulating routine clinical reprocessing. The assessment was done by light and electron microscopic examination of the surface structures as well as by microbiological test methods (JIS Z 2801).

**Results:** Our studies showed strong differences in the durability of the antimicrobial coating depending on the surface texture and the antimicrobially active coating substance used. While some of the coatings were still able to show an antimicrobial activity even after 500-fold wet abrasion, the coating on other test samples was already damaged after the first wiping step with distilled water.

**Conclusion:** Our results suggest that both the antimicrobial active substance itself and the underlying surface material have a strong impact on the durability of the antimicrobial surface technology. Therefore, the assessment of the interplay of these factors is a critical issue and should play an important role in the development of new products with permanent antimicrobial activity in clinical settings.

**Disclosure of Interest**: S. Buhl: None declared, A. Stich: None declared, J. Peter: None declared, R. Brückner Employee of: HECOSOL GmbH, C. Bulitta Consultant for: HECOSOL GmbH

### P411 A SYSTEMATIC LITERATURE REVIEW ON MINIMUM STANDARDS FOR INFECTION PREVENTION AND CONTROL

#### J. Hopman^1^, D. Urlings^1^, A. Twyman^2^, T. Allen^2^, A. Cassini^2^, B. Allegranzi^2^

##### ^1^Medical microbiology, Radboud University Medical Center, Nijmegen, Netherlands; ^2^Infection Prevention and Control Global Unit, World Health Organization , Geneva, Switzerland

###### **Correspondence:** J. Hopman

**Introduction:** Core components for effective infection prevention and control (IPC) programmes are defined by the World Health Organization (WHO). However, successful implementation and sustainability in low-resource settings is challenging.

**Objectives:** To identify minimum standards for IPC programmes at the national and facility level.

**Methods:** A systematic literature review was performed using CIHAHL, Pubmed, GIM and Embase databases. Articles were included if the original study identified IPC programmes or measures as being minimum standards or requirements. Interventions were categorized as either horizontal (e.g. IPC programme, education) or vertical (e.g. prevention of surgical site infections).

**Results:** A total of 7871 articles were screened by title and abstract, of which 164 were reviewed in full and 47 were finally included. Overall, included studies showed low quality (reviews, systematic reviews and before-after studies) evidence. Most studies were performed in high-income countries (40/47) and were facility-based (40/47). Horizontal IPC measures mentioned as minimum were: hand hygiene (91%), transmission-based precautions (including triage) (87%), surveillance (85%), education/training (81%), built environment/infrastructure (77%), guidelines (70%) and decontamination (70%) and monitoring/audits/feedback (66%). The most frequent vertical interventions were those to prevent catheter-related bloodstream infections (36%), ventilator-associated pneumonia (26%), and surgical site infections (21%), while among pathogen-specific ones, those related to the prevention of the spread of MRSA (32%) and Clostridium difficile (19%) were the most frequent.

**Conclusion:** Horizontal interventions were more frequently mentioned as minimum standards compared to vertical interventions. The use of multimodal and stepwise approaches is critical for their integration. This work will guide the identification of minimum requirements for IPC as the starting point to implement the WHO core components, in particular to help prioritization in low-resource settings.

**Disclosure of Interest**: None declared

### P412 COPPER-CONTAINING GLASS CERAMIC PARTICLES WITH HIGH MICROBICIDAL EFFICACY AGAINST ESKAPE PATHOGENS AND NON-ENVELOPED VIRUSES

#### J. Lahiri, A. Golas, J. Kurzejewski, B. Balakrisnan, S. Caracci, P. Novak on behalf of Corning Incorporated

##### Corning Incoporated, Painted Post, United States

###### **Correspondence:** J. Lahiri

**Introduction:** Studies suggest that continuously killing antimicrobial surfaces based on copper can reduce bioburden and lower the risk of infection [1]. Copper-containing glass ceramic particles in materials such as architectural wall paint stabilize and release Cu(I) by a slow-release mechanism imparting microbicidal properties to the coated surface [2].

**Objectives:** Evaluation of the bactericidal efficacy of coated surfaces containing copper-glass ceramic particles against ESKAPE pathogens – leading cause of nosocomial infections, and non-enveloped viruses (Adenovirus 5 and Feline Calicivirus). Comparison with currently available antimicrobial paints containing silver ions and quaternary ammonium compounds (QACs).

**Methods:** Bactericidal efficacy tests including study controls were performed as described in the US EPA’s test for efficacy of copper alloy surfaces as a sanitizer. A 20μL inoculum of test microbes was exposed to coated coupons containing copper-glass ceramic particles for 2 hours at ambient temperature and humidity (23°C; 42% RH). Following the exposure period, coupons were neutralized and assayed for survivors/infectivity. Differences in CFU/PFU counts between control and test coupons were calculated to evaluate efficacy.

**Results:** A 3 log (≥99.9%) reduction in colony counts and or reduction of bacteria down to limit of detection was observed against all six ESKAPE pathogens, Adenovirus 5, and Feline Calicivirus when exposed to coatings containing copper-glass ceramic particles. In contrast, commercially available paints with silver ions did not demonstrate ≥3 log kill when screened against *S. aureus* under real-use contamination conditions.

**Conclusion:** Copper-containing glass particles in decorative coatings and other high-touch environmental surfaces offer a pragmatic and broadly deployable solution for hospital infection control.

**References**

[1] Salgado, C. D. et al. Copper surfaces reduce the rate of healthcare-acquiredinfections in the intensive care unit. Infect. Control Hosp. Epidemiol. 34,479–486 (2013).

[2] Gross et al. Copper-containing glass ceramic with high antimicrobial efficacy. Nat Commun. 10(1),1979 (2013)

**Disclosure of Interest**: J. Lahiri Employee of: Corning Incorporated, A. Golas Employee of: Corning Incorporated, J. Kurzejewski Employee of: Corning Incorporated, B. Balakrisnan Employee of: Corning Incorporated, S. Caracci Employee of: Corning Incorporated, P. Novak Employee of: Corning Incorporated

### P413 POTENTIAL FOR IMPROVEMENT OF MEDICAL DEVICES THROUGH ANTIMICROBIAL SURFACE COATING

#### S. Buhl^1^, M. Engelmann^1^, R. Brückner^2^, C. Bulitta^1^

##### ^1^Institute of Medical Engineering, Technical University of Applied Sciences Amberg-Weiden, Weiden, ^2^HECOSOL GmbH, Bamberg, Germany

###### **Correspondence:** S. Buhl

**Introduction:** Among other factors, the successful control of nosocomial infections includes the proper planning and observance of cleaning, disinfection and sterilization measures. To which extent constructional and constructive measures of medical devices should be improved by an additional antimicrobial coating is currently the focus of various research projects.

**Objectives:** This study aimed to assess the impact of "hygienic design" and antimicrobial coating for hygienically relevant surfaces.

**Methods:** In this work an evaluation of the "hygienic design" for hygienically relevant surfaces was done with two exemplary surfaces. On the one hand with the Fresenius 4008S hemodialysis machine and on the other with different surfaces of cabinets and drawers in the preparation room of the research OR of OTH Amberg-Weiden. In a first step we evaluated the "hygienic design" of the surfaces, by applying fluorescent markers as well as artificial microbiological contamination in order to evaluate the effectiveness of cleaning and disinfection measures. In a second step we examined the impact of antimicrobial TiTANO® technology after artificial microbiological contamination.

**Results:** In our setup, nearly all surfaces could be fully decontaminated with standard C&D procedures. In general, it turned out that most of the examined objects essentially fulfill the requirements of an easy to clean "hygienic design". Only a few constructive elements (covers, edges), have been detected which are difficult to clean and disinfect. Additional antimicrobial coating leads to an overall reduction of the microbiological contamination.

**Conclusion:** “Hygienic design” is an important factor for effective cleaning and disinfection of hygienically relevant surfaces. An antimicrobial coating may contribute to a reduction of the hazard potential. Based on the results, antimicrobial functionality may be considered as a supporting measure in connection to intra-procedural hygiene cleaning and disinfection protocols.

**Disclosure of Interest**: S. Buhl: None declared, M. Engelmann: None declared, R. Brückner Employee of: HECOSOL GmbH, C. Bulitta Consultant for: HECOSOL GmbH

### P414 INFECTIOUS RISK ASSESSMENT VISIT IN A LIVER TRANSPLANT UNIT IN A UNIVERSITY HOSPITAL

#### E. Charpy^1^, C. Elias^1,2^, B. Cracco^1^, M. Pérard^1^, B. Grisi^1^, P. Vanhems^3,4^, C. Dananché^1,4^

##### ^1^Unité d'Hygiène, Epidémiologie, Infectiovigilance et Prévention, Groupement Hospitalier Nord, Hospices Civils de Lyon; ^2^Université Claude Bernard Lyon 1 (UCBL1); ^3^Unité d'Hygiène, Epidémiologie, Infectiovigilance et Prévention, Groupement Hospitalier Centre, Hospices Civils de Lyon; ^4^Laboratoire des Pathogènes Émergents – Fondation Mérieux, Équipe Épidémiologie et Santé Internationale, Centre International de Recherche en Infectiologie, Institut National de la Santé et de la Recherche Médicale U1111, CNRS Unité Mixte de Recherche 5308, École Nationale Supérieure de Lyon, Université Claude Bernard Lyon 1 (UCBL1), Lyon, France

###### **Correspondence:** C. Elias

**Introduction:** Liver transplant unit hosts patients at high risk of infection due to their comorbidities and immunocompromised status. Recurrence of extensively resistant bacteria and *Clostridium difficile* outbreaks in a liver transplant unit in a university hospital urged for an infectious risk assessment visit.

**Objectives:** This was the opportunity to experiment an a priori assessment risk method with the aim to highlight critical points in infection prevention and control in order to implement corrective measures accordingly.

**Methods:** Data were collected via three pre-tested questionnaires, in which professional practices, maintenance of the premises and knowledge of standard and complementary infection control precautions among health care workers through face to face interviews were explored. The risk assessment visit took place during one full day where medical, surgical and nurse wards rounds were observed, in addition to maintenance of the premises. A qualitative data analysis was performed.

**Results:** A total of 48 questionnaires were collected, highlighting a lack of hand hygiene, a non-compliant glove use (either lack or excess) and an improper chronology when removing personal protective equipments. Among the 14 questionnaires gathered on the maintenance of the premises, none of them reported an expiry date on hand hygiene solutions or the use of an airless solution. In addition, insufficient time was dedicated to the maintenance of premises. In addition, 13 face-to-face interviews were administered to four medical personnel and nine nurses and care-givers, showing a lack of hands-on training in infection prevention and control, a limited professional experience among paramedical staff, a lack of knowledge in complementary precautions and some issues in sharing the patient’s infectious status during transfer.

**Conclusion:** Various critical points were described, encouraging the implementation of corrective and sustainable actions and a meticulous assessment of professional practices to adapt infection control measures and prevent the reemergence of future multidrug resistant organisms’ outbreaks in liver transplant units.

**Disclosure of Interest**: None declared

### P415 A SPECIFIC OUTBREAK SUPPORT TEAM IN BELGIUM

#### E. Miller, H. De Pauw

##### Outbreak Support Team & Healthcare-associated Infections and Antimicrobial Resistance, Sciensano, Brussels, Belgium

###### **Correspondence:** E. Miller

**Introduction:** The Outbreak Support Team, legally mandated by Royal Decree since 2013, provides specific support to healthcare settings during the management of outbreaks with involvement of multidrug resistant organisms (MDRO) and other nosocomial bacteria in Belgium. An overview of the number of healthcare settings and agents involved is presented here.

**Objectives:** Evaluation of the support provided by the Outbreak Support Team between December 2014 and November 2018.

**Methods:** Operational from 2014 onwards, the Outbreak Support Team (OST) is composed of the departments of infection control of the Belgian Federated Entities, the scientists of Sciensano (Belgian Public Health Institute), the experts of the dedicated National Reference Centers and other experts when appropriate.

The support to the healthcare settings consist of gathering and validation of the data, analysis of hospital’s indicators (MDRO surveillances, hand hygiene, antibiotic use, infection control infrastructure, work load), scientific advice, prioritization and evaluation of measures, mediation between actors, visits and sampling on the field, and if needed, specific control measures (cohorting, closing of wards).

**Results:** Between December 2014 and November 2018, the OST received 33 requests for support. As the assistance for OST support is not compulsory in Belgium, the number of reported outbreaks is not representative for the countrywide situation related to MDRO and nosocomial infections.

The incriminated bacteria (n) were as follows: vancomycin resistant *Enterococcus* (10); community acquired methicillin resistant *Staphylococcus aureus* (4); carbapenemase producing enterobacteriacae (11); *Pseudomonas aeruginosa* (2); *Streptococcus pyogenes* (1); Extended spectrum beta-lactamases (3); *Staphylococcus capitis* (1).

The OST intervention helped to manage and control these outbreaks. The vast majority of healthcare settings mentioned a clear added value during the outbreak management. Most of them are now considered resolved.

**Conclusion:** Thirty-three healthcare settings in Belgium (> 30%) have experienced benefit of the OST support between December 2014 and November 2018, with resolution in the majority of the cases.

**Disclosure of Interest**: None declared

### P416 HOW TO DISPLAY RESULTS FOR RARE EVENTS USE OF G CHART FOR INFECTION SURVEILLANCE RESULTS

#### M. Walberg, E. Brustad

##### Infection control physician, Vestre Viken, Baerum hospital, Drammen, Norway

###### **Correspondence:** M. Walberg

**Introduction:** Serious (deep, organ/space) surgical site infections (SSI) are rare events, and such results should be easy to understand for leaders who continually expect improved display of surveillance results. In accordance, we continually optimize our diagrams, and our latest hit is to include “number of surgeries between events” (g chart) in our varied menu of charts.

**Objectives:** Our work shows the usefulness of g chart.

**Methods:** In Norwegian hospitals SSI surveillance is mandatory and based upon criteria from European Centre for Disease Prevention and Control (chosen surgeries only); post discharge patients are also included. Infections control departments are responsible for data collection and for reporting results to National Institute of Health. Our work was based on data from Vestre Viken, a Norwegian trust of four hospitals with 250.000 annual bed days.

**Results:** In order to be able to reveal clustering of rare events, we have decided to introduce use of g chart. This tool gives leaders vital support to trace development vs time and is essential for their engagement in improvement work and patient safety. In our g charts we have chosen to use a delayed moving window estimate of the mean based on 30 observations and a delay of 10. Previously, our menu for displaying surveillance results (standardized national and locally developed graphs) included tabular data, monthly moving average, monthly incidence rate or benchmark data only.

**Conclusion:** G chart is useful for displaying surveillance results for rare events like SSIs as this method can reveal process out of control at an early stage. This tool is powerful for hospital leaders who are engaged in improving patient care. Infection control staff should implement g chart in their display of surveillance results of rare events.

**References**

Walberg M et al. Inf Contr Hosp Epidemiol 2008;29:635-41

**Disclosure of Interest**: None declared

### P417 GUIDELINE VDI 5706 "CLASSIFICATION AND DESIGN OF HYGIENICALLY RELEVANT AREAS IN CLINICAL SETTINGS"

#### C. Bulitta^1,2,^ on behalf of VDI 5706 "classification and design of hygienically relevant areas" on behalf of VDI FA Guideline VDI 5706

##### ^1^Technical University of Applied Sciences Amberg-Weiden, Weiden, ^2^VDI, Düsseldorf, Germany

###### **Correspondence:** C. Bulitta

**Introduction:** Cleaning and disinfection (C&D) of high touch and therefore hygienically relevant surfaces is an important element in infection control. For many hygienically relevant areas in the clinical environment there are no normative or standardized procedures for reprocessing defined. A risk management based classification may help to optimize product design and reprocessing procedures and therefore could contribute to reduce infection risks.

**Objectives:** The aim is to create a VDI guideline that standardizes the risk management of hygienically relevant surfaces and the "hygienic design" of non-critical medical devices and other patient near objects.

**Methods:** Search results of international literature and regulations on classification, risk management and the design of hygienically relevant surfaces and objects in patient care and other industries (e.g. food industry) was performed, reviewed and assessed by a panel of experts from manufacturers, scientists and infection control professionals (including VAH and RKI). Classification methods and guidelines for hygienic design were derived or adapted for medical devices and clinically relevant surfaces.

**Results:** The following key criteria for the risk assessment were identified: location of the area, conditions and frequency of surface contact, endangered vs. endangering persons, surface composition and type of contamination. Other criteria are described as potentially influencing factors. Based on these criteria, a risk management process similar to DIN EN ISO 14971 is defined. Taking into account functional aspects, recommendations for design of hygienically relevant surfaces of non-medical products as well as non-critical medical devices is derived.

**Conclusion:** This guideline shall lead to hygienically optimized, robust and efficient products and solutions.

**Disclosure of Interest**: None declared

### P418 AN INNOVATED SYSTEM IDENTIFIES THE 5 MOMENTS AND HCWS TO END HAND-HYGIENE NON-COMPLIANCE: DECADES OF NON-COMPLIANCE IS A FAILURE OF THE SYSTEM, NOT OF HCW.

#### R. Govindarajan

##### Operations Management, Innovation and Data Sciences, ESADE Business School, Barcelona, Spain

**Introduction:** For four decades the hand-hygiene compliance level is below 50%. This historically stable non-compliance levels tell us that things won´t get any better on their own.

**Objectives: Better system is needed to create the missing habit**

There are five systemic issues that may make compliance difficult for employees:

- When employees want to sanitize, they may not always find the sanitizer flask around.
- Even when a sanitizer flask is found when needed, it may be empty.
- The sanitizer gel quality could be another issue.
- The amount of gel dispensed is insufficient for correct disinfection.
- There is no consequence for non-compliance for employees but there is for patients.

In the presence of unresolved systemic issues and in the absence of a control system, employees cannot be blamed.

**Methods:** Govisystem™ is an innovation that puts the burden on the system, not on its users. Through this system, supervisors can observe from their desk and know in real time which employee obtained gel, which of the five hygiene moments was practiced, at which dispenser location (which patient room), at what time, how many times and at what intervals, given the clinical task assigned to that person.

**Results:** Govisystem™ with its **LAVAGE** formula facilitates complainace.

▫▫ **Location**: Non-portable gel dispensers are placed at the point of use.

▫▫ **Availability**: Gel availability in all dispensers is monitored in real-time on the computer screen.

▫▫ **Validity**: The validity of the gel is guaranteed through quality controls.

▫▫ **Amount**: Gel dispensation amount is personalized for each user´s hand size.

▫▫ **Guidance**: Individual performance targets are set to comply with the WHO protocol.

▫▫ **Evaluations**: Individual evaluations and follow up are done.

**Conclusion:** With this improved system design, hand hygiene compliance can be achieved and Hospital Acquiered Infections (HAI) can be reduced, because we are able to identify each of the 5 hand-hygiene moments and each HCW so a follow-up can be done to help create the missing habit.

**Disclosure of Interest**: None declared

### P419 A UNIVERSAL PHLEBOTOMY PRACTICE GUIDELINE: INCORPORATING MY FIVE MOMENTS FOR HAND HYGIENE

#### S. Jain^1^, M.-L. McLaws^2^

##### ^1^Healthcare Associated Infections, Clinical Excellence Commission, Haymarket; ^2^School of Public Health and Community Medicine, University of New South Wales, Kensington, Australia

###### **Correspondence:** S. Jain

**Introduction:** World Health Organization (WHO) Guidelines on *drawing blood: best practices in phlebotomy* (1) was developed in 2010 incorporated two hand hygiene moments, 1 and 4, from the WHO *My Five Moments for Hand Hygiene* (2). A revision of the Guidelines (1) can be revised to reduce glove use with increased hand hygiene.

**Objectives:** To contemporises the Guidelines (1) by incorporating *My Five Moments for Hand Hygiene* for glove use by phlebotomists.

**Methods:** The *WHO Guidelines on drawing blood: Best Practices in Phlebotomy* (1) 16 steps for inpatients and outpatients was revised to reduce glove use and include additional hand hygiene moments.

**Results:** Hand hygiene was included in the steps prior to preparing phlebotomy related items to maintain the cleanliness of collection trolley and other phlebotomy accessories. Further changes include hand hygiene immediately before glove donning in accordance with moment 2. Immediately after phlebotomy revision provided a new step, removal of potentially contaminated gloves followed by hand hygiene, moment 3, to avoid contamination of the collection trolley and patient zone. These revised steps allow the phlebotomists to clean reusable items such as tourniquet and collection tray to prevent transmission of blood borne viruses and other microorganisms from patient to patient zone.

**Conclusion:** This revision includes *My Five Moments for hand Hygiene* (2) practices that explicitly incorporates hand hygiene for glove use. The revised steps have reduced the number of glove changes, the potential for glove contamination and contamination of the patient zone from used gloves for inpatients and outpatients. s. The revised steps would benefit from further validation among different phlebotomy settings.

**References**

1. World Health Organization. WHO guidelines on drawing blood: best practices in phlebotomy Geneva: The World Health Organization; 2010 http://www.euro.who.int/__data/assets/pdf_file/0005/268790/WHO-guidelines-on-drawing-blood-best-practices-in-phlebotomy-Eng.pdf?ua-1 Last accessed on May 19, 2019

2. World Health Organisation. Guidelines on Hand Hygiene in Health Care-First Global Patient Safety Challenge Clean Care is Safer Care Geneva: The World Health Organization; 2009, http://www.who.int/gpsc/5may/tools/who_guidelines-handhygiene_summary.pdf Last accessed on May 19. 2019

**Disclosure of Interest**: None declared

### P420 MONITORING THE HOSPITAL ENVIRONMENT: HOW A NOVEL SOFTWARE-BASED SYSTEM SUPPORTS IMPROVEMENT PLANS AT ST. CONSTANTIN HOSPITAL, ROMANIA

#### A. Moldovan^1^, S. Singerozan ^2^

##### ^1^Infectious Diseases and Infection Prevention and Control , St.Constantin Hospital , Brasov; ^2^Infection Prevention and Control , Emergency County Hospital, Miercurea Ciuc , Romania

###### **Correspondence:** A. Moldovan

**Introduction:** 2013 Award Winner St. Constantin has a vision to go above and beyond Hand Hygiene Excellence by including the hospital environment in its Hygiene Improvement Plans.

Romanian legislation relevant for Cleaning and Disinfection in Hospitals (Ordinul 961/2016) requires a mandatory monitoring program of to determine the hygiene status of defined environmental compartments.

**Objectives:** To support legislative compliance and to drive improvement plans, St. Constantin Hospital was looking for solutions to manage their monitoring program effectively to generate data for immediate interventions or to observe trends that could be addressed in improvement plans.

**Methods:** A software-based environmental monitoring system was tested in a pilot trial to assess the suitability of the system. It was also used to expand the program for internal quality control purposes.

The monitoring methods used were:

- Settle plates

- Water testing

- Sterility testing

- Surface and hand swabs

- Dip slides

- UV Fluorescence

**Results:** Use of a software-based tool allowed the hospital to

- map the facility, define all sampling points and allocate risk classes

- plan sampling activities in a structured way with respect to sites, samples to take, methods, sampling intervals

- capture all data in one place, allowing immediate alerts upon exceeding any limits as well as trending and reporting

The system resulted in a time saving of 25% compared to manual sampling, as well as gaining full commitment of the team involved.

173 samples were investigated between December 2018 and March 2019.

7 sterility samples exceeded the limits, resulting in a repetition of tests and additional controls of the sterilization process (equipment and personnel) and re-training of staff.

A few dip slide samples were above limit triggering a new instruction of medical and cleaning staff.

**Conclusion:** Introduction of a software-based system helped to manage mandatory monitoring activities in a more structured way, saved time and allowed additional testing in specific critical areas.

All air and water samples, surface and hand swabs were within their limits. For sterility and dip slides samples corrective actions were taken. Thus a very good hygiene status was confirmed.

**Disclosure of Interest**: None declared

### P421 IMPLEMENTATION OF INFECTIOUS RISK ASSESSMENT AT ACUTE CARE PATIENT ADMISSION

#### F. Vieira, I. Devesa, D. Peres, I. Neves

##### Infection Control and Antimicrobial Resistance Unit, Matosinhos Local Health Unit, Matosinhos, Portugal

###### **Correspondence:** F. Vieira

**Introduction:** Infection Control Standard Precautions (ICSP) should be applied by all healthcare professionals to all patients. "Patient placement" is part of the ICSP according infectious risk to prevent cross-transmission. The Portuguese Health Directorate recommends"at patient admission the infectious risk should be evaluated, guiding the decision on patient placement and/or isolation." The CDC recommends to implement systems for early detection/management of infectious persons, at admission”.

**Objectives:** (1) infectious risk assessment (IRA) strategy implementation at admission to an acute care hospital; (2) execution of specific infection control measures(isolation and/or active screening) in a selected population.

**Methods:** (1) risk factors definition based on bibliography and institutional guidelines for specific microorganisms control, (2) IRA grid construction/pilot testing; (3) IRA guideline; (4) grid digital integration in Electronic Patient Record (EPR); (5) clinicians training to use the grid, (6) grid definitive implementation applied to all patients.

**Results:** We considered the following risk factors: patients from other health institutions/nursing homes; from countries with high prevalence of Carbapenemase-Producing *Enterobacteriaceae* (CPE); last 6 months history of admission and/or exposure to antibiotics; presence of invasive devices/chronic wounds; patient on haemodialysis program; presence of diarrhoea/cough;pulmonary tuberculosis suggestive symptoms; multidrug-resistant microorganisms in the last 12 months; patient in “comfort measures”. Grid available in EPR, with "yes-no" response. When validating the grid, the clinician receives an automated response regarding the measures to be implemented (ICSP or isolation and/or active screening for EPC or MRSA).

**Conclusion:** The implementation of an IRA at patient admission allows, if necessary, specific infection control measures in order to minimize cross-transmission at hospital. Its inclusion in the network, with automated clinical decision responses, facilitates the process and minimizes errors. In the future, the recorded information will allow to evaluate the cost-effectiveness of this strategy.

**Disclosure of Interest**: None declared

### P422 CARBAPENEM-RESISTANT ENTEROBACTERIACEAE (CRE): PATIENT AND INFORMATION FLOWS THROUGH DIFFERENT LEVELS OF HEALTHCARE

#### I. Devesa, D. Peres, F. Vieira, I. Neves

##### Infection Control and Antimicrobial Resistance Unit, Matosinhos Local Health Unit, Matosinhos, Portugal

###### **Correspondence:** I. Devesa

**Introduction:** Hospital discharge paradigm has changed: today its approach integrates the different levels of care in the health system. In a well-developed and competent structure (primary and long term-care), hospital discharges occur earlier. Nowadays, epidemiological and demographic variations increase the risk of disseminating multidrug resistant organisms among health care levels.

**Objectives:** To describe a local surveillance system directed to patients CRE colonized/infected in our facility (374 bed acute care hospital and primary health care centre).

**Methods:** Laboratory-based surveillance system, from the microbiology laboratory results and automatic email alert messages to Infection Control and Antimicrobial Resistance Unit (ICARU). At ICARU the CRE case is investigated and verified infection control measures and information management: hospitalized patient, isolation precautions and direct contacts active screening; outpatient, the family doctor and nurse are informed by email; nursing home patient, information by phone; patient transfer to other healthcare facility, its ICARU is informed by phone/email. Inform to National Reference Laboratory by facility microbiology laboratory and local ICARU informs regional ICARU.

**Results:** From 1^st^ January 2018 to 22^nd^ May 2019: 144 patients CRE infected/colonized were discharged from hospital; mean age 76 years; 107 enrolled in our primary care and 37 to other facility. 39 patients (27%) were readmitted more than two times.

**Conclusion:** A laboratory-based surveillance system and automatic alert messages, directed to CRE patients, allows to implement adequate infection control measures and monitor patient flow. This patients have high admission rate, so sharing real time information with other local, regional and national institutions, can reduce the spread of microbial resistance. In the future, we expected that full automatic surveillance system help ICARU give fast response using less human resources.

**Disclosure of Interest**: None declared

## Poster session: Innovative technologies and concepts in hand hygiene

### P423 MULTI-DRUG RESISTANT BACTERIA RECOVERED FROM HANDS OF HEALTH CARE WORKERS AT A TERTIARY HOSPITAL IN KAMPALA UGANDA

#### P. Katongole, D. Bulwadda, H. B. Kyobe, F. Bwanga, F. C. Najjuka

##### Department of Medical Microbiology, Makerere University College of Health sciences, KAMPALA, Uganda

###### **Correspondence:** P. Katongole

**Background:** Control and prevention of Hospital Acquired Infections is a major challenge to modern medicine. These infections are usually caused by Multi-Drug resistant bacteria and are mostly transmitted by hands of Health Care Workers. Hand hygiene adherence among health care workers is less practiced especially in developing countries. In this study, we investigated the bacterial hand carriage by different health care cadres, commonest pathogenic species and there antimicrobial susceptibility patterns at Naguru hospital in Kampala, Uganda.

**Methods:** All health care workers at Naguru Hospital who met the inclusion criteria were included in the study. Hand swabs were done following standard procedures and samples transported within 2 hours to the laboratory for processing. Positive cultures were followed up, identified and antimicrobial susceptibility patterns done using standard operating procedures.

**Results:** Out of 108 health care workers who participated in the study, 68(63%) were females. Proportion microbial carriage per professional category were highest among nurses 16 (34.8%), Clinical officers 6 (13%), Medical officers 11(23.9%), cleaners 4(8.7%) and others 9(19.6%). Among the wards, surgical and emergency wards had the highest prevalence of pathogenic microbial carriage, 36% and 35.6% respectively. Among the isolated pathogenic bacteria, 25(36.2%) were gram positive whereas 44(63.8%) were gram negatives. Staphylococcus aureus was the most predominant organism isolated, 29% followed by *E.coli, 21.7*%. 50% of *Staphylococcus aureus* were methicillin resistant and one isolate was vancomycin resistant. 66.7% of the *E.coli* isolates were ESBL positive.

**Conclusion:** This study demonstrated that hands of all health care workers, with varying prevalence in Naguru Hospital were colonized with different pathogenic bacteria, some with multidrug resistant forms including MRSA and ESBL. We recommend stringent infection prevention and control measures in this setting.

**Disclosure of Interest**: None declared

### P424 FOMITE-TO-FINGERTIP TRANSFER OF ESCHERICHIA COLI DURING SEQUENTIAL SURFACE CONTACTS WITH AND WITHOUT HOSPITAL NITRILE GLOVES: A MODELLING AND EXPERIMENTAL STUDY

#### M.-F. King^1^, K. Atedoghu^2^, M. López-García^2^, W. Hiwar^3^, N. Zhang^4^, S. J. Dancer^5,6^, C. J. Noakes^1^, L. A. Fletcher^1^

##### ^1^School of Civil Engineering; ^2^UNIVERSITY OF LEEDS, Leeds, United Kingdom; ^3^School of Mathematics, UNIVERSITY OF LEEDS, Leeds, United Kingdom; ^4^Department of Mechanical Engineering, University of Hong Kong, Hong Kong, Hong Kong; ^5^School of Applied Sciences, Edinburgh Napier University, Edinburgh; ^6^Department of Microbiology, Hairmyres Hospital, NHS Lanarkshire, United Kingdom

###### **Correspondence:** M.-F. King

**Introduction:** The transmission of microorganisms from contaminated surfaces to fingers is known to contribute to hospital acquired infections, but the transfer of microorganisms to fingers after multiple sequential surface contacts with and without hospital nitrile gloves remains unexplored.

**Objectives:** To assess the effect of nitrile gloves on the viable concentration of *Escherichia coli* on fingertips after repeated contacts with inoculated plastic surfaces experimentally and mathematically.

**Methods:** Coupons of laminate plastic, were inoculated with *E. coli*, allowed to dry and 35 participants touched these sequentially 8 times using either a bare or nitrile gloved finger. Fingers were swabbed, and colonies cultured for enumeration. A linear mixed effects model was used to examine the effect of gloves as well parameters involved in sampling. An Approximate Bayesian Computation (ABC) method is used to estimate transfer efficiency for a single contact from the data to compare against distributions from literature.

**Results:** Transfer was dependent on finger microbial loading and showed an oscillatory effect becoming negative after 5 contacts. Transfer efficiency for a single contact estimated using ABC, was higher with bare skin (69%, 95% CI=43-94%) than gloved hands (37%, CI=23-51%). Microbial load became statistically stable after four contacts for gloved hands and after six contacts with bare skin. Analysis through Oldham’s method suggests that the initial amount transferred during the first contact only has a significant effect on finger loadings up to the fourth contact, after which there is no discernible effect. Use of gloves has a dominant effect over an individual’s finger-to-fomite pressure and finger surface area (*p*<0.01).

**Conclusion:** On average, gloves reduced loading by 4.7% (CI=-12%>21%) over un-gloved contacts. Highest stochasticity of loading is observed from the first to the second contact, leading to the conclusion that contact with a single contaminated surface may be more important than repeated contacts with equally contaminated surfaces.

**Disclosure of Interest**: None declared

### P425 PREVENTION OF CROSS-TRANSMISSION AFTER TOUCHING A PATIENT (WHO MOMENT 4) USING A SIMPLIFIED METHOD FOR HAND HYGIENE

#### H. Soule, D. Pires, J. Sauser, C. Fankhauser, M. Abbas, D. Pittet

##### Infection Control Programme and WHO Collaborating Centre on Patient Safety, Geneva University Hospitals, Geneva 4, Switzerland

###### **Correspondence:** H. Soule

**Introduction:** In previous studies, we have shown that a simplified method for hand hygiene (HH; 15 sec rubbing with a hand size-adjusted volume of alcohol and fingertips first followed by the rest of the hands) can lead to a reduction of approximately 2 log_10_ on hands artificially contaminated with either *Escherichia coli* or *Staphylococcus aureus*.

**Objectives:** We evaluated the efficacy of this simplified HH method in a laboratory experiment simulating WHO HH moment 4 (after touching patient).

**Methods:** Ten pairs of physicians were enrolled in the study. Each one played the role of the physician or the “patient”. The test was performed on 2 separate days. On day 1, after handwashing with soft soap, each physician rubbed all fingertips of both hands in tryptone soy broth in order to estimate their resident flora. They then performed a standardized medical examination of the patient for 4 minutes, after which their fingertips were sampled to measure the quantity of transient flora collected after touching the “patient’s” skin. On day 2, the experiment was repeated but HH with isopropanol 60% (v/v) according to our simplified method was performed prior to the second sampling. Three dilutions of each sample were inoculated on agar for 48 hours. A linear mixed model with treatment as covariate and subject-specific random effect was used. A log-transformation was applied to meet normality of the residuals. Estimated treatment effect was interpreted as a ratio of geometric means. The same approach was used for estimating the ratio in the group without HH.

**Results:** Without HH, geometric means of the amount of bacteria collected from the fingertips of the 20 doctors before and after touching the patient were respectively 5,527 cfu and 46,501 cfu (ratio = 8.4; 95% CI 3.96-17.84). When hands were rubbed with alcohol after touching the patient, the estimated number of bacteria on fingertips after medical examination was 93% lower than without HH action (p < 0.001).

**Conclusion:** When hands were rubbed with alcohol using the above-described simplified method after touching the patient, the number of bacteria on the physicians’ fingertips was quite similar to the number before medical examination, suggesting efficacy in reducing the transient flora.

**Disclosure of Interest**: None declared

### P426 MICROBIOLOGY-BASED RELIABILITY ASSESSMENT OF THE FLUORESCENT METHOD

#### V. Sari^1^, S. Bansaghi^2^, A. Lehotsky^3^, T. Haidegger^4^

##### ^1^Faculty of Chemical Technology and Biotechnology, Budapest University of Technology and Economics; ^2^Depatrment for Epidemiology, Semmelweis University; ^3^National Institute of Oncology; ^4^University Research, Innovation and Service Center , Óbuda University, Budapest, Hungary

###### **Correspondence:** S. Bansaghi

**Introduction:** Effectiveness of an act of hand hygiene is regularly evaluated by the fluorescent method. This contains performing hand hygiene with a handrub containing a fluorescent marker, then usually human experts evaluating the hands under UV light, and deciding whether the applied handrub covered the whole hand surface, based on the intensity of UV-dye.

**Objectives:** As the UV-dye generates a color-gradient on the hand after application, there is no direct mapping between the color intensity and the effective handrub amount. The aim of this study was to investigate how differently experts judge the same coverage, and compare that to microbiology for validation.

**Methods:** Hands of volunteers were contaminated with high concentration of apathogenetic *Staphylococcus epidermidis* suspension, than incompletely disinfected with UV-labeled handrub. Four different devices (Schülke Optics UV, Dermalite Check-Box UV, Stery-Hand and Semmelweis Scanner) were used to take pictures of the given hands under UV light. Next, hands were pressed to a special, hand-size agar plate. Plates were incubated for 24 hours at 37°C. Size of inadequately disinfected areas on the hands were determined in two different ways. First, based on microbiology; an expert evaluated the areas, where colonies were grown. Second, four independent senior infection control specialists were asked to mark the “missed” areas on UV pictures.

**Results:** 8 hands of volunteers were examined. From each hand, images were recorded with all the four devices. Each expert evaluated every image; thus 128 results were compared. Expert evaluations were highly inconsistent. The biggest difference between the human assessments was found in the case of hand #6, where properly covered hand surface spanned between 21.6% and 61.1%. Microbiology results were weakly correlated with the mean values of expert evaluations; in half of the cases, there were more than 10% difference.

**Conclusion:** Considering the result of the expert evaluations, variability was disconcertingly high. Software based assessment, microbiology cultivation and other objective methods should be employed, since evaluating the fluorescent method is challenging, even for highly experienced professionals.

**Disclosure of Interest**: V. Sari Employee of: HandinScan Zrt, manufaturer of the Semmelweis Scanner, S. Bansaghi Employee of: HandinScan Zrt., A. Lehotsky Shareholder of: HandinScan Zrt., T. Haidegger Shareholder of: HandinScan Zrt.

### P427 IMPLEMENTATION OF AN AUTOMATED MONITORING SYSTEM IN TWO DIFFERENT WARDS OF A HOSPITAL

#### A. K. Witte, M. Grohmann

##### HTK Hygiene Technologie Kompetenzzentrum GmbH, Bamberg, Germany

###### **Correspondence:** A. K. Witte

**Introduction:** Proper hand hygiene remains the most important factor for the reduction of nosocomial infections and therefore, besides absolute consumption of disinfectants and direct compliance observations, several automated monitoring systems for more detailed consumption of disinfectants have been invented.

**Objectives:** In this study, we aimed to explore the personnel’s self-perception and to investigate the implementation of such a system in two different wards of a hospital.

**Methods:** Two wards were equipped with an automated monitoring system detecting each disinfection process in detail with the possibility to distinguish between occupational groups. A transponder worn by every participant transmits any information to a database for analysis. Prior to the implementation, the personnel’s self-evaluation of disinfection performance was surveyed with a questionnaire.

**Results:** Before starting the project, consultations with the works council, the installation company, in-house IT and hygiene department were indispensable. We aimed to address and apprise the majority off all affected employees before the installation. Therefore, we informed all leading positions in personal communication within small groups. Furthermore, all employees were invited to introductory events on each ward. Within this context, the survey dealing with the behaviour of hand hygiene was introduced. After activating the monitoring system, the wards were reminded repeatedly and performance summaries were communicated regularly. The survey showed that employees feel well informed about hygiene in the respective hospital and that the amount of disinfectants dispensers is mostly sufficient. However, due to time and personnel shortage, hand hygiene compliance is negatively impacted.

**Conclusion:** Although the systematic and organized implementation of an automated disinfection monitoring system is mandatory and time consuming, such a system offers more detailed information about the actual disinfectant consumption concerning dispensers, during outbreaks and distribution among the days and occupational groups.

**Disclosure of Interest**: A. Witte Other conflict with: HTK is an independent company collaborating with both GWA and Sozialstiftung Bamberg without any financial interest or support. , M. Grohmann Other conflict with: HTK is an independent company collaborating with both GWA and Sozialstiftung Bamberg without any financial interest or support.

### P428 COMPARISON BETWEEN CONVENTIONAL DIRECT OBSERVATION AND WEB CAMERA FOR WHO HAND HYGIENE COMPLIANCE RATE IN TERTIARY HEALTHCARE CENTER

#### N. A. Bouafia^1^, W. A. Mazi^1^, S. H. Alwagdani^1^, R. I. Abu-Taha^2^, M. Dawoud^2^, K. AlKatiri^2^, A. Dahlawi^3^, M. R. ALYAMI^4^

##### ^1^INFECTION PREVENTION AND CONTROL; ^2^NEONATAL INTENSIVE CARE UNIT, KING FAISAL MEDICAL COMPLEX; ^3^MEDICAL DIRECTOR, MATERNITY TOWER -KING FAISAL MEDICAL COMPLEX; ^4^HOSPITAL DIRECTOR, KING FAISAL MEDICAL COMPLEX, TAIF, Saudi Arabia

###### **Correspondence:** N. A. Bouafia

**Introduction:** Hand hygiene (HH) is the single most important factor in preventing hospital-associated infections. Evidence is in growing that video monitoring with real-time feedback increases hand hygiene compliance rates.

**Objectives:** To compare between conventional direct observation and web camera methods in HH compliance rate.

**Methods:** Pilot comparative study 400 opportunities in monitoring HH compliance among healthcare workers (HCW) in Neonatal Intensive care unit (92-bed capacity) and Nursery department (60 – bed capacity) was conducted during January -April 2019 at King Faisal Medical Complex, Taif, Saudi Arabia. The first method was the conventional one based on direct observation by trained infection control nurse using WHO observation. The second method is based on registered video from the camera installed in these departments. These videos were relayed to infection control office via the hospital’s intranet to be examined on the real time or to be saved on a server for later observation. Patient to nurse ratio in both clinical departments was maintained with no special awareness activities for HH during the two periods. Patients’ and healthcare workers’ rights were preserved.

**Results:** HH compliance pooled mean rate was dropped from 89.8 % to 82.8 in NICU (p=0. 89) and from 83.8 to 60% (*p*= 0.009) in Nursery department based on conventional observation and web camera, respectively.

There was increasing in HH compliance rate before patient contact (88.3% *vs* 93.6%, *p* 0.26) in NICU and (48.9% *vs* 69.3%, *p* 0.04) in Nursery department with video monitoring and real-time feedback. Contact with patient surrounding was 69% in NICU and 61% in Nursery.

**Conclusion:** Monitoring with a web camera assumed accurate and benefit to HH compliance rate compared with conventional observation method. Validation is required to prove the study.

**Disclosure of Interest**: None declared

### P429 BETTER FEEDBACK FOR HAND HYGIENE WITH AN INNOVATIVE TECHNICAL SUPPORT SYSTEM REDUCE OF HEALTHCARE-ASSOCIATED INFECTIONS (HAI)

#### P. Brass, A. Waldhaus

##### Infection Prevention , Helios Klinikum Krefeld, Krefeld , Germany

###### **Correspondence:** P. Brass

**Introduction:** HCAIs prevalence is 14.8% for ICU patients and lead to increased patient morbidity and mortality. Better hand hygiene (HH) prevents a major part of HAIs. Although the importance of HH in preventing HCAIs is clear, HH compliance is generally low (_~_30%).

**Objectives:** HKK generally has best practices regarding HH, e.g. Aktion Saubere Hände gold certificate. However, additional innovative measures were taken to ensure and further improve high HH compliance.

**Methods:** In three ICUs, the existing hygiene bundles were supplemented by a technical support system, provided by HyHelp AG, Frankfurt. Caretakers attach a wearable device to their breast that detects hand disinfections (HD) by means of a gas sensor. The devices then show a “green light for the patient” and give positive, reinforcing feedback for the caretaker. A display on the device enables comparison of the individual HD count with the ward HD count. Anonymous ward statistics are displayed on a wall mounted display enabling team dynamics and continuous improvement in the team. HD reminder configurations, reporting and email alert functionality add options to foster internal HH communication. The additional feedback system was integrated in the overall communication bundle for HH and introduced in a stepwise process.

Before and within 9 months after intervention (14.364 patient days in the intervention period), there were measurements of

1.

Alcohol Based Hand Rub consumption per patient day (ABHR/PD)

2.

Compliance by direct observation

3. Amount of HAI according to the Helios iNOK system (most important multidrug resistant and infectiologically relevant pathogens).

**Results:** 1. ABHR/PD increased by 35% (120 to 160 ml/PD)

2. Compliance increased by 15% (45% to 60%). User feedback of HCW was positive, self-reported increase of quality of hand disinfection.

3. The average HAI-rate decreased by 49% or 25 multidrug resistant HAI cases within 9 months after intervention (51 to 26).

**Conclusion:** Permanent, additional individual and collective feedback by an electronic device can contribute to a sustainable increase in HH compliance, if well integrated in the overall communication bundle. The decrease in infection rate in the same period seems to be due to improved HH compliance.

**Disclosure of Interest**: None declared

### P430 NAMING A NEW HAND HYGIENE DEVICE: WHAT’S IN A NAME?

#### C. Fankhauser, T. H. Borzikowsky, D. Pires, E. Tartari, J. Sztajzel-Boissard, A. Peters, C. Guitart, Y. Martin, D. Pittet

##### Infection Control Program, HUG, Geneva, Switzerland

###### **Correspondence:** C. Fankhauser

**Introduction:** Hand hygiene (HH) campaign fatigue obliges stakeholders in healthcare to look for new ideas and tools to promote and improve behavior change. To address this challenge, we developed a device designed to improve the quality of the HH action, and needed to identify the most appropriate name for it.

**Objectives:** To identify the most appropriate name for the device designed to improve the quality of the HH action.

**Methods:** The company Catalyx^®^ was mandated to conduct research to finding a name for the device. Two meetings were held to explain the device’s background, development, and function. A half-a-day workshop (WS) was organized with Catalyx^®^, and 12 experts in HH, innovation and technology transfer to brainstorm about the potential uses of the device and to find fitting names. The 12 preferred names, picked by more than one WS participant, were kept. A survey questionnaire was built and reviewed by experts. It was composed of 3 sections: mental association with the device, a proposed ranking of names, and analysis of drivers for name preferences. To avoid bias in the survey participant’s choices, all names proposed by the WS participants were listed, including those chosen only once. The questionnaire, together with an explanatory video, was sent to 172 infection prevention and control (IPC) experts worldwide. The survey ran from Nov 6^th^ - Dec 23^rd^ 2017.

**Results:** A total of 85 experts responded, resulting in a participation rate of 49%. There were 220 completed activities with 21 ratings and comments, and 27 discussion posts. The HH device was seen as an innovative, practical and positive idea; some concerns about its use in the context of existing guidelines and policies were raised. A good name should reflect both the positive outlook and the device’s function. Concerning name preference drivers, emotional drivers scored higher than rational ones. Two of the 17 proposed names were preferred - both used the term “rub”- and the most frequently first-ranked name was SmartRub.

**Conclusion:** Two names fulfilled all pre-determined criteria, and SmartRub^®^ powered by iQati^TM^ (SmartRub^®^) has been chosen to designate the current device in use for testing.

**Disclosure of Interest**: None declared

### P431 ASSESSMENT OF A NEW ELECTRONIC EDUCATION DEVICE FOR MEASURING THE QUALITY OF HAND HYGIENE ACTION IN CONTROLLED LABORATORY CONDITIONS

#### C. Guitart^1^, Y.-A. Robert^2^, S. Fourquier^3^, Y. Martin^1^, D. Pires^1^, R. Beuchat^3^, D. Pittet^1^

##### ^1^SPCI, HUG; ^2^iQati; ^3^HEPIA, Geneva, Switzerland

###### **Correspondence:** C. Guitart

**Introduction:** Despite significant advances in hand hygiene (HH) monitoring technology, assessment of its accuracy remains scarce. We developed a unique electronic device, SmartRub® powered by iQati ^TM^ (SmartRub®), designed to educate and monitor the quality of HH action by health workers. The device, composed of a wristband and a cylinder included into an alcohol-based handrub (ABHR) pocket-sized dispenser, measures the duration of each HH action and the volume of ABHR poored.

**Objectives:** To assess the sensitivity and specificity of SmartRub® in controlled laboratory conditions.

**Methods:** Voluntary participants performed successive actions, based on real-life gestures, aimed to activate the device (“True actions corresponding to HH actions”) and to challenge the device (“Actions resembling but not related to HH actions”) under controlled conditions. Actions were compared to the raw data captured by SmartRub® and device sensitivity and specificity were calculated accordingly. We evaluated independently the pocket dispenser and the bracelet as well as the overall device.

**Results:** We tested 6 ABHR pocket dispensers (3 rinse; 3 gel) coupled to 6 bracelets. For the dispenser assessment’ step, three volunteers performed 986 actions of which half were true HH actions and half were not related to HH. A total of 510 actions were performed with ABHR rinse and 476 with ABHR gel. The sensitivity and specificity were 90.8% and 100% for the gel category and 99.2% and 91.0% for the rinse category, respectively. For the bracelet’ assessment step, three volunteers performed 422 actions of which half were true HH actions and half were not related to HH. The sensitivity and specificity were 88.2% and 74.7%, respectively. During a third step, we assessed the overall device combined (bracelet + dispenser); seven volunteers performed 816 actions of which half were true HH actions and half were not related to HH. The sensitivity and specificity were 94.1% and 99.0%, respectively.

**Conclusion:** These results indicate the outstanding performance of SmartRub® to capture the quality of HH actions with high sensitivity and high specificity. Next steps will include the assessment of the system in clinical conditions.

**Disclosure of Interest**: None declared

### P432 ASSESSING THE PERFORMANCE OF SMARTRUB® FOR MEASURING HAND HYGIENE ACTION QUALITY BY HEALTH WORKERS DURING A CLINICAL PLANNED PATH IN A HOSPITAL WARD

#### C. Guitart^1^, Y.-A. Robert^2^, S. Fourquier^3^, Y. Martin^1^, D. Pires^1^, R. Beuchat^3^, D. Pittet^1^

##### ^1^SPCI, HUG; ^2^iQati; ^3^HEPIA, Geneva, Switzerland

###### **Correspondence:** C. Guitart

**Introduction:** The electronic device SmartRub® powered by iQati^TM^ (SmartRub®) was designed to monitor the quality of hand hygiene (HH) action by health workers (HCW). SmartRub® consists of a wristband and a cylinder included into an alcohol-based handrub (ABHR) pocket-sized dispenser, to measure both the volume of ABHR poured and the duration of each HH action. Additionally, a feedback/vibration is given to the HCW when these two parameters are well respected.

**Objectives:** We conducted a rigorous validation of the device performance using a planned clinical path scenario.

**Methods:** The planned path was performed in a hospital ward to allow for the evaluation of the device by activating it with pre-determined actions. It consisted of five “true actions” (real HH action gestures) and five “wrong actions” (actions resembling but not related to HH), precisely defined in time and space along the planned path. The performance of SmartRub® was evaluated by quantifying its ability to capture these actions performed by volunteers. In order to assess if the feedback influenced the device accuracy, we performed half of the study with and half without feedback activation.

**Results:** Overall, 11 volunteers performed 85 planned paths; a total of 835 actions were performed of which 419 were “true actions” and 416 were “wrong actions”. In total, 6 ABHR dispensers (3 gel; 3 rinse) and 6 bracelets were used. A total of 427 actions were performed with the feedback activated and 408 without. When the feedback was activated, sensitivity and specificity were 90.6% and 82.7%, respectively; when the feedback was not activated, sensitivity and specificity were 97.1% and 82.8%, respectively. Overall, the sensitivity of SmartRub® to detect HH actions was 93.8%; the specificity 82.8%. Importantly, the “wrong” action “cleaning the environmental surface with the ABHR” contributed largely to the decreased specificity.

**Conclusion:** These results indicate the great performance of using SmartRub® to capture individual HCW behaviors regarding the quality of their HH actions in a close-to-real life situation using a pre-determined clinical planned path. The next phase will be to perform a study in clinical conditions.

**Disclosure of Interest**: None declared

### P433 FIELD TESTING SMARTRUB®: A NEW ELECTRONIC INDIVIDUAL DEVICE TO MONITOR AND FEEDBACK THE QUALITY OF HAND HYGIENE ACTION TO CLINICAL STAFF

#### C. Guitart^1^, Y.-A. Robert^2^, S. Fourquier^3^, Y. Martin^1^, D. Pires^1^, R. Beuchat^3^, D. Pittet^1^

##### ^1^SPCI, HUG; ^2^iQati; ^3^HEPIA, Geneva, Switzerland

###### **Correspondence:** C. Guitart

**Introduction:** SmartRub® powered by iQati^TM^ (SmartRub®) is an electronic device designed to monitor the quality of hand hygiene (HH) action by health workers (HCW). It takes in account two parameters: the duration of HH action and the volume of alcohol-based handrub (ABHR) used. We performed a 3-phases validation approach of SmartRub® performance comparing the system to the gold standard of direct observation of HH practices. Following the two first testing phases in the laboratory and a planned path in a clinical ward, we validated the device in real life clinical conditions.

**Objectives:** To assess the performance of SmartRub® in real-world clinical practices.

**Methods:** The study was performed in one clinical ward in a tertiary level hospital in Geneva, Switzerland. Observers documented all consecutive activities of volunteer HCWs by direct observation. This real-life validation phase was designed to quantify SmartRub®'s ability to capture HH actions in the clinical environment. Data collected included: the number of HH actions, the exact time and the device code tested by each volunteer. A “true” HH action was defined as a HH action observed within the sequence of patient care; a “wrong” action was defined as the time lapse between two “true” actions/HH actions.

**Results:** In total, 17 volunteers (nurses and nursing assistants) participated. In April 2019, they were observed during 903 minutes in total. They performed 485 actions of which 249 were “true” actions; 236 were “wrong actions”. In total, 92% of “true” actions were performed using an ABHR rinse and 8% with a gel. The overall sensitivity of SmartRub® to detect “true” HH actions was 96.8% and the specificity was 98.3%. Four different devices were tested; no significant difference in terms of sensitivity (range 93.2% to 100%) and specificity (range 97.6% to 100%) was observed between the 4 devices.

**Conclusion:** The performance of SmartRub® in the currently tested clinical environment was outstanding. Objective measures of sensitivity and specificity indicate the promise of SmartRub® to capture behaviors associated with individual ABHR use among HCWs in clinical conditions.

**Disclosure of Interest**: None declared

### P434 VIEWS AND EXPERIENCES OF HEALTHCARE WORKERS ON THE USE OF AN INNOVATIVE WRISTBAND (SMARTRUB®) TO IMPROVE HAND HYGIENE ACTION

#### D. Pires^1^, C. Fankhauser^1^, A. Peters^1^, E. Tartari^1^, F. Tymurkaynak^1^, A. Gayet-Ageron^1^, Y. Robert^2^, C. Guitart^1^, S. Fourquier^3^, H. Soule^1^, R. Beuchat^3^, W. Zingg^1^, F. Bellissimo-Rodrigues^1^, Y. Martin^1,2,3^, D. Pittet^1^

##### ^1^IPC, University of Geneva Hospitals; ^2^iQati; ^3^HEPIA, Geneva, Switzerland

###### **Correspondence:** D. Pires

**Introduction:** SmartRub^®^ powered by iQati^TM^ is an innovative wristband that provides automatic and individual feedback on the correct duration of hand friction and volume of alcohol-based handrub (ABHR) used.

**Objectives:** To address the views and experiences of healthcare workers (HCWs) regarding SmartRub^®^.

**Methods:** In March 2018 we emailed a questionnaire to 97 participants of a 6-month clinical trial aimed at testing the effect of SmartRub^®^ on hand hygiene (HH) compliance. The 1^st^ part of the survey was based on the Theoretical Domains Framework and the Technology Acceptance Models and included the constructs perceived usefulness, perceived easy of use, attitude, subjective norm, facilitating conditions, anxiety, voluntariness and behaviour intention. The 2^nd^ part inquired about HH: knowledge and beliefs on self-effectiveness, work motivation, and professional role.

**Results:** A total of 70 HCWs (72%) returned the questionnaire: 41 nurses, 22 auxiliary nurses and 7 others. The majority agreed that SmartRub^®^ was helpful as a reminder of the correct performance of HH action (55). Most perceived the tool comprehensible (49) and easy to use (47). Only 19 HCWs were concerned about confidentiality issues and 16 found that using the wristband bothered the performance of clinical activities. 40 HCWs would continue to use it after the trial once technical issues were resolved. SmartRub^®^ is perceived as a great tool to train students (62) and new HCWs (59), but ultimately, it is regarded as potentially useful to improve HH among all HCWs (53). Volume of ABHR, duration, and technique of HH are seen as important to prevent healthcare-acquired infections by 63, 64 and 63 HCWs, respectively. After participating in the trial, HCWs affirm to use more ABHR (36), and rub hands longer (43).

**Conclusion:** SmartRub^®^ is perceived by the majority of HCWs as an easy and useful tool for HH training. Up to a half of HCWs affirm to have changed their behaviour towards HH regarding the volume of ABHR and duration of hand friction after the use of SmartRub^®^.

**Disclosure of Interest**: None declared

### P435 UNDERSTANDING THE ACCEPTABILITY OF DIFFERENT ABHR FOAM DOSE SIZES WITH HEALTHCARE WORKERS IN THE CONTEXT OF THEIR HAND SIZE, FREQUENCY OF USE, PROFESSION AND EXPERIENCE.

#### K. Ormandy^1^, G. Oxley^1^, A. McGeer^2^, C. Moore^2^, L. McCreight^2^

##### ^1^SC Johnson Professional, Denby, United Kingdom; ^2^Mt Sinai Heath System, Toronto, Canada

###### **Correspondence:** K. Ormandy

**Introduction:** Effective ABHRs and healthcare worker (HCW) compliance to hand hygiene guidelines are important in the prevention of infection transmission in healthcare settings. Compliance to hand hygiene guidelines is affected by many factors including education, ABHR availability, time pressure, skin health and user acceptance of the dose size.

**Objectives:** To assess different ABHR foam dose sizes that are within the WHO recommended drying time of between 20-30 secs (1.3, 1.5, 1.6 and 1.7mL) with a variety of healthcare workers and consider how repeated use, hand size and years in the jobs might affect acceptability.

**Methods:** A total of 197 HCWs evaluated a random combination of 3 out of the 4 dose sizes, in a random order, during a central location test at Mount Sinai Hospital, CA. the acceptability of each dose was assessed and rated on a 7-point agreement scale based on their level of agreement with the following statement: ‘this product is ideal for me and my patients’. The number of 'acceptability' responses, and the number of top box responses (5-7) for each dose size were analysed using the Chi-Squared test statistical method.

**Results:** - 80% of assessments scored 1.3ml and 1.5ml as acceptable opposed to 70% for 1.6ml and 1.7ml. 1.3ml was rated significantly higher than 1.6ml and 1.7ml on ideality.

- Considering the first product tested only, there were no significant differences in dose size rating. When multiple doses were tested, 1.3ml scored higher than the other doses.

- Dose size acceptability was influenced by hand size - larger hand sizes were more accepting of the doses

- HCWs employed by Mount Sinai for less than 3 years were less accepting of the dose sizes than those working at the hospital for 6-20 years.

- 47% of HCWs felt all 3 of their assessed dose sizes were acceptable.

**Conclusion:** When defining the right dose, testing in isolation does not reflect the effect of repeated use. There is a decline in acceptability after 1.5ml, with 1.3ml being the more favoured dose. Smaller doses may therefore increase hand hygiene compliance. Hand size was also an important factor affecting dose size acceptability.

**Disclosure of Interest**: K. Ormandy: None declared, G. Oxley: None declared, A. McGeer Other conflict with: Customer of SCJP, C. Moore Other conflict with: Customer of SCJP, L. McCreight Other conflict with: Customer of SCJP

### P436 EVALUATION OF VIRUCIDAL EFFICACY OF ALCOHOL-BASED HAND RUBS AGAINST NOROVIRUS – COMPARISON OF INTERNATIONAL TEST METHODS

#### J. Steinmann^1^, S. Pahl^1^, K. Ormandy^2^, F. Brill^1^

##### ^1^Dr. Brill + Partner GmbH, Hamburg, Germany; ^2^SC Johnson, Denby, United Kingdom

###### **Correspondence:** S. Pahl

**Introduction:** The virucidal efficacy of alcohol-based hand rubs (ABHR) is essential ensuring infection prevention. While in Europe virucidal claims are accepted, the FDA does not allow such claims. Efficacy can be tested with in-vivo methods including the whole hand method ASTM E2011-13 and EN 1500.

**Objectives:** We studied the activity of a commercial ABHR and 70 % ethanol as reference against the clinical relevant norovirus using the murine norovirus (MNV) as surrogate. The question was, how to get scientific and regulatory acceptance evaluating virucidal efficacy of ABHR.

**Methods:** The reference “70% (w/w) ethanol” and a commercial “preparation” with 80% (w/w) ethanol as active agent have been included in our study. The test virus was MNV strain S99. Activity tests have been performed in-vivo according to ASTM E2011-13 with five subjects in each group and in a cross-over design based on EN 1500 with 19 subjects.

**Results:** Based on EN 1500 70% (w/w) ethanol and the “preparation” showed comparable mean log reductions (RF) of 2.74 and 2.88, respectively. According to ASTM E2011-13 the RF were 4.05 for 70% (w/w) ethanol and 4.25 for the “preparation”. No statistical difference was measured between the test preparations (p > 0.05). However, the difference between the two methods is significant (p < 0.05).

**Conclusion:** Methodological factors like contamination, way of application and recovery might result in different RFs of both methods. However, the methods are able to yield similar results when comparing with the ethanol reference. The EN 1500 suggests a cross-over design with a mandatory control group. This results in an activity evaluation, which is independent from contributing factors including differences between subjects, application etc. The ASTM E2011-13 additionally allows comparing disinfecting with mechanical effect. A combination of both methods might be considered to get an internationally accepted method for virucidal efficacy evaluation of ABHRs.

**Disclosure of Interest**: J. Steinmann Grant/Research support from: SC Johnson, S. Pahl Grant/Research support from: SC Johnson, K. Ormandy Employee of: SC Johnson, F. Brill Grant/Research support from: SC Johnson

### P437 SPRAYED ALCOHOL-BASED HAND RUB WITH RUBBING: AN ALTERNATIVE METHOD FOR EFFECTIVE HAND HYGIENE

#### J. B. X. Tan^1^, M. De Kraker^2^, D. Pires^2^, H. Soule^2^, D. Pittet^2^

##### ^1^Singapore General Hospital, Singapore, Singapore; ^2^Infection Control Program, Geneva University Hospitals and Faculty of Medicine, Geneva, Switzerland

###### **Correspondence:** M. De Kraker

**Introduction:** Hand hygiene is crucial in infection prevention and control. It is unclear whether sprayed alcohol-based hand rub (ABHR) has a role to play in effective hand hygiene in the healthcare setting.

**Objectives:** We tested whether the reduction in bacterial counts on hands after the utilization of sprayed ABHR with or without rubbing was non-inferior (margin log_10_ 0.6 Colony Forming Units [CFU]/ml) to the standardized WHO technique.

**Methods:** We conducted an experimental study based on the European Norm 1500. Experienced volunteers performed hand hygiene using (1) liquid ABHR with rubbing, (2) sprayed ABHR with rubbing, and (3) sprayed ABHR without rubbing. Hands were contaminated with *Escherichia coli* ATCC 10536 prior to each hand hygiene action. Each volunteer had all fingertips of both hands rubbed in tryptone soy broth before and after hand hygiene. Dilutions were inoculated on tryptic soy agar incubated under aerobic conditions for 48 hours. A generalized linear mixed model with a random effect to account for within-subject clustering was applied to analyze the reduction in bacterial counts post-hand hygiene.

**Results:** A total of 19 healthcare workers participated in the study performing all three experiments. hand hygiene using sprayed ABHR with rubbing was non-inferior to using liquid ABHR with rubbing: bacterial count reduction was log_10_ 3.66 CFU/ml (95% CI 1.68-5.64) and log_10_ 3.46 CFU/mL (95% CI 1.27-5.65), respectively. Conversely, non-inferiority was not found for hand hygiene using sprayed ABHR without rubbing (reduction: log_10_ 2.76 CFU/ml, 95% CI 1.65-3.87).

**Conclusion:** In an experimental setting, hand hygiene using sprayed ABHR combined with rubbing was non-inferior to using the WHO method of liquid ABHR with rubbing in reducing bacterial counts on hands. Therefore, sprayed ABHR with rubbing may be an acceptable alternative hand hygiene method. However, sprayed ABHR without rubbing is inappropriate for effective infection prevention and control. These findings require further confirmation in other settings, including different pathogens and spraying devices.

**Disclosure of Interest**: None declared

### P438 CRITICAL VOLUMEN LOSS OF COMMON TYPE HANDRUB DISPENSERS

#### S. Bansaghi^1^, C. Guitart^2^, H. Soule^2^, A. Peters^2^, Y. Martin^2^, D. Pires^2^, P. Szeremy^3^, A. Lehotsky^4^, I. Barcs^1^, D. Pittet^2^, T. Haidegger^5^

##### ^1^Depatrment for Epidemiology, Semmelweis University, Budapest, Hungary; ^2^Infection Control Program, Geneva University Hospitals, Geneva, Switzerland; ^3^Faculty of Medicine, University of Szeged, Szeged; ^4^National Institute of Oncology; ^5^Research, Innovation and Service Center , Óbuda University, Budapest, Hungary

###### **Correspondence:** S. Bansaghi

**Introduction:** Proper hand hygiene requires the complete coverage of hand surfaces with alcohol-based handrub (ABHR). The volume of ABHR used plays a critical role, using insufficient amount of ABHR would result in untreated areas on hands, and thus risk of cross-transmission.

**Objectives:** To assess the occurrence of inaccurate dosing in the case of typical wall-mounted ABHR dispensers in hospitals, and to identify key contributing factors to this phenomenon.

**Methods:** A multicenter study was conducted, divided into 3 parts. First, 22 manual and automatic wall-mounted ABHR dispensers were investigated, both gravitational and regular pumped ones. In the second part, 7 different commercially available ABHR of different compositions were tested with the same dispenser, that previously proved to lose significant amount of ABHR within a short period of time. Last, different formulations – gel and liquid – were tested on a leaking dispenser type. In each part, samples were taken from the dispensers after 0, 1, 2, 4, 8 and 12 hours of resting idle. Each measurement was repeated 5 times.

**Results:** 10 of the 22 investigated dispensers suffered at least 20% loss of dispensed volume after 8 hours without use. Liquid level in their container proved to have significant effect on the dispensed volume. In the second part, ABHR composition was shown to have no effect on the dispensed volume. Third, the percentage of the original amount aliquoted was 1.4% after only 4 hours versus 93.6% after 12 hours when the dispenser was filled with a liquid rinse versus a gel, respectively.

**Conclusion:** Some types of dispensers loose significant volume in a relatively short period of time, leading to frequent sub-optimal volume distribution. According to our results, type of dispensers, ABHR formulation (gel or liquid) and volume level in the container are the main contributing factors. Our study draws the attention to the criticality of infrastructure suggesting in hospitals, suggesting that ABHR dispensers should be regularly audited even before purchasing, and periodically thereafter.

**Disclosure of Interest**: None declared

### P439 OPTIMISING HAND HYGIENE PRODUCT DISPENSER DESIGN TO SUPPORT HAND HYGIENE COMPLIANCE

#### D. P. Limbert, J. D. Hines

##### S C Johnson Professional RD&E, Denby, United Kingdom

###### **Correspondence:** D. P. Limbert

**Introduction:** There are many barriers to effective hand hygiene, including the positioning and availability of the hand hygiene products. The design of the hand hygiene product dispenser is key in minimizing this one such barrier.

**Objectives:** Understand the key hand hygiene product dispenser design parameters and their effect on product availability and usage.

**Methods:** A series of qualitative interviews were held with Healthcare workers to understand the key hand hygiene product dispenser design attributes that act as barriers to hand hygiene compliance.

**Results:** The key attributes to be optimized were identified as:

Ease of dispenser refilling to ensure product is always available

Intuitive dispenser activation to ensure that product is easy to obtain

Dispenser aesthetics that that support ease of installation and clarity of hand hygiene product type.

Additional a series of insights were gathered leading to the best combination of features to deliver each of the key attributes.

**Conclusion:** By utilizing a healthcare user centric approach to optimize hand hygiene product dispenser design, it will be possible to improve product positioning and availability in support of hand hygiene compliance.

**Disclosure of Interest**: None declared

### P440 NURSES' SATISFACTION WITH HAND HYGIENE PRODUCTS IN A UNIVERSITY HOSPITAL IN IRAN

#### S. Ghayoomi Noghabi^1^, I. Mostafavi^2^, S. Taherzadeh^2^, M. H. Aelami^3^

##### ^1^Shariati hospital, ^2^Nursing office, ^3^Pediatrics &Infection Control and Hand Hygiene Research Center, MASHHAD UNIVERSITY OF MEDICAL SCIENCES, Mashhad, Iran, Islamic Republic Of

###### **Correspondence:** I. Mostafavi

**Introduction:** Hand hygiene is the first step in preventing and control ofhealth- care associated infections. Nurses' satisfaction with hand hygiene products plays an important role in increasing hand hygiene compliance.

**Objectives:** The aim of this study was to evaluatenurses' level of satisfaction about facilities needed for hand hygiene in Shariati university hospital in Mashhad (a city in north-eastern of Iran).

**Methods:** This cross-sectional study was carried out on 144 employed nurses in Shariati hospital during year 2016.A questionnaire designed to assess product acceptability for hand rub, hand wash and hand scrub according to WHO guideline on hand hygiene in health care facilities. Statistical *analysis* was performed with *SPSS* Version 14.0.

**Results:** Seventy nurses (49.3%)were satisfied with the quality of hand rub product. Only 58 (40.2%)accepted liquid hand soap. The quality of hand scrub productwas poor in majority of nurses; 113(78.4%). The availability of hand rub products was also low in wards, only 65(45.1%) of nurses were satisfied.

**Conclusion:** According to the results of this study it is necessary for hospitals toknow about the employees' satisfaction about thequality and the quantity of hand hygiene products.

**Disclosure of Interest**: None declared

### P441 HAND HYGIENE AND HYPERBARIC OXYGEN THERAPY: BALANCING THE RISK BETWEEN FIRE AND INFECTION

#### S. Masson-Roy^1^, M. Abbas^2^, H. Soule^2^, R. Pignel^3^, D. Pires^2^, D. Pittet^2^

##### ^1^Département de Microbiologie et d’Infectiologie, Centre Hospitalier Affilié Universitaire Hôtel Dieu de Lévis, Lévis, Canada; ^2^Infection Control Programme, Geneva University Hospitals and Faculty of Medicine; ^3^Division of Hyperbaric Medicine, Geneva University Hospitals , Geneva, Switzerland

###### Correspondence: S. Masson-Roy

**Introduction:** Hyperbaric oxygen (HBO) therapy can be used for a diverse range of medical conditions. In the last decade, with the demonstration of its efficacy for patients with specific diseases, its use in medical institutions has increased. With more patients accessing this therapy in multiplace chambers where risks of cross-transmission are higher, best practices in infection prevention and control (IPC) must be addressed and revised. Hand hygiene (HH) is challenging in HBO therapy settings because alcohol-based hand rubs (ABHRs), the preferred products for HH, are flammable in oxygen-enriched environments. The purpose of this narrative review is to assess fire risks associated with use of HH agents in HBO chambers and to evaluate alternatives.

**Objectives:** To assess risk of fire associated with use of HH agents in HBO chambers and to evaluate alternatives.

**Methods:** We conducted a non-systematic literature search using Pubmed, Embase and Google Scholar, and the following key words: hyperbaric oxygen therapy; hand hygiene; hand rubbing; fire; alcohol-based hand rub; hand sanitizer; hand disinfection; infection prevention and control. Products such as alcohol-based hand rub and soap and alternative non-flammable hand hygiene products were considered. The search included articles written in French or English and published up to October 2018.

**Results:** Scientific evidence regarding HH best practices in HBO chambers is scarce. We found no guidelines issuing specific recommendations regarding flammable agents used for HH nor on alternative products. Even though alcohol is prohibited in HBO chambers according to international guidance, ABHR is frequently used in this setting. Soaps containing combustible substances such as glycerin may also represent a risk of fire in HBO chambers. However, safer alternatives to these HH agents could be employed during HBO therapy such as aqueous chlorhexidine 2% or povidone-iodine.

**Conclusion:** Hand antisepsis with formulations containing either aqueous chlorhexidine 2% or povidone-iodine are the best alternatives to ABHR and glycerin-containing soap in HBO chambers. Additional scientific data are needed to assess the compatibility of other HH agents currently prohibited in HBO chambers because of their combustible content.

**Disclosure of Interest**: None declared

## Poster session: Tuberculosis

### P442 TRENDS OF TUBERCULOSIS AND INCIDENCE OF ANTIBIOTIC RESISTANCE (RIFAMPICIN) AMONG TUBERCULOSIS CASES: IMPLICATIONS FOR ANTIMICROBIAL STEWARDSHIP PROGRAMMING IN LIBERIA

#### M. B. Bolongei^1^, M. K. Jeuronlon^2^, G. J. Mulbah^3^

##### ^1^Infection Prevention and Control, WHE; ^2^Disease Prevention and Control, World Health Organization; ^3^Laboratory, WHE Cluster, WHO, Monrovia, Liberia

###### **Correspondence:** M. B. Bolongei

**Introduction:** Tuberculosis (TB) is yet a significant health problem in Liberia as the country is among the ten countries with estimated incidence rate of 308/100,000 populations and with a minimum number of 10,000 cases per year. The high TB prevalence in Liberia is further complicated by inadequate funding, increase in prevalence of drug resistance, TB and other socioeconomic determinants such as inadequate housing and poor-quality health care services; hence, the need to increase control efforts and support for TB in Liberia.

**Objectives:** To find out the nationwide trends in tuberculosis and incidence of antibiotic sensitivity that would inform antimicrobial stewardship programming in health care settings in order to mitigate risk of antimicrobial resistance in the country.

**Methods:** Retrospective data were analyzed from multiple tuberculosis treatment centers, collected by the National Leprosy and Tuberculosis Program (NLTCP) in collaboration with partners. Data was analyzed using Microsoft Excel Version 2013.

**Results:** The estimated incidence and notification of TB cases were 12,987 and 6,667 in 2010, as compared to 15,584 and 8,335 in 2018 respectively**.** Ninety four (94) Acid Fast Bacillus (AFB) microscopy centers in 6 counties are activated for conducting AFB microscopy test, with a TB case detection rate of 35.1%; a total of 2,928 new TB cases were detected (1,233 cases by AFB microscopy method (Ziehl Neesen Stain) and 1,695 cases by GeneXpert method. A total of 1,614 Drug Sensitivity Testing (DST) was conducted at 17 GeneXpert testing sites of which 462 were found to be rifampicin resistant TB. The country is estimated to notify 15,584 all forms of TB cases and 390 MDR/RR-TB cases in 2018, only 8,335 (53%) and 65 (17%) were notified in 2018.

**Conclusion:** The response to tuberculosis is one of the major priorities of the Ministry of Health and key MOH partners including WHO, UNAIDS, UNICEF, etc. Findings from the study had underscored the need to intensify control efforts and support for TB as well AMR stewardship programs in Liberia.

**References**

WHO. (2018). WHO- Liberia Country Office, Annual Report

WHO. (2017). Global Tuberculosis Report

Ministry of Health, Republic of Liberia. (2016). Joint Health Sector Review

**Disclosure of Interest**: None declared

### P443 OUTCOMES OF NURSES LED COMMUNITY-BASED INTENSIVE-PHASE MULTI-DRUGS RESISTANT TUBERCULOSIS (MDR-TB) TREATMENT PILOT IN MONTSERRADO, COUNTY, LIBERIA

#### L. Z. Mianue

##### Infection Prevention and Control (IPC)., TB ANNEX HOSPITAL MINISTRY OF HEALTH, Monrovia, Liberia

**Introduction:** The estimated TB incidence rate in Liberia is 308/100000 population, up from 282/100 000 population in 2009 (WHO, 2016). The high TB prevalence is complicated by increase drug resistance and other socioeconomic determinants, including inadequate housing, poor-quality health care services.

**Objectives:** To evaluate the implementation of community-based intensive-phase of MDR-TB treatment pilot that would inform decentralizing MDR-TB treatment centers to mitigate the disease burden.

**Methods:** A prospective cohort study with real-time data collection was adopted, where all six patients spent about six months in the National TB Hospital before transition in the community-based treatment pilot. The Hospital Management held engagement meeting, involving health workers, patients and their family caregivers to explain the aims of the pilot. Patients signed consent forms before discharge from the TB Hospital. Each patient had at least 3 consecutive negative sputum smears using Acid-Fast Bacillus before enrolling the community-based treatment. Laboratory monitoring, including audiometric exams were conducted, health education on infection control, drugs adverse effects were provided to patients and their families by Nurses.

**Results:** Fifty (50%) of the patients were males and 50% females. The overall recovery rate was 100%; no defaulter nor death. The patients’ age ranged from 20-35 years, with mean age 26, while 50% were between 25-29 years old. The average length of stay during the intensive treatment phase was 224 days (8 months). The average days spent in the community-based treatment were 53, minimum and maximum days of 33 and 51. Improved patients’ satisfaction, reduced caseload on clinicians and rigorous promotion of IPC practices, cough etiquette were observed amongst patients.

**Conclusion:** Addressing TB is a key priority in Liberia, but human resource and facility capacity constraints remain in managing MDR-TB. Once patients are stabilized with at least 3 consecutive negative AFB smears, they can be safely transitioned to home-based treatment, with routine follow by trained health professionals; which reduces crowdedness in facility and improve recovery.

**References**

WHO. (2018). WHO Liberia Country Office, Annual Report

WHO. (2017). Global Tuberculosis Report

Ministry of Health. (2016). Joint Health Sector Review, Ministry of Health, Republic of Liberia

**Disclosure of Interest**: None declared

### P444 PROLONGED MDR-TB INFECTIOUSNESS AND ITS DETERMINANTS AMONG HIV CO-INFECTED PATIENTS IN A RURAL SOUTH AFRICAN PROVINCE: ARE WE LEARNING FROM THE HISTORY OR REPEATING IT?

#### T. Apalata^1,2^, S. Mvo^1^, A. S. Ikabu^3,4^

##### ^1^Division of Medical Microbiology, Department of Pathology & Laboratory Medicine, Faculty of Health Sciences, Walter Sisulu University; ^2^Department of Medical Microbiology, National Health Laboratory Services, Nelson Mandela Academic Complex, Mthatha, South Africa; ^3^Excellent Medical Laboratory Services, Oshakati, Namibia; ^4^Department of Medical Microbiology, College of Health Sciences, University of KwaZulu-Natal, Durban, South Africa

###### **Correspondence:** T. Apalata

**Introduction:** A case with active pulmonary tuberculosis (TB) can infect up to 10 to 15 new people on average each year. This is of concern, particularly in high HIV prevalence areas such as South Africa.

**Objectives:** The study aimed at determining HIV status and multidrug-resistance (MDR) on TB infectiousness following the initiation of an appropriate TB therapy.

**Methods:** Newly diagnosed patients with pulmonary TB were prospectively enrolled from 6/2017 to 2018 in a MDR-TB clinic in South Africa. Sputum was tested using Xpert® MTB/RIF assay and line probe assays. Microscopy tests using Ziehl-Neelsen and fluorescence staining were performed at baseline, 4, 8 and 12 weeks after TB therapy-start, and graded using the WHO/IUATLD TB management classification. Microscopical conversion was modelled using Kaplan-Meier plots and Cox regression analyses.

**Results:** Of 200 patients, 100 were MDR-TB (50.0%), 103 (51.5%) were male, 55 (27.5%) were ≥35 years, and 114 (57%) were HIV positive. After 12 weeks, there was significant microscopy conversion among non-MDR-TB patients [43/45 (95.6%)] compared to MDR-TB patients [54/69 (78.3%)] (P=0.009), all co-infected with HIV. A significant conversion rate was also identified among non-MDR-TB patients [48/55 (69.6%)] compared to MDR-TB patients [21/31(30.4%)] (P=0.03). Time to microscopy conversion in HIV positive patients ≥35 and below 35 years was 4.58 ± 2.97 and 5.69 ± 3.25 weeks, respectively. Conversion in HIV negative patients ≥35 and below 35 years was 5.00 ± 2.83 and 6.86 ± 3.59 weeks, respectively (P=0.003). Correlation between CD4 cell count at baseline and conversion after 12 weeks was significant (P=0.010). At 8 weeks, all MDR-TB patients with a baseline smear count from scanty to 1+ converted negative while 25% of patients with a baseline count of 2+/3+ remained positive at the end of 12 weeks (P=0.014). Only the baseline microscopy count was independently associated with infectiousness of MDR-TB patients after 12 weeks (P=0.014).

**Conclusion:** Isolation precaution measures for TB patients should remain in place beyond 12 weeks of effective anti-TB therapy when managing MDR-TB patients, irrespective of their HIV status.

**Disclosure of Interest**: None declared

### P445 WHAT IS REQUIRED TO PREVENT TUBERCULOSIS AT REGIONAL HOSPITALS IN JAPAN?

#### N. Suzuki^1^, Y. Kitagawa^2^

##### ^1^Medical Safety Division, NATIONAL HOSPITAL AORGANIZATION HIGASHIOWARI NATIONAL HOSPITAL, Nagoya; ^2^Department of Infection Control, National Center for Geriatrics and Gerontology, Obu, Aichi, Japan

###### **Correspondence:** N. Suzuki

**Introduction:** The prevalence of tuberculosis (TB) in healthcare workers (HCWs) is generally about twice that of regular people the same age. In Japan, in recent years, we have a serious problem with Doctor‘s Delay of elderly people’s diagnosis of TB because the symptoms of elderly TB were not typical and not always accompanied by fever and prolonged cough symptoms. However, the actual status of tuberculosis infections and countermeasures at community hospitals, which are mainly responsible for elderly medical care, has not yet been known.

**Objectives:** In recent years,we would like to clarify whether there is an outbreak of TB in elderly patients or HCWs within the regional hospitals in urban areas in Japan, and what factors are involved.

**Methods:** In October 2018, we surveyed whether the hospitals experienced TB onset of patients or staff in the hospitals in recent years, on regional hospitals in urban areas of central Japan. We also investigated whether they had diagnosed for Latent Tuberculosis Infection (LTBI) caused by TB in-hospital occurrence were carried out. And we examined the facility background concerning TB infection control measures. We used Pearson's correlation coefficient for statistical analysis.

**Results:** The subjects were 98 regional hospitals with the average bed of 102.0 beds and the average patient age of 75.5 years old. 61 (64%) hospitals had experienced TB onset of patients in 3 years, and 7 (7%) hospitals had experienced TB in their HCWs. 65 (68%) hospitals had conducted diagnostic tests for Latent Tuberculosis Infection (LTBI) caused by TB outbreak in 5 years. Patients’ ages (*r*= 0.327, p = 0.002), the number of beds (*r*= 0.342, p = 0.001), and the number of airborne precaution rooms (*r*= -0.342, p = 0.022) were associated with TB outbreak in the hospitals. And HCWs' TB onset was associated with patient TB onset (*r* = - 0.240, p = 0.023).

**Conclusion:** In Japan, even in regional hospitals, the onset of TB in the HCWs associated with the outbreak of TB in elderly patients. As a countermeasure to this, it should consider reducing the number of hospital beds and increasing the airborne precaution room.

**Disclosure of Interest**: None declared

### P446 IMPLEMENTATION OF TB INFECTION CONTROL IN NEPAL: CHALLENGES AND WAY FORWARD

#### P. Shrestha

##### Health Research and Social Development Forum (HERD), Kathmandu, Nepal

**Introduction:** Infection control is a major components of END TB (Tuberculosis) Strategy. The risk of transmission of TB is high in healthcare and congregated settings. In Nepal, infection control is a key component of TB preventive services. The National Strategic Plan (NSP) 2016-2021 envisions TB infection control as a part of national infection prevention and control policy throughout the country.

**Objectives:** The major objective of this study was to identify the implementation status of TB infection control measures in Nepal.

**Methods:** Desk review was conducted. Different literatures, reports and policy documents related to infection control were reviewed. Descriptive analysis was conducted.

**Results:** In Nepal, IC program focuses on Drug Resistant (DR) TB. DR TB centres were provided with exhaust fan, Ultraviolet Germicidal Irradiation (UVGI), N95 masks and simple surgical masks. All TB trainings incorporated sessions related to infection control. Another activity was implementation of FAST (Finding Actively, Separate temporarily and Treat effectively) approach in 15 major hospitals of Nepal that aimed to actively find people with cough, testing through rapid molecular diagnostics and enabling prompt treatment decreasing TB transmission.

The National Strategic Plan (NSP) 2016-2021 foresees improvements of infection control measures in all DR TB centers and subcentres based on proposed infection control guidelines. It also states the ppointment of focal person for IC and gradual increase in the budget for infection control. National infection control policy is still not in place and no focal person has been appointed. Different evidences show lack of awareness about IC measures. In the fiscal year 2017/18, there were no provision of budget for IC through domestic funds.

**Conclusion:** Significant efforts are required to address the issue. The national policy and guidelines on Infection control should be prepared to facilitate implementation of IC measures in all health facilities focusing on integrated implementation of administrative control, environmental control and respiratory protection. Current IC measures should be strengthened with gradual expansion to all centers. A designated focal person should be assigned for overseeing the programs related to IC. Domestic funding for IC should be considered. In addition, there is a need to raise awareness on TB IC among all types of health workers, patients and community members.

**Disclosure of Interest**: None declared

## Poster session: Surveillance 2

### P447 POSTOPERATIVE WOUND INFECTIONS IN THE PRE-POST-INTERVENTION COMPARISON OF A PROSPECTIVE COHORT STUDY ON THE IMPLEMENTATION OF INFECTION PREVENTION MEASURES

#### M. M. Strybos^1^, R. Otchwemah^1^, I.-K. Dombrowski^1^, J. Hoffmann^1^, F. Mattner^1^

##### ^1^Institute for Hygiene, Kliniken der Stadt Köln gGmbH, Cologne, Germany

###### **Correspondence:** M. M. Strybos

**Introduction:**

In the project "HygArzt" (ZMVI1-2516FSB111), funded by the Federal Ministry of Health (BMG), the effects of Infection prevention measures (IPM) (e.g. decolonisation of patients before surgery and introduction of a new dressing change concept, introduced by physicians responsible for hygiene in trauma surgery/orthopedics) were investigated.

**Objectives:**

Therefore, in this prospective cohort study, all NI (patient outcomes) on three orthopedic trauma surgery normal wards are for the first time completely recorded.

**Methods:**

In order to identify and document NI, clinical signs of infection were recorded according to KISS and CDC definitions. Data from pre-existing conditions and from current and past infections from the hospital management system, admission forms, discharge letters and care documentation were aggregated with current patient data and laboratory findings. In addition, early visits were made three times a week in order to record signs of infection that had not yet been documented.

**Results:**

In order to check the effects of the implemented IPM on the infection rates (NI, SSI), the pre- and post-intervention phases were compared. In the first five months of the pre-phase (1100 surgeries - partly multiple surgeries on the same patient) the rates were NI (n=42) 3.8% (CI 95% 2.7; 4.9) and SSI (n=34) 3.1% (CI 95% 2.1; 4.1). In the first five months of the post phase (1127 surgeries - partly multiple surgeries on the same patient) total infection rates of NI (n=22) of 1.9% (CI 95% 1.1; 2.7) or SSI (n=17) of 1.5% (CI 95% 0.8; 2.2) were found. For NI (*RR* = 0.51 (CI 95% 0.31; 0.85), *p* = .009) as well as for SSI (*RR* = 0.48 (CI 95% 0.27; 0.87), *p* = .015) significant differences were found between pre and post-phase.

**Conclusion:**

By introducing IPM, the relative risk of getting an infection (SSI and NI) in the post phase could be reduced by almost half compared to the pre phase. While only infection rates from indicator operations have been determined in previous research, and none at departmental level specifically for orthopedic/trauma surgery, study data suggested to better tailor infection prevention measures for a specified medical discipline.

**Disclosure of Interest**: None declared

### P448 MICROBIOLOGICAL PARTICULARITIES OF SURGICAL SITE INFECTIONS IN ONCOLOGIC ORTHOPEDIC SURGERY COMPARED TO NON-ONCOLOGIC SURGERY - SINGLE CENTER EXPERIENCE AND LITERATURE REVIEW

#### I. Uçkay^1^, T. Rod-Fleury^2^, T. Studhalter^1^

##### ^1^Balgrist University Hospital, Zürich, ^2^Geneva University Hospital, Geneva, Switzerland

###### **Correspondence:** I. Uçkay

**Introduction:** Tumor orthopedic surgery has higher incidences of surgical site infections (SSI) than non-oncologic surgery.

**Objectives:** However, their epidemiologic microbiology is rarely published. The knowledge of this epidemiology is necessary to tailor specific perioperative antibiotic prophylaxis regimens.

**Methods:** In our large tertiary composite database of adult orthopedic infections, we compare SSIs in adult oncologic patients to adult non-oncologic orthopedic patients.

**Results:** Among 2752 different first episodes of orthopedic infections in adults, only 14 (0.5%) concerned SSI at the site of prior oncologic surgery. Oncologic patients had no more prior antibiotic therapy (before intraoperative samplings) than non-oncologic patients, but they witnessed significantly more SSIs due to enterococci, Gram-negative pathogens, or infections due to multi-resistant skin commensals. In contrast, the proportion of classic orthopedic pathogens such as Staphylococcus aureus or streptococci was not different from the control group. We couldn’t link the germs to prior oncologic treatment, nor to the length of perioperative surgical antibiotic prophylaxis.

**Conclusion:** The microbiology of orthopedic SSI in adult oncologic patients is significantly different than in non-oncologic patients. Retrospectively, the standard antibiotic prophylaxis is inadequate for the involved pathogens. More studies are needed to tailor a specific perioperative prophylaxis in terms of choice of the agents, rather than of duration of the standard prophylaxis.

**Disclosure of Interest**: None declared

### P449 SURGICAL SITE INFECTION SURVEILLANCE FOLLOWING CARDIAC SURGERIES IN PEDIATRIC PATIENTS AT A TERTIARY CARE CENTER IN LEBANON

#### J. Tannous^1^, N. K. Zahreddine^1^, I. El Rassi ^2^, R. Wakim^3^, Z. Kanafani^4^, S. Kanj^4^

##### ^1^Infection Prevention and control; ^2^cardiac surgery; ^3^pediatric infectious diseases; ^4^Infectious diseases , AUBMC, Beirut, Lebanon

###### **Correspondence:** J. Tannous

**Introduction:** Surgical site infections (SSIs) following cardiac surgeries in pediatric patients are associated with prolonged hospitalization, increased costs and higher mortality. Known risk factors include low birth-weight, non-compliance with antibiotic prophylaxis, and prolonged duration of surgery.

**Objectives:** This study was conducted to determine the SSI risk factors associated with pediatric cardiac surgeries.

**Methods:** A prospective SSI surveillance following pediatrics cardiac procedures was conducted by the infection control (IC) team between 2015 and 2018 at the American University of Beirut Medical Center. The surveillance was based on the CDC/NHSN (Centers Disease Control and Prevention/National Health Care Safety Network) definitions.

**Results:** 17 SSIs were identified following 669 procedures at a rate of 2.5%. SSIs were classified into superficial (53%), deep (18%), and organ-space infections (29%). The NHSN risk index score was 1 in 82% and 2 in 18% of the cases. 76% of the patients were < 1 year old with 69% <15 days old. 59% of the patients weighed < 3.6 Kg. The intra-operative temperature was < 34.9°C in 71% of the patients (range 30-34.8°C). The duration of the surgery lasted >5 hours in 24% of the cases. Compliance with antibiotic prophylaxis was 100% in the drug choice, 82% in the time of administration, and 56% in the intraoperative re-dosing when indicated. 11 (65%) of the patients were ventilated for >6 days and 14 (82%) had a central venous catheter for >5 days post-surgery. 14 (82%) were hospitalized for >10 days after surgery. *Staphylococcus* spp. were the most common causative organisms in 59% of the cases (80% *S. aureus*, mostly methicillin-sensitive, and 20% *coagulase negative Staphylococcus*). 2 of the patients underwent surgical debridement and all the patients recovered.

**Conclusion:** Key strategies were envisioned to minimize the modifiable risk factors related to the surgeries such as improving compliance with antimicrobial prophylaxis and pre-operative bathing with antiseptic soap. Ongoing surveillance and sustaining adequate IC practices are fundamental in preventing SSIs in this population.

**Disclosure of Interest**: None declared

### P450 EPIDEMIOLOGY OF SURGICAL SITE INFECTION IN A QUATERNARY CARE HOSPITAL IN INDIA

#### M. Pillai^1^, A. Warrier^2^, S. Joy^3^, R. Babu^3^, S. Bernadit^1^, K. Charles^1^, M. Jayan^1^ on behalf of Aster Medcity

##### ^1^Infection control; ^2^Infectious diseases and Infection control; ^3^Microbiology, Aster DM Healthcare LTD, Kochi, India

###### **Correspondence:** R. Babu

**Introduction:** SSI is the most frequent type of HAI in low- and middle-income countries (LMICs). Approximately one in 10 people who have surgery in LMIC’s acquire a SSI. In India, there is a paucity of data regarding SSI. Objective of the study was thus to describe the epidemiology of Surgical Site Infections in a Quaternary care Hospital in India.

**Objectives:** To describe the epidemiology of SSI in our hospital, a quaternary care hospital in India and to study some of the risk factors related to SSI in the patients who developed SSI. The pathogens isolated from cases of SSI will also be studied.

**Methods:** Retrospective analysis of case files of surgical patients that were identified with a surgical site infection by the SSI surveillance team using the protocol of the US Centre for Disease and Control and Prevention(CDC) National healthcare and Safety Network(NHSN), in the months of January to December 2018 was done.The cumulative prevalence rate and rate across departments were calculated for the 12 months. Prevalence of risk factors for SSIs and the causative organism with the antibiogram were also looked at in each case.

**Results:** A total of 167 SSI for the 8253 Surgeries done in this period were reported with an annual rate of 2.02%. 22% were reported from surgical Gastroenterology, 16% were reported from Neurosurgery and 13% were reported from Cardiothoracic unit. The most common organism isolated was *Klebisella pneumoniae* followed by *Pseudomonas aeruginosa*. 23.31% of the patients had hypothermia during the perioperative period, 35.83% patients had documented poor glycemic control while antibiotic prophylaxis was non compliant in 7.18% of the cases studied.

**Conclusion:** The prevalence of SSI is quite high in our centre compared to the global benchmarks. Overall SSI rates are reported least in the USA (0.9%) to maximum in SE Asian countries (7.8%). Our rates are around 2% - which is less that for most SE Asia countries. Most of the SSI were contributed by the Surgical Gastroenterology, Neurosurgery and Cardiac surgery departments.

In spite of good adherence to antibiotic prophylaxis - poor compliance to preventing hypothermia and hyperglycemia were identified - these are to be focused now

**Disclosure of Interest**: None declared

### P451 INCIDENCE OF SURGICAL SITE INFECTIONS IN THE SERVICE OF OBSTETRIC GYNECOLOGY IN 2016

#### G. Brahimi, H. KELLAF, S. AIT SEDDIK, N. CHEBOUB, A. REBOUH, S. BOUADI, R. BELKAID

##### ÉPIDÉMIOLOGIE , CHU BENI MESSOUS, ALGER, Algeria

###### **Correspondence:** G. Brahimi

**Introduction:** Monitoring of surgical site infections (SSI) is a priority in our facility. In the Obstetrics Gynecology department, the incidence of SSIs has significantly decreased since it joined the active surveillance network since 2006.

**Objectives:** - Calculate the SSI's incidence

- Identify risk factors related to the occurrence of SSI

**Methods:** Descriptive longitudinal study for analytical purposes. Data collection was carried out between 1st February and 30th May 2016 with a follow-up up to + day 30. The SSI diagnosis was established according to the criteria of the CDC Atlanta. Data entry and analysis was done on the Epi Data Entry 3.02 and Epi Data Analysis software.

**Results:** A total of 193 interventions were included. The average age is 38 +/- 11 years old. 60.1% had an ASA score = 1, the average length of stay was 5.6 +/- 3.3 days. The percentage of patients reviewed on D + 30 was 100%. More than two thirds of the interventions were clean contaminated (69%), over half of operated had a score of NNIS = 0 (64.8%) and almost a third of the interventions carried out in emergency (28.5%). The SSI's incidence is 4.7% (09 patients), the average age of infected patients was 50.44 +/- 12.76 years and mean time to onset of infection is 6.3 +/- 4.5 days.

The SSI's incidence was significantly greater when the patients had performed a depilation using a razor, 9% versus 1.7% for the other types of depilation: cream and mowing, p <0.01. Infected patients had an average duration of surgery significantly greater than uninfected patients (102 minutes versus 76 minutes, p <0.02). The SSI rate was significantly higher when the class Altemeier was contaminated (10.3% versus 2.3% clean contaminated, p <0.05).

**Conclusion:** The surveillance of SSI in Gynecological Obstetrics has highlighted risk factors that should be taken into account to improve the management and prevention of risks associated with surgical care. The aim is to reduce the average duration of surgery, to be cautious in patients with multitares, to avoid mechanical shaving and to promote other types of hair removal: cream and hair trimmers.

**Disclosure of Interest**: None declared

### P452 ADMISSION AND DISCHARGE SCREENING FOR MULTI-RESISTANT CARRIAGE AMONG ADULT PATIENTS IN ORTHOPEDIC SURGERY - EXPERIENCE AT THE BALGRIST / ZURICH

#### T. Studhalter, I. Uçkay

##### Balgrist University Hospital, Zürich, Switzerland

###### **Correspondence:** T. Studhalter

**Introduction:** The Balgrist University Hospital in Zurich is a tertiary referral center for orthopaedic surgery and acute paraplegic care; with an estimated 20% of direct transfers from other institutions all over Switzerland and abroad.

**Objectives:** To verify our isolation and screening policy regarding multiresistant pathogens (partially representative for the Greater Zurich area).

**Methods:** During March 2019, we screened adult patients, hospitalized for more than 48 hours, at entry (+/- 1 day) and at discharge (- 3 days) for nasal, pharyngeal, inguinal and rectal carriage of MRSA, multi-resistant Gram-negative rods and VRE. To reduce sampling bias, the Infection Control nurse and the Infectious Diseases physician sampled all specimens by themselves. In case of doubt, identical species underwent typisation.

**Results:** Of a total of 115 patients hospitalized within the three wards, we sampled 115 at admission (median age 57 years; 42% women), and 100 at discharge. Only 15 patients (13%) were lost at discharge. Another 9 (8%) refused rectal screenings, but accepted groin swabs. The median length of hospital stay was 5 days (range, 2-21 d). Among the study population, 3.4% were known for multi-resistant carriage due to previous targeted screenings.

The MRSA and VRE incidences were both 0% at entry and 0% at discharge; without any acquisition during the stay. This was 6% and 4% for Gram-negatives, respectively, which were all various ESBL. The intra-hospital new acquisition risk was 0.0% an. One possible ESBL transmission revealed different typisation results. The preceding observed global hand hygiene compliance was 68% (418 correct actions out of 615 indications; 83% among the nursing staff).

**Conclusion:** In our institution, we detected no new intra-hospital acquisition of multi-resistant germs. We keep our screening and isolation policies unchanged; with new evaluations in distant future or in case of eventual outbreaks.

**Disclosure of Interest**: None declared

### P453 ANTIMICROBIAL RESISTANCE SURVEY AT OSPEDALE POLICLINICO SAN MARTINO, GENOVA, LIGURIA, NORTH-WEST ITALY

#### G. Noberasco^1^, A. Battaglini^1^, F. Butera^1^, I. G. Iavarone^1^, F. Grammatico^1^, A. Battistini^2^, D. Bellina^2^, B. Guglielmi^2^, A. Marchese^1^, A. Morando^2^, L. Sticchi^1^, A. Orsi^1^

##### ^1^University of Genoa, Genoa, Italy, ^2^Ospedale Policlinico San Martino, Genova, Italy

###### **Correspondence:** A. Orsi

**Introduction:** Antimicrobial resistance (AMR) threatens to send us back to a time when we were unable to easily treat infections and it is listed as one of the top 10 threats to global health in 2019 by the WHO. Resistance in one microorganism against one particular drug may drive treatment decisions of clinicians, thereby fostering selection pressure to resistance development against another antibiotic, often rising complex and difficult scenarios.

**Objectives:** We conducted an investigation on AMR in our 1300 beds tertiary care structure, Ospedale Policlinico San Martino, Genova, Italy. We evaluated the AMR epidemiology in our hospital in comparison with national and european data, to provide an useful tool for the management of resistant bacteria.

**Methods:** AMR was tested following the guidelines of the 2017 Annual report of the European Antimicrobial Resistance Surveillance Network (EARS-Net). Blood and cerebrospinal fluid were the only source specimen considered. We evaluated every positive isolate from 1/1/2014 to 31/12/2018. We excluded same patient cases within 30 days one from another and cases where the AMR was not tested.

**Results:** From 2014 we observed a consistent rise in vancomicyn resistant E. faecium from 20% to 36% of the isolates while the percentage of high dose gentamicyn resistant E. faecalis decreased from 55% to 46 %. We observed that the proportion of meticillin resistant S. aureus is decreasing (from 58% in 2014 to 45% in 2018). K. pneumoniae resistance levels are on a remarkably decreasing trend: through the years we observed a reduction in resistance to fluoroquinolones (from 79% to 54%) , third-generation cephalosporins (from 79% to 55%), aminoglycosides (from 66% to 37%) and carbapenems (from 63% to 39%). We observed a reduction also in the percentage of K. pneumoniae isolates with combined resistance to fluoroquinolones, third-generation cephalosporins and aminoglycosides, from 63% in 2014 to 36% in 2018.

**Conclusion:** AMR is a real issue in public health and needs to be approached with multidisciplinary efforts.

**References**

ECDC. Surveillance of antimicrobial resistance in Europe – Annual report of the European Antimicrobial Resistance Surveillance Network (EARS-Net) 2017. Stockholm, 2018.

**Disclosure of Interest**: None declared

### P454 EPIDEMIOLOGY OF ANTIBIOTIC-RESISTANT BACTERIA AND BLOODSTREAM INFECTIONS IN JAPAN: A MULTICENTER PILOT SURVEILLANCE STUDY

#### T. Tajima^1^, K. Hayakawa^1,2^, N. Matsunaga^1^, M. Endo^1^, T. Suzuki^2^, K. Suzuki^1^, S. Tsuzuki^1^, N. Ohmagari^1,2^

##### ^1^AMR Clinical Reference Center; ^2^Disease Control and Prevention Center, National Center for Global Health and Medicine, tokyo, Japan

###### **Correspondence:** T. Tajima

**Introduction:** The novel national surveillance, Japan Surveillance for Infection Prevention and Healthcare Epidemiology (J-SIPHE), includes microbiological assessment that utilizes the data of preexisting microbiological surveillance (Japan Nosocomial Infections Surveillance: JANIS) at each facility, and enables semi-automated collection of microbiological parameters.

**Objectives:** The epidemiology of major drug-resistant bacteria (DRB) and bloodstream infections (BSI) was evaluated as a multicenter pilot surveillance study using J-SIPHE.

**Methods:** From April to November 2018, 32 facilities provided monthly microbiological data about isolation of major DRBs and BSI due to DRBs through online sources. To avoid duplication, only one episode was counted when the same bacteria was reported from the same patient within one month. These data were divided by denominator data (DRB isolation: /10000 patient-days, BSI: /10000 patient-days).

**Results:** The median number of beds from participating facilities was 432 (IQR: 313-598) and the median length of stay was 12.5 (IQR: 10.5-14.3) days. The median average culture submission rate (number of patients with culture submission/1000 patient-days) was20.8 (IQR: 17.0-24.5). Incidences of isolation of major DRB and BSI are as follows (median [IQR]/mean); MRSA (9.44 [5.73-11.66]/10.1, BSI: 0.67 [0.34-1.01]/0.75), fluoroquinolone-resistant *E.coli* (FQREC) (7.23 [5.26-8.86]/7.6, BSI: 0.70 [0.28-1.36]/0.79), third-generation cephalosporin-resistant *E.coli* (3GCREC) (4.10 [3.46-6.57]/5.0, BSI: 0.36 [0.15-0.84]/0.53), carbapenem-resistant *P. aeruginosa* (CRPA) (1.55 [1.03-2.54]/2.1, BSI: 0 [0-0.19]/0.12), and carbapenem-resistant *Enterobacteriaceae* (CRE) (0.16 [0-0.48]/0.41, BSI: 0 [0-0.02]/0.07).

**Conclusion:** We found that MRSA, FQREC, and 3GCEC were the major DRB endemics in multiple facilities in Japan, whereas CRPA and CRE were only isolated from some participating facilities. As data collection proceeds from additional facilities, J-SIPHE will be able to provide more detailed data on the epidemiology of DRB and BSI in Japan.

**Disclosure of Interest**: None declared

### P455 TRENDS IN FLUOROQUINOLONE RESISTANCE RATE OF ESCHERICHIA COLI (E. COLI) IN JAPAN NOSOCOMIAL INFECTIONS SURVEILLANCE (JANIS)

#### M. Horikoshi^1^, H. Matsuura^2^, Y. Suzuki^1,3,4^, A. Ikeda^1^, H. Noda^5,6^, S. Ikeda^1^, S. Tsuzuki^7^, H. Nishiura^7^, K. Yamagishi^8^, K. Yahara^9^, K. Shibayama^9^, N. Matsunaga^4^, K. Hayakawa^4^, N. Ohmagari^4^

##### ^1^Juntendo University, Bunkyo-ku; ^2^Shoin University, Atsugi; ^3^Osaka Medical College, Takatsuki; ^4^Center Hospital of the National Center for Global Health and Medicine, Shinjuku-ku; ^5^Cabinet Secretariat, Chiyoda-ku; ^6^Osaka University, Suita; ^7^Hokkaido University, Sapporo; ^8^University of Tsukuba, Tsukuba, ^9^National Institute of Infectious Diseases, Shinjuku-ku, Japan

###### **Correspondence:** M. Horikoshi

**Introduction:** Hospitals in Japan have been encouraged to participate in the nosocomial infection surveillance, Japan Nosocomial Infections Surveillance (JANIS), by the medical fee revision in FY2014 decided under national health care insurance system in Japan. As a result, JANIS participating hospitals have been rapidly increasing, and this increase may have affected on the reported trends of the resistance rate.

**Objectives:** The objective of this study is to examine an effect of the hospitals that newly participated in JANIS after 2014 on the reported temporal change of fluoroquinolone resistance of *E. coli*.

**Methods:** Inpatient *E. coli* lab-test records of fluoroquinolone resistance submitted by 1,499 hospitals between 2007 and 2016 were included in the present study. The hospitals were divided into two groups: hospital-A which participated in JANIS before 2014 (n=772) and hospital-B which participated in after 2014 (n=727). The trends of resistance rate adjusted for age, sex, and interaction terms of hospital group and year were calculated using panel data analysis. We also conducted sex-specific age(quartiles)-stratified analysis.

**Results:** The fluoroquinolone resistance rate of *E. coli* increased from 21.2% in 2007 to 34.1% in 2016 (mean annual increase: 1.43%). As limited to examine hospital-A, a significant increased trend in the resistance rate was observed, which was a similar finding using all hospital data. Moreover, the trends of resistance rate were increasing in all age groups for both sex throughout the study period, but older age group tended to have a higher trend compared to other age group.

**Conclusion:** The fluoroquinolone resistance rate of *E. coli* among JANIS registered hospitals has kept increasing since 2007. The trend remained significant even after excluding newly registered hospitals since 2014. Therefore, the medical fee revision in 2014 may not be the only cause of the increase in the resistance rate of Japan.

**Disclosure of Interest**: None declared

### P456 IMPACT OF MODIFIED CDC/NHSN SURVEILLANCE DEFINITION ON THE INCIDENCE OF CAUTI: A STUDY FROM AN INDIAN TERTIARY CARE HOSPITAL.

#### A. Mahapatra^1^, B. Behera^1^, J. Jena^1^, J. Biswala^2^

##### ^1^Microbiology; ^2^Infection control Nurse, All India Institute of Medical sciences(AIIMS), Bhubaneswar, India

###### **Correspondence:** A. Mahapatra

**Introduction:** Catheter-associated urinary tract infections (CAUTIs) is the commonest among all healthcare associated infections. Centers for Disease Control and Prevention(CDC) and National Healthcare Safety Network (NHSN) definitions are widely used for the surveillance of CAUTI. Yeasts were the predominant cause of CAUTIs,(C*andida* sp.-26.1% ) as reported by NHSN in 2013. However, asymptomatic candiduria with >10^5^ colony-forming units/ milliliter(CFUs/ml) in hospitalized patients are considered as colonizers . The definition of CAUTI was revised by NHSN in January 2015 with the modifications -1) *Candida* and nonbacterial isolates as uropathogens were excluded 2) bacterial threshold increased to ≥10^5^ (CFU/ml) in the UTI definitions.

**Objectives:** To determine the impact of 2015 CAUTI definition on the CAUTI rate over that as per 2013 NHSN criteria.

**Methods:** CAUTI rates were calculated/1000 urinary catheter days from patients of different ICUs of All India Institute of Medical Sciences, Bhubaneswar, admitted during 1^st^ August, 2017 to 31^st^ January, as a part of routine HCAI surveillance programme. CAUTI rates were calculated & compared with inclusion & exclusion of *Candida spp.*

**Results:** 86 CAUTIs were observed over 9660 catheter days with 8.9 CAUTIs/1000 catheter days during the study period . *Candida spp*. was identified to be the predominant etiology of CAUTI in 52.32% cases followed by *E. coli* in 15.11% & others. CAUTI rate was reduced to 4.24/1000 catheter days when *Candida spp*. were excluded as an etiology & the absolute incidence rate was reduced to 25%. The mean CAUTI rate was 18.27 when *Candida* were included ,while by exclusion it was decreased to 9.86 CAUTIs/1000 catheter days (95% CI, 0.67–1.34) with an overall mean reduction rate by 46.03% .

**Conclusion:** The CAUTI rate was 8.9/1000 catheter days & was found to be reduced by 46.03%, based solely on excluding cases with candiduria. This significant reduction in the CAUTI rates may be applicable to those institutions having high rates of candiduria in catheterized patients. However, this study has certain limitations such as- single centered, short duration & this reduction in CAUTI may not be applicable in centers with low rates of candiduria.

**Disclosure of Interest**: None declared

### P457 EVALUATION OF BACTERIOLOGICAL TECHNIQUES USED IN MONITORING NOSOCOMIAL INFECTIONS IN YAOUNDE (CAMEROON)

#### N. N. E. Georgette^1^, G. K. Hortense^1^, S. E. Albert^2^, N. Elias^3^

##### ^1^Clinical Biology, CHU Yaounde; ^2^Parasitology, University of Yaounde, ^3^Biological sciences, University of Ngaoundere, Yaounde, Cameroon

###### **Correspondence:** N. N. E. Georgette

**Introduction:** The microbiology laboratory, a key player in bacterial surveillance alone, can detect 50% of nosocomial infections (NSIs).

**Objectives:** Our study aim was to evaluate the credibility of data from Bacteriology laboratories of some hospitals in Yaounde.

**Methods:** We conducted a cross-sectional study from September to December 2018. 10 laboratories participated in our study. A checklist from a standardized tool was used to determine adherence levels of the recommendations by these laboratories. An external evaluation of the quality of their bacteriological diagnosis was then carried out using 5 known bacterial strains (Escherichia coli, Enterobacter faecalis, Pseudomonas aeruginosa, Staphylococcus saprophyticus and Klebsiella pneumoniae) isolated from nosocomial infections (NI).

**Results:** These laboratories had good levels of compliance with the recommendations regarding the availability of tests (90%) and blood culture techniques (60%). 80% of the laboratories did not respect the recommendations of the committees on the fight against NI and that of antibiotic susceptibility testing for resistance detection (70% of the labs). All the laboratories (100%) had good skills for microscopy and isolation. All laboratories (100%) accurately identified Escherichia coli, 20% identified Enterobacter faecalis, 50% identified Pseudomonas aeruginosa, 40% identified Staphylococcus saprophyticus and 60% identified Klebsiella pneumoniae. In addition, 50%.

**Conclusion:** Our findings revealed that there exist limitations on the identification and resistance testing data by Bacteriology laboratories of some hospitals in Yaounde city for an effective monitoring and surveillance of nosocomial infections.

**Disclosure of Interest**: None declared

### P458 ON-SITE VALIDATION OF THE POINT PREVALENCE SURVEY OF HEALTHCARE ASSOCIATED INFECTIONS REVEALS DIFFICULTIES WITH RELIABLE CASE-FINDING IN THE NETHERLANDS

#### J. Wille, T. Hopmans, K. Halonen

##### Senior advisor infection control, RIVM, Bilthoven, Netherlands

###### **Correspondence:** J. Wille

**Introduction:** On-site validations were performed to study the accuracy of the healthcare-associated infections (HAI) registered during the point prevalence surveys (PPS) in The Netherlands. In October 2017 three validations were carried out in context of the European validation study of the ECDC. The aim of the European study was to assess the validity, reliability and inter-country comparability of the data collection. In October 2018 the validation was repeated in three more Dutch hospitals with special attention to the reliability of case-finding.

**Objectives:** The objective of this repeated validations was to collect more data to assess the validity of the case-finding in The Netherlands.

**Methods:** The validations were performed by three infection control practitioners of the Dutch national surveillance network (PREZIES). Data collected by the validation team were disclosed, until the hospital had submitted their data. Afterwards all records were discussed with the hospital staff to identify discordant cases. The survey was performed on patients in the departments where the highest prevalence rates were expected (ICU and surgical departments).

**Results:** In October 2017 and 2018 we validated 327 records (49 to 69 per hospital). There was consensus about 307 records with 34 HAI, after profound discussions 20 discrepancies (2 to 6 per hospital) remained. In 16 of these the validation team considered the patient to have an HAI, in 12 cases the hospital did not and in 4 cases the hospital considered the patient to have an-other type of HAI. In the remaining 4 discrepancies the validation team did consider the patient not to have an HAI, where the hospital did (PPV 89%, NVP 94%).

**Conclusion:** We found 20 discrepancies based on 327 records validated. Although the sample size is small, the findings indicate that a uniform application of the definition set can be difficult. We think that the design of the prevailing protocol and complexity of the definition set may have caused the misinterpretations. On the other hand there was sometimes inadequate knowledge of the protocol and definition set. For now we have introduced assessments of case vignettes in preparation to the PPS which are held twice a year in The Netherlands. Other steps are being considered for the near future.

**Disclosure of Interest**: None declared

### P459 EVALUATING THE STATUS OF INFECTIOUS DISEASE (ID) PRACTICE BY JAPAN SURVEILLANCE FOR INFECTION PREVENTION AND HEALTHCARE EPIDEMIOLOGY (J-SIPHE)

#### T. Suzuki^1,2^, K. Hayakawa^1,2^, T. Tajima^2^, M. Endo^2^, K. Suzuki^2^, S. Tsuzuki^2^, N. Matsunaga^2^, N. Ohmagari^1,2^

##### ^1^Disease Control and Prevention Center; ^2^AMR Clinical Reference Center, National Center for Global Health and Medicine, Tokyo, Japan

###### **Correspondence:** T. Suzuki

**Introduction:** As per the Japan National Action Plan (2016), AMRCRC has developed a national surveillance system (J-SIPHE) for healthcare facilities to enable data collection on several AMR related parameters and to provide prompt feedback.

**Objectives:** Using data from the J-SIPHE pilot surveillance period (04/2018–11/2018), we aimed to elucidate the baseline data of ID practice in Japan on which existing data have been scarce.

**Methods:** Among 5 major categories included in J-SIPHE (microbiological assessment, antimicrobial use/ stewardship, healthcare-associated infection, infection control practices, and ID practice), data on ID practice were evaluated. The *p* value was calculated using Mann-Whitney U test, and *p* < 0.05 was considered statistically significant.

**Results:** In all, 23 hospitals (median inpatient beds: 518 (interquartile range [IQR]: 436–574) registered their data on ID practice. Smaller hospitals (< 500 beds) had more physicians for ID consultations (IDC) than larger hospitals (per 100 beds; total: 0.4 [0.2–0.7], smaller: 0.6 [0.5–2.1], larger: 0.2 [0.2–0.4], p=0.027) (per 1000 patient-days; total: 0.2 [0.1–0.4], smaller: 0.3 (0.2–1.0), larger: 0.1 (0.1–0.2), p=0.042). The number of ID board certified physicians for IDC was similar for both groups (per 100 beds; total: 0.2 [0–0.4], smaller: 0.2 [0–0.6], larger: 0.2 [0–0.2], p=0.392) (per 1000 patient-days; total: 0.1 [0–0.2], smaller: 0.1 [0–0.3], larger: 0.1 [0–0.1], p=0.466). Blood cultures are conducted at in-house microbiology laboratory in most hospitals (n=20, 87%). Gram staining is conducted as soon as blood cultures are identified as positive only in 30% of the facilities. In other hospitals, Gram staining is only conducted on weekdays during the daytime or the technicians’ working time (n=5 [21.7%], respectively).

**Conclusion:** We revealed the baseline data for ID practices in Japan using a novel surveillance system. Parameters such as ID physician numbers seem less satisfactory in Japan compared to those in other countries; further analyses with a large number of hospitals are necessary.

**Table 1 (abstract P127).** The Table shows HH compliance and missed HH actions while gloves are used.

	Hospital-wide (A)	Geriatrics (B)	Orthopedics	Geriatrics (D)
Compliance with hand hygiene (mean ± 95%CI)				
Overall	68.32% (±1.22)	76.97%(±3.39)	71.01% (±6.74)	65.80% (±1.47)
CP	57.12% (±7.0)	61.87% (±15.55)	55.32% (±10.2)	41.21% (±7.53)
SP	68.86% (±1.34)	78.48% (±3.86)	71.35% (±6.77)	66.86% (±1.50)
*p<0.001 for statistical significance between CP and SP*				
Missed HH actions while gloves are used (mean ± 95%CI)				
Overall	16.11% (±2.19) (1201/7457)	23.04% (±2.77) (150/651)	10.66% (±4.86) (69/647)	15.99% (±1.20) (219/1370)
CP	74.19% (±5.64) (342/461)	77.55% (±5.10) (76/98)	76.19%(±%28) (16/21)	82.47% (± 9.98) (80/97)
SP	12.28% (± 1.37) (859/6996)	13.38% (±3.17) (74 /553)	8.47% (±4.10) (53/626)	10.92% (±3.10) (139/1273)

*p<0.001 for statistical significance between CP and SP*

**Disclosure of Interest**: None declared

### P460 PROVIDING A ROADMAP FOR AUTOMATED INFECTION SURVEILLANCE IN EUROPE (PRAISE): RESULTS FROM A SURVEY OF PRACTICES

#### M. Abbas^1^, A. Gomila^2^, A. Johansson^3^, M. B. G. Koek^4^, N. Maldonado^5^, Z. Palacios^5^, S. M. van Rooden^6^, M. S. M. van Mourik^6^ on behalf of Providing a Roadmap for Automated Infection Surveillance in Europe (PRAISE)

##### ^1^Geneva University Hospitals, Geneva, Switzerland; ^2^Hospital Universitari de Bellvitge, Barcelona, Spain; ^3^Umeå University, Umeå, Sweden; ^4^National Institute for Public Health and the Environment (RIVM), Bilthoven, Netherlands; ^5^Hospital Universitario Virgen Macarena, Seville, Spain; ^6^University Medical Center Utrecht, Utrecht, Netherlands

###### **Correspondence:** M. Abbas

**Introduction:** Surveillance of healthcare-associated infections (HAI) is a key component of successful infection prevention programs. Fully automated and semi-automated surveillance (AS) systems for the identification of HAI are being developed in order to improve quality and efficiency of surveillance.

**Objectives:** To describe the current landscape of AS systems in hospitals or networks in European countries.

**Methods:** This study is part of the JPIAMR-funded PRAISE network aiming to provide a roadmap for AS in Europe. An online self-administered survey was sent to network members, a convenience sample of known contacts who have AS systems in place in their hospital or network, and to corresponding authors of publications on existing systems published from 2010 onwards. Snowball sampling was also used.

**Results:** Twenty-four respondents completed the survey in full, of whom 11 work in a hospital, 7 work in a surveillance network, and 4 work in both. Thirteen respondents have an AS system in place, of which 5 were fully automated and 7 were semi-automated (1 unknown). HAIs most frequently under surveillance were surgical site infection (n=10), bloodstream infection (BSI) (n=9), central line-associated BSI (n=8), and urinary tract infection (n=8). The most frequent algorithm applied was a classification model (n=7). Data most frequently need to be extracted from independent databases (n=7), and sometimes can be extracted from data warehouses either fully (n=3) or partially (n=3). Key advantages of AS systems were greater amount of data collected and time efficiency compared to manual surveillance. Key determinants of success were data availability and good collaboration with information technology engineers. The main barriers in implementation were data availability and administrative barriers.

**Conclusion:** Our survey describes characteristics of AS systems in place, as well as preconditions for implementation. Our results may be helpful for hospitals considering implementing AS systems.

**Disclosure of Interest**: None declared

### P461 IMPLEMENTATION OF THE WHO INFECTION PREVENTION AND CONTROL (IPC) CORE COMPONENTS (CC) AT THE NATIONAL LEVEL: A GLOBAL SURVEY

#### E. Tartari^1^, S. Tomczyk^2^, D. Pires^1^, B. Zayed^3^, A. P. Coutinho Rehse^4^, P. Kariyo^5^, W. Zingg^1^, D. Pittet^1^, B. Allegranzi^2^

##### ^1^University Hospital of Geneva; ^2^WHO, Geneva, Switzerland; ^3^WHO, Cairo, Egypt; ^4^WHO, Copenhagen, Denmark; ^5^WHO, Liberville, Gabon

###### **Correspondence:** E. Tartari

**Introduction:** WHO published guidelines on the CC of IPC programmes.

**Objectives:** We aimed at evaluating the current status of national IPC programmes among countries that signed the *Clean Care is Safer Care* pledge.

**Methods:** From June 2017 to November 2018, we conducted semi-structured interviews with selected national IPC focal points (FPs). A target sample size by WHO region and country income was estimated. The questionnaire included six sectionsand 30 questions reflecting the WHO IPC CC: 1) IPC programme; 2) guidelines; 3) education and training; 4) surveillance; 5) multimodal strategies (MMIS); 6) monitoring and feedback. We present here the quantitative results.

**Results:** We interviewed national IPC FPs from 88 countries (20 low-income countries [LIC], 17 lower middle-income countries, 21 upper middle-income countries, and 30 high-income countries. On average, fundamental aspects of the IPC CC reported as implemented ranged from 21.6% to 67.0%. 62.5 % of countries reported having a national IPC programme but only 26.1% have a dedicated budget for IPC. 73% of countries had an AMR national action plan, but IPC was included in only 53%. Significant regional and income differences (p=0.02 and p=0.03) were identified regarding the existence of implementation strategies for guidelines (36.4% of countries on average). Pre- to post-graduate IPC education varied significantly between the regions (35.2%, p=0.005 and 42.1%, p=0.002 respectively). Regional differences were also reported on implemented surveillance systems (46.6%, p < 0.001); understanding of MMIS (51.1%, p=0.002); and monitoring of IPC related indicators (65.9%, p=0.03), with highest proportions in Europe.

**Conclusion:** Our findings demonstrate variations regarding the implementation of IPC CC at national level across countries, with gaps identified in all regions, and large gaps in LICs. Identified gaps should be used to guide future IPC national policies.

**Disclosure of Interest**: None declared

### P462 TESTING OF THE WORLD HEALTH ORGANIZATION (WHO) INFECTION PREVENTION AND CONTROL (IPC) ASSESSMENT FRAMEWORK AT THE ACUTE HEALTH CARE FACILITY LEVEL

#### S. Tomczyk^1^, S. J. S. Aghdassi^2^, J. Storr^1^, S. Hansen^2^, A. Stewardson^3^, P. Bischoff^2^, P. Gastmeier^2^, B. Allegranzi^1^

##### ^1^IPC Global Unit, WHO, Geneva, Switzerland; ^2^Institute for Hygiene and Environmental Medicine, Charité – Universitätsmedizin Berlin, corporate member of Freie Universität Berlin, Humboldt-Universität zu Berlin, and Berlin Institute of Health, Berlin, Germany; ^3^Department of Infectious Diseases, The Alfred Hospital and Monash University, Melbourne, Australia

###### **Correspondence:** S. Tomczyk

**Introduction:** Monitoring and evaluation are an essential part of IPC implementation. We developed an IPC assessment framework (IPCAF) to support the *WHO Guidelines on IPC Core Components* implementation in acute health care facilities. We aimed to evaluate its reliability and usability.

**Objectives:** To ensure that the IPCAF is a reliable and effective tool for global use, we aimed to evaluate its reliability and usability.

**Methods:** The IPCAF is a questionnaire with a scoring system to measure the level of IPC implementation according to the eight WHO core components. The tool was qualitatively pre-tested, revised and selectively translated. A convenience sample of hospitals was invited to participate in the final testing. At least two IPC professionals from each hospital independently completed the IPCAF and a usability questionnaire online. The tool’s internal consistency and inter-observer reliability or intraclass correlation coefficient (ICC) were assessed and usability questions were descriptively summarised.

**Results:** A total of 46 countries, 181 hospitals, and 324 individuals participated; 52 (16%) and 55 (17%) were from low- and lower-middle income countries, respectively. Fifty two percent took less than one hour to complete the IPCAF. Adequate internal consistency and a high ICC (0.92 [95% CI: 0.89-0.94]) was found overall. Ten questions had poor reliability (ICCs < 0.4) and were revised according to usability feedback and expert opinion. The median rating for all usability statements (e.g. ease of use, clarity, usefulness, appropriate time and scoring) was four (“Agree”) from a Likert scale of one (“Strongly disagree”) to five (“Strongly agree”).

**Conclusion:** The WHO IPCAF was tested using a robust methodology in a broad range of countries and finalised based on users’ feedback and reliability assessment. We believe this process has optimised the utility of this tool for IPC situation analysis and improvement in healthcare facilities globally.

**Disclosure of Interest**: None declared

### P463 CROSS-BORDER IMPLEMENTATION OF THE INFECTION RISK SCAN (IRIS) IN 9 HOSPITALS (I-4-1-HEALTH PROJECT)

#### I. Willemsen^1^, M. Verelst^2^, A. Schuermans^2^, J. Kluytmans^1,3^ on behalf of i-4-1-Health study group

##### ^1^AMPHIA HOSPITAL, Breda, Netherlands; ^2^University hospital Leuven, Leuven, Belgium; ^3^Julius Center for Health Sciences and Primary Care, University Utrecht, Utrecht, Netherlands

###### **Correspondence:** I. Willemsen

**Introduction:**

Control of antimicrobial resistance can be improved by creating transparency in the quality of infection control and antimicrobial use.

**Objectives:**

The objective of the i-4-1 Health project is to implement the IRIS in Dutch (NL) and Belgian (BE) hospitals. The IRIS is an standardised, multifactorial tool, based on cross-sectional measurements, to determine the quality of infection control practices and antimicrobial use^1^, in order to improve.

**Methods:**

The IRIS was performed in 32 wards in 9 hospitals. Variables include hand hygiene (HH) performance based on alcohol consumption, environmental contamination (EC) using ATP measurements (ATP Luminometer, 3M), presence of infection control (IC) preconditions, personal hygiene of healthcare workers (HCW), prevalence and appropriateness of indwelling medical devices (MD) and antimicrobial therapy (AMT).

**Results:** The IRIS was performed successfully in all hospitals (n=1598 patients). The prevalence of AMT was comparable in both countries: overall 39% use of AMT and 86% was considered accordance with the local guideline. In both countries, 66% of all patients had at least one MD in situ. Considered unjustified 6% in NL and 13% in BE (p<0.001). A total of 990 ATP measurements were conducted. Median Relative Light Units (RLU) was 189 (range 6-29,613; 13% above 1000 RLU).The median number of handdisinfection moments per patient day was 10. Three hospitals could not deliver alcohol consumption data. No differences were found in IC preconditions and personal hygiene of HCW. Overall, 96.5% (n=656) of all observed HCW were bare below the elbox, not wearing rings, watches or bracelets.

**Conclusion:**

The IRIS was implemented successfully in all hospitals in both countries. A significant difference was observed in the unjustified use of MD. These results provide targets for custom-made interventions and repeated measurements can measure the effect of these interventions. Thereby it can serve as a quality improvement tool for infection control and antimicrobial use.

**References**

1. Willemsen I, Kluytmans J. The Infection Risk Scan (RIS): standardization and transparancy in infection control and antimicrobial use. Antimicrob Resist Infect Control. 2018;7:38

**Disclosure of Interest**: None declared

### P464 INFECTION PREVENTION AND CONTROL PROGRAMS EVALUATIONS: AN EXPLORATORY COMPARATIVE STUDY OF CONTEMPORARY TOOLS

#### M. C. Padoveze^1^, L. M. Abraão^2^, C. N. Júnior^1^, P.-A. Zimmerman^3^

##### ^1^School of Nursing, University of São Paulo; ^2^Americas Medical Services - United Health Group, São Paulo, Brazil; ^3^Griffith University, Gold Coast, Australia

###### **Correspondence:** M. C. Padoveze

**Introduction:** The efficacy of comprehensive Infection Prevention and Control Programs (IPCP) to reduce infection rates is well established. However, the evaluation of these programmes to achieve the best performance remains an issue. In this sense, researchers have worked on developing tools for evaluation IPC.

**Objectives:** To compare three existing IPCP evaluation tools applied in healthcare facilities in Brazil.

**Methods:** Cross-sectional, descriptive, quantitative approach, conducted using three tools concurrently. The tools were: 1.Infection Prevention and Control Programme Evaluation (IPCPE) from Australia; 2.Operating Guides of IPCP indicators (OGIPCP) from Brazil; 3.Assessment tool for hospital IPCP (IPCAF) from World Health Organization. Infection control practitioners (ICP) were recruited using snowball technique. Each ICP applied the tools to their setting. Data collection was performed by using a standardized semi-structured questionnaire, including 35 Likert scale items to inform the feasibility and comprehensiveness of tools.

**Results:** Among invited ICP, 12 participants applied all evaluation tools. The average experience in infection prevention was 8 years (range 1.5 to 15 years). The average time to apply the evaluation tools were: IPCPE: 3h, OGIPCP: 1h, and IPCAF 1.5h. The majority of positive agreements regarding comprehensiveness were obtained by IPCPE (100% of positive agreement in 10 questions), followed by IPCAF (100% of positive agreement in 9 questions). Time spent applying the tools was only considered acceptable for OGIPCP and IPCAF. No tool achieved 100% agreement in 10 questions regarding comprehensiveness to assess the support of microbiology and other services to the IPCP, and links with public health. None of the tools achieved 100% agreement to recommend their use in extra-hospital settings such as primary care.

**Conclusion:** Both IPCPE and IPCAF were considered as more comprehensive, but still lacking potential to access all relevant issues for IPCP. IPCAF and OGIPCP were considered less time consuming. Next, we will perform a qualitative approach to better understand the improvement gaps.

**Disclosure of Interest**: None declared

## Poster session: Public reporting / benchmarking

### P465 TRENDS IN HEALTHCARE ASSOCIATED INFECTIONS AND ANTIMICROBIAL USE IN HOSPITALS, BASED ON POINT PREVALENCE STUDIES IN THE NETHERLANDS FROM 2007-2016.

#### E. Smid^1^, T. Hopmans^1^, J. Wille^1^, M. Koek^1^, S. de Greeff^1^, M. Vos^2^, S. Geerlings ^3^

##### ^1^Department of Epidemiology and Surveillance, National Institute for Public Health and the Environment (RIVM), Bilthoven; ^2^Department of Medical Microbiology and Infectious Diseases, Erasmus University Medical Center, Rotterdam; ^3^Department Internal Medicine: Infectious Diseases, Amsterdam UMC, Amsterdam, Netherlands

###### **Correspondence:** E. Smid

**Introduction:** Healthcare associated infections (HAI) contribute to morbidity and mortality.

**Objectives:** To measure prevalence of HAI and antimicrobial use in hospitals, voluntary national point prevalence surveys (PPS) have been performed in the Netherlands since 2007.

**Methods:** The annual data of PPSs from 2007 until 2016 were analysed for trends in patient characteristics, use of medical devices, use of antibiotics, and presence of HAI on the survey day. Data available concerned all hospitalized patients, except for patients in the day-care unit and psychiatric wards. Analyses were performed using linear and logistic regression.

**Results:** Data were reported for 171,116 patients. Crude annual prevalence of patients with HAI with onset during hospitalization decreased from 6.1% in 2007 to 3.6% in 2016. The Odds Ratio (OR) for trend was 0.92 (95%CI 0.91-0.93) per year. Most prominent trends were seen for surgical site infections (1.6% to 0.7%, OR: 0.31 (0.26-0.38)), urinary tract infections (2.1% to 0.6%, OR: 0.18 (0.15-0.22)) and combined other infections (0.7% to 0.4%, OR: 0.26 (0.19-0.35)). Over the years, the distribution of gender, age and McCabe-score remained stable. The mean length of stay (LOS) decreased from 10 to 7 days. The percentage of patients treated with antimicrobials increased from 31% to 36% (OR: 1.03,(1.02-1.03).

**Conclusion:** PPS-data from 2007-2016 show a decreasing trend in the prevalence of HAI with onset during hospitalization, but also a decreasing LOS, while the percentage of patients using antibiotics increased during these years.

**Disclosure of Interest**: None declared

### P466 BENCHMARKS FOR HEALTHCARE ASSOCIATED INFECTIONS IN SURGICAL PROCEDURES FROM BRAZILIAN HOSPITALS AND INTENSIVE CARE UNITS

#### G. L. E. Souza^1^, A. D. N. Silveira^1^, H. D. D. D. Carvalho^1^, R. F. de Andrade Rocha^1^, C. D. D. M. Oliveira^1^, L. G. Giarola^1^, E. M. M. Leite^2^, B. R. G. M. Couto^3^, E. U. Silva^4^, C. E. F. Starling^5^ on behalf of NOIS Project

##### ^1^Instituto de Ciências Biológicas e da Saúde, Centro Universitário de Belo Horizonte; ^2^Risoleta Tolentino Neves Hospital; ^3^Centro Universitário de Belo Horizonte; ^4^Madre Teresa Hospital; ^5^Lifecenter Hospital, Belo Horizonte, Brazil

###### **Correspondence:** G. L. E. Souza

**Introduction:** Benchmarking is utterly important for healthcare quality assessment. Thus, updated research is necessary in order to create representative data.

**Objectives:** This descriptive, multicentered study provides benchmarks to the southeast population of Brazil and also to similar populations from developing countries.

**Methods:** The NOIS Project uses SACIH, software for hospital infection control (www.sacihweb.com), which retrieves data provided by different Brazilian hospitals. All hospitals comply with prospective Healthcare Associated Infections (HAI) surveillance NHSN/CDC protocols. A variety of 42 types and a total of 189252 surgical procedures, from 11 hospitals and 13 intensive care units (ICU's), were analyzed from 2014 to 2018. Benchmarks were defined as 10^th^, 50^th^, and 90^th^ percentiles (p10, p50, p90) of HAI rates from each type of surgical procedure. Only a small selection from all data was comprised in this abstract.

**Results:** Benchmarks were hereby defined as the pooled mean of the p10, p50, and p90 of HAI rates for each procedure: Cesarean section: 2,1%. Hysterectomy: 1,5%. Cholecystectomy: 1,1%. Herniorrhaphy: 1,3%. Peripheral vascular bypass surgery: 1,2%. Genitourinary surgery: 4,8%. Prostate surgery: 1,0%. Bariatric surgery: 0,9%. Colon surgery: 3,2%. Appendix surgery: 2,2%. Breast surgery: 0,9%. Kidney transplant: 4,0%. Craniotomy: 5,5%. Spinal fusion: 3,4%. Knee arthroplasty: 3,1%. Cardiac surgery: 3,7%. Bile duct, liver or pancreatic surgery: 10,6%. Otorhinolaryngology surgery: 0,6%. Limb amputation: 8,1%. Oral and maxilofacial surgery: 0,3%. Exploratory abdominal surgery: 4,8%.

**Conclusion:** Benchmarks for HAI's have been calculated, and can be used by infection control professionals in Brazil and other developing countries.

**Disclosure of Interest**: None declared

### P467 STATEWIDE SURVEILLANCE SYSTEM FOR SURGICAL SITE INFECTION: RESULTS FROM SIX YEARS AFTER IMPLEMENTATION OF SELECTED PROCEDURES MONITORING

#### D. S. D. Mello^1^, G. Madalosso^2^, D. B. D. Assis^2^, M. C. Padoveze^1^

##### ^1^School of Nursing, University of São Paulo; ^2^Center for Epidemiological Surveillance , State Health Department of São Paulo, São Paulo, Brazil

###### **Correspondence:** D. S. D. Mello

**Introduction:** The healthcare-associated infections (HAI) are recognized as public health problem. Health authorities should establish priorities for HAI surveillance.

**Objectives:** To describe the results of six years of surveillance of surgical site infections (SSI) in São Paulo state, Brazil.

**Methods:** Eleven surgical procedures were selected to be monitored in the SSI surveillance system of the state. Healthcare Facilities (HF) reported data using standardized criteria and through a spreadsheet sent monthly. A descriptive analysis was performed including data from January 2012 to December 2017. The data were aggregate for each year and the percentiles distribution were calculated for each procedure.

**Results:** The number of participating HF increased over the years: 555 in 2012 to 569 in 2017, representing respectively 83% and 89% of eligible HF. The most frequent procedure in the period was cesarean section (n=1,961,504) and the less frequent was colectomy by laparoscopic (LP) (n=16,434). The rates of laparoscopic appendectomy, colectomy, hysterectomy and of mastectomy were zero up to percentile 75; their percentile 90 rates varied along the years ranging, respectively, from 1.1 to 3.6%; 0 to 7.8%; 1.0 to 2.0% and 3 to 5.5%. Craniotomy had 0% SSI rate up to the p50 and varied along the years ranging from 5 to 6.8% at the p75 and from 10 to 14.3% at the p90. The p50 ranged within the years from 0.2 to 0.23% for cesarean section and from 2.5 to 5.2% for coronary artery bypass graft. The p90 for laparoscopic cholecystectomy varied along the years ranging from 0.8 to 1.2%.

**Conclusion:** The results showed high adherence of HF to the statewide surveillance system. The high number of hospitals with rate of zero pointed out flaws in the surveillance performed by the HF. The high rates demand strategies by both the health authorities and HF aiming to reduce the SSI.

**References**

Organización Mundial de La Salud. Prevención de lãs infecciones nosocomiales. Guía Practica. 2ª edición, 2003. Disponível em: http://www.who.int/csr/resources/publications/ES_WHO_CDS_CSR_EPH_2002_12.pdf [acesso: 22/07/2011].

**Disclosure of Interest**: None declared

### P468 BENCHMARKING DEVICE-ASSOCIATED INFECTIONS FOR PREVENTION IN DEVELOPING COUNTRIES: A QUANTITATIVE MULTICENTERED STUDY IN BRAZILIAN HOSPITALS AND INTENSIVE CARE UNITS

#### G. L. E. Souza^1^, A. D. N. Silveira^1^, H. D. D. D. Carvalho^1^, C. D. D. M. Oliveira^1^, R. F. A. Rocha^2^, E. U. Silva^3^, E. M. M. Leite^4^, B. R. G. M. Couto^2^, C. E. F. Starling^5^ on behalf of NOIS Project

##### ^1^Instituto de Ciências Biológicas e da Saúde; ^2^Centro Universitário de Belo Horizonte; ^3^Madre Teresa Hospital; ^4^Risoleta Tolentino Neves Hospital; ^5^Infection Control Center, Lifecenter Hospital, Belo Horizonte, Brazil

###### **Correspondence:** G. L. E. Souza

**Introduction:** Benchmarking is an effective tool for monitoring device-associated infections (DAI). Thus, updated research is necessary in order to create representative data.

**Objectives:** This descriptive, multicentered study provides benchmarks to the southeast population of Brazil and similar populations from developing countries.

**Methods:** The NOIS Project uses SACIH, software for hospital infection control (www.sacihweb.com), which retrieves data provided by different Brazilian hospitals. All hospitals comply with prospective Healthcare Associated Infections (HAI) surveillance NHSN/CDC protocols. A total of 189252 surgical and intensive care unit (ICU) procedures, from 11 hospitals and 13 ICU's, were analyzed from 2014 to 2018. Benchmarks were defined as 10th , 50th , and 90th percentiles (p10, p50, p90) of DAI rates from each type of surgical procedure. Only a small selection from all data was comprised in this abstract.

**Results:** Benchmarks for DAI were here by defined as the pooled mean of the 10^th^, 25^th^, 50^th^, 75^th^, 90^th^ percentiles of the infection rate of each procedure. Utilization ratio was defined by the number of device days divided by the number of patient days: Central line-associated primary bloodstream infections (CLABSI) per 1,000 central line-days: 6,5%. Ventilator-associated pneumonias (VAP) per 1,000 ventilator-days: 8,8%. Urinary catheter-associated urinary tract infections (CAUTI) per 1,000 urinary catheter-days: 3,6%. Central line utilization ratio: 47%. Ventilator utilization ratio: 39%. Urinary catheter utilization ratio: 55%.

**Conclusion:** VAP presented higher rates of DAI in Brazilian hospitals. This study also calculated benchmarks, which can be used for healthcare quality assessment in developing countries.

**Disclosure of Interest**: None declared

### P469 STATEWIDE SURVEILLANCE SYSTEM FOR ENDOPHTHALMITIS AFTER INVASIVE INTRAOCULAR PROCEDURES: PRELIMINARY DATA OF AN IMPLEMENTATION STUDY CASE

#### R. A. Luz^1,2^, D. B. Assis^3^, G. Madalosso^3^, S. Timmons^4^, M. C. Padoveze^2^

##### ^1^School of Nursing, Santa Casa de São Paulo School of Medical Sciences; ^2^School of Nursing, University of São Paulo; ^3^Center for Epidemiological Surveillance , State Health Department of São Paulo, São Paulo, Brazil; ^4^Business School, Nottingham University, Nottingham, United Kingdom

###### **Correspondence:** M. C. Padoveze

**Introduction:** Endophthalmitis is one of the most severe complications after invasive intraocular procedures (IIP), whose magnitude is unknown in Brazil due to the lack of a surveillance system.

**Objectives:** To assess the implementation process of a surveillance system for endophthalmitis after invasive intraocular procedures

**Methods:** *Study design*: implementation research, prospective case study, using the Consolidated Framework for Implementation Research (CFIR). *Context*: São Paulo State, Brazil. The implementation strategy was developed in partnership with the State Health Department (SHD). The study was carried out in five phases: 1) Design of the surveillance system (SIVEN); 2) Context assessment; **3)** Recruitment & training of pilot phase participants; **4)** Data analysis: adherence and endophthalmitis rates; **5)**: Evaluation & scaling up. Eligible settings were Healthcare Facilities (HF) which undertook IIP in the State. SIVEN includes a monthly report of number of cataract surgeries, intravitreal injections, and endophthalmitis cases. After the pilot phase (16 months), implementation strategy adjustments were carried out and scaling up proceeded.

**Results:** The adherence rate in pilot phase was 7.0% (31/443); with the number of HF participants rising steadily, from 10 to 31. The number of IIP performed was 62,445; endophthalmitis incidence rate: 0.05% (n=30). The context assessment showed failures in infection prevention standards among HF. Barriers identified during the pilot phase were: lack of reliable data in National Registry of HF to detect eligible settings, poor regularity in data sending by participants, and low adherence rate. This is an ongoing project: During the current scaling up phase, another 18 HF began to participate.

**Conclusion:** The implementation process of SIVEN has been shown to be complex. Detecting barriers and enablers provided useful information for the scaling up phase, including the development of strategies to raise infection prevention awareness and enhancing adherence to the system.

**Key words:** Implementation Science, Surveillance, Endophthalmitis, Nursing

**Disclosure of Interest**: None declared

## Poster session: Pediatrics Settings

### P470 PREVALENCE AND RISK FACTORS OF CARBAPENAMASE PRODUCING ORGANISMS (CPO) IN NEONATES AT A MARTERNITY HOSPITAL IN THE SOUTH OF VIETNAM

#### V. K. Nguyen^1^, E. Athan^2^, T. H. Phan^1^ on behalf of An HQ3, Stephanie J4, Hang TTT5, Tiên TTB6, Phuong KTD7, Thang DT7; Thang QV8, Duy KN8; 3.P&CI Dept of HV hospital, Gynecologist; 4.ID Physician, Service Director, General Medicine at Monash Health

##### ^1^Prevention & Control of Infection, Hung Vuong hospital, Ho Chi Minh, Viet Nam; ^2^Department of Infectious Disease (GCEID), Barwon Health, Melbourne, Australia

###### **Correspondence:** V. K. Nguyen

**Introduction:** Carbapenemase producing organism (CPO) pose major challenges for prevention, control and treatment of the infections. We aimed to determine the prevalence and risk factors of CPO detected in Neonates in NICU in Maternity hospital, in Vietnam

**Objectives:** are to determine the prevalence and risk factors of CPO detected in Neonates in NICU in Maternity hospital, in Vietnam.

**Methods:** a prospective cross-sectional study included 83 neonates chosen randomly. A **rectal swab** was used to collect samples. To screen for CPOs we used **MELAB Chromogenic CARBA** disks. We then identified the CPO and the relevant antibiogram using the **BD Phoenix automated direct identification system**. SPSS software version 18.0 was used to analyze the demography characteristics and prevalence of CPO, risk factors associated with CPO and the adjusted odds ratio with a 95% confidence interval was considered significant. Statistical significance was set at P < 0.05.

**Results:** The prevalence of CPO was 48.2% (n= 83) screened with MELAB Chromogenic CARBA disks and 42.2 % (n=83) with BD Phoenix automated system. The majority of CPOs were E Coli of which 27 had an identical antibiogram profile. Risk factors for CPO colonization included neonatal age (days) (OR: 26 (7.891 - 85.664), prior exposure to antibiotics (OR: 18.764 (5.829 - 60.405), the length of hospital stay (OR: 19.5 (4.197 - 90.602). Other risk factors were associated with procedures performed on neonates. We identified nosocomial cross infection was significant.

**Conclusion: Nosocomial cross-infection** associated with CPO was detected in this study. Preventing & controlling CPO should be an urgent priority at the Neonatal department in HV hospital. The authors declare that they have no conflict of interest.

**References**

**Disclosure of Interest**: None declared

### P471 Withdrawn

### P472 BUILDING AND IMPLEMENTING AN INFECTION PREVENTION AND CONTROL COURSE FOR A GLOBAL AUDIENCE

#### M. A. Caniza, M. Homsi, B. Happ, C. Rodriguez Galindo

##### Global Pediatric Medicine, St Jude Children's Research Hospital, Memphis, United States

###### **Correspondence:** M. A. Caniza

**Introduction:** Preventing and controlling healthcare-associated infections and the emergence of multidrug-resistant organisms are essential components of safe healthcare. A core component of the WHO guidelines for infection prevention and control (IPC) programs is the training of professionals in infection control.

**Objectives:** Describe how we built and implemented a comprehensive training curriculum for infection preventionists.

**Methods:** An assessment was conducted among attendees at the 2018 St. Jude Global Alliance meeting to ascertain needs for training in IPC. The goals, objectives, and educational strategies for the course design, content, certification, implementation, and evaluation were outlined. Basic statistics were used to report the results.

**Results:** Of the 150 healthcare providers for children with cancer, representing 50 countries, attending the St. Jude Global Alliance meeting, 45 participated in a 17-question survey on IPC and the adequacy of care delivery at their institutions. Deficiencies noted were a lack of IPC programs, policies and procedures, personnel competent in IPC, and knowledge of infections, as well as poor hand hygiene infrastructure and poor adherence to IPC practices essential for safe care. As a first step towards improvement, we designed a global IPC training program for professionals. The training program comprised 8 weeks of distance learning and 1 week of in-person training. The distance learning consisted of 4 major modules covering infectious processes, basic statistics and epidemiology, infection prevention procedures, and program management. The residential training provided hands-on practice in conducting HAI surveillance and writing policies and procedures. In the inaugural course, 62 participants representing 19 countries attended the distance learning sessions. The knowledge gain, as assessed through module-based pre/post examination, was significant (*P* < 0.001). Thirty-three top participants were selected for residential training, which was delivered at regionally targeted, collaborative sites. Attendees expressed a high level of satisfaction with the training, and many have indicated their intention to apply the learned concepts at their own institutions.

**Conclusion:** We have demonstrated the feasibility of a flexible, comprehensive, and effective IPC course designed to build sustainable local expertise to manage IPC programs.

**Disclosure of Interest**: None declared

### P473 IDENTIFYING INFECTION PREVENTION AND CONTROL GAPS CONTRIBUTING TO HEALTHCARE-ASSOCIATED NEONATAL SEPSIS IN RESOURCE-LIMITED SETTINGS: RESULTS FROM A MODIFIED DELPHI PROCESS

#### D. Yee^1^, J. Weiss^1^, H. Osuka^1^, J. Kriengkauykiat^2^, S. Coffin^3^, J. Hopman^4^, J. Johnson^5^, P. Ram^6^, F. Serbanescu^1^, P. Srikantiah^7^, B. Park^1^

##### ^1^CDC; ^2^Grady Health Systems, Atlanta; ^3^Children's Hospital of Philadelphia, Philadelphia, United States; ^4^Radboud University Medical Center, Nijmegan, Netherlands; ^5^Johns Hopkins University, Baltimore; ^6^USAID, Washington DC; ^7^Bill and Melinda Gates Foundation, Seattle, United States

###### **Correspondence:** D. Yee

**Introduction:** In resource-limited settings (RLS), healthcare-associated neonatal sepsis is associated with high morbidity, mortality, length of hospital stay, and healthcare cost. Understanding infection prevention and control (IPC) gaps contributing to risk in healthcare settings can help identify interventions.

**Objectives:** Identification of IPC gaps contributing to healthcare-associated neonatal sepsis in RLS.

**Methods:** We conducted a literature review to identify studies describing IPC gaps contributing to healthcare-associated neonatal sepsis in RLS neonatal intensive care units (NICU) and interventions to reduce risk. Full-text articles published in English from 1990 to 2018 focused on neonatal infections in NICUs were included. A panel of global experts in neonatology and IPC participated in a modified Delphi process to review literature review findings, discuss experiences and achieve consensus on critical IPC priorities.

**Results:** Among 2,325 articles, we reviewed 114 articles; 87 identified relevant risk factors. Most frequent risk factors included device use (i.e. ventilators, central lines), inadequate hand hygiene, and inadequate injection safety. An additional 27 articles described IPC interventions to address neonatal infection, including multimodal strategies and hand hygiene compliance. The modified Delphi process identified 13 priority IPC gaps: patient safety culture; hand hygiene; nurse-to-neonate ratio; alcohol-based hand rub at point of care; neonate co-bedding; sterilization and reprocessing of multi-use items; reuse of single-use items; inadequate cleaning of non-critical items; facility exceeding capacity; lack of running water, sinks, soap and paper towels; sterile compounding of medications; aseptic technique for device insertion and device maintenance and care.

**Conclusion:** This literature review and modified Delphi process identified important IPC gaps leading to healthcare-associated neonatal sepsis. Prioritizing IPC interventions and strategies for these areas may reduce neonatal sepsis in RLS.

**Disclosure of Interest**: None declared

### P474 MOLECULAR DIAGNOSTICS AS A TOOL FOR ACTIVE SURVEILLANCE OF ANTIMICROBIAL RESISTANCE IN A PEDIATRIC INTENSIVE CARE UNIT (PICU)

#### E. Iosifidis^1^, M. Simitsopoulou^1^, A. Giampani^1^, E. Chorafa^1^, S. Kalamitsou^2^, P. E. Mantzafleri^2^, E. Chochliourou^2^, M. Svirkos^2^, M. Sdougka^2^, E. Roilides^1^

##### ^1^3rd Pediatric Department, Aristotle University of Thessaloniki; ^2^Pediatric intensive care unit, Hippokration Hospital of Thessaloniki, Thessaloniki, Greece

###### **Correspondence:** E. Iosifidis

**Introduction:** Infections caused by highly resistant bacteria (HRB) in patients of Pediatric Intensive Care Units (PICU) significantly increase morbidity, length of stay, mortality rates and costs.

**Objectives:** The aim of this project was to design and implement an active surveillance clinical protocol as part of a pilot study in order to develop high clinical value molecular diagnostics directly to clinical samples as a guide for monitoring antimicrobial resistance in a PICU endemic for HRB.

**Methods:** This study was conducted in an 8-bed PICU located in a tertiary level general hospital. Patients hospitalized for at least 7 days were included. Stool samples were collected between July 2018 and February 2019 and stored at -80^o^C until processing by PCR. The burden of resistance to antibiotics was assessed using PCR following DNA isolation directly from stool samples. The presence of four carbapenemases: blaKPC, blaOXA-48, blaVIM, and blaNDM as well as of blaTEM and blaSHV were evaluated. A cumulative “resistome” report was distributed to healthcare personnel on a daily basis.

**Results:** Stool samples were processed from 36 patients. In 18 of them (50%), at least one carbapenemase was detected: 7 patients carried blaKPC gene, 5 blaVIM and 6 both blaKPC and blaVIM. blaOXA-48 and blaNDM were not detected. The blaTEM was detected in 22 patients and half of them co-carried blaVIM and blaKPC. The blaSHV was detected in 12 patients, six of them co-carried blaKPC and blaVIM. During the last two months, blaKPC fecal carriage in PICU patients was reduced.

**Conclusion:** Direct implementation of a targeted and customized rapid molecular detection assay to clinical samples was effective to recognize the burden of bacterial resistance in a clinical setting endemic to resistant bacteria. Molecular antimicrobial resistance surveillance combined with prompt notification of clinical staff on patients’ colonization status, may have contributed in the reduction of KPC-carrying strains in this study.

**Disclosure of Interest**: None declared

### P475 IMPACT OF A PEDIATRIC INFECTIOUS DISEASE SPECIALIST FOR THE MANAGEMENT OF BACTEREMIA IN CHILDREN

#### M. Remillieux^1^, J. Arata-Bardet^1^, C. Masson^2^, P. Pavese^3^, Y. Caspar^4^, C. Recule^4^, D. Plantaz^1^, J. P. Stahl^3^, C. Bost-Bru^1^, C. Landelle^2^

##### ^1^Department of pediatrics; ^2^Infection control unit; ^3^Department of infectious disease; ^4^Laboratory of bacteriology, CHU Grenoble Alpes , Grenoble, France

###### **Correspondence:** C. Landelle

**Introduction:** Empiric antibiotics should be started rapidly when bacteremia is suspected in children, and then adapted to the isolated bacteria. Bacteremia monitoring is a part of antimicrobial stewardship programs, developed by infectious diseases specialists (IDS) in order to optimize antibiotic use.

**Objectives:** The aim of our study was to evaluate the impact of advices by a pediatric IDS for the management of children presenting with positive blood culture.

**Methods:** We performed a monocentric retrospective study. In our university hospital, all positive blood cultures are monitored since January 2016. Everyday microbiologists enter all positive blood culture results into a database. The pediatric IDS is informed and can confirm or change the patient’s treatment. He reports in the database his intervention (several advices can be given in one intervention) and the compliance to advices by physicians in charge of the patient.

**Results:** A total of 130 positive blood cultures were reported between July 1, 2016 and June 30, 2017. The contamination rate of these blood cultures was 1.3%. Advice was given by a pediatric IDS for 100 positive blood cultures (71 bacteremia and 29 contamination). Gram-positive Cocci were present in 63% and Gram-negative Bacilli in 37% of bacteremia respectively. Catheter-related bloodstream infections were 48% of bacteremia. Pediatric IDS gave an advice in 53% of cases. In 75% of advices, antibiotics prescriptions were modified or adapted. In case of adaptation, antibiotics were initiated in 11% of cases and stopped in 7% of cases. Further investigations were required in 32% of cases. In 15% of cases, pediatric IDS recommended a change or a removal of the catheter involved in the bacteremia. Advice was followed by physicians in 93% of cases.

**Conclusion:** This study demonstrates that the systematic intervention of a pediatric IDS is useful in pediatrics wards, resulting in optimization of the treatment. Physicians complied with IDS advices, that is a major point.

**Disclosure of Interest**: None declared

## Poster session: Public Health and Patient Safety

### P476 PATIENT SAFETY AT HEALTH FACILITIES IN INDONESIA

#### A. Ainy, M. Misnaniarti, D. Safriantini

##### Public Health, Sriwijaya University, Palembang, Indonesia

###### **Correspondence:** A. Ainy

**Introduction:** Patient safety is a public health concern that affects patients in all developed or developing countries. Healthcare errors associate to patient safety practices. Therefore patient safety is the foundation of high-quality health care.

**Objectives:** This study aimed to systematically review the literature on the patient safety practices at health facilities in Indonesia.

**Methods:** The PubMed, Google Scholar, DOAJ databases search was set to find recently published papers within the last ten years (between 2010 and 2019). The authors also searched and selected all studies related to patient safety practices at health facilities from Indonesian journals that indexed by IPI or SINTA1-4. The articles were assessed and analyzed in term of coordination in patient safety program, safety culture, and association between patient safety and quality of healthcare.

**Results:** The type of coordination between units at health facilities in Indonesia that is most identified is pooled interdependence, safety-related perception of health care providers was the most important thing to promote safety culture at health facilities, and patient safety practice has a potential to improve the patients’ perceptions of the healthcare quality.

**Conclusion:** In conclusion, the results of this study highlight the need for good coordination among units at health facilities in implementing patient safety program The results also suggest the need for designing strategies to effectively implement culture of safety, which can consequently influence the healthcare quality.

**Disclosure of Interest**: None declared

### P477 MAPPING THE HEALTHCARE STRUCTURES AND THE PRACTICE OF GUIDELINES FOR INFECTION PREVENTION AND CONTROL STAFF IN THE DUTCH-GERMAN BORDER REGION IN 2016

#### A. Jurke^1^, C. Glasner^2^, D. Rocker^3^, I. Daniels-Haardt^4^, A. W. Friedrich^5^

##### ^1^Infectious Disease Epidemiology, NRW Centre for Health, Bochum, Germany; ^2^Department of Medical Microbiology and Infection Prevention, University Medical Center Groningen, Groningen, Netherlands; ^3^Department Communicable Diseases, The Governmental Institute of Public Health of Lower Saxony, Hannover; ^4^Department Health Protection, Health Promotion, NRW Centre for health, Bochum, Germany; ^5^Department for Medical Microbiology, University Medical Center Groningen, Groningen, Netherlands

###### **Correspondence:** A. Jurke

**Introduction:** Since 2011, EU patients have a right to seek healthcare anywhere in the EU. Little is known on the influence of differences in acute healthcare structures on infection prevention. Moreover, guidelines and realization of those for infection prevention and control (IPC) staff differ and might impact the quality of healthcare.

**Objectives:** We performed an analysis of those parameters in the Dutch-German border region in order to indicate differences and similarities.

**Methods:** For the German border region hospitals (GBH) we extracted annual quality records of hospitals from the Federal Joint Committee. For the Dutch border region hospitals (DBH) the data was requested at press offices of hospitals. We used population statistics and survey data in fulltime equivalents on staff in GBH in 2015.

**Results:** There are more than 4 times more hospitals, hospital beds and inpatient cases per inhabitants in the German than in the Dutch region. Both guidelines recommend similar number of IPC staff. Twenty out of 29 DHB (76.9%) fulfill the Dutch IPC staff guideline for infection control doctors (ICD), 23 (88.5%) for infection control nurse (ICN). Nine out of 35 GBH (25.7%) adhere to the German IPC staff guideline for ICD. All GBH have at least one ICD as an external consultant; 21 (60.0%) have enough ICN. Estimating the actual numbers of IPC staff in DBH with the German guideline, 21 DBH (80.8%) fulfil them for ICD and 19 (73.1%) for ICN. If the Dutch IPC staff guideline would be valid in GBH, 14.5% for ICD and 40.0% for ICN would fulfil it.

**Conclusion:** The observed large differences in acute healthcare structures in the Dutch-German border region cannot be explained by the different population density. There are more and smaller hospitals in Germany hampering the employment of sufficient IPC staff. The fourfold more inpatient cases per inhabitants in the German region enhance the exposure to healthcare, antibiotics and MDRO. Co-operation in education of IPC staff and recognition of degrees could facilitate closing the gap of supply and demand in IPC staff at both sides.

**Disclosure of Interest**: None declared

### P478 WEBQUEST AS INNOVATIVE EDUCATION TOOL FOR INFECTION PREVENTION: BRAZILIAN EXPERIENCE ON IMPLEMENTATION

#### R. M. Figueiredo^1^, I. P. B. D. Passos^1^, M. C. Padoveze^2^, S. Timmons^3^

##### ^1^Nursing Department, Federal University of Sao Carlos, São Carlos; ^2^School of Nursing, University of São Paulo, São Paulo, Brazil; ^3^Business School, The University of Nottingham, Nottingham, United Kingdom

###### **Correspondence:** R. M. Figueiredo

**Introduction:** Implementation science is helpful to overcome the challenge of translating scientific evidence into clinical practice. This study presents preliminary data from a Webquest (WQ) implementation project aiming to enhance knowledge on infection prevention in primary health care (PHC). WQ is an innovative, inquiry-oriented lesson format in which most information comes from the web, but we have adapted it for use off-line since in PHC internet access is frequently poor.

**Objectives:** To evaluate the feasibility of the WQ strategy and analyze the local health managers opinion about viability of this strategy in PHC.

**Methods:** Settings: PHC units in a countryside city of Brazil. Design: Mixed method. A quantitative approach assessed the feasibility of the WQ strategy with 50 members of PHC nursing teams. A qualitative approach assessed the perception of three local health managers through interviews and content analysis.

**Results:** The mean time spent to perform the WQ was 32.5min. Most of participants (96%; 48/50) considered the activity easy and pleasant. Managers thought infection prevention was an important issue, but it was not a priority for them. They pointed out potentially positive influential stakeholders, such as the nursing supervisor and the municipal continuing education staff. The enablers of the implementation process were the feasibility of performing WQ during journeys to work and the low technology requirement. The barriers were the lack of effective continuing education on infection prevention; a perception of devaluation and demotivation of personnel; understaffing; previous experience with research that had not changed clinical practice.

**Conclusion:** The WQ has potential as an effective and innovative tool for infection prevention in PHC. There is a need for strategies to raise the priority of infection prevention on the PHC agenda. The identification of potential positive influential stakeholders, enablers and facilitators will support the next step of the implementation process, increasing the chance of success in the transfers of scientific evidence to clinical practice.

**Disclosure of Interest**: None declared

## Poster session: Disinfection / sterilization / environment 2

### P479 EVALUATION OF THE EFFECTIVENESS OF LIQUID HYDROGEN PEROXIDE ON SURFACE CONTAMINATION

#### C. Hidalgo-Lopez^1^, C. González Juanes^1^, M. Posso^1^, M. Martín Sánchez^1^, R. Bover González^2^, A. Ramírez Marinero^3^, L. Martínez Muñoz^4^, N. Saavedra Martí^4^, Y. Taouil Sbay^5^, N. Martín Sánchez^1^, E. Padilla León^3^

##### ^1^Epidemiology & Evaluation Department; ^2^Hotel Services Department, Hospital del Mar_Parc de Salut Mar; ^3^Microbiology Department, Laboratori de Referència de Catalunya; ^4^Urology and Traumatology Department; ^5^Nephrology Department, Hospital del Mar_Parc de Salut Mar, Barcelona, Spain

###### **Correspondence:** C. Hidalgo-Lopez

**Introduction:** Sodium hypochlorite and quaternary ammonium plus amines are used as surfaces disinfectants. However, new improved hydrogen peroxide disinfectants are a promising alternative.

**Objectives:** We assessed the effectiveness of liquid hydrogen peroxide (Oxivir H+®) on reducing surface contamination.

**Methods:** We evaluated the cleaning and disinfection effectiveness of liquid hydrogen peroxide on five high-touch hospital room surfaces (upper and lower surfaces of the food table, call botton, toilet flap, and bed center) in two hospitalization units (one surgical and one predominantly medical) at the University Hospital del Mar, Spain. For each surface, both adenosine triphosphate (ATP) and aerobic colony counts (ACC) measurements were collected before and after cleaning. Mann–Whitney U test was used for assessing medians differences in the ATP and ACC. Stratified analysis by hospitalization unit, isolated status and contact surface was performed.

**Results:** Overall, we evaluated 161 surfaces. Compared to post-cleaning values, pre-cleaning surfaces showed a higher median of both ATP relative light units (RLU) as well as ACC (310 vs. 55 P=0.000, and 54 vs. 24 P=0.000, respectively). Surfaces in the medical unit presented higher pre-cleaning RLU rates than in the surgical one but in both cases a significant reduction was achieved after cleaning (from 325 to 71 P=0.000 and from 245 to 35 P=0.000). Notably, not isolated patient’s room’s surfaces revealed higher median pre-cleaning RLU and ACC values than isolated patient’s room’s surfaces. In both cases, a significant reduction after cleaning was attained.

**Conclusion:** Our results suggest high effectiveness of liquid hydrogen peroxide for cleaning and disinfection of high-touch hospital room surfaces. Further comparison with the standard cleaning care is needed.

**Disclosure of Interest**: C. Hidalgo-Lopez Grant/Research support from: This study was partly funded by Diversey®. The authors did not receive direct funding from the sponsor., C. González Juanes Grant/Research support from: This study was partly funded by Diversey®. The authors did not receive direct funding from the sponsor., M. Posso Grant/Research support from: This study was partly funded by Diversey®. The authors did not receive direct funding from the sponsor., M. Martín Sánchez Grant/Research support from: This study was partly funded by Diversey®. The authors did not receive direct funding from the sponsor., R. Bover González Grant/Research support from: This study was partly funded by Diversey®. The authors did not receive direct funding from the sponsor., A. Ramírez Marinero Grant/Research support from: This study was partly funded by Diversey®. The authors did not receive direct funding from the sponsor., L. Martínez Muñoz Grant/Research support from: This study was partly funded by Diversey®. The authors did not receive direct funding from the sponsor., N. Saavedra Martí Grant/Research support from: This study was partly funded by Diversey®. The authors did not receive direct funding from the sponsor., Y. Taouil Sbay Grant/Research support from: This study was partly funded by Diversey®. The authors did not receive direct funding from the sponsor., N. Martín Sánchez Grant/Research support from: This study was partly funded by Diversey®. The authors did not receive direct funding from the sponsor., E. Padilla León Grant/Research support from: This study was partly funded by Diversey®. The authors did not receive direct funding from the sponsor.

### P480 STRONG REDUCTION OF ENVIRONMENTAL CONTAMINATION IN HOSPITALS IN THE DUTCH/BELGIAN BORDER AREA AS A RESULT OF IMPROVED CLEANING BASED ON ATP MEASUREMENTS

#### A. Van Arkel^1^, I. Willemsen^1^, P. De Waegemaeker^2^, I. Leroux-Roels^2^, M. Verelst^3^, J. Kluytmans^1^ on behalf of i-4-1 Health Study Group

##### ^1^Microvida Laboratory for Microbiology, Breda, Netherlands; ^2^Universitair ziekenhuis Gent, Gent; ^3^Universitair ziekenhuis Leuven, Leuven, Belgium

###### **Correspondence:** A. Van Arkel

**Introduction:** Measurement of hospital cleanliness is mostly conducted by visual inspection. However, visual inspection is subjective and not very sensitive. Environmental contamination can also be quantified by the measurement of adenosine triphosphate (ATP), a molecule which is present in all organic cells. The measured ATP is expressed in Relative Light Units (RLU), which correlates with the amount of organic material.

**Objectives:** The objective of this study was to determine the effects of improved cleaning based on ATP measurements in hospitals in the Dutch/Belgian border area (as a part of the i-4-1 Health project).

**Methods:** ATP measurements (ATP Luminometer, 3M) were conducted using a standardized methodology in 9 hospitals in 2 to 4 wards, depending on the hospital size. In each ward 30 pre-defined fomites were tested. Four different fomite groups were defined: medical devices, patient bound materials, ward bound materials and sanitary items. Cross-sectional measurements were conducted at two points in time, with a one year interval. After the first round of ATP measurements, improvements of cleaning were implemented based on the results of ATP-data (focus on cleaning of the items with the highest RLU values). Furthermore, responsibilities for specific cleaning tasks were more clearly assigned.

**Results:** In total 1950 ATP measurements were conducted (960 and 990 respectively). The overall median RLU-value in the first round of ATP measurement was 577, in the second round 189 (P<0.001). The median RLU-value in the patient bound materials group in the first round was 937, in the second round 225 (P<0.001). The median RLU-value in the medical devices group in the first round was 663 and in the second round 187 (P<0.001). The median RLU-value in the ward bound materials group in the first round was 626, in the second round 293 (P<0.001). The median RLU-value in the sanitary items group in the first round was 415, in the second round 133 (P<0.001).

**Conclusion:** There was a strong and highly significant difference between the first and second round of measurements. ATP measurements provide specific information of surface contamination and thereby they are useful tools for improvement of cleaning in hospitals.

**Disclosure of Interest**: None declared

### P481 PRACTICE-LIKE VIRUCIDAL EFFICACY TESTING OF DISINFECTANT WIPES IN THE 4-FIELD-TEST

#### S. Pahl, J. Steinmann, B. Becker, D. Paulmann, B. Bischoff, F. Brill

##### Dr. Brill + Partner GmbH, Hamburg, Germany

###### **Correspondence:** F. Brill

**Introduction:** The use of wipes in hospitals and other medical settings is continuously increasing. These wipes should be able to inactivate all types of microorganisms on environmental surfaces thus preventing transfer from dirty to clean areas. The European norm (EN) 16615:2015 describes the killing and possible transfer of bacteria and fungi by disinfectant wipes by movement of the wipe from the contaminated field 1 to fields 2-4 and back to the starting point (4-field test).

**Objectives:** In the existing EN 16615 no virucidal activity is measured. Therefore, it was the aim of our study to include test viruses in the 4-field test allowing a virucidal claim of disinfectant wipes.

**Methods:** The 4-field test was performed with four commercially available disinfectant wipes. The active solutions of these wipes were included. Adenovirus (AdV) type 5, murine norovirus (MNV) as surrogate of human noroviruses, and polyomavirus SV40 (SV40) as surrogate of papillomaviruses served as test viruses representing three stable, non-enveloped test viruses.

**Results:** One wipe based on PAA was able to inactivate all three test viruses resulting in a four log10 reduction on test field 1, whereas two QAC-based products failed to reach such reduction. Both QAC-based wipes were able to inactivate SV40. Only the active solution of one QAC-based wipe and not the wipe itself was effective against MNV. The fourth wipe with 2-propanol as active ingredient showed no efficacy against all three test viruses. In general, there was a good agreement between the results of the wipes and the corresponding fluids showing no influence of the material of wipes. Tests with the PAA-based wipe showed no transfer of all three test viruses to the non-contaminated test fields 2-4. SV40 was transferred by one QAC-based wipe to these additional fields. In all other cases no virus transfer to test fields 2-4 was observed.

**Conclusion:** The successful performance of a 4-field test with viruses demonstrated that the existing wiping method with bacteria and fungi can be used with viruses for measuring virucidal efficacy. The virus-inactivating properties of disinfectant wipes could be evaluated therefore with a test simulating practical conditions with mechanics. With the 4-field test more reliable data for wipes than the existing quantitative suspension tests and/or a carrier test without any mechanics are possible.

**Disclosure of Interest**: None declared

### P482 FEASIBILITY OF LOCAL CHLORINE PRODUCTION FOR SURFACE DISINFECTION IN MALI HOSPITAL AND GABRIEL TOURE HOSPITAL IN BAMAKO (MALI).

#### A. T. Traore^1^, L. Bengaly^2^, R. SANOGO^3^, B. KOURIBA^4^

##### ^1^Hôpital du Mali, Pharmacie Hospitalière; ^2^Pharmacie Hospitalière, CHU Gabriel Touré; ^3^Medecine traditionnelle, INRSP; ^4^CICM, CICM, Bamako, Mali

###### **Correspondence:** A. T. Traore

**Introduction:** In the context of prevention of nosocomial infections, we proposed to study local production of chlorine, to be used for disinfection of surfaces. As part of our PhD thesis, we explored feasibility of using this technique to overcome diffusion of harmful germs.

**Objectives:** To study feasibility of the local production of chlorine by electrolysis with the WATA® device for disinfection of surfaces of operating theatres and delivery rooms in two University Hospitals of Mali.

**Methods:** This was a descriptive study initiated in October 2017 for a period of three years at Mali Hospital and Gabriel TOURE Hospital. WATA® technology was used for local production of chlorine by electrolyse, transforming a salt water solution into sodium hypochlorite with a concentration of 6 g / l.

**Results:** We have trained 46 Operating Room Nurses, Midwives and Surface Technicians on different methods of chlorine dilution. Also, 20 Technicians of Labo-Pharmacy and Technicians of Hygiene were trained in techniques of sampling as well as in local production of chlorine. A production of 420 litres of chlorine of controlled quality has been conducted. We noted that cost of buying one litre of bleach is 500 FCFA on Malian market against 34 FCFA for local production. Before use of locally produced chlorine, 56 samples were taken from operating theatres for microbiological analysis. We found that 35.71% of samples had pathogenic germs such as: Sphingomonas paucimobilis, Klebsiela pneumoniae, Enterococcus spp, Pantonea spp, Neisseria animaloris/zoodegmatis and Acinetobacter baumanni complex. After using local chlorine, 23.30% of samples had pathogenic germs including Sphingomonas paucimobilis, Staphylococcus saprophyticus and Staphylococcus aureus. Data collection and results analysis continue.

**Conclusion:** Our preliminary results show feasibility of local production of low-cost chlorine for disinfection of surfaces of operating theatres and delivery rooms. After using local chlorine contamination rate decreased by 34.75%. Use of locally produced chlorine could contribute to prevention of care-associated infections.

**Disclosure of Interest**: None declared

### P483 EFFICACY TESTING OF HOCL AS A DISINFECTANT FOR HIGH-RISK HPV.

#### L. Robins^1^, C. Meyers^2^, J. Milici^2^, J. Williams^3^, R. Robison^4^

##### ^1^Physical Science Division, School of STEM, University of Washington Bothell, Bothell; ^2^Department of Microbiology and Immunology, Penn State College of Medicine, Hershey; ^3^Briotech Inc., Woodinville; ^4^Department of Microbiology and Molecular Biology, Brigham Young University, Provo, United States

###### **Correspondence:** C. Meyers

**Introduction:** High-risk human papillomavirus (HPV 16 & 18) are the causative agents of cervical, anal, and oropharyngeal cancers. Endocavitary ultrasound devices are used in healthcare facilities to examine the cervix, anal canal, and oropharynx; the three areas commonly infected by high-risk HPV. Our previous studies using suspension and carrier tests demonstrated poor efficacy of widely used hospital disinfectants, including GTA (2.4% and 3.4%) and OPA (0.55%) in inactivating high-risk HPV. The potential for iatrogenic transmission in the healthcare environment from inadequate disinfection practices has become an area of contention and discussion. Risk potentials have commonly been based on outdated criteria or inadequately performed studies. There is a need for effective, convenient chemical decontamination measures.

**Objectives:** To determine the efficacy of exposure to a pure hypochlorous acid preparation (from Briotech Inc.) for inactivation of HPV 16 & 18 using a validated approach based on treatment of high amounts of authentic infectious virus, and well-established methods of replication and testing of high-risk HPVs.

**Methods:** Infectious stocks of HPV16 and HPV18 were produced, titered, and infectivity-tested as previously described. Both suspension and carrier tests were performed with contact times spanning 15 seconds to 20 minutes. Following contact any remaining HOCl was neutralized using 0.1% Tween 80; 1% peptone; 1% cysteine; 0.5M Tris buffer (pH 7.5). Residual HPV was isolated and measured by our published infectivity methods.

**Results:** All HOCl treatment times produced >99.99% reduction in infectivity of HPV16 and HPV18, comparable to the efficacy of 0.87% sodium hypochlorite. Exposure of cell fractions enriched by vector expression of L1 and L2 capsid proteins of BPV to HOCl for 30 seconds resulted in rapid aggregation of these and other proteins on SDS PAGE gels.

**Conclusion:** HOCl proved to be a highly effective disinfectant even with short contact times, using the largest amount of mature infectious HPV16 and HPV18 possible. Rapid changes in capsid proteins may be responsible for the decline in infectivity. HOCl is not toxic on topical application to human skin and mucous membranes, raising the potential for its use not only as a disinfectant, but also in direct applications to human skin, oral and other tissues.

**Disclosure of Interest**: L. Robins: None declared, C. Meyers: None declared, J. Milici: None declared, J. Williams Employee of: Briotech Inc., R. Robison: None declared

### P484 ROOM DISINFECTION WITH UV-C LIGHT FOR VANCOMYCIN-RESISTANT ENTEROCOCCI – AN ADDITIONAL SAFETY? A PILOT STUDY.

#### A. Portmann, M. Dangel, A. F. Widmer

##### Division of Infectious Diseases & Hospital Epidemiology, University Hospital Basel, Basel, Switzerland

###### **Correspondence:** A. Portmann

**Introduction:** Surfaces may be contaminated by patients colonized or infected with multi-resistant germs. In order to avoid nosocomial infections, the rooms are undergoing a terminal disinfection (TD) when these patients are discharged. If the TD is inadequate, there is a risk of germ transmission to the next patient.

**Objectives:** Does UV-C disinfection of rooms occupied by patients colonized or infected with Vancomycin-resistant Enterococci (VRE) lead to a measurable decrease in pathogens compared to standard TD procedures?

**Methods:** Between October 2018 and April 2019, 20 rooms were examined at the University Hospital of Basel after discharge of patients with VRE. Eight sample sites have been checked: Toilet seat, toilet button flush, toilet paper cover, tap, floor, patient bed bell, bedside drawer and folding table. Standard TD was performed by a mix of quaternary ammonium compound with aldehyde (Deconex ® 50 FF, 0.5%).

The microbiological samples were taken using RODAC contact plates (Merck) and eSwab™ (COPAN) at three times: a) before TD, b) after TD and before UV-C disinfection, c) after UV-C disinfection. 0.2 ml of liquid were taken from eSwab™ and spread on CNA plates. Contact and CNA plates were incubated for 48 hours at 35°C. When growth was detected, isolates were subcultured on Columbia blood agar and CNA plates, and identified by MALDI-TOF. Susceptibility pattern was evaluated by VITEK. VRE was phenotyped followed by typing by Next-Generation Sequencing.

**Results:** Overall, 352 samples were analyzed. In one patient room, 8 samples could not be taken after TD and before UV-C. At time point a) 32 of 160, b) 4 of 152 and c) 0 of 160 samples were positive for VRE. Significant reductions were achieved before TD and after UV-C disinfection (p<0.0001) However, the addition of UV-C after TD did not reach statistical significance (p = 0.055). The number of VRE positive rooms was 55% (11/20) of all investigated rooms before TD among positive rooms, 36% (4/11) remained positive after TD. The addition of UV-C to TD lead to 0/11 positive rooms (p<0.01).

**Conclusion:** The applied TD failed to completely eliminate VRE. The additional exposure with UV-C succeeded to eliminate VRE from the environment.

**Disclosure of Interest**: None declared

### P485 QUANTITATIVE SUSPENSION TEST FOR THE EVALUATION OF BASIC BACTERICIDAL ACTIVITY OF DISEPT

#### A. Ziaeemehr^1^, K. Ghazvini^2^, M. Momen heravi^3^, M. malekmohamadi ^3^, F. Moavenian^1^

##### ^1^Surgical Oncology Research center, Mashhad University of Medical Sciences; ^2^Mashhad unioversity of medical sciences; ^3^Mashhad university of medical sciences, Mashhad, Iran, Islamic Republic Of

###### **Correspondence:** A. Ziaeemehr

**Introduction:** This study specifies a test method and the minimum requirements for basic bactericidal activity of Disept as a chemical disinfectant that form a homogeneous, physically stable preparation when diluted with water. Products can only be tested at a concentration of %80 or less as some dilution is always produced by adding the test organisms and water.

**Objectives:** This study only could apply to active substances (antibacterial biocides) and to formulations under development that are planned to be used in food, industrial, domestic and institutional, medical and veterinary areas. This study was done based on Iranian national standard (10504).

**Methods:** A sample of Disept as delivered (highest test concentration = 80%) is added to a test suspension of bacteria. The mixture is maintained at (20 ± 1) °C for 5 min ± 10 s (obligatory test conditions). At the end of this contact time, an aliquot is taken; the bactericidal and/or the bacteriostatic activity in this portion is immediately neutralized or suppressed by a validated method. The method of choice is dilution-neutralization. The numbers of surviving bacteria in each sample are determined and the reduction is calculated.

The test is performed using *Pseudomonas aeruginosa* and *Staphylococcus aureus* as test organisms (obligatory test conditions).

**Results:** In both the bacteria pseudomonas aeruginosa and staphylococcus aureous, the number of colony forming units per ml of test mixture was < 1.5 ×10^2^ and the number of living organism decreased to >10^5^

**Conclusion:** For the Disept E-1 as ready to use possesses bactericidal activity in the conditions of the test. The basic bactericidal concentration determined according to this study is ready to use for both test organism Pseudomononas aeruginosa and Staphylococcus aureus and showed a 5 log reduction or more at a this concentration.

**Disclosure of Interest**: None declared

### P486 SENSITIVITY AND SPECIFICITY OF CLASS-5 CHEMICAL INDICATOR TO DETECT STEAM STERILIZATION FAILURES IN PUBLIC HOSPITALS IN NEPAL.

#### G. Panta^1^, A. K. Richardson^2^, I. C. Shaw^3^

##### ^1^Save the Children International, Kathmandu, Nepal; ^2^School of Health Sciences; ^3^Department of Physical and Chemical Sciences, University of Canterbury, Christchurch, New Zealand

###### **Correspondence:** G. Panta

**Introduction:** Steam sterilization is the most commonly used method for sterilizing reusable medical devices in healthcare facilities. Biological indicators (BIs) are considered ‘gold standard’ for monitoring effectiveness of steam sterilization processes. However, because of associated costs and level of skills required, BIs are rarely used in low-income countries like Nepal. Class-5 chemical indicators could be an reasonable alternative to BIs, but their sensitivity and specificity to detect sterilization failures in a public hospital setting in Nepal are not known.

**Objectives:** To determine sensitivity and specificity of class-5 chemical indicator to detect steam sterilization failures in public hospitals in Nepal.

**Methods:** Of the 88 primary and secondary care public hospitals in Nepal, 13 hospitals were sampled randomly using stratified clustered design. In total, 189 steam sterilization cycles were tested for effectiveness using BIs (containing 10^6^ spores of *Geobacillus stearothermophilus*) and class-5 chemical indicators following manufacturers’ instructions. Test results of both the indicators were cross-tabulated and sensitivity and specificity of class-5 chemical indicators against BIs were determined. Effect of clustering and stratification of study units on outcome measures were considered when analyzing the data.

**Results:** The sensitivity and specificity of class-5 chemical indicator to detect steam sterilization failures were 95.3% (95% CI 81.0% - 99.0%) and 92.6% (95% CI 84.3% - 96.7%) respectively. The proportions of steam sterilization failures with the biological and class-5 chemical indicators were 71.0% (95% CI 46.8% - 87.2%) and 69.8% (95% CI 44.4% - 87.0%) respectively.

**Conclusion:** This study demonstrated a high sensitivity and a high specificity of class 5 chemical indicators to detect steam sterilization failures in public hospitals in Nepal. These indicators, which are generally cheaper than BIs, can be used routinely to monitor effectiveness of sterilization in these hospitals. The hospitals had a high proportion of stem sterilization failures and therefore, there is an urgent need for improving steam sterilization practices in these hospitals to minimize the risk of infections associated with inadequately sterilized medical devices.

**Disclosure of Interest**: None declared

### P487 INACTIVATION OF SCRAPIE PRIONS AND ANTIMICROBIAL PROPERTIES OF HYPOCHLOROUS AND HYPOBROMOUS ACIDS

#### L. Robins^1^, L. Contreras^1^, A. Hughson^2^, B. Caughey^2^, D. Terry^3^, J. Williams^3^

##### ^1^Physical Sciences Division, University of Washington Bothell, Bothell; ^2^Laboratory of Persistent Viral Diseases, Rocky Mountain Laboratories, NIAID, NIH, Hamilton, ^3^Briotech Inc. , Woodinville, United States

###### **Correspondence:** L. Robins

**Introduction:** Infectious prion proteins cause neurodegenerative diseases in animals and humans including Mad Cow disease and Creutzfeldt–Jakob disease, respectively. They have been shown to be transmissible via contaminated instruments, tools, and environmental surfaces and require strong and often hazardous conditions (e.g., 20-40% bleach) for disinfection and sterilization. Recently, we showed that hypochlorous acid (HOCl) causes rapid and high-level inactivation of infectious prions. However, hypobromous acid (HOBr) is known to be more potent than HOCl against some microbes including the polio virus. Both of these hypohalous acids are produced *in vivo* in response to pathogens.

**Objectives:** We characterized solutions of HOCl and HOBr for direct comparisons against scrapie prions and *Escherichia coli, Staphylococcus aureus*, and *Aspergillus niger.*

**Methods:** HOCl and HOBr were characterized using analytical spectroscopy and spectrophotometry. The hypohalous acids were tested against scrapie prions using the real-time quaking-induced conversion (RT-QuIC) *in vitro* system.

**Results:** HOCl at pH 4 showed no detectable changes after storage for months at 22 °C, and had surprising tolerance to heat stress, even at 70 °C. In contrast, HOBr degraded within hours to form mixtures of aqueous bromine species. Scrapie prion inactivation was similar for both HOCl and HOBr. Both hypohalous acids were tested against *Escherichia coli, Staphylococcus aureus*, and *Aspergillus niger* and the two differed demonstrably in their efficacies against the various microbes.

**Conclusion:** Therefore, occasions where shelf life and thermal stability are advantageous, HOCl would be the preferred disinfectant. Alternatively, HOBr tolerance of a wider range of pH conditions and superior efficacy against certain microbes may offer benefits in dealing with other decontamination challenges.

**Disclosure of Interest**: L. Robins: None declared, L. Contreras: None declared, A. Hughson: None declared, B. Caughey: None declared, D. Terry Employee of: Briotech Inc., J. Williams Employee of: Briotech Inc.

### P488 FAECAL CONTAMINATION AND THE QUALITY OF HOUSEHOLD DRINKING WATER IN NIGERIA : FINDINGS FROM THE MICS 2017

#### V. O. Pinheiro^1^, O. T. Olugbade^2^

##### ^1^Department of Jurisprudence and Inernational Law, University of Ibadan, Ibadan; ^2^Field Epidemiology Practice, Nigeria Field Epidemiology and Laboratory Training Programme (N-FELTP), Abuja, FCT, Nigeria

###### **Correspondence:** V. O. Pinheiro

**Introduction:** Access to clean uncontaminated water is a basic human right and a United Nations Sustainable Development Goal. Presence of Escherichia coli (E. coli) in water is an important indicator of fecal contamination.

**Objectives:** Using the MICS 2016/17 data we identified levels and risk of fecal contamination and factors associated with quality of household drinking water in Nigeria.

**Methods:** The MICS 2016/2017 was a national household-based cross-sectional survey. Information about the microbiological quality,availability and location of drinking water at both the source and household levels, was collected from 13,605 households.Presence of E. coli colony based on the colony forming units (CFU), was recorded and classified into low-risk (<1 per 100 mL), medium-risk(1-10 per 100 mL), high-risk (11-100 per 100 mL) and very high-risk (>100 per 100 mL).

**Results:** Overall,46.4% of the total household population surveyed had very high fecal contamination risk in source of drinking water,14.6% had high risk,16.3% had moderate risk,and 22.7% had low-risk.Households with highest risk had water from unprotected wells and springs{UWS}(85%); moderate risk from collected rain water{CRW}(22.5%);those at low risk had packaged and bottled water {PBW} (70%),as source.Fecal contamination risk in household drinking water was highest in water from UWS (84.3%),PWS (68.8%),CRW (47.7%) and tube well/bore hole{TWBH} 43.4%).Rain water (96.7%), and protected/ stream (90.2%),were the most accessible source of water to highest proportion of households.The TWBH (85.8%),CRW(83.5%)were the most available sources.Of households with unimproved sources of water, 88.5% do not treat their water,and only 2.4% boil.Highest availability in these households was CRW (96.7%),highest accessibility UWS (44%), only 2% of these sources were without E.coli.Factors associated with fecal contamination were uneducated household head,rural residence, poorest wealth quintile and exposure to untreated unimproved water sources (p value <0.05).

**Conclusion:** Fecal contamination risk is very high with overall low quality of household drinking water in Nigeria.There is poor accessibility, availability and affordability of water from improved sources.Health-education and empowerment-initiatives must address ignorance and poverty to prevent infections and outbreaks in order to break the chain of disease transmission.

**Disclosure of Interest**: None declared

### P489 “BEHIND THE SINKS”: ENDEMIC SERRATIA MARCESCENS IN A SWISS INTENSIVE CARE UNIT (ICU)

#### R. Martischang, V. Sauvan, S. Leo, V. Lazarevic, M.-N. Chraïti, Y. Martin, H. Soule, J. Schrenzel, Z. Koyluk Tomsuk, J. Pugin, S. Harbarth

##### Geneva University Hospital (HUG), Geneva, Switzerland

###### **Correspondence:** R. Martischang

**Introduction:** There is growing evidence of a nosocomial aquatic reservoir for *S.marcescens.* In summer 2017, the investigation of an outbreak at the adult ICU of HUG revealed endemic occurrence of wildtype *S.marcescens*. One third of patients had severe infection.

**Objectives:** To elucidate and control endemic *S.marcescens*.

**Methods:** Patient and environmental screenings were performed during summer seasons 2017 and 2018, targeting ICU patients, patient areas and sinks. 49 *S.marcescens* sampled at HUG between 2015 and 2017, including outbreak strains and 2 environmental isolates, were sequenced (2x300) using Illumina MiSeq 2x151, followed by cgMLST and phylogenic analysis. An audit monitored current sink-related care practices in the ICU.

**Results:** Sequencing revealed polyclonal outbreaks in 2017 and 2018, suggesting an external source. *S.marcescens* was isolated in two different sinks. One of these environmental isolates belonged to a cluster of 11 patients, of which 6 had staggered ICU stays between 2015 and 2017. The audit observed a proximity between sinks and washer-disinfectors, and temporary storage of potentially contaminated material near sinks. Based on molecular data and observed practices, *S.marcescens* was hypothesized to originate from sinks and to have contaminated patients through water exposure during their morning toilet. We recommended to change ICU siphons, coupled with regular sink disinfection through pressured steam. Toilet care was optimized before the implementation of waterless care for ICU patients, through single-use wipes for patient cleaning. The added annual expenditures of this strategy were estimated around 25K CHF. Since late 2018, the incidence density of nosocomially acquired *S.marcescens* isolates in the ICU has dropped.

**Conclusion:** The control of endemic *S.marcescens* is a challenging task and requires multimodal interventions, including the introduction of waterless patient care. According to cost-minimization analysis, waterless care might be cost-effective in an endemic situation, especially when considering the clinical impact of *S.marcescens*-related infection.

**Disclosure of Interest**: None declared

### P490 SINK-TRAPS AS A MAJOR SOURCE OF CPE TRANSMISSION; THE EXTENT OF THE PROBLEM

#### G. Regev-Yochay^1^, G. Smollan^2^, I. Tal^1^, N. Pinas Zade^1^, S. Segal^1^, H. Jaber^1^, G. Rahav^3^, E. Zimlichman^4^, N. Keller^2^

##### ^1^Infection Prevention & Control, ^2^Microbiology, ^3^Infectious disease, ^4^Sheba Medical Center, Ramat Gan, Israel

###### **Correspondence:** G. Regev-Yochay

**Introduction:** Sink-traps have been recognized as a potential source of Gram-negative MDRO outbreaks in ICUs.

**Objectives:** Here, we report widespread CPE contamination of sink-traps/sink-outlets in many departments of a large tertiary medical center, their role in CPE transmission among patients and our efforts to manage it.

**Methods:** During May 2017-Oct 2018, we screened sink-traps/sink-outlets in 34 departments of our tertiary medical center. 240 sinks were repeatedly screened on a weekly/monthly basis for >6m. Environmental samples were obtained by Copan Amies sterile transport swabs, and grown on Chromagar KPC plates. Carbapenemase genes were identified using PCR by the Xpert Carba-R cartridge (GeneXpert Cepheid). Genetic relatedness between CPE isolates from sinks and from patients was determined using Pulsed-Field Gel Electrophoresis (PFGE). Six different measures were tested in 70 persistently contaminated sink-traps (including replacement by copper sink-trap, self-disinfecting siphon, weekly acetic-acid treatment and others).

**Results:** A total of 631 sink-traps/sink-outlets were screened. CPE contaminated sinks were detected in 22 (65%) departments. In total, 20% of all screened sinks were contaminated with CPE. Recently renovated or newly built departments were typically spared from contamination, while older departments, with aged pipes were heavily contamination. Identical CPE strains between patients and sinks were detected in >50 cases, with various bacterial species and various carbapenemase genes. In 20% of these the temporal relation indicated sink to patient transmission. None of the intervention measures was fully successful in eliminating sink-trap contamination.

**Conclusion:** We report high levels of CPE contamination of sink-trap/outlets and show that these serve as source of CPE transmission, difficult to manage.

**Disclosure of Interest**: None declared

### P491 STILL THINK THAT HAND WASHING IS BEST – THINK AGAIN?

#### B. Shaik Ismail, M. L. Ling, J. H. Seah, H. X. Toh

##### Infection Prevention and Epidemiology, Singapore General Hospital, Singapore, Singapore

###### **Correspondence:** B. Shaik Ismail

**Introduction:** There has been increasing reports of sink contamination with multidrug-resistant gram-negative bacteria (GNB) globally. This poses a significant risk to patients especially severely immunocompromised patients such as neonates. Common pathogens seen in neonatal units are GNB from the *Enterobacteriaceae* family. In a neonatal unit in Singapore, during a cluster investigation of an extended-spectrum β-lactamase (ESBL)-producing *Escherichia coli (E.coli),* sink contamination from the *Enterobacteriaceae* family proved to be a persistent problem as well.

**Objectives:** This paper describes the containment of ESBL-producing *E.coli* cluster involving 2 neonates in a 28-bedded neonatal unit at an acute tertiary hospital in Singapore including the findings of sink contamination with ESBL-producing *Enterobacteriaceae* family that was done as part of the cluster investigation.

**Methods:** Sink sampling were collected from each sink's faucet, drainage and bottle trap. Other stringent infection control measures included contact tracing, eye equipment sampling, enhanced environmental cleaning and sink scrubbing, educational briefings, audits and strategic sink removals.

**Results:** A total of 23 sinks were sampled. Sixty nine samples were collected from each sink’s faucet, drainage and bottle trap. Of these, 27 samples yielded negative results. However the remaining 42 samples showed that all sinks had an organism growing either in the faucet, drainage or bottle trap. Twenty five samples yielded positive for environmental organisms such as *Stenotrophomonas maltophilia, Elizabethkingia* species, and organisms from the *Pseudomonadaceae* family. Seventeen samples yielded positive for GNB from *Enterobacteriaceae* family; 13 of which were ESBL-producing *Enterobacter cloacae* complex from the drainage and bottle traps found in the neonatal rooms, staff room, staff toilet, milk room and the dirty utility.

**Conclusion:** One of the lessons learnt during this cluster investigation is that sinks remain contaminated and acts as a potential reservoir despite housekeeping efforts with daily sink scrubbing in high risk areas such as the neonatal unit. In the future neonatal units, sinks should not be installed in the patient’s room. Instead this area should feature more use of alcohol-based hand rubs. Sinks, if required, should perhaps be installed outside of patient care areas such as the corridor or utility room in the unit.

**Disclosure of Interest**: None declared

### P492 ASSESSMENT OF BACTERIOLOGICAL CONFORMITY OF POINT-OF-USE WATER FILTERS AT 62 DAYS

#### M. Gallouche^1^, H. Majjaty^1^, P. Saviuc^1^, I. Pelloux^2^, M. R. Mallaret^1^, C. Landelle^1,3^

##### ^1^Infection control unit; ^2^Bacteriology laboratory, Grenoble Alpes University Hospital; ^3^TIMC-IMAG, Univ. Grenoble Alpes, CNRS, Grenoble INP, Grenoble, France

###### **Correspondence:** M. Gallouche

**Introduction:** Hospital water systems often contain waterborne pathogens and can become a source for healthcare-associated infections.

**Objectives:** Our aim was to assess the bacteriological conformity of 62-days water filters.

**Methods:** A study was conducted in a university hospital on 62-days point-of-use (POU) water filters. Three types of filters were considered: filters for shower heads (F1), spray head filters for taps (F2) and straight filters for taps (F3). Interventions were planned at t_0_ (when the filter was set up or just after the change of the filter, assuming that the filter conditions would be similar to those at the time of the previous change) and t_60_ (60 days after set up and before the change) and consisted of several actions, especially: swab sample (SS) of filter surface (t_60_), filtered water (FW) sample (t_60_), non-filtered water (NFW) sample after filter withdrawal (t_60_). Conformity at t_60_ was defined as follows: flora after incubation at 22°C ≤ 1UFC/100mL and *Pseudomonas aeruginosa* < 1UFC/100mL for FW and negative culture for SS. Data from routine surveillance of FW were used for interpretation and comparison between 31-days filters before 2017 and 62-days filters after 2017 (market change).

**Results:** Interventions took place on 60 filters for 62-days use (25 F1, 10 F2, 25 F3) in 12 units. Conformity at t_60_ was obtained for 39 filters (65%). Non-conformity for FW and/or SS was associated with NFW conformity for 11 POU (18%). In routine surveillance, FW conformity was 98% (88/90) over the period 2014-2016 (31-days filters only), 94% (16/17) in 2017 (switch to 62-days filters) and 83% (59/71) in 2018 (62-days filters only). The trend was statistically significant (p=0.004).

**Conclusion:** Non-conformity at t_60_ was identified for 21 filters for 62-days use, including probable retro-contamination in 11 cases, and we observed a deterioration of FW conformity in routine checks. Two solutions have been considered: reinforcing maintenance on 62-days filters or going back to 31-days filters.

**Disclosure of Interest**: None declared

### P493 RISK ASSESSMENT OF MICROBIOLOGICAL CONTAMINATION OF HEATER-COOLER UNITS FOR EXTRACORPOREAL CIRCULATION

#### B. Grandbastien, D. S. Blanc, M. Limet Dutoit, E. De Stefano, M. Kirsch, L. Senn

##### CHUV - centre hospitalier universitaire vaudois, Lausanne, Switzerland

###### **Correspondence:** L. Senn

**Introduction:** *Mycobacterium chimaera* infections have been linked to contaminated heater-cooler units (HCUs) during open-heart surgery. The Swiss health authorities, based on manufacturers’ recommendations, required monthly microbiological controls including nontuberculous mycobacteria (NTM).

**Objectives:** We carried out a risk assessment taking into account both the implementation of measures to prevent the risk of aerosolization around the HCU and the impact of a withdrawal of contaminated HCUs.

**Methods:** In our Center, 4 HCUs (3 LivaNova 3T and one HCU30 Maquet) are used. So far, no patients have been identified with *M. chimaera* infection. Disinfection and maintenance are performed according to manufacturers' recommendations. HCUs water is sampled every month and analyzed for the total viable count of the mesophilic flora, the presence of *M. chimaera*, *Legionella* sp. and of *Pseudomonas aeruginosa*. Actions in case of unsatisfactory bacteriological results were formalized in a risk assessment that has been validated by our institution in October 2018. We proposed a double disinfection in case of massive contamination and only the persistence of contamination implicated the withdrawal of the HCU and its return to the manufacturer.

**Results:** Between April 2017 and March 2019, 61 water samples were analyzed. Mesophilic flora >2000 CFU/ml was frequently found (44%). No sample has ever identified *P. aeruginosa* or *Legionella sp*; however, for 14 samples (23%), this analysis was impossible due to predominant mesophilic flora. Before the implementation of our risk assessment, 3 LivaNova and one Maquet HCUs were found positive for *M. chimaera*: all were returned to the manufacturer. Since the implementation of our risk assessment and the double disinfection process, neither persistent mesophilic flora nor *M. chimaera* was found.

**Conclusion:** Prevention of *M. chimaera* risk requires measures in order to control HCUs aerosols, disinfection and water surveillance. A double disinfection seems to be suitable to disinfect effectively the HCUs without the need to return the HCU to the manufacturer. A simplification of microbiological controls could target exclusively NTM.

**Disclosure of Interest**: None declared

### P494 EXPANSION OF ULTRASOUND IN HEALTHCARE: CHALLENGES AND SOLUTIONS FOR INFECTION PREVENTION

#### R. Carrico

##### Infectious Diseases, School of Medicine, University of Louisville, Louisville, United States

**Introduction:** Ultrasound has become an increasingly useful clinical tool over recent decades to the point that it is now used in almost every healthcare department. While this uptake has brought great benefit to patients, infection risks have been documented and guidelines developed to improve patient safety.

**Objectives:** To i) analyze evidence of infection risk associated with use and reprocessing of ultrasound probes; ii) identify guidelines related to endocavitary and surface ultrasound; iii) address knowledge gaps in specific areas of practice.

**Methods:** Systematic review of peer-reviewed literature and regulator alerts identified infection risks associated with ultrasound. Guidelines were compared to identify the global consensus on reprocessing which was contrasted with published surveys on actual practice. After identification of knowledge gaps around interventions, a systematic search of online content was conducted to determine the frequency of contact between probes and different tissues (e.g., sterile, intact skin) in common procedures.

**Results:** Risks associated with ultrasound use were identified including cases of infection which have resulted in regulators setting new reprocessing standards, ordering reviews of infection prevention practices and triggering guideline development. The consensus among guidelines is that probes contacting mucous membranes or non-intact skin must undergo HLD, and critical probes that contact sterile tissue require sterilization or if not possible, HLD with use of a sterile sheath. Surveys revealed variable practice for ultrasound-guided procedures. Analysis of 114 online educational videos across 6 common percutaneous interventions found 50% of probes directly contacted sterile tissue at the puncture site with a further 30% <10mm away.

**Conclusion:** Our literature analysis identified infection risks associated with ultrasound use and reprocessing. Though there are guidelines, surveys show high levels of non-compliance. Ultrasound-guided percutaneous interventions were found to be one of the fastest growing applications of ultrasound with a knowledge gap in infection risk. Risk to patients due to improper reprocessing is non-discriminatory and applicable worldwide. Facilities should review their use of ultrasound probes and address any gaps to ensure patient safety.

**Disclosure of Interest**: R. Carrico Consultant for: Nanosonics

## Poster session: ICPIC Clip Award

### P495 WASHING HAND FOR SAVING HUMAN LIFE

#### H. T. Phan

##### Hung Vuong hospital, Ho Chi Minh, Viet Nam

**Content of this video**

The content of this video describes two lives: One is the life of Dr Ignace Philippe Semmelweis, a selfless and ardent person and very responsive to his patients; second is the lives of women died due to postpartum fever. The generosity and progressive spirit of Dr Philippe Semmelweis helped him overcome the selfishness and jealousy of his colleagues to bring peace and life to pregnant women by hand washing method with an effective, but inexpensive chlorine solution.

He has surpassed the fear of a man's instincts when cornered, and replaced it with the courage to do the right thing to save his patients: hand washing for saving a life. The end of the video is a grave, and he is also the holder of his cross. This message suggests that the cross is for everyone.

The cross will bring glory when we transform the cross, which is adversity, becomes glory and joy. Transform our cross to become joy, peace for others. Dr Philippe Semmelweis transform his cross to be peace for women and her children. When he died and put a cross on his own grave, it is the time others put flowers on his grave. The final message of the video is " The love what he offered his patients is great. " Dr Philippe Semmelweis has done that.

**Objective of this video**

The objective of this video is to raise the importance of hand hygiene. It showed it is dirty hands of doctors and nurses leading to the death of pregnant in Dr Philippe Semmelweis’ age. So, in a Maternity Hospital, as Hung Vuong hospital, hand hygiene is extremely crucial because it will prevent hospital-acquired infections such as neonatal sepsis, pneumonia, surgical site infection... Hygiene will help to reduce the prevalence of carbapenemase-producing organism due to cross –infection from mother to neonates. Hand hygiene does not take much time and cost, but its results bring to be happiness, safety for the pregnant and neonates.

Therefore, health workers should perform hand hygiene before and after taking care of pregnant women, neonates to save lives and to provide safe care.

**Disclosure of Interest**: None declared

### P496 MELODY ON HAND HYGIENE INSPIRES THE PEOPLE OF VIETNAM

#### T. A. T. Le

##### Ho Chi Minh City Society, Ho Chi Minh, Viet Nam

**Abstract video clip:** Ho Chi Minh City Infection Control Society (HICS) was established in 2008 and has grown beyond 1000 members today. HICS has built and promoted infection control as a daily activity in healthcare facilities to improve quality of patient care. HICS aims to ensure hospital environmental safety, to prevent cross infections in Vietnamese hospitals, and to achieve the goal of protecting and caring for people’s health. HICS also provides authorities with consultation, staff training, and policy development regarding hospital infection control.

Raising awareness on hand hygiene is one of the most essential programs that HICS focuses on. Inspired by the official song for the World Hand Hygiene Day 2019, HICS translated this melody into Vietnamese to carry the message “Clean care for all – It’s in your hands” deeply into the people heart. The message spreads to all hospitals from the North to the South, from the mountain to the plain. It inspires all people, from doctors, pharmacists, nurses, technicians, patients to their relatives and the community as a whole. The message motivates everyone to take action to protect the communities and improve the safety of hospitals.

**Disclosure of Interest**: None declared

### P497 CELEBRATION OF THE INTERNATIONAL HAND HYGIENE DAY - MAY 2019

#### J. Tannous, N. K. Zahreddine, R. Ahmadieh, T. Kardas, A. Ibrahim, S. Kanj

##### Infection Prevention and control, AUBMC, Beirut, Lebanon

###### **Correspondence:** J. Tannous

**Abstract video clip:** Each year in May, the Infection Control and Prevention Program (ICPP) team at the American University of Beirut Medical Center (AUBMC), Lebanon celebrates the International Hand Hygiene (HH) Day.

This year, "Clown Doctors" joined us to add spice and fun to the celebrated educational activities.

Healthcare Workers (HCW) , visitors and patients joyfully participated and cleaned their hands using the advertized contact time and techniques.

During that day, the clown doctors imitated HH anonymous auditors in a theatrical manner.

The first part of the video featured the most common breaches that were previously identified by the ICPP team such as improper HH technique and timing as well as touching the contaminated door knobs with cleaned hands. Later on, the proper technique of HH was performed between both clown doctors.

The 5 hand hygiene moments, techniques, and contact time were entertainingly featured and promoted by the clown docotrs to raise awareness about the HH importance in preventing transmission of infections as well as harmful pathogens among patients, HCW and visitors.

"Clean Care for ALL - It's in your Hands" theme was printed on the shirts worn by the ICPP team who accompanied the clown HCW during the celebrations throughout the Medical Center.

This entertaining video clip will be used by ICPP as part of the annual mandatory Infection Control educational sessions given to all hospital staff at AUBMC.

**Disclosure of Interest**: None declared

### P498 IT CAN PREVENT SEPTICEMIA AT HEALTH CARE SETTING. IT IS IN YOUR CAPACITY

#### H. T. Phan

##### Hung Vuong hospital, Ho Chi Minh, Viet Nam

**Abstract video clip:** The content of this video includes the activities described as following; Firstly, Director of Hung Vuong Hospital states the importance of hand hygiene and launches the hand hygiene program for all the hospital. Then, the Board of Directors signed a commitment of hand hygiene with the Heads of Department. Continuously, the flashmob shows the battle between health care workers and bacteria. Through hands, hand hygiene brings a smile, then protect life for mother and baby. Next, a commitment of hand hygiene, which is performed by the fingerprints of the Board of Directors, Heads of Departments, includes the Board of Hospital Directors, Heads of Departments and patients. In addition to, activities of transferring alcohol hand rub bottle to the next person seem like an effective solution for encouraging hand-hygiene, and after that, all employees are present in the festival. Moreover, The Broad of Director's organizers gives Lucky gifts (bubble rain) to health care workers, patients' relatives. Finally, the Board of Directors, health care workers and patients take the photos at the photo corner decorated at flower gardens and departments

**Objective of this video**

The objectives of this video clip are that the Board of Directors launches a hand hygiene movement in the whole hospital and the participants in hand hygiene include healthcare workers, patients and patient relatives. Therefore, the objectives of this video clip are to re-affirm:

1. Hand hygiene is a mandatory medical practice that healthcare workers must practice properly

2. Provide proper knowledge and practices of hand hygiene for patients and patients' relatives comprehending the benefits of hand hygiene: preventing and avoiding contagious diseases caused by dirty hands.

3. At the same time, emphasizing the rights of patients and relatives is a reflection of cases where health workers do not practice hand hygiene when performing procedures for patients.

**Disclosure of Interest**: None declared

### P499 SURVEILLANCE REGARDING KNOWLEDGE AND ATTITUDE REGARDING CARE OF HEPATITIS B VIRUS (HBV) POSITIVE PATIENTS AND VACCINATION STATUS AMONGST THE NURSES

#### S. Kaur, S. Kaur, R. K. Dhiman, A. Duseja, P. Arora

##### National Institute of Nursing Education, Post Graduate Institute of Medical Education and Research Chandigarh, Chandigarh, India

###### **Correspondence:** S. Kaur

**Introduction:** Nurses are at high risk of acquiring and transmitting the blood borne infections. They need to be aware regarding the prevention and spread HBV infection.

**Objectives:** To assess the knowledge and attitude regarding care of Hepatitis B Virus positive patients and hepatitis B vaccination status amongst the nurses.

**Methods:** A total of 400 bedside nurses working in various areas viz Emergency Unit, Surgical Unit, Operation Theatre, ICUs, Obstetrics Unit, Medicine Unit and Dialysis unit were studied. Around 50% nurses working in each said area were taken up for the study. A self administered questionnaire with questions on knowledge, attitudes, and practices regarding HBV was used to collect the data.

**Results:** The overall mean knowledge score regarding hepatitis B was 25.7±1.64 (56%). As per the various categories of the disease, mean percentage score was maximum (80%) regarding diagnosis of hepatitis B followed by Management of Hepatitis B and mode of transmission with almost equal percentage score (71.7% and 70%) respectively. The minimum score was regarding knowledge regarding vaccination of hepatitis B and clinical symptoms. Regarding the attitude of the nurses regarding care of HBV positive patients, 43% strongly agreed and 38% agreed that their job puts them at risk for Hepatitis B. Around one fourth each strongly agreed and agreed that these patients should be isolated from other patients Regarding opinion about Hepatitis B vaccination, 67.2% strongly agreed that Hepatitis B vaccine should be compulsory for all the health workers. Regarding vaccination, only half had taken all the three doses of hepatitis B.

**Conclusion:** The nurses need to be sensitized periodically to update their awareness regarding care of the HBV positive patients.

**References**

1. Mehriban N, Ahsan GU, Islam T. . Knowledge and preventive practices regarding hepatitis B among nurses in some selected hospitals of Dhaka city, Bangladesh. South East Asia Journal of Public Health 2014;4(1):48-52.

2. Abdulla SA, Abdulla Z. Effect of an educational program on nurses' knowledge and practices toward Hepatitis B virus in emergency hospitals in Erbil City. Zanco J. Med. Sci 2014; 18(1): 618-24

**Disclosure of Interest**: None declared

### P500 THE HUMANITARIAN CITY

#### M. M. Solatorio, on behalf of Infection Prevention and Control Team, Environmental Services Department, Nursing Services Department, Public Relation, Quality Management, Rehabilitation Services Department

##### Quality Management, Sultan Bin Abdulaziz Humanitarian City, Riyadh, Saudi Arabia

###### **Correspondence:** M. M. Solatorio

**Abstract video clip:** The role of the Infection Prevention and Control Team is to ensure that the risk of infection to patients, visitors and staff is minimized through a range of prevention and control processes.

Every day, patients get infections in healthcare facilities while they are being treated for something else. These infections can have devastating emotional, financial, and medical effects. Worst of all, they can be deadly.

Campaigning is one important part of reaching people, improving behaviour and achieving safer, high quality health care practices.

Antimicrobial stewardship (AMS) is the systematic effort to educate and persuade prescribers of antimicrobials to follow evidence-based prescribing, in order to stem antibiotic overuse and thus antimicrobial resistance.

For the desired antimicrobial use, goals need to be formulated:

- Define "appropriate", rational antimicrobial use for the institution, individual patient units, and define empiric treatment versus culture-directed antimicrobial treatment.
- Establish treatment guidelines for clinical syndromes. These can be disseminated in the form of memos, in-services or grand rounds and may be most effective in the form of decision making tools at the point of ordering the prescription.

Care is provided in a wide range of settings including a person’s own home, hospital day and inpatient units and long term care facilities. Inpatient/care home settings can provide ideal conditions for micro-organisms to be transferred between those who receive and give care. The close proximity and frequent physical contact in a shared working and living environment all contribute to increased risk of transmission.

No patient should come to harm under our care, so keeping them safe at all times is our top priority.

**Disclosure of Interest**: None declared

### P501 VIDEO CLIP OF E LEARNING OF HAND HYGIENE: CAMPAIGN "WE WILL BE CLEAN AND HEALTHY IF WE WASH OUR HANDS"

#### L. Mazzillo Vega

##### CRITICAL CARE, HOSPITAL INFANTIL LOS ANGELES, PASTO, Colombia

**OBJECTIVE:** Sensitize ,motivate and train health personnel on the importance and technique of hand washing to improve compliance of hand hygiene

**Introduction:** Every year thousands of patients acquire bacteria that cause infections associated with health care that increase morbidity and mortality and the cost of care. These bacteria are transmitted by the hands of health personnel and improve the compliance to hand hygiene in the 5 moments of care with an appropriate technique protects patients and the health workers to acquire these infections.

**Methodology:** Project is carried out with a logical framework methodology to implement antimicrobial stewardship

The projects was to design and learning where hand hygiene videos were included

It is implemented E learning hand hygiene in 2017 where staff is trained on hand hygiene benefits and the results of improving the adherence to it for this virtual course several videos are produced to achieve the objectives described above

**Results:** They have made and approved the virtual course. 196 people years 2017-2018

We have presented improvement to the adherence to hand hygiene year 2016 92%, year 2017 93 % , year 2018:94%

The overall rate of infections has decreased. Year 2016: 0.92, year 2017: 0.71 and year 2018: 0.37.

Conclusions:

1. The learning strategy improves the adherence to hand hygiene in our institution.

2. By increasing the adherence to hand hygiene the overall rate of infections is reduced.

Bibliography:

1. Allegrancy B, Pitet D. Rol of Hand Hygiene in healthcare associated infection prevention, the J Hosp Infect. 2009;73;4:305-315

2. Salama M, Jamal W, Al Mousa H,et al. The effect of Hand Hygiene compliance on Hospital-acquired infections in a ICU setting in a kuwaiti teaching hospital.2013;6;1:27-34

**Disclosure of Interest**: None declared

### P501b MONITORING HAND HYGIENE WITH SENSOR TECHNOLOGY "NOSOEX"

#### T. Gebhardt

##### GWA Hygiene, Stralsund, Germany

###### **Correspondence:** T. Gebhardt

**Abstract video clip:** Hospital Lüneburg is the pioneer for our hand hygiene monitoring "NosoEx". The video shows how our technology is used in this German hospital. The infection control officer provides insights from the pilot project. The disinfectant use has been increased by 35%. That growth was important because the dispensed amount has been below the average on certain hospital wards. Moreover, feedback regarding the hand hygiene behavior can be provided more targeted due to the job group based monitoring. Exemplary groups can be physicians, nurses and therapists. Consequently, individual healthcare-workers are not monitored what makes NosoEx data protection law compliant. Furthermore, the monitoring of the dispensers is based on sensors that are added to the existing dispensers (retrofit approach). To sum it up, NosoEx can be seamlessly integrated in the infrastructure and routines of the hospital.

**Disclosure of Interest**: None declared

## Poster session: Chlorhexidine-based antisepsis

### P502 THE BENEFIT OF PREOPERATIVE WASHING WITH CHLORHEXIDINE GLUCONATE-IMPREGNATED CLOTHS ON THE INCIDENCE OF SURGICAL SITE INFECTIONS: A SYSTEMATIC REVIEW

#### V. Forget^1^, O. Azzam^2^, C. Terreaux-Masson^2^, M.-R. Mallaret^2^, C. Khouri^2^, C. Landelle^2^

##### ^1^Infection Control Unit, Centre hospitalier metropole savoie, Chambery; ^2^Infection Control Unit , Grenoble Alpes University Hospital, Grenoble, France

###### **Correspondence:** V. Forget

**Introduction:** Preoperative washing with plain or antimicrobial soap is recommended by the World Health Organization (WHO) for the prevention of surgical site infections (SSI). WHO did not formulate recommendation on the use of chlorhexidine gluconate (CHG)-impregnated cloths because of limited and low quality evidence.

**Objectives:** The purpose of this systematic review was to evaluate the benefit of preoperative washing with CHG-impregnated cloths on the incidence of SSI.

**Methods:** The PICO methodology was used. Randomized controlled trials (RCT), quasi-randomized, case-control and cohort studies on patients with surgery (Population) having preoperative washing by CHG cloths (Intervention) or antiseptic soap, plain soap, placebo, no washing, no washing instruction (Comparator) were included. The principal Outcome was the SSI occurrence. Publications were searched on Medline, CENTRAL, Web of Science, Clinical Trial between 01/01/1990 and 30/06/2018. The quality of the studies was assessed with the Cochrane and Newcastle-Ottawa tools, the quality of evidence with the GRADE method. Statistics were performed on RevMan5.3.

**Results:** A total of 1108 publications were identified, 3 were included: 1 RCT and 2 prospective cohort studies. The meta-analysis of the 2 cohort studies comparing the effect of preoperative bathing with CHG-impregnated cloths the evening and the morning before intervention to noncompliance with preoperative washing showed an Odds-ratio (OR) equal to 0.25 (95%CI [0.13-0.50], p<0.0001). The RCT showed an OR equal to 0.12 (95%CI [0.02-1.00], p=0.05) for CHG-impregnated cloths the evening and the morning before intervention versus a shower with antibacterial soap the evening before the intervention.

**Conclusion:** Studies quality was high but concern orthopedic surgery only. The available studies show a benefit for CHG-impregnated cloths on SSI occurrence, but without comparison with usual practices (antiseptic or plain soap washing the evening and/or the morning before intervention). Further studies are needed to confirm the CHG-impregnated cloths benefit for the preoperative washing.

**Disclosure of Interest**: None declared

### P503 THE USE 2% CHLORHEXIDINE GLUCONATE IMPREGNATED CLOTH X 2% CHLORHEXIDINE GLUCONATE LIQUID AS REDUCTION OF SKIN MICROORGANISMS COUNT: INTEGRATIVE REVIEW

#### F. D. O. Andrade^1^, V. D. B. Poveda^2^, R. T. N. Turrini^3^

##### ^1^Nursing, Hospital of Clinic of Federal University of Parana, Curitiba; ^2^Nursing, School Nursing of University of Sao Paulo; ^3^Nursing, School of Nursing of University of Sao Paulo, Sao Paulo, Brazil

###### **Correspondence:** F. D. O. Andrade

**Introduction:** Surgical site infections are considered one of the largest and most important postoperative complications, with preoperative bath being one of the stages of surgical preparation and aimed at reducing surgical risk by reducing microbial counts of the skin, acting as a coadjuvant in the prevention of infection of the surgical site.

**Objectives:** To compare and evaluating the efficacy of 2% chlorhexidine gluconate-impregnated cloth and pre-operative 2% chlorhexidine gluconate liquid in reducing the microorganisms of the skin in adults.

**Methods:** This is an integrative literature review, performed through the databases: Virtual Health Library, Cranial, Cochrane, Embase, LILACS, PubMed and Scopus, using the keywords: antisepsis, chlorhexidine and preoperative period.

**Results:** 177 studies were included, of which 8 were included, observational or experimental, being 3 (37.5%) meta-analysis, 4 (50%) cohort, 1 (12.5%) randomized clinical trial, published between 2013 and 2017, in the English language, produced in the United States. In the analyzed studies, participants who used 2% chlorhexidine gluconate-impregnated cloth a greater reduction in the number of microorganisms in the skin compared to participants who used 2% chlorhexidine gluconate liquid. The authors note that this fact may be related to the persistence of 2% chlorhexidine gluconate in the skin, when used in the form of impregnated cloth.

**Conclusion:** Considering the infection of the surgical site as responsible for high morbidity and mortality and that the microbiota of the patient's skin is an important source for the development of this complication, the preparation of the skin should be the focus of attention of the health team. Therefore, new health care technologies need to be evaluated in relation to their cost-effectiveness and compared to existing practices, allowing health professionals to provide safer preoperative care.

**Disclosure of Interest**: None declared

### P504 EFFECTIVENESS OF DAILY CHLORHEXIDINE BATHING TO REDUCE HOSPITAL ACQUIRED INFECTIONS AND COLONIZATIONS IN THE INTENSIVE CARE UNIT OF A TERTIARY CARE CENTER IN LEBANON

#### J. Tannous, N. K. Zahreddine, A. Ibrahim, R. Ahmadieh, T. Kardas, S. Kanj

##### ^1^Infection Prevention and control, AUBMC, Beirut, Lebanon

###### **Correspondence:** J. Tannous

**Introduction:** Preventing Hospital Acquired Infections is a major safety challenge involving labor-intensive efforts in intensive care units (ICUs).

**Objectives:** The purpose of this study was to evaluate the effectiveness of daily bathing with Chlorhexidine 4% (CHG) in controlling the spread of Multidrug-Resistant Acinetobacter baumannii (MDR-Ab) as well as reducing Device Associated Infections (DAI). This intervention was accompanied by other essential Infection Control (IC) measures such as adherence to hand hygiene, care bundle, and contact precautions.

**Methods:** A new protocol for CHG daily bathing of patients was introduced in 2016 in response to an outbreak of MDR-Ab in 2015 in the medical/surgical 12-beds ICU at the American University of Beirut Medical Center. The prospective study period was from January 2015 to December 2018. Nursing team was reeducated and trained on daily CHG bathing of all patients admitted to ICU. The yearly transmission rates of MDR-Ab and the colonization pressure (CP) were respectively estimated as new transmissions per 1000 patient-days (PD) and MDR-Ab PD per 1000 ICU PD. The definition and analysis of DAI was based on the CDC/NHSN (Centers for Disease Control and Prevention/National health and Safety Network).

**Results:** A significant decrease (p-value<0.0001) in both MDR-Ab transmission rates and CP was noted in ICU between 2015 (16.0‰ and CP 339.3‰) and 2018 (3.6 ‰ and CP 112.7‰). Ventilator Associated Events (VAE) significantly decreased (p-value<0.0001) from 7.8 ‰ device days (DD) to 0.7‰ DD. Catheter Associated Urinary Tract Infections (CAUTI) significantly decreased (p-value=0.01) from 5.2 ‰ DD to 1.4‰ DD. Central Line Associated Bloodstream Infections (CLABSI) decreased from 2.0 ‰ DD to 0.8‰ DD.

**Conclusion:** The significant decrease was commensurate with the overall compliance with IC measures during the study period. It entailed dedicated education and training of the nursing staff supplemented by other costly and labor-intensive practices. Our study supports daily CHG bathing in a setting where all other IC measures cannot be disregarded. However, skin eradication of MDR-Ab from colonized patients remains a challenge in healthcare settings.

**Disclosure of Interest**: None declared

### P505 EFFECT OF DAILY BATHING WITH CHLORHEXIDINE ON HEALTHCARE ASSOCIATED INFECTIONS CAUSED BY CARBAPENEMS RESISTANT GRAM-NEGATIVE BACTERIA

#### W. Huang, F. Qiao, L. Wei, J. Lin, Z. Zong

##### Infection Control Department, West China Hospital, Sichuan University, P.R.China, Chengdu, China

###### **Correspondence:** W. Huang

**Introduction:** Daily bathing with chlorhexidine gluconate (CHG) of intensive care unit (ICU) patients has been shown to reduce healthcare-associated infections (HAIs) caused by multidrug resistant organisms (MDROs). However, reports from developed countries such as USA and Europe concerns about CHG reduced MRSA and VRE mostly, and rarely about Carbapenems resistant gram-negative bacteria such as Carbapenems resistant *Acinetobacter baumannii* (CRAB), Carbapenems resistant *Pseudomonas aeruginosa* (CRPA) and Carbapenem resistant *Enterobacteriaceae* (CRE), which are the dominant MDROs in China.

**Objectives:** To evaluate the effect of daily CHG bathing on the incidence rate of HAIs caused by CRAB, CRPA and CRE in an ICU ward.

**Methods:** This was a pre-post with comparison type of quasi-experimental intervention design comparing a 6 months pre-intervention period (July 2015–December 2015), during which inpatients of the ICU ward were bathed with regular liquid soap, with an equally long intervention period (July 2016–December 2016), during which inpatients of that ward were routinely bathed with 2% CHG. At the same time, we chose medical wards and Surgical wards without CHG use as parallel controls to show the effect of CHG by difference-in-difference model.

**Results:** A total of 1009 patients were admitted to the unit during the intervention period, and 827 (81.9%) were bathed with CHG. Incidence density of CRAB decreased from 7.5 (95% CI: 5.7-9.3 per 1,000 patient days) to 6.0 (95% CI: 4.4-7.6 per 1,000 patient days) (P<0.01). Incidence density of CRPA decreased from 4.6 (95% CI: 3.3-6.3 per 1,000 patient days) to 2.5 (95% CI: 1.6-3.8 per 1,000 patient days) (P<0.01). There was no significant difference in incidence density of CRE between the two periods (0.5 vs 0.6 per 1,000 patient days (P=0.25).

**Conclusion:** Daily bathing with CHG reduces the incidence rate of HAI caused by CRAB and CRPA in an ICU ward.

**Disclosure of Interest**: None declared

